# The Golden Section as Optical Limitation

**DOI:** 10.1371/journal.pone.0131045

**Published:** 2015-07-08

**Authors:** Mark A. Elliott, Joy Kelly, Jonas Friedel, Jennifer Brodsky, Paul Mulcahy

**Affiliations:** 1 School of Psychology, National University of Ireland Galway, Galway, Republic of Ireland; 2 Department of Psychology, University of Salzburg, Salzburg, Austria; 3 Department of Psychology, Union College, Schenectady, New York, United States of America; Medical University of Graz, AUSTRIA

## Abstract

The golden section, ϕ = (1 + √5)/2 = 1.618… and its companion ϕ = 1/ϕ = ϕ -1 = 0.618…, are irrational numbers which for centuries were believed to confer aesthetic appeal. In line with the presence of golden sectioning in natural growth patterns, recent EEG recordings show an absence of coherence between brain frequencies related by the golden ratio, suggesting the potential relevance of the golden section to brain dynamics. Using Mondrian-type patterns comprising a number of paired sections in a range of five section-section areal ratios (including golden-sectioned pairs), participants were asked to indicate as rapidly and accurately as possible the polarity (light or dark) of the smallest section in the patterns. They were also asked to independently assess the aesthetic appeal of the patterns. No preference was found for golden-sectioned patterns, while reaction times (RTs) tended to decrease overall with increasing ratio independently of each pattern’s fractal dimensionality. (Fractal dimensionality was unrelated to ratio and measured in terms of the Minkowski-Bouligand box-counting dimension). The ease of detecting the smallest section also decreased with increasing ratio, although RTs were found to be substantially slower for golden-sectioned patterns under 8-paired sectioned conditions. This was confirmed by a significant linear relationship between RT and ratio (p <.001) only when the golden-sectioned RTs were excluded [the relationship was non-significant for the full complement of ratios (p = .217)]. Image analysis revealed an absence of spatial frequencies between 4 and 8 cycles-per-degree that was exclusive to the 8-paired (golden)-sectioned patterns. The significance of this was demonstrated in a subsequent experiment by addition of uniformly distributed random noise to the patterns. This provided a uniform spatial-frequency profile for all patterns, which did not influence the decrease in RT with increasing ratio but abolished the elevated RTs to golden-sectioned patterns. This suggests that optical limitation in the form of reduced inter-neural synchronization during spatial-frequency coding may be the foundation for the perceptual effects of golden sectioning.

## Introduction

The golden section or ϕ (phi) is an irrational number, which in conjunction with π and e are determined to be one of the most significant constants in mathematics. Amongst other properties ϕ describes the relationship of numbers in the Fibonacci sequence as well as the geometry of pentagrams, in which each intersection of edges sections other edges in the golden section. While the term ‘Golden Section’ (goldener Schnitt) is ascribed to a text written by Martin Ohm in 1835 [1], knowledge of its mathematical significance is much older. The number was originally defined by early Greek mathematicians including Pythagoras, for whom it may have been instrumental in the discovery of irrational numbers by virtue of its application to geometrical structure [2]. Expressed formally, the relation *r* = a/b = b/(a + b) permit only two possibilities for *r*: *ϕ* = (√5–1)/2 = 0.618… and -ϕ = -(1 + √5)/2 = -1.618…, which is the geometric definition of the golden section.

The golden section is commonly believed to be present in ancient architecture, for example the Parthenon [3] and Egyptian religious buildings [4]. In antiquity, design employing golden sectioning is believed to generalize to other structures: 2,500 year-old Greek kantheroi have been argued to incorporate the golden section in their design [5]. There is also evidence for design employing golden sectioning in which the mean length/width ratios of 7,000-year old projectile points were found to vary in very close agreement with the ϕ (upper and lower 95% confidence intervals 1.59–1.64) [6].

While compelling, there are convincing arguments that claims of golden sectioning in almost all cases involve seemingly arbitrary calculations of the actual dimensions of the structures concerned [2]. In contemporary thinking the golden section is associated with aesthetics: in the 16^th^ century, *De Divina Poportione* [7] proposed ϕ to be the fundamental unit in artistic perspective with application to architectural design. In the mid-19^th^ century it was suggested that many works of art include golden sectioning in their composition [8,9]. These claims lead the founder of modern psychophysics, Gustav-Theodor Fechner, to examine the aesthetic properties of simple rectangular figures, and to report a clear preference for golden-sectioned rectangles [10]. However, in the 150 years since Fechner’s work there are an almost equal number of studies claiming support for Fechner’s finding as there are studies finding no preference. This lack of agreement may be based upon misunderstandings or misreading of the early experimental protocols, as well as imprecise replications of experimental conditions [11]. However, the lack of consensus has lead to claims that there is no evidence to support the idea that golden sectioning is an important principle in art and structural design [2].

Phi does appear in natural growth patterns, such as flower and leaf arrangements that develop pentagonal symmetry. The morphologies of a variety of sea creatures adapt to golden sectioning, for instance, the logarithmic spiral that is constructed from golden rectangles characterizes the shells of Nautilus, Abalone and Triton [4,11]. Another example is the “golden-angled” (137.5°) spiral configurations of pineapples, sunflowers, pinecones, cauliflowers, etc. Neither of these examples are numerological coincidences, but rather adaptations following ϕ as a physical constraint. Seashells that follow this growth pattern are able to maintain their shape while growing in size, and petal growth sequences can maximize their individual exposure to the environment by having this arrangement, in both cases producing a visual appearance that is self-similar across spatial scales [11].

It has recently been argued that in the human EEG, different neural oscillation frequencies will never synchronize (in a mathematical sense) if the frequency relationship is close to ϕ: mathematical modeling indicates that frequencies separated by close to ϕ exhibit the most irregular pattern of interaction possible between the excitatory phases of two or more independent sources of neuronal activity [12].

Neural oscillation frequency is believed to be important because it is associated with the rhythmic synchronization of neuronal firing, which in turn is believed to facilitate the formation of functional neuronal assemblies [13–15]. In the EEG literature, neural oscillations are divided into different frequency bands according to associated cognitive function: for instance, frequencies in the EEG beta (4–7 Hz) and alpha (8–12 Hz) bands are associated with working memory and attention while those in the EEG gamma band (30–70 Hz) are associated with functions that include long-term memory storage and retrieval, as well as perceptual processing [16–18]. It is also believed that cross-frequency phase synchronization is necessary for the functional integration of assemblies undertaking different cognitive tasks [19–21]. Assuming the model offered in [12] is of significance to functional integration, it would entail that for neural assembles oscillating at frequencies separated by ϕ, for example two assemblies oscillating at 50 and 31 Hz (1:0.613), there would be little or no functional interaction between the two assemblies and therefore no cognitive function associated with their co-activation.

This may come about as a property of ϕ as the “most irrational” of all the irrational numbers [6, 12]. This description arises because ϕ possesses an infinitely recursive continued fraction where none of the integer values are above 1. This is extremely slow to converge in comparison with other irrational approximations as it has the smallest possible sequence of denominators, simply adding 1 to each recursive iteration [2]. As such, the numerator and denominator representing the golden ratio are more incommensurable than other irrationals. In terms of oscillatory activity in the brain, this translates to phase sequences of two neural frequencies that are, in combination, least able to produce an integer value relationship that allows for phase locking [12]. By this account, a ratio at or close to ϕ would entail the two frequencies will rarely synchronize, with the consequence that cognition will either not occur or its function will be impaired. This is consistent with the idea that an irregular patterning in cross-frequency phase meetings conferred by ϕ characterize the resting state of the brain, in which no selective information processing is argued to take place [12].

The idea that cognition and particularly visual cognition related to aesthetic preference may be influenced by ϕ at the level of neural dynamics provided the idea from which the research presented here developed. More specifically, we believed size relations in golden ratio might influence judgments concerning areal relations. This seems plausible given conclusion that geometrical illusions, which illustrate size contrast [22,23] and size assimilation [24], are best described as contour interaction illusions, which, are in turn explicable in terms of the mutual adaptation (likely mediated by the synchronization) of spatial-frequency detectors in the visual system [25–27]. Related to the frequency of synchronization, it is generally held that variations in the amplitude of the EEG response relates to the number of synchronized neurons. In turn, we assumed that amplitude and therefore synchronization frequency also differs between neural assemblies of different size. This assumption was based upon EEG evidence, which has found amplitude to be proportional to the number of synchronously active neural elements [28], with slowly oscillating cell assemblies comprising more neurons than assemblies of higher frequencies [29]. Related to synchronization a simulation study [30] has shown that amplitude increases and frequency decreases with an increasing number of interconnecting neurons and therewith the need to synchronize neurons across different patterns of spike timing or membrane potential fluctuations as well as different patterns of synaptic delay.

In addition, much of the evidence for the way that ratio proportions are represented psychologically deals with their symbolic or numerical notation (i.e. fractions). Experiments usually involve comparison of a pair of fractions; with participants deciding which of two values is larger. Results show a numerical distance effect; that is, the distance between the absolute magnitudes of the individual fractions predicts performance in both accuracy and speed [31,32]. For symbolic comparisons, this suggests that the representations of the integrated fractions are implemented according to an analogical labeled line code (i.e. a holistic processing strategy) rather than being due to cross-comparison of the rational components in an online computation. The integration of fraction components into a holistic representation is shown to exhibit theta and gamma band synchrony over frontal and central-parietal areas in the EEG [33], implying the binding of the individual numerator and denominator representations into a common neural code. A holistic processing strategy is also shown to produce less alpha-band desynchronization, implying that this process does not actively down regulate cortical networks concerned with attention and executive control; i.e. it relies on more intensive neural resources than strategies using online computation [33]. In this case, irrationals may slow or impair responding by making the process of holistic integration more difficult than the analogical code would otherwise account for. In other words, the magnitude estimation of a single ratio relationship might be especially difficult according to the irrationality of the numerator and denominator. Evidence from an fMRI study shows that when a proportion magnitude was presented as an adapting stimulus, followed by a test proportion, BOLD signal adaptation and recovery was similar to that for numerical fractions [34]. In this case, regions surrounding the intraparietal sulcus showed stronger recovery for test proportions that deviated further from the adapting stimulus. On this basis it is argued that proportion coding is closely comparable to symbolic fractional representation as it also subject to the numeric distance effect, is implemented automatically, and independent of presentation format [34].

Golden sectioning may therefore influence the efficiency of visual scene coding, in which context it would seem likely to impair or at least slow processing of holistic structure. Accordingly, the purpose of this study was to investigate the proposal that fast and efficient areal comparisons would be impaired when the relevant areas are in golden-ratio proportions to each other relative to areas in other ratios.

## Materials and Methods

### Participants

Twelve naïve participants (5 male, mean age of 25.27 years +/- 3.41 years) took part in Experiments 1 and 4. Eight participants (4 male, mean age 26.125 +/- 4.2 years) took part in Experiment 2 and 10 participants (6 male, mean age 22.625 +/- 2.5 years) in Experiment 3. Participants had normal or corrected to normal vision. All participants provided written consent to participate in this study. The complete protocol, including provision of written consent, was approved by the institutional Research Ethics Committee at the National University of Ireland Galway.

### Apparatus and Stimuli

**Fig 1:**
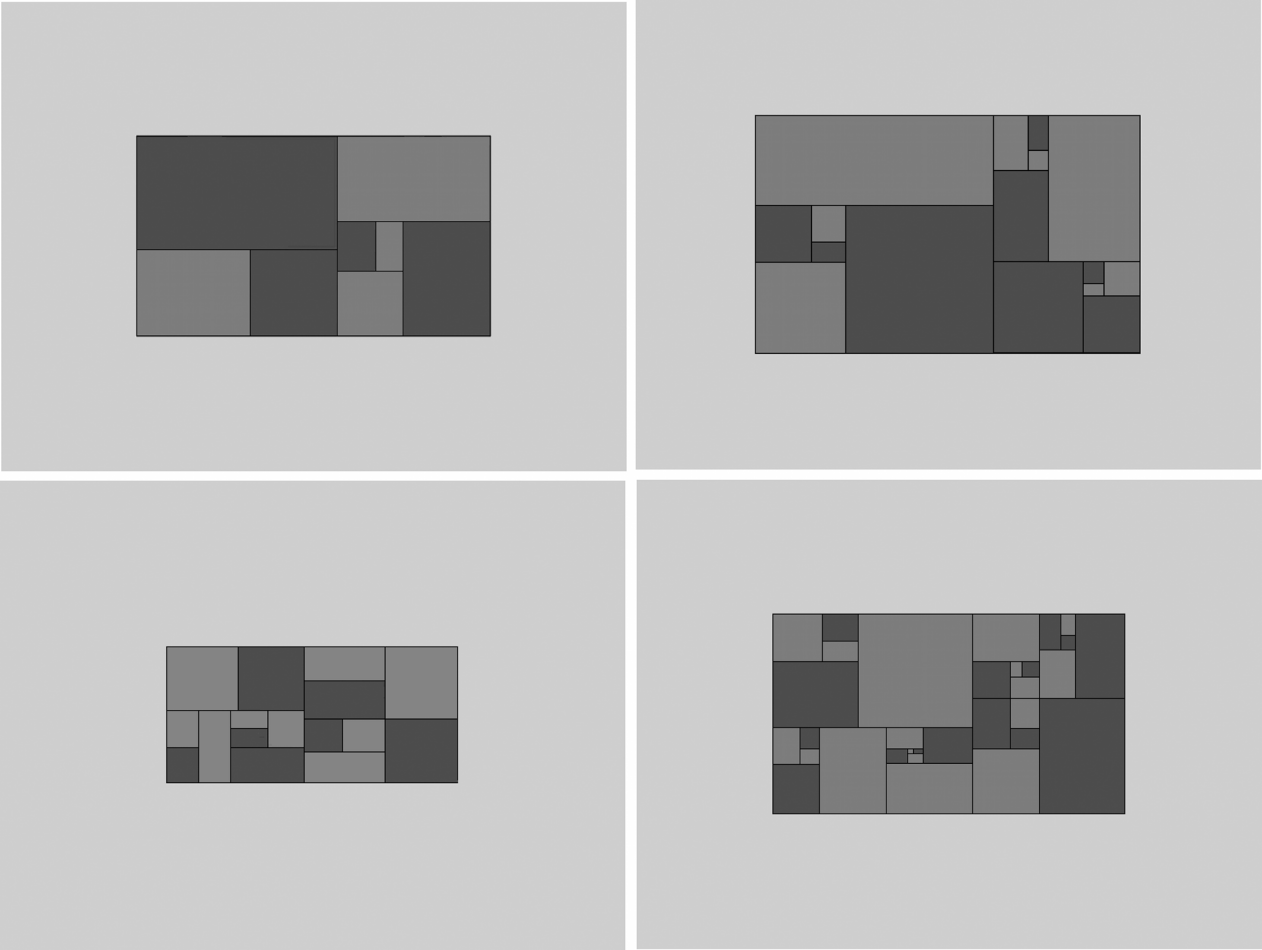
Example grids used in all experiments, from top to bottom, left to right a 4-paired sectioned pattern with sections in area ratio 1|0.568, an 8-paired sectioned pattern with sections in area ratio 1|0.618 (the golden section), an 8-paired sectioned pattern with sections in area ratio 1|0.568 and a 16-paired sectioned pattern with sections in area ratio 1|0.668. The participants’ task was to respond by button press as rapidly and accurately as possible to the luminance of the smallest section. Patterns varied in size with 4, 8 or 16 paired sections and the ratio in area of the sections with ratios of (1|0.468, 1|0.518, 1|0.568, 1|0.618, 1|0.668), where 1|0.618 is the golden section.

Stimuli were generated using E-prime 2.0 PRO (Psychology Software tools Inc.) on a Pentium 4 PC running Windows XP. Stimulus control and presentation were programmed using native E-prime macro scripts with system integrity checked by means of an E-prime refresh clock test which gave a diagnostic classification of ‘good’ (measurement error +/- 1 ms). Stimuli were presented on a 19” Magic Displays monitor, model CPD-4402 Trinitron with resolution 1024x768 and refresh rate 75 Hz. Responses were recorded using a two key Ergodex DX1 Input System. To approximate a relationship between golden sectioning in composition with brain activity we constructed patterns sectioned with various ratios between their larger and smaller sections that included the golden section (Fig 1). We reasoned that areal ratios in golden section would promote neural responses corresponding approximately in terms of the number of contributive neurons to the ratios of larger to smaller areas. This seemed a plausible assumption given there are non-integer as well as harmonic relations between neural oscillation frequencies in the gamma band, which is the band most closely associated with coding sensory structure [35]. If the number of neurons responding to a given stimulus relates to the frequency at which those neurons synchronize, then the frequency-response adopted by neurons across two areas in golden ratio should also be, approximately, in golden ratio. Stimuli were rectangular, horizontally (Experiments 1, 3 and 4) or vertically (Experiment 2) oriented Mondrian-like patterns with 4-, 8- or 16-paired sections (Fig 1). Each pattern contained a set of differently sized, paired sections with self-similar dimensions. The ratios of the paired sections examined were 1:0.468, 1:0.518, 1:0.568, 1:0.618*, 1:0.668 (*0.618 is the golden ratio, or more precisely it’s reciprocal, but in this case the two are interchangeable) [36]. The set of ratio intervals and sizes were chosen because they allowed equal increments that would not cause large discontinuities in the appearance of the stimuli across ratios. They also avoid the possibility of patterns producing overly small targets when sectioned beyond a certain point. The position of the golden ratio as 4^th^ largest within the range of 5 ratios used sought to avoid any biased responding that may arise from a central position in the range of ratios [37,38]. The sectioning procedure generates the stimuli in each case by first drawing the rectangular boundary of the whole pattern irrespective of ratio, the dimensions of the pattern match those of the screen to avoid biasing responses towards ratios that more closely match the screen dimensions (in other words we did not rule out the possibility that self-similarity or the effects of fractal complexity could extend beyond the stimulus). The pattern was overall 0.5 times the area of the screen and centrally placed. The color of the pattern’s interior was randomly assigned to one of either of the potential target colors “light gray” and “dark gray” in VBA/E-Basic designations. The first sectioning was then achieved by multiplying the length along the x-axis to achieve the subdivisions of the relevant ratios. After establishing the first section, the sectioning algorithm then performed the same operation on the smaller area, with the section matching the target ratio being assigned to the smaller of the two areas. The larger sectioned area then underwent the same operation recursively and with the number of sectioning operations determined by set size. The two subsections of each paired section were shaded light and dark gray with equal probability across the overall set of patterns with this procedure the same for all ratios in order to establish a consistent layout, and to make sure that the target appears in approximately the same location. Larger set sizes follow the same procedure as longer series of repetitions. Overall pattern size did vary slightly leading to 4 different grid sizes of between 1 and 2° of visual angle at 55 cm viewing distance. All patterns were presented at the center of the monitor screen. In Experiment 3, randomly distributed pixels accounting for 20% of each pattern employed in Experiment 1 were transformed from light or dark grey to either white or black with the result of pixelating the pattern displays.

### Design and Procedure

Experiments 1, 2 and 3 used a within subjects design and a speeded-response task in which participants were asked to determine the shading (e.g. dark–right key, light–left key) of the overall smallest pattern section and respond by button press as rapidly and accurately as possible. Some previous studies have linked golden-section preference with visual field morphology or to the velocity of visual scan which exhibits a ^3^/_2_ horizontal/vertical ratio [39–41]. Accordingly, in Experiment 2 we examined the hypothesis that if an egocentric, horizontal reference frame defined by the visual field influenced participants’ performance, presentation of vertical instead of horizontally oriented patterns would exclude any influence of the golden section. Experiment 3 added uniform visual noise to the golden-section patterns to convolve with pattern structure and to assess whether or not this influenced the effects of golden sectioning on RTs. Experiment 4 employed a paired comparison task in which participants were asked to rate the relative pleasantness or aesthetic value of one pattern against another.

Each trial started with the presentation of a central fixation cross for 500 milliseconds (ms). The fixation cross was then immediately replaced by the search pattern, to which observers responded. Patterns remained on screen until a response was recorded. In case of an erroneous response or a time-out (i.e., after a period of 2,500 ms without response), feedback was given by a computer-generated tone and an alert was presented for 500 ms at the center of the screen. Each trial was separated from the next by variable intervals of 500–1,000 ms. Following a 20-trial practice session participants completed 600 trials in one 15-block session ensuring 40 trials per experimental condition. Pattern presentation was fully randomized across all conditions, across blocks and randomized separately for each participant. The experiment was carried out in a sound proof booth with low ambient lighting with a chin rest used to ensure distance between participant’s eyes and the monitor was kept constant at 55 cm.

Experiment 4 used a paired comparisons procedure to investigate whether or not there was a significantly higher preference for the golden ratio relative to the other ratios used in Experiment 1. Experiment 4 used the same stimulus patterns with presentation order fully randomized for 40 trials per condition and 600 trials overall. Participants were asked to report which pattern of the two they thought was the most ‘pleasing’. The stimuli, stimulus presentation and experimental conditions were identical to those employed in Experiment 1. Pattern-pair presentation was fully randomized across 25, 40-trial blocks and separately for each participant. The running order of Experiments 1 and 4 were varied such that Experiment 1 was conducted first for 50% of participants.

## Results

### Analysis of Aesthetic Judgments

**Fig 2:**
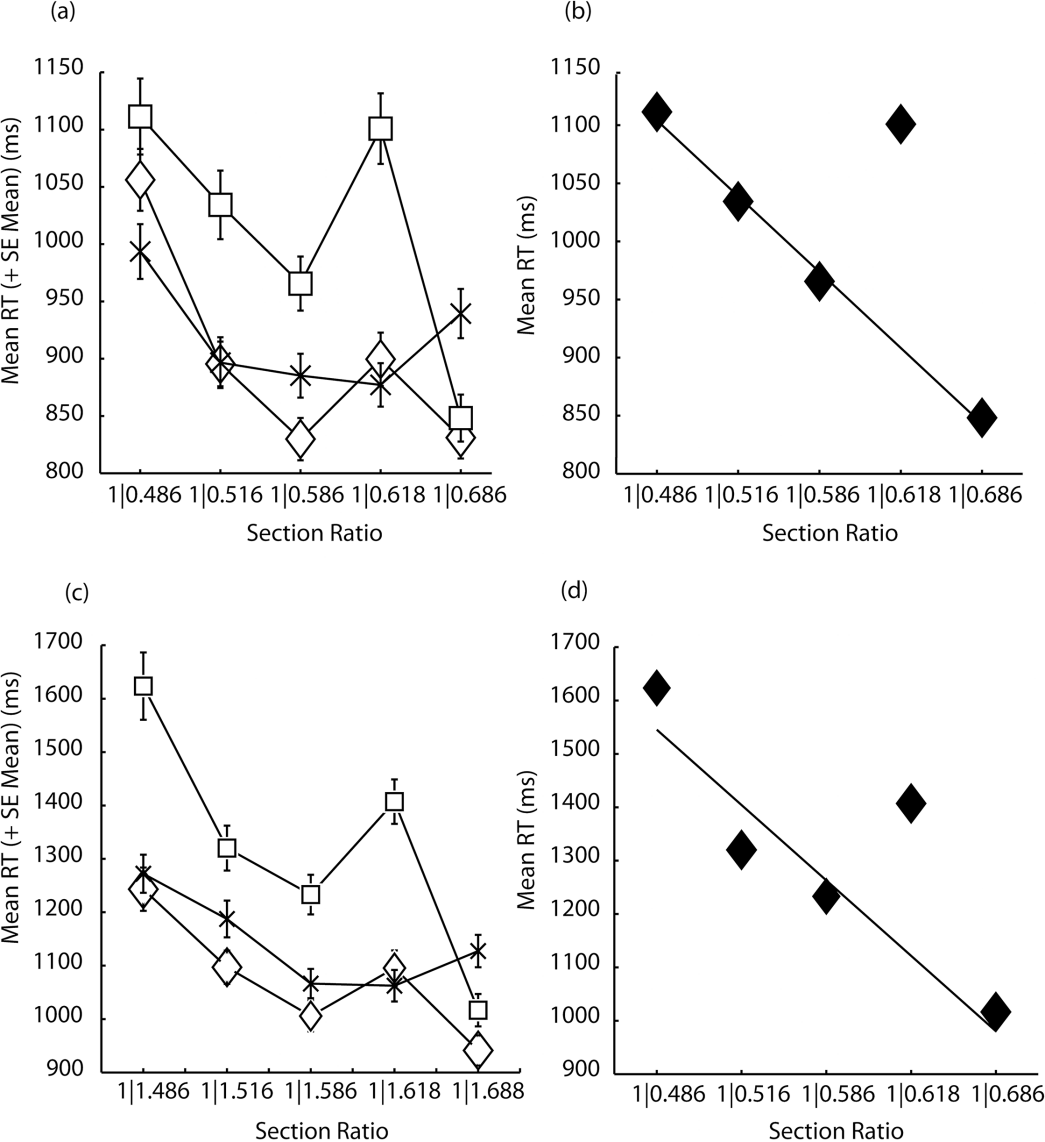
For Experiment 1 (a) shows mean RTs and their standard errors as a function of ratio for the three pattern sizes 4-paired sections (diamonds), 8-paired sections (squares) and 16-pairted sections (stars), respectively. In (b) is shown the regression line of RTs over ratio when the golden section is excluded from analysis alongside diamonds signifying the mean RTs for all ratio conditions. A similar patterning describes RTs for Experiment 2 (b and c).

The multiple pairwise-comparison data obtained in Experiment 4 (**S1 Table**) were analyzed according to the law of comparative judgment [42], but failed to identify any patterning as ranking significantly differently to the median ranking [χ^2^ (4, N = 60) = 0.52, p = .97]. The results of Experiments 1 and 2 are illustrated in Fig 2(A)–2(D). For Experiments 1–3, repeated-measures analyses were carried out using SPSS 21.0 (SPSS, IBM, Inc.), which returns observed or post-hoc power, and partial eta squared (*η* ^2^) statistics. Trials with error responses were removed from the data prior to subsequent analyses. Error RTs tended to be slower overall than correct RTs, and analysis of the probability correct by RT revealed no significant correlation between RT and accuracy. This argues against the correct data being contaminated by accuracy-speed trade-offs. Examination of the correct RTs revealed non-normal distribution with pronounced positive skew. A Kolmogorov ‘D’ test showed RT distributions to be approximately lognormal and on this basis subsequent analyses were conducted on the exponents of the means of log-transformed RT distributions [43,44]. Huynh-Feldt, Greenhouse Geisser or Lower bound epsilon adjustments were applied where sphericity assumptions are not met [45].

### Reaction-Time Analyses (Experiments 1 and 2)

In Experiment 1, examination of the log-transformed correct RTs (85%) (**S1 Dataset**) by means of a repeated-measures ANOVA revealed significant main effects for ratio [F(2.1, 23.8) = 41.24, p < .001, *η* ^2^ = .79, power = 1], and pattern [F(2, 22) = 16.9, p < .001, *η* ^2^ = .61, power = .91] as well as a significant interaction [F(3, 33.46) = 13.25, p < .001, *η* ^2^ = .55, power = 1].

Fig 2(A) shows that RTs did not increase linearly as a function of an increase in the pattern sections as might be expected if participants engaged in serial search for the target section. Fig 2(A) also indicates the interaction to be complex: RTs decreased overall with increasing ratio although this effect was compromised for both 4 and 8-paired sectioned patterns, which are characterized by notably slower RTs to the golden-sectioned patterns. As illustrated in Fig 2(A), the different pattern x ratio RTs were likely to be explained by different models and as a result, subsequent analyses sought to establish the model that best described the decrease in RT with ratio for each pattern separately. RTs decreased nonlinearly with increasing ratio for the 4- and 16-paired-sectioned patterns [logarithmic r^2^ = .752, F(1,3) = 9.072, p = 0.057 and quadratic r^2^ = .964, F(2,2) = 26.4, p < .036, respectively]. The 8-paired sectioned pattern was explained by a near perfect linear function, but only following removal of the golden ratio RTs [r^2^ = .996, F(1,3) = 454.8, p < .001], and was not linear if analysis included these RTs [r^2^ = .448, F(1,3) = 2.43, p = .217, see Fig 2(B)]. Note that the logarithmic fit of RT over ratio also improved slightly for the 4-paired sectioned patterns following removal of the golden ratio RTs, but the fit failed to achieve significance (logarithmic r^2^ = 0.862). Because RTs did not decrease linearly with an increase in the number of sections we might conclude that RT variability relates more to discrimination at the target location than it does to search.

Fig 2(C) shows that the pattern of correct RTs (92%) in Experiment 2 (**S2 Dataset**) very closely matched those in Experiment 1: there were significant main effects for ratio, [F(1.6, 11.4) = 24.06, p < .001, *η* ^2^ = .78, power = .99], and pattern [F(1.11, 7.8) = 25, p = .001, *η* ^2^ = .78, power = .99] while the interaction was also significant [F(1.82, 12.73) = 7.39, p = .008, *η* ^2^ = .51, power = .85]. As with Experiment 1, RTs decreased nonlinearly with increasing ratio for the 4- and 16-paired-sectioned patterns [logarithmic r^2^ = .776, F(1,3) = 10.4, p = 0.048 and quadratic r^2^ = .946, F(2,2) = 17.57, p = .054, respectively]. Again consistent with Experiment 1, examination of RTs to the 8-paired sectioned patterns revealed a linear function only following removal of the golden section RTs [r^2^ = .919, F(1,3) = 22.72, p = .018], and not on the basis of analysis of the full range of RTs over ratio [linear r^2^ = .637, F(1,3) = 5.27, p = .105, see Fig 2(D)]. Similar again to Experiment 1, there was a logarithmic fit of RT over ratio following removal of the golden section RTs in the 4-paired sectioned patterns [r^2^ = .987, F(2,2) = 154.72, p = .006], which was not found for the 16-paired sectioned patterns.

### Response-Error Analyses (Experiments 1 and 2)

Analysis of response error (15% and 7.9% of trials overall in Experiments 1 and 2 **S2 Table** and **S3 Table**) were carried out using the same ANOVA as for the RTs but on the arcsined square-root error proportions. These analyses revealed significant main effects for ratio in both experiments [F(2.1, 23.4) = 39.9, p < .001, *η* ^2^ = .78, power = 1 and [F(4, 28) = 39.5, p < .001, *η* ^2^ = .85, power = 1, for Experiments 1 and 2, respectively], which is consistent with the corresponding RT effects, i.e. improved task performance is associated with faster RTs. In Experiment 1 simple-effects analysis of the ratio x pattern interaction [F(7.2, 78.8) = 24, p < .001, *η* ^2^ = .69, power = 1] showed 8-paired (golden)-sectioned patterns produced significantly higher errors than were produced for the 1:0.518 (p < .001), 1:0.568 (p = .013) and 1:0.668 (p < .001) ratios. However and unlike the RT analyses, this pattern was not replicated for Experiment 2 in which a corresponding simple-effects analysis showed no differences in error production between the 8-paired (golden)-section and other patterns. These patterns in error production tend to suggest that the task becomes overall easier with increasing ratio, and the increasing conspicuity of the smallest sections. However, the pattern of RT performance to the 8-paired section conditions is not consistently matched by variations in task difficulty.

### Fractal Patterning (Experiments 1 and 2)

Because self-similarity characterizes both natural scenes and art, including abstract compositions [46,47] it was decided to examine any effects of the fractal dimensionality of the stimulus grids on the RTs from Experiment 1. Analysis of these RT data suggests that golden sectioning influences processing at the level of the local section and not mechanisms involved in pattern search. However the specificity of this effect to the 8-paired sectioned patterns suggests that pattern composition plays a critical role. All of the patterns were fractal in that smaller sections were reduced copies of larger sections and golden sectioning may be a mathematical fractal when the ratio refers to a formula that is based upon recursive iteration. Accordingly, it seemed sensible to assess whether or not RTs to golden-section patterns were influenced by fractal dimensionality. If RTs were found to correspond to the fractal dimension (d) between golden ratio and other ratios, it might be conjectured that slowed RTs to the golden-sectioned grids came about due to an inability to successfully separate the target from the background as a function of fractal dimensionality.

**Fig 3:**
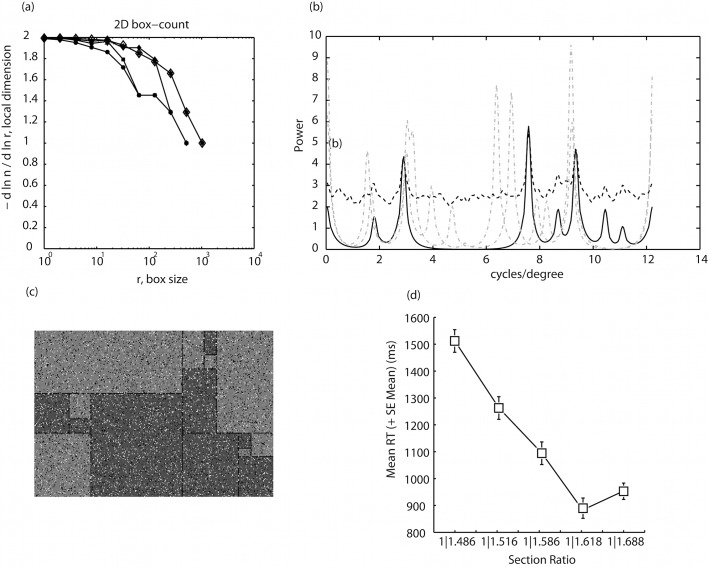
(a) The Minkowski–Bouligand or box-counting dimension for the 5 ratios (1|0.468, 1|0.518 and 1|0.568 circles, squares and diamond, respectively) as well as (1|0.618, and 1|0.668, large open diamond and star). Box counting characterizes a fractal set by determining the number (N) of boxes of size R required to cover the fractal set, following the power law N = N0*R^-df^ with df < = *d* (or fractal dimension). In (b) the spatial frequency structure of the golden-16-sectioned patterns, determined by application of a bank of log-Gabor filters is illustrated by the black continuous line, with golden-4- and 16-paired sectioned patterns presented for comparison purposes as gray discontinuous lines. Unlike any other pattern the 8-paired (golden) sectioned pattern exhibits an absence in spatial frequency information in the middle of the range of spatial frequencies possessed by the pattern, at around 3–7 cycles per degree of visual angle. Addition of uniformly distributed random noise (white or black pixels) to 20% of the 8-paired sectioned patterns convolved with the natural pattern amplitude spectra of the patterns to raise all lower values in the amplitude spectrum, particularly, raising zero values above zero. The black dashed line illustrates the resulting amplitude spectrum. An example pattern is given in (c); (d) shows that following this modification, in Experiment the 3, golden-section RTs were not elevated as had been found in Experiments 1 and 2 but corresponded to the approximately linear negative function describing RT over ratio.

Fig 3(A) illustrates the Minkowski–Bouligand or box-counting dimension of the different ratio conditions. Box counting characterizes a fractal set by determining the number (N) of boxes of size R required to cover the fractal set, following the power law N = N0*R-df with df < = d. Estimating the log-log fit for each distribution of N for different box sizes revealed the following, near identical exponents for each ratio condition (Table 1):

**Table 1:** Exponents describing the fractal dimensionality for each of the ratios employed in Experiments 1, 2 and 3.

Ratio	Exponent
1:0.468	-0.5822
1:0.518	-0.5699
1:0.568	-0.5341
1:0.618	-0.5424
1:0.668	-0.5405

It can be observed from Fig 3(A) that as the number of boxes increases the degrees of freedom or number of dimensions decrease in this case over the range 2–1. Within this range, analysis of all sample points (dimensions) using logistic regression analysis showed mean RTs correlated reasonably well (although non-significantly) with the number of boxes. While by no means conclusive this analysis is suggestive of a relationship between fractal dimensionality and RTs, with the best correlation for d = 1 where McFadden’s pseudo r^2^ = .418, χ^2^(4, N = 5) = 6.73, p = .151. However and related to our question, explanatory power was only slightly reduced following the removal of the golden-section RTs (r^2^ = .406) suggesting that the golden-section RTs are unlikely to deviate substantially from any more general relationship between RTs and fractal dimensionality [48].

### Spatial-Frequency Structure (Experiments 1 and 2)

We believed a second possibility was that the spatial-frequency structure of the patterns themselves related to the slowed RTs. In a first step all patterns were subject to analysis using a bank of log-Gabor filters [49] to better approximate the ^1^/_*f*_ amplitude spectra present in natural images than Gabor filters while also being analogous with measures of human visual-system function, which indicate we have neuronal responses that are symmetric on the log frequency scale. The results of this analysis are given in Fig 3(B) [50] Here it can be seen that unlike any other patterns tested, the 8-paired (golden)-sectioned patterns exhibit an absence of information in the amplitude spectrum across a bandwidth of over 3 cycles-per-degree within the range 4–7 cycles-per-degree. One corollary with this might be in the number of light or dark sections that sit adjacent but are of different polarity to the target section, however RTs were found not to correlate (r^2^ = .04) with the number of adjacent sections of opposing polarity.

### Experiment 3: Reaction-Time and Response-Error Analysis

In Experiment 3 the spatial frequency structure of the 16-sectioned patterns was masked by addition of uniformly distributed random noise (white or black pixels). Twenty-percent of each pattern was masked by noise allowing target sections to remain discriminable while at the same time influencing the amplitude spectra of the patterns: analysis of the resulting patterns using log-Gabor filters had the effect, illustrated in Fig 3(B), of convolving spatial frequency information in the noise with the existing spatial-frequency structure, thus augmenting lower values in the amplitude spectrum and raising all zero values above zero. If the unusual spatial-frequency structure of golden-sectioned patterns were responsible for the RT outliers in Experiments 1 and 2, the addition of uniformly distributed noise in the current experiment should reduce the golden-section RTs, which should then not be found to deviate significantly from the function describing decreasing RT over ratio. In Experiment 3 the ratio main effect was the same as that found in Experiments 1 and 2 [F(1, 9) = 51, p < .001, *η* ^2^ = .85, power = 1]. However and consistent with expectations, RTs decreased linearly with increasing ratio ([r^2^ = .9, F(1,3) = 26.97, p = .014 Fig 3(D) **S3 Dataset**] and did not show the increase in RTs to the golden-section patterns that was found in Experiments 1 and 2.

Analysis of response error (5.5% of trials overall in Experiment 3 **S4 Table**) was carried out using the same ANOVA as for the RTs but on the arcsined square-root error proportions. These analyses revealed significant main effects for ratio [F(1, 9) = 32.8, p < .001, *η* ^2^ = .79, power = 1], which is consistent with the corresponding RT effects, i.e. improved task performance is associated with faster RTs. No other effects were found.

## Discussion

Experiments 1–3 show that golden-sectioned patterns yield less efficient visual target discrimination as a function of unusual spatial-frequency distributions within the patterns. In this context the golden section serves as a limit to the efficient functioning of perceptual processing. Fig 2 panels (a) and (c) show RTs be approximately distributed such that discrimination performance is fastest for 4, then 16 and slower for the 8-paired sectioned patterns. This suggests that it is not target-section search (which, if serial would favor a 4-8-16-section order in RT magnitude) but time taken to process at the target location that is influenced by golden sectioning. This is consistent with the idea that inefficient and/or absent synchronization between neurons participating in spatial frequency analysis requires additional processing resources (for example top-down driven synchronization) and therefore more time to successfully process the pattern. Linkage with previously discussed ideas [12] derives from the assumption that within-pattern segmentation is achieved by virtue of frequency or phase differences between neural responses to the larger and smaller sections. On this assumption, inefficient or absent synchronization between neural responses might result in inefficient, and as a consequence slowed processing because neurons coding the different sections would operate in different temporal codes that would not easily combine to facilitate their processing within a common framework.

Concerning the relationship of the golden section to aesthetic preference, the contribution of Experiment 4 to the mixture of findings for and against, weighs in against the idea of golden sectioning as a major principle underlying aesthetic preference [2]. However, this conclusion may be premature and the relationship between golden sectioning and aesthetic appeal more complex than is often taken into account. To illustrate this: in difficult visual-search displays, it may happen that a participant exercises an “optimal stopping rule”, application of the rule equivalent to responding because “it never usually takes this long to make a response”, and under such circumstances, the rule might explain a peak in errors at slow response latencies. In the present context, and given an understanding that there may a basis for aesthetic appeal in some of the presented patterns, a participant might judge patterns that are more difficult to process as aesthetic simply because they are taking unusually long to view. In spite of the fact that this judgment would likely occur without introspection on the fact that it is made based upon noticeably prolonged viewing, if it were the case, golden-section preference might be considered a demand characteristic in an experimental procedure. However and equally, contemporary art forms similar to the stimuli employed in this study can be considered to possess aesthetic value. In theses cases, prolonged viewing might be sufficient to encourage an aesthetic dialog, proceeding from the observation that “I have been viewing this work for slightly longer than usual”. Of course this does not account for why, in Experiment 4, participants did not prefer the golden-sectioned patterns over other patterns. Perhaps in this case initiation of the aesthetic dialog relies upon prior, relevant knowledge of the potential for the patterns to possess aesthetic appeal. If so, and if participant samples were not deliberately biased towards non-naive participants, measuring aesthetic preference may end up being a matter of chance rather than based upon systematic variation of the experimental conditions.

Two future research agendas emerge from this: agenda (a) recommends using neuroscience methods to investigate the functional dynamics of mechanisms engaging, via synchronized activity, two or more neural assemblies either in golden ratio or in response to a stimulus characterized by the golden ratio; agenda (b) rests on the assumption that, under some circumstances, an aesthetic response could evolve as a consequence of the viewer responding implicitly (and in the same sense as governs operation of an optimal stopping rule in visual search) to viewing a particular pattern for unusually longer than other patterns. In an artistic context this may encourage more explicit introspection and the development of an aesthetic dialog, in this instance with golden-sectioned images.

In conclusion, golden sectioning influences the efficiency of visual processing. This appears to be a function of the absent spatial-frequencies within the 8-paired sectioned patterns but it is not related to the fractal dimensionality of the golden-sectioned patterns. It is interesting to note that 4–7 cycles-per-degree is found within the human contrast sensitivity function, which shows a band-pass filter shape peaking at around 4 cycles-per-degree [51]. This suggests that the optical limit possessed by golden-sectioned patterns lies in the absence of spatial frequencies to which the visual system is maximally sensitive. The resultant slowing in processing may arise due to the need to engage spatial frequency analyzers by means of top-down activation. This leads to the tentative hypothesis that, slowed processing to a particular pattern within a set of similarly structured patterns, may be sensed by the observer and under particular circumstances (e.g. given art-like pattern structure and when the observer is aware of the possibility for aesthetic dialog) may initiate an aesthetic response. While not a general theory of aesthetics, this account might describe why under some circumstances, the golden section appears to be of aesthetic value and on other occasions does not. This conclusion requires further scientific study.

## Supporting Information

S1 Dataset Experiment 1, raw and tabulated RT data. (DOCX)

S2 Dataset Experiment 2, raw and tabulated RT data. (DOCX)

S3 Dataset Experiment 3, raw and tabulated RT data. (DOCX)

S1 Table Tabulated data for Experiment 4. (DOCX)

S2 Table Experiment 1, tabulated error data. (DOCX)

S3 Table Experiment 2, tabulated error data. (DOCX)

S4 Table Experiment 3, tabulated error data. (DOCX)
