# Supplementary material for: The Golden Section as Optical Limitation
Source: PLoS One. 2015 Jul 8;10(7):e0131045. doi: 10.1371/journal.pone.0131045 (PMC4495923; doi:10.1371/journal.pone.0131045)
Supplement: S1 Dataset — (DOCX) [file pone.0131045.s001.docx]

**Dataset S1. Mean Reaction Times Experiment 1**

| **c1** | **c2** | **c3** | **c4** | **c5** | **c6** | **c7** | **c8** | **c9** | **c10** | **c11** | **c12** | **c13** | **c14** | **c15** | **S** |
| --- | --- | --- | --- | --- | --- | --- | --- | --- | --- | --- | --- | --- | --- | --- | --- |
| 1143.79 | 974.07 | 867.73 | 951.23 | 882.09 | 1000.96 | 1004.88 | 1081.64 | 1053.15 | 932.37 | 1101.05 | 946.88 | 974.80 | 922.15 | 974.40 | 2 |
| 1035.59 | 906.67 | 893.08 | 1049.26 | 890.90 | 1297.11 | 1226.11 | 1055.30 | 1278.26 | 917.95 | 984.94 | 942.80 | 883.79 | 859.15 | 913.10 | 3 |
| 1046.47 | 869.34 | 764.01 | 825.09 | 784.00 | 1220.67 | 1029.45 | 895.80 | 1066.72 | 743.95 | 918.24 | 852.75 | 803.39 | 812.02 | 906.12 | 4 |
| 898.05 | 766.44 | 729.16 | 754.21 | 690.09 | 969.26 | 936.42 | 817.80 | 990.29 | 748.43 | 861.47 | 813.36 | 787.33 | 776.72 | 866.58 | 5 |
| 1153.55 | 968.42 | 875.78 | 1179.98 | 923.79 | 1400.72 | 1367.55 | 1125.78 | 1241.76 | 928.77 | 1079.68 | 995.32 | 973.92 | 910.09 | 950.87 | 6 |
| 991.98 | 936.20 | 848.30 | 851.52 | 832.98 | 1192.11 | 1148.09 | 957.34 | 1141.29 | 863.45 | 1159.33 | 979.69 | 892.75 | 865.17 | 928.19 | 7 |
| 1037.89 | 886.12 | 828.72 | 830.55 | 804.70 | 859.21 | 850.13 | 876.56 | 974.70 | 783.41 | 863.81 | 808.32 | 837.70 | 856.10 | 861.96 | 8 |
| 1399.48 | 1138.31 | 1020.4 | 1130.78 | 967.62 | 1521.39 | 1314.44 | 1224.14 | 1571.71 | 1054.72 | 1226.29 | 1111.85 | 1093.72 | 1169.55 | 1311.05 | 9 |
| 900.29 | 819.02 | 772.44 | 789.52 | 765.48 | 942.32 | 907.93 | 880.16 | 845.18 | 780.81 | 910.33 | 799.28 | 792.64 | 809.77 | 816.64 | 10 |
| 952.88 | 829.74 | 789.49 | 801.18 | 820.08 | 929.90 | 946.20 | 873.24 | 991.23 | 810.23 | 872.83 | 793.27 | 827.91 | 807.22 | 846.84 | 11 |
| 1182.89 | 942.62 | 839.85 | 926.14 | 870.85 | 1227.10 | 1047.67 | 1009.28 | 1258.76 | 909.40 | 1129.89 | 1023.09 | 1025.22 | 993.67 | 1147.94 | 12 |
| 931.09 | 708.10 | 729.05 | 704.88 | 740.83 | 776.56 | 633.37 | 790.07 | 797.35 | 704.46 | 815.27 | 692.50 | 729.47 | 744.17 | 749.30 | 13 |

Key Row 1:

C1 4-paired sections 1:1.468 ratio

C2 4-paired sections 1:1.518 ratio

C3 4 paired sections 1:1.568 ratio

C4 4-paired sections 1:1.618 ratio

C5 4-paired sections 1:1.668 ratio

C6 8-paired sections 1:1.468 ratio

C7 8-paired sections 1:1.518 ratio

C8 8 paired sections 1:1.568 ratio

C9 8-paired sections 1:1.618 ratio

C10 8-paired sections 1:1.668 ratio

C11 16-paired sections 1:1.468 ratio

C12 16-paired sections 1:1.518 ratio

C13 16 paired sections 1:1.568 ratio

C14 16-paired sections 1:1.618 ratio

C15 16-paired sections 1:1.668 ratio

S = Participant number

2 1 16 1947 1 3

2 2 16 1224 1 1

2 3 32 1618 1 2

2 4 8 843 1 3

2 5 16 936 1 5

2 6 16 1500 1 2

2 7 16 911 0 1

2 8 32 1162 1 2

2 9 32 749 1 2

2 10 32 966 1 1

2 11 32 904 1 2

2 12 16 1204 1 1

2 13 8 1061 1 2

2 14 32 748 1 4

2 15 16 1092 1 2

2 16 8 835 1 4

2 17 16 1072 1 1

2 18 16 1416 0 3

2 19 32 941 0 5

2 20 16 1457 0 1

2 21 8 938 1 2

2 22 8 1175 1 1

2 23 32 1337 1 1

2 24 16 1149 0 4

2 25 8 895 1 3

2 26 16 1768 1 3

2 27 8 1127 1 5

2 28 32 751 0 5

2 29 8 1102 1 3

2 30 32 876 0 3

2 31 16 939 0 2

2 32 8 900 1 5

2 33 8 972 1 5

2 34 8 1079 1 4

2 35 16 796 1 3

2 36 16 1114 1 3

2 37 32 1028 1 3

2 38 16 900 0 2

2 39 8 785 0 3

2 40 8 945 0 1

2 41 32 949 1 3

2 42 8 1034 1 3

2 43 8 685 1 3

2 44 8 888 0 2

2 45 16 685 1 4

2 46 32 2162 1 4

2 47 8 906 0 1

2 48 8 639 1 2

2 49 8 813 1 4

2 50 16 724 0 4

2 51 32 951 1 2

2 52 8 811 1 2

2 53 16 1096 1 2

2 54 16 710 0 2

2 55 32 685 1 3

2 56 8 1184 1 4

2 57 32 1041 1 1

2 58 32 723 1 4

2 59 16 1442 1 4

2 60 8 789 1 3

2 61 8 912 1 5

2 62 8 932 1 5

2 63 16 1305 1 4

2 64 16 680 1 3

2 65 16 1048 0 1

2 66 8 899 1 4

2 67 8 840 1 2

2 68 16 1250 1 3

2 69 16 964 1 2

2 70 32 992 1 5

2 71 16 885 1 5

2 72 16 1547 1 1

2 73 8 797 1 3

2 74 32 1835 1 1

2 75 16 894 1 4

2 76 8 936 1 5

2 77 32 874 1 1

2 78 8 767 0 1

2 79 8 925 0 4

2 80 32 1686 1 3

2 81 16 601 0 5

2 82 16 1132 0 5

2 83 16 938 1 4

2 84 16 637 1 5

2 85 32 830 1 1

2 86 32 702 1 2

2 87 16 721 1 1

2 88 32 860 1 4

2 89 16 892 1 5

2 90 32 1240 1 1

2 91 16 784 1 2

2 92 32 831 0 5

2 93 8 936 1 1

2 94 32 658 1 5

2 95 8 837 1 4

2 96 8 768 1 5

2 97 32 1047 1 4

2 98 32 699 1 3

2 99 32 979 0 4

2 100 16 950 0 5

2 101 8 1042 1 1

2 102 32 1184 1 5

2 103 32 1063 1 5

2 104 32 1081 1 4

2 105 16 464 0 5

2 106 8 744 0 2

2 107 32 799 0 3

2 108 8 1102 1 1

2 109 8 1335 1 2

2 110 16 1631 0 4

2 111 32 856 1 2

2 112 8 1150 1 1

2 113 32 1558 1 1

2 114 32 747 0 5

2 115 8 1001 0 5

2 116 32 862 1 4

2 117 16 1361 0 3

2 118 8 916 0 4

2 119 32 1165 1 3

2 120 32 1468 1 2

2 121 32 1006 1 5

2 122 16 997 1 1

2 123 16 851 1 5

2 124 32 562 1 1

2 125 8 740 1 2

2 126 32 867 1 2

2 127 32 846 1 3

2 128 8 911 0 4

2 129 32 1387 1 2

2 130 16 866 1 3

2 131 16 769 0 4

2 132 32 1580 1 2

2 133 8 869 1 1

2 134 32 830 1 3

2 135 16 826 1 5

2 136 32 540 0 4

2 137 32 611 1 1

2 138 8 962 1 2

2 139 8 825 0 2

2 140 8 1858 0 2

2 141 16 1344 1 3

2 142 16 840 1 1

2 143 8 878 1 3

2 144 32 784 1 2

2 145 32 1067 1 3

2 146 16 1097 0 3

2 147 8 931 1 4

2 148 8 832 1 4

2 149 8 1071 1 1

2 150 32 1083 1 1

2 151 16 1118 0 1

2 152 32 956 1 4

2 153 8 1615 1 3

2 154 8 1031 1 3

2 155 16 844 1 1

2 156 32 979 0 5

2 157 16 1097 1 2

2 158 32 1736 1 3

2 159 8 775 1 3

2 160 16 1281 0 3

2 161 8 1372 1 5

2 162 8 1010 0 5

2 163 8 1045 0 4

2 164 32 826 1 5

2 165 32 927 0 3

2 166 16 1004 1 2

2 167 8 616 0 3

2 168 8 1143 1 3

2 169 8 871 1 5

2 170 16 1549 1 4

2 171 16 1020 0 5

2 172 8 1640 1 3

2 173 32 1113 0 1

2 174 16 2302 1 5

2 175 8 1346 1 1

2 176 16 772 1 5

2 177 8 1327 1 2

2 178 16 1037 1 1

2 179 8 1041 1 5

2 180 32 971 1 3

2 181 8 1253 0 2

2 182 16 885 0 3

2 183 8 1259 1 1

2 184 32 930 1 4

2 185 16 1254 0 4

2 186 16 870 1 3

2 187 8 1083 1 4

2 188 8 1043 1 1

2 189 32 838 1 2

2 190 32 1534 1 1

2 191 32 1790 1 4

2 192 32 766 1 4

2 193 8 1355 1 5

2 194 16 925 1 5

2 195 8 1110 1 2

2 196 32 1305 0 1

2 197 8 1124 1 4

2 198 8 1373 1 1

2 199 8 1638 1 1

2 200 8 1187 1 4

2 201 32 1320 1 2

2 202 16 708 1 2

2 203 16 669 1 4

2 204 16 820 1 4

2 205 16 592 1 5

2 206 8 605 1 5

2 207 8 275 1 4

2 208 16 851 1 1

2 209 16 1365 1 4

2 210 32 977 1 4

2 211 32 1101 1 5

2 212 8 764 1 3

2 213 16 861 0 2

2 214 16 849 0 1

2 215 16 1407 0 3

2 216 16 1374 1 1

2 217 16 786 0 4

2 218 8 514 0 5

2 219 32 1247 1 2

2 220 32 1177 1 5

2 221 16 841 1 5

2 222 32 1100 1 4

2 223 8 1012 1 1

2 224 32 670 1 4

2 225 16 801 0 2

2 226 16 541 0 2

2 227 16 1147 0 2

2 228 8 1789 1 5

2 229 32 1329 1 2

2 230 32 1045 1 1

2 231 16 904 1 4

2 232 32 1232 1 3

2 233 8 1103 1 2

2 234 32 1495 1 3

2 235 32 1159 1 5

2 236 16 975 1 3

2 237 32 1244 1 1

2 238 32 946 1 5

2 239 32 601 0 5

2 240 16 1166 0 2

2 241 32 953 1 3

2 242 16 756 1 3

2 243 32 816 1 2

2 244 32 1198 1 1

2 245 16 1176 0 2

2 246 16 1105 1 2

2 247 8 1041 0 2

2 248 8 1163 1 4

2 249 16 1009 0 4

2 250 32 812 1 5

2 251 32 858 1 5

2 252 8 1225 1 5

2 253 16 1391 1 1

2 254 8 961 1 3

2 255 8 768 1 5

2 256 16 1713 0 2

2 257 16 752 1 5

2 258 32 623 1 2

2 259 32 646 0 1

2 260 8 912 1 3

2 261 8 716 1 3

2 262 32 1296 1 3

2 263 16 1037 1 3

2 264 16 642 0 5

2 265 32 860 1 2

2 266 32 1752 1 5

2 267 32 653 1 4

2 268 32 919 1 2

2 269 32 588 1 3

2 270 32 738 1 3

2 271 8 1225 1 2

2 272 32 810 0 5

2 273 8 979 1 2

2 274 32 940 1 5

2 275 8 814 1 5

2 276 8 822 1 1

2 277 8 640 1 4

2 278 8 838 1 1

2 279 16 933 1 2

2 280 16 828 1 1

2 281 16 1208 0 4

2 282 32 429 1 2

2 283 8 513 1 4

2 284 16 447 0 4

2 285 32 458 0 3

2 286 8 1268 0 1

2 287 16 1130 0 3

2 288 16 1581 0 2

2 289 32 968 1 4

2 290 8 1387 1 2

2 291 16 744 1 2

2 292 32 1319 0 2

2 293 32 718 1 4

2 294 8 627 1 2

2 295 8 944 1 5

2 296 16 1455 0 1

2 297 16 1003 1 5

2 298 16 832 1 1

2 299 16 996 1 2

2 300 8 838 1 4

2 301 32 786 1 5

2 302 32 800 1 3

2 303 32 881 1 1

2 304 16 1355 1 4

2 305 8 1052 1 5

2 306 32 1035 1 1

2 307 32 798 1 5

2 308 16 1400 1 5

2 309 32 696 1 4

2 310 8 952 0 4

2 311 8 926 1 1

2 312 8 647 1 3

2 313 8 735 1 4

2 314 32 808 1 4

2 315 8 848 0 5

2 316 32 826 1 2

2 317 8 653 0 1

2 318 16 992 1 5

2 319 32 1138 1 2

2 320 8 766 1 2

2 321 16 712 1 3

2 322 32 780 0 1

2 323 16 322 0 2

2 324 8 1306 1 1

2 325 16 805 1 3

2 326 16 486 0 3

2 327 16 1316 1 4

2 328 8 554 0 3

2 329 16 707 0 5

2 330 32 1410 1 4

2 331 16 1708 1 1

2 332 32 1096 1 1

2 333 16 1102 0 4

2 334 16 1368 1 3

2 335 8 1038 1 2

2 336 32 971 0 5

2 337 8 949 1 3

2 338 32 729 1 4

2 339 8 1587 1 2

2 340 8 807 1 1

2 341 8 742 1 4

2 342 8 817 1 3

2 343 8 739 1 5

2 344 16 940 1 1

2 345 8 695 1 4

2 346 8 705 1 1

2 347 32 1399 1 1

2 348 16 1176 1 5

2 349 8 653 1 3

2 350 8 689 1 5

2 351 16 695 0 5

2 352 32 675 0 4

2 353 16 1169 1 3

2 354 32 1006 1 3

2 355 16 1060 0 1

2 356 16 1294 1 1

2 357 16 761 1 4

2 358 16 1857 0 4

2 359 32 1195 1 1

2 360 32 672 1 3

2 361 8 595 0 4

2 362 32 817 0 4

2 363 32 917 0 3

2 364 8 1431 0 4

2 365 32 909 1 4

2 366 8 1253 1 1

2 367 8 806 1 2

2 368 8 687 1 4

2 369 16 35 1 4

2 370 32 642 1 5

2 371 8 1892 1 1

2 372 8 707 1 3

2 373 8 1347 1 4

2 374 32 704 0 1

2 375 32 694 1 1

2 376 8 962 1 1

2 377 8 1002 1 5

2 378 8 919 1 5

2 379 32 653 1 4

2 380 8 913 1 5

2 381 32 1261 1 5

2 382 32 831 1 3

2 383 8 826 1 1

2 384 8 1353 0 2

2 385 16 590 1 2

2 386 8 671 1 3

2 387 32 712 0 1

2 388 32 965 0 5

2 389 32 963 1 3

2 390 16 824 1 3

2 391 32 1173 1 5

2 392 16 869 1 2

2 393 16 1156 0 2

2 394 16 1141 1 3

2 395 16 1018 1 3

2 396 8 1767 0 1

2 397 32 932 1 2

2 398 32 1348 1 2

2 399 32 770 0 4

2 400 8 1032 1 4

2 401 8 870 0 4

2 402 8 770 1 3

2 403 8 744 1 5

2 404 16 1186 0 2

2 405 32 759 1 3

2 406 16 1219 1 1

2 407 16 660 1 5

2 408 8 579 1 3

2 409 8 569 1 5

2 410 8 1004 1 2

2 411 8 761 1 3

2 412 32 1642 0 2

2 413 8 1597 1 2

2 414 16 1467 1 1

2 415 32 1275 1 5

2 416 8 781 1 3

2 417 8 785 1 5

2 418 16 891 0 5

2 419 16 1012 0 5

2 420 32 994 1 2

2 421 16 1164 1 5

2 422 32 810 0 5

2 423 16 706 1 5

2 424 8 687 1 4

2 425 32 793 1 3

2 426 16 761 0 2

2 427 16 1377 0 1

2 428 8 620 0 1

2 429 16 1949 0 1

2 430 16 1673 1 4

2 431 16 813 0 3

2 432 16 1061 1 5

2 433 16 1013 1 5

2 434 16 1280 0 4

2 435 32 1232 1 5

2 436 32 1057 1 3

2 437 16 1373 1 3

2 438 16 1083 0 1

2 439 32 1406 1 2

2 440 8 819 0 1

2 441 16 1140 0 2

2 442 8 972 1 2

2 443 32 936 1 3

2 444 16 742 1 4

2 445 16 756 1 2

2 446 32 536 1 2

2 447 32 574 1 4

2 448 32 570 1 5

2 449 32 843 1 4

2 450 8 847 0 4

2 451 32 891 1 2

2 452 32 1140 1 1

2 453 32 761 1 4

2 454 16 937 0 1

2 455 16 1647 0 4

2 456 32 1108 1 1

2 457 32 929 1 2

2 458 16 827 1 4

2 459 8 1355 0 1

2 460 16 679 0 2

2 461 16 1716 1 4

2 462 16 760 1 3

2 463 32 1183 1 1

2 464 32 986 1 1

2 465 16 781 1 3

2 466 32 692 0 3

2 467 32 962 1 4

2 468 16 1034 1 1

2 469 16 740 1 4

2 470 8 679 0 5

2 471 8 863 0 5

2 472 16 1638 1 3

2 473 8 904 1 3

2 474 16 789 1 1

2 475 16 922 1 5

2 476 32 1328 1 1

2 477 8 777 1 2

2 478 8 1270 0 2

2 479 8 1245 1 3

2 480 8 1103 1 2

2 481 8 1967 0 2

2 482 16 801 1 4

2 483 16 2049 0 2

2 484 8 1082 1 2

2 485 8 801 1 5

2 486 32 1104 1 4

2 487 8 957 1 3

2 488 8 1005 1 5

2 489 16 806 1 1

2 490 32 956 1 1

2 491 32 890 1 2

2 492 8 761 0 1

2 493 8 844 1 4

2 494 16 540 1 5

2 495 32 1283 1 4

2 496 8 867 1 5

2 497 8 1977 1 4

2 498 32 1064 1 5

2 499 32 771 0 5

2 500 32 694 1 5

2 501 16 886 1 5

2 502 16 838 0 2

2 503 16 966 1 5

2 504 16 725 1 4

2 505 32 892 1 3

2 506 16 979 0 4

2 507 16 910 1 5

2 508 8 732 1 5

2 509 16 973 1 2

2 510 32 830 1 3

2 511 32 779 1 5

2 512 32 743 1 4

2 513 8 1038 1 3

2 514 8 964 1 2

2 515 8 890 1 1

2 516 8 563 1 3

2 517 16 691 0 2

2 518 32 645 1 5

2 519 16 749 0 1

2 520 16 1017 1 5

2 521 32 728 0 4

2 522 16 811 1 1

2 523 8 987 0 2

2 524 8 1480 1 4

2 525 32 1021 1 4

2 526 8 990 0 5

2 527 8 814 1 3

2 528 32 1174 1 3

2 529 32 1238 1 1

2 530 16 890 0 1

2 531 32 938 0 3

2 532 16 854 0 1

2 533 16 795 1 2

2 534 16 1071 1 1

2 535 16 1464 1 3

2 536 32 1048 1 3

2 537 16 952 1 3

2 538 16 861 0 3

2 539 16 1773 1 3

2 540 8 921 1 4

2 541 16 1922 1 3

2 542 16 1406 0 4

2 543 32 926 1 5

2 544 8 714 1 5

2 545 8 1846 1 1

2 546 16 813 1 5

2 547 32 1060 1 5

2 548 32 1220 1 2

2 549 32 1243 0 1

2 550 32 1091 1 2

2 551 8 2513 0 3

2 552 16 1380 0 3

2 553 8 1247 1 1

2 554 16 1777 1 2

2 555 8 1501 0 4

2 556 8 1279 0 1

2 557 8 631 1 1

2 558 8 796 1 2

2 559 8 1102 1 1

2 560 32 1134 0 2

2 561 32 985 1 5

2 562 16 660 1 4

2 563 8 716 1 3

2 564 16 1621 0 1

2 565 16 1080 1 3

2 566 8 806 1 4

2 567 16 559 0 3

2 568 8 666 1 3

2 569 32 682 0 3

2 570 32 811 0 2

2 571 32 837 1 1

2 572 16 641 1 5

2 573 8 1913 1 1

2 574 32 758 1 3

2 575 32 977 1 2

2 576 16 944 0 2

2 577 8 2172 1 4

2 578 16 1361 0 2

2 579 16 1921 0 4

2 580 32 1098 1 4

2 581 8 956 1 5

2 582 8 968 1 4

2 583 16 1420 0 4

2 584 32 665 0 3

2 585 32 1165 1 1

2 586 32 1042 0 1

2 587 32 907 1 2

2 588 32 656 1 4

2 589 8 1049 0 2

2 590 16 1990 0 4

2 591 16 1956 1 5

2 592 8 1400 1 3

2 593 16 1435 1 1

2 594 32 1399 1 1

2 595 32 778 1 4

2 596 32 724 1 2

2 597 8 550 1 5

2 598 32 1256 1 1

2 599 8 2155 1 2

2 600 8 967 1 2

2 601 16 2012 1 4

2 602 16 666 1 5

2 603 8 712 1 2

2 604 8 573 1 4

2 605 16 775 1 1

2 606 8 491 1 2

2 607 16 697 1 5

2 608 16 924 0 1

2 609 16 875 1 2

2 610 8 1032 0 1

2 611 32 1134 1 2

2 612 16 966 1 2

2 613 32 567 1 4

2 614 32 758 1 4

2 615 16 802 1 3

2 616 32 730 1 2

2 617 8 1118 1 1

2 618 16 891 1 1

2 619 8 1574 1 5

2 620 8 623 1 5

2 621 32 1606 1 1

2 622 8 1259 1 1

2 623 8 923 0 2

2 624 32 1785 1 2

2 625 16 675 0 2

2 626 32 858 1 5

2 627 8 1201 1 4

2 628 8 673 1 2

2 629 8 735 1 3

2 630 16 1396 1 1

2 631 32 626 1 5

2 632 8 1471 1 2

2 633 16 700 1 5

2 634 8 846 0 3

2 635 8 1355 1 3

2 636 16 1031 0 4

2 637 16 718 0 2

2 638 16 1728 0 2

2 639 32 776 1 4

2 640 32 1606 1 1

2 641 32 903 1 4

2 642 16 800 1 2

2 643 16 998 1 2

2 644 8 669 1 4

2 645 16 474 0 3

2 646 16 913 0 4

2 647 8 1110 1 5

2 648 8 900 1 3

2 649 32 1416 1 3

2 650 32 1367 1 3

2 651 32 592 1 2

2 652 32 765 1 3

2 653 8 955 1 5

2 654 8 1276 1 4

2 655 16 1318 1 3

2 656 8 849 0 5

2 657 16 651 1 5

2 658 16 707 0 1

2 659 32 890 0 1

2 660 16 1289 1 5

2 661 8 1092 1 4

2 662 8 987 1 5

2 663 32 1070 1 2

2 664 32 1050 1 4

2 665 16 1199 1 3

2 666 16 1098 1 1

2 667 8 636 1 1

2 668 32 1406 1 1

2 669 32 1084 1 1

2 670 8 794 1 2

2 671 32 926 1 4

2 672 16 1028 1 4

2 673 8 649 1 5

2 674 16 1038 1 5

2 675 8 877 0 3

2 676 8 1066 1 1

2 677 32 1104 1 3

2 678 8 877 1 1

2 679 16 1460 1 4

2 680 16 1458 1 2

2 681 8 676 1 3

2 682 32 1094 1 5

2 683 32 848 0 2

2 684 8 879 1 4

2 685 16 679 1 3

2 686 16 917 1 5

2 687 8 961 1 1

2 688 32 602 1 1

2 689 8 522 1 3

2 690 16 675 0 3

2 691 32 1159 0 4

2 692 16 1093 1 4

2 693 16 1489 1 3

2 694 16 1388 1 5

2 695 32 1055 1 3

2 696 32 1166 1 5

2 697 32 1410 1 4

2 698 32 1104 1 5

2 699 32 753 1 3

2 700 8 853 1 2

2 701 32 872 1 5

2 702 32 1465 1 1

2 703 16 1129 0 4

2 704 32 995 1 2

2 705 8 1060 1 4

2 706 8 1396 0 1

2 707 16 692 0 1

2 708 32 933 1 3

2 709 32 656 1 2

2 710 16 946 1 4

2 711 32 1170 0 1

2 712 8 1259 0 4

2 713 32 982 0 3

2 714 8 1192 1 3

2 715 8 680 1 2

2 716 16 806 1 1

2 717 32 982 1 5

2 718 16 1094 1 3

2 719 8 712 1 5

2 720 32 1083 1 5

2 721 8 979 1 4

2 722 8 1010 0 5

2 723 8 1201 0 1

2 724 8 1138 1 1

2 725 32 1036 1 3

2 726 32 744 1 5

2 727 16 1026 0 3

2 728 8 1596 1 4

2 729 32 1381 1 1

2 730 32 913 1 3

2 731 32 1378 0 1

2 732 16 1092 1 3

2 733 32 868 0 5

2 734 16 1272 1 4

2 735 16 1553 1 4

2 736 8 1666 1 1

2 737 16 1015 0 4

2 738 32 923 0 1

2 739 16 4354 1 3

2 740 8 887 0 3

2 741 16 961 1 1

2 742 8 951 1 3

2 743 8 758 1 5

2 744 16 1040 1 5

2 745 16 1596 1 2

2 746 16 888 1 4

2 747 32 1285 1 2

2 748 16 1067 1 3

2 749 32 770 1 2

2 750 8 1875 1 1

2 751 16 1389 0 2

2 752 16 906 1 1

2 753 32 1196 1 4

2 754 16 642 0 2

2 755 16 979 0 1

2 756 16 1141 1 5

2 757 32 982 0 2

2 758 32 979 1 3

2 759 16 916 1 1

2 760 8 1031 0 1

2 761 16 1219 1 3

2 762 32 1020 1 4

2 763 16 702 1 5

2 764 8 808 0 2

2 765 16 753 1 1

2 766 8 703 1 2

2 767 16 1438 0 1

2 768 32 1362 1 1

2 769 8 666 1 3

2 770 8 1275 1 1

2 771 16 1047 0 4

2 772 32 719 1 2

2 773 8 819 1 3

2 774 16 741 1 5

2 775 16 551 1 3

2 776 16 837 1 4

2 777 32 850 1 3

2 778 32 704 1 3

2 779 32 1503 1 1

2 780 32 881 1 5

2 781 16 832 1 1

2 782 8 725 1 5

2 783 8 820 1 2

2 784 8 566 1 5

2 785 32 924 1 4

2 786 8 1274 1 3

2 787 32 878 1 4

2 788 16 1185 1 3

2 789 8 485 0 2

2 790 8 751 1 3

2 791 16 859 0 2

2 792 32 1069 1 4

2 793 8 660 1 5

2 794 32 925 1 5

2 795 32 892 1 2

2 796 32 898 1 2

2 797 32 677 1 5

2 798 32 906 0 4

2 799 8 1386 1 4

2 800 8 800 1 4

2 801 8 1027 1 2

2 802 32 643 0 4

2 803 16 1179 1 4

2 804 8 748 1 4

2 805 32 865 1 1

2 806 8 1686 1 1

2 807 32 1042 0 1

2 808 32 2166 1 2

2 809 32 947 0 1

2 810 16 1533 1 1

2 811 32 1014 1 3

2 812 16 1748 1 3

2 813 8 1201 1 3

2 814 32 839 1 5

2 815 32 1085 1 3

2 816 8 1225 1 2

2 817 8 666 1 4

2 818 32 830 0 2

2 819 8 853 0 2

2 820 16 1502 1 4

2 821 8 1221 1 5

2 822 8 1141 1 5

2 823 32 1801 1 3

2 824 16 841 1 5

2 825 16 737 0 5

2 826 8 748 0 4

2 827 16 930 1 2

2 828 8 738 1 2

2 829 16 1266 1 5

2 830 8 895 1 4

2 831 8 786 1 5

2 832 32 869 0 5

2 833 32 859 0 5

2 834 8 1015 1 1

2 835 16 708 1 5

2 836 16 1468 0 2

2 837 16 844 0 2

2 838 8 959 1 3

2 839 16 1631 1 2

2 840 32 965 0 4

2 841 32 1566 1 5

2 842 16 949 1 1

2 843 32 1119 1 1

2 844 16 925 1 5

2 845 32 771 1 5

2 846 16 777 0 3

2 847 8 760 1 5

2 848 8 784 1 3

2 849 32 1085 1 1

2 850 32 622 1 3

2 851 32 943 1 3

2 852 8 1009 1 5

2 853 8 1173 1 5

2 854 16 1157 1 3

2 855 8 772 1 5

2 856 8 1351 1 4

2 857 8 644 1 5

2 858 8 1041 1 3

2 859 16 896 0 5

2 860 16 1392 1 2

2 861 16 914 1 2

2 862 32 959 1 4

2 863 32 700 1 2

2 864 8 860 0 1

2 865 16 1820 0 4

2 866 32 1142 0 3

2 867 32 851 1 2

2 868 8 755 1 1

2 869 16 1379 1 4

2 870 16 821 1 2

2 871 32 1079 1 4

2 872 32 1308 0 1

2 873 16 2449 1 4

2 874 8 785 1 5

2 875 32 960 1 2

2 876 32 895 0 3

2 877 32 556 0 4

2 878 32 1817 1 1

2 879 16 643 0 2

2 880 8 1402 1 5

2 881 16 970 0 2

2 882 16 1326 0 2

2 883 8 1694 1 4

2 884 32 1365 1 3

2 885 16 651 1 1

2 886 32 703 1 4

2 887 32 746 1 2

2 888 16 1245 1 1

2 889 8 778 1 4

2 890 32 876 1 1

2 891 32 915 1 1

2 892 16 900 1 3

2 893 16 994 0 4

2 894 8 718 0 2

2 895 16 1535 1 3

2 896 8 1151 1 1

2 897 32 701 1 5

2 898 8 1170 1 2

2 899 16 966 1 3

2 900 32 858 1 3

2 901 8 1494 1 1

2 902 8 695 0 3

2 903 8 941 1 4

2 904 16 890 1 5

2 905 16 1267 1 4

2 906 32 1385 1 2

2 907 8 1148 1 4

2 908 8 783 1 3

2 909 32 1136 1 4

2 910 8 957 1 4

2 911 32 957 0 2

2 912 8 737 1 3

2 913 32 707 0 5

2 914 16 1095 1 1

2 915 8 775 1 2

2 916 8 856 1 1

2 917 8 537 1 3

2 918 8 850 1 1

2 919 32 1445 1 4

2 920 16 1013 1 3

2 921 32 1879 1 5

2 922 32 793 1 2

2 923 16 885 1 1

2 924 8 2087 1 4

2 925 16 1111 1 5

2 926 16 1093 1 2

2 927 32 1135 1 1

2 928 32 1209 1 1

2 929 16 1363 0 4

2 930 8 1059 1 5

2 931 16 1065 1 5

2 932 32 700 1 4

2 933 32 880 1 2

2 934 8 1401 0 2

2 935 8 804 1 4

2 936 16 831 1 5

2 937 8 1619 0 2

2 938 32 778 0 4

2 939 16 783 1 5

2 940 8 1497 0 2

2 941 8 922 1 1

2 942 32 1230 0 5

2 943 16 1215 1 4

2 944 16 1421 1 1

2 945 16 1326 1 4

2 946 16 907 1 1

2 947 32 948 1 3

2 948 16 607 1 3

2 949 8 925 1 3

2 950 32 1249 1 5

2 951 32 700 1 3

2 952 8 713 0 1

2 953 32 764 1 5

2 954 16 1112 1 5

2 955 16 1315 0 2

2 956 16 796 1 1

2 957 16 926 1 3

2 958 8 953 0 2

2 959 8 1298 1 2

2 960 8 634 1 3

2 961 8 1113 0 1

2 962 8 1712 1 1

2 963 8 653 0 4

2 964 32 706 0 5

2 965 16 2127 0 2

2 966 16 1266 1 5

2 967 16 938 1 1

2 968 16 1535 1 4

2 969 32 1059 1 2

2 970 8 909 1 4

2 971 16 1162 0 1

2 972 16 2052 1 5

2 973 8 826 1 5

2 974 8 1492 1 1

2 975 8 971 1 2

2 976 32 2737 1 5

2 977 32 1159 1 4

2 978 32 1242 1 2

2 979 16 985 1 5

2 980 8 994 1 2

2 981 16 1013 1 5

2 982 32 1046 1 5

2 983 8 885 0 3

2 984 32 1788 1 1

2 985 8 593 1 4

2 986 16 695 0 4

2 987 8 1646 0 2

2 988 16 1147 1 3

2 989 16 609 1 3

2 990 16 1054 0 4

2 991 32 721 0 4

2 992 16 1719 1 3

2 993 32 760 0 3

2 994 16 911 1 2

2 995 32 686 1 4

2 996 8 772 0 4

2 997 16 1930 1 4

2 998 32 599 1 3

2 999 32 534 1 4

2 1000 16 1572 1 4

2 1001 8 716 1 5

2 1002 32 984 1 5

2 1003 32 699 1 5

2 1004 8 720 1 5

2 1005 16 1802 1 4

2 1006 32 1597 1 3

2 1007 16 744 0 2

2 1008 16 941 0 2

2 1009 16 875 1 3

2 1010 8 735 1 3

2 1011 16 1888 0 2

2 1012 32 1066 1 1

2 1013 8 1266 1 2

2 1014 32 886 1 3

2 1015 8 1128 1 5

2 1016 8 1244 1 3

2 1017 8 862 0 5

2 1018 8 739 1 3

2 1019 32 1242 1 4

2 1020 8 923 1 3

2 1021 16 929 1 1

2 1022 32 1331 1 3

2 1023 16 799 1 3

2 1024 16 896 0 4

2 1025 32 913 1 2

2 1026 8 750 1 1

2 1027 8 671 1 5

2 1028 16 670 0 5

2 1029 16 764 1 5

2 1030 16 893 1 5

2 1031 8 1520 1 4

2 1032 8 999 1 3

2 1033 8 957 1 2

2 1034 8 1584 1 4

2 1035 8 920 1 1

2 1036 32 815 1 2

2 1037 32 1092 1 3

2 1038 8 1031 1 2

2 1039 32 557 1 1

2 1040 16 791 1 1

2 1041 8 949 1 5

2 1042 32 935 0 2

2 1043 32 612 0 1

2 1044 32 1667 1 1

2 1045 8 558 1 3

2 1046 32 771 0 5

2 1047 32 1388 1 1

2 1048 32 681 0 2

2 1049 8 966 1 5

2 1050 32 1110 1 4

2 1051 32 882 0 5

2 1052 32 684 1 4

2 1053 16 915 1 3

2 1054 16 1225 1 4

2 1055 16 800 1 2

2 1056 8 1787 1 1

2 1057 8 2071 1 1

2 1058 16 1511 1 2

2 1059 8 1336 1 1

2 1060 32 914 1 2

2 1061 16 1466 0 3

2 1062 8 680 1 4

2 1063 8 1055 1 4

2 1064 16 1241 1 1

2 1065 32 1422 0 1

2 1066 16 1176 1 1

2 1067 8 1185 0 2

2 1068 8 1363 1 2

2 1069 32 1208 1 3

2 1070 32 752 1 2

2 1071 32 1171 1 4

2 1072 32 824 1 3

2 1073 16 1343 0 1

2 1074 16 749 0 1

2 1075 16 1167 0 2

2 1076 8 642 0 3

2 1077 16 1143 1 3

2 1078 32 755 1 1

2 1079 32 936 1 5

2 1080 16 960 1 5

2 1081 8 1354 1 4

2 1082 16 824 0 3

2 1083 8 715 1 2

2 1084 8 521 1 4

2 1085 32 1343 1 2

2 1086 8 840 1 4

2 1087 16 1215 0 4

2 1088 8 1463 1 1

2 1089 32 946 0 2

2 1090 16 763 1 2

2 1091 32 826 0 5

2 1092 8 720 0 2

2 1093 8 1606 1 1

2 1094 32 845 1 3

2 1095 16 726 1 3

2 1096 32 820 0 3

2 1097 32 849 1 3

2 1098 16 900 1 1

2 1099 16 1012 0 5

2 1100 32 1469 1 3

2 1101 8 1096 1 1

2 1102 16 763 1 1

2 1103 16 2242 1 3

2 1104 32 1182 1 1

2 1105 16 1724 0 2

2 1106 32 2062 0 2

2 1107 8 1098 1 3

2 1108 16 933 1 5

2 1109 8 736 1 3

2 1110 8 788 1 5

2 1111 8 947 1 4

2 1112 32 1001 1 5

2 1113 16 753 0 4

2 1114 16 792 1 2

2 1115 32 903 1 1

2 1116 32 1384 1 5

2 1117 8 780 0 2

2 1118 32 1413 1 5

2 1119 16 1529 1 2

2 1120 16 1085 1 1

2 1121 16 997 1 1

2 1122 16 677 1 4

2 1123 8 596 1 5

2 1124 8 780 1 5

2 1125 8 1379 1 2

2 1126 8 712 1 2

2 1127 16 808 0 2

2 1128 16 1184 1 1

2 1129 8 749 1 5

2 1130 8 1442 1 5

2 1131 32 1049 1 1

2 1132 32 862 1 2

2 1133 32 864 1 2

2 1134 16 653 1 3

2 1135 32 1771 1 3

2 1136 32 1098 1 1

2 1137 32 1308 1 4

2 1138 16 1136 1 5

2 1139 16 954 1 5

2 1140 16 725 1 3

2 1141 32 1975 1 4

2 1142 16 762 0 4

2 1143 16 805 0 2

2 1144 16 1538 1 3

2 1145 32 723 1 4

2 1146 8 740 0 4

2 1147 8 1573 1 1

2 1148 16 815 1 1

2 1149 16 1067 1 5

2 1150 32 1026 1 1

2 1151 16 1510 1 3

2 1152 32 1315 1 5

2 1153 8 817 1 3

2 1154 8 770 1 1

2 1155 16 577 1 1

2 1156 16 733 1 4

2 1157 32 903 1 4

2 1158 16 692 0 2

2 1159 8 1223 0 4

2 1160 16 1018 0 4

2 1161 8 1221 1 1

2 1162 32 847 1 2

2 1163 32 927 1 1

2 1164 32 1235 1 5

2 1165 16 1094 0 2

2 1166 16 1153 1 1

2 1167 8 988 1 4

2 1168 16 1509 1 4

2 1169 8 837 1 3

2 1170 32 1074 1 3

2 1171 32 666 1 4

2 1172 8 1445 1 3

2 1173 16 787 1 3

2 1174 32 762 0 1

2 1175 8 1205 1 5

2 1176 8 1273 1 3

2 1177 16 831 1 5

2 1178 8 1220 1 2

2 1179 8 767 1 5

2 1180 32 743 1 5

2 1181 8 1121 1 5

2 1182 16 984 1 4

2 1183 8 1017 1 3

2 1184 8 899 1 4

2 1185 32 860 1 4

2 1186 32 736 1 2

2 1187 32 620 1 1

2 1188 8 864 1 2

2 1189 8 1519 1 1

2 1190 32 787 1 3

2 1191 16 786 1 5

2 1192 32 777 1 3

2 1193 32 670 1 2

2 1194 8 1064 0 1

2 1195 32 1109 0 4

2 1196 8 1147 1 2

2 1197 32 1005 1 4

2 1198 32 621 1 5

2 1199 16 890 1 5

2 1200 8 797 1 3

3 1 32 2328 1 2

3 2 8 1769 1 5

3 3 16 1554 1 4

3 4 32 1470 1 5

3 5 32 1260 1 3

3 6 32 1820 1 1

3 7 16 2227 1 3

3 8 8 1473 1 4

3 9 32 1005 1 2

3 10 16 1628 1 2

3 11 32 1300 1 3

3 12 16 1463 1 3

3 13 8 2336 1 1

3 14 32 1385 1 1

3 15 8 1447 1 5

3 16 16 1553 1 5

3 17 32 1697 1 1

3 18 32 1041 1 3

3 19 8 1552 1 3

3 20 32 1891 1 2

3 21 16 1723 1 4

3 22 8 1739 1 1

3 23 16 1136 0 4

3 24 16 1593 1 4

3 25 8 1065 1 2

3 26 16 1271 1 5

3 27 8 1156 1 2

3 28 16 1523 1 2

3 29 32 1306 1 3

3 30 16 1437 1 5

3 31 32 927 1 3

3 32 8 977 1 2

3 33 16 1444 0 1

3 34 16 2297 1 1

3 35 8 1179 1 1

3 36 32 1174 1 1

3 37 8 1288 1 5

3 38 8 1259 1 5

3 39 32 1311 1 2

3 40 32 1101 1 1

3 41 8 1163 1 3

3 42 8 867 1 5

3 43 32 1418 1 2

3 44 16 856 0 3

3 45 16 2254 1 2

3 46 16 1374 1 4

3 47 16 1165 1 5

3 48 16 1414 1 4

3 49 16 2718 1 1

3 50 32 1085 1 3

3 51 32 1214 1 1

3 52 8 1416 1 4

3 53 32 1432 1 5

3 54 16 1929 0 2

3 55 16 2053 1 1

3 56 32 1485 1 1

3 57 32 995 1 5

3 58 32 872 1 4

3 59 32 779 1 5

3 60 32 1029 1 5

3 61 16 2217 1 5

3 62 8 1457 1 4

3 63 8 1142 1 1

3 64 8 1050 1 2

3 65 16 1501 0 1

3 66 32 982 1 4

3 67 32 1511 1 2

3 68 32 1090 1 4

3 69 16 1517 1 5

3 70 16 1295 1 4

3 71 8 968 1 3

3 72 8 1458 1 4

3 73 32 946 1 3

3 74 16 2223 1 1

3 75 32 917 1 1

3 76 32 1099 1 4

3 77 16 891 1 5

3 78 16 1501 1 2

3 79 16 1092 1 3

3 80 8 894 1 5

3 81 32 907 1 4

3 82 8 2674 1 3

3 83 8 2615 1 4

3 84 32 766 1 3

3 85 8 1322 1 3

3 86 16 1380 1 3

3 87 16 1642 0 2

3 88 16 1526 0 3

3 89 16 1866 1 4

3 90 16 916 1 5

3 91 8 1074 1 4

3 92 32 801 1 2

3 93 8 1452 1 2

3 94 8 1931 1 1

3 95 16 1790 1 2

3 96 8 895 1 3

3 97 8 1121 1 1

3 98 8 1143 1 3

3 99 32 1054 1 4

3 100 8 1026 1 2

3 101 8 850 1 1

3 102 8 1135 1 5

3 103 16 1027 1 3

3 104 8 921 1 4

3 105 8 918 1 2

3 106 16 1155 0 1

3 107 32 1237 1 2

3 108 8 806 1 3

3 109 32 973 1 4

3 110 8 1304 1 4

3 111 32 849 1 4

3 112 8 865 1 5

3 113 16 894 1 3

3 114 32 872 1 5

3 115 32 1503 0 5

3 116 16 2805 0 2

3 117 16 5892 1 1

3 118 8 1091 1 2

3 119 32 1805 1 5

3 120 8 1574 1 1

3 121 8 1068 1 1

3 122 16 2590 1 4

3 123 8 866 0 2

3 124 16 966 1 5

3 125 16 1168 1 5

3 126 8 999 1 1

3 127 16 1207 0 2

3 128 8 848 1 3

3 129 32 840 1 5

3 130 8 1061 1 4

3 131 32 1310 0 4

3 132 32 1932 1 1

3 133 32 1082 1 4

3 134 8 2853 1 3

3 135 8 1036 1 1

3 136 32 841 1 4

3 137 16 961 1 5

3 138 8 824 1 3

3 139 32 1397 1 2

3 140 32 1191 1 5

3 141 32 1752 1 4

3 142 8 1344 1 1

3 143 32 1175 1 4

3 144 16 1375 1 1

3 145 8 1029 1 3

3 146 32 1111 1 2

3 147 8 1488 1 4

3 148 16 2365 1 2

3 149 8 853 1 5

3 150 32 1497 1 5

3 151 32 1344 1 5

3 152 8 1875 1 5

3 153 8 1029 1 5

3 154 16 1661 1 5

3 155 8 998 1 2

3 156 32 1064 1 2

3 157 8 847 1 2

3 158 32 1277 1 1

3 159 16 832 1 5

3 160 16 1327 0 2

3 161 32 909 1 4

3 162 8 1113 1 2

3 163 32 916 1 4

3 164 8 1175 1 2

3 165 16 1445 1 4

3 166 16 1208 0 4

3 167 32 1271 1 5

3 168 32 1200 1 5

3 169 16 1221 1 3

3 170 8 1976 1 1

3 171 16 1263 1 3

3 172 32 1094 1 5

3 173 16 1452 1 3

3 174 16 1186 1 3

3 175 32 1330 1 1

3 176 32 1231 1 5

3 177 8 1942 1 5

3 178 8 1014 1 2

3 179 16 1372 1 3

3 180 16 1067 0 1

3 181 32 1359 1 3

3 182 8 1437 1 4

3 183 32 929 1 3

3 184 8 1389 0 4

3 185 32 995 1 3

3 186 32 2604 1 2

3 187 32 1050 1 2

3 188 16 1440 1 5

3 189 8 1155 1 1

3 190 32 864 1 3

3 191 8 1104 1 5

3 192 8 1026 1 5

3 193 32 1072 1 3

3 194 32 1132 1 1

3 195 32 976 1 2

3 196 8 1017 1 2

3 197 16 1335 1 4

3 198 8 951 1 3

3 199 16 1338 1 4

3 200 16 1470 1 4

3 201 32 1081 1 2

3 202 16 1079 1 3

3 203 8 859 1 3

3 204 16 2127 1 4

3 205 32 1283 1 1

3 206 32 1137 1 1

3 207 8 1344 1 4

3 208 32 871 1 2

3 209 16 1059 1 2

3 210 32 842 1 1

3 211 16 1835 0 1

3 212 16 1986 1 1

3 213 16 1404 1 1

3 214 8 796 1 3

3 215 8 1023 1 4

3 216 16 1372 1 2

3 217 16 1472 1 1

3 218 8 1339 1 4

3 219 8 1584 1 4

3 220 32 768 1 1

3 221 16 1317 0 1

3 222 16 948 1 5

3 223 32 865 1 3

3 224 16 1272 0 2

3 225 32 853 1 3

3 226 16 1810 0 1

3 227 16 2936 1 3

3 228 8 891 1 3

3 229 16 1899 1 4

3 230 8 1355 1 1

3 231 16 886 1 5

3 232 8 918 1 5

3 233 32 1082 1 3

3 234 16 1295 1 3

3 235 16 1164 0 2

3 236 8 936 1 5

3 237 8 1186 1 2

3 238 32 911 1 4

3 239 8 1560 1 1

3 240 16 823 1 2

3 241 32 759 1 4

3 242 8 813 1 5

3 243 8 774 1 4

3 244 32 976 1 5

3 245 32 785 1 3

3 246 8 3437 1 1

3 247 16 1971 1 1

3 248 32 1246 1 1

3 249 32 742 1 2

3 250 32 753 1 3

3 251 8 996 1 1

3 252 16 1039 1 3

3 253 16 1192 1 5

3 254 32 725 1 4

3 255 16 835 1 5

3 256 16 1206 0 2

3 257 8 1355 1 4

3 258 8 982 1 4

3 259 32 1013 1 3

3 260 16 928 1 5

3 261 16 1188 1 4

3 262 8 1922 1 3

3 263 16 1247 1 3

3 264 16 1926 1 4

3 265 8 860 1 5

3 266 8 726 1 3

3 267 32 790 1 2

3 268 32 1111 1 1

3 269 8 1735 1 1

3 270 32 745 1 3

3 271 16 836 1 1

3 272 32 1030 1 5

3 273 8 923 1 3

3 274 8 1262 1 3

3 275 32 688 1 4

3 276 8 896 1 3

3 277 16 919 1 5

3 278 32 700 1 5

3 279 16 1053 0 2

3 280 16 1937 1 4

3 281 16 956 1 5

3 282 16 800 1 3

3 283 32 887 1 2

3 284 8 1004 1 2

3 285 8 858 1 5

3 286 8 914 1 2

3 287 16 959 1 4

3 288 32 1018 1 1

3 289 16 1241 1 4

3 290 8 928 1 4

3 291 32 951 1 4

3 292 8 977 1 4

3 293 32 1041 1 1

3 294 8 888 1 1

3 295 32 1101 1 1

3 296 16 1753 0 1

3 297 16 990 1 5

3 298 32 932 1 5

3 299 32 1979 1 3

3 300 8 964 1 5

3 301 8 1447 1 2

3 302 8 989 1 1

3 303 32 803 1 4

3 304 32 682 1 3

3 305 16 1255 1 5

3 306 8 879 1 2

3 307 32 862 1 2

3 308 8 904 1 4

3 309 32 1137 1 5

3 310 32 936 1 4

3 311 16 1026 1 3

3 312 32 971 1 4

3 313 8 969 1 2

3 314 8 952 1 5

3 315 16 1012 1 1

3 316 32 1033 1 3

3 317 32 1052 1 2

3 318 8 996 1 4

3 319 8 882 1 3

3 320 8 912 1 5

3 321 16 1061 1 3

3 322 16 1515 1 1

3 323 16 1236 1 3

3 324 8 775 1 2

3 325 32 874 0 1

3 326 16 1915 1 1

3 327 16 1190 1 1

3 328 8 802 1 4

3 329 32 857 1 4

3 330 16 1702 1 4

3 331 16 1055 1 2

3 332 8 958 1 1

3 333 16 1067 1 2

3 334 32 1013 1 5

3 335 16 1112 0 2

3 336 8 917 1 1

3 337 8 815 1 3

3 338 32 1162 1 2

3 339 8 903 1 1

3 340 32 1169 1 1

3 341 8 1047 1 2

3 342 16 1657 1 1

3 343 16 1714 1 2

3 344 32 854 1 2

3 345 8 6002 1 5

3 346 32 921 1 3

3 347 16 2756 1 3

3 348 32 1725 1 1

3 349 8 700 1 5

3 350 8 866 1 2

3 351 16 786 1 5

3 352 16 1272 1 4

3 353 16 888 1 3

3 354 16 988 1 4

3 355 16 1148 1 2

3 356 16 1409 0 2

3 357 32 1106 1 5

3 358 8 1058 1 3

3 359 32 1418 1 5

3 360 32 1167 1 2

3 361 8 915 1 5

3 362 32 877 1 2

3 363 8 947 1 4

3 364 32 1547 1 5

3 365 32 702 1 4

3 366 32 1009 1 5

3 367 8 895 1 5

3 368 32 1405 1 5

3 369 8 1142 1 2

3 370 32 1224 1 1

3 371 8 1501 1 1

3 372 8 700 1 3

3 373 16 1636 1 4

3 374 16 1165 1 2

3 375 32 784 1 3

3 376 32 1008 1 1

3 377 32 840 1 2

3 378 16 941 1 5

3 379 8 1068 1 3

3 380 32 845 1 3

3 381 16 1079 1 2

3 382 8 809 1 1

3 383 8 918 1 3

3 384 32 1123 1 5

3 385 8 1078 1 1

3 386 16 1045 1 3

3 387 16 1574 1 3

3 388 8 1447 1 4

3 389 16 880 1 5

3 390 16 1633 1 2

3 391 16 1560 1 4

3 392 8 1352 1 2

3 393 32 797 1 4

3 394 8 827 1 4

3 395 16 1412 1 4

3 396 16 1504 1 5

3 397 8 1039 1 2

3 398 32 922 1 5

3 399 8 733 1 2

3 400 32 765 1 3

3 401 16 980 1 5

3 402 8 841 1 4

3 403 8 675 1 3

3 404 32 719 1 3

3 405 8 651 1 5

3 406 32 830 1 1

3 407 8 824 0 5

3 408 32 1068 1 1

3 409 16 1593 1 3

3 410 16 2758 0 1

3 411 8 1031 1 2

3 412 8 954 1 1

3 413 32 816 1 4

3 414 16 2406 0 2

3 415 16 1481 1 4

3 416 32 851 1 5

3 417 32 862 1 4

3 418 8 803 1 3

3 419 32 893 1 1

3 420 32 784 1 5

3 421 8 1002 1 5

3 422 8 2512 1 4

3 423 16 1461 1 2

3 424 16 1132 0 2

3 425 32 1181 1 1

3 426 32 707 1 4

3 427 8 744 1 5

3 428 32 1005 1 2

3 429 16 1176 1 3

3 430 16 927 1 5

3 431 32 804 1 4

3 432 32 1327 1 3

3 433 8 1562 1 1

3 434 16 1737 0 3

3 435 32 1263 1 1

3 436 16 1980 0 1

3 437 16 1798 1 1

3 438 16 1616 1 4

3 439 8 1525 1 3

3 440 8 1235 1 3

3 441 16 1450 1 1

3 442 16 1822 1 1

3 443 16 880 1 3

3 444 32 668 1 3

3 445 32 718 1 3

3 446 32 876 1 1

3 447 16 1479 1 4

3 448 8 892 1 1

3 449 16 882 1 5

3 450 16 1213 1 4

3 451 16 1707 1 1

3 452 16 1033 1 1

3 453 8 1181 1 4

3 454 32 886 1 2

3 455 32 847 1 4

3 456 32 1091 1 3

3 457 8 853 1 2

3 458 32 761 1 4

3 459 8 1015 1 5

3 460 16 1952 1 2

3 461 32 775 1 2

3 462 16 1362 1 3

3 463 16 928 1 5

3 464 8 857 1 5

3 465 8 990 1 4

3 466 32 851 1 2

3 467 16 983 1 3

3 468 8 929 1 2

3 469 8 1113 1 1

3 470 32 843 1 2

3 471 16 1176 1 1

3 472 8 1444 1 3

3 473 8 1158 1 2

3 474 32 898 1 2

3 475 32 790 1 5

3 476 16 825 1 5

3 477 8 1030 1 1

3 478 8 2635 1 4

3 479 16 1353 0 4

3 480 16 1807 0 2

3 481 16 1388 1 1

3 482 8 872 1 4

3 483 32 819 1 2

3 484 16 750 1 5

3 485 32 718 1 4

3 486 16 1515 1 1

3 487 32 1085 1 2

3 488 32 836 1 3

3 489 16 1654 1 4

3 490 32 1370 1 1

3 491 16 1604 1 1

3 492 8 715 1 3

3 493 8 830 1 2

3 494 32 961 1 5

3 495 8 865 1 5

3 496 8 969 1 1

3 497 32 854 1 3

3 498 8 774 1 1

3 499 8 823 1 2

3 500 8 825 1 4

3 501 16 826 1 5

3 502 16 1370 1 4

3 503 16 1859 1 1

3 504 8 3859 1 4

3 505 32 811 1 3

3 506 16 1122 1 1

3 507 8 817 1 5

3 508 32 834 1 5

3 509 8 783 1 5

3 510 32 918 1 1

3 511 16 876 1 5

3 512 32 806 1 1

3 513 16 1083 1 3

3 514 8 819 1 3

3 515 8 891 1 5

3 516 8 911 1 2

3 517 16 1083 1 3

3 518 8 1064 1 3

3 519 8 793 1 2

3 520 8 710 1 2

3 521 16 1298 1 2

3 522 8 668 1 3

3 523 32 776 1 5

3 524 16 978 1 2

3 525 8 1352 1 2

3 526 16 883 1 5

3 527 8 1035 0 3

3 528 16 1293 1 2

3 529 32 923 1 5

3 530 32 914 1 5

3 531 8 1003 1 3

3 532 16 904 1 3

3 533 16 3037 1 5

3 534 16 807 1 5

3 535 16 1054 1 3

3 536 16 776 1 1

3 537 32 1611 1 3

3 538 16 1231 1 4

3 539 8 787 1 1

3 540 32 3998 1 4

3 541 32 921 1 4

3 542 32 752 1 2

3 543 16 1449 0 3

3 544 8 1209 1 4

3 545 32 878 1 2

3 546 32 708 1 4

3 547 16 1580 1 4

3 548 32 732 1 4

3 549 8 1471 1 1

3 550 32 747 1 2

3 551 32 775 1 5

3 552 32 1009 1 2

3 553 32 807 1 4

3 554 32 838 1 3

3 555 32 993 1 2

3 556 16 1361 1 4

3 557 8 1205 1 4

3 558 8 893 1 3

3 559 16 916 1 3

3 560 16 841 1 5

3 561 16 1481 1 4

3 562 16 865 1 5

3 563 16 3619 1 3

3 564 16 1528 0 2

3 565 32 905 1 1

3 566 32 1017 1 4

3 567 8 2768 1 4

3 568 8 1888 1 5

3 569 8 864 1 1

3 570 16 1826 1 1

3 571 16 903 1 1

3 572 32 976 1 1

3 573 16 1590 1 4

3 574 16 1987 1 2

3 575 8 761 1 4

3 576 8 779 1 2

3 577 32 849 1 1

3 578 16 1175 0 2

3 579 8 1029 1 5

3 580 8 776 1 1

3 581 32 874 1 5

3 582 32 717 1 3

3 583 32 1105 1 5

3 584 8 1303 1 5

3 585 32 1063 1 2

3 586 16 1197 1 4

3 587 32 874 1 1

3 588 8 1069 1 4

3 589 32 827 1 3

3 590 16 909 1 2

3 591 16 1215 1 2

3 592 32 1064 1 1

3 593 32 785 1 4

3 594 8 921 1 1

3 595 8 937 1 5

3 596 8 810 1 3

3 597 8 1008 1 1

3 598 32 752 1 3

3 599 8 1136 1 2

3 600 16 770 1 3

3 601 8 1104 1 4

3 602 16 968 1 4

3 603 8 702 1 5

3 604 8 968 1 2

3 605 8 718 1 5

3 606 32 937 1 5

3 607 32 897 1 5

3 608 16 1207 1 4

3 609 16 3209 1 2

3 610 8 840 1 2

3 611 32 893 1 2

3 612 16 932 1 3

3 613 8 756 1 3

3 614 16 959 1 2

3 615 8 741 1 5

3 616 8 783 1 4

3 617 8 799 1 4

3 618 16 772 1 5

3 619 8 733 1 1

3 620 32 655 1 4

3 621 16 895 1 3

3 622 16 712 1 5

3 623 8 741 1 3

3 624 8 869 1 1

3 625 8 904 1 2

3 626 8 762 1 2

3 627 8 760 1 1

3 628 32 744 1 3

3 629 16 1103 1 1

3 630 16 1064 1 2

3 631 16 952 1 4

3 632 16 961 1 2

3 633 32 982 1 5

3 634 8 848 1 4

3 635 32 727 1 1

3 636 16 846 1 4

3 637 16 708 1 5

3 638 16 952 1 2

3 639 8 694 1 5

3 640 16 705 0 4

3 641 8 1015 1 4

3 642 8 711 1 5

3 643 32 724 1 4

3 644 32 787 1 3

3 645 8 1063 1 1

3 646 16 1031 1 2

3 647 16 1120 1 3

3 648 32 784 1 3

3 649 32 944 1 2

3 650 32 659 1 4

3 651 16 931 1 3

3 652 32 715 1 4

3 653 32 696 1 3

3 654 16 2090 1 4

3 655 16 1176 1 1

3 656 32 953 1 3

3 657 32 882 1 2

3 658 16 1518 1 1

3 659 16 1358 1 2

3 660 8 946 0 1

3 661 16 974 1 3

3 662 8 831 1 3

3 663 16 1177 1 5

3 664 8 700 1 3

3 665 16 1043 1 1

3 666 16 1360 1 1

3 667 8 773 1 2

3 668 16 1364 1 4

3 669 16 819 1 5

3 670 32 900 1 1

3 671 8 756 1 1

3 672 16 794 1 4

3 673 16 850 1 1

3 674 32 830 1 1

3 675 16 920 1 3

3 676 32 894 1 2

3 677 8 995 1 4

3 678 8 811 1 5

3 679 32 780 1 2

3 680 32 643 1 3

3 681 32 664 1 5

3 682 32 691 1 5

3 683 32 732 1 5

3 684 32 907 1 2

3 685 8 869 1 1

3 686 16 909 1 2

3 687 8 838 1 2

3 688 32 736 1 3

3 689 32 608 1 3

3 690 16 703 1 5

3 691 32 837 1 2

3 692 32 653 1 4

3 693 8 683 1 3

3 694 16 894 0 1

3 695 32 845 1 1

3 696 32 856 1 4

3 697 32 853 1 4

3 698 8 901 1 3

3 699 8 757 1 3

3 700 16 832 1 3

3 701 32 989 1 1

3 702 16 863 1 3

3 703 32 805 1 2

3 704 32 2325 1 1

3 705 16 1584 1 5

3 706 16 1258 1 1

3 707 32 957 1 1

3 708 8 1170 0 4

3 709 8 893 1 2

3 710 32 977 1 1

3 711 16 984 1 5

3 712 8 1159 1 1

3 713 32 845 1 5

3 714 8 913 1 2

3 715 8 840 1 5

3 716 32 712 1 5

3 717 8 784 1 4

3 718 32 1562 1 4

3 719 8 838 1 3

3 720 8 794 1 5

3 721 8 919 1 2

3 722 16 842 1 3

3 723 8 1247 1 4

3 724 16 626 1 5

3 725 32 893 1 1

3 726 32 744 1 5

3 727 32 1011 1 1

3 728 16 991 1 4

3 729 32 732 1 5

3 730 8 721 1 4

3 731 16 675 1 1

3 732 8 702 1 3

3 733 8 663 1 3

3 734 16 964 1 2

3 735 8 905 1 2

3 736 8 796 1 4

3 737 8 634 1 1

3 738 16 759 1 5

3 739 16 749 0 4

3 740 16 1051 1 5

3 741 16 1081 1 1

3 742 16 1066 0 1

3 743 16 1787 1 1

3 744 8 839 1 1

3 745 32 762 1 5

3 746 16 874 1 3

3 747 32 1013 1 1

3 748 16 667 1 4

3 749 32 662 1 5

3 750 32 550 1 4

3 751 8 739 1 4

3 752 16 737 1 3

3 753 32 747 1 4

3 754 32 721 1 1

3 755 16 640 1 5

3 756 16 646 1 2

3 757 8 733 1 4

3 758 16 779 1 3

3 759 8 718 1 3

3 760 32 826 1 5

3 761 8 960 1 2

3 762 32 758 1 5

3 763 32 696 1 3

3 764 32 929 1 1

3 765 8 1453 1 3

3 766 16 947 1 4

3 767 16 1001 1 4

3 768 16 1328 1 4

3 769 16 1185 1 3

3 770 16 893 1 3

3 771 32 901 1 1

3 772 32 752 1 2

3 773 32 669 1 3

3 774 8 743 1 1

3 775 8 791 1 3

3 776 8 742 1 5

3 777 32 1054 1 3

3 778 32 732 1 2

3 779 16 1168 0 2

3 780 16 803 1 1

3 781 32 895 1 3

3 782 32 1065 1 4

3 783 16 1443 1 1

3 784 32 803 1 3

3 785 32 1216 1 4

3 786 16 662 1 2

3 787 32 765 1 2

3 788 8 870 1 5

3 789 8 747 1 5

3 790 8 808 1 5

3 791 32 990 1 3

3 792 32 672 1 4

3 793 16 1561 1 1

3 794 8 1352 1 3

3 795 32 1553 1 5

3 796 16 1054 1 3

3 797 8 913 1 4

3 798 32 856 1 2

3 799 8 966 1 1

3 800 32 1133 1 1

3 801 32 1274 1 4

3 802 32 987 1 2

3 803 16 1158 1 5

3 804 16 990 1 3

3 805 8 1131 1 4

3 806 8 739 1 5

3 807 32 1213 1 1

3 808 8 789 1 1

3 809 16 1300 1 1

3 810 8 1073 1 2

3 811 32 732 1 3

3 812 8 884 1 5

3 813 8 1001 1 4

3 814 32 1189 1 2

3 815 8 818 1 5

3 816 16 1542 1 4

3 817 16 718 1 5

3 818 32 825 1 2

3 819 8 695 1 2

3 820 16 1101 0 2

3 821 32 1011 1 2

3 822 16 1037 1 4

3 823 8 847 1 2

3 824 16 832 1 5

3 825 8 757 1 5

3 826 8 967 1 1

3 827 32 768 1 4

3 828 32 673 1 5

3 829 16 1205 1 2

3 830 8 747 1 3

3 831 32 734 1 3

3 832 8 734 0 2

3 833 8 775 1 3

3 834 32 669 1 4

3 835 16 604 1 5

3 836 16 1047 0 2

3 837 8 3892 1 1

3 838 8 980 1 1

3 839 16 905 1 2

3 840 8 1003 0 2

3 841 16 1843 1 1

3 842 8 667 1 5

3 843 16 823 1 5

3 844 32 944 1 3

3 845 32 722 1 4

3 846 8 941 1 1

3 847 16 686 1 3

3 848 16 1929 1 4

3 849 8 834 1 2

3 850 8 820 1 3

3 851 32 884 1 1

3 852 32 1016 1 1

3 853 32 1123 1 1

3 854 16 905 0 2

3 855 16 1294 1 4

3 856 16 943 1 2

3 857 32 768 1 3

3 858 16 1264 1 1

3 859 8 826 1 4

3 860 16 1349 1 4

3 861 32 753 1 3

3 862 16 757 1 3

3 863 8 652 1 4

3 864 8 1945 1 4

3 865 32 683 1 4

3 866 8 720 1 3

3 867 16 934 1 5

3 868 32 1044 1 3

3 869 32 729 1 3

3 870 8 848 1 2

3 871 8 698 1 4

3 872 16 910 1 4

3 873 8 604 1 2

3 874 32 670 1 2

3 875 16 1000 1 5

3 876 16 991 1 1

3 877 8 754 0 2

3 878 32 1542 1 4

3 879 32 918 1 5

3 880 32 772 1 4

3 881 16 1180 1 1

3 882 32 887 1 4

3 883 8 926 1 1

3 884 32 709 1 5

3 885 16 936 1 5

3 886 8 1118 1 1

3 887 16 1137 1 5

3 888 32 812 1 2

3 889 32 804 1 5

3 890 8 739 1 1

3 891 16 1193 1 2

3 892 8 765 1 5

3 893 8 699 1 1

3 894 8 928 1 2

3 895 8 789 1 2

3 896 32 721 1 4

3 897 8 707 1 4

3 898 8 640 1 5

3 899 32 1121 1 2

3 900 8 894 1 2

3 901 8 861 1 3

3 902 32 745 1 1

3 903 16 879 1 5

3 904 32 847 1 4

3 905 32 750 1 5

3 906 32 972 1 1

3 907 8 615 1 1

3 908 16 765 1 1

3 909 32 770 1 4

3 910 32 869 1 3

3 911 16 737 1 5

3 912 8 786 1 3

3 913 32 962 1 5

3 914 32 896 1 2

3 915 32 675 1 1

3 916 8 728 1 5

3 917 8 678 1 1

3 918 32 694 1 1

3 919 16 897 1 4

3 920 16 912 1 3

3 921 16 1102 1 3

3 922 8 666 1 5

3 923 16 819 1 3

3 924 16 765 1 2

3 925 32 792 1 1

3 926 16 1117 1 1

3 927 16 893 1 4

3 928 16 1242 1 3

3 929 8 665 1 4

3 930 16 675 1 5

3 931 8 592 1 5

3 932 32 948 1 5

3 933 32 744 1 2

3 934 16 929 1 4

3 935 32 737 1 3

3 936 8 691 1 5

3 937 8 810 1 4

3 938 16 874 1 2

3 939 32 726 1 5

3 940 8 874 1 4

3 941 32 1073 1 3

3 942 8 980 1 1

3 943 8 840 1 5

3 944 32 768 1 2

3 945 16 1192 1 1

3 946 16 853 1 2

3 947 16 826 1 3

3 948 32 653 1 5

3 949 16 1271 1 1

3 950 32 789 1 2

3 951 16 1341 0 2

3 952 32 811 1 2

3 953 8 976 1 3

3 954 8 610 1 3

3 955 16 792 1 3

3 956 8 745 1 3

3 957 16 1500 1 4

3 958 8 590 1 2

3 959 16 856 0 2

3 960 8 715 1 3

3 961 16 1067 1 3

3 962 16 1451 1 2

3 963 32 874 1 2

3 964 32 735 1 5

3 965 8 633 1 3

3 966 16 679 1 5

3 967 8 702 1 1

3 968 16 878 1 1

3 969 8 1209 0 1

3 970 8 893 1 2

3 971 8 844 1 2

3 972 8 834 1 4

3 973 16 1254 1 4

3 974 32 665 1 5

3 975 8 676 1 3

3 976 8 705 1 2

3 977 32 581 1 1

3 978 8 537 1 2

3 979 8 603 1 5

3 980 32 580 1 1

3 981 32 606 1 4

3 982 16 598 1 5

3 983 16 1645 1 4

3 984 8 828 1 4

3 985 16 603 1 5

3 986 16 777 1 4

3 987 32 771 1 2

3 988 16 817 1 3

3 989 32 865 0 1

3 990 8 813 1 3

3 991 32 733 1 4

3 992 16 1379 0 2

3 993 16 1358 1 4

3 994 16 1158 1 2

3 995 32 887 1 1

3 996 8 925 1 4

3 997 8 765 1 3

3 998 32 798 1 3

3 999 32 819 1 3

3 1000 16 1250 1 1

3 1001 8 3345 1 4

3 1002 16 786 1 3

3 1003 8 901 1 5

3 1004 8 919 1 5

3 1005 16 938 1 3

3 1006 32 901 1 5

3 1007 32 815 1 3

3 1008 16 1295 1 1

3 1009 32 731 1 5

3 1010 16 1191 1 3

3 1011 16 1903 0 2

3 1012 16 1304 1 4

3 1013 8 771 1 3

3 1014 16 1415 0 2

3 1015 16 1535 0 2

3 1016 16 1750 1 4

3 1017 32 748 1 1

3 1018 32 739 1 3

3 1019 8 811 1 5

3 1020 16 754 0 5

3 1021 16 1143 1 3

3 1022 32 769 1 1

3 1023 32 833 1 4

3 1024 32 923 1 2

3 1025 32 714 1 2

3 1026 32 855 1 5

3 1027 8 984 1 5

3 1028 8 715 1 5

3 1029 32 904 1 5

3 1030 8 1087 1 2

3 1031 32 907 1 3

3 1032 32 1006 1 4

3 1033 32 1059 1 4

3 1034 8 821 1 1

3 1035 8 1062 1 4

3 1036 8 2032 1 1

3 1037 8 781 1 4

3 1038 32 663 1 4

3 1039 16 1134 1 2

3 1040 8 729 1 2

3 1041 32 694 1 1

3 1042 16 942 1 1

3 1043 32 1063 1 2

3 1044 32 818 1 5

3 1045 16 1343 1 1

3 1046 8 762 1 3

3 1047 16 986 1 3

3 1048 16 1203 1 1

3 1049 16 1067 1 1

3 1050 32 870 1 5

3 1051 8 904 1 3

3 1052 16 1026 1 1

3 1053 8 1073 1 3

3 1054 8 838 1 1

3 1055 8 1119 1 1

3 1056 32 740 1 3

3 1057 16 718 1 5

3 1058 32 668 1 4

3 1059 8 683 1 2

3 1060 32 786 1 3

3 1061 8 756 1 4

3 1062 32 730 1 1

3 1063 16 665 1 5

3 1064 8 1233 1 1

3 1065 8 906 1 5

3 1066 32 1163 1 3

3 1067 32 1143 1 4

3 1068 8 720 1 1

3 1069 16 847 1 3

3 1070 16 1198 1 4

3 1071 16 1201 1 5

3 1072 16 762 1 5

3 1073 16 1501 1 2

3 1074 8 1203 1 4

3 1075 16 874 1 4

3 1076 8 750 0 2

3 1077 32 1179 1 2

3 1078 32 725 1 2

3 1079 8 1043 1 5

3 1080 32 976 1 2

3 1081 32 682 1 4

3 1082 32 657 1 4

3 1083 32 660 1 3

3 1084 16 1141 1 1

3 1085 16 983 1 2

3 1086 16 1485 0 4

3 1087 32 2168 1 3

3 1088 8 1030 1 3

3 1089 32 1541 1 5

3 1090 16 1267 1 1

3 1091 16 1365 1 3

3 1092 32 1288 1 3

3 1093 16 1236 1 2

3 1094 16 707 1 5

3 1095 8 679 1 2

3 1096 32 750 1 1

3 1097 32 796 1 2

3 1098 8 690 1 1

3 1099 32 697 1 4

3 1100 16 851 1 1

3 1101 32 689 1 4

3 1102 32 864 1 2

3 1103 16 826 1 1

3 1104 32 1315 1 3

3 1105 8 802 1 1

3 1106 8 738 1 3

3 1107 16 772 1 5

3 1108 16 811 1 3

3 1109 32 1128 1 2

3 1110 8 697 1 2

3 1111 32 683 1 5

3 1112 16 1025 1 2

3 1113 16 956 1 3

3 1114 8 736 1 3

3 1115 16 682 1 5

3 1116 8 843 1 2

3 1117 8 794 1 4

3 1118 16 774 1 4

3 1119 8 745 1 4

3 1120 32 683 1 4

3 1121 32 845 1 4

3 1122 32 828 1 1

3 1123 8 705 1 5

3 1124 8 847 1 1

3 1125 32 728 1 3

3 1126 8 664 1 5

3 1127 32 667 1 3

3 1128 32 769 1 1

3 1129 32 718 1 2

3 1130 32 718 1 1

3 1131 32 621 1 5

3 1132 16 743 0 3

3 1133 16 733 1 1

3 1134 8 710 1 3

3 1135 16 704 1 1

3 1136 8 764 1 3

3 1137 8 637 1 2

3 1138 16 839 1 5

3 1139 16 1168 1 4

3 1140 16 905 0 2

3 1141 32 1189 1 1

3 1142 16 746 1 4

3 1143 16 1065 1 1

3 1144 8 719 1 4

3 1145 32 729 1 1

3 1146 32 727 1 3

3 1147 8 784 1 2

3 1148 8 661 1 4

3 1149 8 655 1 5

3 1150 16 1185 1 2

3 1151 16 674 1 5

3 1152 16 1206 1 2

3 1153 32 773 1 5

3 1154 8 779 1 5

3 1155 8 1375 1 2

3 1156 8 2056 1 1

3 1157 8 731 1 5

3 1158 8 907 1 4

3 1159 32 675 1 5

3 1160 16 913 1 4

3 1161 16 845 1 4

3 1162 32 814 1 2

3 1163 16 712 1 5

3 1164 16 681 1 3

3 1165 8 609 1 5

3 1166 16 665 1 5

3 1167 32 953 1 4

3 1168 8 680 1 5

3 1169 32 793 1 5

3 1170 32 691 1 3

3 1171 32 647 1 1

3 1172 8 652 1 3

3 1173 8 801 1 1

3 1174 32 749 1 5

3 1175 8 790 1 2

3 1176 16 4681 1 2

3 1177 16 649 1 5

3 1178 32 670 1 2

3 1179 16 1406 1 1

3 1180 32 685 1 5

3 1181 16 715 1 3

3 1182 8 598 1 1

3 1183 16 947 1 2

3 1184 8 796 1 1

3 1185 8 780 1 5

3 1186 16 890 1 3

3 1187 16 783 1 3

3 1188 16 1145 1 4

3 1189 32 958 1 1

3 1190 8 594 1 3

3 1191 8 829 1 2

3 1192 16 1101 1 4

3 1193 8 972 0 4

3 1194 8 942 1 1

3 1195 8 706 1 4

3 1196 8 690 1 4

3 1197 8 746 1 3

3 1198 32 833 1 4

3 1199 32 645 1 2

3 1200 32 1222 1 2

4 1 8 1504 1 3

4 2 16 1270 0 2

4 3 16 2210 1 4

4 4 8 951 1 3

4 5 8 1272 1 2

4 6 16 1027 1 2

4 7 32 1056 1 5

4 8 16 1142 1 3

4 9 32 1104 1 5

4 10 16 1008 1 3

4 11 16 1186 1 3

4 12 16 809 1 3

4 13 16 1545 1 4

4 14 32 804 1 4

4 15 16 1043 1 5

4 16 8 838 1 5

4 17 32 1436 1 1

4 18 8 1028 1 5

4 19 16 1267 1 2

4 20 16 2284 0 2

4 21 8 1108 0 1

4 22 16 2059 1 3

4 23 8 1363 1 5

4 24 8 1264 1 4

4 25 32 963 1 3

4 26 8 1040 1 2

4 27 16 1127 1 5

4 28 16 2290 1 2

4 29 32 925 1 1

4 30 16 1054 1 5

4 31 8 2611 1 1

4 32 16 1060 0 4

4 33 32 985 1 1

4 34 8 1488 1 3

4 35 16 1633 1 4

4 36 32 1625 1 5

4 37 8 945 1 3

4 38 8 738 1 4

4 39 16 1979 1 1

4 40 32 1130 1 2

4 41 32 1305 1 4

4 42 32 824 1 4

4 43 32 897 1 1

4 44 8 922 1 5

4 45 32 1019 1 4

4 46 32 1121 1 4

4 47 8 1219 1 1

4 48 32 1319 1 2

4 49 16 1258 0 2

4 50 8 749 1 3

4 51 32 1141 1 4

4 52 8 1236 1 3

4 53 8 1322 1 4

4 54 32 873 1 2

4 55 32 683 1 3

4 56 16 672 1 2

4 57 16 1551 1 4

4 58 16 903 1 5

4 59 32 2623 1 1

4 60 32 766 1 2

4 61 16 1337 1 1

4 62 32 927 1 5

4 63 8 649 1 5

4 64 16 1323 1 1

4 65 8 725 0 5

4 66 8 1522 0 1

4 67 8 1608 1 4

4 68 16 1754 1 3

4 69 16 907 0 4

4 70 32 1408 1 3

4 71 16 1458 1 3

4 72 8 1392 1 1

4 73 32 896 1 5

4 74 16 1165 1 5

4 75 32 734 1 4

4 76 8 1036 1 2

4 77 32 1100 1 2

4 78 16 784 0 5

4 79 8 1195 1 2

4 80 32 1577 1 2

4 81 16 758 1 1

4 82 16 1469 1 1

4 83 8 1051 1 4

4 84 16 2229 1 1

4 85 16 746 1 4

4 86 8 1101 1 5

4 87 8 1406 1 1

4 88 32 644 1 3

4 89 8 626 1 2

4 90 8 1394 1 1

4 91 32 764 1 3

4 92 32 592 1 1

4 93 16 4419 0 2

4 94 32 1090 1 5

4 95 32 1483 1 2

4 96 8 1324 1 4

4 97 16 1188 1 1

4 98 8 806 1 3

4 99 16 2593 1 5

4 100 32 1013 1 2

4 101 32 768 1 3

4 102 32 911 1 3

4 103 8 854 1 2

4 104 8 1030 1 1

4 105 16 1221 1 1

4 106 8 638 1 3

4 107 32 875 1 3

4 108 32 763 0 4

4 109 32 862 1 1

4 110 16 823 1 3

4 111 32 923 1 5

4 112 8 1270 1 4

4 113 16 1317 1 4

4 114 32 947 1 5

4 115 16 688 1 5

4 116 8 730 0 2

4 117 8 1089 1 4

4 118 8 1032 1 5

4 119 8 1487 1 2

4 120 32 1041 1 1

4 121 32 675 1 4

4 122 16 1153 1 4

4 123 32 707 0 4

4 124 8 677 1 2

4 125 32 797 1 1

4 126 16 1287 1 3

4 127 16 1477 1 2

4 128 8 660 0 2

4 129 32 1034 1 5

4 130 16 2312 1 2

4 131 32 1194 1 1

4 132 16 1382 1 1

4 133 8 1508 1 4

4 134 8 1217 1 1

4 135 8 932 1 3

4 136 16 2371 1 1

4 137 8 1226 1 4

4 138 8 1023 1 1

4 139 16 2260 1 1

4 140 32 1004 1 2

4 141 16 1258 1 4

4 142 8 1220 1 1

4 143 8 615 1 3

4 144 16 620 1 5

4 145 32 640 1 4

4 146 8 783 1 5

4 147 32 794 1 5

4 148 32 951 1 5

4 149 32 1372 1 1

4 150 16 785 0 5

4 151 16 2437 1 2

4 152 32 685 1 4

4 153 8 908 1 5

4 154 32 777 1 3

4 155 8 1218 1 1

4 156 32 980 1 2

4 157 8 774 1 3

4 158 32 627 1 1

4 159 16 768 1 5

4 160 8 721 1 5

4 161 32 1063 1 4

4 162 32 858 1 5

4 163 16 1201 1 2

4 164 16 2441 1 1

4 165 32 1205 1 2

4 166 32 775 1 4

4 167 8 985 1 4

4 168 32 986 1 1

4 169 32 1310 1 3

4 170 16 1160 1 1

4 171 16 1518 1 4

4 172 8 1271 1 4

4 173 32 1050 1 1

4 174 8 1529 0 1

4 175 32 768 1 3

4 176 8 890 1 4

4 177 8 1229 1 4

4 178 8 1028 0 1

4 179 8 993 1 3

4 180 16 1323 1 4

4 181 8 812 1 4

4 182 32 877 1 5

4 183 16 1312 0 1

4 184 16 1229 1 5

4 185 8 708 1 3

4 186 8 635 1 1

4 187 16 1119 1 3

4 188 8 1970 1 2

4 189 32 708 1 2

4 190 32 668 1 2

4 191 8 810 1 3

4 192 8 691 1 2

4 193 8 722 1 5

4 194 8 947 1 3

4 195 8 784 1 2

4 196 32 691 1 2

4 197 16 987 1 3

4 198 16 801 1 5

4 199 32 739 0 3

4 200 8 790 1 5

4 201 32 863 1 4

4 202 16 802 1 4

4 203 16 947 1 3

4 204 8 1120 1 2

4 205 8 681 1 5

4 206 32 849 1 1

4 207 32 859 1 2

4 208 16 1483 1 1

4 209 16 893 1 3

4 210 16 1070 1 3

4 211 32 1433 1 5

4 212 8 760 1 5

4 213 8 928 1 4

4 214 8 711 1 3

4 215 32 855 1 5

4 216 16 1424 1 4

4 217 16 1058 1 1

4 218 16 710 1 3

4 219 16 973 1 3

4 220 32 1298 1 3

4 221 16 1408 1 2

4 222 16 1397 1 5

4 223 32 931 1 1

4 224 8 1288 1 2

4 225 16 1875 1 4

4 226 32 880 1 5

4 227 16 2473 0 2

4 228 16 2080 1 2

4 229 16 928 0 4

4 230 8 1319 1 1

4 231 16 1729 0 2

4 232 32 978 1 2

4 233 16 733 1 5

4 234 8 707 1 5

4 235 32 844 1 3

4 236 8 1163 1 2

4 237 32 1067 1 4

4 238 32 689 1 3

4 239 16 746 1 5

4 240 32 997 1 3

4 241 8 1220 1 2

4 242 32 1377 1 1

4 243 8 701 1 4

4 244 32 887 1 3

4 245 32 759 1 3

4 246 32 653 1 1

4 247 16 1040 1 3

4 248 32 665 1 2

4 249 32 811 1 1

4 250 8 1092 1 5

4 251 8 865 1 3

4 252 32 1003 1 5

4 253 16 1032 1 2

4 254 8 657 1 2

4 255 8 634 1 4

4 256 8 880 1 1

4 257 8 663 1 4

4 258 16 928 0 2

4 259 8 1716 1 4

4 260 8 1112 1 1

4 261 8 749 1 5

4 262 32 1033 1 1

4 263 16 1713 1 2

4 264 16 1092 0 4

4 265 32 1412 1 5

4 266 16 1390 0 1

4 267 32 1197 1 2

4 268 8 1160 1 5

4 269 32 1195 1 3

4 270 8 1111 1 5

4 271 8 1728 1 1

4 272 16 1172 1 3

4 273 16 716 1 5

4 274 32 1261 1 2

4 275 8 1092 1 4

4 276 16 1632 1 1

4 277 32 827 1 5

4 278 16 1819 1 2

4 279 8 781 1 4

4 280 16 1052 1 2

4 281 8 1364 1 1

4 282 16 755 1 2

4 283 32 904 1 1

4 284 16 1556 1 3

4 285 8 834 1 4

4 286 8 410 1 3

4 287 32 782 1 4

4 288 32 842 1 3

4 289 8 1256 1 1

4 290 16 1934 1 4

4 291 32 1047 1 2

4 292 8 874 1 4

4 293 32 1092 1 2

4 294 16 1271 0 1

4 295 32 1151 1 5

4 296 8 1066 1 2

4 297 32 1103 1 3

4 298 16 1104 1 1

4 299 32 691 1 3

4 300 16 1304 1 4

4 301 16 1250 1 5

4 302 32 1040 1 5

4 303 32 665 0 4

4 304 16 1239 1 1

4 305 32 957 1 2

4 306 32 806 1 4

4 307 16 1578 0 1

4 308 32 1292 1 1

4 309 32 862 1 1

4 310 8 1192 1 1

4 311 32 1074 1 2

4 312 32 1285 1 1

4 313 8 1464 0 1

4 314 32 1028 1 2

4 315 16 1507 1 4

4 316 8 711 1 3

4 317 8 868 1 2

4 318 8 1054 1 2

4 319 16 1310 1 1

4 320 16 868 1 5

4 321 16 1103 1 4

4 322 32 937 1 5

4 323 16 929 1 3

4 324 32 786 1 5

4 325 16 1253 1 3

4 326 8 788 1 5

4 327 16 704 1 5

4 328 32 721 1 3

4 329 32 738 1 4

4 330 16 954 1 4

4 331 8 720 1 3

4 332 8 768 1 1

4 333 32 958 1 4

4 334 8 1509 1 2

4 335 8 689 1 3

4 336 16 609 1 3

4 337 8 800 1 2

4 338 16 1046 1 1

4 339 8 734 1 5

4 340 16 591 1 5

4 341 8 732 1 5

4 342 16 612 1 5

4 343 16 723 1 3

4 344 16 1414 1 2

4 345 16 1181 1 4

4 346 16 618 0 3

4 347 8 957 1 5

4 348 32 761 1 4

4 349 32 744 1 4

4 350 32 715 1 3

4 351 8 903 1 3

4 352 16 696 1 5

4 353 16 738 1 5

4 354 16 1352 1 4

4 355 8 687 1 3

4 356 32 702 1 5

4 357 8 1371 1 3

4 358 32 859 1 4

4 359 8 678 0 2

4 360 16 1083 0 2

4 361 16 1135 1 3

4 362 8 849 1 5

4 363 16 695 1 5

4 364 8 673 1 4

4 365 8 733 1 5

4 366 32 1376 1 2

4 367 8 716 1 3

4 368 8 742 1 2

4 369 32 834 1 2

4 370 16 647 0 5

4 371 32 1143 1 3

4 372 32 966 1 3

4 373 32 1936 1 5

4 374 32 709 1 5

4 375 16 1014 1 4

4 376 32 953 1 1

4 377 16 852 1 3

4 378 8 676 1 3

4 379 8 1773 1 1

4 380 32 1275 1 1

4 381 16 752 1 3

4 382 8 981 1 2

4 383 32 767 1 1

4 384 32 1205 1 5

4 385 16 964 1 3

4 386 16 677 0 5

4 387 32 1298 1 2

4 388 16 884 1 2

4 389 32 796 1 3

4 390 32 668 1 2

4 391 16 1117 1 4

4 392 8 1089 1 1

4 393 32 1098 1 1

4 394 32 764 1 3

4 395 16 701 1 1

4 396 16 1477 1 1

4 397 32 1116 1 1

4 398 8 864 1 2

4 399 8 861 1 5

4 400 16 925 1 3

4 401 8 947 1 2

4 402 16 1342 1 4

4 403 16 1198 1 1

4 404 8 822 1 3

4 405 8 619 1 4

4 406 16 1187 1 1

4 407 8 962 1 5

4 408 8 973 1 4

4 409 32 699 1 4

4 410 8 920 1 1

4 411 32 1045 1 3

4 412 8 759 1 4

4 413 8 626 1 3

4 414 16 785 1 5

4 415 16 744 1 5

4 416 16 1424 1 4

4 417 16 1009 1 4

4 418 32 1130 1 5

4 419 32 1000 1 5

4 420 16 897 1 5

4 421 16 985 1 4

4 422 8 830 1 5

4 423 16 1364 1 2

4 424 16 586 1 5

4 425 16 1391 1 1

4 426 32 723 1 2

4 427 32 791 1 4

4 428 32 1000 1 2

4 429 8 1250 1 5

4 430 8 974 1 2

4 431 32 989 1 4

4 432 32 874 1 2

4 433 8 858 1 4

4 434 32 914 1 3

4 435 32 949 1 4

4 436 8 880 1 1

4 437 32 950 1 3

4 438 8 1263 1 1

4 439 8 926 1 2

4 440 8 967 1 1

4 441 16 1047 1 1

4 442 16 771 1 4

4 443 32 875 1 5

4 444 16 743 0 4

4 445 32 1224 1 4

4 446 16 1261 1 2

4 447 16 756 1 5

4 448 16 1046 1 2

4 449 8 774 1 1

4 450 32 660 1 4

4 451 32 555 1 5

4 452 8 861 1 4

4 453 16 574 1 3

4 454 16 792 1 2

4 455 32 769 1 1

4 456 32 774 1 3

4 457 8 694 1 4

4 458 16 1051 1 1

4 459 32 879 1 1

4 460 16 961 0 3

4 461 8 1080 1 1

4 462 16 814 1 3

4 463 32 668 1 2

4 464 32 648 1 4

4 465 8 750 1 3

4 466 8 915 1 5

4 467 32 883 1 4

4 468 8 746 1 3

4 469 8 858 1 5

4 470 8 692 0 2

4 471 8 789 1 4

4 472 16 970 0 2

4 473 8 938 1 2

4 474 32 877 1 5

4 475 16 1332 1 2

4 476 16 1276 0 2

4 477 8 931 1 3

4 478 16 1035 1 1

4 479 32 842 1 1

4 480 8 713 1 3

4 481 8 1020 0 2

4 482 8 711 1 3

4 483 8 612 1 5

4 484 32 837 1 2

4 485 8 1073 1 1

4 486 8 774 1 5

4 487 16 798 1 4

4 488 8 573 1 5

4 489 16 949 0 2

4 490 32 787 1 3

4 491 8 682 0 5

4 492 32 778 1 3

4 493 16 940 1 3

4 494 16 745 1 5

4 495 16 863 1 5

4 496 32 831 1 3

4 497 32 822 1 5

4 498 32 726 1 2

4 499 32 838 1 2

4 500 8 879 1 4

4 501 16 593 1 3

4 502 16 838 1 4

4 503 8 902 1 3

4 504 32 857 1 1

4 505 8 872 1 3

4 506 16 1018 0 2

4 507 8 816 1 4

4 508 8 743 1 4

4 509 32 774 1 4

4 510 16 2455 1 1

4 511 8 719 1 2

4 512 16 1593 1 1

4 513 8 868 1 4

4 514 8 743 0 2

4 515 8 1009 1 3

4 516 16 1215 1 2

4 517 32 626 1 4

4 518 8 1234 1 1

4 519 8 1160 1 2

4 520 8 856 1 2

4 521 16 1060 1 3

4 522 32 662 1 4

4 523 16 624 1 5

4 524 32 788 1 2

4 525 16 976 1 4

4 526 16 919 1 3

4 527 16 1535 1 2

4 528 32 728 1 5

4 529 32 864 1 1

4 530 8 800 1 5

4 531 32 736 1 4

4 532 32 876 1 2

4 533 32 808 1 3

4 534 8 767 1 5

4 535 32 885 1 3

4 536 8 1201 1 2

4 537 32 1050 1 1

4 538 16 1497 1 2

4 539 32 1209 1 4

4 540 32 843 1 5

4 541 32 1280 1 4

4 542 32 1233 1 1

4 543 16 1067 1 2

4 544 8 699 1 4

4 545 16 1533 1 4

4 546 16 1091 1 3

4 547 16 866 1 5

4 548 32 1109 1 2

4 549 32 1066 1 1

4 550 16 899 1 1

4 551 32 1698 1 1

4 552 16 1064 1 1

4 553 16 945 1 3

4 554 32 916 1 3

4 555 32 845 1 2

4 556 8 902 1 2

4 557 32 1148 1 2

4 558 16 2008 1 1

4 559 16 856 1 5

4 560 8 918 1 1

4 561 16 1090 1 4

4 562 8 903 1 2

4 563 32 1290 1 3

4 564 16 1116 1 4

4 565 32 1228 1 5

4 566 8 1006 1 1

4 567 16 1197 1 1

4 568 16 838 1 2

4 569 32 881 1 5

4 570 8 844 1 3

4 571 16 895 1 4

4 572 8 710 1 3

4 573 8 946 1 1

4 574 8 835 1 3

4 575 32 928 1 5

4 576 16 815 1 5

4 577 32 957 1 1

4 578 16 709 1 4

4 579 32 1033 1 5

4 580 8 664 1 4

4 581 16 1108 0 2

4 582 16 894 1 3

4 583 8 1745 1 1

4 584 16 746 1 5

4 585 32 1264 1 4

4 586 8 1707 1 1

4 587 16 1231 1 1

4 588 8 1030 1 3

4 589 8 830 1 4

4 590 16 738 1 5

4 591 8 839 1 4

4 592 32 996 1 5

4 593 32 693 1 4

4 594 16 1511 1 1

4 595 32 1022 1 3

4 596 8 974 1 1

4 597 8 842 1 5

4 598 32 923 1 1

4 599 8 786 1 5

4 600 16 939 0 3

4 601 8 791 1 5

4 602 32 725 1 3

4 603 16 1002 1 2

4 604 16 645 1 5

4 605 16 862 1 3

4 606 8 596 1 3

4 607 16 744 0 1

4 608 8 563 1 4

4 609 32 790 1 2

4 610 32 648 0 4

4 611 8 806 1 3

4 612 16 139 1 2

4 613 32 688 0 4

4 614 32 954 1 1

4 615 8 1001 1 4

4 616 16 796 1 3

4 617 8 659 1 5

4 618 16 1248 1 4

4 619 8 827 1 5

4 620 32 843 1 1

4 621 16 1030 1 3

4 622 16 966 1 1

4 623 8 772 1 2

4 624 8 784 1 5

4 625 16 864 1 4

4 626 8 1166 1 1

4 627 8 837 1 2

4 628 32 791 1 2

4 629 8 759 1 5

4 630 8 686 1 3

4 631 16 1009 1 1

4 632 8 806 0 1

4 633 32 823 1 2

4 634 16 1197 1 1

4 635 16 774 1 5

4 636 32 849 1 5

4 637 32 652 1 4

4 638 32 798 1 5

4 639 16 990 0 1

4 640 32 681 1 4

4 641 32 1054 1 5

4 642 8 695 1 3

4 643 16 757 1 4

4 644 8 871 1 2

4 645 8 768 1 4

4 646 32 750 1 2

4 647 8 880 1 2

4 648 32 862 1 2

4 649 32 694 1 2

4 650 8 918 1 1

4 651 8 864 1 2

4 652 32 647 1 1

4 653 32 584 1 3

4 654 32 759 1 2

4 655 16 642 1 2

4 656 16 677 1 5

4 657 32 591 1 1

4 658 8 634 1 4

4 659 32 764 1 3

4 660 16 808 1 5

4 661 16 857 1 3

4 662 16 1186 1 4

4 663 32 729 1 2

4 664 16 958 1 2

4 665 8 1382 1 1

4 666 16 935 1 1

4 667 16 667 1 4

4 668 8 890 1 4

4 669 16 670 1 4

4 670 32 1122 1 4

4 671 8 834 1 2

4 672 16 675 1 5

4 673 32 1392 1 1

4 674 16 1026 1 3

4 675 32 791 1 5

4 676 32 660 1 3

4 677 8 636 1 4

4 678 8 711 1 2

4 679 16 592 1 3

4 680 8 705 1 4

4 681 8 813 1 2

4 682 8 688 1 3

4 683 16 692 1 5

4 684 8 683 1 3

4 685 16 574 1 2

4 686 16 685 0 1

4 687 32 778 1 5

4 688 8 642 1 1

4 689 16 1159 1 3

4 690 32 875 1 1

4 691 16 684 1 5

4 692 32 702 1 4

4 693 8 925 1 4

4 694 8 768 1 5

4 695 16 1072 0 2

4 696 32 764 1 3

4 697 8 714 1 3

4 698 32 819 1 3

4 699 8 626 1 5

4 700 16 1079 1 2

4 701 16 1042 1 4

4 702 16 1253 1 4

4 703 16 916 1 2

4 704 32 564 1 3

4 705 32 789 1 4

4 706 32 609 0 3

4 707 8 656 1 3

4 708 8 677 1 5

4 709 8 682 1 1

4 710 16 618 1 3

4 711 16 688 1 5

4 712 32 707 1 4

4 713 32 715 1 5

4 714 32 985 0 1

4 715 32 790 1 5

4 716 32 1226 1 1

4 717 8 958 0 1

4 718 8 981 1 1

4 719 32 716 1 5

4 720 16 1149 1 1

4 721 32 714 1 5

4 722 32 991 1 5

4 723 16 788 1 2

4 724 32 1048 1 1

4 725 8 799 1 4

4 726 32 909 1 5

4 727 16 1478 1 1

4 728 32 693 0 4

4 729 32 840 1 1

4 730 8 871 1 1

4 731 32 597 1 2

4 732 32 595 1 3

4 733 16 680 1 3

4 734 16 737 1 4

4 735 32 962 1 2

4 736 32 723 1 1

4 737 8 707 1 5

4 738 8 726 1 2

4 739 16 583 1 5

4 740 16 849 1 4

4 741 32 754 1 4

4 742 32 759 1 3

4 743 8 844 1 4

4 744 8 614 1 5

4 745 16 592 1 5

4 746 8 1085 1 1

4 747 16 741 0 4

4 748 16 1297 1 1

4 749 8 707 1 5

4 750 32 524 1 2

4 751 32 803 1 1

4 752 32 698 1 2

4 753 32 596 1 2

4 754 8 841 1 2

4 755 8 627 1 4

4 756 16 1037 1 3

4 757 32 1219 1 5

4 758 16 1023 1 1

4 759 8 743 1 5

4 760 16 609 1 5

4 761 32 784 1 4

4 762 16 1202 1 1

4 763 16 1041 1 4

4 764 32 857 1 5

4 765 16 761 1 2

4 766 32 682 1 4

4 767 8 984 1 5

4 768 32 1186 1 4

4 769 8 764 1 4

4 770 8 925 1 4

4 771 8 1187 1 1

4 772 8 744 1 3

4 773 16 1066 1 1

4 774 16 867 1 3

4 775 32 879 1 5

4 776 16 700 1 2

4 777 32 757 1 3

4 778 32 700 1 3

4 779 8 816 1 1

4 780 32 736 1 2

4 781 32 1846 1 2

4 782 8 615 1 4

4 783 16 1027 1 5

4 784 16 1115 1 1

4 785 16 1080 1 4

4 786 32 689 1 4

4 787 16 976 1 3

4 788 8 953 1 1

4 789 8 662 1 5

4 790 8 563 1 3

4 791 16 931 1 3

4 792 16 858 1 2

4 793 8 844 1 5

4 794 16 828 1 1

4 795 8 649 1 4

4 796 32 802 1 4

4 797 32 729 1 4

4 798 32 633 0 3

4 799 16 976 1 3

4 800 16 762 1 5

4 801 8 769 1 3

4 802 16 1086 1 3

4 803 8 878 1 2

4 804 32 701 1 3

4 805 8 719 1 4

4 806 8 771 1 2

4 807 16 658 1 3

4 808 32 702 1 1

4 809 16 1764 0 2

4 810 16 870 1 1

4 811 8 724 1 2

4 812 16 836 1 4

4 813 16 1064 1 2

4 814 8 1062 0 1

4 815 8 922 1 3

4 816 32 1000 1 3

4 817 16 600 1 5

4 818 32 839 1 1

4 819 32 859 1 1

4 820 8 717 1 1

4 821 16 659 1 5

4 822 8 822 1 2

4 823 16 903 1 4

4 824 8 833 1 5

4 825 16 575 1 5

4 826 32 1214 1 1

4 827 8 705 1 3

4 828 32 638 1 3

4 829 8 541 1 3

4 830 16 568 0 2

4 831 8 708 1 2

4 832 8 641 0 1

4 833 8 797 1 2

4 834 32 731 1 2

4 835 16 834 1 2

4 836 16 944 1 4

4 837 8 833 1 3

4 838 32 817 1 5

4 839 32 603 1 5

4 840 8 1155 1 3

4 841 32 696 1 4

4 842 8 1047 1 2

4 843 8 863 1 2

4 844 8 857 1 1

4 845 8 789 1 5

4 846 8 714 1 2

4 847 16 1070 0 2

4 848 32 861 1 3

4 849 32 713 1 3

4 850 32 853 1 5

4 851 16 1149 1 1

4 852 8 602 1 4

4 853 16 1114 1 1

4 854 16 814 1 3

4 855 8 689 1 5

4 856 16 1134 1 2

4 857 16 1070 1 4

4 858 8 725 1 5

4 859 16 796 1 4

4 860 16 1002 1 1

4 861 32 689 1 5

4 862 8 714 1 1

4 863 16 608 1 5

4 864 16 681 1 4

4 865 16 713 0 1

4 866 8 768 1 4

4 867 32 691 1 2

4 868 16 753 1 3

4 869 8 1099 1 1

4 870 16 693 1 2

4 871 16 1031 0 2

4 872 8 1331 1 2

4 873 32 1208 1 5

4 874 32 865 1 1

4 875 16 610 1 5

4 876 32 1129 1 2

4 877 32 617 1 2

4 878 32 692 1 3

4 879 8 590 1 4

4 880 32 874 1 4

4 881 32 685 1 4

4 882 32 535 1 2

4 883 16 769 1 3

4 884 16 1159 1 4

4 885 8 956 1 1

4 886 8 612 1 4

4 887 16 699 1 3

4 888 32 694 1 5

4 889 32 865 1 1

4 890 8 793 1 4

4 891 8 760 1 2

4 892 8 720 1 5

4 893 8 521 1 3

4 894 32 724 1 2

4 895 16 1209 1 1

4 896 32 872 1 5

4 897 8 908 1 2

4 898 32 538 0 4

4 899 8 1125 1 1

4 900 16 1403 0 2

4 901 8 994 1 3

4 902 16 1110 1 1

4 903 16 1020 0 2

4 904 8 1212 0 1

4 905 8 786 1 1

4 906 32 1259 1 1

4 907 32 688 1 1

4 908 32 1135 1 5

4 909 8 695 1 2

4 910 8 754 1 5

4 911 16 777 0 4

4 912 16 1309 1 2

4 913 16 657 1 3

4 914 32 757 1 1

4 915 8 707 1 4

4 916 8 783 1 4

4 917 16 875 1 4

4 918 16 1063 1 3

4 919 8 729 1 3

4 920 32 688 1 3

4 921 32 837 1 4

4 922 16 620 1 5

4 923 32 893 1 4

4 924 32 824 1 2

4 925 16 1088 1 2

4 926 8 704 1 5

4 927 16 699 1 5

4 928 32 702 1 2

4 929 32 705 1 4

4 930 16 836 1 3

4 931 8 690 1 3

4 932 8 739 1 5

4 933 32 692 1 1

4 934 32 639 1 3

4 935 16 590 1 5

4 936 16 868 1 4

4 937 16 1080 1 1

4 938 32 681 1 3

4 939 32 623 1 2

4 940 8 601 1 3

4 941 8 689 1 3

4 942 32 881 1 5

4 943 32 728 1 3

4 944 16 800 1 3

4 945 32 761 1 4

4 946 16 666 1 5

4 947 16 750 1 5

4 948 32 611 1 3

4 949 8 731 1 2

4 950 32 1025 1 1

4 951 32 732 1 5

4 952 8 589 1 3

4 953 16 617 1 5

4 954 16 899 1 4

4 955 8 1105 1 4

4 956 8 681 1 5

4 957 8 832 1 3

4 958 8 718 1 1

4 959 16 1359 1 1

4 960 32 704 1 1

4 961 32 732 1 4

4 962 16 735 1 3

4 963 32 594 1 2

4 964 8 625 1 4

4 965 8 571 1 2

4 966 8 550 1 3

4 967 8 722 1 2

4 968 32 652 0 3

4 969 16 653 1 2

4 970 8 735 1 5

4 971 16 1375 1 2

4 972 32 713 1 4

4 973 32 917 1 5

4 974 32 1311 1 1

4 975 32 836 1 1

4 976 16 760 1 5

4 977 32 808 0 3

4 978 16 899 0 4

4 979 8 618 1 5

4 980 8 669 1 5

4 981 32 802 1 5

4 982 8 674 1 3

4 983 16 603 1 5

4 984 8 595 1 5

4 985 16 1634 1 1

4 986 32 758 1 5

4 987 32 745 1 4

4 988 16 907 1 3

4 989 32 733 1 3

4 990 32 669 1 4

4 991 16 750 1 2

4 992 8 658 1 4

4 993 32 715 1 3

4 994 8 781 1 2

4 995 16 765 1 2

4 996 16 1031 0 2

4 997 8 1192 0 1

4 998 32 1011 1 4

4 999 8 743 1 3

4 1000 16 1293 1 3

4 1001 32 685 1 4

4 1002 8 680 1 4

4 1003 16 582 0 2

4 1004 32 677 1 4

4 1005 32 768 1 4

4 1006 8 817 0 1

4 1007 16 576 1 5

4 1008 32 717 1 2

4 1009 8 646 1 2

4 1010 8 611 0 3

4 1011 32 753 1 1

4 1012 16 1419 1 4

4 1013 32 893 1 1

4 1014 16 605 1 5

4 1015 32 626 1 3

4 1016 8 860 1 2

4 1017 8 780 1 3

4 1018 32 1078 1 2

4 1019 16 675 1 5

4 1020 8 919 1 4

4 1021 8 578 1 3

4 1022 16 531 1 3

4 1023 32 827 1 5

4 1024 32 694 1 3

4 1025 16 1667 1 4

4 1026 16 1389 1 1

4 1027 32 748 1 2

4 1028 16 1081 1 2

4 1029 8 1156 1 1

4 1030 8 708 1 4

4 1031 8 676 1 4

4 1032 16 693 1 3

4 1033 32 660 1 1

4 1034 16 833 1 3

4 1035 16 1011 1 4

4 1036 16 1125 1 1

4 1037 32 837 1 5

4 1038 16 1135 1 1

4 1039 16 1152 1 1

4 1040 32 954 1 2

4 1041 8 657 1 4

4 1042 16 887 1 1

4 1043 16 984 1 1

4 1044 8 728 1 3

4 1045 32 667 1 2

4 1046 8 678 1 2

4 1047 8 599 1 5

4 1048 32 642 0 3

4 1049 16 1110 1 4

4 1050 8 1033 1 5

4 1051 16 737 1 4

4 1052 8 795 1 3

4 1053 8 1246 1 1

4 1054 32 634 1 2

4 1055 32 628 1 1

4 1056 16 735 1 3

4 1057 16 733 1 3

4 1058 16 868 1 4

4 1059 8 822 1 1

4 1060 16 710 1 2

4 1061 8 625 1 2

4 1062 16 840 1 1

4 1063 16 737 1 5

4 1064 32 776 1 3

4 1065 32 774 1 5

4 1066 8 948 1 2

4 1067 8 1483 1 5

4 1068 8 672 0 1

4 1069 8 956 0 1

4 1070 32 900 1 5

4 1071 8 677 1 1

4 1072 8 718 1 4

4 1073 32 1078 1 5

4 1074 32 1026 1 2

4 1075 32 709 1 1

4 1076 32 606 1 1

4 1077 16 850 1 5

4 1078 16 764 1 5

4 1079 8 757 1 5

4 1080 16 829 1 4

4 1081 8 608 1 5

4 1082 16 1350 1 1

4 1083 8 583 0 2

4 1084 8 856 1 1

4 1085 16 572 1 5

4 1086 8 996 1 1

4 1087 32 748 1 3

4 1088 16 723 1 5

4 1089 16 781 1 2

4 1090 32 1126 1 5

4 1091 16 1187 1 2

4 1092 32 778 0 5

4 1093 32 750 1 1

4 1094 16 767 1 3

4 1095 8 799 1 4

4 1096 8 790 1 4

4 1097 32 724 0 4

4 1098 16 826 1 2

4 1099 8 728 1 1

4 1100 8 705 1 5

4 1101 16 1054 1 4

4 1102 32 982 1 3

4 1103 32 935 1 4

4 1104 32 785 1 4

4 1105 32 1034 1 1

4 1106 32 867 1 1

4 1107 32 832 1 1

4 1108 32 614 1 2

4 1109 16 924 1 4

4 1110 32 766 1 1

4 1111 16 673 1 5

4 1112 16 894 1 2

4 1113 16 979 0 1

4 1114 16 862 1 3

4 1115 16 882 1 3

4 1116 32 699 1 3

4 1117 16 775 1 3

4 1118 16 1089 1 4

4 1119 8 970 1 1

4 1120 8 750 1 2

4 1121 32 933 1 3

4 1122 32 865 1 2

4 1123 32 870 1 5

4 1124 16 1210 0 2

4 1125 16 695 1 3

4 1126 8 1328 1 1

4 1127 8 778 1 3

4 1128 16 1092 1 1

4 1129 8 813 1 1

4 1130 8 740 1 2

4 1131 8 734 1 5

4 1132 16 700 1 5

4 1133 8 780 1 2

4 1134 32 1207 1 2

4 1135 16 1075 1 2

4 1136 32 731 1 5

4 1137 32 764 1 1

4 1138 16 621 1 5

4 1139 32 834 1 4

4 1140 32 776 1 3

4 1141 32 1007 1 1

4 1142 8 1151 1 1

4 1143 8 717 1 3

4 1144 8 659 1 5

4 1145 16 854 1 4

4 1146 8 884 1 4

4 1147 16 1014 1 2

4 1148 32 722 1 4

4 1149 16 757 1 1

4 1150 32 724 1 5

4 1151 8 647 1 3

4 1152 32 576 1 2

4 1153 8 591 1 2

4 1154 8 669 1 4

4 1155 16 998 0 3

4 1156 32 696 1 5

4 1157 8 915 1 2

4 1158 8 649 1 5

4 1159 16 845 1 4

4 1160 32 966 1 5

4 1161 16 1413 1 1

4 1162 16 1100 1 4

4 1163 16 649 1 5

4 1164 8 756 1 3

4 1165 32 731 1 2

4 1166 32 788 1 3

4 1167 8 784 1 3

4 1168 32 700 1 4

4 1169 16 807 1 1

4 1170 16 737 1 3

4 1171 32 787 1 4

4 1172 32 611 1 3

4 1173 16 1171 1 1

4 1174 16 1178 1 4

4 1175 8 607 1 3

4 1176 32 729 1 1

4 1177 16 1065 0 1

4 1178 8 593 1 5

4 1179 32 706 1 3

4 1180 16 1330 1 4

4 1181 8 676 1 2

4 1182 8 650 1 3

4 1183 32 697 1 4

4 1184 8 718 1 5

4 1185 8 728 1 4

4 1186 16 1150 1 2

4 1187 8 709 1 3

4 1188 32 796 0 2

4 1189 16 719 1 3

4 1190 8 837 0 1

4 1191 8 711 1 5

4 1192 32 750 1 2

4 1193 16 606 1 5

4 1194 8 708 1 2

4 1195 16 589 1 5

4 1196 32 884 1 5

4 1197 8 740 1 4

4 1198 8 680 1 4

4 1199 32 686 1 2

4 1200 8 879 1 4

5 1 16 1491 1 4

5 2 16 826 1 3

5 3 8 892 0 1

5 4 8 739 0 1

5 5 16 1060 1 3

5 6 8 695 1 3

5 7 8 719 1 2

5 8 32 993 1 1

5 9 8 906 1 4

5 10 32 659 1 3

5 11 16 902 1 2

5 12 32 1139 1 5

5 13 32 978 1 5

5 14 32 743 1 1

5 15 8 948 0 3

5 16 32 648 1 4

5 17 32 857 1 4

5 18 16 2335 0 3

5 19 32 953 1 2

5 20 32 944 1 1

5 21 8 839 1 2

5 22 16 1465 1 5

5 23 16 588 1 5

5 24 16 937 0 1

5 25 32 980 1 2

5 26 8 1041 1 3

5 27 32 1090 1 4

5 28 16 1088 1 1

5 29 8 883 1 4

5 30 16 997 1 4

5 31 8 715 0 5

5 32 16 1304 1 4

5 33 8 1676 1 1

5 34 16 882 1 2

5 35 8 846 1 5

5 36 8 1026 1 2

5 37 8 847 1 1

5 38 8 688 1 5

5 39 32 807 1 5

5 40 32 725 0 1

5 41 32 1764 1 4

5 42 8 676 1 5

5 43 8 813 0 1

5 44 16 935 1 5

5 45 8 805 1 1

5 46 32 710 1 3

5 47 8 751 1 2

5 48 32 939 0 1

5 49 16 829 1 5

5 50 16 835 0 1

5 51 16 898 1 5

5 52 16 837 0 4

5 53 8 1048 1 3

5 54 32 711 1 4

5 55 16 1241 0 1

5 56 8 821 1 3

5 57 16 1315 1 3

5 58 32 781 1 3

5 59 32 958 1 5

5 60 32 842 1 4

5 61 32 1039 1 1

5 62 8 978 1 4

5 63 32 1029 1 2

5 64 8 824 1 3

5 65 16 1289 0 1

5 66 16 613 1 1

5 67 16 781 1 3

5 68 32 713 1 4

5 69 16 854 0 2

5 70 8 672 1 3

5 71 8 1516 1 4

5 72 32 963 1 4

5 73 8 671 1 4

5 74 32 949 1 1

5 75 16 777 1 5

5 76 32 750 0 5

5 77 8 915 1 2

5 78 32 1233 1 2

5 79 32 1149 1 2

5 80 16 1126 1 2

5 81 8 687 1 3

5 82 16 1014 1 4

5 83 32 878 1 5

5 84 32 848 1 3

5 85 32 668 1 3

5 86 8 1510 1 2

5 87 32 626 1 2

5 88 16 2085 1 3

5 89 16 1765 1 1

5 90 16 942 1 4

5 91 16 1497 1 3

5 92 16 1186 1 5

5 93 16 1287 1 4

5 94 8 1194 1 2

5 95 16 925 0 2

5 96 16 875 0 3

5 97 32 959 1 5

5 98 8 1104 1 4

5 99 32 1139 1 1

5 100 32 837 1 3

5 101 32 548 1 2

5 102 32 945 1 2

5 103 32 910 1 3

5 104 16 1390 1 5

5 105 16 1905 1 4

5 106 16 1174 1 1

5 107 8 642 1 4

5 108 8 704 0 1

5 109 8 1295 1 2

5 110 8 616 1 5

5 111 32 719 1 3

5 112 32 928 1 5

5 113 16 684 1 2

5 114 16 1918 1 2

5 115 16 501 1 2

5 116 8 715 1 5

5 117 8 553 1 5

5 118 8 545 1 4

5 119 8 691 1 1

5 120 8 739 1 5

5 121 16 530 0 4

5 122 8 756 1 5

5 123 32 974 1 4

5 124 8 488 0 4

5 125 16 682 1 1

5 126 16 579 0 2

5 127 8 614 1 3

5 128 32 974 1 3

5 129 8 1104 1 4

5 130 32 776 1 1

5 131 8 1239 1 5

5 132 32 1121 1 1

5 133 16 1643 1 1

5 134 32 922 1 1

5 135 32 841 1 2

5 136 32 1930 1 1

5 137 16 1594 1 2

5 138 32 927 1 5

5 139 8 973 1 1

5 140 16 823 1 2

5 141 16 888 1 4

5 142 16 655 0 3

5 143 32 517 0 2

5 144 32 1113 1 4

5 145 32 740 1 1

5 146 32 1035 1 5

5 147 32 902 1 4

5 148 8 1008 1 1

5 149 16 1061 1 4

5 150 8 735 1 5

5 151 16 1222 1 3

5 152 32 895 1 5

5 153 8 674 0 4

5 154 8 681 1 2

5 155 32 816 0 1

5 156 32 1169 1 2

5 157 32 1095 0 2

5 158 32 1129 1 4

5 159 8 966 1 1

5 160 8 1458 0 1

5 161 32 1811 1 3

5 162 8 889 1 2

5 163 16 1000 1 5

5 164 16 1084 0 1

5 165 16 1277 1 4

5 166 32 1071 1 2

5 167 32 1160 1 5

5 168 32 840 1 3

5 169 16 1428 0 2

5 170 8 1638 1 1

5 171 32 850 1 5

5 172 32 1032 1 5

5 173 8 954 1 4

5 174 8 823 1 3

5 175 16 1349 1 3

5 176 32 1016 1 5

5 177 16 1565 0 1

5 178 16 698 1 5

5 179 16 873 0 3

5 180 8 1102 1 3

5 181 8 767 0 3

5 182 16 896 1 3

5 183 8 1095 1 2

5 184 16 767 1 5

5 185 32 1254 1 1

5 186 8 935 1 4

5 187 8 1269 1 3

5 188 16 1056 1 3

5 189 32 1110 1 1

5 190 8 653 1 5

5 191 16 783 1 5

5 192 16 1206 1 4

5 193 8 806 1 2

5 194 8 715 0 4

5 195 16 1440 1 5

5 196 8 997 1 2

5 197 32 746 1 3

5 198 8 838 1 4

5 199 8 2482 1 3

5 200 32 1094 1 4

5 201 8 843 1 4

5 202 16 1242 1 5

5 203 16 868 1 3

5 204 32 689 1 4

5 205 16 815 1 4

5 206 32 834 1 4

5 207 16 1162 1 1

5 208 8 675 1 2

5 209 8 807 1 5

5 210 16 1098 1 2

5 211 32 930 1 2

5 212 16 1032 1 2

5 213 8 1704 1 5

5 214 8 808 1 3

5 215 16 822 1 5

5 216 16 778 1 3

5 217 16 722 1 5

5 218 8 675 1 5

5 219 16 656 1 4

5 220 32 1144 1 5

5 221 32 833 1 2

5 222 16 844 0 2

5 223 16 805 1 2

5 224 32 804 1 3

5 225 32 875 1 2

5 226 16 1347 1 1

5 227 32 656 0 3

5 228 8 748 1 5

5 229 32 1015 1 3

5 230 8 871 1 3

5 231 32 789 1 4

5 232 16 751 0 4

5 233 16 2372 0 1

5 234 8 1187 1 1

5 235 32 924 1 3

5 236 8 715 1 2

5 237 8 1100 1 1

5 238 8 946 1 2

5 239 16 906 1 1

5 240 8 1024 1 1

5 241 32 696 1 4

5 242 32 755 1 2

5 243 8 593 0 2

5 244 8 873 1 2

5 245 8 1034 1 1

5 246 32 933 1 5

5 247 32 800 1 5

5 248 16 1339 1 5

5 249 8 1209 1 1

5 250 8 871 1 1

5 251 8 622 0 5

5 252 16 992 1 5

5 253 8 860 1 3

5 254 32 878 1 2

5 255 16 1406 1 1

5 256 32 1091 1 3

5 257 8 746 1 1

5 258 16 686 1 5

5 259 8 1164 1 4

5 260 8 697 1 4

5 261 8 2068 1 3

5 262 16 1055 1 2

5 263 16 1056 1 3

5 264 8 985 1 4

5 265 16 1439 0 3

5 266 32 1133 1 3

5 267 32 694 1 3

5 268 8 791 1 2

5 269 8 827 1 3

5 270 32 808 1 3

5 271 32 832 1 5

5 272 16 765 1 3

5 273 8 891 0 4

5 274 8 717 1 3

5 275 8 825 1 2

5 276 16 1758 0 4

5 277 32 956 1 2

5 278 32 1022 0 5

5 279 16 2472 1 2

5 280 16 932 1 5

5 281 32 1065 1 1

5 282 32 953 1 4

5 283 16 1378 1 2

5 284 32 780 1 4

5 285 16 973 0 2

5 286 32 799 1 2

5 287 16 1068 1 4

5 288 32 928 1 3

5 289 16 1291 1 4

5 290 32 882 1 5

5 291 16 1525 1 1

5 292 8 628 1 3

5 293 32 927 1 1

5 294 16 941 0 3

5 295 32 1255 1 2

5 296 8 689 1 4

5 297 8 831 1 5

5 298 16 922 0 1

5 299 16 1688 1 1

5 300 32 836 1 3

5 301 32 782 1 4

5 302 32 799 1 1

5 303 16 1164 1 5

5 304 32 906 1 1

5 305 16 733 1 5

5 306 32 726 1 4

5 307 32 774 1 3

5 308 16 784 1 3

5 309 16 944 1 2

5 310 32 1373 1 5

5 311 16 941 0 2

5 312 32 767 1 4

5 313 32 996 1 2

5 314 8 760 1 5

5 315 16 1110 1 2

5 316 8 1016 1 1

5 317 16 889 0 5

5 318 16 1104 1 4

5 319 8 898 1 2

5 320 16 1001 0 1

5 321 8 819 1 4

5 322 32 910 1 1

5 323 8 872 1 2

5 324 16 1202 1 4

5 325 8 1055 1 3

5 326 32 727 1 4

5 327 32 806 1 2

5 328 32 854 1 2

5 329 8 653 1 5

5 330 8 883 1 3

5 331 8 1388 1 1

5 332 8 765 1 4

5 333 32 799 1 1

5 334 16 755 1 3

5 335 8 864 1 5

5 336 32 1164 1 5

5 337 8 694 0 1

5 338 8 770 1 2

5 339 16 960 1 3

5 340 32 976 1 1

5 341 16 881 1 3

5 342 16 856 1 1

5 343 32 684 1 4

5 344 16 579 1 5

5 345 8 537 1 5

5 346 8 577 1 4

5 347 8 839 1 1

5 348 32 667 1 3

5 349 8 633 0 5

5 350 16 866 0 4

5 351 16 1051 1 1

5 352 16 1059 0 4

5 353 16 1390 1 1

5 354 16 1020 1 4

5 355 32 1476 1 1

5 356 8 968 1 5

5 357 32 989 1 5

5 358 16 1105 0 2

5 359 8 1008 1 2

5 360 8 1060 1 3

5 361 8 968 1 3

5 362 16 1547 1 2

5 363 32 641 1 4

5 364 16 824 1 5

5 365 32 770 1 1

5 366 16 697 1 5

5 367 32 836 1 1

5 368 16 584 1 5

5 369 16 854 1 4

5 370 16 753 1 5

5 371 8 684 1 5

5 372 32 675 1 4

5 373 32 684 1 2

5 374 8 954 1 2

5 375 32 850 1 1

5 376 8 548 0 1

5 377 16 1310 0 2

5 378 16 723 1 2

5 379 32 860 1 1

5 380 8 704 1 5

5 381 32 966 1 2

5 382 16 939 1 2

5 383 8 584 1 4

5 384 16 642 1 5

5 385 32 827 1 1

5 386 16 1042 1 4

5 387 16 970 0 1

5 388 16 903 1 1

5 389 32 1055 1 5

5 390 32 844 1 2

5 391 16 1409 0 2

5 392 16 750 1 4

5 393 16 1448 1 4

5 394 16 1326 1 3

5 395 8 732 1 2

5 396 8 734 1 4

5 397 32 826 1 2

5 398 8 965 1 1

5 399 32 831 1 2

5 400 32 731 1 2

5 401 8 722 1 1

5 402 8 578 1 5

5 403 32 717 1 5

5 404 8 709 1 2

5 405 32 751 1 2

5 406 16 747 1 3

5 407 8 609 1 3

5 408 32 955 1 3

5 409 32 701 1 1

5 410 8 721 1 5

5 411 16 953 1 4

5 412 32 587 1 2

5 413 16 796 1 2

5 414 16 604 1 5

5 415 8 706 1 5

5 416 32 1015 1 4

5 417 32 822 1 3

5 418 32 1150 1 5

5 419 16 898 1 1

5 420 32 826 1 4

5 421 8 603 1 5

5 422 16 785 1 1

5 423 16 1195 1 4

5 424 8 676 1 1

5 425 32 685 1 3

5 426 8 624 1 4

5 427 16 631 1 3

5 428 32 567 1 5

5 429 16 559 1 5

5 430 16 715 1 3

5 431 32 687 1 5

5 432 8 803 1 2

5 433 16 590 1 2

5 434 8 672 0 1

5 435 8 778 1 3

5 436 8 820 0 1

5 437 16 840 1 4

5 438 32 796 1 3

5 439 8 560 1 2

5 440 8 1114 1 2

5 441 16 631 1 3

5 442 16 755 1 3

5 443 8 606 1 3

5 444 32 822 1 5

5 445 32 701 1 3

5 446 8 1188 1 4

5 447 32 582 0 4

5 448 8 575 0 2

5 449 16 824 1 4

5 450 32 886 1 4

5 451 16 813 1 1

5 452 8 1061 1 3

5 453 8 577 1 4

5 454 16 813 1 2

5 455 32 785 1 4

5 456 8 864 1 3

5 457 8 533 1 4

5 458 8 635 1 3

5 459 8 544 1 1

5 460 32 950 1 3

5 461 16 1357 1 3

5 462 8 576 1 3

5 463 32 504 0 3

5 464 8 874 1 5

5 465 16 794 1 5

5 466 32 806 1 5

5 467 8 621 1 5

5 468 32 891 1 4

5 469 32 746 1 5

5 470 8 702 1 1

5 471 16 974 1 1

5 472 16 755 0 1

5 473 32 859 1 1

5 474 8 563 0 2

5 475 32 731 1 1

5 476 16 1155 0 1

5 477 16 734 1 3

5 478 8 578 0 4

5 479 8 1032 1 4

5 480 32 821 0 3

5 481 8 999 1 2

5 482 8 666 1 5

5 483 8 575 1 3

5 484 32 1072 1 4

5 485 16 1234 1 1

5 486 32 677 1 3

5 487 16 761 0 4

5 488 8 882 0 5

5 489 8 945 1 4

5 490 32 905 1 5

5 491 32 892 1 1

5 492 8 952 1 1

5 493 16 788 1 3

5 494 16 617 1 5

5 495 16 954 0 2

5 496 32 728 1 3

5 497 16 1324 0 2

5 498 8 1052 1 1

5 499 8 794 1 1

5 500 32 941 1 1

5 501 8 734 1 3

5 502 32 694 1 1

5 503 16 832 1 2

5 504 8 635 1 2

5 505 16 872 1 4

5 506 16 755 1 3

5 507 8 822 1 5

5 508 32 864 1 1

5 509 8 522 1 3

5 510 32 640 1 3

5 511 8 536 1 5

5 512 8 643 0 2

5 513 32 789 1 3

5 514 8 824 1 1

5 515 16 1064 1 1

5 516 32 713 1 4

5 517 8 861 1 3

5 518 16 813 1 1

5 519 16 669 1 3

5 520 32 1034 1 2

5 521 16 1281 1 4

5 522 32 766 1 1

5 523 32 850 1 2

5 524 32 737 1 4

5 525 16 1146 1 2

5 526 16 777 1 3

5 527 8 636 1 4

5 528 8 830 1 2

5 529 32 716 1 1

5 530 16 823 1 2

5 531 8 554 0 4

5 532 16 991 1 4

5 533 8 674 1 5

5 534 8 616 1 5

5 535 16 1001 1 5

5 536 8 583 1 3

5 537 32 794 1 5

5 538 32 533 0 4

5 539 16 1175 1 4

5 540 8 665 1 4

5 541 16 1138 1 2

5 542 16 659 1 3

5 543 8 766 1 2

5 544 32 941 0 2

5 545 32 1054 1 2

5 546 16 983 1 3

5 547 8 1010 1 4

5 548 32 934 1 3

5 549 8 710 1 5

5 550 8 811 1 1

5 551 16 1055 0 1

5 552 32 740 1 4

5 553 16 766 1 5

5 554 32 788 1 5

5 555 16 896 1 4

5 556 32 872 1 2

5 557 32 715 1 4

5 558 8 648 1 3

5 559 32 750 1 2

5 560 8 539 1 4

5 561 16 1133 1 3

5 562 32 956 1 2

5 563 8 1327 1 3

5 564 16 557 1 5

5 565 32 626 0 1

5 566 8 641 1 2

5 567 16 829 1 1

5 568 32 665 1 4

5 569 16 715 1 3

5 570 16 1045 1 4

5 571 16 1734 1 5

5 572 32 720 1 5

5 573 16 918 1 4

5 574 32 732 1 4

5 575 16 683 0 2

5 576 16 884 1 2

5 577 8 667 1 4

5 578 16 1164 0 1

5 579 32 775 1 3

5 580 8 657 0 1

5 581 32 1052 1 5

5 582 16 554 1 5

5 583 8 742 1 2

5 584 8 689 1 1

5 585 32 697 1 5

5 586 8 644 1 4

5 587 32 787 1 3

5 588 32 981 1 1

5 589 8 720 1 2

5 590 16 1323 1 1

5 591 32 731 1 3

5 592 16 683 1 5

5 593 8 546 1 3

5 594 8 648 0 5

5 595 16 1111 1 1

5 596 32 900 1 5

5 597 8 1069 1 1

5 598 16 776 1 5

5 599 32 905 1 2

5 600 32 777 1 5

5 601 8 856 1 3

5 602 16 892 1 1

5 603 16 683 1 3

5 604 32 714 1 1

5 605 16 655 1 5

5 606 16 1022 0 1

5 607 16 1049 1 4

5 608 8 1080 1 5

5 609 8 890 1 5

5 610 8 793 1 4

5 611 32 729 1 4

5 612 32 807 1 5

5 613 8 828 1 2

5 614 32 671 1 2

5 615 8 708 1 1

5 616 32 706 1 2

5 617 8 602 1 2

5 618 16 770 1 2

5 619 16 2885 1 1

5 620 16 549 1 5

5 621 16 661 1 2

5 622 16 783 1 5

5 623 16 681 1 3

5 624 8 1634 1 4

5 625 8 712 0 5

5 626 16 1052 0 1

5 627 16 616 1 5

5 628 8 601 1 5

5 629 32 818 1 3

5 630 32 719 1 4

5 631 32 718 1 5

5 632 8 596 1 5

5 633 16 775 1 3

5 634 8 652 1 2

5 635 16 642 1 3

5 636 8 657 1 2

5 637 16 684 1 1

5 638 32 738 1 1

5 639 8 675 1 2

5 640 16 569 1 5

5 641 16 1019 1 4

5 642 32 723 1 5

5 643 16 1091 1 4

5 644 32 1101 1 5

5 645 16 584 1 3

5 646 32 682 1 2

5 647 8 1158 1 1

5 648 32 778 1 2

5 649 8 629 1 3

5 650 16 1000 1 4

5 651 8 742 0 1

5 652 8 921 1 3

5 653 32 593 1 1

5 654 8 1735 1 5

5 655 32 738 1 5

5 656 16 692 1 5

5 657 32 644 1 3

5 658 16 921 1 2

5 659 32 576 1 4

5 660 8 539 1 3

5 661 8 638 1 4

5 662 16 745 1 1

5 663 8 556 1 4

5 664 32 815 1 2

5 665 16 809 0 2

5 666 8 812 1 4

5 667 32 705 1 3

5 668 8 706 1 1

5 669 32 565 1 4

5 670 16 669 1 5

5 671 32 698 1 5

5 672 32 755 1 1

5 673 8 600 1 1

5 674 16 736 1 4

5 675 8 681 1 1

5 676 8 657 1 5

5 677 32 1358 1 3

5 678 32 754 1 4

5 679 8 545 1 3

5 680 32 809 1 2

5 681 8 2024 1 1

5 682 16 676 1 1

5 683 32 607 1 3

5 684 8 536 1 3

5 685 8 818 1 4

5 686 16 1169 0 1

5 687 16 699 1 3

5 688 16 1067 1 2

5 689 16 1269 1 2

5 690 8 549 1 1

5 691 8 626 1 2

5 692 16 843 1 4

5 693 32 794 1 4

5 694 32 798 1 5

5 695 16 586 1 3

5 696 32 664 1 4

5 697 32 872 1 1

5 698 32 613 1 4

5 699 16 727 1 3

5 700 32 749 0 5

5 701 16 703 1 5

5 702 32 782 1 1

5 703 32 712 1 3

5 704 16 916 1 2

5 705 8 672 1 2

5 706 16 1338 1 4

5 707 32 685 1 1

5 708 8 579 1 4

5 709 8 1253 1 2

5 710 16 894 1 4

5 711 8 576 0 3

5 712 8 606 1 4

5 713 32 726 1 1

5 714 32 899 1 3

5 715 16 663 1 2

5 716 8 579 1 3

5 717 32 576 1 2

5 718 32 634 1 3

5 719 8 617 1 5

5 720 32 682 0 2

5 721 32 1033 1 4

5 722 8 869 1 2

5 723 16 610 1 5

5 724 16 993 1 2

5 725 32 749 1 3

5 726 16 1309 1 4

5 727 32 872 1 2

5 728 16 812 1 3

5 729 16 962 1 1

5 730 8 608 1 5

5 731 32 1007 1 5

5 732 8 664 1 4

5 733 8 579 1 5

5 734 16 1104 1 1

5 735 8 579 1 5

5 736 32 742 1 5

5 737 16 1132 1 1

5 738 16 1128 1 5

5 739 32 705 1 2

5 740 32 897 1 4

5 741 16 1003 0 2

5 742 8 876 1 1

5 743 8 642 1 3

5 744 8 610 1 2

5 745 32 919 1 2

5 746 32 708 1 3

5 747 32 806 1 5

5 748 8 590 1 2

5 749 8 844 1 1

5 750 32 611 1 3

5 751 32 676 1 5

5 752 32 704 1 2

5 753 32 628 1 5

5 754 16 664 1 2

5 755 32 906 1 1

5 756 16 907 0 2

5 757 8 572 1 4

5 758 8 921 1 1

5 759 32 1532 1 2

5 760 32 937 1 5

5 761 16 898 0 2

5 762 32 807 1 1

5 763 8 600 1 4

5 764 32 1084 1 1

5 765 16 692 1 3

5 766 16 738 1 3

5 767 16 723 1 2

5 768 32 738 1 1

5 769 8 748 1 2

5 770 8 863 1 4

5 771 8 528 1 1

5 772 16 736 0 4

5 773 32 1321 1 3

5 774 32 744 1 1

5 775 16 888 1 3

5 776 16 712 1 3

5 777 16 964 1 5

5 778 8 881 1 3

5 779 8 720 1 5

5 780 32 910 1 4

5 781 8 556 1 4

5 782 16 792 0 1

5 783 8 687 1 5

5 784 32 694 1 1

5 785 8 617 1 2

5 786 8 603 1 3

5 787 8 722 1 4

5 788 8 554 1 3

5 789 32 774 1 4

5 790 32 753 1 4

5 791 16 1123 1 4

5 792 16 611 1 5

5 793 16 1254 1 4

5 794 16 1006 1 1

5 795 32 631 1 3

5 796 8 799 1 4

5 797 32 651 1 1

5 798 8 617 1 5

5 799 16 669 1 5

5 800 32 762 1 1

5 801 8 537 0 5

5 802 8 795 1 4

5 803 8 729 1 2

5 804 16 863 1 4

5 805 16 625 1 5

5 806 16 795 1 4

5 807 8 584 1 3

5 808 16 650 0 1

5 809 16 869 1 2

5 810 32 968 1 2

5 811 8 648 1 3

5 812 8 655 1 1

5 813 16 650 1 3

5 814 32 848 1 2

5 815 8 634 1 3

5 816 32 835 1 4

5 817 16 679 1 5

5 818 32 592 1 3

5 819 8 487 1 3

5 820 32 989 1 3

5 821 16 697 1 2

5 822 32 796 1 5

5 823 8 886 1 2

5 824 8 639 1 2

5 825 8 874 1 1

5 826 32 869 1 3

5 827 16 1001 1 4

5 828 8 800 1 5

5 829 8 989 0 1

5 830 16 692 1 1

5 831 8 829 1 1

5 832 16 898 1 3

5 833 16 1169 1 4

5 834 16 736 1 3

5 835 32 698 1 2

5 836 16 580 1 5

5 837 32 829 1 5

5 838 16 736 1 1

5 839 32 706 1 4

5 840 32 704 1 4

5 841 16 743 1 1

5 842 16 624 0 1

5 843 16 718 1 5

5 844 8 570 1 2

5 845 32 1127 1 1

5 846 8 630 1 5

5 847 8 952 1 3

5 848 8 670 1 1

5 849 8 755 1 1

5 850 32 1052 1 5

5 851 16 624 1 5

5 852 16 961 0 1

5 853 8 896 1 4

5 854 8 913 0 1

5 855 8 640 1 2

5 856 16 767 1 4

5 857 8 750 1 4

5 858 32 723 1 3

5 859 8 858 1 4

5 860 16 2870 0 1

5 861 32 878 1 4

5 862 8 771 1 2

5 863 8 1043 1 4

5 864 32 666 1 3

5 865 8 600 1 5

5 866 16 663 1 5

5 867 16 682 0 1

5 868 8 811 1 1

5 869 8 865 1 2

5 870 32 635 1 2

5 871 32 752 1 5

5 872 16 691 1 3

5 873 16 858 1 4

5 874 16 549 1 3

5 875 8 554 1 5

5 876 16 750 0 2

5 877 8 758 1 4

5 878 32 667 1 4

5 879 32 654 1 5

5 880 16 1086 1 3

5 881 16 703 1 5

5 882 16 656 1 3

5 883 8 541 1 5

5 884 8 590 1 2

5 885 8 588 1 3

5 886 16 952 0 1

5 887 8 836 1 3

5 888 32 547 1 2

5 889 8 528 1 3

5 890 16 1093 1 2

5 891 32 687 1 2

5 892 32 630 1 4

5 893 8 746 1 2

5 894 32 741 1 4

5 895 8 530 1 4

5 896 8 655 1 2

5 897 32 809 1 3

5 898 16 857 1 2

5 899 8 578 1 4

5 900 32 549 1 4

5 901 16 705 1 4

5 902 16 845 1 4

5 903 16 547 1 5

5 904 16 712 0 2

5 905 16 813 1 5

5 906 32 776 1 5

5 907 32 1112 1 5

5 908 16 732 1 4

5 909 16 945 1 3

5 910 16 827 1 1

5 911 32 784 1 2

5 912 32 664 1 3

5 913 32 1558 1 1

5 914 32 825 1 5

5 915 8 656 1 4

5 916 16 776 1 4

5 917 32 865 1 1

5 918 32 643 1 3

5 919 8 580 1 3

5 920 16 1027 0 1

5 921 32 709 0 2

5 922 32 954 1 5

5 923 16 940 1 4

5 924 16 815 1 3

5 925 32 926 1 1

5 926 8 765 1 3

5 927 8 645 1 2

5 928 32 621 1 1

5 929 16 742 1 3

5 930 16 1134 0 2

5 931 16 939 1 5

5 932 32 772 1 2

5 933 32 757 1 4

5 934 8 625 1 5

5 935 32 789 1 2

5 936 32 688 1 3

5 937 8 561 1 3

5 938 8 592 1 5

5 939 16 868 0 2

5 940 32 748 1 3

5 941 8 696 1 1

5 942 8 864 1 1

5 943 32 699 1 3

5 944 32 661 1 1

5 945 16 901 0 2

5 946 8 671 1 5

5 947 8 1033 1 1

5 948 32 1029 1 1

5 949 16 776 1 5

5 950 8 1342 0 1

5 951 16 769 1 3

5 952 8 746 1 5

5 953 8 665 1 3

5 954 32 858 1 2

5 955 32 844 1 5

5 956 32 763 1 4

5 957 32 657 1 4

5 958 32 636 1 1

5 959 16 807 1 4

5 960 16 871 1 2

5 961 32 645 1 2

5 962 32 1496 1 5

5 963 32 898 1 3

5 964 8 637 1 2

5 965 8 706 1 4

5 966 32 797 1 5

5 967 8 527 0 4

5 968 32 729 1 2

5 969 16 952 1 1

5 970 16 828 0 3

5 971 16 1151 1 4

5 972 32 748 1 5

5 973 8 801 1 5

5 974 32 759 1 2

5 975 16 654 1 1

5 976 8 593 1 5

5 977 16 1597 0 2

5 978 8 900 1 1

5 979 16 638 1 2

5 980 16 695 1 3

5 981 32 831 1 1

5 982 8 582 1 5

5 983 16 741 1 2

5 984 16 526 1 5

5 985 8 816 1 1

5 986 16 569 1 5

5 987 8 558 1 4

5 988 16 676 1 3

5 989 32 755 1 3

5 990 32 627 1 4

5 991 8 622 1 4

5 992 16 825 1 4

5 993 8 548 1 5

5 994 32 754 1 1

5 995 16 864 0 1

5 996 32 905 1 1

5 997 16 915 0 2

5 998 16 829 1 2

5 999 8 584 1 3

5 1000 16 989 1 4

5 1001 8 624 0 3

5 1002 8 657 1 2

5 1003 32 674 1 5

5 1004 8 933 1 1

5 1005 8 575 1 5

5 1006 32 947 1 1

5 1007 32 716 1 4

5 1008 16 637 0 4

5 1009 8 730 1 3

5 1010 16 671 1 3

5 1011 32 690 0 3

5 1012 8 766 0 5

5 1013 8 828 1 1

5 1014 32 787 1 4

5 1015 32 763 1 2

5 1016 8 1230 1 2

5 1017 8 577 1 2

5 1018 16 1037 1 4

5 1019 8 577 1 2

5 1020 32 940 1 4

5 1021 16 558 0 5

5 1022 16 584 1 5

5 1023 8 640 1 3

5 1024 16 629 1 3

5 1025 16 766 0 2

5 1026 16 772 1 3

5 1027 8 667 1 1

5 1028 32 612 1 3

5 1029 16 1030 1 4

5 1030 16 838 1 4

5 1031 8 952 1 1

5 1032 8 494 1 5

5 1033 16 659 0 1

5 1034 8 630 1 4

5 1035 8 626 1 3

5 1036 32 1025 1 3

5 1037 32 631 1 4

5 1038 32 830 1 2

5 1039 8 575 1 4

5 1040 16 726 1 3

5 1041 16 878 1 1

5 1042 32 579 1 3

5 1043 16 752 0 2

5 1044 8 566 1 3

5 1045 32 767 1 4

5 1046 16 674 1 3

5 1047 32 709 1 3

5 1048 32 977 1 5

5 1049 8 762 1 5

5 1050 8 528 1 3

5 1051 16 609 1 5

5 1052 8 1061 1 1

5 1053 32 755 1 1

5 1054 16 774 1 5

5 1055 8 859 1 4

5 1056 32 821 1 5

5 1057 16 613 1 5

5 1058 16 1254 1 4

5 1059 32 646 1 2

5 1060 32 856 1 2

5 1061 32 779 1 1

5 1062 32 704 1 1

5 1063 32 783 1 3

5 1064 8 661 1 2

5 1065 8 600 1 2

5 1066 16 732 1 2

5 1067 32 859 1 5

5 1068 8 730 1 2

5 1069 32 762 1 5

5 1070 8 639 1 3

5 1071 8 640 1 4

5 1072 8 549 1 1

5 1073 16 679 1 1

5 1074 32 846 1 1

5 1075 16 588 1 5

5 1076 16 637 1 1

5 1077 16 827 1 1

5 1078 32 805 1 2

5 1079 32 606 1 4

5 1080 32 660 1 4

5 1081 16 804 1 3

5 1082 8 1082 1 4

5 1083 8 619 1 3

5 1084 8 674 1 2

5 1085 32 672 1 3

5 1086 8 714 1 4

5 1087 32 660 1 2

5 1088 8 1758 1 1

5 1089 32 873 1 3

5 1090 32 702 1 1

5 1091 16 791 1 3

5 1092 8 597 1 5

5 1093 8 640 1 4

5 1094 32 824 1 3

5 1095 16 1917 1 2

5 1096 16 1048 1 4

5 1097 16 878 1 3

5 1098 32 882 1 3

5 1099 16 715 1 5

5 1100 16 854 1 4

5 1101 8 688 1 2

5 1102 32 791 1 5

5 1103 16 804 1 4

5 1104 8 674 1 5

5 1105 16 722 1 3

5 1106 16 1051 1 3

5 1107 8 627 1 5

5 1108 32 841 1 4

5 1109 32 696 1 2

5 1110 32 893 1 1

5 1111 8 735 0 2

5 1112 16 900 1 5

5 1113 8 1025 1 4

5 1114 8 658 1 5

5 1115 16 1217 1 2

5 1116 16 643 1 5

5 1117 32 752 1 4

5 1118 16 1067 1 4

5 1119 8 664 1 1

5 1120 8 635 1 2

5 1121 32 804 1 1

5 1122 16 787 1 2

5 1123 32 752 1 3

5 1124 32 660 1 4

5 1125 32 775 1 5

5 1126 8 617 1 2

5 1127 16 746 1 1

5 1128 32 861 1 5

5 1129 16 575 1 5

5 1130 32 692 1 5

5 1131 32 857 1 2

5 1132 16 1039 1 3

5 1133 16 846 1 1

5 1134 32 1115 1 1

5 1135 32 827 1 2

5 1136 32 752 1 4

5 1137 32 798 1 5

5 1138 8 1055 1 1

5 1139 32 856 1 1

5 1140 32 729 1 5

5 1141 8 905 1 1

5 1142 8 667 1 2

5 1143 8 1442 1 1

5 1144 32 690 1 3

5 1145 32 829 1 1

5 1146 32 902 1 4

5 1147 8 717 1 2

5 1148 8 564 1 5

5 1149 8 531 1 3

5 1150 16 751 1 2

5 1151 8 567 1 5

5 1152 8 932 1 1

5 1153 16 722 1 5

5 1154 8 584 1 3

5 1155 16 849 1 3

5 1156 16 854 0 1

5 1157 8 731 1 4

5 1158 32 852 1 1

5 1159 32 751 1 3

5 1160 8 735 1 3

5 1161 8 2641 1 1

5 1162 32 606 1 2

5 1163 8 679 1 4

5 1164 16 702 1 5

5 1165 32 955 1 1

5 1166 8 857 1 2

5 1167 16 1238 0 2

5 1168 16 705 1 1

5 1169 32 711 1 2

5 1170 32 713 1 3

5 1171 8 611 1 3

5 1172 16 943 1 1

5 1173 8 598 1 3

5 1174 16 854 1 4

5 1175 8 924 1 3

5 1176 16 1291 1 1

5 1177 32 772 1 4

5 1178 32 1536 1 5

5 1179 16 1010 1 1

5 1180 32 680 1 4

5 1181 32 676 1 2

5 1182 16 933 1 4

5 1183 8 787 1 1

5 1184 16 828 1 2

5 1185 8 640 1 3

5 1186 32 677 1 2

5 1187 16 651 1 5

5 1188 16 1315 1 2

5 1189 16 540 1 5

5 1190 16 843 1 1

5 1191 32 858 1 5

5 1192 32 681 1 4

5 1193 8 601 1 5

5 1194 8 725 1 4

5 1195 16 691 1 4

5 1196 16 927 1 4

5 1197 8 580 1 5

5 1198 16 734 0 2

5 1199 16 677 1 3

5 1200 8 649 1 4

6 1 16 1697 1 3

6 2 16 1550 1 5

6 3 32 1071 1 1

6 4 16 1002 1 5

6 5 8 1044 1 2

6 6 32 1089 1 5

6 7 8 1342 1 2

6 8 32 1344 0 4

6 9 8 1162 1 5

6 10 16 1147 1 3

6 11 16 1013 1 3

6 12 16 2375 1 3

6 13 8 1407 1 1

6 14 32 1110 1 1

6 15 8 1027 1 5

6 16 32 1031 0 2

6 17 32 949 1 5

6 18 8 1119 1 3

6 19 32 1154 1 2

6 20 16 1040 1 4

6 21 16 1223 1 1

6 22 16 2198 0 2

6 23 8 1137 0 1

6 24 16 1025 0 2

6 25 8 1004 1 2

6 26 8 1817 1 2

6 27 8 964 1 5

6 28 8 769 1 3

6 29 8 1257 1 5

6 30 8 2378 0 5

6 31 32 1472 1 1

6 32 32 1041 1 5

6 33 32 1081 1 1

6 34 8 833 1 5

6 35 32 839 1 2

6 36 16 1209 1 4

6 37 32 868 0 2

6 38 32 1178 1 1

6 39 32 1030 1 3

6 40 16 1033 1 5

6 41 8 1243 0 4

6 42 32 923 1 3

6 43 32 1120 1 3

6 44 16 1457 1 1

6 45 16 1439 1 2

6 46 8 1193 1 1

6 47 16 921 1 3

6 48 16 2074 1 1

6 49 8 1229 1 3

6 50 32 1397 1 5

6 51 8 1984 1 1

6 52 16 1332 1 3

6 53 16 1682 1 1

6 54 16 1013 1 5

6 55 32 1408 0 2

6 56 8 1570 1 1

6 57 32 1307 1 4

6 58 32 940 1 3

6 59 8 1008 1 3

6 60 32 1532 0 4

6 61 8 1290 1 3

6 62 32 1004 1 3

6 63 16 1449 1 4

6 64 8 1064 1 1

6 65 16 1172 1 3

6 66 16 937 1 2

6 67 32 934 1 5

6 68 8 1294 1 2

6 69 16 852 0 3

6 70 8 1445 1 4

6 71 16 1262 1 4

6 72 32 1533 1 1

6 73 32 1093 1 4

6 74 16 1516 1 1

6 75 8 926 0 4

6 76 32 1096 0 5

6 77 8 1903 1 1

6 78 8 821 1 3

6 79 16 1701 1 2

6 80 16 1502 1 2

6 81 8 1371 1 4

6 82 16 1194 1 5

6 83 8 1506 1 1

6 84 32 1181 1 3

6 85 32 1294 1 1

6 86 8 1514 1 2

6 87 32 858 1 3

6 88 32 1044 1 1

6 89 32 885 1 4

6 90 8 793 1 3

6 91 16 999 1 5

6 92 8 1231 1 2

6 93 8 1585 1 4

6 94 16 1318 0 4

6 95 32 772 1 4

6 96 32 732 1 2

6 97 32 701 1 2

6 98 16 1497 0 2

6 99 16 1074 1 4

6 100 32 850 1 3

6 101 16 1680 1 4

6 102 16 918 1 5

6 103 16 1065 1 5

6 104 16 2158 1 1

6 105 8 1062 1 3

6 106 8 846 1 5

6 107 16 1373 1 4

6 108 32 883 1 4

6 109 32 1002 1 5

6 110 16 2052 1 1

6 111 32 1433 1 4

6 112 16 2361 0 2

6 113 16 3787 0 1

6 114 32 851 1 2

6 115 8 1296 1 4

6 116 8 1155 1 4

6 117 32 1144 1 5

6 118 8 1879 1 4

6 119 8 680 1 2

6 120 8 983 1 5

6 121 16 1719 1 3

6 122 8 661 1 3

6 123 32 861 1 1

6 124 16 1657 0 2

6 125 8 1771 1 4

6 126 16 1324 1 3

6 127 8 1404 1 3

6 128 32 1442 1 1

6 129 8 938 1 1

6 130 8 964 1 2

6 131 32 987 1 5

6 132 32 1001 1 2

6 133 16 1814 1 4

6 134 8 1458 1 1

6 135 8 1385 1 4

6 136 8 958 1 2

6 137 32 1097 1 1

6 138 32 1076 1 5

6 139 32 1007 1 5

6 140 8 1010 1 1

6 141 32 1169 1 5

6 142 8 1397 1 2

6 143 16 1173 0 2

6 144 16 1559 1 3

6 145 16 1301 1 1

6 146 16 2146 1 1

6 147 32 1660 1 2

6 148 16 976 0 4

6 149 16 877 1 5

6 150 32 1265 1 1

6 151 32 1127 1 2

6 152 16 1358 1 1

6 153 16 1282 1 4

6 154 8 1129 1 3

6 155 32 1130 1 1

6 156 8 1114 1 5

6 157 8 851 1 3

6 158 32 933 1 3

6 159 8 1247 0 4

6 160 16 954 1 5

6 161 8 1370 0 5

6 162 16 1657 1 4

6 163 32 898 1 5

6 164 16 1067 1 4

6 165 16 1343 1 1

6 166 8 1461 1 3

6 167 16 1142 1 5

6 168 8 755 1 5

6 169 16 1019 0 2

6 170 32 755 1 4

6 171 32 1288 0 1

6 172 32 1019 1 3

6 173 8 1263 1 1

6 174 16 1038 0 4

6 175 32 695 1 2

6 176 32 948 1 4

6 177 32 897 1 5

6 178 16 1796 1 1

6 179 16 1406 1 1

6 180 8 1004 1 4

6 181 32 1052 1 2

6 182 8 814 1 3

6 183 16 1125 1 3

6 184 8 1403 1 1

6 185 8 1011 1 2

6 186 32 1484 1 3

6 187 8 1471 1 2

6 188 16 1274 1 5

6 189 16 872 1 5

6 190 16 1171 1 3

6 191 32 1049 1 4

6 192 16 1002 0 5

6 193 32 1150 1 4

6 194 8 886 1 3

6 195 16 1413 1 2

6 196 32 1076 1 4

6 197 16 1222 1 5

6 198 32 1017 1 4

6 199 32 1019 1 5

6 200 16 1441 1 3

6 201 8 1321 1 5

6 202 32 1331 1 3

6 203 32 1335 0 2

6 204 8 3177 1 5

6 205 8 3578 1 4

6 206 8 1363 1 4

6 207 8 994 1 5

6 208 32 1732 1 4

6 209 32 1307 1 3

6 210 8 1922 1 4

6 211 32 1063 1 1

6 212 16 1450 1 3

6 213 32 1033 1 3

6 214 32 1667 1 1

6 215 16 1895 1 1

6 216 8 999 1 4

6 217 16 1255 1 5

6 218 8 1317 1 2

6 219 16 1202 0 4

6 220 8 1319 1 1

6 221 32 1215 1 2

6 222 32 1421 1 2

6 223 16 1266 1 2

6 224 16 1262 1 3

6 225 16 1643 1 2

6 226 16 1608 0 2

6 227 8 1367 1 2

6 228 32 1398 1 5

6 229 8 2187 1 5

6 230 8 1732 1 3

6 231 32 3165 1 3

6 232 8 1187 0 1

6 233 16 3011 1 1

6 234 8 938 1 2

6 235 8 1502 1 1

6 236 16 992 0 2

6 237 32 949 1 3

6 238 8 1092 1 5

6 239 32 1099 1 4

6 240 16 1082 1 4

6 241 16 932 1 5

6 242 16 1379 1 3

6 243 32 1433 1 5

6 244 8 1478 1 4

6 245 8 970 1 3

6 246 8 1050 1 3

6 247 32 866 1 1

6 248 16 838 1 4

6 249 32 932 1 1

6 250 8 843 1 4

6 251 8 979 1 1

6 252 32 758 1 4

6 253 32 790 1 2

6 254 8 843 1 2

6 255 8 1257 1 1

6 256 16 1519 1 5

6 257 32 966 1 3

6 258 16 1238 1 2

6 259 8 916 1 5

6 260 32 889 1 1

6 261 16 1598 1 4

6 262 16 2737 0 2

6 263 16 670 1 5

6 264 32 1353 1 3

6 265 16 1818 1 5

6 266 32 838 1 1

6 267 16 1455 1 1

6 268 8 1700 1 4

6 269 16 1316 0 4

6 270 32 844 1 5

6 271 8 1306 1 1

6 272 32 1096 1 5

6 273 32 827 1 5

6 274 8 1016 1 1

6 275 8 1097 0 2

6 276 8 887 1 3

6 277 16 1555 0 2

6 278 8 1065 1 3

6 279 16 788 0 4

6 280 16 1195 1 5

6 281 32 1277 1 3

6 282 32 988 1 3

6 283 16 2765 1 1

6 284 32 727 1 3

6 285 16 973 1 5

6 286 8 1167 1 3

6 287 16 1015 1 5

6 288 16 1503 1 1

6 289 32 857 1 4

6 290 8 1044 1 2

6 291 8 1251 1 1

6 292 8 963 1 5

6 293 8 899 1 3

6 294 8 1338 1 4

6 295 8 1183 1 5

6 296 32 1596 1 1

6 297 32 1344 1 3

6 298 8 1544 1 1

6 299 32 1155 1 2

6 300 8 1071 1 1

6 301 8 1211 1 2

6 302 8 788 1 3

6 303 8 842 1 2

6 304 16 1152 0 4

6 305 16 1446 0 4

6 306 32 1198 1 2

6 307 32 748 1 4

6 308 8 890 1 3

6 309 32 1073 1 5

6 310 16 1212 1 2

6 311 16 964 1 3

6 312 32 1067 1 5

6 313 16 1341 0 1

6 314 16 1504 1 1

6 315 32 1272 0 4

6 316 32 1221 1 2

6 317 8 1173 1 4

6 318 8 1110 1 4

6 319 32 811 1 4

6 320 8 1538 1 1

6 321 16 1140 1 3

6 322 16 991 1 1

6 323 32 1113 1 5

6 324 32 976 1 5

6 325 32 977 1 3

6 326 16 5022 1 2

6 327 16 2655 1 3

6 328 8 1104 1 5

6 329 32 1308 1 1

6 330 8 911 1 5

6 331 8 1082 1 2

6 332 16 1614 1 1

6 333 32 936 1 1

6 334 16 1776 0 2

6 335 16 1918 1 2

6 336 8 1089 1 5

6 337 16 930 1 4

6 338 32 1176 1 2

6 339 16 1431 1 4

6 340 16 1125 1 3

6 341 32 1187 1 2

6 342 32 1184 1 1

6 343 8 1011 1 5

6 344 8 924 1 2

6 345 8 1019 1 5

6 346 16 1595 1 3

6 347 32 1014 1 2

6 348 16 1472 1 2

6 349 8 709 1 4

6 350 32 1051 1 3

6 351 16 1425 1 1

6 352 16 946 1 5

6 353 8 1111 1 4

6 354 32 1474 1 4

6 355 32 863 1 4

6 356 32 1614 1 4

6 357 16 1264 1 3

6 358 8 1129 1 2

6 359 32 1588 1 2

6 360 16 1336 1 3

6 361 16 896 0 4

6 362 8 1060 1 1

6 363 16 791 1 4

6 364 32 939 1 5

6 365 32 1134 1 5

6 366 8 1070 1 3

6 367 8 1290 1 1

6 368 8 842 1 5

6 369 32 1033 1 5

6 370 16 1070 1 5

6 371 16 1616 1 3

6 372 16 2936 1 4

6 373 8 1250 1 4

6 374 16 1891 1 1

6 375 16 1473 1 5

6 376 8 957 1 2

6 377 8 1513 1 4

6 378 8 647 1 3

6 379 16 1476 1 4

6 380 8 929 1 3

6 381 32 974 1 2

6 382 16 809 1 5

6 383 32 890 1 4

6 384 16 1151 1 3

6 385 8 797 1 3

6 386 16 837 1 5

6 387 32 770 1 4

6 388 8 1504 1 1

6 389 8 866 1 1

6 390 32 1148 1 2

6 391 16 832 1 2

6 392 32 925 1 3

6 393 32 791 1 4

6 394 16 795 1 5

6 395 32 882 1 5

6 396 16 1694 1 4

6 397 32 722 1 3

6 398 8 1082 1 5

6 399 16 2097 1 1

6 400 8 793 1 3

6 401 32 1144 1 1

6 402 8 699 0 2

6 403 32 959 1 1

6 404 32 855 1 1

6 405 32 1016 1 1

6 406 32 1114 1 2

6 407 8 1113 1 4

6 408 32 1077 1 2

6 409 32 914 1 2

6 410 16 1912 0 2

6 411 16 852 1 3

6 412 32 1462 1 1

6 413 8 1411 0 4

6 414 16 966 1 3

6 415 16 1761 1 4

6 416 32 849 1 5

6 417 16 1877 0 1

6 418 8 1025 1 5

6 419 32 964 1 4

6 420 8 1502 0 1

6 421 8 1175 1 4

6 422 16 1465 1 1

6 423 32 1104 1 3

6 424 32 1448 1 5

6 425 16 1768 0 2

6 426 8 895 1 2

6 427 8 735 1 3

6 428 32 1252 1 1

6 429 8 1387 1 4

6 430 16 1118 1 5

6 431 8 957 1 5

6 432 32 1642 1 1

6 433 16 2206 0 2

6 434 16 1284 1 3

6 435 16 1063 1 3

6 436 16 1086 1 2

6 437 8 968 1 5

6 438 16 1014 1 5

6 439 16 2876 1 2

6 440 16 1552 1 1

6 441 16 1455 1 1

6 442 32 750 1 4

6 443 32 942 1 4

6 444 8 909 0 2

6 445 8 1138 1 3

6 446 16 3234 0 1

6 447 8 949 1 2

6 448 32 1158 1 2

6 449 32 911 1 3

6 450 16 1342 1 3

6 451 32 866 1 5

6 452 32 1296 1 1

6 453 8 951 1 2

6 454 16 1420 1 4

6 455 32 749 1 2

6 456 8 1038 1 2

6 457 16 1297 1 5

6 458 8 1119 1 2

6 459 16 1202 1 3

6 460 32 859 1 4

6 461 8 920 1 5

6 462 32 889 1 5

6 463 8 916 1 1

6 464 8 817 1 1

6 465 8 696 0 4

6 466 16 1367 1 4

6 467 32 1029 1 3

6 468 8 904 1 1

6 469 32 1237 0 3

6 470 32 720 1 2

6 471 16 1443 0 1

6 472 16 1428 0 2

6 473 16 2095 0 2

6 474 8 939 1 5

6 475 8 691 1 4

6 476 8 1000 1 5

6 477 8 1196 1 3

6 478 32 2038 1 3

6 479 32 609 1 3

6 480 32 1072 1 4

6 481 16 2083 1 4

6 482 32 787 1 5

6 483 16 1017 1 3

6 484 16 811 1 5

6 485 16 1287 0 2

6 486 32 664 1 2

6 487 32 818 1 5

6 488 32 662 1 4

6 489 8 1133 1 3

6 490 32 695 1 4

6 491 16 1464 1 2

6 492 8 1022 1 3

6 493 16 1384 1 4

6 494 32 771 1 1

6 495 32 569 1 3

6 496 8 1182 1 2

6 497 8 890 1 2

6 498 8 796 1 5

6 499 16 908 0 4

6 500 32 883 1 5

6 501 32 1024 1 5

6 502 8 1057 1 5

6 503 32 1330 1 1

6 504 32 1046 1 2

6 505 8 1030 1 3

6 506 8 1153 1 2

6 507 8 1124 1 4

6 508 16 883 1 3

6 509 8 1073 1 5

6 510 8 1005 1 2

6 511 32 1304 1 4

6 512 8 720 1 2

6 513 16 1152 1 4

6 514 8 897 0 1

6 515 8 1564 1 1

6 516 16 2611 1 2

6 517 32 875 1 5

6 518 16 960 1 5

6 519 32 1062 1 2

6 520 32 1145 1 1

6 521 8 1228 1 4

6 522 32 841 1 3

6 523 8 897 1 5

6 524 8 1290 1 1

6 525 32 1033 1 1

6 526 8 1187 1 1

6 527 32 748 1 4

6 528 16 850 0 4

6 529 8 1112 1 1

6 530 8 829 1 5

6 531 32 1072 1 1

6 532 16 1768 1 1

6 533 16 840 1 5

6 534 32 1105 1 5

6 535 32 1076 1 1

6 536 16 1329 1 1

6 537 8 897 1 5

6 538 16 3232 1 1

6 539 8 1602 1 4

6 540 8 1010 1 1

6 541 16 730 1 3

6 542 16 732 1 5

6 543 32 923 1 5

6 544 8 1254 1 1

6 545 16 1334 1 1

6 546 16 1327 0 2

6 547 8 2432 1 4

6 548 16 1781 0 2

6 549 32 945 1 3

6 550 32 1393 1 3

6 551 16 1531 1 2

6 552 16 1354 1 5

6 553 8 852 1 3

6 554 32 1539 1 2

6 555 32 1223 1 2

6 556 32 753 0 2

6 557 32 999 1 1

6 558 16 1213 1 1

6 559 8 1008 1 4

6 560 16 1079 1 2

6 561 8 1529 0 1

6 562 32 1351 1 3

6 563 16 1291 0 4

6 564 8 931 1 5

6 565 16 1002 1 3

6 566 32 1162 1 4

6 567 8 1279 1 2

6 568 16 1487 1 1

6 569 16 926 1 3

6 570 32 968 1 3

6 571 8 1259 1 3

6 572 32 908 1 3

6 573 32 704 1 4

6 574 16 839 1 5

6 575 8 990 1 2

6 576 32 780 1 5

6 577 8 688 1 4

6 578 8 681 1 2

6 579 16 1310 1 2

6 580 16 2060 1 3

6 581 16 1271 1 3

6 582 8 812 1 3

6 583 8 797 1 3

6 584 32 858 1 4

6 585 16 773 1 5

6 586 16 1016 1 3

6 587 16 907 1 5

6 588 8 1634 1 4

6 589 32 821 1 3

6 590 32 863 1 4

6 591 16 1817 1 4

6 592 16 2700 0 1

6 593 32 1293 1 2

6 594 16 1027 1 4

6 595 8 1263 1 4

6 596 16 1271 1 1

6 597 8 1217 1 3

6 598 32 1314 1 2

6 599 32 1135 1 1

6 600 8 1024 1 5

6 601 16 1315 1 3

6 602 8 705 1 2

6 603 8 796 1 4

6 604 16 874 1 3

6 605 8 895 1 1

6 606 32 1191 1 3

6 607 16 1751 1 1

6 608 16 1178 1 1

6 609 8 765 1 3

6 610 8 1052 1 1

6 611 8 695 1 3

6 612 8 1047 1 1

6 613 8 784 1 2

6 614 16 936 1 2

6 615 8 1083 1 2

6 616 32 1003 1 4

6 617 8 835 1 5

6 618 16 1071 1 3

6 619 8 891 1 3

6 620 32 1034 1 5

6 621 32 833 1 5

6 622 16 1492 1 5

6 623 32 842 1 3

6 624 8 1203 1 2

6 625 32 1058 1 2

6 626 16 1171 1 3

6 627 32 897 1 4

6 628 8 915 1 2

6 629 8 992 1 1

6 630 32 957 1 3

6 631 8 858 1 2

6 632 32 950 1 2

6 633 32 819 1 5

6 634 16 838 1 5

6 635 32 804 1 3

6 636 16 708 1 5

6 637 32 868 1 3

6 638 32 848 1 2

6 639 32 802 1 4

6 640 8 825 1 1

6 641 16 1265 1 4

6 642 32 851 1 4

6 643 8 1033 1 1

6 644 8 704 1 3

6 645 16 1136 0 2

6 646 8 830 1 4

6 647 32 931 1 3

6 648 16 1076 1 2

6 649 16 856 1 1

6 650 8 970 1 5

6 651 32 1240 1 1

6 652 32 865 1 4

6 653 16 1076 0 2

6 654 16 1758 0 2

6 655 16 1038 1 4

6 656 8 874 1 2

6 657 8 631 1 5

6 658 32 829 1 4

6 659 32 1185 1 1

6 660 32 803 1 2

6 661 32 1185 1 1

6 662 32 941 1 3

6 663 16 912 1 5

6 664 16 996 1 4

6 665 32 834 1 5

6 666 8 903 1 3

6 667 16 827 0 4

6 668 16 796 1 5

6 669 32 673 1 4

6 670 32 671 1 2

6 671 16 1211 1 1

6 672 32 717 1 1

6 673 16 953 1 2

6 674 32 934 1 2

6 675 16 930 1 3

6 676 8 992 1 5

6 677 8 1497 1 4

6 678 16 640 1 3

6 679 8 991 1 4

6 680 16 754 1 5

6 681 8 1153 1 1

6 682 32 792 1 5

6 683 16 1029 1 1

6 684 16 922 1 5

6 685 8 753 1 3

6 686 16 1000 1 4

6 687 32 982 1 2

6 688 32 692 1 5

6 689 16 1034 1 4

6 690 8 948 1 5

6 691 16 873 1 3

6 692 16 1608 1 4

6 693 8 1088 1 1

6 694 32 1158 1 1

6 695 32 1242 1 1

6 696 32 1034 1 3

6 697 16 2079 1 1

6 698 8 872 1 3

6 699 32 875 1 5

6 700 32 740 1 2

6 701 16 1362 1 2

6 702 16 1129 1 1

6 703 32 1001 1 1

6 704 8 848 1 4

6 705 16 791 1 4

6 706 8 854 1 3

6 707 32 1079 1 1

6 708 8 735 1 5

6 709 8 629 1 5

6 710 8 848 1 4

6 711 16 778 1 1

6 712 8 757 1 4

6 713 8 642 1 4

6 714 8 670 1 5

6 715 16 580 1 5

6 716 8 1021 1 2

6 717 32 727 1 4

6 718 32 1016 1 5

6 719 16 1647 1 2

6 720 16 926 0 3

6 721 16 1204 1 2

6 722 16 686 1 5

6 723 16 1251 1 4

6 724 16 1328 0 3

6 725 8 668 1 3

6 726 16 1169 1 1

6 727 8 681 1 5

6 728 16 794 1 1

6 729 8 807 1 5

6 730 8 981 1 2

6 731 16 1356 1 2

6 732 32 657 1 1

6 733 16 1201 0 2

6 734 8 678 0 1

6 735 8 1489 1 1

6 736 8 795 1 3

6 737 16 738 1 1

6 738 16 637 1 5

6 739 32 620 1 3

6 740 32 1022 1 3

6 741 32 1174 1 1

6 742 32 756 1 2

6 743 32 984 1 5

6 744 8 741 1 3

6 745 8 1125 1 1

6 746 8 806 1 4

6 747 8 680 1 2

6 748 32 665 1 5

6 749 16 701 1 5

6 750 8 840 1 5

6 751 8 1039 1 2

6 752 16 792 1 5

6 753 8 1656 1 4

6 754 8 834 1 1

6 755 8 816 1 1

6 756 32 953 1 5

6 757 8 768 1 3

6 758 8 736 1 2

6 759 16 1191 1 1

6 760 32 965 1 2

6 761 8 815 1 4

6 762 16 880 1 3

6 763 32 810 1 5

6 764 16 1233 1 2

6 765 8 816 1 4

6 766 32 1179 0 2

6 767 8 1573 1 2

6 768 32 779 0 3

6 769 32 1124 1 1

6 770 32 913 1 2

6 771 32 1090 1 3

6 772 16 1150 1 1

6 773 32 885 1 4

6 774 16 1018 1 3

6 775 8 1020 1 5

6 776 8 1572 1 4

6 777 32 771 1 4

6 778 32 969 1 3

6 779 16 1189 1 2

6 780 16 845 0 4

6 781 32 578 1 1

6 782 32 563 1 4

6 783 16 513 1 5

6 784 32 662 1 3

6 785 8 671 1 3

6 786 16 2034 1 4

6 787 16 1719 1 1

6 788 32 943 1 5

6 789 32 609 1 2

6 790 32 644 1 1

6 791 16 1115 0 3

6 792 16 1859 1 3

6 793 32 1126 1 1

6 794 8 1145 1 4

6 795 8 1608 1 1

6 796 8 1105 1 4

6 797 32 1613 1 3

6 798 32 940 1 2

6 799 16 952 1 5

6 800 32 762 1 1

6 801 16 637 1 5

6 802 16 847 1 3

6 803 16 1429 1 5

6 804 32 675 1 5

6 805 8 693 1 5

6 806 32 923 1 1

6 807 16 739 1 4

6 808 32 679 1 4

6 809 16 1073 1 1

6 810 16 860 1 4

6 811 8 891 1 3

6 812 32 779 1 5

6 813 32 849 1 4

6 814 32 740 1 2

6 815 8 965 1 5

6 816 8 955 1 2

6 817 32 981 1 2

6 818 16 897 1 3

6 819 16 1479 1 1

6 820 16 948 0 4

6 821 8 596 1 5

6 822 32 765 1 5

6 823 16 871 0 4

6 824 32 941 1 4

6 825 16 4274 1 2

6 826 8 859 1 2

6 827 16 1065 1 3

6 828 8 882 1 3

6 829 8 713 1 3

6 830 16 1223 0 2

6 831 32 906 1 4

6 832 8 1043 1 1

6 833 16 800 1 4

6 834 32 1184 1 4

6 835 8 923 1 5

6 836 8 1238 1 1

6 837 8 1202 1 4

6 838 16 1614 1 2

6 839 32 940 1 3

6 840 8 877 1 2

6 841 32 1050 1 2

6 842 16 1025 1 3

6 843 16 1332 1 2

6 844 32 927 1 5

6 845 8 790 1 3

6 846 16 965 1 5

6 847 8 731 1 2

6 848 32 1052 1 2

6 849 32 796 1 4

6 850 32 844 1 3

6 851 8 904 0 2

6 852 8 917 1 5

6 853 16 1195 0 3

6 854 16 1855 1 1

6 855 16 1463 0 2

6 856 32 947 1 4

6 857 32 1105 1 1

6 858 16 3161 0 1

6 859 16 976 1 1

6 860 8 689 1 3

6 861 8 723 0 2

6 862 16 862 1 3

6 863 8 1137 1 5

6 864 8 1107 1 1

6 865 16 1295 0 3

6 866 32 950 1 1

6 867 32 948 1 4

6 868 32 938 1 4

6 869 8 869 1 5

6 870 16 2107 0 2

6 871 8 1233 1 1

6 872 32 1729 1 4

6 873 8 784 1 3

6 874 16 1067 1 4

6 875 16 1568 1 1

6 876 8 1724 1 1

6 877 8 921 1 1

6 878 16 1484 1 4

6 879 16 996 1 3

6 880 8 745 1 2

6 881 16 1850 0 1

6 882 32 1220 1 2

6 883 8 1047 1 5

6 884 32 719 1 3

6 885 32 821 1 1

6 886 32 665 1 4

6 887 16 1389 1 1

6 888 8 1041 1 4

6 889 8 833 1 2

6 890 16 1056 1 5

6 891 8 1267 1 1

6 892 16 1522 0 4

6 893 16 1077 1 4

6 894 16 2505 1 2

6 895 16 1900 0 2

6 896 8 652 1 5

6 897 8 559 1 3

6 898 32 1161 0 3

6 899 8 1082 1 2

6 900 16 1002 0 4

6 901 8 1052 0 5

6 902 8 1779 1 4

6 903 8 1161 1 3

6 904 16 885 1 5

6 905 8 1457 1 4

6 906 32 1148 1 5

6 907 32 906 1 1

6 908 16 1673 1 1

6 909 32 1234 1 3

6 910 8 871 1 2

6 911 16 1244 0 4

6 912 8 1049 1 4

6 913 32 1350 1 4

6 914 16 1195 1 1

6 915 32 1342 1 1

6 916 16 1185 1 3

6 917 32 908 1 2

6 918 8 1168 1 5

6 919 32 1185 1 5

6 920 32 854 1 5

6 921 32 1122 1 1

6 922 8 881 1 3

6 923 16 1368 0 2

6 924 32 1081 1 5

6 925 8 1096 1 3

6 926 32 1145 1 3

6 927 32 1332 1 3

6 928 8 1711 1 4

6 929 8 984 1 4

6 930 32 1011 1 5

6 931 16 1213 1 2

6 932 8 1532 1 1

6 933 8 925 1 1

6 934 16 1576 1 4

6 935 16 781 1 5

6 936 16 1764 1 4

6 937 32 1441 1 1

6 938 32 1366 1 2

6 939 32 2081 1 1

6 940 8 895 1 1

6 941 16 986 1 5

6 942 32 1205 1 5

6 943 8 868 1 2

6 944 32 5924 1 2

6 945 8 2166 1 4

6 946 8 982 1 4

6 947 32 1462 1 2

6 948 8 890 1 5

6 949 16 1228 1 2

6 950 32 787 1 3

6 951 16 824 1 5

6 952 16 928 1 3

6 953 32 609 1 3

6 954 32 857 1 5

6 955 32 1164 1 4

6 956 16 1155 1 5

6 957 32 619 1 2

6 958 16 792 1 5

6 959 8 734 1 3

6 960 16 1175 1 3

6 961 16 879 1 3

6 962 8 1097 1 4

6 963 8 988 1 3

6 964 8 883 1 5

6 965 16 1344 0 2

6 966 16 909 1 3

6 967 8 805 1 3

6 968 16 1141 1 4

6 969 32 1031 1 2

6 970 16 901 1 1

6 971 8 832 1 1

6 972 32 769 1 3

6 973 8 619 1 5

6 974 16 1354 1 4

6 975 16 1182 1 1

6 976 32 1001 1 1

6 977 16 1454 1 4

6 978 8 2282 1 4

6 979 32 835 1 3

6 980 16 1049 1 5

6 981 8 753 1 2

6 982 32 958 1 4

6 983 16 964 1 5

6 984 16 1034 1 3

6 985 8 872 1 4

6 986 16 776 1 5

6 987 16 2346 1 1

6 988 16 1214 1 2

6 989 16 937 1 3

6 990 32 923 1 4

6 991 8 763 1 2

6 992 8 708 1 3

6 993 8 1041 1 1

6 994 32 1025 1 3

6 995 32 1271 1 1

6 996 32 1338 1 4

6 997 16 1219 0 2

6 998 32 625 1 3

6 999 32 901 1 4

6 1000 16 894 1 5

6 1001 32 863 1 3

6 1002 8 699 1 5

6 1003 8 649 1 2

6 1004 16 722 0 4

6 1005 8 1107 1 1

6 1006 8 730 1 3

6 1007 32 756 1 2

6 1008 8 1262 1 1

6 1009 16 746 1 4

6 1010 8 696 1 2

6 1011 8 814 1 4

6 1012 8 775 1 4

6 1013 16 781 1 3

6 1014 32 713 1 2

6 1015 8 708 0 3

6 1016 16 829 0 2

6 1017 8 1140 1 1

6 1018 32 1124 1 2

6 1019 32 799 1 5

6 1020 8 1026 1 2

6 1021 32 806 1 3

6 1022 32 1093 1 5

6 1023 16 873 1 2

6 1024 16 870 0 3

6 1025 8 774 1 3

6 1026 8 690 1 3

6 1027 32 1344 1 1

6 1028 16 827 1 4

6 1029 16 1256 1 1

6 1030 32 891 1 1

6 1031 8 805 1 2

6 1032 32 1036 1 5

6 1033 32 1290 1 1

6 1034 32 908 1 4

6 1035 32 1190 1 5

6 1036 32 926 1 2

6 1037 8 1203 1 1

6 1038 16 930 1 5

6 1039 16 1195 1 2

6 1040 32 968 1 5

6 1041 8 882 1 4

6 1042 8 1177 1 4

6 1043 32 852 1 5

6 1044 8 805 1 5

6 1045 32 935 1 3

6 1046 8 865 1 5

6 1047 8 820 1 5

6 1048 32 873 1 2

6 1049 16 1378 1 1

6 1050 16 690 1 5

6 1051 32 760 1 5

6 1052 8 897 1 3

6 1053 8 973 1 5

6 1054 32 1138 1 1

6 1055 32 845 1 2

6 1056 32 1140 1 1

6 1057 16 1006 1 3

6 1058 16 1978 0 2

6 1059 16 1539 1 1

6 1060 32 1147 1 5

6 1061 8 1096 1 1

6 1062 16 1234 1 4

6 1063 16 809 1 5

6 1064 32 880 1 4

6 1065 32 1294 1 1

6 1066 16 1551 1 2

6 1067 8 852 1 2

6 1068 16 786 1 5

6 1069 16 956 1 3

6 1070 32 992 1 2

6 1071 16 1148 1 4

6 1072 16 1393 1 1

6 1073 16 1244 1 1

6 1074 32 930 1 4

6 1075 8 1306 1 5

6 1076 32 988 1 4

6 1077 8 1035 1 1

6 1078 8 1070 1 4

6 1079 32 956 1 3

6 1080 8 706 1 2

6 1081 32 1129 1 3

6 1082 16 761 1 5

6 1083 32 836 1 1

6 1084 8 1190 1 2

6 1085 8 805 1 5

6 1086 16 1378 1 3

6 1087 16 1011 1 4

6 1088 16 741 1 5

6 1089 8 1175 1 2

6 1090 8 987 1 2

6 1091 16 826 1 5

6 1092 32 762 1 1

6 1093 16 793 0 3

6 1094 8 1168 1 1

6 1095 16 714 1 1

6 1096 32 880 1 2

6 1097 16 1136 1 4

6 1098 32 772 1 4

6 1099 16 1499 1 1

6 1100 8 712 1 1

6 1101 32 805 1 5

6 1102 32 655 1 3

6 1103 32 659 1 4

6 1104 16 790 1 5

6 1105 32 877 1 3

6 1106 32 675 1 2

6 1107 32 681 1 1

6 1108 16 1415 1 1

6 1109 32 723 1 2

6 1110 8 844 1 3

6 1111 8 693 1 1

6 1112 8 627 1 3

6 1113 16 881 1 1

6 1114 8 712 1 3

6 1115 8 737 1 2

6 1116 32 1028 1 1

6 1117 16 1461 1 1

6 1118 8 857 1 5

6 1119 8 1205 0 4

6 1120 32 752 1 5

6 1121 8 723 1 3

6 1122 16 884 1 2

6 1123 32 588 1 2

6 1124 8 646 1 5

6 1125 32 1094 1 2

6 1126 8 611 1 3

6 1127 16 1257 1 2

6 1128 32 885 1 3

6 1129 32 703 1 5

6 1130 32 929 1 2

6 1131 16 1486 1 4

6 1132 8 1443 1 1

6 1133 32 1290 1 1

6 1134 8 770 1 5

6 1135 8 1027 1 2

6 1136 32 1627 1 2

6 1137 8 1304 1 4

6 1138 8 772 1 3

6 1139 16 1244 1 3

6 1140 16 879 1 2

6 1141 32 862 1 3

6 1142 16 2291 0 2

6 1143 16 757 1 1

6 1144 16 802 0 4

6 1145 16 1006 1 2

6 1146 16 826 1 2

6 1147 8 1150 1 4

6 1148 32 910 1 5

6 1149 32 705 1 4

6 1150 8 759 1 5

6 1151 8 771 1 1

6 1152 32 620 1 4

6 1153 32 586 0 3

6 1154 8 1900 1 4

6 1155 32 857 1 5

6 1156 8 702 1 5

6 1157 16 1034 1 3

6 1158 8 868 1 4

6 1159 16 931 1 4

6 1160 32 955 1 2

6 1161 8 914 1 5

6 1162 8 1226 1 1

6 1163 16 1064 1 1

6 1164 32 828 1 4

6 1165 8 769 1 2

6 1166 32 896 1 4

6 1167 32 909 1 1

6 1168 8 898 1 2

6 1169 8 751 1 3

6 1170 32 1003 1 1

6 1171 8 804 1 4

6 1172 32 1082 1 1

6 1173 8 980 1 4

6 1174 8 1413 1 1

6 1175 16 730 0 4

6 1176 16 1004 1 5

6 1177 16 1298 1 4

6 1178 16 944 1 3

6 1179 32 1073 1 5

6 1180 16 1342 1 2

6 1181 32 897 1 5

6 1182 16 1100 1 3

6 1183 32 1008 1 3

6 1184 32 967 1 5

6 1185 16 1301 1 5

6 1186 32 780 1 4

6 1187 8 878 1 4

6 1188 32 821 1 3

6 1189 16 1024 1 2

6 1190 32 725 1 4

6 1191 16 664 1 3

6 1192 16 779 1 5

6 1193 16 1199 1 4

6 1194 8 1325 1 1

6 1195 8 686 1 5

6 1196 16 1214 1 1

6 1197 16 658 1 5

6 1198 8 959 1 2

6 1199 8 879 0 3

6 1200 16 872 0 3

7 1 32 638 0 5

7 2 32 636 1 3

7 3 32 1033 1 1

7 4 16 655 1 5

7 5 16 670 1 4

7 6 16 723 1 3

7 7 8 552 0 1

7 8 16 791 1 3

7 9 8 660 0 5

7 10 16 637 1 5

7 11 16 648 1 5

7 12 16 915 0 1

7 13 32 540 1 5

7 14 32 668 1 4

7 15 32 508 1 4

7 16 8 532 0 5

7 17 8 1247 0 4

7 18 32 1045 1 1

7 19 32 511 1 5

7 20 16 787 1 2

7 21 16 1072 1 3

7 22 8 626 0 3

7 23 16 522 1 5

7 24 8 443 0 5

7 25 8 849 0 4

7 26 32 624 1 1

7 27 16 952 1 3

7 28 8 561 0 5

7 29 32 528 1 4

7 30 32 497 1 5

7 31 16 909 1 1

7 32 8 740 0 4

7 33 32 590 1 3

7 34 16 931 1 1

7 35 8 624 0 2

7 36 32 410 1 4

7 37 32 1092 0 2

7 38 16 709 1 1

7 39 8 1058 0 1

7 40 16 519 1 5

7 41 16 1145 1 2

7 42 32 522 1 3

7 43 16 677 1 4

7 44 8 560 0 4

7 45 16 1242 1 1

7 46 8 663 0 4

7 47 8 945 0 1

7 48 16 556 1 2

7 49 16 976 1 2

7 50 8 892 1 5

7 51 16 646 0 1

7 52 32 558 1 3

7 53 8 811 1 2

7 54 16 830 1 3

7 55 32 494 1 4

7 56 16 1039 1 1

7 57 32 490 1 5

7 58 8 644 0 3

7 59 8 880 1 1

7 60 32 477 1 2

7 61 16 769 0 4

7 62 32 1133 1 3

7 63 32 732 0 1

7 64 8 691 0 1

7 65 16 470 0 4

7 66 32 751 1 1

7 67 8 500 0 3

7 68 16 496 0 4

7 69 16 700 1 4

7 70 16 745 1 5

7 71 8 1650 0 2

7 72 8 541 0 5

7 73 8 688 1 5

7 74 16 1805 1 2

7 75 8 498 0 2

7 76 8 510 0 2

7 77 8 546 0 3

7 78 8 623 0 1

7 79 8 1275 1 2

7 80 32 747 1 2

7 81 32 1082 1 2

7 82 32 893 1 2

7 83 32 1017 1 3

7 84 8 985 1 2

7 85 8 698 1 3

7 86 16 1270 1 2

7 87 32 1342 1 5

7 88 32 779 0 1

7 89 8 605 0 3

7 90 16 739 1 2

7 91 32 833 1 2

7 92 8 957 1 4

7 93 8 1221 1 1

7 94 8 729 1 4

7 95 8 902 0 3

7 96 32 585 1 1

7 97 16 1329 1 3

7 98 8 977 0 5

7 99 16 494 1 5

7 100 16 635 0 2

7 101 8 975 1 2

7 102 16 1269 1 5

7 103 8 934 1 1

7 104 16 1552 1 3

7 105 32 713 1 3

7 106 32 879 0 1

7 107 8 799 1 3

7 108 32 1067 1 5

7 109 32 731 0 2

7 110 32 1268 1 4

7 111 16 795 0 1

7 112 8 955 0 4

7 113 16 852 0 4

7 114 16 646 1 3

7 115 32 1434 1 2

7 116 16 1646 0 4

7 117 32 662 1 3

7 118 32 1056 1 5

7 119 32 667 1 4

7 120 32 762 0 4

7 121 8 608 0 4

7 122 8 523 0 4

7 123 32 484 1 3

7 124 32 612 1 4

7 125 16 638 0 1

7 126 8 459 0 4

7 127 8 1562 1 1

7 128 32 730 1 5

7 129 8 655 0 4

7 130 8 545 0 2

7 131 8 803 1 4

7 132 8 776 0 1

7 133 8 485 1 2

7 134 32 1162 1 2

7 135 32 1481 0 3

7 136 16 797 1 2

7 137 16 874 1 1

7 138 32 623 1 2

7 139 8 650 0 3

7 140 8 1050 1 1

7 141 32 879 1 5

7 142 8 509 0 5

7 143 8 1145 0 2

7 144 16 3185 1 2

7 145 16 644 0 4

7 146 16 594 1 2

7 147 8 766 0 5

7 148 8 1221 0 1

7 149 8 1232 1 3

7 150 8 570 0 3

7 151 32 543 1 4

7 152 8 657 0 1

7 153 32 1083 0 5

7 154 32 1322 1 1

7 155 8 831 1 3

7 156 16 687 1 1

7 157 16 781 1 5

7 158 16 1260 0 3

7 159 8 701 0 2

7 160 8 746 0 3

7 161 32 1302 0 5

7 162 8 930 0 2

7 163 8 892 0 5

7 164 16 728 0 4

7 165 8 523 0 3

7 166 32 654 1 4

7 167 8 1281 0 2

7 168 32 791 1 4

7 169 16 691 0 4

7 170 16 999 1 1

7 171 32 627 1 1

7 172 32 807 1 4

7 173 8 511 0 5

7 174 16 781 0 1

7 175 32 1543 0 1

7 176 16 1028 1 4

7 177 16 901 0 4

7 178 32 689 1 5

7 179 8 703 0 2

7 180 32 1582 1 4

7 181 16 1183 0 3

7 182 8 1160 0 5

7 183 16 1254 1 1

7 184 32 1185 1 3

7 185 16 939 1 3

7 186 16 611 1 3

7 187 32 635 1 3

7 188 8 415 0 3

7 189 16 947 1 5

7 190 32 1208 1 5

7 191 16 2102 1 3

7 192 8 703 0 5

7 193 16 714 1 5

7 194 16 655 1 5

7 195 16 639 1 3

7 196 32 540 1 2

7 197 16 590 1 3

7 198 8 394 1 4

7 199 8 603 0 4

7 200 16 516 1 4

7 201 32 1112 0 2

7 202 8 833 0 5

7 203 32 659 0 3

7 204 32 1163 1 3

7 205 32 1303 0 5

7 206 16 777 1 5

7 207 16 1057 0 5

7 208 32 526 1 3

7 209 32 907 1 2

7 210 32 913 0 1

7 211 32 904 0 5

7 212 16 805 1 1

7 213 16 685 0 2

7 214 16 744 0 5

7 215 8 501 0 3

7 216 16 1167 1 4

7 217 16 2360 0 2

7 218 16 778 1 2

7 219 32 724 1 1

7 220 8 682 1 2

7 221 8 1322 1 1

7 222 32 921 1 2

7 223 32 799 1 1

7 224 8 759 1 1

7 225 16 1372 1 4

7 226 32 1239 1 1

7 227 8 806 1 5

7 228 8 1155 0 1

7 229 32 515 1 1

7 230 8 1335 1 4

7 231 16 1008 1 2

7 232 32 928 1 4

7 233 32 743 0 2

7 234 32 433 1 4

7 235 16 751 0 1

7 236 32 1700 0 2

7 237 16 616 1 3

7 238 32 698 1 3

7 239 16 1793 0 2

7 240 16 1596 1 5

7 241 32 956 1 3

7 242 16 1191 1 1

7 243 8 866 0 4

7 244 32 1423 0 5

7 245 32 723 1 3

7 246 32 1071 0 1

7 247 16 607 0 4

7 248 16 1260 1 1

7 249 8 1275 0 5

7 250 32 784 1 4

7 251 8 2116 1 3

7 252 32 1881 1 2

7 253 8 1603 0 5

7 254 8 959 1 2

7 255 16 913 0 1

7 256 8 870 0 3

7 257 32 1146 0 1

7 258 32 1225 0 1

7 259 16 1399 1 2

7 260 16 892 1 1

7 261 8 1336 0 4

7 262 8 1204 1 5

7 263 8 913 0 4

7 264 32 799 1 2

7 265 16 1152 0 1

7 266 16 2007 0 5

7 267 16 1708 1 1

7 268 8 530 0 3

7 269 16 953 0 2

7 270 32 1262 0 4

7 271 32 2462 1 1

7 272 8 1843 1 2

7 273 8 1367 1 1

7 274 16 892 1 5

7 275 32 1361 1 1

7 276 32 784 1 4

7 277 16 729 0 4

7 278 8 1136 0 2

7 279 8 598 1 3

7 280 8 777 0 2

7 281 32 1288 0 1

7 282 16 1724 1 5

7 283 8 852 0 5

7 284 32 961 1 3

7 285 32 1007 0 4

7 286 16 1023 1 5

7 287 8 1031 0 5

7 288 32 646 1 4

7 289 32 1234 0 5

7 290 32 865 1 4

7 291 8 777 0 1

7 292 16 688 0 3

7 293 32 1491 0 1

7 294 16 821 0 4

7 295 32 1117 0 2

7 296 32 1020 1 2

7 297 8 710 0 3

7 298 32 1242 1 5

7 299 16 1166 1 3

7 300 32 636 1 5

7 301 16 704 0 2

7 302 8 956 1 4

7 303 32 657 1 4

7 304 16 739 1 5

7 305 8 676 1 1

7 306 8 626 0 5

7 307 16 483 0 4

7 308 16 890 0 4

7 309 16 1592 0 2

7 310 16 630 1 5

7 311 8 1152 1 4

7 312 16 1250 0 1

7 313 16 1025 1 2

7 314 16 770 1 3

7 315 8 724 0 5

7 316 16 1400 0 1

7 317 32 798 1 3

7 318 32 513 1 4

7 319 8 443 0 2

7 320 32 233 0 5

7 321 32 1359 0 2

7 322 16 1094 1 2

7 323 8 435 0 3

7 324 8 404 1 1

7 325 8 683 1 2

7 326 16 801 1 5

7 327 32 1379 0 3

7 328 16 8898 1 4

7 329 32 1550 1 2

7 330 16 524 1 2

7 331 8 318 1 1

7 332 32 468 1 3

7 333 32 674 1 5

7 334 16 1233 1 3

7 335 8 647 0 4

7 336 8 2629 1 1

7 337 8 1765 1 3

7 338 8 1360 0 4

7 339 32 1978 1 1

7 340 16 1234 1 3

7 341 8 2963 0 4

7 342 8 1091 1 3

7 343 32 1844 1 2

7 344 8 1391 1 1

7 345 16 3667 1 2

7 346 16 1349 1 3

7 347 8 1008 1 2

7 348 16 1394 1 4

7 349 16 1459 1 5

7 350 32 2555 1 3

7 351 8 1785 1 2

7 352 32 2613 1 3

7 353 16 2215 1 4

7 354 32 2417 1 5

7 355 16 2291 0 3

7 356 16 1239 1 3

7 357 8 1196 1 5

7 358 8 1127 1 1

7 359 32 2829 0 5

7 360 32 1709 1 2

7 361 16 1199 1 3

7 362 16 1041 1 5

7 363 16 673 1 5

7 364 16 1045 0 2

7 365 8 1043 1 2

7 366 32 1790 0 5

7 367 8 1385 1 5

7 368 16 1923 1 1

7 369 32 1664 0 2

7 370 16 1424 0 1

7 371 8 1187 1 3

7 372 32 2146 1 1

7 373 16 1997 1 4

7 374 32 1910 1 5

7 375 32 1301 1 2

7 376 32 2461 1 1

7 377 32 1642 1 1

7 378 8 1454 1 3

7 379 8 1649 1 4

7 380 32 1661 1 4

7 381 16 1493 1 5

7 382 16 1387 1 3

7 383 16 2425 1 1

7 384 32 2196 1 1

7 385 16 2887 1 2

7 386 8 1060 1 5

7 387 8 1093 1 4

7 388 8 1736 1 5

7 389 16 1575 1 5

7 390 32 1351 0 5

7 391 32 1530 1 1

7 392 16 1295 1 5

7 393 8 930 1 5

7 394 16 1843 1 2

7 395 32 1693 1 2

7 396 16 1420 1 1

7 397 8 807 1 4

7 398 16 1901 0 2

7 399 32 1376 1 4

7 400 8 2606 1 1

7 401 16 2288 1 1

7 402 8 992 1 4

7 403 16 1257 1 5

7 404 16 1105 1 4

7 405 8 749 0 2

7 406 32 898 0 2

7 407 32 981 1 1

7 408 16 1132 1 4

7 409 32 1336 1 4

7 410 16 923 1 2

7 411 8 1168 1 3

7 412 32 1635 1 2

7 413 8 1029 1 5

7 414 16 1061 0 2

7 415 16 1110 1 3

7 416 16 1278 0 4

7 417 8 883 1 3

7 418 32 1399 1 4

7 419 16 2486 0 2

7 420 32 1579 1 5

7 421 8 1267 1 3

7 422 32 1118 1 3

7 423 8 1909 1 2

7 424 8 730 1 1

7 425 16 1478 0 2

7 426 8 771 1 5

7 427 16 1130 0 3

7 428 8 778 1 3

7 429 32 1385 1 3

7 430 32 970 1 5

7 431 32 1947 1 2

7 432 16 846 1 4

7 433 8 997 0 4

7 434 32 1639 1 2

7 435 8 1185 0 1

7 436 16 1246 0 4

7 437 32 1395 1 3

7 438 8 1055 1 2

7 439 16 2263 1 1

7 440 16 1413 1 3

7 441 32 1240 1 4

7 442 32 949 1 5

7 443 32 959 1 2

7 444 8 1036 1 3

7 445 8 1246 1 1

7 446 32 1155 1 3

7 447 8 1293 1 2

7 448 8 950 1 3

7 449 16 2385 0 4

7 450 16 974 1 5

7 451 8 998 1 2

7 452 8 996 1 4

7 453 32 1053 1 3

7 454 8 1398 1 2

7 455 32 1415 1 5

7 456 16 1803 1 5

7 457 32 1105 1 5

7 458 16 1289 1 1

7 459 8 1055 1 1

7 460 32 1373 1 4

7 461 32 1038 1 4

7 462 16 1343 1 3

7 463 16 1176 1 3

7 464 8 1293 1 2

7 465 32 1230 1 4

7 466 8 866 1 4

7 467 32 1060 0 1

7 468 32 1158 1 3

7 469 8 1081 1 5

7 470 8 973 1 1

7 471 8 1873 0 1

7 472 32 975 1 3

7 473 16 975 0 4

7 474 8 682 1 5

7 475 16 1607 0 1

7 476 8 934 1 1

7 477 16 944 0 3

7 478 8 923 1 4

7 479 32 1236 1 1

7 480 32 972 1 3

7 481 16 862 1 3

7 482 8 1477 1 2

7 483 32 960 1 2

7 484 32 831 1 4

7 485 32 795 1 3

7 486 16 912 0 2

7 487 8 735 1 3

7 488 32 1741 0 2

7 489 32 1238 1 3

7 490 32 1129 1 4

7 491 16 1199 1 3

7 492 32 1305 1 2

7 493 16 1864 0 1

7 494 32 1599 0 2

7 495 8 2694 0 1

7 496 16 2761 0 1

7 497 32 938 0 4

7 498 8 1282 1 4

7 499 16 1167 0 4

7 500 16 2962 1 1

7 501 16 3196 1 1

7 502 8 992 1 2

7 503 8 776 1 3

7 504 16 1207 1 5

7 505 16 1180 0 2

7 506 8 1623 1 1

7 507 32 1128 1 4

7 508 16 974 1 3

7 509 16 912 1 5

7 510 32 3788 1 1

7 511 8 1504 1 1

7 512 8 1055 1 2

7 513 8 1047 1 4

7 514 8 781 1 1

7 515 8 1129 1 4

7 516 8 956 1 3

7 517 32 1322 1 3

7 518 32 1233 1 4

7 519 32 598 1 2

7 520 16 888 1 3

7 521 32 885 0 5

7 522 8 1559 1 3

7 523 16 3026 1 2

7 524 8 702 1 5

7 525 32 945 0 3

7 526 32 947 1 3

7 527 16 2445 1 5

7 528 16 2519 1 2

7 529 16 3485 1 2

7 530 16 1280 1 5

7 531 16 1066 1 3

7 532 16 1854 1 2

7 533 32 1250 1 4

7 534 16 1034 1 5

7 535 16 1257 1 3

7 536 16 1234 1 4

7 537 32 901 1 4

7 538 16 1666 1 4

7 539 32 1055 1 5

7 540 8 1920 1 1

7 541 8 1127 1 4

7 542 32 900 1 5

7 543 8 749 1 3

7 544 16 1973 1 1

7 545 32 2352 1 1

7 546 32 1099 1 5

7 547 16 1234 1 5

7 548 16 1386 0 4

7 549 8 908 1 1

7 550 8 910 1 2

7 551 8 941 1 5

7 552 8 965 1 2

7 553 32 3135 1 1

7 554 8 1189 1 2

7 555 16 1048 1 4

7 556 32 1438 1 1

7 557 32 904 1 3

7 558 16 769 1 5

7 559 16 838 1 4

7 560 32 1457 1 1

7 561 32 835 1 1

7 562 32 1196 0 5

7 563 8 776 1 3

7 564 8 949 1 2

7 565 8 728 1 4

7 566 8 832 0 3

7 567 8 1011 1 5

7 568 8 1419 1 1

7 569 16 820 1 5

7 570 16 1564 1 1

7 571 32 1101 1 3

7 572 32 1000 1 4

7 573 16 1211 1 3

7 574 32 1404 1 2

7 575 16 1506 1 1

7 576 32 1147 1 5

7 577 32 1381 1 2

7 578 8 1217 1 5

7 579 32 1341 1 1

7 580 16 2825 0 2

7 581 16 793 0 4

7 582 8 673 1 5

7 583 32 1348 1 5

7 584 16 1296 1 1

7 585 32 1031 1 5

7 586 8 1114 1 5

7 587 8 852 1 3

7 588 32 800 0 3

7 589 8 1482 0 2

7 590 8 1180 0 4

7 591 8 1234 1 1

7 592 32 2002 1 2

7 593 32 2003 1 1

7 594 16 1292 1 3

7 595 8 1733 1 4

7 596 8 941 1 5

7 597 16 3237 1 2

7 598 8 788 1 4

7 599 16 1494 1 4

7 600 8 794 1 5

7 601 8 1081 1 3

7 602 32 1039 1 3

7 603 32 965 1 2

7 604 16 804 1 5

7 605 32 805 1 5

7 606 8 692 1 3

7 607 8 1058 0 2

7 608 16 692 1 5

7 609 16 1210 0 1

7 610 32 962 1 3

7 611 32 1756 1 1

7 612 16 982 1 3

7 613 8 919 1 2

7 614 16 1200 1 3

7 615 32 1048 1 1

7 616 16 3212 0 1

7 617 32 1543 1 2

7 618 8 1135 1 1

7 619 32 960 1 2

7 620 32 840 1 2

7 621 32 1188 1 5

7 622 8 1031 1 2

7 623 16 1002 1 4

7 624 8 683 1 3

7 625 32 977 1 5

7 626 8 843 1 3

7 627 8 750 1 2

7 628 32 780 1 3

7 629 8 684 1 1

7 630 32 701 1 5

7 631 16 795 0 4

7 632 8 658 1 5

7 633 32 730 1 4

7 634 16 1073 1 3

7 635 32 1243 0 4

7 636 8 1647 1 4

7 637 8 678 1 5

7 638 32 923 1 2

7 639 16 690 1 5

7 640 16 745 1 3

7 641 16 1209 1 1

7 642 16 1104 1 2

7 643 32 811 1 4

7 644 16 803 0 4

7 645 32 1185 1 3

7 646 8 994 1 1

7 647 16 2048 0 2

7 648 8 738 1 5

7 649 8 1035 1 1

7 650 8 737 1 5

7 651 32 1337 1 1

7 652 8 689 1 4

7 653 32 824 1 3

7 654 32 667 1 3

7 655 32 1084 1 3

7 656 32 1637 1 1

7 657 8 952 1 4

7 658 32 1010 1 4

7 659 32 897 0 2

7 660 16 646 1 5

7 661 8 1187 1 1

7 662 16 1642 1 4

7 663 32 1174 1 1

7 664 8 698 0 5

7 665 8 875 1 2

7 666 32 739 1 4

7 667 8 803 1 4

7 668 8 731 1 1

7 669 16 1080 1 2

7 670 32 760 1 4

7 671 16 678 0 5

7 672 8 603 0 4

7 673 16 629 1 5

7 674 8 658 1 3

7 675 16 1391 1 2

7 676 32 1217 1 5

7 677 32 1049 1 1

7 678 8 737 1 2

7 679 8 770 1 1

7 680 16 1168 1 4

7 681 16 1204 0 4

7 682 8 804 1 3

7 683 8 955 1 5

7 684 16 837 1 3

7 685 32 1271 1 1

7 686 16 1127 1 1

7 687 8 812 1 3

7 688 32 1100 1 5

7 689 16 702 1 3

7 690 16 735 1 4

7 691 16 785 1 1

7 692 16 1058 0 2

7 693 16 1552 1 1

7 694 16 1035 1 5

7 695 8 1389 1 4

7 696 32 855 1 2

7 697 8 776 1 5

7 698 8 841 1 2

7 699 32 870 1 5

7 700 16 1112 1 1

7 701 16 661 1 5

7 702 32 848 1 5

7 703 32 726 0 3

7 704 8 935 0 1

7 705 8 854 1 2

7 706 16 847 1 4

7 707 32 1357 1 1

7 708 8 657 1 3

7 709 16 1017 1 3

7 710 16 935 1 3

7 711 16 1041 1 2

7 712 32 1121 1 4

7 713 8 801 0 4

7 714 16 1235 0 2

7 715 8 881 1 5

7 716 8 776 1 4

7 717 32 905 1 4

7 718 16 1116 0 2

7 719 32 904 1 2

7 720 16 807 1 1

7 721 32 1031 1 4

7 722 16 755 1 1

7 723 8 695 1 2

7 724 32 752 1 1

7 725 8 1005 1 5

7 726 32 611 1 3

7 727 8 641 1 5

7 728 8 643 1 5

7 729 16 803 0 2

7 730 32 677 0 2

7 731 16 684 1 5

7 732 8 842 1 1

7 733 8 683 1 4

7 734 16 643 1 5

7 735 16 707 1 2

7 736 16 720 1 4

7 737 8 656 0 1

7 738 16 677 0 4

7 739 32 962 1 2

7 740 16 936 1 2

7 741 8 659 1 4

7 742 8 611 1 4

7 743 8 852 1 3

7 744 32 914 1 4

7 745 32 901 1 5

7 746 16 1029 1 2

7 747 32 1870 1 1

7 748 8 657 1 4

7 749 8 722 1 3

7 750 8 857 1 4

7 751 32 977 1 1

7 752 16 1018 1 5

7 753 8 834 1 1

7 754 8 759 1 5

7 755 8 1025 1 1

7 756 16 780 1 5

7 757 16 813 1 2

7 758 32 1077 1 2

7 759 8 713 1 1

7 760 32 949 1 2

7 761 32 913 1 1

7 762 16 1745 1 4

7 763 16 881 1 1

7 764 8 1754 1 1

7 765 32 1539 1 4

7 766 16 1472 1 4

7 767 8 630 1 2

7 768 8 1358 1 2

7 769 32 717 1 2

7 770 32 626 1 2

7 771 8 775 1 4

7 772 32 712 1 3

7 773 8 582 1 3

7 774 32 793 1 5

7 775 16 1291 1 4

7 776 32 540 0 3

7 777 32 990 1 1

7 778 32 756 1 5

7 779 32 610 1 3

7 780 32 888 0 3

7 781 8 1031 1 1

7 782 32 788 1 4

7 783 32 616 1 3

7 784 16 1574 1 5

7 785 32 934 1 1

7 786 8 848 1 2

7 787 16 1778 1 1

7 788 16 963 1 1

7 789 8 665 1 3

7 790 16 581 0 3

7 791 16 1471 0 1

7 792 32 1035 0 5

7 793 8 1048 1 2

7 794 32 782 0 5

7 795 32 1016 1 5

7 796 16 1221 1 1

7 797 8 775 1 1

7 798 16 752 1 1

7 799 8 1083 1 3

7 800 16 676 1 5

7 801 32 1237 1 4

7 802 32 610 1 4

7 803 16 1016 1 3

7 804 8 642 1 5

7 805 16 826 1 3

7 806 8 717 1 5

7 807 16 889 1 4

7 808 16 846 0 1

7 809 32 883 0 1

7 810 16 540 1 5

7 811 8 838 1 3

7 812 8 683 1 4

7 813 32 819 1 5

7 814 32 884 1 5

7 815 32 891 1 3

7 816 16 594 1 3

7 817 16 551 1 3

7 818 16 557 0 3

7 819 8 1186 1 2

7 820 8 906 1 4

7 821 16 965 0 2

7 822 16 829 0 2

7 823 32 939 1 4

7 824 16 925 1 5

7 825 16 1622 1 4

7 826 32 800 1 2

7 827 8 807 1 2

7 828 32 759 0 3

7 829 8 830 1 2

7 830 16 1064 0 2

7 831 8 738 1 5

7 832 8 781 1 3

7 833 16 1394 1 4

7 834 8 805 1 3

7 835 8 947 1 5

7 836 32 1244 1 1

7 837 16 931 0 3

7 838 32 899 0 4

7 839 16 1650 1 3

7 840 32 1247 1 2

7 841 16 68 0 3

7 842 32 1018 1 3

7 843 32 907 1 5

7 844 16 1030 1 3

7 845 16 1167 1 1

7 846 32 1253 1 3

7 847 16 800 1 3

7 848 16 825 1 5

7 849 8 699 1 3

7 850 8 540 1 3

7 851 32 742 1 5

7 852 8 1087 1 5

7 853 32 1227 1 1

7 854 32 786 1 1

7 855 8 691 1 2

7 856 32 842 1 1

7 857 16 977 0 2

7 858 8 718 1 1

7 859 32 842 1 4

7 860 32 801 1 2

7 861 32 872 1 2

7 862 32 684 1 2

7 863 16 754 1 3

7 864 8 713 1 3

7 865 32 764 1 4

7 866 16 924 1 5

7 867 32 1343 1 2

7 868 32 643 1 5

7 869 16 1071 0 1

7 870 8 679 1 4

7 871 32 787 0 1

7 872 16 1407 0 2

7 873 16 767 1 4

7 874 8 1157 1 3

7 875 8 992 1 4

7 876 16 996 1 5

7 877 16 1739 1 1

7 878 16 1338 1 4

7 879 8 887 1 1

7 880 16 1045 1 1

7 881 8 1063 1 1

7 882 8 933 1 1

7 883 8 719 1 4

7 884 16 978 1 2

7 885 8 822 1 5

7 886 8 727 1 2

7 887 16 727 0 2

7 888 8 641 1 1

7 889 16 753 1 5

7 890 32 852 0 4

7 891 16 888 1 1

7 892 16 727 1 5

7 893 8 997 1 2

7 894 16 1231 1 3

7 895 32 946 1 2

7 896 8 584 1 4

7 897 8 594 1 2

7 898 32 1134 1 2

7 899 32 904 1 1

7 900 16 1207 1 4

7 901 32 829 1 3

7 902 32 659 1 5

7 903 32 932 1 5

7 904 16 725 1 3

7 905 16 612 1 5

7 906 8 675 1 5

7 907 8 911 1 5

7 908 8 907 1 3

7 909 16 783 1 1

7 910 16 634 1 5

7 911 8 775 1 2

7 912 32 871 1 4

7 913 32 1766 1 3

7 914 16 835 0 2

7 915 8 833 1 3

7 916 8 726 1 5

7 917 32 849 1 4

7 918 32 769 1 1

7 919 32 824 0 1

7 920 16 1019 1 4

7 921 8 754 1 4

7 922 32 709 1 2

7 923 8 1525 1 2

7 924 16 1034 1 4

7 925 8 1069 1 1

7 926 8 737 1 5

7 927 16 1009 0 4

7 928 8 573 1 3

7 929 32 813 1 1

7 930 32 789 1 5

7 931 16 609 0 4

7 932 16 603 0 4

7 933 8 700 1 4

7 934 16 1013 1 1

7 935 32 981 1 4

7 936 32 925 1 3

7 937 8 908 1 2

7 938 16 895 1 3

7 939 16 1079 1 2

7 940 32 1012 1 5

7 941 8 1269 0 1

7 942 32 852 1 3

7 943 32 857 1 4

7 944 16 842 0 2

7 945 8 629 1 5

7 946 32 652 1 4

7 947 8 1208 1 4

7 948 32 862 1 3

7 949 32 1157 1 3

7 950 8 763 1 5

7 951 32 944 1 2

7 952 8 858 1 3

7 953 16 1107 0 2

7 954 16 793 1 5

7 955 16 1046 0 1

7 956 8 990 1 1

7 957 32 1128 1 5

7 958 8 688 1 4

7 959 16 726 1 3

7 960 8 869 1 2

7 961 16 858 1 5

7 962 8 684 1 4

7 963 16 635 1 5

7 964 8 839 0 4

7 965 32 1053 1 5

7 966 32 798 1 2

7 967 16 925 0 1

7 968 8 836 1 2

7 969 8 826 1 2

7 970 8 783 1 5

7 971 16 792 1 3

7 972 32 725 0 1

7 973 32 940 0 3

7 974 16 636 0 2

7 975 8 673 1 3

7 976 16 966 1 4

7 977 8 651 1 3

7 978 16 1929 1 1

7 979 32 732 1 3

7 980 8 905 0 1

7 981 16 1456 1 3

7 982 16 1050 1 5

7 983 32 1089 1 4

7 984 32 916 1 2

7 985 8 700 1 5

7 986 8 658 1 1

7 987 8 658 1 1

7 988 16 1173 1 4

7 989 16 979 0 3

7 990 16 880 0 1

7 991 32 951 1 2

7 992 16 792 0 2

7 993 32 872 1 2

7 994 16 708 1 5

7 995 16 965 1 4

7 996 32 1156 1 4

7 997 8 1055 1 1

7 998 32 945 1 1

7 999 8 811 1 2

7 1000 8 733 1 1

7 1001 32 870 1 3

7 1002 16 903 0 4

7 1003 16 814 1 3

7 1004 8 872 1 1

7 1005 8 754 1 3

7 1006 32 968 1 4

7 1007 32 766 1 5

7 1008 32 621 1 3

7 1009 8 720 1 5

7 1010 16 759 1 5

7 1011 8 1137 1 5

7 1012 32 633 1 2

7 1013 32 934 1 1

7 1014 8 1019 1 2

7 1015 8 700 1 3

7 1016 32 835 1 4

7 1017 16 1026 1 4

7 1018 32 811 1 3

7 1019 32 773 0 4

7 1020 8 1057 1 3

7 1021 8 1030 1 2

7 1022 16 800 1 5

7 1023 8 796 1 2

7 1024 16 956 1 1

7 1025 32 1055 1 1

7 1026 8 941 1 2

7 1027 32 1136 0 1

7 1028 32 1057 0 5

7 1029 16 1389 1 1

7 1030 32 883 1 1

7 1031 16 1107 1 3

7 1032 8 1028 1 3

7 1033 8 816 1 5

7 1034 16 1181 0 2

7 1035 32 741 1 2

7 1036 16 696 1 5

7 1037 8 1081 1 4

7 1038 16 995 1 2

7 1039 32 691 1 1

7 1040 8 905 1 5

7 1041 16 1196 0 2

7 1042 16 748 0 4

7 1043 32 942 1 5

7 1044 32 924 1 3

7 1045 16 828 0 2

7 1046 8 705 1 4

7 1047 16 943 1 3

7 1048 16 1242 1 4

7 1049 32 1077 0 2

7 1050 8 821 1 4

7 1051 8 1085 1 1

7 1052 32 968 1 3

7 1053 32 981 1 5

7 1054 32 605 1 4

7 1055 8 770 1 3

7 1056 32 948 1 4

7 1057 16 1213 1 5

7 1058 8 838 1 5

7 1059 32 800 1 2

7 1060 16 946 1 3

7 1061 8 644 1 5

7 1062 32 1203 1 1

7 1063 8 669 1 4

7 1064 32 725 1 4

7 1065 32 962 1 5

7 1066 8 614 1 4

7 1067 16 1076 1 1

7 1068 32 774 1 3

7 1069 32 807 1 5

7 1070 8 796 1 2

7 1071 16 933 1 4

7 1072 8 652 1 3

7 1073 16 787 1 2

7 1074 32 866 1 5

7 1075 8 666 1 4

7 1076 16 1033 1 1

7 1077 16 948 1 1

7 1078 16 739 1 2

7 1079 16 1018 1 3

7 1080 8 938 1 1

7 1081 16 862 1 5

7 1082 32 663 0 4

7 1083 16 621 0 2

7 1084 16 1206 1 4

7 1085 16 933 1 1

7 1086 8 647 1 3

7 1087 16 672 1 2

7 1088 32 756 1 3

7 1089 8 640 1 5

7 1090 8 998 1 1

7 1091 32 707 1 4

7 1092 32 761 1 5

7 1093 16 928 1 1

7 1094 16 806 0 4

7 1095 8 816 1 2

7 1096 8 790 1 3

7 1097 32 581 1 3

7 1098 16 861 0 3

7 1099 16 897 1 4

7 1100 32 1240 1 1

7 1101 16 861 1 2

7 1102 16 1054 1 1

7 1103 8 658 1 4

7 1104 16 795 0 4

7 1105 32 642 1 4

7 1106 32 881 1 2

7 1107 8 697 1 4

7 1108 32 841 1 2

7 1109 32 887 1 1

7 1110 8 1449 1 1

7 1111 32 826 1 3

7 1112 32 778 1 5

7 1113 8 685 1 2

7 1114 32 1297 1 5

7 1115 8 661 1 4

7 1116 16 1117 1 4

7 1117 8 596 0 4

7 1118 32 905 1 4

7 1119 32 1026 1 2

7 1120 8 631 1 5

7 1121 16 757 0 4

7 1122 32 804 1 2

7 1123 16 660 1 5

7 1124 16 868 1 3

7 1125 16 999 1 1

7 1126 8 679 0 3

7 1127 8 910 1 2

7 1128 32 1025 1 5

7 1129 8 682 1 5

7 1130 8 946 1 5

7 1131 32 793 1 1

7 1132 32 871 1 4

7 1133 8 793 1 3

7 1134 16 583 1 3

7 1135 16 1183 1 1

7 1136 16 1007 1 3

7 1137 16 1171 1 1

7 1138 8 818 1 2

7 1139 32 821 1 4

7 1140 8 1492 1 1

7 1141 32 962 1 1

7 1142 16 642 1 5

7 1143 16 649 1 3

7 1144 8 745 1 2

7 1145 32 1123 1 1

7 1146 16 1045 1 1

7 1147 16 759 1 2

7 1148 32 807 0 5

7 1149 16 856 0 1

7 1150 32 918 0 1

7 1151 8 754 1 3

7 1152 8 956 1 4

7 1153 16 837 0 5

7 1154 32 891 1 3

7 1155 8 603 1 4

7 1156 8 922 1 5

7 1157 8 1786 1 2

7 1158 32 1127 1 4

7 1159 8 744 1 3

7 1160 32 1263 1 1

7 1161 32 945 1 5

7 1162 32 1298 1 3

7 1163 32 604 1 3

7 1164 32 794 1 4

7 1165 16 1349 1 5

7 1166 8 861 1 3

7 1167 8 687 0 1

7 1168 32 714 1 2

7 1169 8 662 1 2

7 1170 8 754 1 5

7 1171 32 873 1 3

7 1172 8 901 0 4

7 1173 32 603 1 2

7 1174 16 692 1 2

7 1175 8 717 1 5

7 1176 8 893 1 1

7 1177 16 752 0 2

7 1178 8 558 1 2

7 1179 16 723 1 5

7 1180 16 674 0 2

7 1181 32 786 1 2

7 1182 16 682 1 5

7 1183 32 865 1 1

7 1184 8 733 1 5

7 1185 16 925 1 2

7 1186 32 946 1 5

7 1187 8 731 1 1

7 1188 16 1063 1 4

7 1189 8 1105 1 4

7 1190 32 836 1 5

7 1191 8 779 1 3

7 1192 16 821 1 3

7 1193 8 810 0 1

7 1194 16 788 1 5

7 1195 8 832 1 1

7 1196 16 943 1 4

7 1197 16 1040 1 3

7 1198 32 742 0 2

7 1199 32 1137 1 3

7 1200 16 707 1 3

8 1 32 982 0 4

8 2 32 858 1 5

8 3 8 942 1 4

8 4 8 1134 1 1

8 5 32 761 1 3

8 6 32 756 1 5

8 7 32 684 1 5

8 8 16 692 1 5

8 9 32 788 1 4

8 10 16 731 1 5

8 11 16 1258 1 1

8 12 8 1001 1 3

8 13 8 897 1 1

8 14 16 1541 1 2

8 15 32 757 1 1

8 16 32 598 1 2

8 17 16 625 0 3

8 18 32 697 1 2

8 19 8 642 1 2

8 20 8 1011 1 4

8 21 16 733 1 4

8 22 32 700 1 3

8 23 8 884 1 3

8 24 32 888 1 3

8 25 8 1323 0 2

8 26 8 646 1 3

8 27 8 871 1 5

8 28 16 1155 1 1

8 29 16 687 1 3

8 30 8 661 1 2

8 31 16 814 1 5

8 32 32 837 1 2

8 33 32 629 1 4

8 34 8 1329 1 5

8 35 16 899 1 1

8 36 8 731 1 3

8 37 16 922 1 5

8 38 8 814 1 1

8 39 8 645 1 4

8 40 32 1128 1 1

8 41 32 816 1 1

8 42 16 957 1 5

8 43 8 537 1 2

8 44 16 1344 1 4

8 45 8 674 1 5

8 46 16 663 0 3

8 47 8 897 1 1

8 48 16 514 1 2

8 49 8 820 0 1

8 50 16 716 1 4

8 51 32 889 1 5

8 52 16 1145 0 5

8 53 32 681 0 2

8 54 32 1148 1 3

8 55 16 1013 1 3

8 56 16 780 1 3

8 57 32 849 1 1

8 58 16 932 1 3

8 59 8 528 1 5

8 60 16 629 0 4

8 61 16 980 0 1

8 62 16 1164 1 1

8 63 16 1372 1 1

8 64 16 1088 0 2

8 65 32 1152 1 4

8 66 16 1164 1 5

8 67 32 1162 1 4

8 68 32 834 1 2

8 69 32 976 1 2

8 70 16 644 1 5

8 71 16 880 0 2

8 72 32 770 0 5

8 73 8 612 1 3

8 74 8 1430 1 2

8 75 32 1364 0 1

8 76 8 795 1 3

8 77 16 734 1 3

8 78 32 831 1 3

8 79 8 1078 1 4

8 80 8 623 1 5

8 81 8 1174 1 1

8 82 16 797 0 4

8 83 8 815 1 5

8 84 32 1076 1 4

8 85 16 1094 1 3

8 86 8 1005 1 2

8 87 8 1179 1 5

8 88 32 825 1 4

8 89 16 1030 1 4

8 90 8 645 1 3

8 91 32 842 1 3

8 92 32 733 1 1

8 93 32 852 1 2

8 94 8 977 1 1

8 95 16 703 0 1

8 96 16 1380 1 4

8 97 32 956 1 3

8 98 16 1251 1 2

8 99 8 1207 0 4

8 100 8 1095 1 2

8 101 32 540 1 3

8 102 16 1496 1 2

8 103 32 1567 1 5

8 104 8 866 1 3

8 105 16 860 1 4

8 106 16 761 1 2

8 107 8 1113 1 4

8 108 32 1064 1 1

8 109 32 675 1 4

8 110 32 666 1 1

8 111 32 801 1 2

8 112 16 764 0 2

8 113 8 970 0 1

8 114 8 943 1 2

8 115 32 899 1 5

8 116 16 1260 0 1

8 117 8 916 1 4

8 118 8 659 1 4

8 119 8 663 1 5

8 120 32 848 1 5

8 121 16 1051 0 3

8 122 32 801 0 4

8 123 16 761 1 3

8 124 16 1023 0 2

8 125 8 871 1 2

8 126 32 679 1 2

8 127 8 1177 1 4

8 128 8 720 1 3

8 129 32 1018 1 2

8 130 8 1107 1 5

8 131 8 1022 1 5

8 132 8 661 0 3

8 133 32 1228 1 3

8 134 8 1254 1 2

8 135 32 681 0 4

8 136 32 857 1 1

8 137 16 683 1 1

8 138 16 849 0 5

8 139 8 1347 1 5

8 140 8 606 1 5

8 141 32 694 1 4

8 142 32 693 1 5

8 143 8 972 1 2

8 144 32 1025 1 1

8 145 32 708 1 2

8 146 32 798 1 1

8 147 16 891 0 5

8 148 8 872 0 3

8 149 16 850 1 1

8 150 16 820 1 4

8 151 32 429 0 5

8 152 16 692 1 4

8 153 16 1377 1 4

8 154 16 1143 1 3

8 155 8 1053 1 4

8 156 32 683 1 1

8 157 16 677 1 5

8 158 16 559 0 2

8 159 32 974 1 3

8 160 16 1002 1 1

8 161 32 847 1 2

8 162 32 801 1 5

8 163 8 974 1 2

8 164 8 846 1 4

8 165 8 880 1 5

8 166 16 441 0 2

8 167 8 602 1 1

8 168 8 866 0 3

8 169 8 618 0 4

8 170 32 800 0 3

8 171 16 867 1 2

8 172 32 552 1 3

8 173 16 663 1 4

8 174 8 761 1 3

8 175 32 652 1 4

8 176 16 644 1 5

8 177 32 653 1 2

8 178 32 918 1 1

8 179 16 685 0 1

8 180 8 687 1 5

8 181 32 1069 0 5

8 182 32 830 0 1

8 183 16 583 1 5

8 184 32 554 0 5

8 185 16 498 1 3

8 186 32 1170 1 4

8 187 32 643 0 5

8 188 16 779 0 2

8 189 32 1093 1 5

8 190 8 995 1 3

8 191 16 745 0 5

8 192 8 626 1 3

8 193 16 525 0 3

8 194 8 1138 0 1

8 195 32 977 1 3

8 196 8 674 0 1

8 197 8 731 1 2

8 198 32 1152 1 5

8 199 8 1080 1 2

8 200 8 1189 1 5

8 201 16 809 0 4

8 202 32 874 1 2

8 203 16 818 1 1

8 204 32 624 1 2

8 205 16 1021 0 2

8 206 32 820 1 4

8 207 8 865 1 2

8 208 8 902 1 4

8 209 16 1279 1 4

8 210 16 1002 1 1

8 211 32 1027 0 2

8 212 8 1595 1 1

8 213 8 735 1 1

8 214 32 815 1 3

8 215 32 1284 0 3

8 216 32 1291 1 1

8 217 32 815 1 1

8 218 8 1054 1 2

8 219 16 823 0 2

8 220 32 868 1 3

8 221 32 1222 1 4

8 222 16 933 1 3

8 223 8 649 1 4

8 224 8 613 0 1

8 225 16 952 1 5

8 226 16 813 1 2

8 227 8 1209 0 1

8 228 16 1091 1 5

8 229 8 989 1 1

8 230 8 679 1 4

8 231 8 993 1 5

8 232 16 788 0 1

8 233 16 786 0 3

8 234 16 766 0 3

8 235 8 1136 0 4

8 236 16 1059 1 4

8 237 8 635 1 3

8 238 16 846 0 4

8 239 32 818 1 4

8 240 16 1368 0 1

8 241 32 861 1 1

8 242 32 791 1 3

8 243 8 859 1 1

8 244 16 828 1 5

8 245 8 1367 1 2

8 246 8 757 1 2

8 247 16 535 0 2

8 248 32 878 1 1

8 249 32 826 1 5

8 250 16 757 1 3

8 251 8 954 1 3

8 252 16 877 0 1

8 253 8 806 0 4

8 254 8 1428 1 1

8 255 8 610 1 4

8 256 16 587 1 2

8 257 16 1022 0 2

8 258 32 1130 1 4

8 259 8 788 1 4

8 260 16 1047 0 4

8 261 8 659 1 2

8 262 16 518 1 1

8 263 32 723 1 3

8 264 8 1114 1 3

8 265 8 719 1 4

8 266 16 651 1 5

8 267 8 1034 1 2

8 268 8 767 1 5

8 269 32 672 1 4

8 270 16 644 0 3

8 271 32 847 0 1

8 272 16 960 1 1

8 273 32 1125 1 3

8 274 32 678 1 2

8 275 32 786 1 5

8 276 32 759 1 2

8 277 32 1275 1 2

8 278 16 673 0 4

8 279 32 217 0 2

8 280 32 1048 1 4

8 281 8 844 1 2

8 282 32 936 1 1

8 283 8 731 1 3

8 284 8 1065 1 4

8 285 16 1206 1 5

8 286 16 1027 0 4

8 287 16 413 0 2

8 288 32 1082 1 4

8 289 32 690 1 2

8 290 16 674 1 5

8 291 16 822 0 1

8 292 32 755 1 1

8 293 8 1857 1 2

8 294 8 874 1 5

8 295 32 997 1 2

8 296 32 953 1 5

8 297 8 941 1 3

8 298 32 919 1 3

8 299 32 1017 0 3

8 300 32 846 1 2

8 301 8 635 0 1

8 302 8 732 1 5

8 303 16 938 0 3

8 304 16 1012 1 3

8 305 8 992 1 3

8 306 16 966 0 5

8 307 32 815 1 2

8 308 16 762 1 1

8 309 16 1381 1 4

8 310 16 719 1 1

8 311 8 668 1 5

8 312 32 863 1 1

8 313 16 865 1 4

8 314 16 777 1 3

8 315 16 741 1 4

8 316 8 704 1 3

8 317 8 539 1 5

8 318 8 501 1 4

8 319 16 560 0 2

8 320 32 912 1 3

8 321 8 1003 1 1

8 322 32 659 1 3

8 323 8 819 1 5

8 324 8 1241 1 3

8 325 32 1024 1 5

8 326 8 1207 1 5

8 327 32 655 1 5

8 328 32 561 1 5

8 329 8 791 1 2

8 330 16 694 0 5

8 331 16 737 1 3

8 332 8 957 1 1

8 333 8 547 1 1

8 334 8 497 0 4

8 335 8 575 1 4

8 336 8 754 1 2

8 337 16 727 0 2

8 338 8 584 1 5

8 339 16 532 0 3

8 340 32 1046 0 1

8 341 16 1258 1 4

8 342 32 781 1 3

8 343 32 791 1 5

8 344 32 703 1 4

8 345 32 705 1 1

8 346 32 915 1 4

8 347 8 1022 0 1

8 348 16 1143 0 1

8 349 16 743 1 3

8 350 32 1294 0 5

8 351 16 756 1 1

8 352 16 806 1 5

8 353 32 737 1 4

8 354 8 744 0 3

8 355 8 1457 1 1

8 356 16 2194 1 4

8 357 16 973 0 2

8 358 16 682 1 2

8 359 32 657 1 4

8 360 16 845 1 5

8 361 32 741 1 1

8 362 16 841 1 5

8 363 32 849 0 2

8 364 16 102 1 5

8 365 8 1163 1 2

8 366 16 649 0 2

8 367 8 810 1 4

8 368 8 1280 1 3

8 369 32 735 1 4

8 370 16 682 0 4

8 371 8 940 1 4

8 372 16 719 0 4

8 373 16 533 0 3

8 374 16 1248 1 5

8 375 8 596 1 5

8 376 32 1164 1 5

8 377 32 748 1 3

8 378 8 895 0 1

8 379 32 810 1 5

8 380 16 611 1 3

8 381 32 746 1 4

8 382 8 1021 1 4

8 383 32 1348 1 4

8 384 32 682 1 3

8 385 16 769 1 2

8 386 16 743 1 1

8 387 32 874 1 5

8 388 16 607 0 4

8 389 32 632 1 4

8 390 16 852 1 5

8 391 8 639 1 4

8 392 16 617 1 4

8 393 16 868 1 2

8 394 32 1007 1 5

8 395 32 967 1 3

8 396 16 750 1 1

8 397 32 787 0 2

8 398 32 729 1 1

8 399 32 616 0 4

8 400 16 868 0 4

8 401 8 866 0 2

8 402 16 750 0 4

8 403 16 608 1 5

8 404 8 870 1 1

8 405 8 844 1 2

8 406 32 826 1 2

8 407 8 691 1 4

8 408 8 620 1 4

8 409 8 694 1 3

8 410 32 649 1 1

8 411 16 816 0 2

8 412 16 853 1 1

8 413 32 831 1 3

8 414 16 810 1 4

8 415 16 697 1 3

8 416 32 683 1 2

8 417 16 859 1 3

8 418 8 1169 1 1

8 419 8 1095 0 1

8 420 16 834 0 2

8 421 8 1000 0 3

8 422 16 1278 1 5

8 423 16 1043 0 1

8 424 32 1179 1 3

8 425 16 637 1 1

8 426 32 860 1 2

8 427 16 1131 0 3

8 428 16 1036 1 2

8 429 32 1071 1 2

8 430 32 1144 1 3

8 431 8 834 1 3

8 432 16 679 1 1

8 433 8 1029 1 5

8 434 8 866 0 5

8 435 32 878 0 4

8 436 32 1244 0 1

8 437 8 742 0 5

8 438 8 721 1 5

8 439 32 650 1 3

8 440 16 594 0 5

8 441 32 1227 1 5

8 442 32 1006 1 2

8 443 8 1355 1 2

8 444 8 1093 1 1

8 445 16 716 0 3

8 446 16 1638 1 5

8 447 16 860 1 4

8 448 16 868 0 2

8 449 16 954 0 2

8 450 16 1548 1 3

8 451 32 1073 0 2

8 452 32 902 0 5

8 453 8 856 1 2

8 454 8 573 1 3

8 455 8 930 1 1

8 456 8 1897 1 1

8 457 32 987 1 1

8 458 8 665 1 5

8 459 8 643 1 4

8 460 32 627 1 1

8 461 8 832 1 4

8 462 32 1386 0 1

8 463 16 1333 0 1

8 464 8 799 1 2

8 465 16 1092 1 3

8 466 8 1074 1 5

8 467 8 604 1 5

8 468 8 529 1 3

8 469 32 681 1 4

8 470 8 590 0 3

8 471 8 661 1 3

8 472 32 920 1 3

8 473 8 783 1 2

8 474 32 813 1 5

8 475 32 875 0 5

8 476 8 1175 1 1

8 477 8 1152 1 2

8 478 16 750 1 1

8 479 32 758 0 4

8 480 32 1570 1 1

8 481 16 621 0 2

8 482 32 632 1 5

8 483 8 775 1 3

8 484 8 1107 1 1

8 485 16 729 1 5

8 486 16 1018 1 4

8 487 8 699 1 5

8 488 16 1374 1 4

8 489 16 890 0 1

8 490 16 838 0 1

8 491 16 1215 1 2

8 492 8 682 1 5

8 493 8 1261 1 1

8 494 16 1082 0 4

8 495 8 599 1 4

8 496 16 745 1 3

8 497 16 717 1 1

8 498 8 693 1 4

8 499 8 572 0 4

8 500 32 1343 0 5

8 501 8 1125 0 2

8 502 8 939 1 5

8 503 16 1141 0 2

8 504 16 1482 0 2

8 505 16 628 1 5

8 506 16 1214 1 5

8 507 16 1068 0 4

8 508 16 872 1 4

8 509 8 808 1 1

8 510 32 1041 0 1

8 511 32 1086 1 2

8 512 32 832 1 2

8 513 16 1344 1 4

8 514 16 1061 1 3

8 515 32 885 1 3

8 516 32 1152 0 2

8 517 32 726 1 3

8 518 32 1001 1 3

8 519 32 1171 1 1

8 520 8 624 1 1

8 521 32 655 1 2

8 522 8 878 1 1

8 523 8 563 0 4

8 524 16 921 1 2

8 525 8 630 1 3

8 526 32 732 1 2

8 527 8 797 1 4

8 528 32 828 0 1

8 529 32 846 1 3

8 530 32 1563 1 5

8 531 16 1206 1 3

8 532 8 648 1 4

8 533 32 797 1 5

8 534 8 1169 0 4

8 535 32 1340 1 2

8 536 8 1523 1 3

8 537 16 1129 1 5

8 538 8 1274 1 1

8 539 8 817 1 5

8 540 32 1007 1 4

8 541 8 717 1 5

8 542 16 965 1 1

8 543 16 766 0 2

8 544 16 570 1 5

8 545 32 1556 0 3

8 546 8 1588 1 2

8 547 32 1032 1 2

8 548 16 875 1 5

8 549 16 1440 1 1

8 550 8 1152 1 3

8 551 32 693 1 4

8 552 8 716 1 2

8 553 16 633 1 5

8 554 16 622 0 1

8 555 16 1413 1 2

8 556 8 1033 1 5

8 557 8 930 1 2

8 558 16 576 0 3

8 559 8 1057 1 5

8 560 32 1065 0 3

8 561 32 817 1 4

8 562 32 1060 1 5

8 563 32 885 1 5

8 564 32 805 0 5

8 565 32 1429 1 4

8 566 32 821 1 2

8 567 16 1283 1 3

8 568 32 1259 1 4

8 569 16 911 1 5

8 570 16 1455 0 1

8 571 16 1062 0 2

8 572 8 1326 1 2

8 573 32 1357 1 1

8 574 16 805 0 3

8 575 8 633 1 2

8 576 16 1188 1 1

8 577 8 889 0 5

8 578 16 1386 1 4

8 579 8 1612 0 1

8 580 16 1378 1 4

8 581 32 814 1 1

8 582 32 646 1 1

8 583 8 868 1 3

8 584 32 639 0 1

8 585 32 665 1 4

8 586 32 619 1 1

8 587 8 739 0 2

8 588 16 1115 1 3

8 589 8 919 1 1

8 590 32 714 1 3

8 591 8 431 0 2

8 592 32 800 1 5

8 593 8 741 1 3

8 594 16 811 1 3

8 595 32 700 1 4

8 596 8 1473 1 3

8 597 32 946 1 3

8 598 8 808 1 3

8 599 8 1032 1 4

8 600 32 740 1 4

8 601 8 1380 1 1

8 602 16 1131 1 2

8 603 8 739 1 2

8 604 32 764 1 3

8 605 8 669 1 3

8 606 16 722 1 5

8 607 8 1049 1 1

8 608 8 934 1 2

8 609 32 946 1 5

8 610 8 751 1 1

8 611 32 1002 1 1

8 612 8 699 1 5

8 613 8 981 1 4

8 614 8 908 1 2

8 615 16 1008 1 3

8 616 16 640 1 2

8 617 32 719 1 5

8 618 32 733 1 2

8 619 8 773 1 3

8 620 8 1167 1 1

8 621 32 885 1 5

8 622 8 996 1 4

8 623 32 1125 1 3

8 624 8 788 1 5

8 625 32 1176 1 3

8 626 16 697 1 4

8 627 32 715 1 3

8 628 16 1238 1 1

8 629 16 1090 1 4

8 630 16 584 0 2

8 631 16 835 0 2

8 632 8 835 1 4

8 633 8 617 1 5

8 634 16 938 0 2

8 635 32 704 1 4

8 636 32 730 0 4

8 637 32 995 0 2

8 638 8 634 1 4

8 639 16 703 0 3

8 640 16 876 1 3

8 641 8 1080 1 5

8 642 32 823 1 2

8 643 16 626 1 5

8 644 16 1559 1 4

8 645 16 1448 0 4

8 646 8 1119 1 4

8 647 8 913 1 3

8 648 8 853 1 1

8 649 32 717 0 3

8 650 16 1170 1 1

8 651 16 547 1 5

8 652 16 417 1 3

8 653 16 614 1 4

8 654 16 1151 1 4

8 655 16 635 1 5

8 656 8 753 1 5

8 657 32 815 1 1

8 658 32 759 1 5

8 659 32 670 1 4

8 660 32 787 1 5

8 661 8 870 0 1

8 662 8 813 0 4

8 663 32 759 0 5

8 664 32 1663 1 4

8 665 8 871 1 2

8 666 8 779 1 4

8 667 32 1037 0 5

8 668 32 867 1 5

8 669 16 1179 1 1

8 670 32 750 1 1

8 671 8 1137 1 1

8 672 32 690 1 2

8 673 8 857 1 3

8 674 32 690 1 2

8 675 8 641 0 4

8 676 16 509 1 1

8 677 16 586 0 2

8 678 32 728 1 4

8 679 8 705 0 2

8 680 32 1253 1 4

8 681 32 660 1 3

8 682 8 1043 1 3

8 683 8 613 1 5

8 684 16 616 1 2

8 685 32 445 0 1

8 686 8 1268 0 2

8 687 16 1196 1 3

8 688 16 945 1 2

8 689 32 1352 1 1

8 690 32 917 1 1

8 691 32 720 1 4

8 692 16 878 1 5

8 693 32 938 1 1

8 694 32 716 1 4

8 695 32 799 1 2

8 696 16 725 1 4

8 697 8 764 1 5

8 698 32 668 0 2

8 699 32 1233 1 3

8 700 8 684 1 2

8 701 8 707 1 3

8 702 16 1007 1 5

8 703 16 1156 1 3

8 704 16 1183 0 4

8 705 16 678 0 3

8 706 8 1505 1 5

8 707 8 1528 0 3

8 708 16 1375 1 1

8 709 16 739 1 1

8 710 16 755 1 5

8 711 16 532 0 1

8 712 32 748 1 1

8 713 32 1072 1 2

8 714 16 814 1 1

8 715 32 640 1 3

8 716 8 530 1 3

8 717 8 753 0 1

8 718 16 935 0 5

8 719 16 1733 1 3

8 720 8 1836 0 2

8 721 16 985 1 5

8 722 32 954 1 4

8 723 8 864 0 3

8 724 8 1488 1 1

8 725 32 719 1 2

8 726 32 842 1 3

8 727 16 801 1 5

8 728 16 735 1 3

8 729 32 1304 1 5

8 730 16 698 1 5

8 731 8 939 1 2

8 732 32 1203 1 1

8 733 8 643 1 5

8 734 16 619 0 2

8 735 16 736 1 1

8 736 32 960 1 2

8 737 16 1191 1 3

8 738 16 2431 1 4

8 739 8 812 1 1

8 740 8 694 1 4

8 741 32 682 1 5

8 742 8 705 1 2

8 743 8 1186 0 1

8 744 32 530 1 1

8 745 32 1581 1 2

8 746 8 601 1 3

8 747 8 466 1 2

8 748 8 591 1 5

8 749 8 1029 1 2

8 750 8 1230 0 1

8 751 32 884 1 3

8 752 32 845 1 5

8 753 32 1042 1 3

8 754 8 791 1 5

8 755 8 588 1 5

8 756 32 695 1 5

8 757 32 642 1 5

8 758 8 553 0 3

8 759 8 928 1 5

8 760 8 695 1 2

8 761 32 612 0 4

8 762 32 590 1 2

8 763 32 804 1 1

8 764 32 1119 0 1

8 765 16 669 1 5

8 766 16 612 1 1

8 767 8 843 0 5

8 768 16 677 1 1

8 769 32 682 1 3

8 770 16 760 1 2

8 771 32 1017 1 4

8 772 8 1789 1 2

8 773 32 922 1 1

8 774 32 1160 0 2

8 775 8 890 1 3

8 776 16 692 1 4

8 777 32 993 0 5

8 778 8 1111 1 4

8 779 32 731 1 4

8 780 8 754 0 5

8 781 32 960 1 1

8 782 8 649 0 1

8 783 32 1162 1 2

8 784 8 1828 1 3

8 785 8 973 1 3

8 786 8 968 1 4

8 787 32 1293 1 3

8 788 32 872 1 1

8 789 8 792 1 4

8 790 8 1152 1 1

8 791 16 599 1 3

8 792 16 723 1 4

8 793 32 610 1 3

8 794 32 1153 0 4

8 795 16 854 0 2

8 796 8 850 1 4

8 797 8 943 1 3

8 798 16 620 1 2

8 799 16 654 1 1

8 800 32 791 0 3

8 801 16 1143 1 4

8 802 16 1993 1 2

8 803 16 1306 1 5

8 804 8 1018 1 1

8 805 8 1026 1 2

8 806 16 1798 0 1

8 807 32 827 1 2

8 808 16 2337 1 3

8 809 16 1871 1 4

8 810 32 795 1 4

8 811 16 1174 0 5

8 812 16 1312 1 2

8 813 32 780 1 3

8 814 16 702 1 4

8 815 16 690 0 2

8 816 16 699 1 1

8 817 16 686 1 3

8 818 16 781 0 1

8 819 8 1613 1 4

8 820 8 817 1 3

8 821 8 858 1 5

8 822 16 1090 1 4

8 823 16 850 1 2

8 824 8 665 1 4

8 825 32 1232 1 5

8 826 8 1106 1 4

8 827 32 862 1 5

8 828 16 672 1 5

8 829 8 1856 1 2

8 830 16 860 1 5

8 831 16 740 0 1

8 832 32 673 0 4

8 833 16 1008 1 3

8 834 32 990 1 1

8 835 8 820 1 1

8 836 32 702 0 2

8 837 16 483 0 3

8 838 16 1267 1 3

8 839 16 719 1 4

8 840 32 806 0 4

8 841 32 696 1 4

8 842 8 1201 1 3

8 843 16 971 1 5

8 844 16 880 1 3

8 845 16 895 0 4

8 846 16 1179 1 4

8 847 16 896 0 2

8 848 8 924 1 4

8 849 32 987 1 4

8 850 8 718 1 5

8 851 8 691 1 5

8 852 8 739 0 4

8 853 8 1064 1 1

8 854 8 546 1 3

8 855 32 1046 0 2

8 856 8 1133 0 1

8 857 32 746 1 3

8 858 16 766 0 2

8 859 8 1219 1 3

8 860 8 575 1 2

8 861 8 825 1 4

8 862 8 2779 1 4

8 863 8 983 1 5

8 864 8 1239 1 1

8 865 32 724 0 4

8 866 8 832 1 2

8 867 32 700 1 2

8 868 16 615 1 2

8 869 8 944 1 1

8 870 32 761 1 5

8 871 16 737 1 5

8 872 32 563 1 1

8 873 8 451 1 5

8 874 8 612 1 4

8 875 8 707 0 1

8 876 16 1017 1 4

8 877 16 611 1 3

8 878 32 446 1 2

8 879 32 414 1 5

8 880 16 656 0 4

8 881 32 875 1 1

8 882 32 783 1 3

8 883 32 620 1 5

8 884 32 790 0 2

8 885 8 869 1 5

8 886 16 867 0 4

8 887 8 706 1 4

8 888 8 981 1 3

8 889 8 1268 1 3

8 890 32 1427 1 1

8 891 8 673 1 2

8 892 32 647 0 3

8 893 16 902 1 2

8 894 8 775 1 5

8 895 32 1026 1 5

8 896 16 644 1 3

8 897 32 1263 1 4

8 898 32 595 1 2

8 899 16 504 0 2

8 900 32 1394 1 2

8 901 8 1046 1 3

8 902 16 524 1 1

8 903 32 446 0 5

8 904 32 626 1 1

8 905 16 492 1 2

8 906 32 608 0 5

8 907 16 516 0 5

8 908 16 755 1 1

8 909 32 447 1 1

8 910 32 625 1 4

8 911 16 558 0 2

8 912 8 709 1 2

8 913 16 740 0 5

8 914 32 1266 1 4

8 915 8 851 0 5

8 916 32 1211 1 3

8 917 32 820 1 3

8 918 32 555 0 3

8 919 16 780 1 1

8 920 16 1342 1 4

8 921 16 984 0 1

8 922 16 835 1 4

8 923 8 800 1 5

8 924 8 1011 0 1

8 925 16 1002 1 3

8 926 8 710 1 4

8 927 32 844 1 4

8 928 8 1238 1 1

8 929 16 713 0 4

8 930 16 620 0 1

8 931 16 889 0 1

8 932 16 1026 1 3

8 933 32 1352 1 1

8 934 16 667 1 3

8 935 16 705 1 5

8 936 16 592 1 3

8 937 8 529 1 4

8 938 32 717 1 4

8 939 16 603 1 5

8 940 32 513 1 2

8 941 8 438 1 2

8 942 16 334 0 5

8 943 16 866 1 5

8 944 32 598 1 2

8 945 8 545 1 3

8 946 32 736 1 3

8 947 32 721 1 5

8 948 32 676 1 1

8 949 8 496 1 2

8 950 32 928 1 5

8 951 32 1068 1 3

8 952 16 710 1 3

8 953 8 822 1 3

8 954 16 862 0 1

8 955 16 669 1 2

8 956 8 968 1 1

8 957 8 358 1 2

8 958 16 623 1 1

8 959 32 501 1 1

8 960 8 460 0 2

8 961 32 862 1 4

8 962 16 1532 1 1

8 963 32 1046 1 4

8 964 8 923 0 1

8 965 16 1090 1 5

8 966 16 1182 0 2

8 967 8 694 1 2

8 968 32 826 0 1

8 969 16 604 1 5

8 970 16 637 1 5

8 971 32 851 1 5

8 972 8 658 1 4

8 973 32 718 1 1

8 974 32 831 1 1

8 975 32 684 1 5

8 976 32 1071 1 1

8 977 16 543 0 1

8 978 32 664 1 3

8 979 8 740 1 3

8 980 16 738 0 2

8 981 16 509 0 3

8 982 16 1083 1 3

8 983 8 700 1 5

8 984 16 698 1 5

8 985 32 967 1 5

8 986 16 630 1 2

8 987 16 634 1 3

8 988 8 500 1 3

8 989 8 737 0 4

8 990 32 1461 0 2

8 991 32 410 1 2

8 992 16 723 0 5

8 993 16 639 0 1

8 994 32 861 1 5

8 995 8 994 1 3

8 996 32 1073 1 4

8 997 8 1483 1 1

8 998 8 741 1 3

8 999 8 1160 1 4

8 1000 8 810 1 4

8 1001 16 685 1 3

8 1002 8 916 1 5

8 1003 32 622 0 3

8 1004 32 574 1 2

8 1005 8 537 0 3

8 1006 32 1195 1 4

8 1007 8 830 1 2

8 1008 16 844 1 4

8 1009 32 761 1 4

8 1010 8 873 0 2

8 1011 16 958 1 4

8 1012 16 538 1 2

8 1013 16 590 1 3

8 1014 16 685 1 4

8 1015 32 964 1 1

8 1016 16 826 0 3

8 1017 16 766 1 4

8 1018 32 662 0 4

8 1019 32 787 1 3

8 1020 16 1134 0 1

8 1021 32 850 0 3

8 1022 16 1280 1 4

8 1023 16 1429 1 3

8 1024 16 719 0 1

8 1025 32 970 1 5

8 1026 32 766 1 2

8 1027 32 938 1 2

8 1028 8 1893 1 1

8 1029 16 816 1 5

8 1030 8 708 0 5

8 1031 16 900 0 1

8 1032 8 937 1 2

8 1033 16 979 1 4

8 1034 8 926 1 1

8 1035 8 941 1 5

8 1036 16 544 0 5

8 1037 8 801 0 5

8 1038 32 1042 1 1

8 1039 16 815 0 2

8 1040 8 1120 1 3

8 1041 8 934 1 4

8 1042 32 914 1 4

8 1043 32 1074 0 2

8 1044 8 815 0 4

8 1045 32 988 1 5

8 1046 32 1387 1 3

8 1047 16 885 0 2

8 1048 32 778 1 3

8 1049 32 780 0 1

8 1050 8 961 1 5

8 1051 32 929 1 5

8 1052 32 633 1 4

8 1053 32 1063 1 1

8 1054 32 904 1 3

8 1055 8 930 1 3

8 1056 8 1924 1 1

8 1057 16 1269 1 1

8 1058 8 732 1 2

8 1059 8 919 1 4

8 1060 16 840 1 4

8 1061 32 738 1 3

8 1062 8 1159 1 1

8 1063 16 712 0 5

8 1064 8 831 0 2

8 1065 16 1627 1 3

8 1066 8 1057 0 5

8 1067 8 751 1 2

8 1068 8 907 0 1

8 1069 32 1218 1 5

8 1070 8 603 1 3

8 1071 16 854 1 1

8 1072 16 699 1 2

8 1073 16 1417 0 4

8 1074 8 727 1 5

8 1075 32 854 1 2

8 1076 8 1099 1 2

8 1077 8 709 1 4

8 1078 8 803 1 1

8 1079 16 696 1 2

8 1080 32 1098 1 2

8 1081 32 772 1 2

8 1082 8 581 1 3

8 1083 16 793 0 2

8 1084 32 1175 0 5

8 1085 16 1058 0 5

8 1086 8 647 1 5

8 1087 8 807 1 4

8 1088 32 1235 1 2

8 1089 32 1030 1 1

8 1090 8 781 0 2

8 1091 8 770 1 4

8 1092 16 758 0 2

8 1093 32 789 1 4

8 1094 32 772 1 4

8 1095 32 755 0 5

8 1096 8 794 0 4

8 1097 32 746 1 3

8 1098 8 1014 1 2

8 1099 8 775 1 5

8 1100 32 851 1 1

8 1101 16 764 1 4

8 1102 8 778 0 5

8 1103 16 727 0 4

8 1104 32 847 1 4

8 1105 16 712 1 2

8 1106 32 760 1 3

8 1107 16 625 1 4

8 1108 8 682 1 3

8 1109 32 1002 1 2

8 1110 16 673 0 5

8 1111 8 1286 1 2

8 1112 32 662 1 2

8 1113 32 863 1 3

8 1114 8 737 0 2

8 1115 8 685 1 1

8 1116 16 727 1 5

8 1117 16 561 0 1

8 1118 8 969 1 2

8 1119 8 792 1 5

8 1120 32 871 1 5

8 1121 8 1116 1 4

8 1122 16 561 1 5

8 1123 16 1286 1 5

8 1124 16 932 1 3

8 1125 16 1564 0 4

8 1126 8 886 1 3

8 1127 32 1008 1 4

8 1128 8 910 1 4

8 1129 8 1133 1 5

8 1130 8 791 1 3

8 1131 32 1376 1 1

8 1132 8 1424 1 1

8 1133 16 828 1 1

8 1134 32 859 1 5

8 1135 32 934 1 1

8 1136 32 784 1 1

8 1137 16 922 0 2

8 1138 16 770 0 1

8 1139 8 1315 1 1

8 1140 8 857 1 2

8 1141 8 669 1 1

8 1142 16 611 0 3

8 1143 16 812 0 2

8 1144 32 829 1 5

8 1145 16 842 1 3

8 1146 16 896 1 5

8 1147 8 931 0 5

8 1148 8 1069 1 2

8 1149 16 927 0 3

8 1150 32 881 1 2

8 1151 16 1317 1 2

8 1152 8 778 0 1

8 1153 16 928 1 4

8 1154 32 810 1 5

8 1155 8 708 1 4

8 1156 16 737 0 1

8 1157 8 1013 1 1

8 1158 16 592 1 5

8 1159 16 609 1 3

8 1160 32 981 1 5

8 1161 16 1000 0 1

8 1162 8 970 1 5

8 1163 16 914 1 5

8 1164 16 1225 1 1

8 1165 8 726 0 4

8 1166 16 646 0 2

8 1167 32 774 1 4

8 1168 32 838 1 4

8 1169 32 1220 1 1

8 1170 32 789 1 2

8 1171 8 746 1 4

8 1172 8 762 1 3

8 1173 16 652 1 4

8 1174 16 444 1 1

8 1175 16 706 1 4

8 1176 16 883 1 3

8 1177 8 1335 1 2

8 1178 32 648 1 4

8 1179 16 785 1 2

8 1180 8 690 0 1

8 1181 16 1098 1 4

8 1182 32 808 1 2

8 1183 32 551 1 3

8 1184 8 697 1 3

8 1185 32 906 1 3

8 1186 16 1275 1 1

8 1187 16 611 1 3

8 1188 16 731 1 3

8 1189 32 586 1 3

8 1190 32 603 1 4

8 1191 32 617 1 1

8 1192 32 623 1 3

8 1193 32 754 1 5

8 1194 8 1133 1 3

8 1195 32 872 1 3

8 1196 8 830 0 3

8 1197 8 1021 0 1

8 1198 32 792 1 1

8 1199 32 631 1 2

8 1200 8 859 1 5

9 1 8 878 1 2

9 2 16 768 1 5

9 3 16 1044 1 2

9 4 32 1418 1 5

9 5 32 1686 1 1

9 6 8 1510 1 1

9 7 16 2038 1 4

9 8 32 1097 1 4

9 9 16 1719 1 1

9 10 16 1352 1 4

9 11 32 1071 1 1

9 12 8 854 1 3

9 13 8 1236 1 2

9 14 32 1459 1 3

9 15 16 1083 1 5

9 16 32 1649 1 1

9 17 16 1873 1 2

9 18 8 1458 1 4

9 19 16 1303 1 3

9 20 16 1020 1 5

9 21 16 1054 1 3

9 22 16 1329 0 2

9 23 16 1680 1 4

9 24 16 1137 1 1

9 25 32 1109 1 1

9 26 8 1905 1 1

9 27 32 1873 1 4

9 28 16 1692 1 1

9 29 8 1698 1 1

9 30 16 3091 1 3

9 31 16 2500 0 1

9 32 8 1297 1 5

9 33 32 1350 1 3

9 34 32 1331 1 4

9 35 16 1600 1 2

9 36 8 1396 1 4

9 37 8 1998 0 1

9 38 32 1894 1 4

9 39 16 4450 1 4

9 40 16 1635 1 3

9 41 32 1581 1 3

9 42 32 2035 1 4

9 43 32 1625 1 5

9 44 16 1683 1 3

9 45 16 1638 0 2

9 46 16 3103 0 2

9 47 16 1751 1 5

9 48 32 2413 1 5

9 49 32 1773 1 2

9 50 8 2444 1 1

9 51 8 1286 1 3

9 52 8 1593 1 5

9 53 32 1740 1 5

9 54 32 1772 1 4

9 55 32 2028 1 2

9 56 16 3872 1 1

9 57 8 1220 1 5

9 58 8 2714 1 1

9 59 32 1918 1 2

9 60 8 1558 0 3

9 61 16 1739 1 3

9 62 16 2465 0 1

9 63 32 1814 1 1

9 64 8 3192 1 1

9 65 8 1594 1 4

9 66 8 1803 1 3

9 67 16 2066 1 2

9 68 16 1996 1 5

9 69 32 2079 1 1

9 70 32 1870 1 3

9 71 16 2787 1 4

9 72 16 1558 1 5

9 73 8 1625 1 4

9 74 32 1309 1 3

9 75 32 1196 1 1

9 76 8 1930 1 1

9 77 8 1672 1 2

9 78 8 1573 0 2

9 79 8 4913 1 2

9 80 32 1314 1 3

9 81 32 1733 1 1

9 82 8 2659 1 3

9 83 8 1325 1 5

9 84 8 845 1 5

9 85 16 2253 1 4

9 86 16 1289 1 3

9 87 16 937 1 5

9 88 32 1575 1 5

9 89 16 2412 1 3

9 90 16 3395 0 2

9 91 8 1232 1 5

9 92 32 1493 1 4

9 93 8 1316 1 2

9 94 32 1757 1 2

9 95 16 3116 1 4

9 96 32 1727 1 2

9 97 8 1912 1 2

9 98 32 2413 0 5

9 99 8 3137 1 3

9 100 8 1234 1 5

9 101 8 1681 1 3

9 102 16 2155 1 4

9 103 32 1774 1 2

9 104 8 1992 1 4

9 105 8 1730 1 4

9 106 32 997 1 3

9 107 8 1501 1 2

9 108 32 1066 1 5

9 109 32 1372 1 5

9 110 16 1309 1 1

9 111 16 2079 1 1

9 112 8 1378 1 4

9 113 32 1479 1 3

9 114 32 998 1 2

9 115 8 1078 1 4

9 116 8 1165 1 5

9 117 16 1521 1 5

9 118 8 1342 1 3

9 119 32 3105 1 2

9 120 32 1789 1 4

9 121 8 974 1 4

9 122 8 1773 1 5

9 123 32 1395 1 1

9 124 32 792 1 2

9 125 8 804 1 3

9 126 16 1123 1 3

9 127 16 801 1 3

9 128 16 1510 1 4

9 129 16 1384 1 3

9 130 8 2785 1 1

9 131 16 1438 1 1

9 132 16 2307 0 4

9 133 16 1772 0 2

9 134 32 1661 0 5

9 135 32 864 1 2

9 136 32 2680 0 5

9 137 8 1039 1 4

9 138 8 2240 1 4

9 139 16 2671 1 5

9 140 32 1114 1 1

9 141 8 3360 1 1

9 142 32 2104 1 4

9 143 32 2124 1 5

9 144 32 1098 1 1

9 145 8 1257 1 1

9 146 8 1017 1 4

9 147 16 2156 0 1

9 148 16 2109 1 5

9 149 32 1668 1 3

9 150 8 2076 0 1

9 151 16 2584 1 4

9 152 32 1830 1 2

9 153 8 1215 1 4

9 154 8 1906 1 1

9 155 8 1197 1 2

9 156 8 899 1 3

9 157 32 1194 1 2

9 158 8 1285 1 5

9 159 16 3605 0 1

9 160 16 2771 1 2

9 161 32 1434 1 2

9 162 8 800 1 2

9 163 8 1132 1 4

9 164 16 1582 1 5

9 165 8 1207 1 3

9 166 32 1818 1 5

9 167 32 2052 1 1

9 168 8 2737 0 1

9 169 16 1904 1 4

9 170 32 1407 1 3

9 171 32 2571 1 3

9 172 16 12378 1 1

9 173 32 2698 1 5

9 174 16 1544 1 3

9 175 32 581 0 2

9 176 32 3003 1 4

9 177 16 2004 1 5

9 178 8 1291 1 2

9 179 32 1716 1 1

9 180 32 2948 1 2

9 181 32 1190 1 4

9 182 32 1160 1 1

9 183 8 1754 1 1

9 184 16 1521 0 2

9 185 8 939 1 5

9 186 16 1633 1 3

9 187 8 971 1 5

9 188 32 931 1 3

9 189 16 2147 1 3

9 190 32 1279 1 2

9 191 16 1712 1 1

9 192 8 893 1 5

9 193 8 1443 0 3

9 194 16 1858 1 1

9 195 16 1869 1 5

9 196 16 1986 0 2

9 197 16 2189 1 4

9 198 32 1398 1 3

9 199 16 1364 1 3

9 200 16 2006 0 2

9 201 16 1764 1 4

9 202 8 1104 1 3

9 203 32 1155 1 4

9 204 8 1002 1 4

9 205 32 1704 1 3

9 206 8 1137 1 5

9 207 8 1072 1 1

9 208 8 2084 1 2

9 209 16 1652 1 4

9 210 8 1504 1 4

9 211 8 1079 1 3

9 212 16 1667 1 1

9 213 8 1276 1 3

9 214 8 891 1 5

9 215 16 1607 1 4

9 216 8 1734 1 2

9 217 16 1369 1 2

9 218 32 1160 1 4

9 219 16 1463 1 1

9 220 16 925 1 5

9 221 16 1606 1 3

9 222 32 1981 1 4

9 223 8 1283 1 2

9 224 8 2064 1 2

9 225 32 1921 1 4

9 226 16 1696 0 2

9 227 16 1261 1 2

9 228 32 1255 1 4

9 229 32 1667 1 3

9 230 32 1889 1 5

9 231 32 1481 1 5

9 232 8 3288 1 2

9 233 16 1292 1 5

9 234 32 2899 1 1

9 235 8 1089 1 3

9 236 32 2386 1 1

9 237 32 1834 1 5

9 238 8 1923 1 5

9 239 32 1053 1 3

9 240 16 1125 1 5

9 241 16 2303 1 4

9 242 8 1040 1 5

9 243 8 1080 1 3

9 244 32 1015 1 2

9 245 32 1050 1 4

9 246 8 1243 1 2

9 247 16 1020 1 3

9 248 16 1522 1 2

9 249 32 1079 1 1

9 250 16 717 1 5

9 251 16 1502 1 1

9 252 16 1352 1 1

9 253 16 829 1 5

9 254 8 1589 0 1

9 255 32 1052 1 4

9 256 8 1276 1 5

9 257 8 917 1 3

9 258 32 13 1 5

9 259 16 782 1 3

9 260 8 976 1 2

9 261 8 1319 1 5

9 262 8 996 1 4

9 263 32 1577 0 4

9 264 8 794 1 5

9 265 16 807 1 5

9 266 16 1203 1 4

9 267 32 1138 1 3

9 268 32 976 1 2

9 269 16 1033 1 2

9 270 32 1170 1 5

9 271 32 1031 1 4

9 272 8 752 1 5

9 273 8 2882 1 4

9 274 16 1944 1 1

9 275 8 859 1 3

9 276 8 838 1 5

9 277 8 1195 1 4

9 278 32 1365 1 4

9 279 8 907 1 3

9 280 8 776 1 4

9 281 8 1287 1 2

9 282 32 764 1 3

9 283 32 889 1 3

9 284 16 1450 1 3

9 285 8 1214 1 2

9 286 32 1483 1 2

9 287 8 2098 1 1

9 288 16 1395 1 5

9 289 16 1924 1 3

9 290 8 2256 1 3

9 291 32 1526 1 5

9 292 8 1259 1 1

9 293 32 1714 1 1

9 294 16 1290 1 2

9 295 32 980 1 2

9 296 8 1854 1 2

9 297 16 1211 1 3

9 298 32 1557 1 4

9 299 8 1015 1 3

9 300 16 1181 0 2

9 301 16 1381 1 4

9 302 8 1129 1 1

9 303 8 1550 0 1

9 304 16 1040 1 1

9 305 16 785 0 1

9 306 8 878 1 5

9 307 32 709 1 2

9 308 32 640 1 1

9 309 16 781 1 5

9 310 8 759 1 3

9 311 8 772 1 2

9 312 32 973 1 3

9 313 32 739 1 3

9 314 32 1492 1 5

9 315 16 1098 0 2

9 316 16 778 1 5

9 317 8 832 1 1

9 318 16 1028 1 5

9 319 32 946 0 5

9 320 32 1359 0 5

9 321 16 1541 1 2

9 322 16 1241 1 4

9 323 32 739 1 1

9 324 32 879 1 1

9 325 8 1110 1 1

9 326 16 1718 0 4

9 327 16 1480 0 4

9 328 32 872 0 5

9 329 8 847 0 5

9 330 16 1024 1 4

9 331 8 1374 1 2

9 332 16 1692 1 1

9 333 8 1003 1 4

9 334 16 1477 1 3

9 335 32 1490 1 4

9 336 16 1694 1 5

9 337 32 1087 1 3

9 338 16 2190 1 4

9 339 32 1705 1 1

9 340 8 1624 1 4

9 341 32 998 1 1

9 342 32 1293 1 2

9 343 32 1165 1 1

9 344 32 1459 1 4

9 345 16 1702 1 3

9 346 8 1671 1 4

9 347 8 1861 0 2

9 348 32 1444 1 3

9 349 8 744 1 4

9 350 16 1071 0 2

9 351 8 1404 1 1

9 352 16 1629 0 1

9 353 16 884 0 2

9 354 32 1328 1 3

9 355 8 816 0 3

9 356 32 1483 1 2

9 357 16 1178 1 1

9 358 16 960 1 3

9 359 32 2239 1 5

9 360 32 1520 0 2

9 361 16 1699 0 1

9 362 32 894 1 4

9 363 32 863 1 4

9 364 16 1040 1 5

9 365 16 2669 1 2

9 366 8 888 1 3

9 367 8 1005 1 4

9 368 8 1251 1 4

9 369 8 1811 0 1

9 370 16 1132 1 5

9 371 8 1121 1 3

9 372 16 1406 1 1

9 373 16 2507 1 1

9 374 8 1638 1 4

9 375 32 2036 1 2

9 376 8 1700 1 1

9 377 8 854 1 5

9 378 32 1453 1 4

9 379 16 1093 0 1

9 380 8 968 1 4

9 381 8 1077 1 2

9 382 8 1596 1 2

9 383 16 2265 1 4

9 384 32 1582 1 5

9 385 32 1397 1 4

9 386 8 857 1 4

9 387 8 1472 1 1

9 388 32 1098 1 4

9 389 16 1798 1 4

9 390 16 1312 1 3

9 391 16 1084 1 5

9 392 16 1693 0 1

9 393 32 1024 1 3

9 394 8 1469 1 1

9 395 32 2323 1 5

9 396 16 849 1 3

9 397 32 786 1 3

9 398 8 1140 1 2

9 399 8 774 1 4

9 400 32 1119 1 4

9 401 8 1417 0 4

9 402 8 1030 1 3

9 403 32 1184 1 2

9 404 16 1535 1 5

9 405 32 944 1 3

9 406 8 1431 1 1

9 407 16 1367 1 3

9 408 32 1022 1 2

9 409 16 1102 1 2

9 410 16 1271 1 2

9 411 8 1250 1 1

9 412 8 984 1 3

9 413 8 803 1 2

9 414 8 754 1 5

9 415 32 940 1 3

9 416 32 1720 1 1

9 417 32 1006 1 3

9 418 8 1531 1 5

9 419 16 1711 1 3

9 420 32 1854 1 1

9 421 32 1627 1 5

9 422 32 1500 1 1

9 423 16 1183 1 2

9 424 16 1141 1 2

9 425 16 1183 1 5

9 426 32 1224 1 1

9 427 32 1519 1 1

9 428 32 1021 1 1

9 429 8 902 1 5

9 430 8 1039 1 2

9 431 16 1354 1 4

9 432 32 752 1 2

9 433 32 1044 1 1

9 434 16 899 1 1

9 435 32 837 1 2

9 436 16 1423 1 5

9 437 32 786 1 3

9 438 16 1691 0 2

9 439 32 1455 1 5

9 440 32 891 1 4

9 441 32 981 1 5

9 442 16 934 1 5

9 443 16 1479 1 4

9 444 16 979 1 3

9 445 16 1013 0 1

9 446 16 1214 0 2

9 447 16 827 0 2

9 448 32 842 1 1

9 449 8 841 1 5

9 450 8 1111 1 5

9 451 16 748 1 3

9 452 8 2195 1 5

9 453 32 1509 1 5

9 454 16 984 1 3

9 455 32 1155 1 5

9 456 16 1231 1 3

9 457 8 904 1 3

9 458 8 1887 1 1

9 459 16 1402 1 4

9 460 8 1352 0 1

9 461 16 1389 1 5

9 462 8 1637 1 2

9 463 8 1407 1 2

9 464 32 1283 1 2

9 465 16 1614 0 1

9 466 32 820 1 2

9 467 8 907 1 3

9 468 16 944 0 4

9 469 8 1075 1 3

9 470 32 1250 1 2

9 471 8 1078 1 4

9 472 8 1204 1 3

9 473 8 1066 1 5

9 474 32 1133 1 3

9 475 8 1315 1 2

9 476 16 1534 1 4

9 477 32 1109 1 3

9 478 16 1511 1 4

9 479 32 893 1 4

9 480 32 1067 1 5

9 481 16 839 1 5

9 482 8 915 1 3

9 483 16 2129 1 2

9 484 32 1134 1 5

9 485 32 1080 1 2

9 486 32 993 1 4

9 487 16 1114 1 3

9 488 32 1172 0 1

9 489 8 1095 1 3

9 490 8 1739 1 1

9 491 16 1449 1 5

9 492 16 1169 1 3

9 493 16 2811 0 4

9 494 8 838 1 4

9 495 8 981 1 1

9 496 8 788 1 2

9 497 32 1399 1 4

9 498 8 939 1 4

9 499 16 1330 1 3

9 500 8 962 1 5

9 501 16 786 0 2

9 502 32 1070 1 1

9 503 32 1040 1 4

9 504 8 752 1 2

9 505 32 1227 1 5

9 506 16 2089 1 4

9 507 8 896 1 3

9 508 32 1377 1 3

9 509 32 762 1 3

9 510 8 1414 1 5

9 511 32 1309 1 2

9 512 8 969 1 3

9 513 16 1343 1 1

9 514 16 2304 0 4

9 515 32 1370 1 4

9 516 16 942 0 2

9 517 16 885 1 5

9 518 32 778 1 2

9 519 16 1249 0 1

9 520 16 853 1 4

9 521 8 939 0 1

9 522 32 998 1 2

9 523 32 1198 1 1

9 524 8 995 1 2

9 525 32 1531 1 3

9 526 8 970 1 5

9 527 16 1232 1 4

9 528 16 1256 0 2

9 529 8 1091 1 3

9 530 16 1241 0 1

9 531 8 1008 1 2

9 532 8 879 1 4

9 533 32 1504 1 5

9 534 8 1654 1 1

9 535 8 2249 1 1

9 536 8 951 1 5

9 537 32 1449 1 1

9 538 32 1061 1 2

9 539 32 1493 1 4

9 540 8 1109 1 5

9 541 32 1828 1 5

9 542 16 1588 1 1

9 543 32 1526 1 1

9 544 32 1100 1 4

9 545 32 1156 1 3

9 546 16 1222 1 2

9 547 16 1165 1 1

9 548 8 765 1 5

9 549 8 1017 1 1

9 550 32 1258 1 5

9 551 16 932 1 3

9 552 32 1160 1 3

9 553 8 1772 1 4

9 554 8 1099 1 2

9 555 32 1361 1 3

9 556 8 1388 1 1

9 557 32 1248 1 4

9 558 16 1077 1 5

9 559 8 1209 1 4

9 560 32 1047 1 3

9 561 16 1367 0 2

9 562 16 953 1 5

9 563 16 1051 0 2

9 564 16 1610 0 1

9 565 32 1439 1 5

9 566 32 1728 1 1

9 567 16 1746 1 1

9 568 8 1313 1 4

9 569 8 1339 1 5

9 570 8 1019 1 2

9 571 8 1260 1 4

9 572 16 767 1 5

9 573 32 1209 1 1

9 574 8 818 1 3

9 575 32 971 1 2

9 576 16 1907 1 4

9 577 16 1564 1 2

9 578 16 954 1 3

9 579 16 1395 1 3

9 580 16 1921 1 4

9 581 8 1486 1 4

9 582 32 1895 1 5

9 583 32 1097 1 3

9 584 16 2968 0 5

9 585 16 2359 1 3

9 586 16 1666 1 3

9 587 8 862 1 5

9 588 32 982 1 4

9 589 16 967 1 5

9 590 32 1428 1 1

9 591 32 1095 1 2

9 592 16 1135 1 1

9 593 32 1368 1 5

9 594 8 870 1 2

9 595 8 1184 1 3

9 596 16 2292 1 4

9 597 8 1057 1 3

9 598 32 1154 1 2

9 599 8 746 1 2

9 600 8 903 0 1

9 601 8 973 1 4

9 602 16 980 1 3

9 603 32 1464 1 4

9 604 16 1302 1 5

9 605 16 931 1 5

9 606 32 1037 1 1

9 607 32 1265 1 2

9 608 32 905 1 4

9 609 8 862 1 2

9 610 16 1252 0 1

9 611 8 828 1 4

9 612 16 1047 1 5

9 613 32 948 1 3

9 614 32 1189 1 3

9 615 16 1340 1 4

9 616 32 860 1 2

9 617 8 852 1 3

9 618 16 1293 1 1

9 619 32 861 1 3

9 620 32 820 1 2

9 621 32 780 1 4

9 622 16 1334 1 1

9 623 8 839 1 3

9 624 32 1082 1 2

9 625 16 898 1 5

9 626 16 1621 1 4

9 627 16 2833 0 1

9 628 32 1544 1 1

9 629 32 2264 1 3

9 630 32 1212 1 5

9 631 32 1369 1 5

9 632 8 1135 1 5

9 633 32 3128 1 1

9 634 16 1209 1 2

9 635 32 1522 1 3

9 636 32 829 1 4

9 637 32 1015 1 2

9 638 16 1255 1 3

9 639 16 1983 1 4

9 640 8 1454 0 1

9 641 32 1052 1 3

9 642 8 795 1 3

9 643 32 1246 1 5

9 644 16 1404 1 1

9 645 8 1087 1 4

9 646 16 1423 0 2

9 647 16 1348 1 1

9 648 16 779 1 5

9 649 32 938 1 1

9 650 32 990 1 4

9 651 16 1144 1 3

9 652 16 1245 1 3

9 653 8 1088 1 2

9 654 32 987 1 4

9 655 16 1258 1 3

9 656 32 1008 1 1

9 657 32 748 1 3

9 658 16 965 1 2

9 659 32 1366 1 4

9 660 8 812 1 3

9 661 16 1112 1 2

9 662 32 1195 1 5

9 663 8 910 1 2

9 664 32 742 1 1

9 665 8 821 1 3

9 666 16 886 1 1

9 667 8 1250 1 1

9 668 32 1841 1 2

9 669 8 1043 1 4

9 670 32 1122 1 5

9 671 16 1405 1 2

9 672 8 1002 1 2

9 673 8 809 1 5

9 674 8 681 1 3

9 675 8 971 1 4

9 676 8 701 1 5

9 677 16 922 1 5

9 678 8 768 1 3

9 679 8 651 1 5

9 680 8 532 1 5

9 681 16 888 1 4

9 682 32 945 1 5

9 683 8 706 1 1

9 684 16 992 0 1

9 685 8 882 1 5

9 686 32 890 1 5

9 687 32 1419 1 5

9 688 8 936 1 2

9 689 32 1044 1 4

9 690 8 741 1 5

9 691 8 717 1 2

9 692 16 810 1 5

9 693 32 1070 1 2

9 694 16 1918 1 4

9 695 8 995 1 4

9 696 32 1409 1 3

9 697 16 998 0 2

9 698 16 1103 1 4

9 699 32 917 1 2

9 700 16 1308 1 3

9 701 16 1060 1 3

9 702 8 1253 1 5

9 703 8 1062 1 2

9 704 8 1879 1 1

9 705 32 1192 1 1

9 706 8 1615 0 1

9 707 8 1304 1 2

9 708 8 1484 1 1

9 709 8 1731 1 1

9 710 16 1070 1 2

9 711 16 1034 1 5

9 712 16 1013 1 3

9 713 8 793 1 4

9 714 16 1629 1 4

9 715 16 1133 1 4

9 716 32 970 1 1

9 717 8 819 1 4

9 718 8 865 1 3

9 719 16 1313 0 2

9 720 8 1362 1 1

9 721 8 1050 1 4

9 722 16 954 0 2

9 723 32 1104 1 4

9 724 8 952 1 5

9 725 16 1418 0 2

9 726 32 949 1 1

9 727 8 1015 1 3

9 728 16 1073 1 5

9 729 32 1583 1 1

9 730 8 1382 1 1

9 731 32 1135 1 4

9 732 8 808 1 5

9 733 8 886 1 3

9 734 32 988 1 2

9 735 32 905 1 3

9 736 8 1368 1 5

9 737 32 1578 1 4

9 738 16 1009 1 3

9 739 32 956 1 4

9 740 16 855 1 3

9 741 8 780 1 2

9 742 8 1013 1 1

9 743 8 1411 1 4

9 744 16 826 1 5

9 745 16 2243 1 4

9 746 32 1017 1 3

9 747 32 994 1 3

9 748 16 1341 1 2

9 749 8 1268 1 3

9 750 16 1526 1 3

9 751 8 1333 1 2

9 752 16 1038 1 3

9 753 8 1058 1 4

9 754 32 1195 1 4

9 755 16 1245 1 2

9 756 8 1506 0 1

9 757 16 1659 1 1

9 758 16 1003 1 1

9 759 8 1018 1 5

9 760 16 906 0 2

9 761 16 859 1 4

9 762 8 870 1 5

9 763 32 900 1 1

9 764 16 915 1 1

9 765 16 858 1 1

9 766 8 684 1 1

9 767 8 827 1 5

9 768 16 1334 1 4

9 769 32 1612 1 5

9 770 8 752 1 5

9 771 16 1202 1 4

9 772 8 961 1 3

9 773 16 1055 1 3

9 774 32 1191 1 3

9 775 32 996 1 1

9 776 8 1088 0 1

9 777 16 921 1 3

9 778 16 1795 1 2

9 779 16 1634 1 4

9 780 16 1459 0 1

9 781 16 990 1 5

9 782 8 1338 1 2

9 783 16 1376 0 1

9 784 8 997 1 3

9 785 16 1246 1 5

9 786 32 2335 1 1

9 787 32 1167 1 5

9 788 32 1116 1 2

9 789 16 1272 1 4

9 790 16 1027 1 2

9 791 32 865 1 2

9 792 32 1041 1 5

9 793 8 823 0 4

9 794 32 1264 1 1

9 795 8 969 1 3

9 796 32 958 1 5

9 797 32 1153 1 5

9 798 16 2150 1 1

9 799 32 740 1 4

9 800 16 758 0 2

9 801 32 723 1 3

9 802 16 1311 1 4

9 803 32 1320 1 5

9 804 8 984 1 5

9 805 8 795 1 1

9 806 8 745 1 4

9 807 32 1017 1 3

9 808 32 1599 1 5

9 809 32 1248 1 2

9 810 16 1612 1 4

9 811 8 1046 1 4

9 812 8 899 1 2

9 813 32 994 1 2

9 814 8 1413 1 1

9 815 32 838 1 4

9 816 16 1312 1 5

9 817 32 1171 1 3

9 818 32 850 1 1

9 819 8 1182 1 3

9 820 32 944 1 2

9 821 8 818 1 2

9 822 32 1423 1 1

9 823 8 1637 1 2

9 824 32 755 1 2

9 825 8 871 1 4

9 826 32 1161 1 3

9 827 32 920 1 4

9 828 16 1232 0 1

9 829 32 909 1 2

9 830 16 914 1 5

9 831 16 1172 1 3

9 832 8 1329 1 2

9 833 32 2178 1 5

9 834 16 1218 1 3

9 835 8 1067 1 2

9 836 16 776 1 5

9 837 8 954 1 4

9 838 16 766 1 5

9 839 8 1181 1 3

9 840 8 1052 1 1

9 841 8 943 1 3

9 842 32 1031 1 2

9 843 16 1106 1 3

9 844 32 661 1 3

9 845 32 815 1 2

9 846 8 921 1 4

9 847 8 1026 1 3

9 848 8 697 1 5

9 849 8 1019 1 2

9 850 8 631 1 5

9 851 32 1089 1 3

9 852 16 665 1 5

9 853 8 891 0 1

9 854 16 772 1 5

9 855 8 709 1 5

9 856 8 531 1 4

9 857 16 969 1 4

9 858 32 1340 1 2

9 859 16 1087 1 1

9 860 32 1053 1 1

9 861 32 1189 1 4

9 862 16 1630 1 4

9 863 16 1249 1 3

9 864 16 3894 0 1

9 865 32 978 1 1

9 866 32 1098 1 5

9 867 32 1044 0 1

9 868 16 900 1 2

9 869 32 1085 1 4

9 870 8 1297 1 4

9 871 16 1774 0 1

9 872 8 834 1 4

9 873 8 864 1 2

9 874 16 1342 1 3

9 875 16 952 1 5

9 876 32 1053 1 3

9 877 8 758 1 2

9 878 16 947 1 5

9 879 8 880 1 4

9 880 8 681 1 3

9 881 16 1764 1 1

9 882 8 626 1 3

9 883 32 710 1 1

9 884 32 970 1 3

9 885 16 732 0 2

9 886 8 908 1 5

9 887 8 1501 1 1

9 888 8 982 1 1

9 889 16 734 1 2

9 890 32 1198 1 5

9 891 32 667 1 3

9 892 8 1006 1 1

9 893 8 1281 1 1

9 894 8 1181 1 4

9 895 8 926 1 2

9 896 8 726 1 3

9 897 16 1679 1 4

9 898 8 1611 1 4

9 899 32 1429 1 4

9 900 8 1345 1 2

9 901 16 1238 1 3

9 902 16 1004 0 2

9 903 8 241 0 1

9 904 8 891 1 3

9 905 16 1951 1 2

9 906 32 924 1 4

9 907 16 1438 1 4

9 908 32 979 1 3

9 909 16 838 1 3

9 910 32 1533 1 5

9 911 32 1222 1 1

9 912 32 1030 1 5

9 913 32 1009 1 2

9 914 8 1343 1 1

9 915 8 1214 1 2

9 916 8 1390 0 1

9 917 8 1138 1 4

9 918 32 1449 1 5

9 919 32 1356 1 5

9 920 32 1468 1 2

9 921 16 16748 1 1

9 922 16 959 1 2

9 923 8 1020 1 5

9 924 32 1206 1 2

9 925 16 1381 1 4

9 926 32 1212 1 1

9 927 16 1299 1 1

9 928 8 979 1 3

9 929 16 932 1 1

9 930 16 879 1 5

9 931 8 878 1 2

9 932 16 674 1 5

9 933 32 1142 1 3

9 934 16 1397 1 4

9 935 32 835 1 1

9 936 32 917 1 4

9 937 32 866 1 4

9 938 16 1141 1 3

9 939 8 958 1 2

9 940 16 1386 1 3

9 941 32 696 1 2

9 942 16 1596 1 4

9 943 16 1168 0 2

9 944 32 700 1 3

9 945 8 1234 1 5

9 946 32 911 1 4

9 947 8 711 1 5

9 948 16 1048 1 1

9 949 16 798 1 4

9 950 32 653 1 2

9 951 8 1881 1 3

9 952 32 752 1 4

9 953 16 814 1 5

9 954 32 1062 1 1

9 955 32 1007 1 5

9 956 16 1133 1 3

9 957 16 1086 0 2

9 958 16 759 1 5

9 959 8 862 1 5

9 960 32 1062 1 5

9 961 16 1353 1 3

9 962 16 1053 1 2

9 963 8 916 1 4

9 964 16 1294 1 4

9 965 16 706 1 5

9 966 16 1457 1 1

9 967 16 1088 1 1

9 968 16 1334 1 3

9 969 8 1294 1 3

9 970 16 1229 1 3

9 971 32 1199 1 5

9 972 32 862 1 2

9 973 8 927 1 3

9 974 16 1531 1 4

9 975 16 1638 1 2

9 976 32 1009 1 3

9 977 8 1334 1 1

9 978 16 885 1 3

9 979 16 1276 1 5

9 980 32 1063 1 1

9 981 32 938 1 4

9 982 8 947 1 4

9 983 16 1340 1 2

9 984 8 1102 1 1

9 985 32 1294 1 3

9 986 8 1278 1 2

9 987 16 1098 1 4

9 988 8 723 1 3

9 989 32 676 1 1

9 990 8 1198 1 1

9 991 8 1049 1 2

9 992 16 1381 1 1

9 993 32 977 1 2

9 994 8 848 1 5

9 995 8 666 1 4

9 996 32 1084 1 4

9 997 8 780 1 5

9 998 8 838 1 2

9 999 8 1510 1 1

9 1000 16 1102 1 3

9 1001 8 2232 1 4

9 1002 8 5696 1 4

9 1003 16 1846 1 2

9 1004 8 1150 1 4

9 1005 8 812 1 3

9 1006 8 915 1 5

9 1007 32 1117 1 3

9 1008 32 1988 1 5

9 1009 32 895 1 4

9 1010 32 867 1 4

9 1011 16 1094 1 5

9 1012 32 1786 1 4

9 1013 16 2389 1 4

9 1014 32 1187 1 1

9 1015 32 848 1 3

9 1016 16 1028 1 1

9 1017 8 998 1 3

9 1018 16 1462 0 2

9 1019 32 1043 1 2

9 1020 8 1605 1 2

9 1021 32 821 1 4

9 1022 16 1309 1 4

9 1023 8 1016 1 1

9 1024 8 802 1 3

9 1025 32 748 1 3

9 1026 32 1468 1 1

9 1027 8 988 1 5

9 1028 32 1293 1 5

9 1029 32 1484 1 5

9 1030 8 1011 1 2

9 1031 32 668 1 2

9 1032 8 1024 1 2

9 1033 16 984 0 2

9 1034 16 894 1 2

9 1035 32 1373 1 2

9 1036 8 912 1 4

9 1037 32 1178 1 1

9 1038 16 1330 1 1

9 1039 8 1071 1 2

9 1040 16 790 1 3

9 1041 32 780 1 3

9 1042 16 1028 1 4

9 1043 32 800 1 1

9 1044 32 713 1 2

9 1045 32 1069 1 4

9 1046 8 905 0 1

9 1047 16 1002 1 3

9 1048 32 1590 1 1

9 1049 8 1356 1 1

9 1050 16 977 0 2

9 1051 32 1648 1 2

9 1052 8 1023 1 2

9 1053 8 778 1 5

9 1054 16 1383 0 1

9 1055 16 1036 1 3

9 1056 32 864 1 3

9 1057 16 2075 1 4

9 1058 32 1199 1 5

9 1059 32 711 1 2

9 1060 16 900 1 1

9 1061 32 796 1 5

9 1062 16 771 1 5

9 1063 16 794 1 5

9 1064 8 872 1 5

9 1065 16 551 1 5

9 1066 8 1009 1 1

9 1067 16 771 0 1

9 1068 8 919 1 3

9 1069 16 1358 1 4

9 1070 32 1355 1 5

9 1071 16 1825 1 5

9 1072 16 803 1 5

9 1073 8 977 1 5

9 1074 32 1862 1 1

9 1075 32 1159 1 4

9 1076 32 1098 1 3

9 1077 8 869 1 4

9 1078 32 1017 1 5

9 1079 8 1482 1 3

9 1080 8 945 1 5

9 1081 32 1317 1 4

9 1082 32 1444 1 2

9 1083 16 1226 1 5

9 1084 32 943 1 2

9 1085 32 1349 0 4

9 1086 8 1039 1 3

9 1087 8 1096 1 4

9 1088 8 880 1 2

9 1089 32 3004 1 5

9 1090 8 890 1 4

9 1091 16 826 1 3

9 1092 8 1483 1 2

9 1093 32 1185 1 5

9 1094 16 1034 0 1

9 1095 8 786 1 3

9 1096 32 840 1 4

9 1097 16 1121 1 3

9 1098 32 913 1 4

9 1099 8 854 1 3

9 1100 8 1056 1 1

9 1101 32 1032 1 2

9 1102 16 1261 1 4

9 1103 16 1362 1 4

9 1104 8 947 1 5

9 1105 8 1190 1 1

9 1106 16 1192 1 4

9 1107 16 1529 1 2

9 1108 8 862 1 3

9 1109 8 785 1 5

9 1110 16 1732 1 5

9 1111 8 915 1 4

9 1112 16 1595 1 3

9 1113 16 2503 1 4

9 1114 8 992 1 4

9 1115 16 1362 1 3

9 1116 16 1120 0 2

9 1117 32 883 1 1

9 1118 16 802 1 5

9 1119 32 935 1 3

9 1120 32 938 1 1

9 1121 8 1301 1 4

9 1122 32 1102 1 2

9 1123 8 714 1 2

9 1124 32 1036 1 3

9 1125 32 1167 1 5

9 1126 8 885 1 1

9 1127 16 894 0 2

9 1128 16 1169 1 2

9 1129 8 1078 1 2

9 1130 32 1105 1 4

9 1131 8 891 1 5

9 1132 8 961 1 5

9 1133 32 768 1 1

9 1134 32 1624 1 1

9 1135 8 937 1 4

9 1136 32 1011 1 2

9 1137 16 2090 1 1

9 1138 16 747 1 5

9 1139 16 1244 1 1

9 1140 8 868 1 2

9 1141 16 983 1 3

9 1142 32 1428 1 5

9 1143 8 1964 1 3

9 1144 16 1721 1 5

9 1145 8 1046 1 4

9 1146 16 2088 1 1

9 1147 32 1882 1 5

9 1148 16 1038 1 3

9 1149 8 949 1 5

9 1150 32 913 1 1

9 1151 32 1371 1 5

9 1152 8 822 1 5

9 1153 8 844 1 3

9 1154 32 1174 1 1

9 1155 8 1641 1 1

9 1156 32 1276 1 5

9 1157 32 1393 1 4

9 1158 8 1058 1 2

9 1159 16 1154 1 5

9 1160 32 765 1 2

9 1161 8 965 1 2

9 1162 32 792 1 2

9 1163 16 1388 1 4

9 1164 16 894 1 2

9 1165 16 891 0 2

9 1166 8 914 1 3

9 1167 8 725 1 3

9 1168 32 1811 1 3

9 1169 16 1904 1 4

9 1170 8 1550 0 1

9 1171 8 793 1 5

9 1172 8 911 1 4

9 1173 8 803 1 2

9 1174 16 1609 1 1

9 1175 32 811 1 4

9 1176 32 646 1 3

9 1177 32 641 1 1

9 1178 32 930 1 2

9 1179 32 1055 1 3

9 1180 16 1184 1 1

9 1181 16 930 1 5

9 1182 32 1139 1 5

9 1183 32 1018 1 4

9 1184 16 1080 0 3

9 1185 16 1302 1 4

9 1186 16 823 1 2

9 1187 8 1402 1 1

9 1188 8 667 1 5

9 1189 16 997 1 3

9 1190 32 727 1 3

9 1191 32 1366 1 1

9 1192 16 1224 1 1

9 1193 32 1587 1 3

9 1194 16 1498 1 4

9 1195 16 936 0 2

9 1196 16 886 1 5

9 1197 32 922 1 3

9 1198 8 882 0 1

9 1199 16 1473 0 1

9 1200 8 899 0 1

10 1 16 1316 0 1

10 2 32 1168 1 3

10 3 16 1139 1 3

10 4 32 1200 1 5

10 5 32 1072 1 1

10 6 32 950 1 3

10 7 16 1309 0 1

10 8 8 1945 1 1

10 9 16 2126 1 3

10 10 8 1098 1 2

10 11 16 1158 1 4

10 12 32 1189 1 5

10 13 16 1116 1 4

10 14 32 1228 1 3

10 15 16 885 1 3

10 16 8 743 0 2

10 17 16 1164 0 5

10 18 32 1200 1 4

10 19 16 873 0 1

10 20 32 1091 1 4

10 21 32 1212 1 2

10 22 8 972 1 5

10 23 16 922 1 3

10 24 16 908 1 1

10 25 8 1007 1 5

10 26 16 939 0 2

10 27 32 1075 1 1

10 28 16 1163 1 2

10 29 16 1016 1 5

10 30 16 1018 0 2

10 31 32 1098 1 2

10 32 32 754 1 2

10 33 16 925 1 3

10 34 8 1271 1 3

10 35 8 942 1 5

10 36 32 1031 0 1

10 37 8 979 1 3

10 38 32 986 1 1

10 39 16 965 1 4

10 40 32 944 1 3

10 41 16 1061 1 1

10 42 8 1238 1 1

10 43 32 1044 1 2

10 44 8 968 1 2

10 45 8 1115 1 2

10 46 32 1000 1 1

10 47 32 990 1 3

10 48 16 729 1 1

10 49 8 924 1 4

10 50 8 931 1 1

10 51 32 857 1 5

10 52 16 796 1 5

10 53 32 1440 1 4

10 54 16 1113 0 2

10 55 32 872 1 5

10 56 16 1062 1 1

10 57 16 1140 0 2

10 58 32 764 1 3

10 59 8 968 1 4

10 60 32 993 1 5

10 61 8 868 1 5

10 62 32 837 1 4

10 63 16 988 1 1

10 64 8 792 1 5

10 65 16 1000 0 4

10 66 16 661 1 4

10 67 32 935 1 5

10 68 32 834 1 1

10 69 8 1068 1 1

10 70 16 1176 1 5

10 71 16 770 1 5

10 72 8 899 1 5

10 73 8 1006 1 4

10 74 32 918 1 5

10 75 32 896 1 4

10 76 8 1141 0 2

10 77 8 1571 1 3

10 78 32 1249 1 4

10 79 8 1108 1 1

10 80 16 1323 1 3

10 81 8 997 0 4

10 82 8 1968 1 4

10 83 32 942 1 3

10 84 32 748 1 2

10 85 16 927 0 4

10 86 16 2058 0 4

10 87 32 1120 1 2

10 88 32 1042 1 3

10 89 16 1552 1 4

10 90 8 835 1 3

10 91 8 836 1 1

10 92 16 1139 0 2

10 93 16 1066 1 3

10 94 16 1075 1 5

10 95 8 913 1 3

10 96 16 829 1 5

10 97 16 1002 1 2

10 98 8 771 1 4

10 99 16 1021 1 2

10 100 8 965 1 1

10 101 8 851 1 1

10 102 32 980 1 4

10 103 8 877 1 3

10 104 32 1042 1 1

10 105 8 742 0 2

10 106 8 876 1 2

10 107 32 1262 1 4

10 108 8 1132 1 3

10 109 8 937 1 5

10 110 8 814 1 5

10 111 32 1050 1 5

10 112 16 784 1 3

10 113 8 779 1 4

10 114 8 895 1 2

10 115 32 827 1 2

10 116 16 1019 1 5

10 117 32 1025 0 2

10 118 32 1032 0 1

10 119 8 1587 1 4

10 120 8 1317 1 3

10 121 8 973 1 3

10 122 32 869 1 4

10 123 16 834 1 3

10 124 16 820 1 3

10 125 16 964 1 5

10 126 8 948 1 1

10 127 32 919 1 4

10 128 8 914 1 2

10 129 32 814 1 5

10 130 8 1364 1 4

10 131 32 1138 1 1

10 132 8 772 1 4

10 133 8 683 1 4

10 134 8 1405 1 1

10 135 8 866 1 5

10 136 16 1278 0 1

10 137 32 1432 1 1

10 138 16 1273 1 3

10 139 8 963 1 4

10 140 8 983 1 1

10 141 16 1761 0 1

10 142 32 1267 1 2

10 143 8 935 1 2

10 144 32 925 1 3

10 145 8 810 1 2

10 146 32 683 1 3

10 147 8 920 1 2

10 148 8 728 1 5

10 149 8 1314 1 2

10 150 16 829 1 5

10 151 8 1111 0 1

10 152 16 1147 0 2

10 153 32 850 1 1

10 154 32 735 1 3

10 155 16 962 0 4

10 156 32 879 1 4

10 157 32 1120 1 4

10 158 16 879 1 2

10 159 8 874 1 2

10 160 32 703 1 2

10 161 32 880 1 3

10 162 8 887 1 3

10 163 32 909 1 2

10 164 16 959 1 1

10 165 32 818 1 5

10 166 16 891 1 4

10 167 32 674 1 2

10 168 32 868 1 1

10 169 32 997 1 5

10 170 8 785 1 4

10 171 16 1057 1 2

10 172 32 697 1 2

10 173 8 865 1 2

10 174 32 769 1 5

10 175 32 1125 1 1

10 176 16 971 0 1

10 177 16 1374 1 4

10 178 16 1071 1 5

10 179 16 967 1 5

10 180 8 773 1 3

10 181 16 1029 0 2

10 182 32 922 1 5

10 183 32 808 1 5

10 184 8 789 1 3

10 185 8 977 1 5

10 186 32 879 1 1

10 187 16 858 0 2

10 188 8 900 1 3

10 189 16 893 0 2

10 190 16 887 1 3

10 191 32 716 1 4

10 192 16 793 1 3

10 193 16 651 1 5

10 194 16 1111 1 5

10 195 8 1023 1 1

10 196 16 1120 1 4

10 197 32 773 1 2

10 198 32 814 1 3

10 199 32 1314 1 3

10 200 32 941 1 2

10 201 16 1742 1 4

10 202 16 870 0 1

10 203 16 1033 1 5

10 204 8 725 0 5

10 205 8 1195 1 1

10 206 32 823 1 4

10 207 16 880 1 3

10 208 8 838 1 5

10 209 32 935 1 5

10 210 16 1133 0 2

10 211 16 1888 0 4

10 212 8 1024 1 3

10 213 32 1188 1 1

10 214 8 817 1 5

10 215 8 888 1 4

10 216 32 1133 1 1

10 217 16 1419 0 2

10 218 8 1186 1 1

10 219 8 990 1 3

10 220 8 880 1 2

10 221 16 908 1 3

10 222 16 935 1 3

10 223 16 712 1 5

10 224 32 803 1 4

10 225 16 840 1 4

10 226 32 917 1 3

10 227 32 945 1 2

10 228 32 713 1 3

10 229 16 955 1 1

10 230 16 862 0 1

10 231 8 1142 1 3

10 232 16 1350 1 4

10 233 32 832 1 4

10 234 8 780 1 5

10 235 8 898 1 4

10 236 16 930 0 1

10 237 8 914 1 5

10 238 32 727 1 5

10 239 8 764 1 4

10 240 8 793 1 1

10 241 16 1462 0 2

10 242 8 975 1 1

10 243 8 878 1 2

10 244 8 852 1 2

10 245 8 839 1 5

10 246 16 786 0 4

10 247 8 1038 1 5

10 248 16 951 1 1

10 249 8 685 1 2

10 250 32 875 1 4

10 251 8 716 1 1

10 252 8 792 1 4

10 253 8 945 1 3

10 254 32 1025 1 4

10 255 8 730 1 3

10 256 32 727 1 1

10 257 32 773 1 3

10 258 16 775 1 2

10 259 32 640 1 1

10 260 32 757 1 4

10 261 8 810 1 5

10 262 8 727 1 3

10 263 16 822 1 5

10 264 16 741 1 5

10 265 16 785 1 1

10 266 32 866 1 1

10 267 16 995 1 1

10 268 8 784 1 4

10 269 32 716 1 4

10 270 16 911 0 1

10 271 16 768 1 4

10 272 16 1171 1 1

10 273 8 858 1 4

10 274 8 836 1 3

10 275 8 642 1 2

10 276 8 769 1 2

10 277 8 783 0 3

10 278 32 828 1 4

10 279 8 840 1 5

10 280 32 958 1 3

10 281 8 1110 1 1

10 282 8 709 1 5

10 283 8 1023 1 5

10 284 8 1223 1 1

10 285 32 776 1 5

10 286 32 890 1 2

10 287 16 1755 1 1

10 288 32 1055 1 4

10 289 16 1374 1 4

10 290 8 841 1 4

10 291 16 965 1 3

10 292 8 997 1 2

10 293 32 920 1 3

10 294 16 808 1 5

10 295 32 710 1 3

10 296 8 1097 1 1

10 297 16 771 1 3

10 298 16 762 1 2

10 299 16 1804 0 1

10 300 32 798 1 2

10 301 16 1008 1 3

10 302 16 821 1 5

10 303 16 885 1 2

10 304 16 836 1 2

10 305 32 897 1 1

10 306 32 658 1 5

10 307 16 953 1 1

10 308 32 821 1 5

10 309 16 742 1 3

10 310 32 738 0 1

10 311 8 631 1 3

10 312 32 781 0 5

10 313 16 1230 1 5

10 314 16 667 1 5

10 315 32 778 1 3

10 316 32 612 1 5

10 317 8 1065 1 1

10 318 32 922 1 3

10 319 32 721 1 2

10 320 16 1109 0 2

10 321 32 695 1 1

10 322 16 888 0 2

10 323 16 913 1 3

10 324 8 965 0 1

10 325 8 880 1 4

10 326 32 885 1 4

10 327 16 914 1 5

10 328 32 1164 1 5

10 329 8 875 1 3

10 330 16 774 1 4

10 331 32 816 1 5

10 332 32 746 1 3

10 333 8 941 1 4

10 334 8 848 1 5

10 335 32 774 1 4

10 336 16 743 1 4

10 337 8 809 1 5

10 338 16 738 1 4

10 339 32 772 1 2

10 340 32 691 1 2

10 341 32 962 1 2

10 342 8 967 1 2

10 343 32 736 1 2

10 344 16 927 0 4

10 345 8 889 1 4

10 346 8 966 1 4

10 347 8 852 1 3

10 348 32 804 1 5

10 349 32 730 1 2

10 350 16 1239 1 4

10 351 32 661 1 3

10 352 16 776 1 3

10 353 16 905 1 3

10 354 16 587 1 5

10 355 16 929 1 3

10 356 32 865 1 1

10 357 32 928 1 1

10 358 8 933 1 1

10 359 16 1006 0 2

10 360 8 753 1 2

10 361 16 1522 1 3

10 362 32 912 1 4

10 363 16 1012 1 5

10 364 32 618 1 4

10 365 16 893 1 3

10 366 16 724 1 4

10 367 8 674 1 4

10 368 32 1127 1 1

10 369 16 773 1 5

10 370 16 1029 1 3

10 371 16 1087 0 1

10 372 8 936 1 1

10 373 16 1295 0 2

10 374 16 683 1 5

10 375 16 787 1 3

10 376 16 726 1 1

10 377 8 748 1 1

10 378 32 881 1 5

10 379 8 934 1 5

10 380 8 753 1 5

10 381 32 1306 1 2

10 382 16 870 1 5

10 383 8 930 1 2

10 384 16 999 1 4

10 385 32 764 1 3

10 386 16 667 1 4

10 387 16 640 1 4

10 388 32 704 1 2

10 389 16 839 1 3

10 390 8 869 1 2

10 391 8 729 1 3

10 392 32 873 1 4

10 393 8 812 0 3

10 394 8 1362 1 4

10 395 16 832 1 5

10 396 32 621 1 2

10 397 8 736 1 5

10 398 32 976 1 5

10 399 8 736 1 5

10 400 16 994 1 2

10 401 32 939 1 1

10 402 8 906 1 2

10 403 32 783 1 5

10 404 8 936 1 2

10 405 16 865 1 5

10 406 16 756 1 4

10 407 8 576 1 3

10 408 32 650 1 4

10 409 8 880 1 3

10 410 8 790 0 1

10 411 32 750 1 3

10 412 32 905 1 3

10 413 32 715 1 3

10 414 32 837 1 3

10 415 32 602 1 3

10 416 32 912 1 5

10 417 16 804 0 1

10 418 16 1219 1 1

10 419 32 892 1 4

10 420 8 627 1 5

10 421 8 783 1 1

10 422 8 1318 1 1

10 423 8 977 1 5

10 424 32 826 1 5

10 425 8 717 1 4

10 426 32 763 1 4

10 427 32 1024 1 1

10 428 8 863 1 5

10 429 8 714 1 5

10 430 16 748 0 4

10 431 32 1272 1 1

10 432 16 1036 1 2

10 433 32 944 1 2

10 434 8 785 1 4

10 435 16 1606 0 2

10 436 16 826 1 3

10 437 8 1064 1 1

10 438 32 762 1 1

10 439 32 951 1 2

10 440 16 874 1 1

10 441 32 861 1 1

10 442 32 623 1 3

10 443 16 889 1 2

10 444 8 967 1 3

10 445 32 829 1 4

10 446 32 658 1 5

10 447 32 828 1 5

10 448 16 1084 1 1

10 449 16 938 1 1

10 450 32 739 1 2

10 451 16 916 1 3

10 452 8 751 1 4

10 453 8 684 1 1

10 454 32 827 1 5

10 455 8 727 1 3

10 456 16 735 1 5

10 457 8 720 1 2

10 458 8 742 1 4

10 459 16 736 1 4

10 460 8 739 1 4

10 461 32 891 1 2

10 462 32 864 1 1

10 463 8 634 0 1

10 464 8 745 0 4

10 465 8 861 1 3

10 466 8 726 1 2

10 467 32 750 1 3

10 468 8 737 1 3

10 469 16 1393 1 4

10 470 32 653 1 4

10 471 16 791 1 2

10 472 8 1057 1 2

10 473 32 761 1 2

10 474 16 586 0 3

10 475 16 927 0 2

10 476 16 1119 0 1

10 477 16 814 1 5

10 478 16 1179 0 2

10 479 32 1723 1 1

10 480 8 865 1 2

10 481 16 713 1 4

10 482 16 734 1 5

10 483 8 845 1 1

10 484 32 996 1 1

10 485 16 1564 1 1

10 486 32 644 1 1

10 487 8 874 1 1

10 488 8 722 1 2

10 489 8 726 1 1

10 490 8 789 1 3

10 491 16 672 0 4

10 492 16 1109 1 5

10 493 32 879 1 3

10 494 16 878 1 5

10 495 8 755 1 3

10 496 8 792 1 2

10 497 32 1097 1 1

10 498 16 915 1 4

10 499 32 648 1 3

10 500 32 846 1 5

10 501 32 829 1 2

10 502 8 770 1 5

10 503 16 776 1 5

10 504 8 754 1 1

10 505 8 710 1 3

10 506 32 937 1 5

10 507 16 938 1 3

10 508 32 636 1 4

10 509 8 913 1 4

10 510 16 716 1 1

10 511 32 785 1 5

10 512 16 979 1 2

10 513 16 1059 1 2

10 514 32 707 1 3

10 515 16 1055 1 4

10 516 8 696 1 2

10 517 16 769 0 2

10 518 8 827 1 3

10 519 8 877 1 5

10 520 16 714 1 5

10 521 8 1152 1 2

10 522 32 938 1 5

10 523 32 717 1 3

10 524 16 824 0 3

10 525 8 923 1 1

10 526 16 1026 1 3

10 527 16 1156 1 5

10 528 32 754 1 3

10 529 32 734 1 5

10 530 32 926 1 5

10 531 8 1020 1 4

10 532 32 759 1 5

10 533 16 1082 0 2

10 534 8 899 1 3

10 535 8 764 1 4

10 536 8 701 1 5

10 537 32 831 1 4

10 538 32 1750 1 1

10 539 8 786 1 4

10 540 8 934 1 1

10 541 16 1126 0 2

10 542 8 743 1 5

10 543 16 1629 0 4

10 544 8 787 1 4

10 545 32 785 1 2

10 546 16 803 1 3

10 547 32 849 1 3

10 548 16 999 1 1

10 549 32 708 1 4

10 550 32 657 1 2

10 551 8 628 0 5

10 552 16 1003 1 3

10 553 16 786 1 2

10 554 16 1036 1 1

10 555 16 1270 1 1

10 556 8 739 1 5

10 557 32 956 1 1

10 558 16 770 1 3

10 559 32 657 1 2

10 560 32 850 1 2

10 561 32 1106 1 3

10 562 8 943 1 2

10 563 32 846 1 4

10 564 8 885 0 4

10 565 32 846 1 5

10 566 16 758 1 4

10 567 32 676 1 2

10 568 32 760 1 4

10 569 32 790 1 4

10 570 16 811 1 3

10 571 8 784 1 3

10 572 8 700 1 5

10 573 16 718 1 5

10 574 8 1255 1 5

10 575 8 809 1 4

10 576 8 957 1 3

10 577 16 1017 0 1

10 578 32 843 1 4

10 579 16 700 0 3

10 580 16 1444 1 4

10 581 16 811 0 2

10 582 16 886 1 1

10 583 32 1019 1 1

10 584 8 1043 1 1

10 585 32 724 1 4

10 586 8 857 1 1

10 587 8 786 1 4

10 588 32 751 1 1

10 589 8 802 1 2

10 590 16 730 1 5

10 591 32 650 1 3

10 592 32 904 1 2

10 593 16 842 1 4

10 594 8 809 1 2

10 595 8 847 1 2

10 596 16 1219 0 2

10 597 8 768 1 3

10 598 32 784 1 2

10 599 32 743 1 1

10 600 16 887 1 1

10 601 16 1146 1 1

10 602 32 855 1 2

10 603 32 761 1 2

10 604 32 674 1 1

10 605 16 662 1 5

10 606 32 808 1 2

10 607 32 727 1 5

10 608 8 670 1 5

10 609 32 859 1 2

10 610 16 1169 0 2

10 611 16 939 1 3

10 612 16 916 1 3

10 613 8 721 1 1

10 614 32 888 1 2

10 615 8 806 1 3

10 616 8 884 1 2

10 617 16 699 1 5

10 618 8 726 1 1

10 619 8 892 1 3

10 620 16 766 1 4

10 621 16 1076 1 1

10 622 32 720 1 3

10 623 8 973 1 1

10 624 32 838 1 3

10 625 8 690 1 3

10 626 16 905 1 4

10 627 32 660 1 4

10 628 16 774 1 3

10 629 32 957 1 4

10 630 8 944 1 4

10 631 8 651 1 4

10 632 16 1263 1 1

10 633 32 936 1 5

10 634 16 890 1 4

10 635 16 982 1 4

10 636 8 785 1 4

10 637 32 882 1 3

10 638 8 650 1 5

10 639 8 689 1 4

10 640 32 648 1 5

10 641 16 1330 1 2

10 642 32 625 1 4

10 643 8 763 1 5

10 644 8 897 1 2

10 645 16 1510 1 1

10 646 32 764 1 1

10 647 32 777 1 1

10 648 32 921 1 5

10 649 16 847 1 1

10 650 8 862 1 4

10 651 32 799 1 2

10 652 8 846 1 4

10 653 32 900 1 1

10 654 16 965 1 1

10 655 8 780 1 2

10 656 32 637 1 3

10 657 8 794 1 1

10 658 16 510 0 2

10 659 32 567 1 5

10 660 8 648 1 5

10 661 16 607 1 3

10 662 32 630 1 4

10 663 16 650 1 5

10 664 8 672 1 4

10 665 8 669 1 5

10 666 32 737 1 3

10 667 8 1311 1 4

10 668 8 707 1 3

10 669 32 887 1 4

10 670 32 607 1 3

10 671 8 704 1 5

10 672 8 666 1 1

10 673 16 853 0 4

10 674 32 1037 1 1

10 675 16 1168 1 3

10 676 16 592 1 5

10 677 16 1448 0 2

10 678 8 721 1 2

10 679 32 873 1 4

10 680 32 973 1 1

10 681 8 1548 1 3

10 682 8 651 1 2

10 683 16 834 1 2

10 684 16 1029 1 4

10 685 16 824 1 2

10 686 32 730 1 2

10 687 8 632 1 5

10 688 16 853 1 2

10 689 32 641 1 1

10 690 32 661 1 2

10 691 32 766 1 3

10 692 8 798 1 1

10 693 16 1724 1 1

10 694 8 620 1 2

10 695 32 777 1 5

10 696 16 779 1 3

10 697 8 613 0 2

10 698 32 1191 1 5

10 699 16 707 1 5

10 700 8 649 1 3

10 701 8 641 1 2

10 702 16 736 1 3

10 703 16 1041 1 2

10 704 8 672 1 1

10 705 8 764 1 1

10 706 16 684 1 3

10 707 32 804 1 4

10 708 16 664 1 1

10 709 8 557 1 3

10 710 32 659 1 1

10 711 32 875 1 5

10 712 16 777 1 4

10 713 8 604 1 3

10 714 8 645 1 5

10 715 16 905 1 4

10 716 32 747 1 3

10 717 16 807 1 5

10 718 32 759 1 4

10 719 16 611 1 5

10 720 16 635 1 5

10 721 8 870 1 1

10 722 16 874 1 2

10 723 16 686 1 1

10 724 8 638 1 3

10 725 16 803 1 5

10 726 8 713 1 1

10 727 32 731 1 3

10 728 8 622 1 1

10 729 32 551 1 2

10 730 16 550 1 5

10 731 32 570 1 2

10 732 8 725 1 3

10 733 32 743 1 5

10 734 16 600 1 4

10 735 16 534 1 4

10 736 8 752 1 3

10 737 8 782 0 5

10 738 8 738 0 3

10 739 16 1575 1 2

10 740 32 966 1 5

10 741 16 885 1 5

10 742 16 776 1 2

10 743 32 1058 1 1

10 744 16 1047 1 3

10 745 8 867 1 1

10 746 8 1078 1 5

10 747 8 606 1 4

10 748 32 648 1 4

10 749 8 622 1 3

10 750 16 610 1 5

10 751 8 780 1 2

10 752 32 913 1 3

10 753 8 648 1 4

10 754 32 723 1 4

10 755 16 1029 1 3

10 756 16 920 1 5

10 757 8 755 1 1

10 758 16 693 1 4

10 759 8 643 1 4

10 760 32 677 1 1

10 761 32 1174 1 2

10 762 16 657 1 1

10 763 16 609 1 3

10 764 8 570 1 5

10 765 8 586 1 5

10 766 8 844 1 3

10 767 16 760 1 4

10 768 8 761 1 4

10 769 32 669 1 4

10 770 8 656 1 4

10 771 32 548 1 2

10 772 8 901 1 1

10 773 32 1406 1 4

10 774 8 600 1 5

10 775 32 933 1 5

10 776 32 633 1 4

10 777 16 723 1 2

10 778 32 741 1 1

10 779 16 574 1 5

10 780 16 757 0 1

10 781 16 683 1 5

10 782 32 1093 1 3

10 783 32 865 1 1

10 784 8 597 0 4

10 785 8 1465 1 1

10 786 32 651 0 3

10 787 32 678 0 5

10 788 8 753 1 2

10 789 32 832 1 4

10 790 16 1265 1 2

10 791 8 1004 1 5

10 792 32 794 1 5

10 793 8 832 1 4

10 794 32 1255 1 3

10 795 32 711 1 3

10 796 8 766 1 4

10 797 16 800 1 3

10 798 16 826 0 1

10 799 32 1354 1 3

10 800 32 658 1 5

10 801 8 910 1 2

10 802 16 765 1 1

10 803 8 712 1 5

10 804 16 1255 1 2

10 805 32 776 1 4

10 806 16 719 1 2

10 807 32 624 1 4

10 808 16 669 1 4

10 809 16 633 1 3

10 810 8 534 1 5

10 811 8 756 0 2

10 812 16 865 1 3

10 813 32 959 1 3

10 814 8 765 1 1

10 815 32 1048 0 1

10 816 8 710 1 2

10 817 32 879 1 1

10 818 16 697 1 4

10 819 16 696 1 3

10 820 8 512 1 3

10 821 32 1108 1 1

10 822 16 996 1 2

10 823 16 1053 1 1

10 824 16 825 1 1

10 825 32 997 1 1

10 826 16 733 1 5

10 827 8 677 1 2

10 828 16 862 1 3

10 829 32 758 1 5

10 830 32 833 1 2

10 831 32 690 1 2

10 832 32 752 1 5

10 833 8 777 1 3

10 834 16 847 1 1

10 835 32 792 1 2

10 836 8 825 1 2

10 837 16 1063 1 4

10 838 16 1091 0 4

10 839 32 831 1 2

10 840 8 832 1 2

10 841 8 1074 1 4

10 842 8 854 1 2

10 843 32 847 1 3

10 844 8 598 1 5

10 845 8 968 1 3

10 846 32 776 1 5

10 847 8 824 1 5

10 848 32 1192 1 4

10 849 32 808 1 3

10 850 16 1084 1 2

10 851 32 643 1 5

10 852 16 1693 0 1

10 853 8 734 1 3

10 854 16 952 1 4

10 855 8 934 1 2

10 856 32 797 1 4

10 857 32 705 1 1

10 858 32 757 1 5

10 859 32 675 1 2

10 860 32 1255 1 1

10 861 16 1004 1 3

10 862 16 992 1 3

10 863 16 1086 1 1

10 864 32 641 1 2

10 865 16 813 1 2

10 866 32 763 1 2

10 867 8 707 1 5

10 868 8 1220 1 1

10 869 16 726 1 5

10 870 16 1071 0 4

10 871 8 1031 1 4

10 872 8 656 1 2

10 873 8 667 1 5

10 874 8 722 1 4

10 875 8 624 1 4

10 876 32 725 1 4

10 877 16 621 1 5

10 878 8 1428 1 5

10 879 16 1084 0 2

10 880 32 815 1 1

10 881 16 1491 1 1

10 882 8 575 1 3

10 883 16 715 0 2

10 884 32 800 1 3

10 885 32 747 1 3

10 886 32 778 1 2

10 887 16 748 1 5

10 888 16 814 1 3

10 889 16 1060 1 1

10 890 16 890 1 4

10 891 32 993 1 1

10 892 8 1050 1 2

10 893 32 715 1 1

10 894 32 578 1 4

10 895 8 865 1 3

10 896 8 921 1 1

10 897 16 952 1 3

10 898 32 912 1 2

10 899 16 1070 1 3

10 900 8 710 1 4

10 901 8 1190 1 1

10 902 16 1409 1 5

10 903 16 581 1 3

10 904 8 668 1 2

10 905 8 637 1 4

10 906 16 1736 0 1

10 907 32 918 1 1

10 908 8 672 1 3

10 909 32 765 0 2

10 910 32 930 1 2

10 911 8 804 1 1

10 912 8 758 1 5

10 913 16 973 1 4

10 914 8 693 1 3

10 915 32 807 1 4

10 916 32 615 1 5

10 917 16 614 1 4

10 918 8 774 1 5

10 919 8 756 1 1

10 920 32 741 1 3

10 921 32 823 1 4

10 922 8 860 1 2

10 923 8 652 1 3

10 924 8 679 1 4

10 925 32 734 1 1

10 926 16 834 1 2

10 927 8 650 1 5

10 928 8 646 1 4

10 929 32 878 1 4

10 930 16 687 1 4

10 931 16 1081 1 3

10 932 16 1130 1 5

10 933 32 799 1 5

10 934 32 1144 1 4

10 935 32 710 1 3

10 936 8 800 1 2

10 937 32 769 1 5

10 938 16 2267 0 1

10 939 16 1341 0 2

10 940 16 899 1 2

10 941 16 944 1 3

10 942 32 682 1 5

10 943 8 1482 1 1

10 944 16 608 0 2

10 945 16 636 1 4

10 946 32 739 1 5

10 947 16 820 1 5

10 948 8 780 1 1

10 949 16 654 1 5

10 950 16 625 1 5

10 951 32 699 1 3

10 952 8 803 1 1

10 953 8 545 1 3

10 954 16 687 1 4

10 955 32 707 1 3

10 956 16 628 0 1

10 957 8 799 1 2

10 958 32 960 1 1

10 959 16 959 1 1

10 960 32 853 1 2

10 961 32 707 0 3

10 962 16 1206 1 5

10 963 32 908 1 1

10 964 32 643 1 3

10 965 8 921 1 2

10 966 8 761 1 1

10 967 8 617 1 3

10 968 8 590 1 5

10 969 8 521 1 3

10 970 16 698 1 3

10 971 32 818 1 4

10 972 32 679 1 4

10 973 16 773 1 1

10 974 8 792 1 1

10 975 32 610 1 3

10 976 16 533 1 3

10 977 8 774 1 5

10 978 8 799 1 2

10 979 32 835 1 2

10 980 32 623 1 4

10 981 16 1119 1 3

10 982 8 484 1 4

10 983 16 708 1 2

10 984 16 628 1 1

10 985 32 746 1 2

10 986 16 689 1 5

10 987 32 722 1 4

10 988 8 703 1 3

10 989 8 631 1 5

10 990 32 1011 1 1

10 991 16 848 1 2

10 992 32 969 1 5

10 993 32 640 0 4

10 994 16 787 1 5

10 995 8 727 1 1

10 996 16 695 1 4

10 997 16 593 1 3

10 998 8 552 1 4

10 999 16 908 1 2

10 1000 16 601 1 5

10 1001 32 766 1 2

10 1002 16 645 1 5

10 1003 32 821 1 5

10 1004 32 577 1 5

10 1005 32 764 1 5

10 1006 8 699 1 4

10 1007 16 873 1 5

10 1008 16 819 1 3

10 1009 16 718 1 4

10 1010 8 683 1 1

10 1011 32 823 1 3

10 1012 8 714 1 4

10 1013 8 625 1 4

10 1014 16 646 1 4

10 1015 16 753 1 3

10 1016 32 616 1 3

10 1017 8 745 1 2

10 1018 32 669 1 3

10 1019 8 653 1 5

10 1020 16 687 0 5

10 1021 8 593 1 2

10 1022 16 838 1 2

10 1023 8 992 1 1

10 1024 32 632 1 4

10 1025 8 693 1 2

10 1026 32 615 0 3

10 1027 16 788 0 4

10 1028 16 905 0 2

10 1029 8 804 1 5

10 1030 32 780 1 2

10 1031 16 871 1 2

10 1032 32 894 1 1

10 1033 8 807 1 3

10 1034 32 783 1 4

10 1035 32 895 1 2

10 1036 32 895 1 2

10 1037 16 635 1 2

10 1038 8 725 1 5

10 1039 8 731 1 4

10 1040 32 731 1 1

10 1041 8 742 1 1

10 1042 16 743 1 1

10 1043 16 754 1 4

10 1044 32 709 1 4

10 1045 32 820 1 1

10 1046 16 901 1 2

10 1047 8 969 1 1

10 1048 8 609 1 3

10 1049 8 667 1 3

10 1050 32 714 0 5

10 1051 8 656 1 4

10 1052 8 793 1 2

10 1053 8 734 1 1

10 1054 16 974 1 1

10 1055 16 763 0 1

10 1056 8 748 1 2

10 1057 32 886 1 1

10 1058 16 710 1 4

10 1059 8 661 1 4

10 1060 16 705 1 4

10 1061 32 695 1 2

10 1062 8 714 1 5

10 1063 32 686 0 1

10 1064 32 1364 1 5

10 1065 16 914 1 1

10 1066 8 519 1 2

10 1067 8 611 1 5

10 1068 16 834 1 3

10 1069 8 566 1 3

10 1070 32 874 1 5

10 1071 16 621 1 5

10 1072 16 552 1 4

10 1073 8 559 1 3

10 1074 32 844 1 3

10 1075 32 994 1 1

10 1076 16 760 1 1

10 1077 16 799 1 1

10 1078 16 711 1 3

10 1079 32 1164 1 2

10 1080 32 612 1 5

10 1081 8 831 1 4

10 1082 8 692 1 3

10 1083 8 596 1 4

10 1084 32 584 1 1

10 1085 32 684 1 2

10 1086 8 500 1 1

10 1087 8 561 1 3

10 1088 32 775 1 3

10 1089 8 602 1 5

10 1090 32 682 1 3

10 1091 16 586 1 5

10 1092 16 591 0 1

10 1093 8 782 1 5

10 1094 16 687 1 1

10 1095 32 630 1 3

10 1096 32 731 1 5

10 1097 16 802 0 2

10 1098 32 636 1 5

10 1099 8 578 1 4

10 1100 8 835 1 2

10 1101 16 1808 0 2

10 1102 32 747 1 5

10 1103 8 567 1 5

10 1104 8 457 1 3

10 1105 32 636 1 4

10 1106 16 616 1 5

10 1107 16 622 1 5

10 1108 32 775 1 1

10 1109 8 578 1 4

10 1110 32 599 1 4

10 1111 16 718 1 5

10 1112 32 925 1 2

10 1113 32 724 1 5

10 1114 8 527 1 5

10 1115 8 708 1 2

10 1116 32 619 1 5

10 1117 16 607 1 4

10 1118 16 844 1 3

10 1119 16 597 0 4

10 1120 16 494 1 4

10 1121 32 682 1 4

10 1122 16 698 1 2

10 1123 16 822 1 1

10 1124 16 625 0 4

10 1125 8 647 1 3

10 1126 8 693 1 3

10 1127 16 664 1 5

10 1128 16 628 1 1

10 1129 32 675 1 3

10 1130 8 1734 1 1

10 1131 8 786 1 2

10 1132 8 566 1 5

10 1133 32 630 1 2

10 1134 8 590 1 4

10 1135 32 634 0 4

10 1136 32 819 1 5

10 1137 8 929 1 2

10 1138 8 837 1 4

10 1139 32 690 1 3

10 1140 16 862 1 1

10 1141 32 731 0 1

10 1142 32 1372 1 1

10 1143 16 696 1 4

10 1144 32 677 0 5

10 1145 8 851 1 5

10 1146 8 809 1 3

10 1147 32 725 1 4

10 1148 8 783 1 2

10 1149 32 574 1 3

10 1150 16 739 1 5

10 1151 8 586 1 5

10 1152 16 778 1 2

10 1153 16 880 1 2

10 1154 16 839 1 3

10 1155 32 831 1 1

10 1156 16 587 1 5

10 1157 16 772 0 2

10 1158 16 980 1 3

10 1159 16 779 1 1

10 1160 8 731 1 1

10 1161 16 976 0 1

10 1162 16 977 1 2

10 1163 16 1053 1 3

10 1164 8 667 1 4

10 1165 16 721 1 4

10 1166 8 754 1 1

10 1167 16 957 1 3

10 1168 8 710 1 2

10 1169 16 732 1 4

10 1170 16 582 1 5

10 1171 16 742 1 3

10 1172 32 650 0 2

10 1173 8 1115 1 5

10 1174 32 821 1 4

10 1175 8 893 1 1

10 1176 8 828 1 1

10 1177 8 761 1 3

10 1178 16 775 1 3

10 1179 8 695 1 1

10 1180 8 638 1 3

10 1181 8 646 0 1

10 1182 32 840 1 5

10 1183 32 708 1 1

10 1184 16 916 1 3

10 1185 8 645 0 2

10 1186 32 1034 1 1

10 1187 16 1756 1 4

10 1188 32 817 1 2

10 1189 16 1141 0 2

10 1190 32 617 1 3

10 1191 32 561 1 2

10 1192 32 646 1 4

10 1193 32 780 1 2

10 1194 32 650 1 3

10 1195 32 862 1 4

10 1196 8 759 1 2

10 1197 32 898 1 1

10 1198 8 611 1 4

10 1199 32 542 1 2

10 1200 16 1797 0 1

11 1 32 935 1 1

11 2 32 860 1 5

11 3 32 589 1 4

11 4 32 563 1 3

11 5 32 881 1 1

11 6 16 1043 0 2

11 7 8 812 1 3

11 8 8 565 1 3

11 9 8 637 1 4

11 10 16 698 1 5

11 11 8 659 1 5

11 12 16 855 0 2

11 13 32 752 1 1

11 14 16 635 1 4

11 15 8 768 1 1

11 16 32 823 1 3

11 17 32 868 1 5

11 18 8 739 1 4

11 19 8 774 1 3

11 20 32 762 1 2

11 21 32 732 0 2

11 22 16 1123 1 3

11 23 16 630 0 2

11 24 16 870 1 2

11 25 16 855 0 1

11 26 16 984 1 5

11 27 8 996 1 4

11 28 8 792 1 2

11 29 32 899 1 2

11 30 32 757 1 4

11 31 16 603 1 5

11 32 32 826 1 2

11 33 32 871 1 5

11 34 8 993 1 3

11 35 32 677 1 1

11 36 16 1160 1 2

11 37 32 893 1 3

11 38 8 783 1 1

11 39 32 917 1 1

11 40 8 849 1 3

11 41 8 1081 1 4

11 42 32 673 1 2

11 43 8 576 1 5

11 44 16 680 1 4

11 45 8 624 1 3

11 46 16 1413 1 4

11 47 32 907 1 1

11 48 8 787 1 2

11 49 8 2399 1 5

11 50 8 597 1 5

11 51 32 785 1 5

11 52 8 636 1 4

11 53 32 983 1 1

11 54 32 709 1 2

11 55 16 830 0 1

11 56 32 682 0 4

11 57 8 1291 1 4

11 58 16 811 1 5

11 59 16 1020 0 2

11 60 8 807 1 4

11 61 32 813 1 4

11 62 16 1248 1 3

11 63 8 923 1 2

11 64 16 691 1 2

11 65 16 603 1 3

11 66 16 780 1 4

11 67 8 661 1 2

11 68 16 716 1 1

11 69 32 723 1 5

11 70 16 1065 1 5

11 71 8 736 0 1

11 72 16 763 1 3

11 73 8 1126 1 2

11 74 16 799 1 1

11 75 32 946 1 4

11 76 8 926 1 3

11 77 8 1482 0 1

11 78 16 1226 1 4

11 79 8 733 1 3

11 80 32 1135 1 2

11 81 16 1222 0 1

11 82 8 1153 1 1

11 83 32 646 1 2

11 84 32 1068 1 3

11 85 16 1118 1 3

11 86 8 750 1 2

11 87 16 844 0 1

11 88 16 1077 1 5

11 89 16 990 1 2

11 90 32 970 0 3

11 91 8 774 1 2

11 92 8 559 1 2

11 93 16 1046 1 1

11 94 8 862 1 5

11 95 8 1319 1 5

11 96 8 706 1 5

11 97 8 1198 1 1

11 98 16 953 1 3

11 99 32 805 1 3

11 100 16 535 1 5

11 101 8 960 1 1

11 102 32 795 1 5

11 103 32 889 1 5

11 104 8 937 1 1

11 105 16 931 1 3

11 106 16 833 1 4

11 107 16 998 1 5

11 108 16 1812 1 4

11 109 8 1166 1 5

11 110 32 655 1 5

11 111 8 883 1 4

11 112 32 709 1 3

11 113 32 944 1 4

11 114 16 1164 1 1

11 115 32 744 0 3

11 116 32 920 1 4

11 117 32 773 1 4

11 118 16 1393 0 4

11 119 16 1267 1 3

11 120 32 895 0 1

11 121 8 867 1 3

11 122 8 682 1 2

11 123 16 1326 1 2

11 124 8 685 1 5

11 125 32 1252 1 5

11 126 8 1024 0 1

11 127 32 709 1 4

11 128 32 660 1 5

11 129 8 720 1 5

11 130 8 1062 0 1

11 131 32 1210 1 1

11 132 8 698 0 1

11 133 8 720 1 5

11 134 32 903 1 1

11 135 32 635 1 2

11 136 8 801 1 4

11 137 8 1086 1 2

11 138 16 979 1 3

11 139 16 911 1 2

11 140 32 621 1 2

11 141 8 872 1 2

11 142 16 958 1 3

11 143 16 707 0 3

11 144 32 1742 1 5

11 145 32 708 1 3

11 146 16 881 0 4

11 147 32 938 1 5

11 148 32 623 1 4

11 149 16 728 0 3

11 150 16 1016 1 3

11 151 32 845 1 5

11 152 16 796 1 2

11 153 8 648 1 4

11 154 16 672 1 5

11 155 32 845 0 1

11 156 16 783 1 4

11 157 16 588 1 5

11 158 16 826 0 3

11 159 16 1612 1 4

11 160 8 677 1 2

11 161 16 903 1 4

11 162 32 723 1 2

11 163 8 777 1 3

11 164 8 1063 1 5

11 165 32 838 1 3

11 166 16 1630 1 4

11 167 32 1042 1 3

11 168 16 816 1 5

11 169 32 938 1 3

11 170 8 903 1 3

11 171 8 1309 1 1

11 172 32 1193 1 3

11 173 8 631 1 2

11 174 16 876 1 5

11 175 16 1405 1 1

11 176 32 821 1 2

11 177 32 863 1 3

11 178 8 666 1 4

11 179 16 1372 1 1

11 180 16 1602 0 4

11 181 8 1409 1 5

11 182 16 1215 1 4

11 183 16 549 1 2

11 184 32 672 1 4

11 185 8 1179 1 3

11 186 8 731 1 4

11 187 8 1090 1 1

11 188 16 1207 0 1

11 189 8 745 1 3

11 190 32 987 1 5

11 191 16 1183 0 1

11 192 8 691 1 3

11 193 32 1219 1 4

11 194 16 1344 1 4

11 195 16 1167 1 3

11 196 32 985 1 1

11 197 32 977 1 1

11 198 32 1136 0 2

11 199 32 711 1 5

11 200 32 796 1 2

11 201 8 906 1 3

11 202 8 697 1 4

11 203 8 848 1 1

11 204 8 672 1 2

11 205 8 910 1 2

11 206 16 1287 1 5

11 207 32 974 1 3

11 208 16 682 0 1

11 209 16 857 1 1

11 210 32 821 1 4

11 211 8 775 1 5

11 212 32 1302 1 4

11 213 32 681 1 3

11 214 8 642 1 4

11 215 16 634 1 5

11 216 8 766 1 4

11 217 32 863 1 1

11 218 16 1216 0 1

11 219 16 906 1 5

11 220 8 1118 1 1

11 221 16 1671 1 2

11 222 8 653 1 5

11 223 8 960 0 3

11 224 32 746 1 2

11 225 32 1037 1 2

11 226 32 1247 1 1

11 227 16 807 0 2

11 228 32 1198 1 5

11 229 16 905 0 2

11 230 32 1200 1 4

11 231 32 1341 1 1

11 232 16 735 0 2

11 233 32 588 1 4

11 234 8 1660 1 1

11 235 8 112 0 5

11 236 8 763 1 4

11 237 16 1103 1 1

11 238 8 1080 1 2

11 239 16 815 1 3

11 240 16 826 1 5

11 241 32 828 1 2

11 242 8 634 1 5

11 243 8 791 1 5

11 244 16 1638 1 4

11 245 8 669 1 1

11 246 32 998 1 1

11 247 8 986 1 3

11 248 32 697 1 4

11 249 8 836 1 2

11 250 8 770 1 5

11 251 16 562 1 5

11 252 8 748 1 2

11 253 16 823 0 2

11 254 8 836 1 1

11 255 32 698 1 3

11 256 8 572 1 4

11 257 8 764 1 3

11 258 16 1671 1 4

11 259 32 606 1 1

11 260 16 813 1 4

11 261 16 649 1 3

11 262 8 926 1 1

11 263 8 806 1 5

11 264 8 614 1 4

11 265 16 627 0 5

11 266 16 570 1 5

11 267 8 1089 1 2

11 268 8 735 1 3

11 269 32 601 0 4

11 270 32 842 1 2

11 271 8 730 1 4

11 272 16 786 0 2

11 273 32 739 1 5

11 274 32 566 1 5

11 275 16 950 1 3

11 276 32 782 1 5

11 277 32 572 1 3

11 278 16 621 1 5

11 279 16 974 0 5

11 280 32 685 1 1

11 281 32 883 1 5

11 282 8 724 1 5

11 283 8 818 1 2

11 284 32 726 1 5

11 285 32 694 1 3

11 286 16 745 1 4

11 287 32 715 1 3

11 288 32 498 1 3

11 289 32 725 1 3

11 290 16 614 1 3

11 291 8 1201 1 4

11 292 8 513 1 3

11 293 16 861 0 4

11 294 32 651 1 4

11 295 16 445 1 3

11 296 16 645 1 3

11 297 8 489 1 1

11 298 8 745 1 3

11 299 32 833 1 2

11 300 8 981 1 1

11 301 32 1009 0 1

11 302 32 1163 1 1

11 303 8 756 1 2

11 304 8 752 1 5

11 305 8 761 1 1

11 306 16 903 1 1

11 307 16 697 1 4

11 308 16 948 1 1

11 309 16 593 0 1

11 310 32 627 1 1

11 311 16 695 1 1

11 312 16 894 0 2

11 313 8 1274 1 4

11 314 8 782 1 2

11 315 32 824 1 5

11 316 16 1241 1 5

11 317 16 973 1 4

11 318 32 561 1 2

11 319 32 554 1 4

11 320 32 505 0 5

11 321 16 1476 1 2

11 322 8 602 1 4

11 323 32 1057 1 1

11 324 32 801 1 4

11 325 16 1223 1 1

11 326 8 880 0 2

11 327 8 2468 1 4

11 328 8 617 1 5

11 329 16 867 1 1

11 330 16 959 0 1

11 331 8 795 1 4

11 332 8 631 1 2

11 333 16 859 1 2

11 334 32 693 1 2

11 335 16 745 1 4

11 336 16 541 1 5

11 337 16 566 1 3

11 338 8 720 1 3

11 339 16 791 1 5

11 340 8 915 1 3

11 341 32 769 0 5

11 342 32 682 1 4

11 343 32 651 1 4

11 344 32 728 1 3

11 345 16 1000 1 2

11 346 32 879 1 2

11 347 16 925 1 3

11 348 8 1276 1 1

11 349 16 919 0 2

11 350 8 881 1 3

11 351 16 777 0 3

11 352 8 885 1 5

11 353 8 740 1 1

11 354 32 719 0 2

11 355 32 784 1 1

11 356 16 706 0 1

11 357 32 739 1 2

11 358 16 653 0 2

11 359 32 651 1 3

11 360 32 712 1 4

11 361 8 1124 1 2

11 362 16 905 0 2

11 363 32 1459 1 4

11 364 16 862 0 2

11 365 32 978 1 5

11 366 32 725 1 3

11 367 8 868 1 5

11 368 16 1135 1 3

11 369 16 1040 1 4

11 370 8 698 1 5

11 371 16 627 1 3

11 372 32 488 1 4

11 373 16 640 1 4

11 374 8 689 1 3

11 375 32 682 1 5

11 376 8 1396 1 1

11 377 16 984 1 2

11 378 16 880 1 5

11 379 16 729 1 4

11 380 8 1125 1 1

11 381 16 719 1 1

11 382 8 748 1 4

11 383 8 1214 1 2

11 384 32 774 1 1

11 385 32 1161 1 3

11 386 8 856 1 2

11 387 8 625 1 3

11 388 32 654 1 4

11 389 8 736 1 4

11 390 8 635 1 5

11 391 8 848 1 1

11 392 16 706 0 2

11 393 16 1015 1 3

11 394 16 730 0 1

11 395 16 1025 1 1

11 396 32 592 0 3

11 397 32 666 1 1

11 398 8 1052 1 2

11 399 16 667 0 3

11 400 8 675 1 4

11 401 8 690 1 3

11 402 8 927 1 1

11 403 32 1342 1 1

11 404 16 1369 1 4

11 405 8 786 1 5

11 406 16 758 0 2

11 407 32 895 1 2

11 408 32 1210 1 2

11 409 32 1118 1 4

11 410 16 1800 1 4

11 411 8 580 1 3

11 412 8 846 1 1

11 413 32 713 1 2

11 414 16 705 1 5

11 415 32 878 1 5

11 416 8 791 1 1

11 417 8 954 1 5

11 418 32 943 0 1

11 419 32 1064 1 3

11 420 32 902 0 4

11 421 16 1459 1 1

11 422 16 1173 1 5

11 423 16 1032 1 5

11 424 16 764 1 5

11 425 8 831 1 4

11 426 32 742 1 4

11 427 32 652 1 2

11 428 16 670 1 1

11 429 8 576 1 2

11 430 16 593 1 3

11 431 16 663 1 5

11 432 16 895 1 5

11 433 32 728 0 4

11 434 16 1370 1 4

11 435 8 802 1 5

11 436 16 1062 1 3

11 437 16 1235 1 3

11 438 32 820 0 3

11 439 8 1028 1 2

11 440 16 1061 0 2

11 441 32 903 1 4

11 442 8 814 0 3

11 443 32 821 0 2

11 444 16 1180 1 4

11 445 16 1200 0 1

11 446 32 1038 1 3

11 447 32 1006 1 2

11 448 16 1070 1 2

11 449 16 793 1 2

11 450 8 880 1 5

11 451 8 697 1 4

11 452 16 613 0 4

11 453 8 1155 1 4

11 454 32 1542 1 5

11 455 32 590 1 3

11 456 32 798 1 3

11 457 8 1199 1 1

11 458 32 838 1 5

11 459 32 865 1 5

11 460 32 696 1 1

11 461 32 1297 1 1

11 462 32 796 1 1

11 463 16 822 1 1

11 464 8 615 1 3

11 465 32 685 1 5

11 466 8 1048 1 3

11 467 8 555 1 5

11 468 32 1315 1 2

11 469 16 1223 0 3

11 470 8 935 0 2

11 471 8 1030 1 4

11 472 8 1101 1 2

11 473 32 609 1 1

11 474 8 1367 1 1

11 475 8 1203 1 4

11 476 32 1548 1 5

11 477 16 1447 1 1

11 478 8 679 1 3

11 479 16 722 1 5

11 480 32 731 1 2

11 481 16 1145 1 4

11 482 16 1114 1 1

11 483 16 966 1 5

11 484 16 844 0 1

11 485 32 1053 0 5

11 486 32 681 1 4

11 487 32 1005 1 4

11 488 16 1251 1 2

11 489 16 720 1 3

11 490 32 886 1 2

11 491 32 886 1 2

11 492 16 1372 1 4

11 493 32 668 1 1

11 494 16 1089 1 4

11 495 8 1065 1 5

11 496 32 650 1 1

11 497 8 670 1 4

11 498 8 533 1 3

11 499 32 1316 1 3

11 500 8 617 0 1

11 501 32 686 1 5

11 502 16 637 1 4

11 503 8 737 1 3

11 504 8 739 1 4

11 505 16 989 0 2

11 506 16 811 1 3

11 507 16 1170 0 2

11 508 32 652 1 5

11 509 8 1548 1 1

11 510 8 754 1 5

11 511 8 800 1 4

11 512 16 829 1 5

11 513 16 1066 1 4

11 514 8 953 1 5

11 515 8 757 1 4

11 516 16 1206 1 2

11 517 16 575 1 3

11 518 16 1971 1 4

11 519 8 894 1 2

11 520 8 641 1 2

11 521 16 750 0 3

11 522 32 719 1 3

11 523 8 660 1 2

11 524 8 582 1 3

11 525 8 888 1 1

11 526 16 640 1 1

11 527 32 768 1 1

11 528 8 800 1 5

11 529 32 920 1 4

11 530 32 1459 1 2

11 531 32 639 1 5

11 532 16 860 1 5

11 533 32 876 1 2

11 534 8 1144 1 5

11 535 8 585 0 1

11 536 16 1143 1 4

11 537 32 833 1 5

11 538 16 766 1 5

11 539 32 987 0 3

11 540 32 1280 1 1

11 541 32 728 1 4

11 542 32 1021 1 4

11 543 32 643 1 3

11 544 8 583 1 2

11 545 16 603 1 3

11 546 32 605 1 2

11 547 32 788 1 1

11 548 8 1077 1 1

11 549 8 666 1 4

11 550 16 942 1 4

11 551 32 841 1 3

11 552 16 1402 1 2

11 553 32 899 1 4

11 554 32 1029 1 3

11 555 8 762 0 4

11 556 16 1172 0 2

11 557 16 639 1 5

11 558 32 954 1 2

11 559 8 659 1 4

11 560 32 717 1 1

11 561 32 1451 1 4

11 562 8 859 1 5

11 563 32 672 1 2

11 564 8 706 1 1

11 565 16 1124 1 1

11 566 16 1416 1 1

11 567 32 698 1 4

11 568 16 1760 1 5

11 569 16 1171 1 2

11 570 8 627 1 2

11 571 32 623 1 2

11 572 16 671 0 2

11 573 8 781 0 3

11 574 16 1105 0 1

11 575 8 790 1 1

11 576 16 930 1 1

11 577 16 697 0 1

11 578 16 1354 1 3

11 579 32 534 1 3

11 580 8 594 1 5

11 581 8 630 1 3

11 582 8 511 1 3

11 583 8 819 1 1

11 584 8 789 1 3

11 585 32 1191 1 1

11 586 32 597 0 1

11 587 16 820 1 3

11 588 8 727 1 4

11 589 16 1689 1 5

11 590 8 934 1 2

11 591 16 777 1 5

11 592 32 1003 0 5

11 593 32 1232 1 5

11 594 8 749 1 3

11 595 32 855 1 3

11 596 8 699 1 2

11 597 32 885 1 5

11 598 8 899 1 2

11 599 16 988 1 3

11 600 8 778 1 5

11 601 16 1469 0 4

11 602 8 981 1 5

11 603 32 1374 1 5

11 604 16 718 0 2

11 605 8 1005 1 4

11 606 32 781 1 2

11 607 16 815 1 5

11 608 16 738 1 4

11 609 32 610 0 5

11 610 32 821 1 1

11 611 32 577 1 4

11 612 16 1031 1 2

11 613 16 915 1 1

11 614 8 729 0 1

11 615 16 1168 1 3

11 616 16 1135 0 4

11 617 16 1041 1 2

11 618 32 888 1 5

11 619 16 745 1 2

11 620 16 699 1 5

11 621 16 565 1 3

11 622 16 685 1 4

11 623 8 596 1 2

11 624 16 621 1 1

11 625 32 677 1 2

11 626 16 938 1 2

11 627 32 906 1 1

11 628 16 601 1 5

11 629 16 951 0 2

11 630 8 790 1 3

11 631 8 1064 1 3

11 632 8 785 1 2

11 633 32 843 1 2

11 634 16 778 0 5

11 635 32 1117 1 3

11 636 16 1202 1 3

11 637 16 952 1 1

11 638 8 917 1 4

11 639 8 889 1 5

11 640 8 777 0 5

11 641 32 839 1 2

11 642 32 822 1 4

11 643 8 810 1 3

11 644 32 832 1 3

11 645 32 821 1 3

11 646 16 753 1 1

11 647 8 868 1 2

11 648 16 920 1 5

11 649 32 609 0 4

11 650 8 723 1 3

11 651 32 952 1 4

11 652 8 839 1 3

11 653 8 645 1 3

11 654 8 588 1 5

11 655 32 691 1 1

11 656 8 1471 1 2

11 657 8 971 1 1

11 658 8 706 1 3

11 659 32 726 1 3

11 660 16 734 1 4

11 661 32 911 0 5

11 662 32 678 1 1

11 663 32 1033 1 3

11 664 8 931 1 4

11 665 16 1416 0 4

11 666 8 749 1 5

11 667 16 1493 1 4

11 668 16 745 1 1

11 669 16 627 1 1

11 670 8 586 1 2

11 671 32 731 1 4

11 672 8 731 1 1

11 673 16 620 1 1

11 674 32 639 1 4

11 675 8 1291 1 1

11 676 32 1112 1 1

11 677 32 598 0 3

11 678 8 980 1 2

11 679 16 1085 1 3

11 680 16 833 1 2

11 681 32 1010 1 1

11 682 32 855 1 2

11 683 8 737 1 1

11 684 32 1063 1 2

11 685 32 590 0 4

11 686 32 593 0 3

11 687 32 774 1 5

11 688 32 767 1 5

11 689 8 625 1 4

11 690 32 581 1 4

11 691 16 806 1 5

11 692 32 972 1 1

11 693 32 639 1 2

11 694 16 1138 0 3

11 695 16 982 1 2

11 696 32 608 1 5

11 697 8 721 0 4

11 698 8 564 1 1

11 699 8 565 1 5

11 700 8 789 1 1

11 701 32 601 1 5

11 702 8 593 1 4

11 703 16 529 1 3

11 704 16 569 1 5

11 705 8 583 1 2

11 706 16 1026 0 3

11 707 8 831 1 4

11 708 8 550 1 4

11 709 8 1091 1 1

11 710 8 491 1 2

11 711 16 739 0 3

11 712 16 576 0 1

11 713 32 735 1 2

11 714 32 1061 1 1

11 715 16 616 1 5

11 716 8 899 1 5

11 717 8 1552 1 3

11 718 32 902 1 3

11 719 8 918 1 5

11 720 16 860 1 4

11 721 8 767 1 3

11 722 16 885 1 2

11 723 16 614 1 5

11 724 32 1185 1 4

11 725 8 770 1 3

11 726 32 739 1 2

11 727 8 805 0 5

11 728 8 696 0 5

11 729 32 1409 1 3

11 730 32 593 1 3

11 731 16 1187 1 4

11 732 32 714 1 2

11 733 16 948 1 5

11 734 32 1660 1 4

11 735 16 1021 1 3

11 736 32 823 1 5

11 737 32 603 0 2

11 738 32 702 1 1

11 739 32 1031 1 4

11 740 32 686 1 1

11 741 16 910 0 1

11 742 8 1583 1 1

11 743 32 715 1 3

11 744 16 1059 1 3

11 745 8 751 1 3

11 746 16 994 1 1

11 747 8 869 1 5

11 748 16 835 1 2

11 749 8 1318 1 2

11 750 32 685 0 5

11 751 16 924 0 2

11 752 8 1819 1 5

11 753 16 804 0 5

11 754 8 974 1 1

11 755 8 935 1 4

11 756 8 674 1 4

11 757 8 927 1 1

11 758 32 1053 1 4

11 759 8 756 1 2

11 760 16 537 0 1

11 761 8 914 0 2

11 762 32 1272 1 1

11 763 16 637 1 3

11 764 16 906 0 2

11 765 8 1029 1 2

11 766 8 1669 1 2

11 767 8 604 1 1

11 768 16 740 1 5

11 769 16 675 1 3

11 770 8 632 1 4

11 771 16 830 1 4

11 772 16 985 1 3

11 773 16 1371 1 5

11 774 16 700 0 1

11 775 8 1023 1 5

11 776 32 975 1 5

11 777 8 992 1 4

11 778 16 1331 0 2

11 779 16 756 1 4

11 780 16 831 1 5

11 781 16 1132 1 4

11 782 8 690 1 3

11 783 32 563 1 1

11 784 8 738 0 2

11 785 8 777 1 5

11 786 16 696 1 3

11 787 16 647 1 1

11 788 16 724 1 4

11 789 16 791 1 4

11 790 8 774 1 5

11 791 8 1176 1 3

11 792 16 864 1 3

11 793 32 620 1 4

11 794 32 786 1 5

11 795 16 559 1 2

11 796 8 832 0 1

11 797 8 851 1 3

11 798 32 813 1 1

11 799 8 551 0 1

11 800 32 1194 1 3

11 801 32 785 1 5

11 802 32 841 1 2

11 803 32 975 1 5

11 804 32 676 1 4

11 805 16 778 1 5

11 806 16 1103 1 3

11 807 8 711 1 1

11 808 16 631 0 1

11 809 8 588 0 3

11 810 32 819 1 5

11 811 32 646 1 5

11 812 8 700 1 2

11 813 8 1114 1 3

11 814 32 715 1 2

11 815 32 726 1 2

11 816 32 818 0 3

11 817 16 746 0 2

11 818 32 783 1 1

11 819 8 740 1 4

11 820 8 561 1 5

11 821 8 765 0 4

11 822 16 714 1 1

11 823 32 1132 1 1

11 824 16 873 0 2

11 825 32 1161 0 1

11 826 32 832 0 3

11 827 32 566 1 3

11 828 32 786 1 3

11 829 32 663 1 4

11 830 16 1052 0 1

11 831 16 989 1 4

11 832 32 622 1 2

11 833 32 701 1 2

11 834 16 1131 0 4

11 835 8 1486 1 1

11 836 8 1486 0 4

11 837 16 428 1 5

11 838 32 1282 1 4

11 839 8 530 1 2

11 840 8 581 1 4

11 841 8 929 1 1

11 842 32 764 1 5

11 843 8 688 1 2

11 844 16 689 1 1

11 845 8 618 0 3

11 846 16 1296 1 1

11 847 8 700 1 3

11 848 32 894 1 4

11 849 32 586 1 2

11 850 8 1245 0 1

11 851 8 1097 1 2

11 852 16 1809 1 1

11 853 32 793 1 4

11 854 32 1287 1 1

11 855 32 734 1 3

11 856 16 735 1 4

11 857 32 1025 1 1

11 858 32 780 1 4

11 859 32 687 1 2

11 860 16 1109 0 4

11 861 32 836 1 2

11 862 32 688 1 2

11 863 16 1046 1 1

11 864 8 798 1 2

11 865 16 643 1 5

11 866 16 801 0 5

11 867 8 1328 1 3

11 868 16 948 1 4

11 869 16 651 1 5

11 870 16 858 1 4

11 871 32 1009 1 3

11 872 8 1781 1 5

11 873 8 783 1 4

11 874 32 841 1 2

11 875 32 711 1 4

11 876 16 637 0 4

11 877 32 797 1 4

11 878 16 649 0 1

11 879 32 793 1 3

11 880 16 574 1 1

11 881 8 1159 1 5

11 882 8 1219 0 1

11 883 16 846 1 4

11 884 8 598 1 4

11 885 16 763 1 5

11 886 32 1525 1 3

11 887 32 1107 1 5

11 888 16 833 0 2

11 889 16 1166 1 3

11 890 32 615 1 3

11 891 8 742 1 1

11 892 16 735 0 3

11 893 8 921 1 2

11 894 8 773 1 2

11 895 16 625 0 2

11 896 32 1211 1 4

11 897 8 707 1 2

11 898 8 574 1 3

11 899 32 777 1 3

11 900 8 1108 1 3

11 901 32 659 1 5

11 902 32 900 1 3

11 903 16 1002 1 2

11 904 16 766 1 4

11 905 16 639 1 3

11 906 8 620 1 3

11 907 16 755 1 2

11 908 32 783 0 1

11 909 8 932 1 2

11 910 16 705 0 3

11 911 32 900 1 5

11 912 8 606 1 5

11 913 8 1533 1 4

11 914 8 607 0 1

11 915 16 870 1 3

11 916 16 891 1 5

11 917 16 793 1 5

11 918 32 790 1 2

11 919 32 839 0 3

11 920 32 617 1 4

11 921 16 1000 1 1

11 922 8 687 1 3

11 923 8 830 1 1

11 924 16 648 1 3

11 925 16 560 1 3

11 926 8 859 1 4

11 927 32 780 0 4

11 928 32 872 1 5

11 929 32 628 1 5

11 930 16 745 1 3

11 931 8 954 1 1

11 932 16 778 1 5

11 933 8 763 1 5

11 934 8 843 1 1

11 935 32 707 1 1

11 936 16 613 1 5

11 937 8 610 1 4

11 938 8 723 1 4

11 939 32 818 1 5

11 940 16 783 0 2

11 941 16 746 1 4

11 942 8 559 1 5

11 943 32 640 1 5

11 944 16 926 1 2

11 945 32 548 1 1

11 946 16 1211 0 2

11 947 16 749 0 1

11 948 32 797 1 1

11 949 8 657 1 4

11 950 8 1039 1 5

11 951 8 980 1 2

11 952 8 1221 1 3

11 953 32 682 1 2

11 954 32 956 1 1

11 955 32 803 1 1

11 956 16 738 0 2

11 957 8 876 1 5

11 958 8 1111 1 4

11 959 32 681 1 2

11 960 8 999 1 5

11 961 16 847 1 3

11 962 32 753 1 4

11 963 8 779 1 1

11 964 16 924 0 1

11 965 8 649 1 2

11 966 8 817 1 3

11 967 32 786 1 3

11 968 8 600 1 5

11 969 32 751 1 1

11 970 16 1171 1 3

11 971 16 581 1 2

11 972 32 687 1 3

11 973 16 829 0 2

11 974 8 644 1 3

11 975 8 582 0 4

11 976 16 1114 1 1

11 977 32 719 0 5

11 978 32 1053 1 1

11 979 8 660 1 2

11 980 32 861 1 1

11 981 32 600 1 4

11 982 8 593 0 3

11 983 8 1047 1 4

11 984 32 1086 0 1

11 985 32 1404 1 1

11 986 16 843 1 4

11 987 16 873 0 1

11 988 8 1525 1 1

11 989 32 1259 1 5

11 990 16 979 1 5

11 991 32 791 1 5

11 992 16 1057 1 5

11 993 16 773 1 4

11 994 16 807 0 2

11 995 32 704 1 5

11 996 16 596 1 5

11 997 32 881 0 2

11 998 16 1155 0 2

11 999 8 921 1 5

11 1000 16 697 1 4

11 1001 16 555 0 1

11 1002 8 747 1 4

11 1003 8 776 1 5

11 1004 8 724 1 2

11 1005 16 746 1 5

11 1006 8 661 1 5

11 1007 8 958 0 3

11 1008 8 1187 1 2

11 1009 8 918 1 5

11 1010 16 749 1 4

11 1011 8 793 1 4

11 1012 32 621 1 4

11 1013 8 784 0 1

11 1014 16 645 0 5

11 1015 16 911 1 4

11 1016 32 1073 1 2

11 1017 16 1075 0 4

11 1018 8 1043 1 4

11 1019 8 1613 0 1

11 1020 16 863 0 2

11 1021 8 740 1 3

11 1022 8 806 1 3

11 1023 32 701 1 4

11 1024 8 729 1 4

11 1025 8 682 0 3

11 1026 16 1585 1 3

11 1027 8 755 1 2

11 1028 32 696 1 5

11 1029 8 1487 1 1

11 1030 16 1372 1 3

11 1031 8 1220 1 1

11 1032 8 678 1 5

11 1033 16 875 1 4

11 1034 8 927 0 1

11 1035 8 792 1 4

11 1036 32 948 0 3

11 1037 8 692 1 2

11 1038 16 784 0 3

11 1039 32 1107 1 3

11 1040 32 951 1 2

11 1041 32 767 1 2

11 1042 16 1417 1 1

11 1043 8 765 1 3

11 1044 8 1059 1 2

11 1045 32 797 1 2

11 1046 16 1644 1 4

11 1047 16 536 0 1

11 1048 16 747 1 5

11 1049 32 838 1 2

11 1050 16 805 0 2

11 1051 32 926 1 2

11 1052 32 1101 1 5

11 1053 32 791 0 3

11 1054 32 854 1 1

11 1055 8 801 1 5

11 1056 32 632 1 1

11 1057 16 869 0 3

11 1058 16 854 0 3

11 1059 8 843 1 5

11 1060 16 994 1 1

11 1061 32 617 1 4

11 1062 32 563 1 4

11 1063 16 729 0 1

11 1064 16 738 1 5

11 1065 16 829 1 3

11 1066 8 937 1 4

11 1067 32 762 1 3

11 1068 32 1502 1 4

11 1069 32 645 0 3

11 1070 32 823 1 4

11 1071 16 555 0 2

11 1072 32 859 1 3

11 1073 32 1108 1 2

11 1074 32 1613 1 5

11 1075 8 716 1 2

11 1076 16 831 1 5

11 1077 32 835 1 5

11 1078 8 1364 1 1

11 1079 32 760 0 1

11 1080 16 1384 1 2

11 1081 16 889 1 4

11 1082 32 578 1 4

11 1083 16 579 0 1

11 1084 16 1380 1 5

11 1085 16 823 1 1

11 1086 16 945 1 2

11 1087 8 651 0 3

11 1088 32 732 1 3

11 1089 32 1309 1 5

11 1090 16 970 1 4

11 1091 8 741 1 1

11 1092 32 824 1 5

11 1093 8 556 1 4

11 1094 16 594 0 1

11 1095 8 955 1 1

11 1096 32 858 1 1

11 1097 16 1558 0 2

11 1098 32 852 0 1

11 1099 16 1014 1 5

11 1100 16 687 0 2

11 1101 8 810 1 2

11 1102 16 707 0 1

11 1103 8 892 1 3

11 1104 32 732 1 2

11 1105 8 679 1 5

11 1106 8 821 1 5

11 1107 16 777 1 3

11 1108 32 732 1 4

11 1109 8 679 1 2

11 1110 32 669 0 1

11 1111 8 1512 0 1

11 1112 16 904 0 5

11 1113 8 1061 1 2

11 1114 32 640 1 5

11 1115 32 640 1 4

11 1116 32 903 1 3

11 1117 32 597 1 4

11 1118 32 636 1 5

11 1119 16 658 1 3

11 1120 32 1010 1 2

11 1121 8 862 1 5

11 1122 8 768 1 3

11 1123 32 680 1 2

11 1124 32 839 1 4

11 1125 16 1186 1 1

11 1126 8 1042 0 4

11 1127 32 1466 1 4

11 1128 32 1048 0 2

11 1129 8 754 1 5

11 1130 32 793 1 5

11 1131 32 669 1 5

11 1132 16 1836 1 4

11 1133 8 905 0 1

11 1134 16 1204 1 4

11 1135 32 620 1 2

11 1136 16 568 0 1

11 1137 16 704 1 1

11 1138 32 780 1 2

11 1139 8 984 0 5

11 1140 8 1178 1 3

11 1141 32 895 1 5

11 1142 8 822 1 2

11 1143 32 930 1 2

11 1144 32 929 1 3

11 1145 16 1262 0 4

11 1146 32 682 0 5

11 1147 32 718 0 1

11 1148 8 1148 1 3

11 1149 32 619 1 3

11 1150 16 528 1 2

11 1151 16 554 1 5

11 1152 16 1052 1 3

11 1153 32 854 1 1

11 1154 8 965 1 2

11 1155 32 989 1 3

11 1156 32 906 0 3

11 1157 8 1704 0 4

11 1158 16 2061 1 5

11 1159 8 1237 1 2

11 1160 16 770 0 2

11 1161 32 920 1 1

11 1162 8 553 1 5

11 1163 32 1411 1 3

11 1164 8 857 1 1

11 1165 8 811 1 4

11 1166 8 788 0 1

11 1167 16 922 1 5

11 1168 16 1008 1 3

11 1169 32 848 1 4

11 1170 8 893 1 2

11 1171 32 911 1 1

11 1172 16 818 1 5

11 1173 8 1570 1 2

11 1174 16 1462 1 3

11 1175 8 814 1 1

11 1176 16 801 1 1

11 1177 32 855 1 1

11 1178 8 777 1 4

11 1179 8 723 0 4

11 1180 8 909 1 4

11 1181 16 747 1 5

11 1182 16 1938 0 2

11 1183 8 949 1 5

11 1184 16 1169 1 4

11 1185 8 754 1 3

11 1186 32 663 1 2

11 1187 8 961 1 1

11 1188 16 1047 1 3

11 1189 32 879 1 4

11 1190 8 743 1 3

11 1191 8 810 1 5

11 1192 32 616 0 3

11 1193 16 1175 1 3

11 1194 16 677 1 3

11 1195 16 724 1 4

11 1196 16 1099 1 4

11 1197 8 722 0 4

11 1198 8 1054 0 3

11 1199 16 1121 0 2

11 1200 16 1201 1 2

12 1 16 547 1 1

12 2 16 382 0 2

12 3 8 430 1 5

12 4 16 575 0 1

12 5 16 354 1 3

12 6 16 550 1 3

12 7 32 637 0 3

12 8 8 1090 1 3

12 9 32 1480 1 5

12 10 16 1194 1 4

12 11 32 1285 1 1

12 12 32 1079 1 2

12 13 16 880 0 1

12 14 32 982 1 2

12 15 16 1036 1 3

12 16 16 690 1 4

12 17 8 840 1 1

12 18 32 915 1 1

12 19 8 686 0 1

12 20 8 578 0 1

12 21 8 499 1 4

12 22 8 1042 1 5

12 23 16 1207 0 4

12 24 16 1157 0 1

12 25 8 782 0 1

12 26 8 1278 0 4

12 27 16 1592 1 3

12 28 8 726 0 2

12 29 16 1207 1 2

12 30 32 1228 0 3

12 31 16 2087 1 1

12 32 8 2263 1 1

12 33 32 1825 1 3

12 34 32 2022 1 5

12 35 16 1738 1 5

12 36 32 724 1 2

12 37 8 898 1 3

12 38 8 1255 1 2

12 39 32 1726 0 1

12 40 16 751 1 5

12 41 32 1520 1 3

12 42 16 1451 1 1

12 43 8 1306 1 4

12 44 8 917 1 3

12 45 16 1658 1 4

12 46 16 955 1 5

12 47 32 1253 0 5

12 48 32 820 1 4

12 49 32 1460 1 4

12 50 16 887 1 4

12 51 16 1647 1 4

12 52 16 950 1 5

12 53 8 1175 1 4

12 54 32 898 1 2

12 55 16 1495 1 5

12 56 32 679 1 2

12 57 16 896 0 2

12 58 8 1060 1 4

12 59 32 1211 1 4

12 60 32 829 1 4

12 61 32 1431 1 5

12 62 8 1312 1 1

12 63 8 1629 1 1

12 64 8 1343 1 4

12 65 16 720 1 5

12 66 16 546 1 2

12 67 8 988 1 2

12 68 8 858 1 5

12 69 32 935 1 1

12 70 32 1405 1 1

12 71 16 686 0 1

12 72 16 1218 1 2

12 73 16 1092 1 2

12 74 16 1180 0 1

12 75 8 904 1 2

12 76 32 837 1 1

12 77 8 642 1 3

12 78 8 840 1 5

12 79 8 680 1 3

12 80 16 840 1 3

12 81 8 1012 1 1

12 82 8 752 1 4

12 83 32 1339 0 4

12 84 8 806 1 2

12 85 32 1081 1 3

12 86 16 625 0 3

12 87 32 978 1 2

12 88 8 1229 1 4

12 89 8 1327 1 2

12 90 32 910 1 4

12 91 16 686 1 5

12 92 16 957 1 3

12 93 8 641 1 3

12 94 32 819 1 5

12 95 32 699 1 3

12 96 16 877 1 5

12 97 32 552 0 1

12 98 16 1357 0 3

12 99 32 937 1 3

12 100 8 773 1 5

12 101 8 1534 0 2

12 102 32 1719 0 1

12 103 8 628 1 3

12 104 8 934 1 5

12 105 32 750 1 2

12 106 8 705 1 5

12 107 32 1191 1 4

12 108 32 2197 1 5

12 109 8 1171 1 3

12 110 16 2677 1 4

12 111 8 737 1 2

12 112 16 1276 1 2

12 113 16 822 0 2

12 114 16 1194 1 4

12 115 32 1066 1 4

12 116 32 1915 1 5

12 117 32 902 0 2

12 118 8 671 0 5

12 119 32 1874 1 5

12 120 32 1015 1 3

12 121 8 1057 1 3

12 122 16 918 1 5

12 123 16 1087 0 3

12 124 16 1000 1 5

12 125 8 961 1 5

12 126 16 761 1 5

12 127 8 863 0 1

12 128 16 1503 1 1

12 129 16 957 1 5

12 130 8 934 1 4

12 131 32 889 1 5

12 132 16 862 1 2

12 133 16 826 1 2

12 134 8 820 0 3

12 135 16 1710 1 3

12 136 8 1178 1 3

12 137 16 1352 1 1

12 138 8 723 1 5

12 139 32 1273 1 2

12 140 8 1012 1 2

12 141 16 1083 0 4

12 142 8 1475 1 4

12 143 32 926 0 1

12 144 32 786 1 4

12 145 8 1588 1 4

12 146 32 851 1 1

12 147 8 1031 1 1

12 148 16 883 1 1

12 149 32 1073 1 4

12 150 32 955 1 2

12 151 8 1269 1 4

12 152 8 707 1 4

12 153 16 1006 0 4

12 154 32 1150 1 3

12 155 16 756 1 5

12 156 16 918 1 5

12 157 8 2108 1 4

12 158 32 880 1 4

12 159 32 791 0 5

12 160 16 1783 0 1

12 161 16 790 0 4

12 162 8 837 1 1

12 163 32 1025 1 5

12 164 32 795 1 3

12 165 8 823 1 5

12 166 32 1245 1 4

12 167 16 1132 0 2

12 168 8 798 1 5

12 169 8 939 1 4

12 170 32 893 1 3

12 171 32 1254 1 2

12 172 8 1650 1 1

12 173 8 974 1 1

12 174 16 1264 1 4

12 175 8 986 1 2

12 176 32 1152 0 1

12 177 8 895 1 5

12 178 32 649 1 2

12 179 16 721 1 5

12 180 32 1150 1 5

12 181 32 1007 1 5

12 182 16 1234 1 1

12 183 16 1835 0 2

12 184 32 1328 1 5

12 185 16 1221 1 2

12 186 8 1100 1 1

12 187 16 1577 1 4

12 188 8 719 1 3

12 189 8 793 1 2

12 190 16 870 0 3

12 191 8 1026 1 1

12 192 32 1508 1 5

12 193 32 1382 1 4

12 194 32 1309 1 2

12 195 32 1558 1 2

12 196 32 896 1 4

12 197 16 1283 1 2

12 198 16 1079 1 5

12 199 8 750 1 3

12 200 8 754 1 3

12 201 32 1668 1 1

12 202 16 1693 1 1

12 203 8 730 1 3

12 204 16 636 0 2

12 205 16 1272 1 4

12 206 32 875 0 2

12 207 8 962 1 5

12 208 32 1039 1 4

12 209 32 1333 1 2

12 210 32 815 1 1

12 211 16 1006 1 3

12 212 16 1006 1 3

12 213 32 806 1 3

12 214 8 905 1 2

12 215 8 946 1 2

12 216 16 777 1 3

12 217 16 1176 1 1

12 218 32 1566 1 3

12 219 8 1441 1 4

12 220 8 1827 1 2

12 221 8 807 0 1

12 222 16 1352 0 1

12 223 32 1196 1 1

12 224 16 1119 1 3

12 225 32 1106 1 1

12 226 16 1477 1 2

12 227 32 1073 1 4

12 228 16 742 1 3

12 229 8 665 1 2

12 230 8 1408 1 5

12 231 16 2122 0 4

12 232 8 842 1 5

12 233 32 1668 1 3

12 234 32 866 1 3

12 235 32 1447 1 5

12 236 32 1760 1 1

12 237 16 1446 1 4

12 238 8 1156 1 2

12 239 32 1091 1 3

12 240 8 1217 1 3

12 241 32 1021 1 4

12 242 32 757 1 1

12 243 16 693 1 5

12 244 8 1206 1 4

12 245 32 634 1 3

12 246 32 971 1 3

12 247 32 935 1 2

12 248 32 901 1 2

12 249 8 926 1 2

12 250 8 1185 1 1

12 251 16 1134 1 1

12 252 32 1139 1 4

12 253 16 1749 1 4

12 254 8 761 1 2

12 255 16 1796 1 1

12 256 32 1416 1 1

12 257 16 966 1 2

12 258 8 981 0 5

12 259 16 1499 1 4

12 260 8 2370 1 1

12 261 8 1128 1 1

12 262 16 1007 1 5

12 263 8 1672 1 1

12 264 32 1619 1 3

12 265 8 823 1 5

12 266 32 789 1 2

12 267 16 1406 1 5

12 268 8 1017 1 3

12 269 32 1517 1 4

12 270 8 1022 1 2

12 271 32 1206 1 1

12 272 32 697 1 4

12 273 32 980 1 5

12 274 16 1431 1 4

12 275 8 1588 1 2

12 276 16 1434 1 5

12 277 32 1005 1 5

12 278 8 897 1 4

12 279 32 939 1 5

12 280 8 858 1 3

12 281 8 1160 1 5

12 282 16 1102 1 3

12 283 16 924 0 2

12 284 32 1121 1 3

12 285 32 1022 0 3

12 286 32 1680 1 4

12 287 16 1783 1 1

12 288 8 706 1 3

12 289 8 892 1 2

12 290 16 1357 0 2

12 291 16 1111 1 3

12 292 32 875 1 3

12 293 16 810 1 5

12 294 8 686 1 3

12 295 16 1691 1 5

12 296 8 1076 1 4

12 297 8 851 1 2

12 298 16 1619 1 3

12 299 8 820 1 3

12 300 8 717 1 3

12 301 8 778 1 3

12 302 16 1079 1 4

12 303 8 617 1 4

12 304 32 1555 1 1

12 305 8 1291 1 1

12 306 32 1040 1 5

12 307 8 1016 1 4

12 308 32 1275 1 5

12 309 32 1293 1 2

12 310 8 947 1 5

12 311 16 1495 0 2

12 312 16 896 0 3

12 313 32 1079 1 3

12 314 16 902 1 3

12 315 8 1256 1 4

12 316 32 1237 1 4

12 317 32 1473 1 1

12 318 16 980 1 3

12 319 16 974 1 5

12 320 8 1073 1 4

12 321 16 1486 1 2

12 322 16 1302 1 2

12 323 32 1677 1 1

12 324 16 1538 1 4

12 325 8 964 1 5

12 326 32 739 1 2

12 327 32 1117 1 5

12 328 16 808 1 4

12 329 32 1020 1 3

12 330 32 731 1 4

12 331 16 1502 1 3

12 332 16 1348 1 4

12 333 8 1011 1 1

12 334 16 1127 1 1

12 335 32 744 0 2

12 336 32 850 1 4

12 337 8 1368 1 2

12 338 8 1092 1 3

12 339 16 1030 0 1

12 340 16 981 1 5

12 341 32 1088 1 1

12 342 16 785 1 1

12 343 32 993 1 1

12 344 16 778 1 2

12 345 32 841 1 2

12 346 8 1094 1 1

12 347 16 1599 1 1

12 348 8 889 1 2

12 349 16 958 1 2

12 350 32 980 1 5

12 351 32 887 0 5

12 352 8 1135 1 5

12 353 16 1412 1 3

12 354 8 907 1 5

12 355 8 1428 1 4

12 356 8 1964 1 5

12 357 16 942 1 1

12 358 32 813 0 2

12 359 8 1001 1 1

12 360 16 992 1 4

12 361 16 855 1 5

12 362 16 793 0 4

12 363 32 1239 1 4

12 364 16 1236 1 4

12 365 8 897 1 3

12 366 32 851 1 2

12 367 32 1050 1 2

12 368 32 1009 1 4

12 369 32 1126 1 4

12 370 32 871 1 5

12 371 8 948 0 3

12 372 8 987 1 2

12 373 16 1818 1 1

12 374 16 1052 1 4

12 375 32 1551 1 3

12 376 8 970 0 1

12 377 16 1154 1 3

12 378 8 982 1 3

12 379 32 1779 1 1

12 380 32 1053 1 1

12 381 32 1116 1 5

12 382 8 768 1 5

12 383 16 984 1 5

12 384 8 2235 1 1

12 385 8 753 1 2

12 386 8 735 1 5

12 387 32 1212 1 5

12 388 16 1397 1 2

12 389 32 1278 1 3

12 390 32 725 1 5

12 391 8 1124 1 4

12 392 32 764 1 2

12 393 16 1057 1 5

12 394 32 1044 1 4

12 395 16 1758 1 4

12 396 16 1671 0 2

12 397 16 1382 1 3

12 398 8 735 1 5

12 399 16 1431 0 3

12 400 16 864 1 5

12 401 16 1401 0 2

12 402 8 746 1 3

12 403 8 1970 1 5

12 404 16 827 0 1

12 405 32 1233 1 3

12 406 32 1513 1 3

12 407 32 1348 1 1

12 408 16 1439 1 4

12 409 32 1116 1 2

12 410 16 625 1 5

12 411 32 847 1 4

12 412 16 1435 1 1

12 413 8 682 0 3

12 414 8 844 1 5

12 415 32 966 1 2

12 416 16 1171 1 2

12 417 32 1016 1 1

12 418 8 1028 1 4

12 419 16 784 1 3

12 420 32 1334 1 5

12 421 32 849 1 3

12 422 16 1341 1 4

12 423 32 937 1 4

12 424 16 1393 1 1

12 425 8 641 1 3

12 426 32 848 1 2

12 427 8 903 1 1

12 428 16 1399 1 1

12 429 16 1298 1 4

12 430 16 1355 1 5

12 431 32 1716 1 1

12 432 16 1190 1 4

12 433 32 844 0 4

12 434 8 1060 1 2

12 435 8 1142 1 1

12 436 32 1238 1 3

12 437 32 908 1 1

12 438 32 1310 1 3

12 439 8 1225 1 4

12 440 8 733 1 2

12 441 8 1089 1 4

12 442 8 897 1 2

12 443 32 940 1 2

12 444 16 726 1 1

12 445 16 743 1 3

12 446 16 649 1 5

12 447 16 992 1 3

12 448 8 798 1 2

12 449 16 1059 0 3

12 450 8 735 1 3

12 451 8 937 1 5

12 452 32 829 1 4

12 453 8 1088 1 2

12 454 16 926 1 2

12 455 32 903 1 5

12 456 16 834 1 3

12 457 8 767 1 2

12 458 8 1300 0 1

12 459 8 776 1 1

12 460 16 883 0 2

12 461 16 821 1 5

12 462 8 752 0 4

12 463 32 2056 1 5

12 464 8 701 1 4

12 465 8 1211 1 1

12 466 16 1305 0 1

12 467 32 1117 1 5

12 468 8 1214 1 1

12 469 32 1677 1 3

12 470 8 897 1 4

12 471 8 908 1 5

12 472 16 1525 1 1

12 473 32 1321 1 1

12 474 8 603 1 4

12 475 16 1408 0 2

12 476 32 1035 1 2

12 477 8 887 1 5

12 478 8 1952 1 3

12 479 16 998 1 2

12 480 32 1393 1 1

12 481 8 984 0 2

12 482 16 1466 1 5

12 483 16 1214 1 1

12 484 16 890 1 3

12 485 8 1509 1 2

12 486 8 820 1 5

12 487 32 1188 1 4

12 488 16 1869 1 1

12 489 32 1023 1 3

12 490 32 1042 1 5

12 491 8 690 1 3

12 492 16 668 1 5

12 493 32 888 1 2

12 494 32 855 1 5

12 495 32 779 1 5

12 496 8 885 1 2

12 497 32 1410 1 5

12 498 8 898 1 4

12 499 16 853 1 5

12 500 16 815 1 4

12 501 16 873 1 3

12 502 32 793 1 2

12 503 16 1053 1 4

12 504 8 895 1 4

12 505 16 1073 0 2

12 506 8 1016 1 4

12 507 32 723 1 3

12 508 8 1254 1 5

12 509 16 939 1 2

12 510 8 1472 1 1

12 511 8 957 1 1

12 512 8 1145 1 1

12 513 8 1423 1 4

12 514 8 890 1 4

12 515 32 1644 1 1

12 516 8 1003 1 3

12 517 32 1411 1 1

12 518 32 796 1 3

12 519 16 3092 0 1

12 520 16 1107 1 3

12 521 32 1383 1 5

12 522 32 736 1 1

12 523 32 796 1 3

12 524 8 848 1 5

12 525 8 713 1 5

12 526 16 920 1 3

12 527 8 767 1 5

12 528 8 719 1 5

12 529 16 1677 1 1

12 530 16 929 0 3

12 531 8 1120 1 2

12 532 16 810 1 3

12 533 32 1033 1 1

12 534 16 1863 0 2

12 535 32 1529 1 2

12 536 8 1081 1 2

12 537 16 1224 1 1

12 538 16 1044 0 2

12 539 16 1445 1 1

12 540 32 1005 1 4

12 541 8 1180 1 1

12 542 16 792 1 5

12 543 32 1531 1 1

12 544 8 724 1 5

12 545 16 981 0 2

12 546 8 899 1 2

12 547 16 917 1 1

12 548 8 747 1 2

12 549 16 1087 1 4

12 550 16 721 1 5

12 551 8 1009 1 1

12 552 32 873 0 4

12 553 8 865 1 4

12 554 16 999 0 2

12 555 8 657 1 3

12 556 8 1113 0 4

12 557 32 1191 1 2

12 558 16 1097 1 5

12 559 8 1163 1 1

12 560 16 1387 1 4

12 561 8 1207 1 1

12 562 32 1005 1 3

12 563 16 737 1 2

12 564 16 753 1 2

12 565 8 765 1 3

12 566 32 1098 1 2

12 567 32 1354 1 3

12 568 32 807 1 1

12 569 8 879 1 3

12 570 16 754 1 5

12 571 32 1115 1 2

12 572 8 960 1 1

12 573 32 1072 1 1

12 574 32 1058 1 2

12 575 32 877 1 4

12 576 16 755 1 5

12 577 32 1007 1 5

12 578 32 798 1 4

12 579 8 852 1 4

12 580 8 624 1 5

12 581 32 1017 0 3

12 582 32 1720 1 1

12 583 16 1163 1 3

12 584 32 818 1 5

12 585 8 756 1 3

12 586 32 819 0 3

12 587 16 1227 1 4

12 588 16 833 1 4

12 589 32 891 1 2

12 590 8 793 1 3

12 591 16 1397 1 4

12 592 16 967 1 3

12 593 32 881 1 4

12 594 16 1425 1 1

12 595 32 948 1 4

12 596 8 808 1 3

12 597 32 942 1 5

12 598 16 1249 1 4

12 599 8 686 1 2

12 600 32 804 1 4

12 601 16 1532 1 1

12 602 16 1082 1 2

12 603 32 1168 1 5

12 604 32 895 1 5

12 605 8 863 1 5

12 606 16 818 0 4

12 607 8 1056 1 4

12 608 8 1072 1 2

12 609 8 669 1 3

12 610 16 816 1 4

12 611 32 643 1 2

12 612 8 720 1 5

12 613 32 873 1 1

12 614 32 1114 1 3

12 615 8 809 1 3

12 616 32 1319 1 1

12 617 16 757 1 5

12 618 32 979 1 3

12 619 16 1156 1 4

12 620 16 1219 0 2

12 621 32 976 1 4

12 622 8 660 1 3

12 623 32 862 0 2

12 624 16 1177 1 4

12 625 16 1095 0 1

12 626 16 795 1 4

12 627 32 1270 1 1

12 628 16 1118 0 2

12 629 8 1116 0 2

12 630 16 1340 0 1

12 631 16 848 0 5

12 632 8 948 1 2

12 633 16 1178 0 4

12 634 16 618 1 5

12 635 16 1048 1 3

12 636 16 726 0 2

12 637 32 817 1 3

12 638 16 862 1 1

12 639 8 695 0 3

12 640 32 2012 1 5

12 641 8 1888 1 1

12 642 8 607 1 5

12 643 16 1121 0 1

12 644 32 1111 1 4

12 645 8 1181 1 5

12 646 16 569 1 5

12 647 8 856 1 1

12 648 8 829 1 4

12 649 8 792 1 4

12 650 8 684 1 2

12 651 16 766 1 5

12 652 16 883 1 2

12 653 32 1061 1 1

12 654 8 722 1 4

12 655 16 655 1 1

12 656 8 595 1 1

12 657 32 1188 1 1

12 658 32 691 1 3

12 659 8 1012 1 1

12 660 16 947 1 3

12 661 16 1789 0 1

12 662 32 1178 1 2

12 663 32 986 1 3

12 664 8 959 1 2

12 665 8 714 1 4

12 666 32 975 1 5

12 667 8 853 1 4

12 668 16 787 1 3

12 669 32 877 0 2

12 670 32 1433 1 4

12 671 8 679 0 1

12 672 8 896 1 3

12 673 8 803 1 5

12 674 16 964 1 2

12 675 32 780 1 3

12 676 32 1252 1 5

12 677 32 851 1 2

12 678 16 1028 1 1

12 679 32 1389 1 4

12 680 32 875 1 5

12 681 32 1685 1 1

12 682 16 975 1 5

12 683 32 703 0 1

12 684 16 1172 0 5

12 685 32 659 1 4

12 686 32 853 1 2

12 687 8 802 1 4

12 688 16 1070 1 3

12 689 8 1166 1 2

12 690 8 646 1 5

12 691 16 601 1 5

12 692 16 836 1 2

12 693 16 767 1 3

12 694 8 757 1 1

12 695 8 667 1 5

12 696 8 757 0 4

12 697 32 1029 1 2

12 698 16 1101 1 4

12 699 8 808 1 5

12 700 16 1983 0 2

12 701 8 718 1 3

12 702 32 1315 1 3

12 703 16 1024 0 3

12 704 8 1000 1 2

12 705 8 727 1 3

12 706 16 1434 0 3

12 707 8 870 1 2

12 708 32 1242 1 5

12 709 8 1799 1 1

12 710 32 1163 1 4

12 711 32 1166 1 4

12 712 16 788 1 3

12 713 32 980 0 1

12 714 8 782 1 1

12 715 8 839 1 3

12 716 32 1339 1 3

12 717 32 909 0 5

12 718 16 821 0 4

12 719 32 770 1 2

12 720 32 1152 1 4

12 721 16 730 0 3

12 722 32 1155 0 1

12 723 16 977 1 3

12 724 16 748 1 5

12 725 8 636 1 4

12 726 32 811 0 1

12 727 8 996 1 3

12 728 8 666 1 5

12 729 8 920 0 1

12 730 16 1201 1 1

12 731 8 1230 1 4

12 732 8 1449 1 2

12 733 32 1206 1 5

12 734 16 941 0 4

12 735 32 946 1 5

12 736 8 989 1 4

12 737 32 1368 1 4

12 738 32 872 1 4

12 739 16 1627 0 1

12 740 32 1145 1 2

12 741 16 1273 1 5

12 742 32 1118 1 2

12 743 16 1589 1 5

12 744 16 1376 0 1

12 745 8 767 1 5

12 746 16 1627 1 1

12 747 16 1078 0 2

12 748 16 1619 1 4

12 749 16 1030 1 5

12 750 16 2745 0 2

12 751 8 846 0 5

12 752 16 1877 1 1

12 753 16 1542 1 1

12 754 16 1129 1 5

12 755 32 999 1 3

12 756 8 808 1 5

12 757 32 1231 1 3

12 758 32 892 1 4

12 759 8 966 0 2

12 760 32 1621 1 5

12 761 32 1980 1 2

12 762 32 750 1 2

12 763 32 1249 0 1

12 764 32 1032 1 1

12 765 16 1531 1 4

12 766 16 1504 0 2

12 767 16 1583 1 3

12 768 16 1111 1 3

12 769 16 2166 0 2

12 770 16 897 1 3

12 771 32 1342 1 5

12 772 8 984 1 3

12 773 32 1526 1 5

12 774 8 1204 1 4

12 775 8 1450 1 5

12 776 16 1779 1 5

12 777 16 1164 0 2

12 778 16 1468 1 4

12 779 8 1833 1 1

12 780 8 847 1 4

12 781 8 1212 1 1

12 782 16 1122 0 4

12 783 32 1011 1 1

12 784 32 732 1 2

12 785 16 1593 1 4

12 786 32 848 1 4

12 787 32 1356 1 4

12 788 8 1120 1 1

12 789 32 1764 1 3

12 790 32 765 1 3

12 791 8 764 1 3

12 792 16 1243 1 2

12 793 8 770 1 1

12 794 32 1312 1 2

12 795 32 938 1 2

12 796 32 759 1 3

12 797 16 1098 1 3

12 798 8 784 1 3

12 799 16 881 0 3

12 800 32 1727 1 5

12 801 16 1223 1 1

12 802 16 889 1 3

12 803 16 872 1 4

12 804 8 639 1 4

12 805 8 1039 1 2

12 806 32 822 1 3

12 807 8 659 1 4

12 808 16 1172 0 2

12 809 32 990 1 2

12 810 16 1095 1 1

12 811 32 939 1 3

12 812 32 1084 1 4

12 813 32 884 1 4

12 814 8 818 1 2

12 815 8 1077 1 2

12 816 32 794 0 5

12 817 8 1129 1 5

12 818 32 913 1 1

12 819 32 1037 1 1

12 820 8 1050 1 1

12 821 8 715 1 3

12 822 16 983 1 5

12 823 16 611 1 5

12 824 8 764 1 2

12 825 8 1029 1 2

12 826 8 817 1 2

12 827 8 1233 1 1

12 828 32 1164 1 5

12 829 8 748 1 5

12 830 8 684 1 3

12 831 32 875 1 4

12 832 32 890 1 1

12 833 8 651 1 5

12 834 32 929 1 3

12 835 8 819 1 3

12 836 16 1057 0 4

12 837 16 1056 1 2

12 838 8 1502 1 1

12 839 8 706 1 3

12 840 8 1155 1 4

12 841 32 1115 1 2

12 842 32 666 1 4

12 843 8 774 1 2

12 844 32 888 1 2

12 845 32 887 0 1

12 846 16 1034 1 3

12 847 16 1110 1 4

12 848 16 702 0 2

12 849 32 1162 1 1

12 850 8 699 1 4

12 851 16 876 1 1

12 852 16 946 0 1

12 853 16 1438 1 2

12 854 8 907 1 2

12 855 16 1666 0 2

12 856 8 1295 1 1

12 857 8 1154 1 2

12 858 8 854 1 4

12 859 16 808 1 5

12 860 8 665 1 3

12 861 8 741 1 3

12 862 8 1406 1 3

12 863 16 662 0 2

12 864 32 1027 1 5

12 865 16 1294 0 4

12 866 16 1719 1 1

12 867 16 1085 1 5

12 868 8 776 1 3

12 869 32 835 1 2

12 870 16 872 1 5

12 871 16 799 1 5

12 872 8 753 1 4

12 873 32 1475 1 1

12 874 32 928 1 1

12 875 8 1087 1 2

12 876 8 727 1 4

12 877 16 1364 1 1

12 878 16 724 1 3

12 879 32 1577 1 2

12 880 32 965 1 1

12 881 8 684 1 3

12 882 32 779 1 5

12 883 8 704 1 5

12 884 32 930 1 3

12 885 16 1104 1 3

12 886 8 995 1 2

12 887 16 691 1 5

12 888 16 891 1 4

12 889 8 669 1 5

12 890 8 708 0 4

12 891 8 1089 1 3

12 892 8 787 1 4

12 893 16 1039 1 4

12 894 32 1035 1 5

12 895 8 937 1 2

12 896 8 1170 1 1

12 897 32 755 1 2

12 898 32 712 1 3

12 899 16 926 1 3

12 900 8 1332 0 1

12 901 8 1006 1 2

12 902 16 631 1 5

12 903 8 699 1 3

12 904 16 1140 1 1

12 905 8 1179 1 5

12 906 8 633 1 1

12 907 32 870 1 1

12 908 32 691 1 4

12 909 32 859 1 4

12 910 32 877 1 4

12 911 16 693 1 5

12 912 32 869 1 4

12 913 16 1575 1 3

12 914 8 864 1 5

12 915 16 1709 1 4

12 916 8 1527 1 1

12 917 8 832 1 2

12 918 32 1083 1 4

12 919 32 979 1 5

12 920 32 938 1 1

12 921 16 852 1 3

12 922 8 906 1 5

12 923 32 847 1 2

12 924 32 961 1 1

12 925 16 1110 0 2

12 926 32 1131 1 5

12 927 8 861 1 4

12 928 8 998 1 3

12 929 8 979 1 1

12 930 8 772 1 1

12 931 8 1238 1 5

12 932 32 962 1 3

12 933 32 1343 1 5

12 934 32 1383 1 5

12 935 32 945 0 3

12 936 16 1801 0 2

12 937 8 843 1 4

12 938 32 1050 1 3

12 939 32 650 1 4

12 940 8 994 1 5

12 941 32 1095 1 3

12 942 32 976 0 2

12 943 16 1252 1 2

12 944 32 830 1 3

12 945 16 891 1 3

12 946 16 1720 1 4

12 947 8 759 1 5

12 948 16 981 0 2

12 949 16 1509 1 4

12 950 16 802 1 4

12 951 8 1987 1 1

12 952 16 785 1 1

12 953 32 764 1 2

12 954 16 1402 1 1

12 955 32 753 0 4

12 956 16 1718 0 1

12 957 32 1313 1 5

12 958 16 895 1 3

12 959 16 881 1 5

12 960 32 1198 1 3

12 961 32 687 0 1

12 962 16 638 1 1

12 963 8 714 1 2

12 964 32 880 1 5

12 965 32 1342 1 4

12 966 8 887 1 2

12 967 8 577 1 3

12 968 8 802 1 4

12 969 32 940 1 3

12 970 8 1252 1 5

12 971 32 750 0 4

12 972 32 1106 1 3

12 973 16 1086 1 4

12 974 8 639 1 5

12 975 8 1231 1 1

12 976 32 1250 1 5

12 977 8 1758 1 1

12 978 16 1474 1 2

12 979 16 1053 1 3

12 980 8 777 1 4

12 981 16 1256 1 5

12 982 32 1472 1 2

12 983 8 662 1 3

12 984 8 641 1 4

12 985 8 630 1 5

12 986 16 1012 1 3

12 987 16 711 1 5

12 988 16 1107 0 2

12 989 8 1480 1 1

12 990 8 682 1 3

12 991 32 1137 1 3

12 992 8 1075 1 1

12 993 16 1063 0 4

12 994 32 1209 1 1

12 995 32 1374 1 1

12 996 16 1013 1 2

12 997 16 897 1 3

12 998 8 939 1 2

12 999 16 821 1 1

12 1000 16 777 0 4

12 1001 32 1186 1 4

12 1002 8 1000 1 1

12 1003 8 612 1 5

12 1004 8 1331 1 1

12 1005 32 904 1 3

12 1006 16 739 1 1

12 1007 8 1507 1 3

12 1008 16 829 1 3

12 1009 32 1360 1 4

12 1010 32 885 0 2

12 1011 32 645 1 3

12 1012 32 735 1 5

12 1013 16 826 1 4

12 1014 16 994 1 5

12 1015 16 708 0 4

12 1016 8 1742 1 3

12 1017 8 607 1 2

12 1018 32 780 0 2

12 1019 32 1153 1 2

12 1020 8 897 1 5

12 1021 8 845 1 1

12 1022 16 1556 1 1

12 1023 16 1638 1 4

12 1024 16 854 1 3

12 1025 32 1134 1 2

12 1026 16 666 1 5

12 1027 8 785 0 4

12 1028 16 1822 1 3

12 1029 8 1743 1 4

12 1030 16 1862 0 2

12 1031 8 1150 1 2

12 1032 16 1259 1 1

12 1033 16 1850 0 2

12 1034 16 1308 1 5

12 1035 8 1300 1 2

12 1036 32 1867 1 5

12 1037 16 1203 0 2

12 1038 32 1006 1 4

12 1039 32 1100 1 3

12 1040 16 1121 1 5

12 1041 8 838 1 5

12 1042 8 1217 1 5

12 1043 8 848 1 2

12 1044 32 1011 1 1

12 1045 32 936 1 4

12 1046 8 949 1 4

12 1047 16 950 0 1

12 1048 32 815 1 2

12 1049 8 1991 1 3

12 1050 32 2558 1 4

12 1051 32 860 0 1

12 1052 32 1459 1 2

12 1053 16 1478 1 3

12 1054 16 1145 0 4

12 1055 16 1106 1 5

12 1056 32 794 1 3

12 1057 32 1269 1 2

12 1058 16 658 1 5

12 1059 32 895 1 4

12 1060 8 944 1 2

12 1061 32 785 1 5

12 1062 8 680 1 4

12 1063 16 1432 1 2

12 1064 16 2893 0 2

12 1065 16 1306 0 1

12 1066 32 1162 1 3

12 1067 32 1758 1 5

12 1068 8 833 0 3

12 1069 8 776 1 5

12 1070 8 832 1 4

12 1071 16 1798 1 3

12 1072 32 1260 1 1

12 1073 16 1488 1 4

12 1074 32 692 1 1

12 1075 8 1225 1 3

12 1076 32 987 1 5

12 1077 32 1598 1 1

12 1078 16 1323 1 1

12 1079 8 1257 0 1

12 1080 32 1109 1 5

12 1081 8 1385 1 5

12 1082 16 1412 1 1

12 1083 8 1118 1 2

12 1084 32 1314 1 5

12 1085 16 710 1 5

12 1086 16 1260 1 4

12 1087 16 743 1 5

12 1088 32 1866 1 1

12 1089 16 667 1 3

12 1090 32 988 0 2

12 1091 16 1640 0 1

12 1092 16 1802 0 2

12 1093 32 1053 1 3

12 1094 8 604 1 2

12 1095 16 1138 0 4

12 1096 16 1260 0 2

12 1097 16 1191 1 4

12 1098 32 783 1 3

12 1099 8 571 1 4

12 1100 8 732 1 2

12 1101 32 1962 1 2

12 1102 8 769 1 4

12 1103 16 1295 1 4

12 1104 32 1168 1 5

12 1105 16 2282 0 2

12 1106 8 1134 1 4

12 1107 32 1667 1 2

12 1108 8 728 1 4

12 1109 16 1125 1 3

12 1110 8 686 1 3

12 1111 8 710 1 3

12 1112 8 835 1 4

12 1113 32 1078 1 4

12 1114 32 1042 1 5

12 1115 32 1033 1 1

12 1116 8 1108 1 1

12 1117 32 1281 1 2

12 1118 8 802 1 3

12 1119 32 785 1 4

12 1120 8 915 1 2

12 1121 32 866 1 1

12 1122 8 866 1 4

12 1123 8 686 1 3

12 1124 8 702 1 2

12 1125 32 945 1 3

12 1126 32 717 1 4

12 1127 16 680 1 3

12 1128 8 634 1 4

12 1129 32 946 1 3

12 1130 8 945 1 5

12 1131 8 891 1 4

12 1132 32 900 0 5

12 1133 8 920 1 3

12 1134 8 863 1 1

12 1135 16 1000 1 2

12 1136 16 1158 1 1

12 1137 16 860 0 1

12 1138 16 969 1 1

12 1139 8 635 1 5

12 1140 32 985 1 1

12 1141 32 774 1 4

12 1142 8 602 0 5

12 1143 8 1101 1 1

12 1144 16 1754 1 3

12 1145 16 777 1 5

12 1146 16 2052 1 4

12 1147 32 803 1 5

12 1148 32 572 1 4

12 1149 32 972 1 2

12 1150 8 677 0 5

12 1151 8 780 1 5

12 1152 16 1603 1 1

12 1153 16 803 0 1

12 1154 32 901 1 3

12 1155 32 717 1 1

12 1156 16 627 1 5

12 1157 8 1400 1 5

12 1158 16 1819 1 5

12 1159 8 1219 1 2

12 1160 32 799 1 4

12 1161 16 1403 0 1

12 1162 32 1768 0 2

12 1163 32 824 1 3

12 1164 32 796 1 2

12 1165 32 691 0 4

12 1166 8 2031 1 1

12 1167 16 1107 1 2

12 1168 32 1025 1 1

12 1169 8 938 1 2

12 1170 32 1312 0 3

12 1171 16 970 1 2

12 1172 16 1180 1 3

12 1173 16 1421 1 4

12 1174 8 840 1 2

12 1175 8 1446 1 1

12 1176 16 1004 1 5

12 1177 32 972 1 1

12 1178 8 575 1 3

12 1179 8 1660 1 1

12 1180 16 1099 1 3

12 1181 16 663 1 2

12 1182 32 846 1 4

12 1183 32 717 1 1

12 1184 8 596 0 5

12 1185 16 1875 1 4

12 1186 8 1470 1 1

12 1187 16 1478 0 2

12 1188 32 2462 1 2

12 1189 32 809 1 5

12 1190 16 1017 0 5

12 1191 8 1032 1 3

12 1192 32 1547 1 3

12 1193 8 830 1 3

12 1194 16 1131 1 3

12 1195 16 1172 1 5

12 1196 16 1524 1 4

12 1197 32 925 1 5

12 1198 8 1312 1 1

12 1199 32 927 1 5

12 1200 16 1180 1 3

13 1 32 862 1 3

13 2 8 497 1 5

13 3 16 671 0 4

13 4 16 698 1 1

13 5 32 821 1 4

13 6 8 1037 1 2

13 7 8 513 1 3

13 8 8 774 1 3

13 9 32 805 1 1

13 10 32 718 1 1

13 11 8 1016 1 1

13 12 8 745 1 2

13 13 32 771 1 5

13 14 16 590 0 2

13 15 32 644 1 3

13 16 32 637 1 2

13 17 16 584 1 2

13 18 16 561 1 5

13 19 16 526 0 1

13 20 8 717 1 4

13 21 8 562 0 2

13 22 32 1007 0 3

13 23 32 1035 1 4

13 24 16 822 1 3

13 25 32 945 1 5

13 26 8 1323 1 4

13 27 16 1154 0 3

13 28 8 958 1 5

13 29 16 834 1 5

13 30 32 600 1 2

13 31 32 686 1 5

13 32 16 594 0 4

13 33 16 839 1 3

13 34 8 878 0 1

13 35 32 892 1 3

13 36 16 863 0 4

13 37 8 727 1 4

13 38 8 954 1 2

13 39 8 793 1 3

13 40 8 639 1 2

13 41 16 849 1 5

13 42 16 596 0 1

13 43 16 488 0 1

13 44 32 1116 1 1

13 45 8 529 1 1

13 46 8 627 1 5

13 47 32 858 1 1

13 48 32 1146 0 5

13 49 16 662 0 2

13 50 8 681 1 1

13 51 32 121 0 1

13 52 32 961 1 4

13 53 16 1030 1 2

13 54 32 987 1 2

13 55 16 815 1 3

13 56 32 752 1 2

13 57 32 205 0 3

13 58 8 846 1 3

13 59 32 679 1 2

13 60 32 713 1 3

13 61 8 1394 1 1

13 62 32 836 1 2

13 63 16 1278 1 5

13 64 16 736 1 4

13 65 8 518 1 5

13 66 16 645 1 1

13 67 8 474 0 2

13 68 32 667 1 5

13 69 8 767 1 5

13 70 32 931 1 3

13 71 32 711 1 3

13 72 16 661 0 4

13 73 8 800 1 5

13 74 8 844 0 4

13 75 32 1143 1 5

13 76 16 899 1 4

13 77 16 721 0 2

13 78 16 949 0 5

13 79 8 936 1 3

13 80 16 655 1 3

13 81 8 738 1 5

13 82 8 552 1 4

13 83 8 553 1 3

13 84 32 632 1 4

13 85 8 967 1 4

13 86 32 599 1 5

13 87 16 954 1 4

13 88 16 1035 0 3

13 89 32 1222 1 2

13 90 32 595 1 1

13 91 8 1001 1 5

13 92 32 906 1 1

13 93 8 725 1 2

13 94 16 988 1 5

13 95 16 1054 1 5

13 96 16 797 0 2

13 97 16 306 0 1

13 98 8 1732 1 1

13 99 32 1683 1 2

13 100 32 718 1 4

13 101 16 699 1 3

13 102 32 783 1 5

13 103 16 768 1 1

13 104 8 755 0 3

13 105 16 403 1 3

13 106 8 491 1 3

13 107 16 334 1 1

13 108 16 328 1 2

13 109 16 419 1 2

13 110 32 756 1 4

13 111 16 523 1 4

13 112 8 341 0 1

13 113 8 379 0 2

13 114 32 842 1 4

13 115 16 866 1 5

13 116 8 749 1 4

13 117 32 941 1 4

13 118 8 740 1 1

13 119 32 857 0 1

13 120 8 303 1 4

13 121 8 233 1 2

13 122 16 240 0 1

13 123 8 692 0 3

13 124 32 1807 1 2

13 125 16 727 1 2

13 126 32 654 1 3

13 127 16 371 1 1

13 128 32 510 1 1

13 129 8 749 0 4

13 130 16 1428 1 1

13 131 8 1326 1 5

13 132 8 884 1 3

13 133 32 596 1 3

13 134 16 261 1 4

13 135 8 401 1 2

13 136 32 282 0 1

13 137 16 345 0 5

13 138 8 766 0 1

13 139 32 855 1 4

13 140 32 4 1 2

13 141 16 848 1 3

13 142 32 1649 1 1

13 143 32 788 1 4

13 144 8 1364 1 1

13 145 8 683 1 3

13 146 16 1104 1 1

13 147 32 820 1 2

13 148 32 751 1 3

13 149 32 820 1 1

13 150 32 772 1 5

13 151 32 621 1 2

13 152 8 807 1 2

13 153 16 1068 1 3

13 154 8 810 1 5

13 155 8 642 1 1

13 156 8 802 1 2

13 157 16 524 1 5

13 158 32 1232 1 5

13 159 16 675 0 1

13 160 32 371 1 4

13 161 16 828 1 1

13 162 32 649 1 5

13 163 32 808 1 5

13 164 16 958 0 2

13 165 32 831 1 4

13 166 16 914 1 4

13 167 32 886 1 5

13 168 32 830 1 3

13 169 8 880 1 3

13 170 16 1014 1 5

13 171 8 617 1 4

13 172 32 773 1 1

13 173 16 572 1 4

13 174 32 707 0 4

13 175 16 499 1 2

13 176 32 377 1 5

13 177 8 580 1 5

13 178 32 726 1 3

13 179 8 822 1 4

13 180 16 480 0 2

13 181 8 699 1 5

13 182 8 764 1 4

13 183 8 764 1 3

13 184 16 930 0 2

13 185 16 756 1 5

13 186 16 704 0 2

13 187 8 1109 1 5

13 188 16 609 0 2

13 189 16 821 1 1

13 190 8 1418 1 1

13 191 8 931 1 4

13 192 16 778 1 3

13 193 16 719 1 5

13 194 8 635 0 2

13 195 8 885 1 1

13 196 32 751 1 3

13 197 8 716 1 4

13 198 8 553 1 5

13 199 8 560 0 2

13 200 32 611 1 5

13 201 8 1493 0 5

13 202 8 929 1 1

13 203 16 713 0 1

13 204 16 721 1 3

13 205 8 1157 1 4

13 206 32 884 1 2

13 207 8 675 0 4

13 208 8 929 1 2

13 209 32 1437 1 3

13 210 16 530 1 5

13 211 16 636 0 4

13 212 32 643 1 4

13 213 32 882 1 2

13 214 8 1119 1 3

13 215 16 540 0 3

13 216 32 1158 1 4

13 217 32 643 0 1

13 218 16 758 0 4

13 219 16 1172 1 3

13 220 8 579 1 2

13 221 8 503 1 3

13 222 32 649 1 5

13 223 8 1302 1 1

13 224 16 575 1 4

13 225 16 348 1 5

13 226 8 673 1 3

13 227 32 836 1 1

13 228 32 976 1 2

13 229 32 961 1 1

13 230 32 592 1 4

13 231 32 582 0 3

13 232 16 682 0 4

13 233 8 1108 1 1

13 234 16 823 1 5

13 235 16 1088 1 3

13 236 16 577 0 4

13 237 8 1107 1 5

13 238 16 1048 1 3

13 239 32 918 1 2

13 240 16 580 0 2

13 241 8 653 1 5

13 242 8 1435 1 3

13 243 16 584 1 1

13 244 16 652 0 1

13 245 8 822 1 1

13 246 8 562 1 5

13 247 16 1103 1 5

13 248 32 848 1 2

13 249 16 688 1 2

13 250 32 1237 1 5

13 251 32 650 1 5

13 252 16 602 1 1

13 253 8 823 1 1

13 254 8 715 1 2

13 255 16 722 0 4

13 256 32 697 1 2

13 257 8 1218 0 1

13 258 32 709 1 2

13 259 32 810 0 5

13 260 16 1304 1 3

13 261 8 1578 0 2

13 262 16 1708 1 2

13 263 16 912 0 4

13 264 16 899 1 3

13 265 8 596 1 5

13 266 32 831 1 4

13 267 32 743 1 1

13 268 32 730 1 4

13 269 16 529 1 2

13 270 16 593 0 5

13 271 32 852 1 4

13 272 32 1083 1 1

13 273 16 1060 1 5

13 274 16 787 0 4

13 275 8 828 1 4

13 276 16 1695 1 1

13 277 32 757 0 3

13 278 32 807 1 4

13 279 16 512 0 5

13 280 32 711 0 2

13 281 16 1063 1 3

13 282 8 583 1 2

13 283 8 540 1 3

13 284 8 634 1 3

13 285 32 734 1 5

13 286 8 530 1 4

13 287 32 729 1 5

13 288 16 696 0 4

13 289 16 568 1 3

13 290 16 394 1 5

13 291 16 433 0 4

13 292 8 462 0 2

13 293 16 541 1 2

13 294 8 843 1 2

13 295 16 689 1 2

13 296 32 983 1 2

13 297 32 1011 0 1

13 298 16 1045 0 1

13 299 16 866 0 2

13 300 32 1904 1 1

13 301 8 1436 1 1

13 302 8 661 1 4

13 303 32 578 1 2

13 304 16 504 1 2

13 305 16 763 0 1

13 306 16 763 1 3

13 307 32 575 1 3

13 308 32 625 1 3

13 309 8 702 1 2

13 310 32 496 1 2

13 311 16 826 1 5

13 312 8 730 0 5

13 313 16 632 1 4

13 314 8 584 1 5

13 315 32 652 1 5

13 316 32 447 1 5

13 317 32 414 0 3

13 318 16 758 0 1

13 319 32 618 1 4

13 320 8 426 1 3

13 321 16 1333 1 3

13 322 32 1224 1 1

13 323 8 862 1 1

13 324 32 604 0 4

13 325 8 1214 1 4

13 326 32 647 1 3

13 327 8 693 1 1

13 328 16 1246 1 4

13 329 16 465 1 1

13 330 8 697 0 1

13 331 32 723 1 3

13 332 8 712 1 2

13 333 32 714 1 4

13 334 8 740 1 4

13 335 32 832 1 2

13 336 8 599 1 3

13 337 16 548 1 3

13 338 8 349 1 2

13 339 8 566 0 5

13 340 32 487 0 1

13 341 32 2330 1 1

13 342 32 684 1 5

13 343 8 626 0 3

13 344 16 708 0 2

13 345 32 894 1 1

13 346 8 658 1 5

13 347 16 714 1 3

13 348 8 502 1 3

13 349 32 389 1 4

13 350 16 630 0 4

13 351 8 713 0 4

13 352 32 755 0 3

13 353 8 863 1 4

13 354 16 653 1 5

13 355 8 736 1 4

13 356 8 886 1 3

13 357 8 598 1 1

13 358 32 846 1 3

13 359 8 569 1 5

13 360 16 537 0 5

13 361 8 539 1 4

13 362 8 503 1 5

13 363 16 451 1 2

13 364 32 191 0 5

13 365 8 917 1 1

13 366 32 660 0 5

13 367 32 822 1 4

13 368 32 570 1 3

13 369 32 468 0 1

13 370 32 955 0 1

13 371 16 1309 1 3

13 372 16 532 0 2

13 373 16 541 1 1

13 374 32 513 1 4

13 375 32 583 1 5

13 376 32 436 0 2

13 377 16 692 0 5

13 378 8 798 1 5

13 379 8 890 1 1

13 380 8 1000 1 1

13 381 32 743 1 5

13 382 8 538 1 4

13 383 8 4017 1 5

13 384 16 1283 1 2

13 385 8 340 0 2

13 386 8 767 1 3

13 387 32 584 1 1

13 388 16 650 0 4

13 389 32 687 1 4

13 390 32 996 1 4

13 391 16 929 1 5

13 392 32 432 1 2

13 393 8 892 1 2

13 394 8 399 1 4

13 395 32 1053 1 4

13 396 8 1259 0 1

13 397 8 894 1 3

13 398 32 557 1 2

13 399 32 563 1 4

13 400 8 531 1 4

13 401 8 1068 1 5

13 402 8 842 1 3

13 403 32 531 1 3

13 404 32 248 1 1

13 405 8 728 1 4

13 406 32 1171 1 3

13 407 16 572 1 3

13 408 16 709 1 4

13 409 32 753 1 2

13 410 8 790 1 4

13 411 32 614 1 5

13 412 16 997 0 2

13 413 8 643 1 5

13 414 32 1297 0 1

13 415 32 974 1 3

13 416 32 1388 1 1

13 417 8 619 1 4

13 418 16 724 0 4

13 419 16 609 0 2

13 420 16 892 1 4

13 421 32 485 1 1

13 422 8 486 1 3

13 423 32 671 1 4

13 424 32 716 1 2

13 425 32 664 1 4

13 426 16 675 1 4

13 427 16 838 0 4

13 428 16 638 0 5

13 429 16 910 1 3

13 430 16 708 0 1

13 431 8 1144 1 3

13 432 32 603 1 3

13 433 16 495 0 1

13 434 8 682 1 5

13 435 32 620 1 2

13 436 8 593 1 3

13 437 16 531 0 1

13 438 16 767 1 5

13 439 32 608 0 1

13 440 16 611 1 5

13 441 16 760 1 2

13 442 32 656 1 3

13 443 16 905 0 1

13 444 32 863 0 3

13 445 16 1101 0 1

13 446 8 1102 1 2

13 447 8 634 1 5

13 448 8 678 0 1

13 449 8 1225 0 1

13 450 32 1109 1 3

13 451 8 736 1 2

13 452 16 817 1 5

13 453 32 1608 1 2

13 454 16 1173 1 5

13 455 16 679 0 4

13 456 16 1190 1 3

13 457 32 636 1 2

13 458 16 816 0 1

13 459 16 1228 1 3

13 460 32 704 1 5

13 461 8 682 1 4

13 462 8 847 0 2

13 463 8 1049 1 2

13 464 16 934 1 3

13 465 8 921 1 2

13 466 32 530 1 5

13 467 8 489 1 3

13 468 16 442 1 2

13 469 16 403 1 3

13 470 16 470 1 4

13 471 16 476 1 1

13 472 8 568 0 1

13 473 8 617 1 2

13 474 16 653 1 5

13 475 8 574 0 1

13 476 32 836 1 5

13 477 16 594 1 3

13 478 16 714 0 2

13 479 8 595 1 5

13 480 8 551 0 3

13 481 32 1115 1 4

13 482 8 657 1 3

13 483 16 499 1 2

13 484 32 710 1 3

13 485 8 412 0 1

13 486 16 706 0 4

13 487 8 829 1 4

13 488 8 1557 0 1

13 489 32 1084 1 3

13 490 8 858 1 3

13 491 32 885 1 5

13 492 16 661 1 4

13 493 32 1135 1 1

13 494 16 975 1 3

13 495 8 642 0 2

13 496 16 1034 1 5

13 497 8 535 1 3

13 498 8 572 1 4

13 499 8 391 1 1

13 500 32 962 1 2

13 501 8 524 1 2

13 502 32 589 1 3

13 503 32 881 1 5

13 504 32 985 1 3

13 505 8 635 1 5

13 506 16 325 0 3

13 507 32 1380 1 1

13 508 32 853 0 2

13 509 8 701 1 2

13 510 8 902 0 1

13 511 8 843 1 4

13 512 16 503 1 2

13 513 32 587 1 4

13 514 32 710 1 4

13 515 32 604 1 5

13 516 32 596 1 2

13 517 32 332 1 4

13 518 16 358 1 5

13 519 16 366 0 2

13 520 16 537 0 2

13 521 8 775 1 3

13 522 16 179 0 3

13 523 32 815 0 2

13 524 32 914 1 1

13 525 8 1012 0 1

13 526 8 862 1 2

13 527 8 705 1 2

13 528 16 841 1 5

13 529 32 815 0 4

13 530 8 1330 0 1

13 531 32 772 0 5

13 532 16 2232 1 4

13 533 16 924 1 4

13 534 16 1400 1 4

13 535 32 696 1 3

13 536 8 777 1 4

13 537 32 626 1 1

13 538 32 982 1 1

13 539 16 745 1 5

13 540 8 866 1 5

13 541 16 743 0 2

13 542 32 708 1 5

13 543 8 770 1 5

13 544 32 903 1 4

13 545 32 1180 1 1

13 546 16 758 0 1

13 547 32 560 1 2

13 548 16 498 0 3

13 549 8 886 1 5

13 550 32 855 1 3

13 551 16 695 1 5

13 552 8 659 1 5

13 553 16 741 0 1

13 554 8 443 1 5

13 555 16 507 0 4

13 556 16 580 1 3

13 557 32 455 1 1

13 558 16 515 0 2

13 559 8 579 1 4

13 560 8 153 0 3

13 561 32 1056 1 5

13 562 32 665 1 2

13 563 16 518 1 1

13 564 8 571 0 3

13 565 8 770 1 4

13 566 8 564 1 4

13 567 32 891 1 4

13 568 16 1255 0 4

13 569 16 789 0 5

13 570 16 1752 1 1

13 571 8 884 1 4

13 572 32 715 0 3

13 573 8 1026 1 5

13 574 8 964 1 1

13 575 16 574 1 3

13 576 8 737 0 2

13 577 16 742 0 1

13 578 16 876 1 3

13 579 16 703 1 1

13 580 8 508 1 5

13 581 8 441 1 3

13 582 16 434 1 3

13 583 16 432 0 4

13 584 32 894 1 5

13 585 8 697 0 1

13 586 8 694 0 3

13 587 32 637 1 2

13 588 32 489 1 4

13 589 32 704 0 2

13 590 8 1045 1 2

13 591 16 686 0 2

13 592 16 769 1 1

13 593 16 649 1 5

13 594 8 619 1 2

13 595 32 674 1 3

13 596 16 880 0 1

13 597 16 515 0 2

13 598 32 578 1 1

13 599 16 709 1 5

13 600 32 663 1 5

13 601 32 792 1 2

13 602 32 812 1 5

13 603 8 977 1 4

13 604 16 1048 0 2

13 605 16 867 0 4

13 606 32 688 1 5

13 607 8 393 1 2

13 608 16 791 1 5

13 609 16 1272 0 5

13 610 32 1271 1 3

13 611 16 780 1 3

13 612 8 703 1 1

13 613 16 462 1 3

13 614 8 935 1 4

13 615 16 635 1 3

13 616 8 778 1 3

13 617 8 882 1 1

13 618 16 547 1 5

13 619 16 480 0 2

13 620 32 706 1 5

13 621 8 735 1 1

13 622 16 595 1 1

13 623 32 1037 1 4

13 624 32 605 1 4

13 625 32 543 0 1

13 626 32 607 0 3

13 627 16 1189 0 1

13 628 16 1156 1 2

13 629 16 952 1 4

13 630 32 835 0 1

13 631 32 603 0 5

13 632 16 1051 1 4

13 633 16 1264 1 5

13 634 16 658 0 1

13 635 8 886 0 1

13 636 32 841 1 2

13 637 8 999 1 3

13 638 16 855 1 5

13 639 8 539 1 4

13 640 32 121 1 3

13 641 8 627 1 2

13 642 16 551 1 5

13 643 32 814 1 1

13 644 32 784 1 4

13 645 16 1303 1 4

13 646 16 633 1 1

13 647 8 774 0 1

13 648 8 817 1 2

13 649 32 606 0 4

13 650 8 1048 0 3

13 651 32 1444 1 4

13 652 32 749 1 2

13 653 8 621 1 2

13 654 32 757 1 5

13 655 32 716 1 1

13 656 8 688 0 5

13 657 16 936 0 1

13 658 16 804 1 1

13 659 8 590 1 5

13 660 8 604 1 5

13 661 8 867 1 2

13 662 16 927 0 2

13 663 8 496 1 3

13 664 8 1140 1 2

13 665 32 495 1 4

13 666 16 587 1 3

13 667 32 537 1 4

13 668 32 437 1 3

13 669 32 427 1 2

13 670 32 807 1 5

13 671 32 989 0 1

13 672 16 663 0 4

13 673 8 811 1 1

13 674 32 650 0 3

13 675 16 1275 1 1

13 676 16 1061 1 3

13 677 8 761 1 1

13 678 8 838 1 4

13 679 8 484 1 4

13 680 8 463 1 4

13 681 16 696 1 2

13 682 32 896 1 5

13 683 32 701 1 4

13 684 16 764 1 2

13 685 8 1152 1 5

13 686 16 536 1 3

13 687 8 755 0 2

13 688 32 1115 1 1

13 689 32 832 1 2

13 690 8 755 1 1

13 691 16 481 1 2

13 692 8 727 1 4

13 693 16 680 1 5

13 694 8 456 1 5

13 695 32 459 0 2

13 696 32 1039 1 1

13 697 32 900 0 3

13 698 16 1373 0 2

13 699 16 793 1 3

13 700 16 1259 1 4

13 701 8 937 1 5

13 702 16 900 1 4

13 703 8 687 0 3

13 704 8 831 1 3

13 705 8 1324 1 5

13 706 32 761 0 3

13 707 32 1200 1 5

13 708 8 554 1 3

13 709 16 689 1 5

13 710 8 618 1 4

13 711 32 509 1 1

13 712 32 810 1 2

13 713 16 632 1 4

13 714 8 520 1 2

13 715 32 409 1 2

13 716 32 675 1 3

13 717 16 632 1 3

13 718 16 551 0 1

13 719 8 805 1 5

13 720 8 796 1 3

13 721 32 871 0 2

13 722 16 600 0 2

13 723 8 1103 1 1

13 724 8 649 1 3

13 725 16 563 0 3

13 726 8 231 0 3

13 727 16 759 0 5

13 728 16 1425 1 4

13 729 8 692 1 4

13 730 32 1034 1 4

13 731 32 775 1 5

13 732 8 593 1 2

13 733 8 696 0 5

13 734 8 827 1 5

13 735 8 738 1 3

13 736 8 669 0 1

13 737 16 621 0 1

13 738 16 1257 1 1

13 739 8 943 0 5

13 740 8 894 1 2

13 741 16 649 1 3

13 742 32 722 1 1

13 743 16 1158 1 1

13 744 8 763 1 2

13 745 32 803 1 5

13 746 8 1193 1 1

13 747 32 1028 1 3

13 748 32 730 1 3

13 749 8 785 1 5

13 750 16 807 1 3

13 751 32 688 1 1

13 752 8 450 1 2

13 753 16 762 0 4

13 754 8 650 1 4

13 755 32 490 1 4

13 756 8 595 1 3

13 757 16 603 1 5

13 758 32 1102 1 2

13 759 16 879 0 5

13 760 8 770 1 4

13 761 16 694 1 3

13 762 32 688 1 5

13 763 16 926 1 4

13 764 8 536 1 2

13 765 8 803 0 5

13 766 32 1218 1 2

13 767 8 844 1 4

13 768 32 1225 1 5

13 769 8 915 0 5

13 770 32 891 1 1

13 771 32 722 1 5

13 772 32 754 0 4

13 773 16 1684 1 1

13 774 32 845 1 4

13 775 16 716 1 4

13 776 8 991 1 1

13 777 16 534 0 2

13 778 8 755 1 3

13 779 32 656 1 3

13 780 16 562 0 2

13 781 16 654 0 2

13 782 8 745 1 2

13 783 16 619 1 5

13 784 8 701 1 4

13 785 32 480 1 1

13 786 32 395 1 1

13 787 32 411 0 2

13 788 16 598 0 2

13 789 32 716 0 1

13 790 8 1592 1 1

13 791 16 947 1 3

13 792 16 611 0 5

13 793 32 718 1 2

13 794 16 741 0 1

13 795 16 1170 1 4

13 796 32 667 0 4

13 797 32 816 0 5

13 798 16 949 1 1

13 799 8 749 1 3

13 800 16 1214 1 1

13 801 32 870 1 4

13 802 32 563 1 3

13 803 8 507 0 1

13 804 32 603 1 3

13 805 8 564 1 2

13 806 16 605 1 5

13 807 8 731 1 4

13 808 32 707 0 2

13 809 16 1678 1 4

13 810 16 688 0 2

13 811 16 1064 1 4

13 812 32 722 1 4

13 813 16 650 0 2

13 814 8 1114 1 3

13 815 32 726 1 3

13 816 32 996 1 1

13 817 16 506 1 3

13 818 8 664 1 5

13 819 32 809 1 5

13 820 8 658 0 1

13 821 16 973 1 5

13 822 8 760 1 5

13 823 32 584 1 1

13 824 32 690 0 3

13 825 32 833 1 2

13 826 32 760 1 5

13 827 8 982 0 1

13 828 32 1201 1 3

13 829 16 745 1 3

13 830 8 1161 1 4

13 831 16 609 0 1

13 832 8 1915 0 4

13 833 16 1136 1 2

13 834 8 1001 1 2

13 835 32 887 1 2

13 836 16 576 0 3

13 837 16 630 1 5

13 838 32 859 1 4

13 839 8 465 0 3

13 840 16 955 0 4

13 841 8 828 1 5

13 842 32 872 1 3

13 843 32 621 1 5

13 844 16 535 1 3

13 845 8 616 0 1

13 846 32 732 0 1

13 847 32 1297 1 5

13 848 32 1099 1 1

13 849 16 859 1 5

13 850 8 1020 1 2

13 851 8 735 1 5

13 852 16 912 0 3

13 853 32 767 1 1

13 854 8 608 1 5

13 855 8 521 1 3

13 856 8 780 1 3

13 857 32 834 0 2

13 858 8 1041 1 3

13 859 32 601 1 2

13 860 32 507 1 1

13 861 16 496 0 3

13 862 32 995 1 1

13 863 16 711 1 5

13 864 8 541 1 4

13 865 32 904 1 4

13 866 16 570 0 3

13 867 32 676 1 5

13 868 32 643 1 2

13 869 32 1382 1 3

13 870 16 695 1 4

13 871 8 808 1 5

13 872 32 921 1 4

13 873 16 552 1 5

13 874 16 493 1 1

13 875 32 44 0 3

13 876 8 630 1 4

13 877 16 529 1 5

13 878 32 356 0 3

13 879 32 596 1 5

13 880 16 570 1 5

13 881 8 858 0 2

13 882 8 559 1 5

13 883 16 588 1 2

13 884 16 507 0 2

13 885 8 768 1 3

13 886 8 459 0 2

13 887 32 617 1 4

13 888 8 634 1 3

13 889 8 734 1 5

13 890 8 815 0 1

13 891 16 510 1 1

13 892 16 874 1 1

13 893 8 342 0 4

13 894 16 551 1 4

13 895 32 453 0 4

13 896 8 617 1 3

13 897 32 715 1 1

13 898 8 506 1 4

13 899 32 536 1 2

13 900 16 531 1 4

13 901 16 444 1 2

13 902 16 468 0 2

13 903 8 374 0 4

13 904 8 716 1 5

13 905 16 791 1 4

13 906 16 826 1 3

13 907 16 480 0 1

13 908 32 1216 1 4

13 909 32 740 1 3

13 910 8 418 0 2

13 911 32 703 0 2

13 912 16 707 1 4

13 913 32 982 1 2

13 914 8 759 1 2

13 915 8 671 1 1

13 916 16 971 0 1

13 917 16 702 1 2

13 918 8 790 1 4

13 919 32 1159 1 5

13 920 8 758 1 3

13 921 16 588 1 4

13 922 16 563 0 1

13 923 32 822 0 5

13 924 8 834 1 1

13 925 32 838 1 4

13 926 8 538 1 2

13 927 16 494 0 2

13 928 8 388 0 1

13 929 32 1001 1 3

13 930 32 584 1 4

13 931 16 539 0 3

13 932 32 537 1 3

13 933 16 843 1 5

13 934 8 613 1 4

13 935 32 608 1 5

13 936 32 720 1 5

13 937 32 432 1 1

13 938 8 454 1 4

13 939 16 654 0 4

13 940 16 579 1 2

13 941 16 431 0 1

13 942 8 623 1 1

13 943 16 600 1 4

13 944 32 1063 1 3

13 945 16 881 1 1

13 946 16 347 0 3

13 947 16 595 0 3

13 948 8 1112 1 1

13 949 8 837 1 2

13 950 32 661 1 4

13 951 32 774 1 2

13 952 32 506 0 1

13 953 16 428 0 2

13 954 8 1541 1 1

13 955 8 867 1 5

13 956 8 1028 1 3

13 957 32 746 1 2

13 958 16 592 1 5

13 959 8 639 1 2

13 960 16 836 1 5

13 961 8 587 0 4

13 962 32 641 1 5

13 963 8 505 0 3

13 964 16 1060 1 1

13 965 32 750 0 5

13 966 16 933 1 3

13 967 16 976 0 1

13 968 8 1033 1 3

13 969 8 1279 1 1

13 970 8 734 1 5

13 971 32 708 1 2

13 972 32 640 0 3

13 973 16 773 1 1

13 974 8 668 0 1

13 975 32 1165 1 4

13 976 32 797 1 3

13 977 8 731 1 5

13 978 32 711 1 4

13 979 16 606 1 5

13 980 32 881 1 2

13 981 8 1165 1 1

13 982 32 712 1 2

13 983 8 507 1 2

13 984 16 836 1 4

13 985 16 780 0 2

13 986 8 1153 1 4

13 987 16 795 1 2

13 988 16 801 1 3

13 989 8 876 1 3

13 990 8 602 1 5

13 991 16 453 0 2

13 992 8 740 1 4

13 993 8 721 1 5

13 994 16 818 1 5

13 995 16 715 1 5

13 996 32 649 1 5

13 997 16 728 1 5

13 998 32 691 1 3

13 999 16 669 0 1

13 1000 8 895 0 3

13 1001 8 983 0 5

13 1002 16 1405 1 3

13 1003 8 1101 1 5

13 1004 8 984 1 4

13 1005 8 896 1 3

13 1006 16 763 1 4

13 1007 32 689 1 4

13 1008 8 601 0 4

13 1009 16 677 1 5

13 1010 8 1070 0 2

13 1011 8 1174 1 2

13 1012 8 910 1 4

13 1013 32 1075 1 1

13 1014 32 670 1 4

13 1015 16 584 1 4

13 1016 16 742 0 4

13 1017 32 638 1 2

13 1018 16 613 1 5

13 1019 16 616 1 5

13 1020 8 1140 1 2

13 1021 32 850 1 2

13 1022 16 558 1 1

13 1023 32 1052 1 1

13 1024 8 665 1 2

13 1025 32 684 1 5

13 1026 32 594 1 1

13 1027 16 592 0 5

13 1028 32 672 1 3

13 1029 32 759 1 2

13 1030 16 608 0 4

13 1031 32 838 0 2

13 1032 8 705 1 4

13 1033 16 1349 1 3

13 1034 16 761 0 2

13 1035 32 1051 1 1

13 1036 8 656 1 3

13 1037 8 689 1 3

13 1038 16 941 1 1

13 1039 32 699 1 3

13 1040 8 844 1 2

13 1041 16 961 0 2

13 1042 8 1048 0 1

13 1043 32 980 1 3

13 1044 16 1113 1 3

13 1045 32 1022 1 1

13 1046 8 758 0 3

13 1047 32 1368 0 5

13 1048 8 900 0 1

13 1049 32 858 1 4

13 1050 8 530 1 4

13 1051 32 630 1 1

13 1052 16 556 0 3

13 1053 8 1030 1 1

13 1054 32 1078 1 1

13 1055 16 830 1 2

13 1056 16 439 1 1

13 1057 8 496 0 1

13 1058 16 459 1 4

13 1059 16 517 1 2

13 1060 16 407 0 3

13 1061 8 682 1 5

13 1062 16 573 1 4

13 1063 32 413 1 3

13 1064 16 441 0 2

13 1065 32 769 1 3

13 1066 8 545 1 5

13 1067 16 458 0 1

13 1068 8 701 0 1

13 1069 32 615 1 4

13 1070 32 833 1 5

13 1071 16 974 1 4

13 1072 32 853 1 2

13 1073 32 440 1 1

13 1074 32 554 1 4

13 1075 32 355 1 5

13 1076 8 845 1 2

13 1077 32 642 1 4

13 1078 16 557 1 3

13 1079 32 545 1 5

13 1080 8 547 1 2

13 1081 8 655 1 4

13 1082 8 543 0 3

13 1083 32 600 1 2

13 1084 8 482 0 1

13 1085 8 1232 1 1

13 1086 8 955 1 5

13 1087 16 900 1 3

13 1088 8 515 0 2

13 1089 32 691 1 3

13 1090 8 974 1 4

13 1091 32 1145 1 4

13 1092 8 780 1 2

13 1093 16 599 1 5

13 1094 16 463 1 5

13 1095 32 690 1 2

13 1096 16 628 0 3

13 1097 8 1778 1 1

13 1098 8 519 1 4

13 1099 16 575 1 1

13 1100 16 689 0 1

13 1101 16 733 1 5

13 1102 16 494 1 5

13 1103 16 436 1 5

13 1104 8 442 1 1

13 1105 16 431 0 2

13 1106 32 669 1 4

13 1107 32 826 1 1

13 1108 16 1347 1 3

13 1109 8 1226 1 3

13 1110 16 845 1 3

13 1111 16 613 0 2

13 1112 16 517 0 3

13 1113 16 861 0 1

13 1114 16 2080 1 4

13 1115 16 1135 1 4

13 1116 32 673 1 2

13 1117 16 699 1 2

13 1118 32 995 0 4

13 1119 16 1400 1 1

13 1120 8 974 1 5

13 1121 8 1130 1 2

13 1122 8 677 1 1

13 1123 32 817 0 1

13 1124 32 720 1 3

13 1125 8 741 0 1

13 1126 32 1263 1 5

13 1127 8 896 1 3

13 1128 32 730 1 3

13 1129 16 715 1 2

13 1130 16 633 1 4

13 1131 16 452 1 4

13 1132 8 429 0 3

13 1133 16 732 0 4

13 1134 32 1300 0 5

13 1135 16 1239 1 3

13 1136 16 592 1 3

13 1137 16 550 1 2

13 1138 8 666 1 2

13 1139 16 458 0 2

13 1140 8 406 0 3

13 1141 32 510 0 2

13 1142 32 645 1 5

13 1143 8 447 0 5

13 1144 32 1175 1 1

13 1145 8 605 1 4

13 1146 8 409 1 5

13 1147 8 329 0 4

13 1148 8 302 1 2

13 1149 16 316 1 2

13 1150 32 363 1 2

13 1151 32 321 0 3

13 1152 32 978 1 5

13 1153 8 620 0 4

13 1154 32 1248 1 4

13 1155 16 713 1 5

13 1156 8 963 0 4

13 1157 32 964 1 1

13 1158 32 931 1 5

13 1159 16 573 1 3

13 1160 16 372 0 1

13 1161 32 1005 1 4

13 1162 16 575 1 1

13 1163 32 592 1 3

13 1164 8 495 0 2

13 1165 32 460 1 3

13 1166 16 511 1 4

13 1167 32 701 1 4

13 1168 32 673 1 4

13 1169 8 482 1 2

13 1170 32 412 0 2

13 1171 32 785 0 1

13 1172 32 511 0 1

13 1173 8 873 1 3

13 1174 16 473 0 2

13 1175 16 665 0 4

13 1176 8 1106 1 1

13 1177 16 701 1 1

13 1178 8 720 1 3

13 1179 8 775 1 5

13 1180 32 1167 0 1

13 1181 8 1356 1 2

13 1182 8 666 1 5

13 1183 16 1173 1 1

13 1184 32 807 1 1

13 1185 8 664 1 5

13 1186 8 638 1 3

13 1187 32 697 1 3

13 1188 32 697 1 5

13 1189 32 832 1 5

13 1190 32 654 1 5

13 1191 32 426 1 4

13 1192 8 1078 1 1

13 1193 8 906 0 4

13 1194 16 618 1 5

13 1195 32 524 1 2

13 1196 32 773 1 2

13 1197 16 597 0 5

13 1198 16 2478 0 4

13 1199 32 855 0 3

13 1200 8 813 1 5

Key:

Column 1: Participant number

Column 2: Trial number

Column 3: Pattern size (number of sections)

Column 4: Reaction time (milliseconds)

Column 5: Error (0) or correct response (1)

Column 6: Ratio (1) 1:1.468 (2) 1:1.518 (3) 1:1.568 (4) 1:1.618 (5) 1:1.668
