# Supplementary material for: The Golden Section as Optical Limitation
Source: PLoS One. 2015 Jul 8;10(7):e0131045. doi: 10.1371/journal.pone.0131045 (PMC4495923; doi:10.1371/journal.pone.0131045)
Supplement: S2 Dataset — (DOCX) [file pone.0131045.s002.docx]

**Dataset S2. Mean Reaction Times Experiment 2**

| **c1** | **c2** | **c3** | **c4** | **c5** | **c6** | **c7** | **c8** | **c9** | **c10** | **c11** | **c12** | **c13** | **c14** | **c15** | **S** |
| --- | --- | --- | --- | --- | --- | --- | --- | --- | --- | --- | --- | --- | --- | --- | --- |
| 1355.11 | 1277.22 | 1070.43 | 968.42 | 917.66 | 1553.83 | 1392.11 | 1118.92 | 1328.02 | 930.65 | 1257.22 | 1094.31 | 1019.91 | 1096.17 | 1110.06 | 1 |
| 1483.94 | 1363.15 | 1241.27 | 1429.38 | 1387.62 | 1589.79 | 1510.09 | 1637.38 | 1788.35 | 1335.33 | 1572.31 | 1477.33 | 1461.68 | 1384.58 | 1342.98 | 2 |
| 1529.60 | 1246.04 | 1228.47 | 1619.66 | 1150.37 | 2611.77 | 1771.24 | 1548.82 | 1844.21 | 1298.76 | 1542.32 | 1489.10 | 1306.17 | 1360.19 | 1521.18 | 3 |
| 1031.90 | 927.02 | 904.37 | 926.85 | 863.45 | 1503.84 | 1122.14 | 1070.22 | 1151.83 | 906.27 | 1074.40 | 1009.86 | 939.92 | 882.67 | 963.09 | 4 |
| 903.25 | 801.52 | 770.74 | 849.87 | 713.50 | 1086.00 | 951.53 | 896.87 | 1000.87 | 762.30 | 1052.93 | 895.53 | 835.06 | 807.43 | 878.08 | 5 |
| 1474.16 | 1182.87 | 1058.45 | 1095.07 | 905.97 | 2081.50 | 1515.51 | 1442.56 | 1513.57 | 1184.14 | 1473.37 | 1360.59 | 1072.54 | 1036.90 | 1080.00 | 6 |
| 1088.77 | 961.48 | 853.10 | 902.37 | 792.09 | 1323.17 | 1188.66 | 1093.54 | 1282.51 | 892.88 | 1124.71 | 1052.66 | 922.01 | 920.17 | 1051.71 | 7 |

Key Row 1:

C1 4-paired sections 1:1.468 ratio

C2 4-paired sections 1:1.518 ratio

C3 4 paired sections 1:1.568 ratio

C4 4-paired sections 1:1.618 ratio

C5 4-paired sections 1:1.668 ratio

C6 8-paired sections 1:1.468 ratio

C7 8-paired sections 1:1.518 ratio

C8 8 paired sections 1:1.568 ratio

C9 8-paired sections 1:1.618 ratio

C10 8-paired sections 1:1.668 ratio

C11 16-paired sections 1:1.468 ratio

C12 16-paired sections 1:1.518 ratio

C13 16 paired sections 1:1.568 ratio

C14 16-paired sections 1:1.618 ratio

C15 16-paired sections 1:1.668 ratio

S = Participant number

1 1 2 8 1 2 820 0618 2

1 2 2 32 0 2 2237 0668 2

1 3 2 8 1 2 1710 0518 1

1 4 1 16 1 1 1483 0618 1

1 5 1 32 1 1 1508 0468 1

1 6 2 16 1 2 1724 0468 2

1 7 2 32 0 2 1026 0568 2

1 8 1 16 1 1 1258 0468 1

1 9 2 16 1 2 1669 0568 2

1 10 2 32 0 2 1005 0518 2

1 11 2 32 1 2 1380 0568 2

1 12 1 8 1 1 1103 0668 2

1 13 1 16 1 1 1385 0568 1

1 14 1 8 1 1 781 0668 1

1 15 1 32 1 1 1065 0518 1

1 16 1 16 1 1 2660 0468 1

1 17 1 32 1 1 1247 0568 1

1 18 1 8 1 1 1101 0618 1

1 19 1 16 1 1 1282 0668 1

1 20 1 16 1 1 1590 0518 1

1 21 1 16 1 1 997 0568 1

1 22 1 16 1 1 875 0568 1

1 23 1 32 1 1 1915 0518 1

1 24 2 32 1 2 1348 0568 2

1 25 2 8 1 2 1979 0518 1

1 26 1 8 1 1 972 0568 2

1 27 1 32 1 1 1557 0518 1

1 28 2 16 1 2 1997 0518 2

1 29 1 16 1 1 1280 0468 1

1 30 2 8 1 2 1580 0518 2

1 31 2 32 1 2 1682 0618 2

1 32 1 32 0 1 802 0618 1

1 33 2 8 1 2 1132 0618 2

1 34 2 32 1 2 1062 0468 2

1 35 2 8 1 2 1622 0568 2

1 36 1 16 1 1 1634 0568 1

1 37 1 32 1 1 1570 0568 1

1 38 2 32 1 2 915 0618 2

1 39 1 16 1 1 1390 0518 1

1 40 2 32 1 2 1050 0618 2

1 41 2 32 1 2 1309 0568 2

1 42 2 16 1 2 1364 0568 2

1 43 2 8 1 2 934 0668 2

1 44 1 32 1 1 1312 0568 1

1 45 2 32 1 2 2524 0468 2

1 46 2 32 1 2 1202 0468 2

1 47 1 8 1 1 1812 0468 1

1 48 1 32 1 1 2219 0668 1

1 49 2 8 1 2 2122 0468 2

1 50 1 8 1 1 1122 0518 2

1 51 2 8 0 2 1626 0468 2

1 52 1 32 1 1 860 0668 1

1 53 2 32 1 2 1077 0668 2

1 54 2 32 0 2 1496 0518 2

1 55 1 8 1 1 977 0568 1

1 56 2 16 1 2 2182 0518 2

1 57 1 16 1 1 853 0668 1

1 58 2 16 1 2 1142 0668 2

1 59 2 16 1 2 768 0668 2

1 60 1 32 1 1 1367 0468 1

1 61 2 16 0 2 2627 0618 2

1 62 2 16 1 2 1563 0568 2

1 63 2 32 1 2 1553 0668 2

1 64 2 16 1 2 1563 0518 2

1 65 1 8 1 1 2563 0668 1

1 66 2 32 1 2 1049 0618 2

1 67 2 16 0 2 1544 0568 2

1 68 2 16 1 2 2018 0468 2

1 69 1 16 1 1 1169 0518 1

1 70 1 8 1 1 1515 0518 2

1 71 1 16 1 1 1000 0668 1

1 72 2 8 1 2 1846 0468 2

1 73 2 16 0 2 1927 0618 2

1 74 1 8 1 1 1488 0668 2

1 75 2 32 1 2 2738 0518 2

1 76 2 16 1 2 2604 0568 2

1 77 2 8 1 2 1538 0468 2

1 78 2 8 1 2 885 0618 2

1 79 2 16 1 2 1623 0468 2

1 80 1 16 1 1 1379 0618 1

1 81 1 32 1 1 2185 0668 1

1 82 1 32 1 1 941 0518 1

1 83 1 8 1 1 956 0518 2

1 84 2 8 1 2 1430 0468 2

1 85 2 8 1 2 2378 0568 2

1 86 2 8 1 2 1393 0568 1

1 87 1 32 1 1 1457 0518 1

1 88 2 16 1 2 3392 0518 2

1 89 1 8 1 1 1147 0668 2

1 90 1 16 1 1 2099 0568 1

1 91 1 8 1 1 1115 0468 1

1 92 2 16 1 2 2208 0618 2

1 93 2 16 1 2 4302 0468 2

1 94 2 16 1 2 957 0668 2

1 95 1 16 1 1 974 0668 1

1 96 2 32 1 2 2025 0568 2

1 97 2 16 1 2 1966 0668 2

1 98 1 16 1 1 1348 0518 1

1 99 1 8 1 1 3130 0618 1

1 100 1 8 1 1 977 0618 1

1 101 1 8 1 1 810 0468 1

1 102 2 16 1 2 1450 0618 2

1 103 2 8 1 2 1008 0668 2

1 104 2 16 1 2 1318 0518 2

1 105 1 8 1 1 936 0568 2

1 106 1 8 1 1 2252 0518 1

1 107 1 8 1 1 902 0568 1

1 108 1 16 1 1 1500 0518 1

1 109 2 8 1 2 1363 0568 1

1 110 2 8 1 2 880 0668 2

1 111 1 32 1 1 1711 0618 1

1 112 2 8 1 2 2679 0568 2

1 113 1 16 1 1 1568 0568 1

1 114 1 16 1 1 1185 0568 1

1 115 1 8 1 1 1111 0618 1

1 116 2 16 0 2 1171 0468 2

1 117 1 32 1 1 1020 0618 1

1 118 2 16 1 2 3965 0518 2

1 119 2 32 1 2 1311 0518 2

1 120 1 8 1 1 1103 0518 1

1 121 2 16 1 2 1511 0618 2

1 122 2 32 1 2 1197 0668 2

1 123 2 32 1 2 1397 0668 2

1 124 1 32 1 1 1791 0468 1

1 125 2 8 0 2 2871 0518 2

1 126 1 8 1 1 1164 0668 2

1 127 2 32 0 2 800 0568 2

1 128 2 16 1 2 1115 0618 2

1 129 1 8 1 1 1065 0568 1

1 130 1 32 1 1 2585 0468 1

1 131 1 16 1 1 1111 0518 1

1 132 2 32 1 2 1799 0468 2

1 133 2 16 1 2 2082 0468 2

1 134 2 8 1 2 976 0568 2

1 135 2 32 0 2 966 0668 2

1 136 2 16 1 2 1447 0518 2

1 137 1 16 1 1 1833 0468 1

1 138 1 8 1 1 2002 0468 1

1 139 2 16 1 2 826 0668 2

1 140 1 8 1 1 962 0618 1

1 141 1 16 1 1 1428 0468 1

1 142 2 8 1 2 818 0568 2

1 143 2 32 1 2 1208 0618 2

1 144 1 32 0 1 873 0468 1

1 145 1 16 1 1 2238 0618 1

1 146 1 32 1 1 1089 0668 1

1 147 1 32 1 1 972 0668 1

1 148 1 32 1 1 751 0618 1

1 149 1 8 1 1 3389 0518 1

1 150 2 32 1 2 917 0518 2

1 151 2 8 1 2 1227 0668 1

1 152 1 32 0 1 1117 0518 1

1 153 2 8 1 2 1252 0618 2

1 154 1 32 1 1 1230 0568 1

1 155 1 16 1 1 1895 0468 1

1 156 1 16 1 1 1072 0518 1

1 157 1 16 1 1 1610 0618 1

1 158 2 8 1 2 846 0668 2

1 159 1 32 1 1 1700 0618 1

1 160 2 16 1 2 1208 0568 2

1 161 2 32 0 2 1031 0468 2

1 162 2 16 1 2 1275 0568 2

1 163 2 8 1 2 764 0668 1

1 164 2 32 1 2 1143 0668 2

1 165 2 8 1 2 860 0668 2

1 166 1 16 1 1 695 0668 1

1 167 1 32 1 1 1285 0618 1

1 168 1 8 0 1 1100 0468 1

1 169 1 32 1 1 878 0618 1

1 170 1 32 1 1 883 0518 1

1 171 2 32 0 2 923 0568 2

1 172 2 16 0 2 1558 0518 2

1 173 2 8 1 2 1201 0468 2

1 174 1 8 1 1 909 0468 1

1 175 1 8 1 1 1219 0518 1

1 176 1 8 1 1 2300 0468 1

1 177 2 32 0 2 2090 0468 2

1 178 2 16 0 2 2198 0618 2

1 179 1 32 1 1 1460 0618 1

1 180 2 16 1 2 1761 0468 2

1 181 2 32 0 2 2838 0518 2

1 182 2 32 1 2 1370 0618 2

1 183 2 8 1 2 1712 0568 2

1 184 1 32 1 1 1014 0568 1

1 185 1 8 1 1 1579 0568 2

1 186 1 32 1 1 1299 0668 1

1 187 2 16 1 2 845 0668 2

1 188 2 32 1 2 2021 0518 2

1 189 2 8 1 2 1655 0518 2

1 190 1 32 1 1 1351 0568 1

1 191 2 8 1 2 1144 0618 2

1 192 1 16 1 1 1559 0618 1

1 193 2 8 1 2 1375 0618 2

1 194 2 32 1 2 1475 0668 2

1 195 1 16 1 1 2954 0618 1

1 196 1 32 1 1 904 0518 1

1 197 2 8 1 2 819 0668 2

1 198 1 32 1 1 1657 0568 1

1 199 2 16 1 2 1448 0618 2

1 200 1 8 1 1 1340 0618 1

1 201 2 32 1 2 920 0618 2

1 202 1 16 1 1 4034 0468 1

1 203 2 32 1 2 1000 0518 2

1 204 2 8 0 2 1215 0468 2

1 205 2 32 1 2 1116 0468 2

1 206 1 16 1 1 1090 0568 1

1 207 2 8 1 2 1387 0618 2

1 208 1 16 1 1 897 0668 1

1 209 2 8 1 2 1755 0618 2

1 210 1 32 1 1 1979 0468 1

1 211 1 16 1 1 1755 0618 1

1 212 2 16 1 2 1009 0568 2

1 213 2 16 1 2 1721 0668 2

1 214 2 16 1 2 900 0668 2

1 215 1 32 0 1 2012 0468 1

1 216 1 16 1 1 3489 0618 1

1 217 1 8 1 1 1912 0618 1

1 218 1 16 1 1 768 0668 1

1 219 2 8 1 2 1266 0468 2

1 220 2 8 1 2 1493 0518 1

1 221 1 8 1 1 1293 0668 1

1 222 1 8 1 1 3010 0468 1

1 223 1 32 1 1 819 0568 1

1 224 2 8 1 2 1404 0518 2

1 225 1 8 1 1 1005 0568 1

1 226 1 8 1 1 812 0568 2

1 227 2 16 0 2 1683 0468 2

1 228 2 32 1 2 1473 0618 2

1 229 1 8 1 1 1934 0518 2

1 230 2 32 0 2 1258 0468 2

1 231 2 32 1 2 1354 0568 2

1 232 2 8 1 2 1080 0518 1

1 233 1 32 1 1 986 0668 1

1 234 1 8 1 1 1048 0618 1

1 235 1 32 1 1 789 0668 1

1 236 1 8 1 1 726 0668 1

1 237 1 16 1 1 1011 0668 1

1 238 1 32 1 1 1332 0468 1

1 239 1 16 1 1 937 0468 1

1 240 1 16 1 1 873 0518 1

1 241 1 8 1 1 654 0668 1

1 242 1 16 1 1 742 0568 1

1 243 2 8 1 2 846 0668 1

1 244 2 16 1 2 1602 0518 2

1 245 1 32 1 1 1087 0568 1

1 246 1 8 1 1 918 0518 2

1 247 1 32 1 1 1087 0618 1

1 248 2 32 1 2 1649 0618 2

1 249 1 8 1 1 1299 0468 1

1 250 1 32 1 1 1228 0518 1

1 251 2 16 1 2 1273 0568 2

1 252 1 8 1 1 1820 0668 1

1 253 2 16 1 2 872 0668 2

1 254 1 16 1 1 1032 0468 1

1 255 2 32 0 2 962 0468 2

1 256 2 16 1 2 2112 0618 2

1 257 1 8 1 1 1041 0618 1

1 258 2 32 1 2 1425 0468 2

1 259 1 8 1 1 990 0618 1

1 260 2 32 0 2 901 0518 2

1 261 1 32 1 1 1014 0518 1

1 262 2 16 1 2 2904 0518 2

1 263 2 8 1 2 852 0518 1

1 264 2 8 1 2 899 0668 2

1 265 2 32 1 2 1092 0518 2

1 266 2 8 1 2 756 0668 1

1 267 2 32 0 2 1462 0568 2

1 268 1 8 1 1 1318 0568 2

1 269 2 8 1 2 1571 0468 2

1 270 2 32 1 2 1062 0518 2

1 271 2 32 1 2 776 0568 2

1 272 1 32 1 1 835 0618 1

1 273 1 8 0 1 863 0518 2

1 274 1 16 1 1 1383 0618 1

1 275 1 8 1 1 911 0618 1

1 276 2 8 1 2 1092 0668 2

1 277 2 32 1 2 1346 0668 2

1 278 1 32 0 1 1113 0668 1

1 279 2 16 1 2 1509 0618 2

1 280 2 32 1 2 1664 0468 2

1 281 2 32 1 2 1069 0618 2

1 282 2 32 1 2 1619 0468 2

1 283 1 32 1 1 933 0568 1

1 284 1 16 1 1 1192 0468 1

1 285 2 8 1 2 1789 0468 2

1 286 1 8 1 1 2379 0468 1

1 287 1 32 1 1 1649 0518 1

1 288 1 32 1 1 1521 0618 1

1 289 2 16 0 2 1162 0618 2

1 290 2 8 1 2 655 0568 2

1 291 1 8 1 1 688 0668 2

1 292 2 8 1 2 705 0518 1

1 293 1 32 1 1 961 0568 1

1 294 1 16 1 1 1306 0568 1

1 295 1 16 1 1 1026 0618 1

1 296 1 8 1 1 1886 0468 1

1 297 1 32 1 1 970 0468 1

1 298 2 32 0 2 635 0568 2

1 299 1 32 0 1 983 0468 1

1 300 1 32 1 1 797 0568 1

1 301 1 32 1 1 1000 0468 1

1 302 2 16 1 2 2059 0468 2

1 303 2 16 1 2 2108 0618 2

1 304 1 32 1 1 1038 0468 1

1 305 1 16 1 1 919 0618 1

1 306 2 32 1 2 1131 0618 2

1 307 1 16 1 1 1153 0668 1

1 308 1 8 0 1 2172 0468 1

1 309 1 32 1 1 1399 0618 1

1 310 2 32 1 2 1013 0468 2

1 311 1 16 1 1 2433 0518 1

1 312 1 32 1 1 1133 0518 1

1 313 2 16 1 2 2559 0518 2

1 314 1 16 1 1 1058 0518 1

1 315 1 16 1 1 897 0668 1

1 316 1 8 1 1 590 0668 2

1 317 2 16 0 2 1030 0618 2

1 318 1 8 1 1 644 0668 1

1 319 2 16 1 2 728 0668 2

1 320 1 16 1 1 2097 0618 1

1 321 2 8 1 2 787 0568 1

1 322 1 8 0 1 2556 0468 1

1 323 1 16 1 1 847 0668 1

1 324 1 16 1 1 1125 0618 1

1 325 2 16 1 2 1302 0518 2

1 326 1 8 1 1 1736 0568 2

1 327 1 16 1 1 1287 0618 1

1 328 2 8 1 2 1519 0618 2

1 329 2 32 1 2 975 0518 2

1 330 2 16 1 2 908 0668 2

1 331 2 32 0 2 949 0518 2

1 332 1 8 1 1 1352 0568 2

1 333 1 32 1 1 1007 0518 1

1 334 2 16 1 2 1935 0568 2

1 335 2 16 1 2 1430 0668 2

1 336 2 8 1 2 1103 0618 2

1 337 1 16 1 1 1095 0568 1

1 338 1 32 1 1 1145 0518 1

1 339 1 8 1 1 1152 0518 1

1 340 2 16 1 2 1391 0468 2

1 341 2 16 1 2 1172 0668 2

1 342 1 32 1 1 999 0468 1

1 343 2 16 1 2 1074 0618 2

1 344 1 8 1 1 818 0668 2

1 345 2 8 1 2 1692 0618 2

1 346 2 8 1 2 997 0618 2

1 347 1 16 1 1 1157 0618 1

1 348 1 8 1 1 1058 0468 1

1 349 1 16 1 1 1136 0468 1

1 350 1 16 1 1 807 0568 1

1 351 1 32 1 1 806 0618 1

1 352 2 32 1 2 982 0618 2

1 353 1 8 1 1 942 0568 1

1 354 1 32 1 1 741 0468 1

1 355 2 16 0 2 879 0518 2

1 356 1 16 0 1 1447 0468 1

1 357 1 8 1 1 669 0468 1

1 358 2 32 0 2 788 0668 2

1 359 2 16 1 2 1101 0568 2

1 360 1 8 1 1 985 0668 2

1 361 2 16 1 2 1029 0568 2

1 362 1 32 1 1 1134 0568 1

1 363 1 32 1 1 1065 0668 1

1 364 1 16 1 1 1016 0618 1

1 365 1 8 1 1 817 0518 2

1 366 1 8 1 1 881 0618 1

1 367 1 32 1 1 861 0668 1

1 368 2 32 1 2 1593 0568 2

1 369 1 8 1 1 2245 0518 1

1 370 2 8 1 2 1216 0518 2

1 371 1 16 0 1 958 0568 1

1 372 2 32 0 2 891 0518 2

1 373 2 8 1 2 1981 0518 2

1 374 2 32 1 2 1576 0618 2

1 375 2 8 1 2 800 0618 2

1 376 2 16 1 2 1442 0568 2

1 377 2 8 1 2 2316 0468 2

1 378 1 32 1 1 1352 0618 1

1 379 2 32 1 2 932 0668 2

1 380 2 16 1 2 1418 0668 2

1 381 2 32 1 2 1033 0668 2

1 382 2 8 1 2 720 0618 2

1 383 2 32 1 2 1191 0518 2

1 384 1 32 1 1 875 0518 1

1 385 1 16 1 1 1107 0518 1

1 386 2 8 1 2 1615 0668 2

1 387 2 8 0 2 2134 0468 2

1 388 1 16 1 1 862 0668 1

1 389 1 8 1 1 945 0568 1

1 390 2 8 1 2 1505 0568 2

1 391 2 16 1 2 2281 0568 2

1 392 2 32 1 2 1152 0668 2

1 393 1 8 1 1 854 0668 1

1 394 2 32 0 2 2072 0468 2

1 395 2 16 1 2 2443 0468 2

1 396 2 32 1 2 1408 0468 2

1 397 1 8 1 1 980 0618 1

1 398 1 32 1 1 687 0618 1

1 399 2 16 1 2 956 0568 2

1 400 1 16 1 1 1833 0568 1

1 401 1 32 1 1 1018 0668 1

1 402 2 8 1 2 1249 0568 2

1 403 2 8 1 2 1309 0518 2

1 404 1 32 1 1 1067 0468 1

1 405 2 16 1 2 1493 0468 2

1 406 1 16 1 1 1932 0468 1

1 407 2 16 0 2 1230 0618 2

1 408 1 32 1 1 917 0468 1

1 409 2 16 0 2 1174 0668 2

1 410 2 8 1 2 909 0468 2

1 411 2 16 1 2 1759 0468 2

1 412 1 16 1 1 644 0668 1

1 413 1 16 1 1 1324 0518 1

1 414 2 32 1 2 1275 0568 2

1 415 2 8 1 2 1275 0668 2

1 416 1 8 1 1 854 0568 2

1 417 1 32 1 1 870 0618 1

1 418 1 8 1 1 1058 0518 2

1 419 2 16 1 2 1950 0468 2

1 420 1 32 1 1 773 0568 1

1 421 2 16 1 2 1681 0618 2

1 422 2 8 1 2 1488 0568 2

1 423 1 32 1 1 3137 0668 1

1 424 2 8 1 2 908 0668 2

1 425 1 16 1 1 1842 0468 1

1 426 1 8 1 1 876 0518 1

1 427 2 8 1 2 1380 0618 2

1 428 2 8 1 2 1264 0518 1

1 429 2 32 1 2 939 0668 2

1 430 2 8 1 2 812 0518 1

1 431 2 8 1 2 1012 0568 2

1 432 1 16 1 1 1224 0518 1

1 433 1 16 1 1 624 0668 1

1 434 2 16 0 2 1322 0468 2

1 435 2 32 1 2 1106 0568 2

1 436 2 16 1 2 2136 0668 2

1 437 2 32 1 2 963 0668 2

1 438 2 8 1 2 757 0668 2

1 439 2 16 1 2 1471 0518 2

1 440 1 16 1 1 1888 0568 1

1 441 2 8 1 2 819 0468 2

1 442 1 16 1 1 908 0568 1

1 443 2 16 0 2 1248 0518 2

1 444 2 32 0 2 1056 0618 2

1 445 2 32 1 2 1557 0468 2

1 446 1 8 1 1 894 0568 1

1 447 2 32 1 2 1409 0618 2

1 448 1 32 1 1 1227 0568 1

1 449 1 8 1 1 2377 0468 1

1 450 1 32 1 1 1104 0518 1

1 451 1 8 1 1 958 0518 1

1 452 2 8 1 2 883 0468 2

1 453 2 16 1 2 969 0568 2

1 454 2 16 1 2 1658 0518 2

1 455 2 8 1 2 649 0468 2

1 456 2 32 1 2 842 0668 2

1 457 1 16 0 1 989 0518 1

1 458 1 32 1 1 859 0568 1

1 459 1 16 1 1 804 0668 1

1 460 1 16 1 1 1332 0468 1

1 461 1 32 1 1 748 0668 1

1 462 1 8 1 1 980 0618 1

1 463 1 16 1 1 1392 0518 1

1 464 1 32 1 1 748 0668 1

1 465 2 32 1 2 1124 0568 2

1 466 1 16 1 1 1384 0668 1

1 467 1 32 1 1 1100 0668 1

1 468 1 16 1 1 961 0518 1

1 469 2 32 1 2 1069 0518 2

1 470 1 8 1 1 942 0568 1

1 471 2 16 1 2 2485 0468 2

1 472 2 32 1 2 828 0568 2

1 473 2 8 1 2 806 0568 2

1 474 1 8 1 1 1901 0618 1

1 475 2 32 1 2 994 0618 2

1 476 2 8 1 2 2255 0568 1

1 477 2 8 1 2 1816 0618 2

1 478 1 16 1 1 1122 0468 1

1 479 1 8 1 1 947 0618 1

1 480 2 8 1 2 3684 0518 2

1 481 1 16 1 1 1494 0468 1

1 482 2 32 1 2 1242 0518 2

1 483 1 16 1 1 1170 0568 1

1 484 1 8 1 1 911 0568 1

1 485 1 8 1 1 2516 0568 2

1 486 2 16 1 2 894 0568 2

1 487 1 32 1 1 885 0568 1

1 488 2 32 1 2 1074 0668 2

1 489 2 16 0 2 1694 0518 2

1 490 2 32 1 2 1126 0568 2

1 491 1 8 1 1 1616 0618 1

1 492 1 32 1 1 992 0468 1

1 493 1 8 1 1 908 0568 2

1 494 2 16 1 2 1021 0618 2

1 495 1 32 1 1 1266 0468 1

1 496 1 16 1 1 2378 0468 1

1 497 2 8 1 2 1974 0468 2

1 498 2 32 1 2 1529 0468 2

1 499 2 8 1 2 851 0618 2

1 500 1 8 1 1 1424 0518 1

1 501 2 32 1 2 1411 0668 2

1 502 2 16 1 2 1000 0618 2

1 503 2 32 1 2 1143 0518 2

1 504 1 16 1 1 934 0618 1

1 505 2 32 1 2 2028 0468 2

1 506 2 32 0 2 888 0518 2

1 507 1 16 0 1 1033 0618 1

1 508 1 16 1 1 926 0618 1

1 509 1 8 1 1 850 0668 1

1 510 1 16 1 1 913 0468 1

1 511 1 8 1 1 611 0668 1

1 512 2 32 1 2 1558 0668 2

1 513 2 32 1 2 796 0618 2

1 514 2 8 0 2 1369 0668 1

1 515 1 32 1 1 992 0568 1

1 516 1 16 1 1 2172 0468 1

1 517 1 16 1 1 1584 0518 1

1 518 2 16 1 2 1396 0618 2

1 519 1 32 1 1 1125 0668 1

1 520 1 32 1 1 725 0618 1

1 521 2 32 1 2 1640 0468 2

1 522 2 16 0 2 1070 0518 2

1 523 1 8 1 1 893 0518 2

1 524 2 8 1 2 791 0618 2

1 525 2 8 1 2 730 0668 2

1 526 2 32 1 2 1128 0468 2

1 527 1 8 1 1 960 0468 1

1 528 1 32 0 1 1151 0468 1

1 529 1 8 1 1 901 0668 1

1 530 2 32 1 2 1635 0618 2

1 531 1 32 1 1 1490 0618 1

1 532 2 32 1 2 1363 0668 2

1 533 1 32 1 1 970 0568 1

1 534 2 8 0 2 1571 0468 2

1 535 1 16 1 1 2279 0518 1

1 536 1 16 1 1 1004 0568 1

1 537 2 8 1 2 1153 0468 2

1 538 1 32 1 1 2826 0468 1

1 539 1 32 1 1 741 0568 1

1 540 1 8 1 1 764 0618 1

1 541 2 8 1 2 919 0668 2

1 542 2 16 1 2 2174 0518 2

1 543 2 32 1 2 1310 0518 2

1 544 1 16 1 1 917 0518 1

1 545 1 32 1 1 968 0668 1

1 546 2 32 1 2 1410 0668 2

1 547 2 16 1 2 1494 0618 2

1 548 1 8 1 1 1060 0468 1

1 549 2 8 1 2 864 0618 2

1 550 1 32 1 1 876 0568 1

1 551 1 8 1 1 1177 0618 1

1 552 2 8 1 2 1480 0568 2

1 553 1 32 1 1 1400 0618 1

1 554 2 32 1 2 905 0568 2

1 555 1 16 1 1 1509 0668 1

1 556 1 16 1 1 603 0668 1

1 557 1 16 1 1 890 0568 1

1 558 2 8 1 2 886 0668 2

1 559 1 16 0 1 1353 0468 1

1 560 1 8 0 1 1184 0468 1

1 561 1 32 1 1 1077 0618 1

1 562 1 32 1 1 681 0618 1

1 563 1 8 1 1 814 0518 2

1 564 1 8 1 1 669 0568 2

1 565 2 8 1 2 1929 0618 2

1 566 1 32 1 1 902 0668 1

1 567 1 16 1 1 899 0568 1

1 568 1 8 1 1 1418 0468 1

1 569 2 16 1 2 811 0668 2

1 570 1 8 1 1 1175 0618 1

1 571 2 16 1 2 867 0568 2

1 572 1 32 1 1 1937 0568 1

1 573 1 16 1 1 1027 0618 1

1 574 2 8 1 2 1545 0468 2

1 575 2 16 1 2 1134 0518 2

1 576 1 8 1 1 900 0618 1

1 577 2 16 0 2 3216 0468 2

1 578 1 16 0 1 895 0468 1

1 579 1 32 1 1 889 0618 1

1 580 1 32 1 1 926 0468 1

1 581 2 16 1 2 1162 0468 2

1 582 2 16 1 2 1064 0468 2

1 583 1 8 0 1 795 0568 1

1 584 2 32 1 2 1306 0668 2

1 585 1 8 1 1 913 0668 1

1 586 2 8 0 2 1783 0468 2

1 587 2 16 1 2 1723 0618 2

1 588 1 32 1 1 1314 0518 1

1 589 1 32 1 1 775 0568 1

1 590 1 32 1 1 1053 0668 1

1 591 2 8 1 2 1010 0568 1

1 592 2 32 1 2 1296 0568 2

1 593 1 8 1 1 979 0468 1

1 594 2 8 1 2 817 0618 2

1 595 2 8 1 2 945 0618 2

1 596 2 16 1 2 915 0568 2

1 597 2 16 1 2 695 0668 2

1 598 1 16 1 1 998 0618 1

1 599 1 32 1 1 980 0518 1

1 600 2 16 1 2 914 0668 2

1 601 2 8 1 2 2160 0518 2

1 602 2 16 1 2 2924 0518 2

1 603 1 16 1 1 1320 0668 1

1 604 2 16 1 2 3929 0468 2

1 605 1 8 1 1 2415 0518 1

1 606 1 32 1 1 1960 0468 1

1 607 2 16 0 2 1025 0618 2

1 608 1 8 1 1 858 0618 1

1 609 1 8 1 1 2778 0468 1

1 610 2 16 1 2 1374 0518 2

1 611 2 16 0 2 1763 0468 2

1 612 2 16 1 2 1960 0618 2

1 613 2 8 1 2 1754 0568 1

1 614 2 8 1 2 801 0618 2

1 615 1 32 1 1 1125 0618 1

1 616 1 16 1 1 708 0668 1

1 617 2 16 1 2 862 0668 2

1 618 1 32 1 1 1255 0668 1

1 619 2 8 1 2 1462 0668 1

1 620 1 32 1 1 1775 0668 1

1 621 1 8 1 1 972 0518 2

1 622 2 8 1 2 1130 0518 2

1 623 2 32 1 2 1126 0568 2

1 624 2 32 1 2 1352 0618 2

1 625 2 16 0 2 2704 0468 2

1 626 2 16 1 2 862 0568 2

1 627 2 8 1 2 1180 0518 1

1 628 2 32 1 2 1454 0468 2

1 629 1 32 1 1 953 0568 1

1 630 1 16 1 1 845 0668 1

1 631 1 16 0 1 1244 0568 1

1 632 1 16 1 1 1281 0618 1

1 633 2 32 0 2 1179 0668 2

1 634 2 32 0 2 1420 0518 2

1 635 2 8 1 2 1360 0468 2

1 636 1 32 0 1 1903 0518 1

1 637 1 16 1 1 688 0668 1

1 638 2 8 1 2 847 0668 2

1 639 1 16 1 1 1696 0518 1

1 640 1 8 1 1 839 0568 1

1 641 2 16 1 2 1327 0468 2

1 642 2 8 0 2 1002 0668 2

1 643 1 32 1 1 1441 0518 1

1 644 1 8 1 1 907 0518 2

1 645 2 32 0 2 1159 0518 2

1 646 1 8 1 1 759 0568 2

1 647 2 8 1 2 2376 0568 2

1 648 2 8 1 2 1080 0518 2

1 649 2 8 1 2 1004 0468 2

1 650 2 32 1 2 1189 0468 2

1 651 2 32 1 2 1402 0468 2

1 652 2 32 1 2 970 0468 2

1 653 2 32 1 2 952 0568 2

1 654 1 8 1 1 1239 0668 2

1 655 1 16 1 1 881 0568 1

1 656 2 16 0 2 919 0468 2

1 657 1 8 1 1 1006 0468 1

1 658 1 8 1 1 1175 0668 2

1 659 1 16 1 1 605 0668 1

1 660 2 16 1 2 711 0668 2

1 661 2 8 1 2 3826 0518 1

1 662 1 16 1 1 1445 0518 1

1 663 2 8 1 2 3036 0468 2

1 664 2 32 1 2 826 0518 2

1 665 2 32 1 2 860 0568 2

1 666 1 16 1 1 971 0568 1

1 667 1 16 1 1 1048 0618 1

1 668 1 16 1 1 969 0518 1

1 669 1 16 1 1 695 0668 1

1 670 1 32 1 1 774 0668 1

1 671 1 16 1 1 810 0518 1

1 672 1 32 1 1 723 0518 1

1 673 1 16 1 1 692 0568 1

1 674 1 8 1 1 682 0618 1

1 675 2 32 1 2 1080 0618 2

1 676 2 32 1 2 1892 0618 2

1 677 2 16 1 2 1169 0668 2

1 678 2 16 0 2 2246 0518 2

1 679 2 16 1 2 698 0668 2

1 680 2 32 1 2 743 0668 2

1 681 1 8 1 1 922 0518 1

1 682 2 8 1 2 1005 0518 1

1 683 1 8 1 1 742 0468 1

1 684 2 8 1 2 1024 0568 2

1 685 2 32 0 2 1317 0518 2

1 686 1 32 1 1 2436 0618 1

1 687 1 32 1 1 874 0468 1

1 688 1 8 1 1 680 0668 2

1 689 2 8 1 2 1412 0518 2

1 690 2 32 1 2 887 0618 2

1 691 2 8 1 2 944 0568 2

1 692 1 8 0 1 686 0518 1

1 693 2 8 1 2 1178 0518 1

1 694 1 32 1 1 1043 0668 1

1 695 1 8 1 1 938 0618 1

1 696 2 8 1 2 874 0668 2

1 697 2 16 1 2 1063 0568 2

1 698 2 16 1 2 1168 0618 2

1 699 2 32 1 2 1158 0618 2

1 700 2 8 1 2 1632 0568 2

1 701 2 32 1 2 1072 0568 2

1 702 1 32 1 1 1433 0518 1

1 703 1 8 1 1 863 0568 1

1 704 2 16 0 2 934 0568 2

1 705 1 32 1 1 796 0518 1

1 706 2 32 1 2 1018 0568 2

1 707 2 16 0 2 1280 0518 2

1 708 2 8 1 2 1037 0568 2

1 709 1 16 1 1 957 0468 1

1 710 1 32 1 1 930 0518 1

1 711 2 32 1 2 1015 0618 2

1 712 1 32 1 1 804 0468 1

1 713 2 16 1 2 715 0668 2

1 714 2 16 0 2 1365 0568 2

1 715 1 8 1 1 823 0668 2

1 716 2 8 1 2 772 0618 2

1 717 1 16 1 1 934 0518 1

1 718 2 16 1 2 1154 0568 2

1 719 1 16 1 1 1122 0468 1

1 720 1 16 1 1 1599 0618 1

1 721 1 16 1 1 1032 0618 1

1 722 2 16 1 2 1447 0518 2

1 723 1 8 1 1 843 0618 1

1 724 2 32 0 2 1474 0468 2

1 725 1 32 1 1 755 0468 1

1 726 1 32 1 1 800 0618 1

1 727 2 16 1 2 1581 0468 2

1 728 1 32 1 1 892 0618 1

1 729 1 32 1 1 1191 0518 1

1 730 2 32 0 2 757 0668 2

1 731 2 16 1 2 2090 0618 2

1 732 1 16 0 1 1217 0468 1

1 733 1 8 1 1 1192 0518 1

1 734 2 16 1 2 856 0668 2

1 735 2 8 0 2 868 0668 1

1 736 1 8 1 1 813 0618 1

1 737 1 8 1 1 740 0468 1

1 738 2 16 1 2 1299 0668 2

1 739 2 8 1 2 1036 0568 1

1 740 1 16 1 1 905 0568 1

1 741 2 32 1 2 1750 0668 2

1 742 2 16 1 2 2319 0568 2

1 743 1 8 1 1 779 0618 1

1 744 1 32 1 1 956 0668 1

1 745 2 32 1 2 1083 0618 2

1 746 2 32 1 2 1075 0568 2

1 747 1 16 1 1 1428 0518 1

1 748 2 32 1 2 1119 0518 2

1 749 1 16 1 1 2245 0468 1

1 750 1 8 0 1 1575 0468 1

1 751 1 8 1 1 1083 0518 1

1 752 2 16 1 2 1352 0518 2

1 753 2 8 1 2 1974 0468 2

1 754 1 16 0 1 1989 0468 1

1 755 2 8 1 2 1300 0468 2

1 756 1 16 1 1 2893 0468 1

1 757 1 16 0 1 1347 0618 1

1 758 1 8 1 1 1058 0668 1

1 759 2 16 1 2 1032 0518 2

1 760 1 32 1 1 1351 0468 1

1 761 2 8 1 2 1666 0468 2

1 762 1 8 1 1 832 0668 1

1 763 2 32 1 2 955 0518 2

1 764 2 8 1 2 1196 0668 2

1 765 1 32 1 1 791 0568 1

1 766 1 8 1 1 937 0618 1

1 767 2 16 0 2 796 0618 2

1 768 1 16 1 1 986 0568 1

1 769 1 8 1 1 900 0568 2

1 770 1 32 1 1 831 0518 1

1 771 1 16 1 1 756 0568 1

1 772 2 16 1 2 1843 0518 2

1 773 2 8 1 2 1241 0518 2

1 774 1 8 1 1 801 0568 1

1 775 2 8 1 2 1360 0568 1

1 776 2 8 1 2 749 0568 2

1 777 2 32 1 2 913 0668 2

1 778 1 32 1 1 1502 0518 1

1 779 2 32 1 2 1095 0468 2

1 780 1 16 1 1 1889 0668 1

1 781 1 16 0 1 845 0618 1

1 782 2 8 1 2 942 0618 2

1 783 1 8 1 1 918 0518 2

1 784 1 32 1 1 1160 0468 1

1 785 2 8 1 2 831 0568 2

1 786 2 16 1 2 1896 0618 2

1 787 1 32 1 1 1028 0568 1

1 788 1 8 1 1 986 0618 1

1 789 2 32 1 2 1059 0618 2

1 790 2 16 1 2 1051 0568 2

1 791 2 32 1 2 1881 0518 2

1 792 2 8 1 2 1245 0668 2

1 793 1 32 1 1 1292 0668 1

1 794 2 16 1 2 2097 0518 2

1 795 1 8 1 1 857 0668 2

1 796 2 32 1 2 1152 0518 2

1 797 2 8 1 2 2412 0468 2

1 798 1 16 1 1 1021 0668 1

1 799 1 8 1 1 2015 0518 1

1 800 2 16 1 2 994 0568 2

1 801 2 16 1 2 2493 0518 2

1 802 2 16 1 2 1078 0618 2

1 803 2 32 1 2 1017 0568 2

1 804 1 16 1 1 1090 0618 1

1 805 2 16 1 2 2030 0668 2

1 806 2 8 1 2 691 0668 2

1 807 1 8 0 1 782 0568 1

1 808 2 32 1 2 1287 0668 2

1 809 2 16 0 2 1378 0668 2

1 810 1 32 1 1 2149 0518 1

1 811 1 32 1 1 984 0668 1

1 812 2 16 1 2 1457 0618 2

1 813 2 32 1 2 1498 0618 2

1 814 1 32 1 1 978 0618 1

1 815 1 32 1 1 750 0568 1

1 816 1 16 1 1 1354 0668 1

1 817 1 8 1 1 2144 0468 1

1 818 1 32 1 1 987 0618 1

1 819 2 32 1 2 1117 0568 2

1 820 2 16 1 2 1924 0468 2

1 821 2 32 1 2 1202 0668 2

1 822 1 8 1 1 1593 0468 1

1 823 1 8 1 1 1034 0668 1

1 824 1 8 1 1 842 0568 1

1 825 2 16 0 2 1412 0468 2

1 826 1 8 1 1 1341 0668 2

1 827 1 32 1 1 866 0668 1

1 828 1 32 1 1 920 0668 1

1 829 2 16 1 2 827 0568 2

1 830 1 8 0 1 779 0568 2

1 831 1 16 1 1 1309 0568 1

1 832 1 16 1 1 772 0668 1

1 833 1 16 1 1 1322 0568 1

1 834 2 32 1 2 896 0668 2

1 835 1 8 1 1 901 0518 2

1 836 1 32 1 1 906 0568 1

1 837 1 8 1 1 956 0618 1

1 838 2 32 1 2 2369 0568 2

1 839 2 32 1 2 924 0568 2

1 840 1 32 1 1 1421 0468 1

1 841 2 32 1 2 941 0618 2

1 842 1 8 0 1 860 0568 1

1 843 2 16 0 2 837 0468 2

1 844 1 32 0 1 336 0468 1

1 845 1 16 0 1 363 0468 1

1 846 2 16 0 2 66 0668 2

1 847 2 16 1 2 333 0668 2

1 848 1 16 1 1 1929 0518 1

1 849 2 32 1 2 1386 0468 2

1 850 2 16 0 2 1220 0568 2

1 851 1 16 0 1 273 0568 1

1 852 2 8 1 2 53 0618 2

1 853 2 8 0 2 105 0668 2

1 854 2 8 1 2 1426 0568 2

1 855 1 32 1 1 838 0518 1

1 856 1 16 1 1 697 0668 1

1 857 2 8 1 2 920 0468 2

1 858 1 32 1 1 771 0618 1

1 859 2 16 1 2 714 0618 2

1 860 1 8 1 1 1415 0468 1

1 861 1 32 1 1 690 0568 1

1 862 1 8 1 1 659 0568 2

1 863 1 8 1 1 598 0668 2

1 864 2 8 1 2 1265 0468 2

1 865 2 32 1 2 1629 0668 2

1 866 2 32 1 2 712 0568 2

1 867 1 32 1 1 938 0468 1

1 868 2 32 1 2 984 0568 2

1 869 1 16 0 1 916 0618 1

1 870 2 8 1 2 742 0618 2

1 871 2 16 0 2 882 0468 2

1 872 1 32 1 1 1082 0668 1

1 873 2 32 1 2 1150 0518 2

1 874 1 32 1 1 874 0468 1

1 875 1 16 1 1 1004 0518 1

1 876 2 32 0 2 720 0518 2

1 877 2 16 1 2 880 0668 2

1 878 2 16 1 2 930 0668 2

1 879 2 8 1 2 761 0618 2

1 880 1 16 1 1 932 0618 1

1 881 2 8 1 2 1122 0568 2

1 882 1 8 1 1 721 0668 1

1 883 1 8 1 1 744 0468 1

1 884 1 32 1 1 948 0668 1

1 885 1 16 1 1 898 0668 1

1 886 2 32 0 2 892 0468 2

1 887 1 32 1 1 730 0518 1

1 888 2 16 1 2 950 0568 2

1 889 1 8 1 1 2056 0468 1

1 890 2 8 1 2 1196 0518 1

1 891 1 8 1 1 744 0668 2

1 892 2 8 1 2 1760 0518 1

1 893 2 8 1 2 1341 0468 2

1 894 2 8 1 2 906 0518 2

1 895 1 32 1 1 833 0518 1

1 896 2 8 1 2 1527 0568 2

1 897 2 16 1 2 893 0618 2

1 898 2 32 1 2 1534 0468 2

1 899 2 32 1 2 1672 0468 2

1 900 2 32 1 2 1175 0618 2

1 901 1 16 1 1 1010 0518 1

1 902 2 8 1 2 959 0468 2

1 903 2 16 1 2 1582 0568 2

1 904 1 16 1 1 906 0518 1

1 905 2 16 1 2 1925 0468 2

1 906 1 16 1 1 1187 0618 1

1 907 2 32 1 2 1178 0668 2

1 908 2 16 1 2 1271 0618 2

1 909 2 8 1 2 813 0668 2

1 910 1 8 0 1 673 0618 1

1 911 1 32 1 1 929 0668 1

1 912 1 16 1 1 949 0568 1

1 913 1 32 1 1 729 0568 1

1 914 2 8 1 2 1370 0518 1

1 915 1 32 1 1 1216 0618 1

1 916 2 8 1 2 1131 0668 2

1 917 2 8 1 2 1482 0518 1

1 918 2 8 1 2 1324 0518 2

1 919 1 16 1 1 2007 0518 1

1 920 1 16 1 1 1152 0468 1

1 921 1 8 1 1 1182 0518 1

1 922 2 8 1 2 972 0618 2

1 923 2 16 0 2 1695 0518 2

1 924 1 16 1 1 731 0668 1

1 925 1 32 1 1 707 0568 1

1 926 1 32 1 1 1033 0468 1

1 927 1 16 1 1 1240 0618 1

1 928 2 8 1 2 841 0668 1

1 929 2 32 1 2 1296 0618 2

1 930 1 16 1 1 1129 0468 1

1 931 1 16 0 1 1033 0468 1

1 932 1 16 1 1 627 0518 1

1 933 2 16 1 2 795 0568 2

1 934 2 16 1 2 1024 0468 2

1 935 2 8 1 2 994 0618 2

1 936 2 32 1 2 1093 0568 2

1 937 1 8 1 1 836 0468 1

1 938 2 16 0 2 865 0468 2

1 939 2 32 1 2 1270 0618 2

1 940 2 32 1 2 1242 0468 2

1 941 2 32 1 2 1261 0518 2

1 942 2 32 1 2 960 0468 2

1 943 2 16 1 2 930 0518 2

1 944 1 8 1 1 802 0618 1

1 945 1 16 1 1 1283 0518 1

1 946 2 8 1 2 2356 0518 2

1 947 2 8 1 2 842 0568 2

1 948 1 8 1 1 924 0518 2

1 949 1 32 1 1 1723 0618 1

1 950 1 32 1 1 732 0618 1

1 951 2 8 1 2 1055 0618 2

1 952 2 32 1 2 1519 0518 2

1 953 1 16 1 1 764 0668 1

1 954 1 32 1 1 891 0518 1

1 955 1 8 1 1 1041 0518 2

1 956 2 8 1 2 755 0618 2

1 957 1 32 0 1 908 0568 1

1 958 1 16 1 1 1085 0568 1

1 959 2 32 1 2 1023 0618 2

1 960 1 8 1 1 1195 0568 2

1 961 2 32 1 2 1272 0668 2

1 962 2 16 0 2 2232 0568 2

1 963 1 8 1 1 990 0668 2

1 964 2 8 1 2 1066 0618 2

1 965 1 8 1 1 981 0568 2

1 966 1 32 1 1 763 0618 1

1 967 1 16 1 1 739 0668 1

1 968 2 32 0 2 630 0518 2

1 969 2 16 1 2 808 0668 2

1 970 2 8 0 2 1063 0468 2

1 971 1 16 1 1 2791 0668 1

1 972 2 32 1 2 1584 0568 2

1 973 2 8 1 2 771 0518 2

1 974 2 8 1 2 1102 0518 1

1 975 2 8 1 2 862 0568 2

1 976 1 8 1 1 1241 0468 1

1 977 1 8 1 1 675 0518 2

1 978 1 32 1 1 791 0518 1

1 979 1 32 1 1 721 0518 1

1 980 2 16 0 2 1306 0468 2

1 981 2 32 1 2 1201 0468 2

1 982 1 32 1 1 1265 0468 1

1 983 1 32 1 1 1056 0618 1

1 984 1 8 1 1 1868 0518 2

1 985 2 8 1 2 895 0518 1

1 986 2 16 1 2 1090 0468 2

1 987 2 16 0 2 1390 0468 2

1 988 2 8 0 2 644 0618 2

1 989 2 8 1 2 201 0668 1

1 990 1 8 1 1 908 0668 2

1 991 1 8 1 1 1157 0468 1

1 992 1 32 1 1 946 0518 1

1 993 1 8 1 1 842 0468 1

1 994 1 32 1 1 735 0668 1

1 995 2 16 1 2 1973 0618 2

1 996 1 32 1 1 682 0518 1

1 997 2 8 1 2 966 0568 1

1 998 1 8 1 1 943 0468 1

1 999 2 16 1 2 816 0668 2

1 1000 2 8 1 2 759 0668 2

1 1001 1 8 1 1 656 0468 1

1 1002 2 8 1 2 751 0618 2

1 1003 1 8 0 1 727 0468 1

1 1004 1 32 1 1 980 0468 1

1 1005 1 32 1 1 844 0668 1

1 1006 2 16 0 2 709 0618 2

1 1007 1 16 1 1 1009 0568 1

1 1008 2 32 1 2 897 0618 2

1 1009 2 32 1 2 2023 0618 2

1 1010 1 8 1 1 816 0568 1

1 1011 1 16 1 1 639 0568 1

1 1012 2 32 0 2 964 0518 2

1 1013 2 16 1 2 1167 0668 2

1 1014 1 16 0 1 1571 0618 1

1 1015 2 16 1 2 855 0568 2

1 1016 2 32 1 2 1356 0468 2

1 1017 1 16 1 1 1403 0618 1

1 1018 2 8 1 2 1000 0518 2

1 1019 1 16 1 1 1012 0618 1

1 1020 1 16 1 1 820 0518 1

1 1021 2 32 1 2 1077 0568 2

1 1022 1 8 1 1 937 0668 1

1 1023 2 8 1 2 826 0668 1

1 1024 1 8 1 1 807 0518 1

1 1025 2 8 1 2 2201 0468 2

1 1026 1 16 1 1 814 0518 1

1 1027 2 32 1 2 1614 0468 2

1 1028 1 16 0 1 1918 0618 1

1 1029 1 16 1 1 917 0518 1

1 1030 2 8 1 2 787 0668 2

1 1031 2 32 0 2 1017 0518 2

1 1032 1 16 1 1 721 0568 1

1 1033 1 16 1 1 664 0668 1

1 1034 2 16 1 2 1791 0518 2

1 1035 2 8 1 2 970 0668 2

1 1036 1 16 0 1 1207 0568 1

1 1037 2 16 0 2 1358 0518 2

1 1038 1 32 1 1 931 0568 1

1 1039 2 16 1 2 933 0668 2

1 1040 1 32 1 1 1007 0618 1

1 1041 2 8 1 2 966 0618 2

1 1042 1 16 0 1 729 0668 1

1 1043 2 8 0 2 1206 0518 1

1 1044 2 32 1 2 1069 0668 2

1 1045 1 8 1 1 866 0668 1

1 1046 2 16 1 2 1379 0568 2

1 1047 1 8 1 1 1305 0518 2

1 1048 1 16 1 1 672 0518 1

1 1049 2 32 1 2 1124 0618 2

1 1050 1 16 0 1 1183 0518 1

1 1051 1 16 0 1 1092 0468 1

1 1052 2 16 1 2 471 0468 2

1 1053 1 32 0 1 889 0518 1

1 1054 2 8 0 2 306 0518 1

1 1055 1 16 0 1 217 0618 1

1 1056 1 32 1 1 160 0568 1

1 1057 1 16 1 1 721 0568 1

1 1058 1 32 1 1 1145 0468 1

1 1059 2 16 1 2 1203 0468 2

1 1060 2 8 1 2 970 0618 2

1 1061 2 32 1 2 1184 0668 2

1 1062 2 16 1 2 1174 0518 2

1 1063 1 8 1 1 1158 0468 1

1 1064 1 8 1 1 853 0568 1

1 1065 2 32 0 2 1316 0468 2

1 1066 1 8 1 1 1178 0668 1

1 1067 1 32 1 1 1171 0468 1

1 1068 1 8 1 1 773 0618 1

1 1069 2 32 1 2 1188 0618 2

1 1070 2 32 1 2 1813 0468 2

1 1071 2 32 1 2 1331 0568 2

1 1072 2 16 0 2 1206 0468 2

1 1073 1 16 1 1 1988 0618 1

1 1074 1 16 1 1 883 0518 1

1 1075 2 16 1 2 821 0618 2

1 1076 1 8 1 1 944 0568 1

1 1077 2 32 1 2 1052 0568 2

1 1078 2 16 1 2 1298 0668 2

1 1079 1 16 1 1 972 0668 1

1 1080 1 32 1 1 881 0668 1

1 1081 1 8 1 1 2067 0468 1

1 1082 1 32 1 1 733 0568 1

1 1083 2 32 1 2 1096 0668 2

1 1084 1 8 1 1 996 0618 1

1 1085 1 16 1 1 903 0668 1

1 1086 1 32 1 1 793 0568 1

1 1087 2 32 1 2 1053 0518 2

1 1088 2 16 1 2 734 0618 2

1 1089 2 16 1 2 986 0518 2

1 1090 2 16 1 2 903 0468 2

1 1091 2 8 1 2 741 0618 2

1 1092 2 32 1 2 723 0568 2

1 1093 1 16 0 1 689 0468 1

1 1094 2 32 1 2 856 0468 2

1 1095 2 32 1 2 851 0618 2

1 1096 2 16 1 2 713 0668 2

1 1097 1 8 1 1 876 0568 2

1 1098 1 8 1 1 601 0668 2

1 1099 1 16 1 1 1702 0468 1

1 1100 1 16 1 1 1315 0468 1

1 1101 2 8 1 2 1006 0568 2

1 1102 2 8 1 2 1734 0468 2

1 1103 1 32 1 1 1038 0568 1

1 1104 1 32 1 1 642 0618 1

1 1105 2 32 0 2 713 0618 2

1 1106 1 16 1 1 888 0568 1

1 1107 2 32 1 2 838 0618 2

1 1108 2 16 1 2 928 0618 2

1 1109 1 16 1 1 1496 0468 1

1 1110 2 8 1 2 1728 0468 2

1 1111 2 16 1 2 956 0518 2

1 1112 1 8 0 1 725 0618 1

1 1113 1 32 1 1 961 0618 1

1 1114 1 32 1 1 1196 0568 1

1 1115 2 8 0 2 1122 0668 2

1 1116 2 8 1 2 1062 0668 2

1 1117 2 32 0 2 1979 0518 2

1 1118 2 16 1 2 1498 0468 2

1 1119 2 8 1 2 1208 0468 2

1 1120 1 8 1 1 931 0618 1

1 1121 2 32 1 2 2608 0568 2

1 1122 1 16 1 1 1488 0468 1

1 1123 1 8 1 1 764 0618 1

1 1124 1 16 1 1 618 0668 1

1 1125 2 32 0 2 662 0668 2

1 1126 1 32 1 1 1130 0668 1

1 1127 1 32 1 1 659 0668 1

1 1128 1 32 1 1 719 0668 1

1 1129 2 16 1 2 1213 0568 2

1 1130 2 8 1 2 631 0568 2

1 1131 1 8 1 1 1259 0568 2

1 1132 2 32 1 2 1118 0668 2

1 1133 1 8 1 1 1010 0668 2

1 1134 2 16 1 2 1664 0568 2

1 1135 1 32 1 1 1168 0468 1

1 1136 2 16 0 2 1403 0568 2

1 1137 2 8 1 2 999 0568 2

1 1138 2 16 0 2 948 0568 2

1 1139 1 32 1 1 801 0618 1

1 1140 1 32 1 1 917 0618 1

1 1141 1 32 1 1 866 0518 1

1 1142 1 32 1 1 963 0468 1

1 1143 1 8 1 1 908 0518 1

1 1144 1 32 1 1 797 0468 1

1 1145 2 16 1 2 889 0668 2

1 1146 2 32 1 2 772 0518 2

1 1147 2 16 1 2 2173 0518 2

1 1148 1 8 1 1 1107 0518 1

1 1149 1 8 1 1 955 0518 1

1 1150 2 32 1 2 1134 0468 2

1 1151 2 32 0 2 1225 0618 2

1 1152 2 8 1 2 3589 0518 2

1 1153 2 32 1 2 1400 0668 2

1 1154 1 8 1 1 1381 0668 1

1 1155 1 32 1 1 669 0568 1

1 1156 2 32 1 2 906 0518 2

1 1157 2 8 1 2 1883 0468 2

1 1158 2 8 1 2 635 0618 2

1 1159 2 32 1 2 863 0518 2

1 1160 1 16 0 1 936 0568 1

1 1161 2 8 1 2 2112 0518 2

1 1162 1 16 0 1 1042 0618 1

1 1163 1 32 1 1 1094 0668 1

1 1164 1 16 1 1 741 0668 1

1 1165 2 16 0 2 2762 0518 2

1 1166 1 32 1 1 1294 0468 1

1 1167 2 8 1 2 827 0568 1

1 1168 1 8 1 1 739 0568 1

1 1169 1 16 0 1 1697 0468 1

1 1170 2 16 1 2 1492 0618 2

1 1171 1 32 1 1 959 0518 1

1 1172 2 32 0 2 1899 0468 2

1 1173 1 8 1 1 1315 0618 1

1 1174 2 8 1 2 1420 0468 2

1 1175 2 16 1 2 1020 0568 2

1 1176 1 16 1 1 1075 0468 1

1 1177 2 8 1 2 996 0668 2

1 1178 1 16 1 1 2186 0518 1

1 1179 1 8 1 1 687 0618 1

1 1180 2 8 1 2 725 0618 2

1 1181 2 32 1 2 1493 0668 2

1 1182 2 8 1 2 1271 0568 2

1 1183 2 32 1 2 813 0568 2

1 1184 2 16 1 2 1720 0518 2

1 1185 2 16 1 2 865 0668 2

1 1186 2 32 1 2 1032 0568 2

1 1187 1 32 1 1 1119 0668 1

1 1188 2 16 1 2 1294 0618 2

1 1189 1 32 1 1 844 0618 1

1 1190 1 32 1 1 820 0568 1

1 1191 1 8 1 1 750 0568 2

1 1192 1 8 1 1 1075 0518 2

1 1193 1 16 1 1 933 0618 1

1 1194 2 8 1 2 1115 0468 2

1 1195 1 16 1 1 1037 0518 1

1 1196 1 8 1 1 811 0618 1

1 1197 2 16 1 2 904 0618 2

1 1198 1 32 1 1 986 0518 1

1 1199 1 16 1 1 868 0568 1

1 1200 2 8 1 2 774 0568 2

2 1 1 8 1 1 2562 468 1

2 2 2 32 1 2 1117 518 2

2 3 2 32 1 2 1150 618 2

2 4 2 8 1 2 2459 468 2

2 5 2 16 1 2 1677 518 2

2 6 1 8 1 1 1246 568 1

2 7 1 32 1 1 1254 618 1

2 8 1 16 1 1 1183 568 1

2 9 1 16 1 1 1074 568 1

2 10 1 8 1 1 1060 518 1

2 11 1 32 1 1 1106 668 1

2 12 1 8 1 1 1002 518 1

2 13 1 32 1 1 1480 668 1

2 14 2 8 1 2 1497 618 2

2 15 1 16 1 1 2325 568 1

2 16 1 8 1 1 1181 668 2

2 17 2 8 1 2 2117 618 2

2 18 2 8 1 2 774 468 2

2 19 1 8 1 1 1081 568 2

2 20 1 16 1 1 2555 468 1

2 21 1 8 1 1 2153 618 1

2 22 1 32 1 1 1987 568 1

2 23 2 16 0 2 1649 468 2

2 24 2 8 0 2 1233 468 2

2 25 1 8 1 1 1000 668 1

2 26 2 32 1 2 1565 568 2

2 27 2 16 1 2 1782 568 2

2 28 2 8 1 2 1412 568 1

2 29 2 32 1 2 1514 618 2

2 30 2 16 1 2 1584 668 2

2 31 1 8 1 1 1172 618 1

2 32 2 32 1 2 1278 668 2

2 33 2 32 1 2 1307 668 2

2 34 1 32 1 1 1282 668 1

2 35 1 32 1 1 1321 568 1

2 36 2 32 1 2 1458 468 2

2 37 1 16 1 1 2126 618 1

2 38 1 32 1 1 3176 568 1

2 39 2 16 1 2 1877 568 2

2 40 2 16 1 2 4748 468 2

2 41 1 32 1 1 1289 568 1

2 42 2 32 1 2 1089 618 2

2 43 2 32 1 2 1651 618 2

2 44 2 16 1 2 2438 618 2

2 45 1 16 1 1 2958 618 1

2 46 2 8 1 2 1470 668 2

2 47 1 8 1 1 1903 468 1

2 48 1 16 1 1 1645 568 1

2 49 2 32 1 2 1059 568 2

2 50 1 32 1 1 1403 518 1

2 51 1 16 1 1 1548 668 1

2 52 2 16 0 2 1630 518 2

2 53 1 16 0 1 1544 618 1

2 54 1 32 1 1 1659 518 1

2 55 2 32 1 2 1363 668 2

2 56 2 16 1 2 2154 618 2

2 57 2 8 1 2 1685 618 2

2 58 2 32 1 2 1268 668 2

2 59 2 16 0 2 1433 668 2

2 60 2 8 1 2 1438 518 1

2 61 1 8 1 1 1664 518 2

2 62 1 8 1 1 1621 468 1

2 63 2 8 1 2 1311 618 2

2 64 1 32 1 1 1368 668 1

2 65 2 16 0 2 2374 468 2

2 66 2 32 1 2 1354 568 2

2 67 1 16 1 1 2041 618 1

2 68 2 32 1 2 1438 518 2

2 69 1 32 1 1 2701 518 1

2 70 2 16 1 2 1896 518 2

2 71 1 8 1 1 1356 618 1

2 72 2 32 1 2 1216 668 2

2 73 1 32 1 1 1313 468 1

2 74 1 16 0 1 1618 518 1

2 75 2 8 1 2 1270 618 2

2 76 1 32 1 1 1139 618 1

2 77 2 16 0 2 2032 468 2

2 78 1 16 1 1 1596 568 1

2 79 1 32 1 1 1405 468 1

2 80 2 32 1 2 1483 468 2

2 81 2 32 1 2 2024 468 2

2 82 1 16 1 1 1129 518 1

2 83 1 32 1 1 1117 618 1

2 84 2 16 0 2 1416 518 2

2 85 1 8 1 1 1330 518 2

2 86 2 32 1 2 1557 568 2

2 87 2 8 1 2 1481 568 2

2 88 1 8 1 1 1479 668 2

2 89 2 32 1 2 1804 468 2

2 90 1 16 1 1 1185 668 1

2 91 1 8 1 1 1654 518 1

2 92 2 8 1 2 1171 668 2

2 93 1 16 1 1 1243 668 1

2 94 2 32 1 2 1527 518 2

2 95 2 8 1 2 1572 518 1

2 96 2 32 1 2 1461 518 2

2 97 2 16 0 2 1592 468 2

2 98 2 16 1 2 1545 618 2

2 99 2 8 1 2 1295 518 2

2 100 2 16 1 2 2263 468 2

2 101 2 16 1 2 1895 618 2

2 102 1 8 1 1 1189 668 1

2 103 2 8 1 2 1371 568 2

2 104 1 32 1 1 1292 468 1

2 105 2 8 1 2 1243 568 2

2 106 2 32 0 2 1383 468 2

2 107 2 8 1 2 1403 618 2

2 108 2 16 1 2 1336 668 2

2 109 2 8 1 2 1241 668 2

2 110 2 8 1 2 800 568 1

2 111 1 32 1 1 1524 568 1

2 112 1 32 1 1 2423 668 1

2 113 1 16 1 1 1502 618 1

2 114 2 16 0 2 1245 468 2

2 115 1 16 1 1 1544 468 1

2 116 2 16 0 2 1352 518 2

2 117 2 8 1 2 1206 668 1

2 118 2 16 1 2 1192 568 2

2 119 1 8 1 1 1048 568 2

2 120 1 32 1 1 1405 618 1

2 121 2 16 0 2 2108 618 2

2 122 1 16 1 1 1732 518 1

2 123 2 32 1 2 1272 518 2

2 124 2 16 0 2 1677 468 2

2 125 2 16 1 2 1194 568 2

2 126 1 32 1 1 1539 468 1

2 127 1 32 1 1 1819 668 1

2 128 2 8 1 2 1338 468 2

2 129 1 8 1 1 1377 468 1

2 130 1 16 1 1 1312 668 1

2 131 1 32 1 1 1379 618 1

2 132 2 16 1 2 1536 668 2

2 133 2 32 1 2 1355 618 2

2 134 1 16 1 1 1816 668 1

2 135 1 16 1 1 2161 468 1

2 136 2 32 1 2 1235 668 2

2 137 1 8 1 1 1393 468 1

2 138 2 16 1 2 1390 618 2

2 139 1 8 1 1 1273 468 1

2 140 2 8 1 2 1057 568 2

2 141 1 8 1 1 1312 668 2

2 142 1 32 1 1 1207 668 1

2 143 2 8 1 2 2130 668 2

2 144 2 32 1 2 1254 568 2

2 145 1 32 1 1 2113 518 1

2 146 1 16 1 1 1497 468 1

2 147 2 32 1 2 1432 468 2

2 148 2 32 1 2 1073 618 2

2 149 1 8 1 1 1219 518 2

2 150 2 32 1 2 1392 568 2

2 151 1 16 1 1 1224 668 1

2 152 2 16 1 2 1968 568 2

2 153 1 8 1 1 1374 618 1

2 154 1 32 1 1 1481 668 1

2 155 2 8 1 2 1699 668 2

2 156 2 32 1 2 1481 518 2

2 157 1 8 1 1 1377 518 2

2 158 1 16 1 1 2026 618 1

2 159 1 32 1 1 1028 568 1

2 160 1 8 1 1 1174 618 1

2 161 2 16 1 2 2241 568 2

2 162 1 8 1 1 1259 618 1

2 163 2 32 1 2 1176 668 2

2 164 1 16 1 1 1064 468 1

2 165 1 8 1 1 864 568 1

2 166 2 8 1 2 957 518 2

2 167 1 16 1 1 958 468 1

2 168 1 32 1 1 1022 518 1

2 169 1 16 1 1 1094 518 1

2 170 2 32 1 2 1104 618 2

2 171 1 8 1 1 1072 518 1

2 172 2 8 1 2 1382 518 1

2 173 1 32 1 1 1161 468 1

2 174 2 16 0 2 1046 518 2

2 175 2 8 1 2 1213 618 2

2 176 2 16 1 2 1265 568 2

2 177 1 16 1 1 1004 668 1

2 178 2 8 1 2 1214 618 2

2 179 1 16 1 1 1193 568 1

2 180 1 8 1 1 1202 468 1

2 181 2 8 1 2 989 518 1

2 182 2 32 1 2 1033 568 2

2 183 1 8 1 1 1000 568 2

2 184 1 32 1 1 1215 468 1

2 185 2 32 1 2 1207 618 2

2 186 1 8 1 1 1200 618 1

2 187 1 32 1 1 1122 618 1

2 188 2 32 1 2 1805 468 2

2 189 1 8 1 1 1323 468 1

2 190 1 32 1 1 1182 568 1

2 191 2 16 0 2 1467 568 2

2 192 2 8 1 2 1610 668 2

2 193 2 8 1 2 1152 518 2

2 194 2 8 1 2 1520 468 2

2 195 1 8 1 1 1069 568 1

2 196 2 8 1 2 1610 518 2

2 197 1 16 1 1 1163 518 1

2 198 2 32 1 2 1305 468 2

2 199 1 8 1 1 909 668 2

2 200 2 16 1 2 1208 618 2

2 201 2 16 1 2 1502 668 2

2 202 2 8 1 2 1377 568 2

2 203 1 16 1 1 1375 618 1

2 204 2 8 1 2 984 468 2

2 205 1 16 0 1 1225 468 1

2 206 2 16 1 2 1244 668 2

2 207 1 32 1 1 1257 468 1

2 208 1 32 1 1 1588 468 1

2 209 1 8 1 1 1170 618 1

2 210 1 16 1 1 956 668 1

2 211 1 8 1 1 730 668 1

2 212 2 8 1 2 1635 468 2

2 213 2 32 1 2 1700 518 2

2 214 1 16 1 1 2047 518 1

2 215 2 8 1 2 1307 568 2

2 216 2 8 1 2 1331 468 2

2 217 2 16 1 2 1068 518 2

2 218 2 16 1 2 1644 668 2

2 219 2 16 1 2 1015 618 2

2 220 2 32 1 2 1634 568 2

2 221 1 16 1 1 1176 568 1

2 222 2 32 1 2 1134 668 2

2 223 1 16 1 1 1787 468 1

2 224 2 8 1 2 1021 668 1

2 225 1 16 1 1 1524 518 1

2 226 1 8 1 1 899 668 1

2 227 1 16 1 1 1400 618 1

2 228 1 32 1 1 1295 618 1

2 229 1 16 0 1 1519 518 1

2 230 1 32 1 1 1237 518 1

2 231 2 16 1 2 1371 668 2

2 232 2 16 0 2 1847 518 2

2 233 1 8 1 1 1361 568 2

2 234 1 8 1 1 971 568 1

2 235 1 32 1 1 1142 618 1

2 236 2 32 0 2 955 518 2

2 237 1 32 1 1 1832 518 1

2 238 1 32 1 1 1352 518 1

2 239 1 32 1 1 952 568 1

2 240 1 16 1 1 2496 568 1

2 241 2 8 1 2 1676 668 2

2 242 1 8 1 1 1647 518 2

2 243 2 16 0 2 1208 518 2

2 244 1 32 1 1 1258 618 1

2 245 2 8 1 2 1337 668 2

2 246 2 8 1 2 1100 618 2

2 247 1 16 1 1 1307 568 1

2 248 2 8 1 2 1079 618 2

2 249 2 8 1 2 1199 468 2

2 250 1 16 1 1 1462 618 1

2 251 2 8 1 2 1044 468 2

2 252 2 16 0 2 1683 468 2

2 253 2 16 1 2 1562 468 2

2 254 2 32 1 2 1463 468 2

2 255 2 32 1 2 1004 468 2

2 256 1 16 1 1 1939 568 1

2 257 2 8 1 2 1268 468 2

2 258 1 16 1 1 1416 518 1

2 259 1 8 1 1 1206 518 1

2 260 2 32 0 2 1312 518 2

2 261 1 32 1 1 1389 568 1

2 262 2 32 1 2 1941 668 2

2 263 1 16 1 1 1173 668 1

2 264 2 8 1 2 1329 568 2

2 265 2 32 1 2 1000 518 2

2 266 2 32 1 2 2157 468 2

2 267 1 8 1 1 1440 518 2

2 268 2 16 1 2 1622 668 2

2 269 1 8 1 1 1155 568 1

2 270 2 32 1 2 1236 618 2

2 271 1 32 1 1 1077 668 1

2 272 1 16 1 1 1213 668 1

2 273 2 8 1 2 1287 468 2

2 274 2 32 1 2 1105 618 2

2 275 2 8 0 2 1184 468 2

2 276 1 16 1 1 1360 568 1

2 277 1 32 1 1 1750 568 1

2 278 1 32 1 1 1268 468 1

2 279 2 32 1 2 1593 668 2

2 280 2 32 1 2 1145 568 2

2 281 2 16 1 2 1694 518 2

2 282 1 8 1 1 1350 468 1

2 283 1 32 1 1 1371 668 1

2 284 2 8 1 2 1968 668 2

2 285 1 32 1 1 1597 618 1

2 286 2 8 1 2 1539 518 1

2 287 1 32 1 1 1640 618 1

2 288 2 16 0 2 1903 468 2

2 289 2 16 1 2 1595 618 2

2 290 2 16 1 2 1470 518 2

2 291 1 8 1 1 1151 618 1

2 292 1 8 1 1 1435 518 1

2 293 2 16 0 2 805 568 2

2 294 1 32 1 1 1289 468 1

2 295 2 8 1 2 1657 568 2

2 296 2 16 1 2 1187 568 2

2 297 2 16 1 2 984 668 2

2 298 2 16 1 2 1123 668 2

2 299 2 16 1 2 939 668 2

2 300 2 8 1 2 1095 618 2

2 301 1 32 1 1 1583 468 1

2 302 2 32 1 2 2196 518 2

2 303 1 8 1 1 1263 618 1

2 304 1 32 1 1 1261 668 1

2 305 2 32 1 2 1295 468 2

2 306 1 32 1 1 1176 518 1

2 307 1 32 1 1 1345 468 1

2 308 2 8 1 2 1390 518 2

2 309 1 16 1 1 2079 618 1

2 310 1 16 1 1 1543 518 1

2 311 2 8 1 2 1722 468 2

2 312 1 8 1 1 1168 668 1

2 313 1 8 1 1 1232 618 1

2 314 2 8 1 2 1036 568 2

2 315 2 8 1 2 1531 618 2

2 316 2 8 1 2 1501 568 1

2 317 1 32 1 1 2136 468 1

2 318 1 32 1 1 922 468 1

2 319 1 32 1 1 794 468 1

2 320 2 32 1 2 1157 668 2

2 321 1 32 1 1 1792 518 1

2 322 1 32 1 1 998 668 1

2 323 1 32 1 1 904 668 1

2 324 2 32 1 2 984 618 2

2 325 2 16 1 2 2353 618 2

2 326 2 32 1 2 1490 618 2

2 327 2 32 1 2 1068 668 2

2 328 2 16 1 2 1666 468 2

2 329 1 16 1 1 1387 668 1

2 330 2 8 1 2 1181 518 2

2 331 1 32 1 1 1276 668 1

2 332 2 16 1 2 1123 668 2

2 333 1 16 1 1 1316 468 1

2 334 1 32 0 1 959 618 1

2 335 1 32 1 1 1400 518 1

2 336 2 8 1 2 1324 518 1

2 337 1 8 1 1 1479 618 1

2 338 1 32 1 1 886 518 1

2 339 1 8 1 1 1390 518 1

2 340 2 32 1 2 1045 668 2

2 341 1 32 1 1 1172 668 1

2 342 2 32 1 2 1423 618 2

2 343 1 8 1 1 1069 568 1

2 344 2 16 1 2 1595 618 2

2 345 2 16 1 2 1501 668 2

2 346 1 8 1 1 972 618 1

2 347 1 16 1 1 1711 618 1

2 348 1 32 1 1 1131 518 1

2 349 1 8 1 1 1015 568 2

2 350 1 8 1 1 1175 618 1

2 351 2 32 1 2 1311 568 2

2 352 1 32 1 1 1120 568 1

2 353 1 8 1 1 1347 518 2

2 354 1 16 1 1 1333 518 1

2 355 2 8 1 2 1186 568 2

2 356 2 32 1 2 1328 668 2

2 357 1 32 1 1 1107 618 1

2 358 1 8 1 1 1202 468 1

2 359 1 16 1 1 1115 468 1

2 360 1 32 1 1 1602 668 1

2 361 1 8 1 1 1266 568 1

2 362 2 16 0 2 966 568 2

2 363 1 32 1 1 1413 618 1

2 364 1 16 1 1 1563 668 1

2 365 2 8 1 2 2050 618 2

2 366 2 8 1 2 1361 618 2

2 367 1 32 1 1 1359 568 1

2 368 2 8 1 2 1483 668 2

2 369 2 32 1 2 1604 568 2

2 370 1 8 1 1 2289 618 1

2 371 2 16 1 2 1700 618 2

2 372 1 8 0 1 2579 468 1

2 373 2 32 1 2 1322 468 2

2 374 1 16 1 1 1327 518 1

2 375 2 8 0 2 1306 468 2

2 376 1 8 1 1 1208 668 2

2 377 2 32 1 2 1599 518 2

2 378 1 32 1 1 1086 568 1

2 379 1 8 1 1 1153 668 2

2 380 2 16 1 2 1305 618 2

2 381 1 16 1 1 1427 468 1

2 382 1 16 1 1 985 518 1

2 383 1 8 0 1 1637 468 1

2 384 1 16 1 1 1293 568 1

2 385 1 8 1 1 2710 468 1

2 386 1 32 1 1 1070 568 1

2 387 1 16 1 1 1195 468 1

2 388 2 16 1 2 1645 568 2

2 389 1 8 1 1 1052 668 1

2 390 2 32 1 2 2178 618 2

2 391 2 16 1 2 1591 618 2

2 392 2 32 1 2 1650 518 2

2 393 2 16 1 2 1812 518 2

2 394 2 32 1 2 1484 518 2

2 395 2 16 0 2 1901 568 2

2 396 1 8 1 1 1973 568 2

2 397 2 32 1 2 1535 668 2

2 398 2 16 1 2 1480 668 2

2 399 2 32 1 2 899 618 2

2 400 2 16 1 2 1351 568 2

2 401 2 32 1 2 1777 468 2

2 402 1 16 1 1 1082 568 1

2 403 1 32 1 1 1542 518 1

2 404 2 8 1 2 1366 668 1

2 405 2 8 1 2 12606 668 2

2 406 2 16 0 2 1772 468 2

2 407 1 8 1 1 1404 668 2

2 408 1 32 1 1 1573 568 1

2 409 1 32 1 1 747 568 1

2 410 2 8 1 2 1161 668 2

2 411 2 32 0 2 1440 468 2

2 412 1 8 1 1 1143 568 2

2 413 1 16 1 1 1183 668 1

2 414 1 32 1 1 888 618 1

2 415 2 32 1 2 1124 468 2

2 416 2 32 1 2 1341 568 2

2 417 1 32 1 1 1780 468 1

2 418 2 16 1 2 1621 468 2

2 419 2 8 1 2 1680 518 1

2 420 1 32 1 1 1833 518 1

2 421 1 16 1 1 1680 468 1

2 422 1 32 1 1 982 618 1

2 423 1 16 1 1 1743 618 1

2 424 2 16 0 2 1113 568 2

2 425 1 16 1 1 1108 568 1

2 426 2 16 1 2 1113 668 2

2 427 1 8 1 1 1214 668 1

2 428 2 32 1 2 1240 618 2

2 429 2 16 0 2 1268 468 2

2 430 2 16 1 2 1547 568 2

2 431 1 16 1 1 1173 468 1

2 432 1 16 1 1 1151 568 1

2 433 2 8 1 2 1245 618 2

2 434 2 8 1 2 795 618 2

2 435 2 16 1 2 1162 518 2

2 436 1 16 1 1 1960 618 1

2 437 2 16 0 2 1174 468 2

2 438 2 8 1 2 1369 518 2

2 439 1 8 1 1 1032 668 2

2 440 2 32 1 2 1754 568 2

2 441 1 16 1 1 1796 468 1

2 442 2 16 0 2 1388 518 2

2 443 2 16 1 2 1418 618 2

2 444 1 32 0 1 1084 518 1

2 445 1 8 1 1 1407 468 1

2 446 1 16 1 1 931 668 1

2 447 2 8 1 2 1060 568 2

2 448 2 16 0 2 1750 518 2

2 449 1 16 1 1 1509 518 1

2 450 1 16 1 1 857 668 1

2 451 2 32 1 2 2259 568 2

2 452 1 16 1 1 1888 618 1

2 453 2 32 1 2 1283 518 2

2 454 1 8 1 1 1193 518 2

2 455 2 16 1 2 1407 618 2

2 456 1 16 1 1 1158 618 1

2 457 1 8 1 1 1562 518 1

2 458 1 16 1 1 2350 518 1

2 459 2 32 1 2 1482 568 2

2 460 2 8 1 2 1232 668 1

2 461 1 16 1 1 1576 568 1

2 462 1 16 1 1 880 518 1

2 463 1 8 1 1 1378 668 1

2 464 1 8 1 1 1218 568 1

2 465 2 16 1 2 1759 518 2

2 466 1 16 0 1 1523 618 1

2 467 2 8 1 2 2327 568 2

2 468 2 32 1 2 1065 568 2

2 469 1 8 1 1 1445 618 1

2 470 2 8 1 2 1385 518 2

2 471 2 32 1 2 1086 518 2

2 472 1 8 0 1 1933 468 1

2 473 1 16 1 1 1131 668 1

2 474 2 8 1 2 1202 568 1

2 475 2 8 0 2 2109 468 2

2 476 1 8 1 1 844 568 2

2 477 1 8 1 1 1118 468 1

2 478 2 8 1 2 1100 518 1

2 479 2 32 1 2 1128 668 2

2 480 1 16 0 1 1483 468 1

2 481 1 32 1 1 1871 618 1

2 482 1 8 1 1 2917 468 1

2 483 2 16 1 2 1434 518 2

2 484 2 32 1 2 1246 468 2

2 485 2 8 1 2 1032 468 2

2 486 2 32 1 2 931 668 2

2 487 1 16 0 1 1398 568 1

2 488 1 32 0 1 1472 518 1

2 489 2 16 1 2 1311 668 2

2 490 1 32 1 1 3228 468 1

2 491 1 8 1 1 1597 518 2

2 492 1 32 1 1 1153 568 1

2 493 1 32 1 1 783 568 1

2 494 1 8 1 1 853 668 2

2 495 1 32 1 1 1607 568 1

2 496 1 32 1 1 1928 668 1

2 497 1 32 1 1 960 518 1

2 498 1 32 1 1 961 518 1

2 499 2 8 1 2 1526 668 2

2 500 1 32 1 1 1791 668 1

2 501 1 32 0 1 4501 468 1

2 502 1 8 1 1 920 568 2

2 503 2 8 1 2 885 668 2

2 504 2 32 1 2 1198 618 2

2 505 2 8 0 2 1148 668 2

2 506 2 8 1 2 1423 468 2

2 507 2 16 0 2 1861 518 2

2 508 2 8 0 2 1295 618 2

2 509 2 8 1 2 987 518 2

2 510 2 16 1 2 1122 668 2

2 511 2 32 1 2 973 618 2

2 512 2 8 1 2 1519 568 2

2 513 2 32 1 2 1338 518 2

2 514 2 16 1 2 4355 668 2

2 515 2 32 1 2 969 618 2

2 516 2 16 1 2 999 668 2

2 517 1 16 1 1 6000 668 1

2 518 1 8 1 1 1517 468 1

2 519 2 32 0 2 3714 518 2

2 520 1 16 1 1 1773 568 1

2 521 1 32 1 1 1969 468 1

2 522 1 32 1 1 1581 668 1

2 523 2 8 1 2 1280 468 2

2 524 2 32 0 2 1185 568 2

2 525 1 16 1 1 1025 668 1

2 526 1 8 1 1 2421 618 1

2 527 1 16 1 1 782 668 1

2 528 1 32 1 1 933 518 1

2 529 2 32 1 2 1128 668 2

2 530 2 8 1 2 1368 618 2

2 531 2 16 0 2 1220 568 2

2 532 1 16 1 1 2533 518 1

2 533 1 8 1 1 1847 468 1

2 534 2 16 1 2 1509 568 2

2 535 2 8 1 2 1256 668 1

2 536 2 8 1 2 784 568 2

2 537 2 16 0 2 1321 468 2

2 538 1 16 1 1 2070 468 1

2 539 2 8 1 2 1147 568 2

2 540 2 32 1 2 1605 468 2

2 541 1 16 1 1 1066 468 1

2 542 1 32 1 1 1508 568 1

2 543 1 16 1 1 3173 568 1

2 544 2 16 1 2 1645 618 2

2 545 2 8 1 2 1295 518 2

2 546 1 8 0 1 882 518 1

2 547 1 8 1 1 1725 568 2

2 548 1 32 1 1 4126 618 1

2 549 1 8 1 1 932 568 1

2 550 2 16 0 2 672 568 2

2 551 2 32 1 2 1254 468 2

2 552 2 16 1 2 1071 518 2

2 553 1 8 1 1 4824 668 1

2 554 2 16 0 2 1176 668 2

2 555 2 8 1 2 1267 618 2

2 556 1 8 0 1 969 518 1

2 557 2 32 1 2 3319 568 2

2 558 2 8 1 2 1257 568 1

2 559 2 32 1 2 1906 668 2

2 560 2 32 1 2 1586 668 2

2 561 2 8 1 2 1236 468 2

2 562 1 16 1 1 1082 668 1

2 563 2 8 1 2 1473 518 1

2 564 2 16 1 2 1344 568 2

2 565 1 32 0 1 1466 468 1

2 566 1 16 1 1 2461 518 1

2 567 2 32 0 2 2474 518 2

2 568 2 32 1 2 1936 568 2

2 569 2 8 1 2 1940 518 1

2 570 2 8 1 2 1032 618 2

2 571 1 16 1 1 2089 468 1

2 572 1 16 1 1 1526 618 1

2 573 2 16 0 2 1173 668 2

2 574 1 16 1 1 1766 468 1

2 575 1 8 1 1 1642 468 1

2 576 2 8 1 2 1226 568 1

2 577 1 8 1 1 2035 668 1

2 578 2 8 1 2 1165 518 2

2 579 2 32 1 2 1771 468 2

2 580 1 32 1 1 1339 518 1

2 581 1 16 1 1 1628 468 1

2 582 2 32 1 2 867 618 2

2 583 2 16 1 2 1672 568 2

2 584 2 32 1 2 2139 568 2

2 585 2 16 0 2 1272 468 2

2 586 2 32 1 2 1339 618 2

2 587 1 8 1 1 897 518 1

2 588 1 8 0 1 1557 468 1

2 589 2 8 1 2 1494 468 2

2 590 1 16 1 1 2169 668 1

2 591 1 8 1 1 793 568 1

2 592 2 16 0 2 2405 568 2

2 593 2 32 1 2 2198 518 2

2 594 2 16 1 2 2283 618 2

2 595 1 32 1 1 1502 668 1

2 596 2 32 0 2 2834 618 2

2 597 2 8 0 2 1541 568 2

2 598 2 32 1 2 1730 468 2

2 599 2 16 0 2 1288 518 2

2 600 1 32 1 1 1951 618 1

2 601 1 32 1 1 4673 618 1

2 602 2 8 1 2 1533 568 2

2 603 1 16 1 1 1550 568 1

2 604 2 32 1 2 1463 468 2

2 605 2 16 1 2 1841 568 2

2 606 1 16 1 1 1858 518 1

2 607 2 32 1 2 2213 568 2

2 608 2 32 1 2 807 668 2

2 609 1 8 1 1 1328 518 1

2 610 2 8 1 2 1484 668 2

2 611 2 16 0 2 1404 568 2

2 612 2 16 1 2 1147 468 2

2 613 2 16 1 2 1716 468 2

2 614 1 16 1 1 2525 618 1

2 615 2 8 1 2 1230 668 2

2 616 1 32 1 1 2563 518 1

2 617 2 32 1 2 1390 568 2

2 618 1 8 1 1 1281 668 1

2 619 2 8 1 2 2019 468 2

2 620 1 8 1 1 1279 518 2

2 621 2 32 1 2 3064 618 2

2 622 1 8 1 1 992 568 2

2 623 2 16 1 2 2479 518 2

2 624 1 16 1 1 1507 468 1

2 625 2 8 1 2 7570 668 2

2 626 2 16 1 2 913 468 2

2 627 1 8 1 1 1465 468 1

2 628 2 16 1 2 1504 618 2

2 629 1 16 1 1 5264 568 1

2 630 1 8 1 1 1438 468 1

2 631 1 8 1 1 2596 668 2

2 632 1 32 1 1 2680 568 1

2 633 1 32 1 1 2024 668 1

2 634 1 8 1 1 1348 618 1

2 635 1 8 1 1 953 468 1

2 636 1 16 1 1 8728 568 1

2 637 2 32 1 2 3058 518 2

2 638 1 8 1 1 4293 518 2

2 639 1 16 1 1 880 518 1

2 640 1 16 1 1 1091 668 1

2 641 2 8 0 2 1386 518 1

2 642 2 32 1 2 1144 668 2

2 643 1 32 1 1 3714 518 1

2 644 1 32 1 1 1063 618 1

2 645 1 8 1 1 1923 568 1

2 646 2 16 0 2 2090 668 2

2 647 2 8 0 2 2516 468 2

2 648 1 32 1 1 1465 618 1

2 649 2 16 1 2 8732 618 2

2 650 2 16 1 2 1327 618 2

2 651 2 16 1 2 2074 518 2

2 652 2 32 1 2 2362 468 2

2 653 1 16 1 1 1157 668 1

2 654 1 8 1 1 3233 668 2

2 655 1 8 1 1 1793 518 2

2 656 1 16 1 1 1732 618 1

2 657 2 16 1 2 1223 668 2

2 658 2 32 0 2 957 518 2

2 659 2 8 1 2 1902 618 2

2 660 2 16 1 2 6685 618 2

2 661 2 8 1 2 1932 618 2

2 662 1 8 1 1 1215 618 1

2 663 1 8 1 1 1093 618 1

2 664 1 16 1 1 1264 518 1

2 665 1 8 1 1 708 568 1

2 666 1 8 1 1 940 568 2

2 667 2 16 0 2 1238 518 2

2 668 1 16 1 1 2226 618 1

2 669 1 32 1 1 3227 468 1

2 670 1 16 1 1 1228 468 1

2 671 2 16 0 2 3118 468 2

2 672 1 16 1 1 1124 518 1

2 673 1 32 1 1 1693 668 1

2 674 2 16 0 2 1887 518 2

2 675 1 16 1 1 2735 618 1

2 676 1 32 1 1 1451 668 1

2 677 1 8 1 1 8537 618 1

2 678 1 32 1 1 940 618 1

2 679 1 32 1 1 1347 518 1

2 680 1 16 0 1 4958 568 1

2 681 2 16 1 2 1807 618 2

2 682 2 8 1 2 1317 518 2

2 683 2 16 1 2 1004 468 2

2 684 1 16 1 1 1497 618 1

2 685 2 32 1 2 1331 668 2

2 686 2 32 1 2 765 568 2

2 687 1 32 0 1 1931 668 1

2 688 2 8 0 2 1784 618 2

2 689 1 16 1 1 1621 568 1

2 690 1 8 1 1 2694 618 1

2 691 2 8 0 2 1010 518 1

2 692 2 32 1 2 2029 618 2

2 693 2 8 1 2 1789 668 1

2 694 2 32 1 2 1792 518 2

2 695 1 32 0 1 1393 568 1

2 696 2 16 0 2 2071 618 2

2 697 1 8 1 1 1195 618 1

2 698 2 32 0 2 3327 468 2

2 699 2 32 1 2 1272 518 2

2 700 1 32 1 1 3077 468 1

2 701 1 16 1 1 1347 618 1

2 702 1 16 1 1 1823 668 1

2 703 2 32 1 2 2125 668 2

2 704 2 16 0 2 1865 468 2

2 705 1 32 1 1 1149 468 1

2 706 1 32 1 1 1202 568 1

2 707 1 16 1 1 3303 518 1

2 708 1 32 1 1 1711 568 1

2 709 2 32 1 2 1745 568 2

2 710 2 8 1 2 1740 468 2

2 711 2 8 1 2 5410 568 2

2 712 1 8 1 1 1081 668 2

2 713 1 16 1 1 2699 468 1

2 714 1 16 1 1 1467 518 1

2 715 1 32 1 1 7389 618 1

2 716 1 8 1 1 1396 668 1

2 717 2 8 1 2 5681 618 2

2 718 1 8 1 1 1418 618 1

2 719 1 32 0 1 1355 468 1

2 720 1 16 0 1 1908 618 1

2 721 2 8 1 2 1817 618 2

2 722 1 32 1 1 1565 618 1

2 723 1 32 1 1 1802 568 1

2 724 2 8 1 2 1640 518 2

2 725 1 16 1 1 1614 668 1

2 726 2 32 0 2 1087 568 2

2 727 1 16 1 1 1492 618 1

2 728 2 8 1 2 1237 618 2

2 729 1 32 1 1 2025 468 1

2 730 2 8 1 2 1311 668 2

2 731 1 16 0 1 3762 618 1

2 732 1 16 1 1 1770 668 1

2 733 2 32 1 2 1450 568 2

2 734 1 32 1 1 1307 668 1

2 735 1 32 1 1 1272 668 1

2 736 2 16 1 2 1670 668 2

2 737 2 16 1 2 1735 618 2

2 738 2 8 1 2 1839 468 2

2 739 1 32 1 1 1689 618 1

2 740 1 8 1 1 1672 568 1

2 741 1 8 1 1 1636 518 1

2 742 1 8 1 1 1156 568 1

2 743 1 32 1 1 1536 518 1

2 744 2 32 1 2 1500 468 2

2 745 2 32 1 2 1266 618 2

2 746 2 8 1 2 1431 518 2

2 747 2 8 1 2 1346 568 2

2 748 2 16 1 2 1318 668 2

2 749 2 32 1 2 1818 568 2

2 750 2 32 1 2 2146 468 2

2 751 2 32 1 2 1230 618 2

2 752 2 32 1 2 2477 518 2

2 753 2 32 1 2 1269 468 2

2 754 1 32 0 1 1250 668 1

2 755 2 16 1 2 3010 668 2

2 756 2 32 1 2 1564 618 2

2 757 2 16 1 2 2325 568 2

2 758 1 8 1 1 1573 618 1

2 759 2 16 0 2 1459 568 2

2 760 2 8 1 2 1864 468 2

2 761 1 8 1 1 1382 668 1

2 762 2 32 1 2 1249 668 2

2 763 1 16 1 1 1778 518 1

2 764 2 8 1 2 1294 468 2

2 765 1 8 1 1 1225 518 1

2 766 2 8 1 2 1039 668 1

2 767 2 16 1 2 1574 668 2

2 768 1 32 1 1 1528 668 1

2 769 2 16 0 2 1884 518 2

2 770 1 32 1 1 1039 668 1

2 771 1 32 1 1 1063 518 1

2 772 2 16 0 2 1307 518 2

2 773 1 32 1 1 1034 518 1

2 774 1 8 1 1 2048 468 1

2 775 1 8 1 1 923 568 2

2 776 1 32 1 1 1032 618 1

2 777 1 32 1 1 1130 568 1

2 778 1 8 1 1 1054 568 2

2 779 1 8 0 1 1911 468 1

2 780 1 32 1 1 1462 618 1

2 781 1 32 1 1 1343 468 1

2 782 1 16 1 1 1400 518 1

2 783 2 8 1 2 1632 568 1

2 784 1 8 1 1 1031 668 2

2 785 1 16 1 1 1322 568 1

2 786 1 32 1 1 981 518 1

2 787 1 8 1 1 973 518 2

2 788 2 32 1 2 1434 668 2

2 789 2 16 1 2 2286 568 2

2 790 2 16 1 2 1641 468 2

2 791 1 32 1 1 1605 518 1

2 792 2 8 1 2 1458 568 1

2 793 2 16 1 2 1659 668 2

2 794 2 16 1 2 913 518 2

2 795 1 32 1 1 1201 518 1

2 796 1 16 1 1 1582 518 1

2 797 1 16 1 1 725 668 1

2 798 2 16 1 2 1196 568 2

2 799 1 8 0 1 1426 618 1

2 800 2 32 1 2 1895 468 2

2 801 2 8 1 2 1170 468 2

2 802 2 8 1 2 1232 668 2

2 803 2 8 1 2 2019 468 2

2 804 1 32 1 1 1497 468 1

2 805 2 32 1 2 1342 518 2

2 806 1 32 1 1 1245 618 1

2 807 2 32 1 2 1622 518 2

2 808 1 8 1 1 1384 618 1

2 809 2 32 1 2 1664 518 2

2 810 1 16 1 1 1555 568 1

2 811 1 8 1 1 875 668 2

2 812 1 16 1 1 956 518 1

2 813 1 32 1 1 2218 618 1

2 814 1 8 1 1 939 568 2

2 815 1 16 1 1 965 568 1

2 816 1 8 1 1 801 668 2

2 817 2 16 0 2 2063 618 2

2 818 2 8 1 2 1278 668 2

2 819 2 16 0 2 1870 468 2

2 820 2 32 1 2 1597 468 2

2 821 1 16 1 1 1088 668 1

2 822 2 16 1 2 1791 568 2

2 823 2 32 1 2 1626 618 2

2 824 1 16 1 1 1044 468 1

2 825 1 8 1 1 983 518 1

2 826 2 8 1 2 1130 668 2

2 827 1 16 1 1 1134 518 1

2 828 1 16 1 1 1866 618 1

2 829 1 8 1 1 1115 518 2

2 830 2 16 1 2 1770 468 2

2 831 2 16 0 2 1615 468 2

2 832 2 32 1 2 2170 468 2

2 833 2 32 1 2 1375 668 2

2 834 1 8 1 1 2483 568 1

2 835 2 8 1 2 1155 518 1

2 836 2 8 1 2 1577 468 2

2 837 2 32 1 2 1290 568 2

2 838 2 8 1 2 1211 568 2

2 839 1 32 1 1 1263 668 1

2 840 2 8 1 2 1701 618 2

2 841 1 8 1 1 1586 618 1

2 842 1 32 1 1 956 568 1

2 843 1 8 1 1 736 518 2

2 844 2 8 1 2 1025 668 2

2 845 1 8 1 1 1286 568 1

2 846 1 16 1 1 1184 618 1

2 847 2 16 1 2 1543 568 2

2 848 1 32 1 1 1668 468 1

2 849 1 8 1 1 1036 618 1

2 850 2 32 1 2 1210 668 2

2 851 2 8 1 2 1108 518 2

2 852 1 16 1 1 1266 468 1

2 853 2 16 0 2 1362 468 2

2 854 2 16 1 2 2163 568 2

2 855 2 32 0 2 1205 568 2

2 856 1 16 1 1 1334 618 1

2 857 2 16 0 2 1237 518 2

2 858 2 8 1 2 1390 518 1

2 859 2 16 1 2 1617 618 2

2 860 1 32 1 1 1914 518 1

2 861 2 32 1 2 1102 518 2

2 862 1 8 0 1 1985 468 1

2 863 2 32 1 2 1427 618 2

2 864 2 32 1 2 1374 518 2

2 865 1 32 1 1 1165 468 1

2 866 2 16 1 2 2153 468 2

2 867 1 16 1 1 1294 668 1

2 868 1 16 1 1 992 568 1

2 869 1 32 1 1 1579 468 1

2 870 1 8 1 1 1511 618 1

2 871 1 32 1 1 1162 618 1

2 872 1 16 1 1 1131 568 1

2 873 2 32 1 2 1269 568 2

2 874 1 16 1 1 1106 668 1

2 875 1 8 1 1 957 668 1

2 876 2 8 1 2 1614 568 2

2 877 2 8 1 2 1714 618 2

2 878 1 8 0 1 1837 468 1

2 879 2 32 1 2 1270 668 2

2 880 2 16 1 2 2802 468 2

2 881 1 8 0 1 1741 468 1

2 882 2 32 1 2 1243 668 2

2 883 1 32 1 1 1263 468 1

2 884 2 8 1 2 1185 468 2

2 885 2 16 1 2 1270 668 2

2 886 1 16 1 1 1425 468 1

2 887 2 16 1 2 1165 518 2

2 888 1 16 1 1 1612 568 1

2 889 2 8 1 2 1462 568 2

2 890 2 32 1 2 1555 618 2

2 891 1 16 1 1 1294 518 1

2 892 1 16 1 1 1221 468 1

2 893 1 16 1 1 1244 618 1

2 894 1 16 1 1 1110 518 1

2 895 2 8 1 2 1409 618 2

2 896 2 8 1 2 3038 668 1

2 897 2 32 1 2 1838 668 2

2 898 1 16 1 1 1619 568 1

2 899 2 32 1 2 1242 568 2

2 900 2 16 1 2 1635 618 2

2 901 1 16 1 1 1588 468 1

2 902 1 32 1 1 1143 568 1

2 903 1 32 1 1 1266 618 1

2 904 2 8 1 2 1285 668 2

2 905 1 8 1 1 935 668 1

2 906 2 32 1 2 1182 518 2

2 907 2 16 0 2 1237 468 2

2 908 1 8 1 1 1616 468 1

2 909 1 16 1 1 743 668 1

2 910 2 8 1 2 1133 568 2

2 911 1 32 0 1 1330 668 1

2 912 2 16 1 2 1897 618 2

2 913 2 32 1 2 1744 518 2

2 914 1 32 1 1 1247 468 1

2 915 2 32 1 2 1769 618 2

2 916 1 16 1 1 1107 468 1

2 917 2 16 1 2 1674 518 2

2 918 1 16 1 1 1401 568 1

2 919 1 16 1 1 953 518 1

2 920 1 16 1 1 958 468 1

2 921 2 16 1 2 3104 618 2

2 922 2 8 1 2 1132 518 1

2 923 2 32 1 2 1732 468 2

2 924 2 8 1 2 1500 518 1

2 925 1 8 1 1 893 668 2

2 926 2 16 0 2 1101 568 2

2 927 2 8 1 2 1159 568 2

2 928 1 8 1 1 1065 668 1

2 929 1 8 1 1 1340 468 1

2 930 2 8 1 2 1423 618 2

2 931 1 16 1 1 1423 468 1

2 932 1 16 1 1 798 668 1

2 933 1 8 1 1 1188 618 1

2 934 2 8 1 2 962 618 2

2 935 1 32 0 1 1905 668 1

2 936 2 32 1 2 1292 568 2

2 937 2 16 0 2 1502 618 2

2 938 1 32 1 1 1631 518 1

2 939 2 16 1 2 2345 668 2

2 940 1 16 1 1 1766 618 1

2 941 2 8 1 2 2286 468 2

2 942 2 16 0 2 1799 518 2

2 943 2 16 1 2 1322 618 2

2 944 2 8 1 2 1110 618 2

2 945 1 8 1 1 1157 518 1

2 946 1 16 1 1 1847 618 1

2 947 2 16 0 2 1907 518 2

2 948 1 32 1 1 1316 568 1

2 949 1 8 1 1 1606 518 2

2 950 2 32 1 2 1407 468 2

2 951 1 8 1 1 1542 568 2

2 952 2 16 1 2 1630 668 2

2 953 1 32 1 1 1090 568 1

2 954 1 32 1 1 1236 568 1

2 955 1 8 1 1 1221 468 1

2 956 2 32 1 2 1358 618 2

2 957 2 8 1 2 1234 518 2

2 958 1 8 1 1 1209 618 1

2 959 2 32 1 2 1824 668 2

2 960 1 32 1 1 923 568 1

2 961 2 16 1 2 1649 518 2

2 962 2 32 1 2 1019 618 2

2 963 1 8 1 1 1039 568 1

2 964 2 32 1 2 925 618 2

2 965 2 32 1 2 982 618 2

2 966 1 16 1 1 1227 668 1

2 967 1 16 1 1 1432 618 1

2 968 1 16 1 1 1002 568 1

2 969 1 32 1 1 1967 468 1

2 970 2 16 1 2 1708 668 2

2 971 2 32 1 2 1367 668 2

2 972 1 16 1 1 1114 568 1

2 973 1 32 1 1 1154 468 1

2 974 1 8 1 1 1142 568 2

2 975 1 8 1 1 5240 518 1

2 976 2 32 0 2 948 518 2

2 977 2 32 1 2 1484 468 2

2 978 2 16 1 2 2461 568 2

2 979 1 8 1 1 1038 518 2

2 980 1 32 1 1 1078 618 1

2 981 1 8 1 1 1066 618 1

2 982 2 16 0 2 1082 468 2

2 983 2 32 1 2 1628 518 2

2 984 1 8 1 1 1350 618 1

2 985 2 32 1 2 2476 468 2

2 986 1 16 1 1 1023 668 1

2 987 2 8 1 2 1540 468 2

2 988 2 8 1 2 1781 468 2

2 989 1 32 1 1 1603 518 1

2 990 2 16 1 2 2107 668 2

2 991 1 8 1 1 1198 518 2

2 992 1 8 1 1 935 568 1

2 993 2 32 1 2 1100 668 2

2 994 2 32 1 2 1069 668 2

2 995 1 16 1 1 958 668 1

2 996 1 32 1 1 2005 518 1

2 997 2 32 1 2 1323 618 2

2 998 2 16 1 2 1798 518 2

2 999 1 8 1 1 989 568 2

2 1000 1 32 1 1 1497 468 1

2 1001 2 8 1 2 1578 468 2

2 1002 1 16 1 1 1308 468 1

2 1003 1 8 1 1 1293 668 2

2 1004 1 8 1 1 973 618 1

2 1005 2 8 1 2 1197 518 1

2 1006 1 8 1 1 1285 618 1

2 1007 2 8 1 2 1091 618 2

2 1008 1 16 1 1 1907 468 1

2 1009 2 16 1 2 1326 668 2

2 1010 1 32 1 1 1374 668 1

2 1011 1 32 1 1 1939 618 1

2 1012 1 32 1 1 1035 568 1

2 1013 2 8 1 2 2094 618 2

2 1014 1 16 1 1 1713 568 1

2 1015 2 16 1 2 2837 568 2

2 1016 2 16 0 2 1279 468 2

2 1017 1 32 1 1 1511 518 1

2 1018 2 16 1 2 1321 668 2

2 1019 1 32 1 1 1762 568 1

2 1020 2 16 1 2 1819 618 2

2 1021 2 16 1 2 1368 568 2

2 1022 2 8 1 2 1208 618 2

2 1023 1 16 1 1 1165 668 1

2 1024 2 32 1 2 2799 568 2

2 1025 1 8 1 1 1678 668 2

2 1026 2 16 1 2 4244 668 2

2 1027 2 32 1 2 1309 468 2

2 1028 1 8 1 1 1601 568 2

2 1029 1 16 1 1 2347 618 1

2 1030 2 16 1 2 4848 618 2

2 1031 1 16 0 1 1880 468 1

2 1032 2 16 1 2 1436 518 2

2 1033 2 16 1 2 1209 468 2

2 1034 1 32 1 1 1564 518 1

2 1035 2 32 1 2 1291 618 2

2 1036 2 8 1 2 1109 468 2

2 1037 1 32 1 1 1328 618 1

2 1038 2 8 1 2 1169 668 2

2 1039 2 16 0 2 1166 468 2

2 1040 1 16 1 1 1761 618 1

2 1041 2 8 1 2 1231 668 2

2 1042 2 8 1 2 1038 468 2

2 1043 2 8 1 2 897 568 2

2 1044 1 32 1 1 1367 618 1

2 1045 2 8 1 2 1199 518 2

2 1046 2 32 1 2 1065 618 2

2 1047 1 8 1 1 1733 468 1

2 1048 1 8 0 1 2235 468 1

2 1049 2 8 1 2 726 568 2

2 1050 2 32 1 2 1093 468 2

2 1051 1 16 1 1 1297 518 1

2 1052 1 32 1 1 1199 618 1

2 1053 2 16 1 2 1849 568 2

2 1054 2 16 1 2 1323 668 2

2 1055 2 16 1 2 1993 518 2

2 1056 2 8 1 2 1420 518 2

2 1057 1 32 1 1 1886 618 1

2 1058 2 32 1 2 2067 618 2

2 1059 2 8 1 2 1096 468 2

2 1060 1 32 1 1 2177 468 1

2 1061 1 8 1 1 1639 468 1

2 1062 2 32 0 2 1163 518 2

2 1063 2 16 1 2 1572 618 2

2 1064 1 32 1 1 1971 568 1

2 1065 1 16 1 1 1417 618 1

2 1066 1 8 1 1 798 668 2

2 1067 1 16 1 1 1208 618 1

2 1068 2 8 1 2 1205 568 2

2 1069 1 32 1 1 1423 668 1

2 1070 1 8 1 1 1222 518 1

2 1071 1 32 1 1 1133 618 1

2 1072 1 8 1 1 1159 618 1

2 1073 1 16 1 1 1472 618 1

2 1074 1 8 1 1 947 568 1

2 1075 1 16 1 1 956 518 1

2 1076 2 8 1 2 3988 518 1

2 1077 1 16 1 1 1386 618 1

2 1078 2 8 1 2 1105 468 2

2 1079 2 8 1 2 1318 568 2

2 1080 1 32 1 1 4161 468 1

2 1081 1 16 0 1 1640 468 1

2 1082 2 32 1 2 1593 468 2

2 1083 2 8 1 2 1237 618 2

2 1084 1 8 1 1 1395 618 1

2 1085 2 32 1 2 1259 568 2

2 1086 2 16 1 2 1171 518 2

2 1087 2 32 1 2 1946 568 2

2 1088 1 32 1 1 1312 468 1

2 1089 1 8 1 1 2016 468 1

2 1090 1 8 1 1 707 518 2

2 1091 2 32 1 2 1798 518 2

2 1092 1 8 1 1 1156 668 1

2 1093 2 16 1 2 2062 518 2

2 1094 1 32 1 1 1226 668 1

2 1095 2 8 1 2 2255 618 2

2 1096 1 32 1 1 2296 568 1

2 1097 2 32 1 2 1227 618 2

2 1098 2 16 0 2 3195 468 2

2 1099 2 8 1 2 1546 668 1

2 1100 1 32 1 1 1303 518 1

2 1101 2 8 1 2 952 518 2

2 1102 2 16 1 2 971 568 2

2 1103 1 32 1 1 1105 668 1

2 1104 2 32 1 2 1519 518 2

2 1105 2 16 0 2 2457 618 2

2 1106 2 8 1 2 867 568 2

2 1107 1 32 1 1 1259 668 1

2 1108 2 8 1 2 1424 568 2

2 1109 2 32 0 2 3655 518 2

2 1110 1 16 1 1 1345 568 1

2 1111 2 16 1 2 3476 518 2

2 1112 1 8 1 1 1928 618 1

2 1113 2 16 0 2 2363 568 2

2 1114 2 16 1 2 2043 468 2

2 1115 2 16 1 2 6859 618 2

2 1116 1 16 1 1 1011 668 1

2 1117 2 16 0 2 3788 468 2

2 1118 2 32 1 2 1278 568 2

2 1119 1 32 1 1 977 518 1

2 1120 1 16 1 1 1975 568 1

2 1121 2 8 1 2 1407 618 2

2 1122 1 32 1 1 1484 468 1

2 1123 1 8 1 1 1353 468 1

2 1124 1 16 1 1 2720 468 1

2 1125 1 32 1 1 1172 668 1

2 1126 2 8 1 2 1318 568 1

2 1127 1 16 1 1 1205 668 1

2 1128 1 32 1 1 1253 568 1

2 1129 2 32 1 2 1298 668 2

2 1130 2 32 1 2 1644 468 2

2 1131 1 32 1 1 1164 568 1

2 1132 1 8 1 1 1561 518 1

2 1133 1 16 1 1 910 668 1

2 1134 2 32 1 2 1155 668 2

2 1135 1 16 1 1 1109 668 1

2 1136 1 8 1 1 1186 668 2

2 1137 2 32 1 2 1344 668 2

2 1138 2 16 1 2 1685 468 2

2 1139 1 8 1 1 4212 568 2

2 1140 1 16 1 1 3416 518 1

2 1141 2 16 0 2 4965 518 2

2 1142 1 16 1 1 1352 618 1

2 1143 1 32 1 1 963 668 1

2 1144 2 8 1 2 2813 668 2

2 1145 1 32 1 1 2468 568 1

2 1146 2 8 0 2 896 518 1

2 1147 1 16 0 1 2201 518 1

2 1148 2 8 1 2 984 668 2

2 1149 1 32 1 1 1583 518 1

2 1150 2 8 0 2 1822 668 1

2 1151 2 8 1 2 1466 568 1

2 1152 2 16 0 2 3413 618 2

2 1153 2 32 1 2 3449 668 2

2 1154 2 16 1 2 1194 668 2

2 1155 2 16 0 2 4639 568 2

2 1156 1 16 1 1 1190 518 1

2 1157 1 8 1 1 1558 468 1

2 1158 2 32 1 2 1509 468 2

2 1159 2 16 1 2 2596 568 2

2 1160 1 16 1 1 3576 518 1

2 1161 1 32 1 1 1237 618 1

2 1162 2 8 1 2 1353 618 2

2 1163 2 16 1 2 1122 618 2

2 1164 1 16 1 1 1506 568 1

2 1165 1 16 1 1 1392 518 1

2 1166 2 32 1 2 1918 518 2

2 1167 2 8 1 2 2603 468 2

2 1168 1 8 1 1 1024 668 1

2 1169 1 16 1 1 6373 568 1

2 1170 1 8 1 1 1036 518 1

2 1171 2 16 1 2 1183 618 2

2 1172 2 8 1 2 5756 668 2

2 1173 2 8 1 2 744 668 2

2 1174 2 8 1 2 2529 518 1

2 1175 1 16 1 1 1044 468 1

2 1176 1 32 1 1 1211 518 1

2 1177 1 32 1 1 1089 668 1

2 1178 2 32 1 2 1419 568 2

2 1179 1 8 0 1 1551 468 1

2 1180 1 16 1 1 2374 468 1

2 1181 1 16 1 1 1071 568 1

2 1182 1 32 1 1 1317 468 1

2 1183 2 32 1 2 1609 568 2

2 1184 2 8 1 2 3973 518 2

2 1185 1 8 0 1 6082 468 1

2 1186 2 32 1 2 1377 668 2

2 1187 2 8 1 2 1265 618 2

2 1188 2 32 1 2 1788 568 2

2 1189 2 32 1 2 1143 518 2

2 1190 1 8 1 1 2882 668 1

2 1191 2 32 1 2 2985 468 2

2 1192 1 32 1 1 5091 568 1

2 1193 1 8 1 1 1339 568 1

2 1194 2 16 1 2 1097 668 2

2 1195 2 32 1 2 1829 568 2

2 1196 1 8 1 1 1256 618 1

2 1197 1 16 1 1 3916 468 1

2 1198 1 8 1 1 866 668 1

2 1199 1 16 1 1 1224 518 1

2 1200 1 8 1 1 778 518 2

3 1 1 16 1 1 5957 0468 1

3 2 1 8 1 1 2619 0618 1

3 3 1 8 1 1 2630 0568 2

3 4 2 8 0 2 1392 0568 1

3 5 1 32 1 1 2722 0618 1

3 6 2 16 0 2 3574 0568 2

3 7 2 16 1 2 4045 0618 2

3 8 2 8 1 2 3086 0568 2

3 9 2 16 1 2 3180 0668 2

3 10 2 32 1 2 2143 0468 2

3 11 2 8 1 2 2118 0618 2

3 12 1 8 0 1 2590 0618 1

3 13 1 32 1 1 2639 0568 1

3 14 2 16 1 2 6359 0468 2

3 15 2 8 1 2 2891 0668 2

3 16 1 32 1 1 1333 0618 1

3 17 1 32 1 1 1642 0468 1

3 18 1 16 1 1 2033 0518 1

3 19 2 16 0 2 5900 0468 2

3 20 1 32 1 1 2000 0518 1

3 21 2 8 1 2 1931 0618 2

3 22 2 16 1 2 1316 0568 2

3 23 2 16 1 2 9603 0468 2

3 24 2 16 1 2 2678 0568 2

3 25 2 32 1 2 1512 0568 2

3 26 2 32 1 2 2200 0568 2

3 27 2 16 1 2 3950 0518 2

3 28 1 32 1 1 1557 0518 1

3 29 1 8 1 1 1471 0618 1

3 30 1 8 1 1 1080 0568 1

3 31 1 32 1 1 1572 0468 1

3 32 2 32 1 2 1575 0518 2

3 33 2 16 0 2 2925 0518 2

3 34 2 16 1 2 3121 0618 2

3 35 1 16 1 1 4548 0468 1

3 36 2 16 0 2 5431 0568 2

3 37 1 16 1 1 2318 0668 1

3 38 2 8 1 2 2639 0618 2

3 39 2 32 1 2 1051 0568 2

3 40 1 8 1 1 1908 0468 1

3 41 1 32 1 1 2446 0618 1

3 42 1 16 1 1 2039 0468 1

3 43 2 16 1 2 2113 0568 2

3 44 2 32 1 2 2707 0668 2

3 45 1 32 1 1 1556 0468 1

3 46 2 16 1 2 2330 0618 2

3 47 2 16 1 2 4563 0468 2

3 48 2 16 1 2 2544 0518 2

3 49 1 8 1 1 2540 0668 2

3 50 2 8 1 2 1852 0568 2

3 51 2 8 1 2 3199 0668 2

3 52 1 16 1 1 4571 0468 1

3 53 1 8 1 1 1781 0668 2

3 54 2 32 1 2 2396 0618 2

3 55 1 16 1 1 5054 0618 1

3 56 2 8 1 2 2469 0468 2

3 57 1 8 1 1 3548 0568 1

3 58 1 8 1 1 6631 0668 2

3 59 2 8 1 2 2404 0518 1

3 60 2 32 1 2 3158 0668 2

3 61 1 8 1 1 3443 0618 1

3 62 2 16 1 2 3792 0518 2

3 63 1 8 1 1 1974 0568 2

3 64 1 16 1 1 2097 0618 1

3 65 2 16 1 2 1643 0668 2

3 66 1 32 1 1 1357 0568 1

3 67 2 8 1 2 3752 0468 2

3 68 1 32 1 1 1845 0468 1

3 69 1 16 1 1 1487 0668 1

3 70 2 8 1 2 1348 0468 2

3 71 2 8 1 2 3915 0468 2

3 72 2 32 1 2 3790 0568 2

3 73 1 8 1 1 2097 0518 1

3 74 2 8 1 2 1342 0568 2

3 75 1 32 1 1 1853 0618 1

3 76 1 32 1 1 4171 0468 1

3 77 2 32 1 2 2004 0618 2

3 78 1 8 1 1 2607 0518 2

3 79 1 16 1 1 2199 0518 1

3 80 1 32 1 1 4218 0668 1

3 81 2 8 1 2 1401 0668 2

3 82 2 8 0 2 2578 0518 1

3 83 1 32 1 1 2072 0468 1

3 84 1 16 1 1 2610 0468 1

3 85 2 8 1 2 1941 0618 2

3 86 1 16 1 1 2907 0568 1

3 87 2 8 0 2 1054 0668 2

3 88 1 8 1 1 3437 0468 1

3 89 2 8 1 2 859 0568 2

3 90 2 16 0 2 1896 0618 2

3 91 1 16 1 1 4093 0468 1

3 92 2 8 1 2 2050 0468 2

3 93 2 32 1 2 2724 0468 2

3 94 2 8 1 2 3359 0518 2

3 95 1 32 1 1 2055 0468 1

3 96 1 8 1 1 2254 0668 1

3 97 1 16 1 1 777 0668 1

3 98 1 32 1 1 1966 0668 1

3 99 1 8 1 1 1011 0568 1

3 100 2 32 1 2 1147 0568 2

3 101 1 32 1 1 1444 0518 1

3 102 1 16 1 1 3984 0618 1

3 103 2 32 1 2 1950 0468 2

3 104 2 8 1 2 2054 0468 2

3 105 2 16 1 2 2060 0618 2

3 106 1 8 1 1 7443 0618 1

3 107 2 16 1 2 5047 0468 2

3 108 2 32 1 2 3098 0668 2

3 109 2 8 1 2 1074 0668 2

3 110 1 16 1 1 2065 0668 1

3 111 1 16 1 1 4168 0568 1

3 112 1 16 1 1 4083 0518 1

3 113 1 32 1 1 1234 0518 1

3 114 1 8 1 1 1948 0518 1

3 115 2 16 1 2 2009 0518 2

3 116 2 16 1 2 2767 0618 2

3 117 1 32 1 1 4073 0668 1

3 118 2 8 1 2 1899 0618 2

3 119 1 8 1 1 997 0668 1

3 120 1 8 1 1 4510 0568 1

3 121 2 8 1 2 1396 0668 1

3 122 2 32 1 2 1695 0468 2

3 123 2 32 1 2 1130 0618 2

3 124 1 16 1 1 2445 0568 1

3 125 2 16 1 2 2104 0468 2

3 126 2 32 1 2 1187 0618 2

3 127 2 32 1 2 1474 0668 2

3 128 2 32 1 2 1324 0468 2

3 129 1 32 1 1 1209 0618 1

3 130 2 8 1 2 1279 0568 2

3 131 1 8 1 1 1167 0568 2

3 132 2 32 1 2 935 0468 2

3 133 1 8 1 1 1171 0468 1

3 134 1 8 1 1 1185 0518 1

3 135 2 8 1 2 1153 0518 1

3 136 2 16 1 2 1598 0518 2

3 137 1 8 1 1 963 0518 2

3 138 2 16 1 2 1480 0568 2

3 139 1 8 1 1 1214 0518 2

3 140 1 8 1 1 4868 0468 1

3 141 2 32 1 2 3158 0618 2

3 142 1 8 1 1 1108 0568 2

3 143 2 32 1 2 1465 0668 2

3 144 1 16 1 1 2393 0568 1

3 145 1 32 1 1 1129 0568 1

3 146 1 16 1 1 1631 0668 1

3 147 2 16 1 2 1545 0618 2

3 148 2 16 1 2 1038 0568 2

3 149 2 32 1 2 1413 0618 2

3 150 1 32 1 1 1538 0668 1

3 151 1 32 1 1 1973 0668 1

3 152 2 32 1 2 2797 0668 2

3 153 1 8 1 1 2141 0668 2

3 154 1 32 1 1 1626 0518 1

3 155 1 32 1 1 1244 0518 1

3 156 2 8 1 2 2111 0518 1

3 157 1 8 1 1 1584 0468 1

3 158 1 32 1 1 1868 0568 1

3 159 2 32 1 2 2044 0518 2

3 160 2 32 1 2 1865 0668 2

3 161 1 16 1 1 2221 0568 1

3 162 1 16 1 1 1564 0618 1

3 163 1 32 1 1 1089 0618 1

3 164 1 32 1 1 791 0618 1

3 165 1 32 1 1 835 0568 1

3 166 1 16 0 1 1613 0518 1

3 167 1 16 1 1 1575 0568 1

3 168 2 32 0 2 1037 0518 2

3 169 2 32 1 2 946 0518 2

3 170 2 16 1 2 1672 0668 2

3 171 2 16 1 2 1185 0668 2

3 172 1 16 1 1 5072 0518 1

3 173 1 16 1 1 2337 0668 1

3 174 2 8 1 2 1334 0568 2

3 175 2 16 1 2 2185 0618 2

3 176 2 32 1 2 1006 0568 2

3 177 1 32 1 1 1629 0568 1

3 178 1 8 0 1 981 0668 1

3 179 1 16 1 1 2096 0518 1

3 180 2 8 1 2 2404 0618 2

3 181 2 8 1 2 944 0668 2

3 182 2 32 1 2 1416 0518 2

3 183 1 16 1 1 1141 0518 1

3 184 1 8 1 1 1750 0468 1

3 185 2 32 1 2 1653 0468 2

3 186 2 32 1 2 778 0568 2

3 187 1 16 0 1 1468 0468 1

3 188 2 8 1 2 3113 0468 2

3 189 2 8 1 2 762 0668 1

3 190 1 32 1 1 1949 0518 1

3 191 1 16 1 1 2176 0518 1

3 192 2 16 1 2 2029 0668 2

3 193 2 32 1 2 2211 0518 2

3 194 2 32 1 2 829 0618 2

3 195 2 32 1 2 1403 0468 2

3 196 1 32 1 1 1316 0568 1

3 197 1 16 0 1 1126 0568 1

3 198 2 16 1 2 975 0668 2

3 199 1 32 1 1 1284 0668 1

3 200 2 32 1 2 1494 0518 2

3 201 1 16 1 1 1822 0668 1

3 202 2 8 1 2 2473 0618 2

3 203 2 32 1 2 2317 0518 2

3 204 1 32 1 1 2145 0468 1

3 205 2 32 1 2 2119 0668 2

3 206 2 8 1 2 1322 0618 2

3 207 1 16 1 1 1737 0618 1

3 208 1 16 1 1 1652 0468 1

3 209 1 8 1 1 1861 0618 1

3 210 1 8 1 1 2002 0468 1

3 211 2 16 1 2 1833 0668 2

3 212 1 8 1 1 1316 0518 1

3 213 1 32 1 1 1115 0618 1

3 214 1 32 1 1 1856 0668 1

3 215 2 16 1 2 1967 0518 2

3 216 1 16 1 1 1140 0618 1

3 217 1 32 0 1 1937 0668 1

3 218 2 16 0 2 3547 0468 2

3 219 1 8 1 1 754 0668 1

3 220 2 16 1 2 942 0568 2

3 221 2 8 0 2 1172 0568 1

3 222 2 8 1 2 1320 0518 2

3 223 2 32 1 2 1033 0568 2

3 224 1 8 0 1 853 0468 1

3 225 2 8 1 2 1374 0468 2

3 226 1 16 1 1 1195 0568 1

3 227 2 32 1 2 2722 0618 2

3 228 1 32 1 1 1321 0518 1

3 229 2 16 1 2 3917 0518 2

3 230 2 16 1 2 2241 0468 2

3 231 2 8 1 2 1294 0518 2

3 232 1 8 1 1 2259 0618 1

3 233 1 16 1 1 1919 0668 1

3 234 1 8 1 1 1735 0518 2

3 235 1 16 1 1 2877 0618 1

3 236 1 32 1 1 2152 0568 1

3 237 2 16 1 2 1952 0668 2

3 238 1 8 1 1 1745 0618 1

3 239 1 16 1 1 1674 0618 1

3 240 2 8 1 2 1106 0518 2

3 241 2 8 1 2 10608 0568 2

3 242 2 32 1 2 1014 0568 2

3 243 2 16 1 2 1178 0668 2

3 244 1 8 1 1 1280 0618 1

3 245 1 16 1 1 2657 0668 1

3 246 2 16 1 2 3090 0468 2

3 247 2 16 1 2 1616 0668 2

3 248 2 8 1 2 1455 0668 2

3 249 1 8 1 1 1534 0568 2

3 250 1 32 1 1 1274 0618 1

3 251 2 32 1 2 1353 0618 2

3 252 1 8 1 1 3483 0468 1

3 253 2 32 1 2 1584 0568 2

3 254 2 32 1 2 1690 0518 2

3 255 1 16 1 1 1904 0668 1

3 256 2 8 1 2 2283 0618 2

3 257 1 8 1 1 1262 0518 1

3 258 2 32 1 2 2048 0618 2

3 259 1 32 1 1 10427 0668 1

3 260 1 32 1 1 4871 0518 1

3 261 1 8 1 1 2762 0568 1

3 262 1 32 1 1 2724 0618 1

3 263 2 16 1 2 2282 0618 2

3 264 1 8 1 1 1268 0668 1

3 265 1 16 1 1 9769 0468 1

3 266 2 8 1 2 3691 0668 1

3 267 2 16 1 2 5535 0468 2

3 268 2 16 1 2 2731 0518 2

3 269 2 8 1 2 1926 0518 1

3 270 1 16 1 1 4196 0618 1

3 271 1 8 1 1 4531 0468 1

3 272 1 16 1 1 3995 0518 1

3 273 2 8 1 2 1846 0618 2

3 274 1 16 1 1 5647 0568 1

3 275 2 32 1 2 2880 0468 2

3 276 2 16 1 2 6626 0518 2

3 277 1 8 1 1 2658 0668 1

3 278 1 16 1 1 1900 0618 1

3 279 1 16 1 1 2110 0468 1

3 280 2 8 1 2 770 0668 2

3 281 1 32 1 1 1674 0668 1

3 282 1 8 1 1 1238 0468 1

3 283 2 8 1 2 1190 0468 2

3 284 1 16 0 1 1048 0568 1

3 285 1 16 1 1 1335 0568 1

3 286 1 32 1 1 1098 0668 1

3 287 2 8 1 2 841 0568 2

3 288 1 16 1 1 1248 0468 1

3 289 1 32 1 1 770 0468 1

3 290 2 32 1 2 671 0568 2

3 291 1 16 1 1 1132 0568 1

3 292 1 32 1 1 832 0618 1

3 293 1 16 1 1 953 0518 1

3 294 1 16 1 1 684 0668 1

3 295 2 8 1 2 1018 0468 2

3 296 1 8 1 1 966 0518 1

3 297 2 16 0 2 1602 0618 2

3 298 2 32 1 2 1789 0518 2

3 299 2 8 1 2 878 0568 2

3 300 1 8 1 1 1170 0468 1

3 301 1 16 1 1 1070 0618 1

3 302 2 16 1 2 981 0518 2

3 303 1 8 1 1 757 0668 2

3 304 2 8 1 2 1452 0668 2

3 305 1 16 1 1 1105 0618 1

3 306 1 32 1 1 809 0568 1

3 307 1 8 1 1 762 0568 2

3 308 2 8 1 2 1145 0468 2

3 309 1 16 1 1 1017 0618 1

3 310 1 8 1 1 1275 0668 2

3 311 2 16 1 2 1150 0568 2

3 312 1 8 1 1 1300 0468 1

3 313 1 32 1 1 933 0518 1

3 314 1 8 1 1 778 0668 1

3 315 2 8 1 2 1043 0568 2

3 316 1 16 1 1 720 0668 1

3 317 1 16 1 1 1451 0618 1

3 318 1 8 1 1 1003 0618 1

3 319 1 8 1 1 1009 0568 1

3 320 1 16 1 1 950 0668 1

3 321 2 32 1 2 1487 0518 2

3 322 1 16 1 1 1456 0618 1

3 323 1 16 1 1 2023 0668 1

3 324 2 32 1 2 1076 0668 2

3 325 1 8 1 1 976 0518 2

3 326 1 32 1 1 1484 0668 1

3 327 2 16 1 2 1499 0568 2

3 328 2 8 1 2 1549 0618 2

3 329 1 32 1 1 1299 0668 1

3 330 1 32 1 1 1341 0518 1

3 331 1 8 1 1 2164 0618 1

3 332 2 32 1 2 1575 0668 2

3 333 2 32 1 2 1216 0468 2

3 334 2 32 1 2 1401 0518 2

3 335 2 16 0 2 2000 0468 2

3 336 2 8 1 2 1233 0668 2

3 337 1 16 1 1 1739 0518 1

3 338 2 16 1 2 2240 0468 2

3 339 1 8 1 1 2234 0468 1

3 340 2 16 1 2 2676 0618 2

3 341 2 16 1 2 3208 0568 2

3 342 2 8 1 2 2127 0468 2

3 343 2 32 1 2 1596 0468 2

3 344 2 32 1 2 887 0518 2

3 345 1 32 1 1 1298 0518 1

3 346 1 16 1 1 2727 0518 1

3 347 2 8 1 2 1049 0668 1

3 348 1 16 1 1 1637 0618 1

3 349 1 8 1 1 2650 0618 1

3 350 2 16 1 2 1168 0568 2

3 351 1 32 1 1 1501 0568 1

3 352 2 8 1 2 948 0518 1

3 353 1 16 1 1 1390 0518 1

3 354 2 8 1 2 2768 0618 2

3 355 2 16 1 2 1417 0518 2

3 356 1 32 1 1 1437 0568 1

3 357 1 8 1 1 1111 0568 1

3 358 2 8 1 2 979 0568 1

3 359 1 8 1 1 1736 0568 1

3 360 2 32 1 2 1263 0468 2

3 361 2 16 1 2 1223 0518 2

3 362 2 32 1 2 1023 0568 2

3 363 2 32 1 2 1031 0668 2

3 364 1 8 1 1 1130 0518 2

3 365 2 16 1 2 1308 0518 2

3 366 2 16 1 2 1434 0618 2

3 367 1 8 1 1 891 0668 2

3 368 2 8 1 2 1034 0518 2

3 369 2 16 1 2 1157 0618 2

3 370 1 32 1 1 1007 0618 1

3 371 2 32 1 2 924 0468 2

3 372 2 8 1 2 782 0468 2

3 373 1 8 1 1 1093 0618 1

3 374 1 32 1 1 1457 0568 1

3 375 1 32 1 1 891 0668 1

3 376 1 8 1 1 960 0568 2

3 377 1 16 1 1 2432 0518 1

3 378 2 32 1 2 1010 0618 2

3 379 1 32 1 1 1106 0618 1

3 380 2 16 0 2 1647 0468 2

3 381 2 8 1 2 2840 0618 2

3 382 1 16 1 1 1037 0568 1

3 383 1 8 1 1 1788 0568 2

3 384 1 16 1 1 977 0568 1

3 385 1 8 1 1 1893 0618 1

3 386 2 32 1 2 982 0668 2

3 387 2 16 0 2 2417 0568 2

3 388 2 8 1 2 1556 0618 2

3 389 2 32 1 2 1028 0468 2

3 390 1 32 1 1 1289 0468 1

3 391 2 8 1 2 813 0668 2

3 392 1 16 1 1 1448 0468 1

3 393 1 16 1 1 1376 0668 1

3 394 2 32 1 2 1209 0518 2

3 395 1 32 1 1 1460 0668 1

3 396 2 16 1 2 1122 0568 2

3 397 1 32 1 1 910 0618 1

3 398 2 32 1 2 899 0618 2

3 399 1 32 1 1 2898 0618 1

3 400 2 16 1 2 1638 0618 2

3 401 2 8 1 2 1935 0518 2

3 402 1 32 0 1 1128 0568 1

3 403 2 8 1 2 769 0518 1

3 404 2 32 1 2 1781 0568 2

3 405 1 8 1 1 889 0668 2

3 406 2 8 1 2 943 0518 2

3 407 1 16 1 1 2705 0518 1

3 408 2 32 1 2 1257 0668 2

3 409 2 8 1 2 1738 0618 2

3 410 2 16 1 2 1586 0668 2

3 411 2 32 1 2 1909 0668 2

3 412 1 32 1 1 2087 0468 1

3 413 1 32 1 1 2313 0468 1

3 414 2 8 1 2 1020 0568 1

3 415 1 16 1 1 1376 0518 1

3 416 2 32 1 2 1403 0668 2

3 417 1 8 1 1 1256 0518 2

3 418 2 16 1 2 1546 0618 2

3 419 2 32 1 2 1273 0618 2

3 420 2 16 1 2 1382 0568 2

3 421 2 16 1 2 959 0668 2

3 422 2 32 1 2 1361 0468 2

3 423 2 8 1 2 944 0468 2

3 424 1 32 0 1 1278 0518 1

3 425 1 8 1 1 1646 0618 1

3 426 2 8 1 2 2793 0518 1

3 427 2 32 1 2 2282 0518 2

3 428 2 16 1 2 1205 0568 2

3 429 2 8 1 2 1135 0568 2

3 430 2 16 1 2 1639 0468 2

3 431 2 16 1 2 801 0668 2

3 432 1 8 1 1 1149 0468 1

3 433 2 16 1 2 8201 0468 2

3 434 2 16 0 2 5728 0468 2

3 435 1 8 1 1 2626 0618 1

3 436 1 32 1 1 4534 0468 1

3 437 1 8 1 1 895 0518 2

3 438 1 16 1 1 2321 0668 1

3 439 2 32 1 2 1371 0618 2

3 440 2 32 1 2 1245 0518 2

3 441 2 32 1 2 920 0618 2

3 442 1 32 1 1 2934 0518 1

3 443 2 32 1 2 1120 0668 2

3 444 2 16 1 2 899 0668 2

3 445 2 32 1 2 818 0618 2

3 446 1 32 1 1 1403 0468 1

3 447 1 16 1 1 2610 0468 1

3 448 1 16 0 1 3017 0468 1

3 449 2 32 1 2 1620 0468 2

3 450 1 8 1 1 1413 0518 1

3 451 2 16 1 2 953 0668 2

3 452 1 16 1 1 1172 0568 1

3 453 2 16 1 2 1440 0618 2

3 454 1 8 1 1 1062 0668 1

3 455 1 32 1 1 2138 0568 1

3 456 2 8 1 2 1176 0668 2

3 457 2 32 0 2 1561 0568 2

3 458 2 8 1 2 1182 0518 2

3 459 2 16 0 2 1649 0518 2

3 460 2 8 1 2 1371 0568 2

3 461 1 32 1 1 1610 0568 1

3 462 2 16 1 2 1406 0518 2

3 463 1 32 1 1 1553 0468 1

3 464 1 32 1 1 1276 0618 1

3 465 1 8 1 1 1051 0518 1

3 466 2 32 1 2 1674 0568 2

3 467 1 32 1 1 1389 0568 1

3 468 1 32 1 1 1855 0518 1

3 469 1 16 1 1 2212 0468 1

3 470 2 8 1 2 942 0618 2

3 471 2 16 1 2 1498 0668 2

3 472 2 32 1 2 1444 0568 2

3 473 1 32 1 1 1469 0518 1

3 474 1 32 0 1 1321 0468 1

3 475 1 8 1 1 1312 0468 1

3 476 1 16 1 1 1235 0568 1

3 477 2 8 1 2 1471 0468 2

3 478 1 16 0 1 1059 0468 1

3 479 1 32 1 1 1039 0668 1

3 480 2 8 1 2 1133 0468 2

3 481 2 32 1 2 1110 0668 2

3 482 1 8 1 1 932 0518 1

3 483 2 16 1 2 777 0668 2

3 484 1 32 1 1 907 0518 1

3 485 1 32 1 1 954 0468 1

3 486 1 16 1 1 1179 0468 1

3 487 2 16 1 2 1268 0468 2

3 488 1 16 1 1 1211 0518 1

3 489 1 16 1 1 1602 0618 1

3 490 2 16 1 2 968 0568 2

3 491 1 8 1 1 789 0568 2

3 492 2 16 1 2 1162 0668 2

3 493 2 16 1 2 921 0668 2

3 494 1 32 1 1 1675 0568 1

3 495 2 16 1 2 932 0568 2

3 496 1 16 1 1 1444 0568 1

3 497 1 16 1 1 2321 0618 1

3 498 2 32 1 2 1803 0468 2

3 499 1 32 1 1 1309 0618 1

3 500 1 32 1 1 1958 0618 1

3 501 2 8 1 2 2656 0618 2

3 502 1 8 1 1 1162 0518 1

3 503 1 8 1 1 1375 0668 1

3 504 2 16 1 2 1463 0568 2

3 505 2 8 1 2 1711 0568 2

3 506 2 16 1 2 2053 0668 2

3 507 2 32 1 2 2449 0568 2

3 508 2 8 1 2 1772 0618 2

3 509 2 8 1 2 1022 0468 2

3 510 1 32 1 1 1497 0518 1

3 511 2 8 1 2 1861 0518 1

3 512 2 16 1 2 2037 0568 2

3 513 1 32 1 1 1622 0468 1

3 514 1 16 1 1 1777 0568 1

3 515 2 16 1 2 2613 0618 2

3 516 1 8 1 1 1342 0568 2

3 517 2 16 1 2 2173 0518 2

3 518 2 16 0 2 3342 0468 2

3 519 2 32 1 2 1438 0518 2

3 520 1 16 1 1 1330 0668 1

3 521 1 8 1 1 1373 0668 1

3 522 2 8 1 2 1698 0618 2

3 523 2 32 1 2 788 0568 2

3 524 2 8 0 2 1165 0468 2

3 525 1 32 0 1 1391 0468 1

3 526 2 32 1 2 1107 0468 2

3 527 1 8 1 1 928 0618 1

3 528 1 16 1 1 1234 0518 1

3 529 2 16 1 2 1189 0518 2

3 530 1 32 1 1 1330 0518 1

3 531 2 32 1 2 1169 0468 2

3 532 2 8 1 2 1787 0468 2

3 533 2 8 1 2 1394 0518 2

3 534 1 8 1 1 820 0668 1

3 535 1 8 1 1 1005 0518 2

3 536 2 32 1 2 1363 0618 2

3 537 1 32 1 1 1682 0668 1

3 538 2 8 1 2 1292 0668 2

3 539 1 32 1 1 1159 0568 1

3 540 1 8 1 1 1247 0468 1

3 541 1 16 1 1 1243 0568 1

3 542 2 32 1 2 1808 0668 2

3 543 1 8 1 1 1084 0518 1

3 544 1 32 1 1 2168 0568 1

3 545 2 16 1 2 1298 0618 2

3 546 2 32 1 2 1076 0468 2

3 547 2 16 1 2 6790 0468 2

3 548 1 32 1 1 1994 0568 1

3 549 2 32 1 2 1914 0518 2

3 550 2 8 1 2 1363 0668 2

3 551 1 16 1 1 1415 0668 1

3 552 2 32 1 2 774 0618 2

3 553 1 16 1 1 2388 0468 1

3 554 1 8 1 1 1101 0568 2

3 555 1 8 1 1 827 0568 1

3 556 1 16 1 1 2385 0468 1

3 557 2 8 1 2 1482 0468 2

3 558 2 8 1 2 1020 0518 1

3 559 1 8 1 1 875 0568 1

3 560 1 32 1 1 1429 0618 1

3 561 1 8 1 1 1985 0668 2

3 562 1 32 1 1 1175 0668 1

3 563 2 32 1 2 1260 0568 2

3 564 2 8 1 2 1043 0568 2

3 565 1 32 1 1 3096 0668 1

3 566 2 8 1 2 1615 0618 2

3 567 2 32 1 2 1052 0568 2

3 568 1 16 1 1 1238 0518 1

3 569 1 16 1 1 1722 0468 1

3 570 2 16 1 2 1612 0518 2

3 571 1 8 1 1 1692 0568 1

3 572 1 32 1 1 1483 0618 1

3 573 2 32 1 2 1244 0618 2

3 574 1 8 1 1 1122 0618 1

3 575 1 8 1 1 795 0668 2

3 576 1 16 1 1 1297 0518 1

3 577 2 8 1 2 943 0518 1

3 578 2 16 1 2 2036 0518 2

3 579 1 8 1 1 1940 0518 2

3 580 2 16 1 2 2869 0468 2

3 581 2 8 1 2 952 0618 2

3 582 2 16 1 2 1229 0618 2

3 583 2 32 1 2 1147 0468 2

3 584 1 8 1 1 882 0518 2

3 585 2 8 1 2 974 0668 2

3 586 2 32 1 2 1116 0668 2

3 587 2 32 1 2 1736 0618 2

3 588 1 16 1 1 2066 0618 1

3 589 1 16 1 1 1825 0568 1

3 590 2 16 1 2 934 0668 2

3 591 2 32 1 2 1453 0468 2

3 592 1 16 1 1 1571 0668 1

3 593 2 16 1 2 1419 0668 2

3 594 2 16 1 2 2086 0618 2

3 595 1 8 1 1 1367 0618 1

3 596 1 32 1 1 3222 0618 1

3 597 1 32 1 1 2793 0468 1

3 598 2 8 1 2 1098 0668 1

3 599 1 32 1 1 1261 0468 1

3 600 1 16 1 1 1502 0668 1

3 601 1 32 1 1 1893 0468 1

3 602 1 32 1 1 765 0568 1

3 603 2 8 1 2 1566 0468 2

3 604 2 16 1 2 1042 0568 2

3 605 2 8 1 2 829 0568 2

3 606 1 8 1 1 1030 0468 1

3 607 2 32 1 2 1368 0518 2

3 608 2 16 1 2 938 0518 2

3 609 2 16 1 2 1144 0518 2

3 610 2 16 1 2 1546 0518 2

3 611 1 16 1 1 2245 0518 1

3 612 2 32 1 2 1811 0518 2

3 613 2 8 1 2 1395 0618 2

3 614 1 16 1 1 2068 0518 1

3 615 1 32 1 1 1817 0468 1

3 616 1 16 1 1 6720 0468 1

3 617 1 8 1 1 1936 0618 1

3 618 2 16 1 2 1931 0468 2

3 619 2 32 1 2 1622 0518 2

3 620 1 8 1 1 1106 0668 1

3 621 2 8 1 2 1295 0518 2

3 622 1 32 1 1 1624 0668 1

3 623 2 8 1 2 832 0568 2

3 624 2 8 1 2 1727 0568 2

3 625 2 32 1 2 1620 0568 2

3 626 1 16 1 1 1498 0668 1

3 627 1 8 1 1 3953 0468 1

3 628 2 16 1 2 3946 0518 2

3 629 2 8 1 2 867 0668 2

3 630 1 32 1 1 2229 0568 1

3 631 1 32 1 1 1654 0618 1

3 632 1 8 1 1 1969 0618 1

3 633 2 16 1 2 1589 0618 2

3 634 1 8 1 1 977 0668 2

3 635 2 32 1 2 1528 0668 2

3 636 2 16 1 2 2809 0568 2

3 637 1 8 1 1 1458 0518 1

3 638 2 8 1 2 1084 0518 2

3 639 1 16 1 1 2013 0618 1

3 640 2 8 1 2 979 0518 1

3 641 2 8 1 2 2208 0618 2

3 642 2 16 1 2 1439 0568 2

3 643 2 32 1 2 1343 0568 2

3 644 1 16 1 1 1007 0568 1

3 645 1 16 1 1 1975 0618 1

3 646 2 32 1 2 1456 0668 2

3 647 2 32 1 2 1256 0518 2

3 648 2 16 1 2 4902 0468 2

3 649 1 32 1 1 1919 0568 1

3 650 2 8 1 2 1108 0668 2

3 651 1 32 1 1 1356 0668 1

3 652 1 32 1 1 2061 0518 1

3 653 2 32 1 2 1429 0618 2

3 654 2 8 1 2 1346 0568 1

3 655 1 32 1 1 2094 0668 1

3 656 1 16 1 1 1869 0618 1

3 657 2 16 1 2 1448 0668 2

3 658 1 32 1 1 1608 0618 1

3 659 2 8 1 2 940 0568 2

3 660 1 16 1 1 994 0668 1

3 661 1 16 1 1 1513 0518 1

3 662 1 8 1 1 3488 0468 1

3 663 2 16 1 2 7284 0468 2

3 664 1 16 1 1 4477 0468 1

3 665 1 32 1 1 1768 0668 1

3 666 1 32 1 1 1134 0568 1

3 667 2 16 1 2 1983 0618 2

3 668 2 16 1 2 1703 0668 2

3 669 2 8 1 2 837 0468 2

3 670 1 8 1 1 1047 0618 1

3 671 1 16 1 1 1793 0618 1

3 672 1 8 1 1 1403 0468 1

3 673 2 16 1 2 1299 0468 2

3 674 1 16 1 1 1170 0568 1

3 675 1 8 1 1 1062 0468 1

3 676 2 32 1 2 1237 0518 2

3 677 1 8 0 1 17605 0468 1

3 678 2 8 1 2 1559 0568 1

3 679 2 32 1 2 2306 0618 2

3 680 1 8 1 1 1721 0568 2

3 681 2 8 1 2 886 0668 2

3 682 1 16 1 1 4486 0468 1

3 683 2 16 1 2 3025 0618 2

3 684 1 32 1 1 1414 0518 1

3 685 2 32 1 2 1225 0668 2

3 686 2 16 1 2 1527 0568 2

3 687 2 32 1 2 830 0518 2

3 688 1 32 1 1 1454 0468 1

3 689 1 16 1 1 2353 0568 1

3 690 1 32 1 1 1506 0618 1

3 691 1 16 1 1 1427 0468 1

3 692 2 32 1 2 1082 0668 2

3 693 1 32 1 1 1150 0518 1

3 694 2 8 1 2 2418 0618 2

3 695 2 32 1 2 1131 0668 2

3 696 1 16 1 1 2024 0518 1

3 697 2 32 1 2 1353 0468 2

3 698 2 32 1 2 968 0468 2

3 699 1 32 1 1 1022 0668 1

3 700 1 16 1 1 1659 0618 1

3 701 2 32 1 2 1169 0618 2

3 702 2 32 1 2 1341 0568 2

3 703 1 8 1 1 1504 0618 1

3 704 1 16 1 1 1385 0668 1

3 705 2 32 1 2 1484 0568 2

3 706 2 8 1 2 853 0668 1

3 707 1 8 1 1 1607 0618 1

3 708 1 8 1 1 1090 0568 1

3 709 2 8 1 2 1054 0468 2

3 710 2 16 0 2 1130 0618 2

3 711 1 8 1 1 930 0468 1

3 712 1 32 1 1 917 0518 1

3 713 1 32 1 1 1388 0518 1

3 714 2 8 1 2 998 0518 2

3 715 1 8 1 1 930 0518 2

3 716 1 16 1 1 1167 0668 1

3 717 1 16 1 1 1162 0568 1

3 718 1 8 1 1 763 0668 2

3 719 2 8 1 2 1183 0468 2

3 720 2 32 1 2 1851 0618 2

3 721 2 16 1 2 957 0518 2

3 722 2 8 1 2 842 0518 1

3 723 2 8 1 2 628 0568 2

3 724 2 16 1 2 1498 0518 2

3 725 1 32 1 1 1397 0568 1

3 726 1 32 1 1 1054 0518 1

3 727 2 16 1 2 1055 0668 2

3 728 2 16 1 2 1601 0568 2

3 729 1 32 1 1 1004 0568 1

3 730 1 32 1 1 1261 0518 1

3 731 2 8 1 2 1218 0518 1

3 732 1 8 1 1 969 0468 1

3 733 2 8 1 2 1413 0518 1

3 734 1 8 1 1 1191 0568 1

3 735 2 8 1 2 1277 0668 2

3 736 2 32 1 2 1759 0568 2

3 737 1 16 1 1 990 0668 1

3 738 1 32 1 1 1271 0618 1

3 739 2 16 0 2 3467 0468 2

3 740 1 32 1 1 3012 0518 1

3 741 2 32 1 2 1269 0518 2

3 742 2 32 1 2 1538 0568 2

3 743 1 8 1 1 1038 0468 1

3 744 1 32 1 1 1169 0568 1

3 745 2 16 1 2 2995 0618 2

3 746 2 32 1 2 2078 0618 2

3 747 1 16 1 1 3263 0618 1

3 748 2 32 1 2 1179 0668 2

3 749 1 8 1 1 1767 0468 1

3 750 1 16 1 1 1812 0568 1

3 751 2 32 1 2 1171 0518 2

3 752 1 32 1 1 1230 0568 1

3 753 1 32 1 1 2015 0618 1

3 754 2 16 1 2 969 0568 2

3 755 2 8 1 2 2739 0518 2

3 756 1 8 1 1 1427 0468 1

3 757 1 8 1 1 955 0568 2

3 758 1 16 1 1 1300 0668 1

3 759 1 32 1 1 979 0568 1

3 760 2 8 1 2 1813 0618 2

3 761 1 32 1 1 2351 0468 1

3 762 1 32 1 1 1117 0468 1

3 763 1 16 1 1 2534 0568 1

3 764 2 32 1 2 1053 0568 2

3 765 2 32 1 2 1027 0618 2

3 766 2 16 1 2 3124 0468 2

3 767 1 32 1 1 1278 0668 1

3 768 2 32 1 2 2995 0668 2

3 769 1 16 1 1 5054 0468 1

3 770 2 32 1 2 873 0618 2

3 771 1 32 1 1 1646 0468 1

3 772 2 32 1 2 1056 0568 2

3 773 2 32 1 2 2557 0518 2

3 774 2 32 1 2 995 0668 2

3 775 1 8 1 1 1007 0568 1

3 776 1 8 1 1 1328 0618 1

3 777 1 8 1 1 1943 0518 1

3 778 1 16 1 1 919 0668 1

3 779 2 8 1 2 938 0668 1

3 780 2 16 1 2 5872 0468 2

3 781 1 16 1 1 1758 0618 1

3 782 2 32 1 2 1217 0468 2

3 783 2 8 1 2 2260 0568 2

3 784 2 8 1 2 899 0668 2

3 785 2 16 1 2 1959 0468 2

3 786 2 32 1 2 1570 0468 2

3 787 2 8 1 2 1109 0568 2

3 788 2 8 1 2 1442 0668 2

3 789 1 32 1 1 1338 0668 1

3 790 1 8 1 1 1056 0518 1

3 791 2 16 1 2 1071 0568 2

3 792 2 32 1 2 1072 0468 2

3 793 2 32 1 2 1142 0468 2

3 794 2 16 1 2 1309 0568 2

3 795 1 32 1 1 1127 0568 1

3 796 2 16 1 2 1026 0468 2

3 797 1 8 1 1 1011 0568 1

3 798 1 16 1 1 2764 0468 1

3 799 2 32 1 2 1118 0618 2

3 800 2 16 1 2 2100 0468 2

3 801 1 16 1 1 1739 0618 1

3 802 2 32 1 2 1020 0668 2

3 803 2 32 0 2 1053 0618 2

3 804 1 8 1 1 1171 0518 2

3 805 2 16 1 2 1086 0618 2

3 806 2 32 1 2 1028 0668 2

3 807 1 8 1 1 1917 0618 1

3 808 1 32 1 1 1622 0468 1

3 809 2 16 1 2 1545 0618 2

3 810 2 32 1 2 920 0568 2

3 811 1 16 1 1 1790 0618 1

3 812 1 16 1 1 1497 0468 1

3 813 1 32 1 1 852 0618 1

3 814 1 32 1 1 971 0618 1

3 815 1 8 1 1 1001 0668 2

3 816 1 8 1 1 2194 0618 1

3 817 1 8 1 1 996 0568 2

3 818 1 16 1 1 4464 0518 1

3 819 2 8 1 2 878 0568 1

3 820 1 8 1 1 938 0468 1

3 821 2 32 1 2 995 0568 2

3 822 2 16 1 2 3185 0618 2

3 823 2 16 1 2 1643 0568 2

3 824 2 16 1 2 1617 0568 2

3 825 1 8 1 1 1545 0568 2

3 826 2 8 1 2 869 0668 1

3 827 1 32 1 1 2210 0668 1

3 828 1 16 1 1 1184 0568 1

3 829 2 16 1 2 1509 0618 2

3 830 1 16 1 1 1666 0618 1

3 831 1 16 1 1 1360 0518 1

3 832 2 32 1 2 1045 0468 2

3 833 2 8 1 2 1879 0468 2

3 834 1 16 1 1 1111 0468 1

3 835 1 16 1 1 1532 0568 1

3 836 2 16 1 2 849 0668 2

3 837 2 8 1 2 1041 0568 2

3 838 1 32 1 1 1154 0518 1

3 839 1 16 1 1 973 0668 1

3 840 2 32 1 2 1112 0468 2

3 841 2 32 1 2 2448 0568 2

3 842 1 32 1 1 1293 0468 1

3 843 1 32 1 1 2478 0518 1

3 844 2 8 1 2 1392 0618 2

3 845 1 16 1 1 1471 0668 1

3 846 2 8 1 2 973 0518 2

3 847 2 8 1 2 827 0518 2

3 848 1 8 1 1 1099 0668 1

3 849 1 8 1 1 1186 0518 2

3 850 1 32 1 1 1053 0518 1

3 851 2 16 1 2 1002 0668 2

3 852 2 16 1 2 1635 0568 2

3 853 2 8 1 2 755 0618 2

3 854 1 16 1 1 1054 0668 1

3 855 1 32 1 1 3747 0518 1

3 856 1 32 1 1 975 0568 1

3 857 2 16 1 2 1367 0518 2

3 858 2 8 1 2 1960 0518 2

3 859 1 16 1 1 1189 0568 1

3 860 2 16 1 2 1113 0668 2

3 861 1 16 1 1 1578 0568 1

3 862 2 8 1 2 903 0568 2

3 863 1 8 1 1 1199 0518 2

3 864 2 16 1 2 947 0668 2

3 865 1 16 1 1 2021 0618 1

3 866 1 8 1 1 5497 0468 1

3 867 1 8 1 1 1431 0468 1

3 868 2 8 1 2 1542 0618 2

3 869 2 8 1 2 1324 0568 1

3 870 1 16 1 1 2288 0468 1

3 871 2 8 1 2 3084 0468 2

3 872 1 8 1 1 1578 0668 1

3 873 2 32 1 2 1713 0518 2

3 874 1 8 1 1 1278 0618 1

3 875 1 32 1 1 2598 0568 1

3 876 2 16 1 2 5140 0518 2

3 877 2 16 1 2 1858 0668 2

3 878 2 16 1 2 1975 0618 2

3 879 1 32 1 1 1570 0468 1

3 880 2 32 1 2 907 0518 2

3 881 1 8 0 1 1225 0568 2

3 882 2 8 1 2 1177 0518 1

3 883 1 16 1 1 1404 0618 1

3 884 2 16 1 2 1309 0468 2

3 885 1 32 1 1 1252 0618 1

3 886 2 8 1 2 1033 0568 2

3 887 2 16 1 2 858 0518 2

3 888 1 16 1 1 994 0518 1

3 889 1 32 1 1 2094 0668 1

3 890 2 32 1 2 1498 0618 2

3 891 1 8 1 1 2207 0618 1

3 892 2 16 1 2 1212 0518 2

3 893 2 32 1 2 2216 0468 2

3 894 2 16 1 2 2373 0518 2

3 895 2 16 1 2 915 0518 2

3 896 1 16 1 1 1684 0468 1

3 897 1 8 1 1 961 0668 2

3 898 1 8 1 1 838 0568 1

3 899 2 32 1 2 1334 0568 2

3 900 1 8 1 1 986 0618 1

3 901 1 32 1 1 959 0618 1

3 902 2 32 1 2 1111 0518 2

3 903 1 8 1 1 831 0668 1

3 904 1 16 1 1 1157 0668 1

3 905 1 8 1 1 770 0668 2

3 906 1 8 1 1 785 0518 2

3 907 2 16 1 2 750 0668 2

3 908 1 16 1 1 999 0518 1

3 909 2 8 1 2 864 0668 2

3 910 1 32 0 1 1280 0668 1

3 911 1 16 1 1 1467 0618 1

3 912 1 8 1 1 928 0518 1

3 913 2 8 1 2 789 0668 2

3 914 2 8 1 2 1514 0618 2

3 915 1 32 1 1 1204 0668 1

3 916 1 8 1 1 1687 0668 2

3 917 1 16 0 1 1651 0518 1

3 918 2 8 1 2 795 0618 2

3 919 2 32 1 2 1040 0518 2

3 920 2 16 1 2 1727 0668 2

3 921 2 8 1 2 2194 0468 2

3 922 1 8 1 1 827 0668 1

3 923 2 16 1 2 3619 0568 2

3 924 1 16 0 1 1074 0518 1

3 925 2 8 1 2 879 0468 2

3 926 1 8 0 1 738 0518 1

3 927 2 16 1 2 1308 0618 2

3 928 2 8 1 2 2099 0618 2

3 929 2 8 1 2 919 0668 2

3 930 1 32 0 1 1055 0468 1

3 931 2 32 1 2 932 0518 2

3 932 1 8 1 1 984 0468 1

3 933 1 8 1 1 1516 0618 1

3 934 1 32 0 1 1007 0468 1

3 935 2 32 1 2 1086 0668 2

3 936 1 32 1 1 1535 0668 1

3 937 2 32 1 2 1818 0468 2

3 938 1 16 1 1 2007 0568 1

3 939 2 32 1 2 1246 0668 2

3 940 1 32 1 1 842 0618 1

3 941 2 8 1 2 959 0468 2

3 942 1 32 1 1 1098 0668 1

3 943 1 32 1 1 1015 0618 1

3 944 1 16 1 1 1853 0568 1

3 945 2 8 1 2 894 0468 2

3 946 1 16 1 1 971 0468 1

3 947 2 32 1 2 1081 0618 2

3 948 2 8 0 2 973 0468 2

3 949 1 16 1 1 3929 0468 1

3 950 2 8 1 2 917 0618 2

3 951 1 16 1 1 2091 0518 1

3 952 2 32 1 2 955 0618 2

3 953 2 8 1 2 2041 0468 2

3 954 1 32 1 1 3200 0518 1

3 955 1 16 1 1 1960 0518 1

3 956 2 16 1 2 1566 0618 2

3 957 1 16 1 1 2202 0668 1

3 958 1 8 1 1 1443 0618 1

3 959 2 32 1 2 1215 0668 2

3 960 2 16 1 2 2584 0468 2

3 961 2 16 1 2 1629 0668 2

3 962 2 8 1 2 1366 0618 2

3 963 1 8 1 1 967 0668 2

3 964 2 16 1 2 915 0668 2

3 965 1 16 1 1 1912 0518 1

3 966 2 32 1 2 1180 0668 2

3 967 1 16 1 1 1398 0668 1

3 968 1 8 1 1 1005 0518 2

3 969 1 8 1 1 1353 0618 1

3 970 2 32 1 2 1319 0468 2

3 971 2 8 1 2 1235 0518 1

3 972 2 8 1 2 1066 0568 2

3 973 1 32 1 1 1230 0468 1

3 974 1 8 1 1 3265 0468 1

3 975 2 32 1 2 2262 0518 2

3 976 1 16 1 1 1874 0468 1

3 977 1 8 0 1 12009 0468 1

3 978 2 16 1 2 2649 0618 2

3 979 2 8 1 2 1587 0618 2

3 980 2 16 1 2 860 0668 2

3 981 1 16 1 1 779 0668 1

3 982 2 32 1 2 1182 0468 2

3 983 1 16 1 1 1232 0618 1

3 984 1 8 1 1 3000 0618 1

3 985 1 16 1 1 3016 0568 1

3 986 2 32 1 2 1128 0468 2

3 987 2 16 1 2 1946 0468 2

3 988 2 8 1 2 1096 0468 2

3 989 1 8 1 1 931 0518 1

3 990 2 32 1 2 1074 0668 2

3 991 1 16 1 1 2544 0468 1

3 992 2 16 1 2 770 0668 2

3 993 2 16 1 2 1476 0518 2

3 994 2 8 1 2 886 0468 2

3 995 2 16 1 2 691 0518 2

3 996 1 32 1 1 1210 0668 1

3 997 1 32 1 1 1057 0468 1

3 998 2 16 0 2 1322 0468 2

3 999 2 8 1 2 1046 0518 2

3 1000 2 16 0 2 887 0618 2

3 1001 1 16 1 1 2016 0518 1

3 1002 2 32 1 2 812 0518 2

3 1003 1 16 0 1 1541 0518 1

3 1004 2 16 1 2 1180 0568 2

3 1005 1 32 1 1 1362 0568 1

3 1006 2 32 1 2 1940 0518 2

3 1007 1 8 1 1 1005 0568 2

3 1008 1 16 1 1 1238 0568 1

3 1009 1 32 1 1 2828 0618 1

3 1010 2 16 1 2 2377 0468 2

3 1011 1 32 1 1 2386 0668 1

3 1012 2 8 1 2 999 0668 2

3 1013 2 8 1 2 1752 0468 2

3 1014 2 32 1 2 1718 0468 2

3 1015 1 16 1 1 1662 0518 1

3 1016 1 8 1 1 3145 0668 1

3 1017 2 32 1 2 1133 0568 2

3 1018 2 32 1 2 1262 0518 2

3 1019 1 16 1 1 1108 0518 1

3 1020 2 16 1 2 1223 0468 2

3 1021 2 16 1 2 1734 0518 2

3 1022 1 16 1 1 2114 0518 1

3 1023 1 32 1 1 1570 0618 1

3 1024 1 8 1 1 747 0668 1

3 1025 1 8 1 1 753 0668 2

3 1026 2 32 1 2 1058 0518 2

3 1027 2 16 1 2 3316 0568 2

3 1028 2 8 1 2 778 0568 1

3 1029 1 16 1 1 1648 0618 1

3 1030 1 32 1 1 2510 0568 1

3 1031 2 8 1 2 1072 0468 2

3 1032 2 16 1 2 2046 0618 2

3 1033 1 8 1 1 972 0568 2

3 1034 2 8 1 2 1173 0618 2

3 1035 2 32 1 2 872 0568 2

3 1036 1 16 1 1 1160 0668 1

3 1037 1 32 1 1 1081 0668 1

3 1038 1 32 1 1 1191 0518 1

3 1039 1 32 1 1 845 0468 1

3 1040 2 8 1 2 792 0618 2

3 1041 1 32 1 1 2069 0518 1

3 1042 1 32 1 1 785 0568 1

3 1043 2 32 1 2 780 0618 2

3 1044 2 8 1 2 1074 0518 1

3 1045 1 16 1 1 973 0568 1

3 1046 1 16 1 1 873 0668 1

3 1047 2 8 1 2 889 0618 2

3 1048 2 32 1 2 1356 0518 2

3 1049 2 32 1 2 1425 0518 2

3 1050 1 8 1 1 850 0468 1

3 1051 1 8 1 1 1198 0468 1

3 1052 2 32 1 2 870 0568 2

3 1053 1 16 1 1 6471 0468 1

3 1054 2 32 1 2 1244 0618 2

3 1055 2 16 1 2 806 0668 2

3 1056 2 32 1 2 962 0668 2

3 1057 2 8 1 2 787 0568 2

3 1058 1 32 1 1 1548 0618 1

3 1059 2 8 1 2 1507 0668 2

3 1060 2 16 1 2 1225 0618 2

3 1061 2 8 1 2 1064 0618 2

3 1062 1 32 1 1 1446 0668 1

3 1063 1 16 1 1 1718 0568 1

3 1064 2 8 1 2 833 0668 1

3 1065 2 32 1 2 847 0618 2

3 1066 2 32 1 2 856 0568 2

3 1067 1 8 1 1 835 0568 2

3 1068 1 32 1 1 1292 0518 1

3 1069 1 16 1 1 3195 0618 1

3 1070 2 8 1 2 922 0468 2

3 1071 2 16 1 2 1801 0668 2

3 1072 2 32 1 2 2520 0668 2

3 1073 2 32 1 2 1084 0468 2

3 1074 2 8 1 2 782 0668 2

3 1075 1 8 1 1 1061 0668 1

3 1076 2 32 1 2 1066 0618 2

3 1077 2 32 1 2 852 0568 2

3 1078 2 32 1 2 737 0568 2

3 1079 2 8 1 2 905 0618 2

3 1080 1 8 1 1 1135 0468 1

3 1081 1 32 1 1 2367 0468 1

3 1082 1 16 1 1 1211 0518 1

3 1083 1 8 1 1 783 0518 1

3 1084 2 16 1 2 879 0568 2

3 1085 1 8 1 1 968 0668 2

3 1086 1 8 1 1 949 0568 1

3 1087 1 8 1 1 933 0468 1

3 1088 1 32 1 1 1044 0518 1

3 1089 1 32 1 1 818 0618 1

3 1090 2 16 1 2 955 0668 2

3 1091 1 8 1 1 745 0468 1

3 1092 1 16 1 1 1917 0468 1

3 1093 1 8 1 1 1086 0618 1

3 1094 2 16 1 2 1261 0518 2

3 1095 1 8 1 1 1795 0618 1

3 1096 2 8 1 2 1944 0468 2

3 1097 2 32 1 2 1076 0668 2

3 1098 1 32 1 1 830 0618 1

3 1099 1 8 1 1 1487 0618 1

3 1100 2 32 1 2 1143 0518 2

3 1101 2 16 1 2 1260 0568 2

3 1102 1 32 1 1 1464 0468 1

3 1103 2 8 1 2 938 0618 2

3 1104 2 16 1 2 2070 0518 2

3 1105 2 16 1 2 1974 0568 2

3 1106 1 32 1 1 1590 0518 1

3 1107 2 32 1 2 1060 0668 2

3 1108 1 16 1 1 2846 0618 1

3 1109 1 16 1 1 1439 0568 1

3 1110 1 32 1 1 1084 0568 1

3 1111 1 16 1 1 1596 0668 1

3 1112 2 8 1 2 1464 0568 2

3 1113 1 16 1 1 894 0668 1

3 1114 2 8 1 2 985 0568 2

3 1115 1 32 1 1 1802 0468 1

3 1116 1 32 1 1 2657 0518 1

3 1117 1 32 1 1 3358 0468 1

3 1118 2 32 1 2 3838 0468 2

3 1119 2 8 1 2 1625 0518 1

3 1120 1 16 1 1 1354 0668 1

3 1121 2 8 1 2 1626 0568 2

3 1122 2 8 1 2 1063 0668 2

3 1123 1 8 1 1 965 0468 1

3 1124 2 16 1 2 1604 0518 2

3 1125 1 16 0 1 1091 0468 1

3 1126 2 8 1 2 733 0568 2

3 1127 2 8 1 2 725 0518 1

3 1128 1 16 1 1 876 0618 1

3 1129 1 8 0 1 745 0518 1

3 1130 2 8 1 2 729 0518 2

3 1131 2 16 1 2 1335 0618 2

3 1132 1 16 1 1 1303 0468 1

3 1133 2 16 1 2 1573 0468 2

3 1134 2 16 1 2 1688 0618 2

3 1135 1 8 1 1 1152 0518 1

3 1136 2 32 1 2 942 0468 2

3 1137 2 32 1 2 993 0668 2

3 1138 1 32 1 1 1369 0618 1

3 1139 1 32 1 1 1975 0618 1

3 1140 1 16 1 1 1060 0518 1

3 1141 1 8 1 1 718 0668 2

3 1142 1 32 1 1 906 0568 1

3 1143 1 8 1 1 1277 0518 2

3 1144 2 32 1 2 937 0468 2

3 1145 1 8 1 1 1184 0618 1

3 1146 1 32 1 1 1124 0668 1

3 1147 1 8 1 1 1261 0618 1

3 1148 2 8 1 2 2243 0518 2

3 1149 1 16 1 1 2930 0468 1

3 1150 1 8 1 1 691 0668 1

3 1151 2 32 1 2 1376 0618 2

3 1152 2 8 1 2 1081 0568 1

3 1153 1 8 1 1 2177 0618 1

3 1154 1 32 1 1 3073 0468 1

3 1155 2 16 1 2 1227 0518 2

3 1156 2 16 1 2 848 0568 2

3 1157 2 8 1 2 842 0518 2

3 1158 2 32 1 2 911 0568 2

3 1159 1 32 1 1 926 0568 1

3 1160 2 16 1 2 2626 0568 2

3 1161 2 16 1 2 2809 0618 2

3 1162 2 32 1 2 1998 0618 2

3 1163 1 16 1 1 2256 0568 1

3 1164 1 8 1 1 1721 0518 2

3 1165 2 8 1 2 1602 0468 2

3 1166 1 8 1 1 1289 0518 2

3 1167 2 16 1 2 2169 0518 2

3 1168 1 16 1 1 2006 0568 1

3 1169 1 8 0 1 1406 0568 1

3 1170 2 32 1 2 1622 0568 2

3 1171 2 16 0 2 1649 0468 2

3 1172 1 32 1 1 1675 0668 1

3 1173 1 16 1 1 824 0568 1

3 1174 2 16 1 2 897 0568 2

3 1175 1 16 1 1 1137 0618 1

3 1176 1 32 1 1 1406 0568 1

3 1177 1 32 1 1 1322 0568 1

3 1178 2 32 1 2 933 0668 2

3 1179 2 16 1 2 1197 0618 2

3 1180 1 16 1 1 1738 0618 1

3 1181 1 8 1 1 1455 0568 1

3 1182 1 32 1 1 1035 0518 1

3 1183 2 32 1 2 3932 0618 2

3 1184 1 8 1 1 1006 0568 2

3 1185 2 8 1 2 870 0668 2

3 1186 1 32 1 1 3038 0668 1

3 1187 1 32 1 1 1048 0668 1

3 1188 1 32 1 1 1724 0518 1

3 1189 1 16 1 1 1523 0468 1

3 1190 1 16 1 1 3386 0668 1

3 1191 2 8 1 2 1189 0668 1

3 1192 2 8 1 2 2028 0468 2

3 1193 2 32 1 2 1021 0618 2

3 1194 2 8 1 2 710 0668 2

3 1195 2 16 1 2 1036 0468 2

3 1196 1 32 1 1 832 0618 1

3 1197 1 8 1 1 838 0568 1

3 1198 2 16 1 2 1882 0468 2

3 1199 2 16 1 2 807 0668 2

3 1200 1 16 1 1 1331 0618 1

4 1 1 32 1 1 4021 0468 1

4 2 2 16 1 2 2286 0668 2

4 3 1 32 1 1 2064 0468 1

4 4 2 8 1 2 1639 0568 2

4 5 2 16 1 2 2172 0518 2

4 6 2 8 1 2 1293 0668 2

4 7 1 16 1 1 1808 0618 1

4 8 2 32 1 2 1919 0518 2

4 9 2 32 1 2 2713 0468 2

4 10 2 32 1 2 2049 0518 2

4 11 1 8 1 1 1153 0568 2

4 12 2 16 1 2 1484 0668 2

4 13 2 32 1 2 2347 0468 2

4 14 2 8 1 2 1418 0518 1

4 15 2 32 1 2 1596 0568 2

4 16 1 8 1 1 1120 0618 1

4 17 1 16 1 1 1946 0668 1

4 18 1 32 1 1 1218 0618 1

4 19 2 8 1 2 2047 0468 2

4 20 1 16 1 1 1841 0568 1

4 21 1 8 1 1 941 0518 2

4 22 1 32 1 1 978 0468 1

4 23 1 16 1 1 1778 0468 1

4 24 2 16 1 2 1593 0618 2

4 25 2 8 1 2 1115 0518 1

4 26 1 8 1 1 1275 0468 1

4 27 2 32 1 2 1079 0618 2

4 28 1 16 1 1 2210 0568 1

4 29 1 8 1 1 1251 0468 1

4 30 1 16 1 1 1702 0618 1

4 31 2 16 1 2 1768 0518 2

4 32 2 16 1 2 1264 0568 2

4 33 1 8 1 1 1002 0618 1

4 34 2 8 1 2 1160 0618 2

4 35 2 16 1 2 2101 0568 2

4 36 2 32 1 2 986 0618 2

4 37 2 8 1 2 886 0618 2

4 38 1 16 1 1 1626 0618 1

4 39 2 32 1 2 1028 0568 2

4 40 2 32 1 2 1210 0618 2

4 41 1 8 1 1 1187 0518 2

4 42 1 8 1 1 848 0568 1

4 43 2 16 1 2 4957 0468 2

4 44 2 16 1 2 1277 0618 2

4 45 2 32 1 2 1034 0468 2

4 46 2 32 1 2 1340 0668 2

4 47 2 16 1 2 1515 0668 2

4 48 1 16 1 1 1642 0518 1

4 49 2 16 1 2 6484 0468 2

4 50 2 8 1 2 1115 0518 2

4 51 1 32 1 1 1708 0668 1

4 52 2 8 1 2 1083 0668 2

4 53 1 32 1 1 1112 0518 1

4 54 1 16 1 1 3835 0518 1

4 55 2 16 1 2 2716 0468 2

4 56 2 32 0 2 1408 0468 2

4 57 2 8 1 2 1219 0568 1

4 58 1 32 1 1 1088 0618 1

4 59 2 8 1 2 982 0568 2

4 60 1 8 1 1 1060 0468 1

4 61 1 16 1 1 1278 0668 1

4 62 2 32 1 2 1356 0568 2

4 63 1 16 1 1 1214 0568 1

4 64 2 8 1 2 960 0468 2

4 65 1 16 1 1 1125 0568 1

4 66 1 8 1 1 1106 0568 2

4 67 2 16 1 2 1069 0668 2

4 68 1 16 1 1 2665 0468 1

4 69 1 16 1 1 1314 0468 1

4 70 1 16 1 1 2491 0468 1

4 71 1 32 1 1 953 0468 1

4 72 1 32 1 1 1439 0468 1

4 73 1 32 1 1 984 0618 1

4 74 1 8 1 1 840 0518 1

4 75 1 16 1 1 1352 0468 1

4 76 1 32 1 1 678 0518 1

4 77 2 32 1 2 1310 0618 2

4 78 2 8 1 2 984 0518 2

4 79 2 8 1 2 980 0618 2

4 80 2 16 1 2 950 0668 2

4 81 1 32 1 1 988 0518 1

4 82 2 16 1 2 1469 0668 2

4 83 1 16 1 1 1017 0668 1

4 84 1 8 1 1 1317 0618 1

4 85 2 8 1 2 904 0518 1

4 86 1 16 1 1 1466 0618 1

4 87 2 16 1 2 822 0568 2

4 88 1 16 1 1 923 0668 1

4 89 2 16 1 2 1649 0618 2

4 90 2 16 0 2 1277 0618 2

4 91 1 16 1 1 916 0668 1

4 92 2 32 1 2 1079 0618 2

4 93 2 8 1 2 961 0618 2

4 94 2 16 1 2 1345 0568 2

4 95 2 8 1 2 1044 0668 2

4 96 2 32 1 2 1240 0668 2

4 97 2 32 1 2 1436 0568 2

4 98 1 8 1 1 1192 0668 1

4 99 2 8 1 2 1893 0468 2

4 100 1 8 1 1 940 0668 1

4 101 2 8 1 2 1057 0568 2

4 102 1 16 1 1 816 0668 1

4 103 1 8 1 1 874 0518 2

4 104 2 32 1 2 2084 0518 2

4 105 2 8 1 2 1231 0668 2

4 106 1 8 1 1 917 0518 1

4 107 1 8 1 1 1009 0468 1

4 108 1 8 1 1 981 0518 1

4 109 2 8 1 2 2880 0468 2

4 110 1 16 1 1 1202 0618 1

4 111 2 32 1 2 1266 0618 2

4 112 2 8 1 2 1566 0568 2

4 113 1 8 1 1 1137 0468 1

4 114 1 8 1 1 1215 0568 1

4 115 1 32 1 1 988 0668 1

4 116 1 16 1 1 1244 0568 1

4 117 2 8 1 2 918 0518 2

4 118 2 16 1 2 1749 0618 2

4 119 1 8 1 1 1311 0618 1

4 120 2 32 1 2 1170 0668 2

4 121 2 32 1 2 1309 0568 2

4 122 2 8 1 2 919 0568 1

4 123 1 32 1 1 960 0668 1

4 124 1 32 1 1 846 0668 1

4 125 2 32 1 2 830 0618 2

4 126 2 16 1 2 1652 0468 2

4 127 2 16 1 2 1127 0618 2

4 128 2 32 1 2 1438 0518 2

4 129 1 8 1 1 1251 0568 1

4 130 2 32 1 2 1047 0518 2

4 131 1 32 1 1 946 0568 1

4 132 1 8 1 1 1377 0618 1

4 133 2 8 1 2 665 0568 2

4 134 1 32 1 1 1771 0468 1

4 135 2 16 1 2 1602 0468 2

4 136 2 16 0 2 802 0668 2

4 137 1 8 1 1 1359 0618 1

4 138 2 32 1 2 1340 0468 2

4 139 1 32 1 1 887 0468 1

4 140 2 16 1 2 968 0668 2

4 141 1 8 1 1 1101 0518 2

4 142 1 32 1 1 1081 0618 1

4 143 2 32 1 2 1087 0468 2

4 144 2 16 1 2 1214 0618 2

4 145 1 8 1 1 1023 0568 2

4 146 1 8 1 1 823 0568 1

4 147 2 8 0 2 851 0468 2

4 148 2 8 1 2 1353 0518 1

4 149 2 32 1 2 962 0618 2

4 150 1 32 1 1 1051 0568 1

4 151 1 32 1 1 1198 0568 1

4 152 1 8 1 1 944 0468 1

4 153 1 16 0 1 915 0618 1

4 154 2 8 1 2 1522 0618 2

4 155 1 16 0 1 1565 0468 1

4 156 1 16 1 1 1589 0518 1

4 157 2 8 1 2 1176 0518 2

4 158 1 32 1 1 934 0568 1

4 159 1 32 1 1 1067 0568 1

4 160 1 16 1 1 1787 0518 1

4 161 2 32 1 2 1090 0568 2

4 162 2 16 0 2 1439 0518 2

4 163 1 8 1 1 1077 0668 2

4 164 1 16 1 1 871 0568 1

4 165 2 16 1 2 1024 0568 2

4 166 2 32 1 2 1184 0668 2

4 167 1 8 1 1 1054 0568 2

4 168 1 32 1 1 988 0618 1

4 169 2 8 1 2 1035 0568 2

4 170 2 16 1 2 1155 0518 2

4 171 1 32 1 1 978 0568 1

4 172 2 32 1 2 1382 0468 2

4 173 1 8 1 1 1024 0618 1

4 174 2 16 1 2 1189 0618 2

4 175 2 32 1 2 1289 0518 2

4 176 1 16 0 1 1345 0468 1

4 177 1 16 1 1 1394 0568 1

4 178 1 32 1 1 923 0668 1

4 179 2 8 1 2 1228 0468 2

4 180 1 16 1 1 874 0668 1

4 181 1 32 1 1 804 0668 1

4 182 1 32 1 1 747 0568 1

4 183 2 8 1 2 1133 0468 2

4 184 2 32 1 2 921 0668 2

4 185 1 32 1 1 1412 0518 1

4 186 2 16 1 2 1286 0568 2

4 187 1 32 1 1 692 0618 1

4 188 2 8 1 2 914 0618 2

4 189 1 32 1 1 766 0618 1

4 190 2 8 1 2 1001 0668 2

4 191 1 16 1 1 1499 0618 1

4 192 2 32 1 2 1000 0518 2

4 193 2 16 1 2 3575 0468 2

4 194 1 8 1 1 1488 0668 1

4 195 2 8 1 2 2723 0468 2

4 196 2 8 1 2 905 0618 2

4 197 1 32 1 1 811 0568 1

4 198 1 16 1 1 1620 0518 1

4 199 1 32 1 1 869 0518 1

4 200 1 16 0 1 1006 0468 1

4 201 2 16 1 2 1984 0518 2

4 202 2 8 1 2 945 0668 1

4 203 1 32 1 1 804 0668 1

4 204 2 16 1 2 1500 0518 2

4 205 1 8 1 1 1079 0618 1

4 206 2 16 1 2 1295 0518 2

4 207 2 32 1 2 988 0668 2

4 208 1 8 1 1 987 0468 1

4 209 2 8 1 2 1160 0668 2

4 210 2 32 1 2 1103 0468 2

4 211 1 32 1 1 855 0468 1

4 212 1 32 1 1 749 0518 1

4 213 2 32 1 2 802 0668 2

4 214 1 8 1 1 1063 0668 2

4 215 1 32 1 1 935 0618 1

4 216 2 16 0 2 1188 0468 2

4 217 1 8 1 1 1204 0518 1

4 218 1 8 1 1 1551 0468 1

4 219 1 16 1 1 1134 0518 1

4 220 2 16 1 2 1066 0518 2

4 221 1 32 1 1 1105 0668 1

4 222 2 16 1 2 2130 0468 2

4 223 2 16 1 2 1044 0568 2

4 224 1 16 1 1 1006 0618 1

4 225 2 16 1 2 841 0568 2

4 226 1 32 1 1 725 0518 1

4 227 1 16 0 1 987 0518 1

4 228 2 32 0 2 703 0568 2

4 229 2 32 1 2 1217 0668 2

4 230 1 16 1 1 1008 0568 1

4 231 1 16 1 1 1148 0518 1

4 232 1 8 1 1 819 0668 2

4 233 1 8 1 1 627 0668 1

4 234 1 16 1 1 778 0668 1

4 235 1 32 1 1 872 0518 1

4 236 2 8 1 2 817 0618 2

4 237 2 32 1 2 1178 0568 2

4 238 2 32 1 2 793 0518 2

4 239 1 8 1 1 922 0668 2

4 240 2 8 1 2 963 0668 1

4 241 1 8 1 1 1332 0518 1

4 242 1 16 1 1 1054 0618 1

4 243 2 8 1 2 813 0568 1

4 244 2 32 1 2 1068 0618 2

4 245 2 8 1 2 1314 0518 2

4 246 1 8 1 1 904 0518 1

4 247 1 8 1 1 849 0668 1

4 248 1 32 1 1 765 0568 1

4 249 1 8 1 1 740 0468 1

4 250 2 8 1 2 1138 0518 2

4 251 1 8 1 1 968 0618 1

4 252 1 8 1 1 957 0568 2

4 253 1 32 1 1 750 0468 1

4 254 2 32 1 2 1108 0568 2

4 255 2 32 1 2 830 0618 2

4 256 1 32 1 1 780 0618 1

4 257 2 16 1 2 1297 0618 2

4 258 2 8 1 2 1366 0568 2

4 259 2 8 1 2 762 0668 2

4 260 2 16 1 2 2689 0618 2

4 261 2 32 1 2 1257 0568 2

4 262 1 32 1 1 874 0668 1

4 263 2 8 1 2 1385 0468 2

4 264 2 8 1 2 999 0668 2

4 265 2 8 1 2 865 0468 2

4 266 1 16 1 1 1058 0468 1

4 267 2 8 1 2 1057 0468 2

4 268 1 32 1 1 988 0468 1

4 269 1 32 1 1 685 0568 1

4 270 2 32 1 2 844 0618 2

4 271 1 16 1 1 1297 0618 1

4 272 1 16 1 1 1399 0518 1

4 273 2 16 1 2 1357 0468 2

4 274 1 8 1 1 1038 0468 1

4 275 2 16 1 2 1094 0618 2

4 276 1 8 1 1 938 0668 2

4 277 2 8 1 2 795 0468 2

4 278 2 32 0 2 785 0518 2

4 279 1 8 1 1 1154 0468 1

4 280 2 8 1 2 842 0668 1

4 281 2 8 0 2 1210 0668 2

4 282 2 32 1 2 1255 0668 2

4 283 2 32 1 2 979 0668 2

4 284 1 32 1 1 1028 0668 1

4 285 1 8 1 1 1725 0618 1

4 286 2 8 1 2 795 0618 2

4 287 1 16 1 1 1181 0618 1

4 288 1 16 1 1 941 0668 1

4 289 1 16 1 1 1059 0618 1

4 290 2 32 1 2 900 0618 2

4 291 1 32 1 1 1447 0468 1

4 292 1 16 1 1 954 0618 1

4 293 1 8 1 1 837 0668 2

4 294 1 8 1 1 698 0568 2

4 295 2 16 1 2 1181 0518 2

4 296 1 8 1 1 876 0568 1

4 297 2 16 1 2 1093 0468 2

4 298 2 32 1 2 890 0618 2

4 299 2 32 1 2 1104 0568 2

4 300 1 32 1 1 870 0518 1

4 301 1 8 1 1 1053 0618 1

4 302 2 16 1 2 1161 0568 2

4 303 1 8 1 1 938 0668 2

4 304 2 32 1 2 1461 0468 2

4 305 2 32 1 2 924 0568 2

4 306 1 16 1 1 1168 0568 1

4 307 1 8 1 1 987 0668 1

4 308 2 8 1 2 1172 0468 2

4 309 2 32 1 2 1309 0518 2

4 310 1 8 1 1 836 0618 1

4 311 1 16 1 1 1085 0618 1

4 312 1 32 1 1 692 0518 1

4 313 1 32 1 1 907 0518 1

4 314 2 8 1 2 729 0618 2

4 315 1 32 1 1 704 0568 1

4 316 2 32 1 2 849 0568 2

4 317 2 16 1 2 1184 0568 2

4 318 2 16 1 2 1180 0618 2

4 319 1 8 1 1 779 0618 1

4 320 2 16 1 2 827 0668 2

4 321 1 32 1 1 734 0568 1

4 322 1 16 1 1 1030 0468 1

4 323 2 16 1 2 1037 0518 2

4 324 2 8 1 2 771 0518 1

4 325 2 8 1 2 792 0518 1

4 326 2 16 1 2 762 0668 2

4 327 1 32 1 1 698 0568 1

4 328 1 8 1 1 705 0668 1

4 329 1 16 1 1 1567 0668 1

4 330 1 32 1 1 835 0518 1

4 331 1 8 1 1 954 0618 1

4 332 1 32 1 1 750 0668 1

4 333 1 32 1 1 636 0618 1

4 334 1 32 1 1 624 0668 1

4 335 1 16 1 1 979 0568 1

4 336 1 32 1 1 879 0518 1

4 337 1 16 1 1 954 0568 1

4 338 2 8 1 2 1202 0618 2

4 339 1 8 1 1 860 0618 1

4 340 2 16 1 2 1072 0468 2

4 341 1 32 1 1 725 0618 1

4 342 1 16 1 1 1138 0518 1

4 343 2 16 0 2 1670 0468 2

4 344 1 16 1 1 981 0668 1

4 345 2 32 1 2 758 0518 2

4 346 2 16 1 2 839 0518 2

4 347 1 8 1 1 1024 0668 2

4 348 2 32 1 2 1030 0468 2

4 349 2 32 1 2 794 0618 2

4 350 2 32 1 2 1061 0518 2

4 351 1 16 1 1 857 0668 1

4 352 1 16 0 1 1104 0468 1

4 353 2 32 1 2 1359 0468 2

4 354 1 16 1 1 777 0668 1

4 355 1 8 1 1 1741 0468 1

4 356 2 8 1 2 920 0668 2

4 357 2 16 1 2 1137 0468 2

4 358 2 8 1 2 815 0518 2

4 359 2 32 1 2 939 0668 2

4 360 2 32 1 2 1191 0468 2

4 361 1 16 0 1 1228 0468 1

4 362 1 32 1 1 1149 0518 1

4 363 1 16 1 1 1764 0518 1

4 364 2 32 1 2 1053 0618 2

4 365 1 8 1 1 902 0518 2

4 366 1 8 1 1 1108 0518 2

4 367 1 16 1 1 1267 0618 1

4 368 1 32 1 1 690 0518 1

4 369 1 8 1 1 802 0568 1

4 370 1 32 1 1 679 0618 1

4 371 2 8 1 2 867 0518 1

4 372 2 16 1 2 651 0518 2

4 373 1 16 1 1 922 0568 1

4 374 1 32 1 1 1027 0618 1

4 375 2 8 1 2 874 0618 2

4 376 2 32 1 2 1033 0518 2

4 377 2 8 1 2 796 0568 2

4 378 1 32 1 1 861 0468 1

4 379 2 16 1 2 820 0668 2

4 380 2 16 1 2 837 0618 2

4 381 2 32 1 2 1024 0568 2

4 382 2 16 1 2 711 0668 2

4 383 1 32 1 1 915 0568 1

4 384 2 16 1 2 794 0668 2

4 385 1 32 1 1 790 0668 1

4 386 1 8 0 1 914 0568 2

4 387 2 8 1 2 1501 0568 1

4 388 2 16 1 2 1016 0468 2

4 389 2 16 1 2 739 0668 2

4 390 1 8 1 1 716 0668 1

4 391 1 32 1 1 672 0668 1

4 392 1 16 1 1 1256 0518 1

4 393 2 32 0 2 810 0468 2

4 394 2 16 1 2 999 0468 2

4 395 2 16 1 2 617 0668 2

4 396 2 8 1 2 618 0668 2

4 397 2 32 0 2 843 0468 2

4 398 1 8 0 1 1097 0568 1

4 399 1 16 0 1 1559 0468 1

4 400 2 16 1 2 1223 0618 2

4 401 1 16 1 1 761 0668 1

4 402 2 8 1 2 949 0568 2

4 403 2 16 1 2 830 0668 2

4 404 2 32 1 2 1103 0568 2

4 405 2 32 0 2 858 0668 2

4 406 2 16 1 2 1251 0618 2

4 407 2 8 1 2 853 0618 2

4 408 2 8 1 2 889 0518 2

4 409 1 16 0 1 853 0468 1

4 410 1 16 1 1 791 0668 1

4 411 2 32 1 2 1027 0668 2

4 412 1 16 1 1 1127 0518 1

4 413 2 32 1 2 1071 0568 2

4 414 1 16 1 1 777 0668 1

4 415 2 8 1 2 959 0668 1

4 416 1 32 1 1 1110 0468 1

4 417 1 32 1 1 920 0668 1

4 418 2 32 1 2 1046 0468 2

4 419 1 16 1 1 1330 0618 1

4 420 2 8 1 2 731 0618 2

4 421 2 16 1 2 843 0518 2

4 422 2 8 1 2 672 0468 2

4 423 1 8 1 1 853 0518 2

4 424 1 16 1 1 770 0518 1

4 425 2 8 1 2 745 0568 2

4 426 1 32 1 1 795 0618 1

4 427 2 32 1 2 1130 0518 2

4 428 1 8 1 1 872 0518 1

4 429 2 8 1 2 1049 0618 2

4 430 1 16 0 1 1133 0468 1

4 431 2 8 1 2 1092 0568 2

4 432 2 32 1 2 936 0618 2

4 433 1 32 1 1 1954 0668 1

4 434 2 32 1 2 832 0668 2

4 435 1 32 1 1 1010 0468 1

4 436 1 32 1 1 656 0518 1

4 437 1 8 1 1 782 0618 1

4 438 1 16 1 1 656 0568 1

4 439 1 16 1 1 722 0568 1

4 440 1 8 1 1 728 0518 1

4 441 1 8 1 1 834 0468 1

4 442 2 8 0 2 630 0468 2

4 443 2 8 1 2 987 0668 2

4 444 1 8 1 1 779 0518 2

4 445 2 16 0 2 1264 0518 2

4 446 2 16 1 2 1045 0518 2

4 447 2 8 1 2 796 0468 2

4 448 2 16 1 2 895 0568 2

4 449 1 32 1 1 1449 0568 1

4 450 1 8 1 1 918 0568 1

4 451 2 32 1 2 1126 0668 2

4 452 2 32 1 2 803 0518 2

4 453 1 8 1 1 1064 0468 1

4 454 2 32 1 2 1093 0518 2

4 455 2 16 1 2 1447 0618 2

4 456 1 32 1 1 973 0468 1

4 457 2 16 1 2 978 0568 2

4 458 1 16 1 1 890 0568 1

4 459 1 32 1 1 816 0618 1

4 460 1 32 1 1 856 0568 1

4 461 1 16 1 1 1020 0518 1

4 462 2 32 1 2 912 0468 2

4 463 2 16 1 2 1347 0568 2

4 464 1 16 0 1 772 0468 1

4 465 2 16 1 2 1092 0568 2

4 466 1 8 1 1 1141 0468 1

4 467 1 32 1 1 1359 0468 1

4 468 1 16 1 1 914 0518 1

4 469 2 16 1 2 1043 0568 2

4 470 2 32 1 2 965 0668 2

4 471 1 32 1 1 831 0618 1

4 472 2 16 1 2 1170 0468 2

4 473 1 16 0 1 1093 0568 1

4 474 2 8 1 2 1087 0518 1

4 475 1 8 1 1 812 0568 2

4 476 2 8 1 2 815 0618 2

4 477 2 8 1 2 819 0568 2

4 478 2 16 0 2 978 0568 2

4 479 1 8 1 1 2492 0468 1

4 480 2 16 1 2 1124 0518 2

4 481 2 32 1 2 1016 0668 2

4 482 2 8 1 2 1231 0518 1

4 483 2 32 1 2 792 0618 2

4 484 1 32 1 1 1310 0518 1

4 485 2 16 1 2 1745 0568 2

4 486 2 8 1 2 1119 0468 2

4 487 1 8 1 1 955 0568 1

4 488 1 32 1 1 781 0568 1

4 489 2 8 1 2 828 0568 1

4 490 1 16 0 1 784 0518 1

4 491 2 8 1 2 837 0668 1

4 492 2 32 1 2 823 0468 2

4 493 1 8 1 1 779 0668 1

4 494 2 8 1 2 728 0618 2

4 495 2 32 1 2 1067 0668 2

4 496 1 32 1 1 871 0518 1

4 497 1 32 1 1 806 0518 1

4 498 2 32 1 2 921 0568 2

4 499 2 32 1 2 770 0618 2

4 500 1 32 1 1 713 0568 1

4 501 2 16 1 2 1097 0518 2

4 502 2 16 1 2 837 0668 2

4 503 2 8 1 2 929 0518 2

4 504 1 8 1 1 839 0518 1

4 505 1 16 1 1 786 0668 1

4 506 1 8 1 1 614 0668 2

4 507 2 16 1 2 876 0518 2

4 508 1 8 1 1 835 0568 1

4 509 1 16 1 1 708 0468 1

4 510 1 8 1 1 703 0518 2

4 511 1 16 1 1 667 0568 1

4 512 1 8 1 1 734 0568 1

4 513 1 16 1 1 644 0668 1

4 514 2 16 1 2 720 0668 2

4 515 2 32 1 2 1598 0468 2

4 516 1 32 1 1 937 0668 1

4 517 2 32 1 2 1003 0518 2

4 518 1 32 1 1 816 0618 1

4 519 1 16 1 1 1204 0618 1

4 520 1 16 1 1 1019 0518 1

4 521 1 16 1 1 1023 0618 1

4 522 2 8 1 2 984 0468 2

4 523 2 8 1 2 913 0568 2

4 524 2 16 1 2 748 0618 2

4 525 1 8 1 1 968 0518 1

4 526 1 8 1 1 638 0668 1

4 527 1 32 1 1 731 0568 1

4 528 1 32 1 1 766 0518 1

4 529 2 8 1 2 923 0568 2

4 530 2 16 1 2 663 0568 2

4 531 1 16 1 1 955 0568 1

4 532 2 32 1 2 1345 0618 2

4 533 2 16 1 2 1140 0668 2

4 534 2 16 1 2 739 0618 2

4 535 2 16 1 2 901 0518 2

4 536 2 32 1 2 1041 0568 2

4 537 1 32 1 1 812 0618 1

4 538 1 32 1 1 1017 0468 1

4 539 1 8 1 1 851 0468 1

4 540 2 16 1 2 997 0568 2

4 541 2 8 1 2 610 0668 1

4 542 1 16 0 1 707 0518 1

4 543 2 8 1 2 970 0668 2

4 544 1 16 1 1 825 0518 1

4 545 1 8 1 1 720 0568 2

4 546 2 32 0 2 627 0518 2

4 547 1 16 1 1 1147 0518 1

4 548 2 16 1 2 824 0518 2

4 549 2 32 1 2 828 0618 2

4 550 1 8 1 1 840 0618 1

4 551 2 32 1 2 1027 0518 2

4 552 1 8 1 1 844 0618 1

4 553 2 16 1 2 746 0668 2

4 554 2 8 1 2 631 0668 2

4 555 2 32 1 2 1637 0668 2

4 556 1 16 1 1 1007 0468 1

4 557 1 32 1 1 668 0568 1

4 558 1 32 1 1 595 0468 1

4 559 1 16 1 1 780 0618 1

4 560 2 16 1 2 1489 0618 2

4 561 1 32 1 1 908 0668 1

4 562 2 8 1 2 824 0618 2

4 563 1 16 1 1 774 0518 1

4 564 2 32 1 2 1062 0568 2

4 565 1 8 1 1 789 0468 1

4 566 2 8 1 2 676 0618 2

4 567 1 8 1 1 672 0518 2

4 568 2 32 1 2 835 0618 2

4 569 2 16 0 2 1125 0518 2

4 570 1 8 0 1 699 0668 2

4 571 2 32 1 2 1354 0518 2

4 572 2 16 1 2 810 0518 2

4 573 1 8 1 1 708 0568 2

4 574 2 8 1 2 821 0618 2

4 575 1 8 1 1 844 0468 1

4 576 2 16 0 2 824 0568 2

4 577 2 8 1 2 940 0568 2

4 578 1 16 1 1 838 0618 1

4 579 2 32 1 2 885 0668 2

4 580 1 16 0 1 1274 0468 1

4 581 1 16 1 1 683 0668 1

4 582 2 8 1 2 746 0668 2

4 583 2 32 1 2 1206 0668 2

4 584 1 32 1 1 807 0618 1

4 585 1 16 1 1 1025 0518 1

4 586 1 16 0 1 1077 0568 1

4 587 1 16 1 1 1889 0468 1

4 588 1 8 1 1 921 0668 2

4 589 1 16 1 1 857 0568 1

4 590 1 16 1 1 713 0568 1

4 591 2 16 1 2 1047 0468 2

4 592 1 32 1 1 793 0568 1

4 593 2 16 1 2 1076 0468 2

4 594 2 16 0 2 733 0468 2

4 595 2 16 1 2 1420 0618 2

4 596 1 32 1 1 831 0518 1

4 597 1 32 1 1 625 0518 1

4 598 2 8 1 2 1023 0668 2

4 599 2 32 1 2 926 0668 2

4 600 2 16 0 2 865 0468 2

4 601 2 8 1 2 1448 0468 2

4 602 2 8 1 2 1107 0518 2

4 603 2 8 1 2 1393 0618 2

4 604 1 16 1 1 868 0668 1

4 605 2 32 1 2 865 0618 2

4 606 2 16 1 2 828 0618 2

4 607 1 32 1 1 841 0668 1

4 608 1 8 1 1 909 0468 1

4 609 1 32 1 1 874 0568 1

4 610 2 32 1 2 1101 0518 2

4 611 2 32 1 2 1083 0568 2

4 612 2 8 1 2 1203 0518 1

4 613 2 32 1 2 822 0568 2

4 614 1 32 1 1 816 0468 1

4 615 2 8 1 2 768 0618 2

4 616 1 8 1 1 831 0468 1

4 617 1 16 0 1 1095 0468 1

4 618 1 32 1 1 872 0668 1

4 619 2 32 1 2 1428 0468 2

4 620 1 32 1 1 952 0668 1

4 621 2 16 1 2 1605 0468 2

4 622 1 32 1 1 835 0618 1

4 623 2 8 1 2 818 0468 2

4 624 1 16 0 1 697 0468 1

4 625 2 16 1 2 4716 0468 2

4 626 2 32 1 2 1085 0668 2

4 627 1 8 1 1 932 0618 1

4 628 1 8 1 1 1299 0618 1

4 629 2 16 1 2 1116 0468 2

4 630 2 8 1 2 1032 0468 2

4 631 2 16 1 2 1021 0618 2

4 632 1 8 1 1 904 0668 1

4 633 1 16 1 1 1031 0568 1

4 634 2 16 1 2 1178 0518 2

4 635 2 16 1 2 983 0568 2

4 636 1 16 1 1 1528 0468 1

4 637 2 8 1 2 1127 0518 1

4 638 2 16 1 2 862 0668 2

4 639 2 16 1 2 758 0518 2

4 640 1 16 1 1 907 0568 1

4 641 1 8 1 1 1118 0518 2

4 642 1 32 1 1 847 0618 1

4 643 1 32 1 1 632 0618 1

4 644 2 8 1 2 978 0568 2

4 645 1 32 1 1 834 0668 1

4 646 2 8 1 2 1073 0518 2

4 647 2 16 1 2 856 0468 2

4 648 2 8 1 2 1136 0468 2

4 649 2 8 1 2 817 0468 2

4 650 1 32 1 1 703 0618 1

4 651 1 16 1 1 1063 0568 1

4 652 1 32 1 1 985 0468 1

4 653 1 8 1 1 911 0618 1

4 654 2 32 0 2 614 0468 2

4 655 2 32 1 2 901 0568 2

4 656 1 32 1 1 733 0568 1

4 657 2 32 1 2 1121 0568 2

4 658 2 32 1 2 867 0668 2

4 659 1 8 1 1 967 0468 1

4 660 1 32 1 1 844 0668 1

4 661 2 8 1 2 928 0468 2

4 662 2 16 1 2 813 0568 2

4 663 2 8 1 2 683 0518 2

4 664 2 32 1 2 831 0618 2

4 665 2 16 1 2 726 0668 2

4 666 2 8 1 2 687 0518 1

4 667 2 32 1 2 830 0518 2

4 668 2 32 1 2 653 0468 2

4 669 2 32 1 2 947 0618 2

4 670 1 8 1 1 848 0618 1

4 671 1 16 1 1 906 0668 1

4 672 1 32 1 1 999 0468 1

4 673 2 32 1 2 768 0518 2

4 674 1 16 1 1 887 0668 1

4 675 2 16 1 2 837 0568 2

4 676 2 16 1 2 638 0668 2

4 677 1 8 1 1 727 0518 1

4 678 1 16 1 1 805 0618 1

4 679 2 8 1 2 745 0568 1

4 680 1 8 1 1 736 0618 1

4 681 2 8 1 2 969 0618 2

4 682 1 32 1 1 818 0468 1

4 683 2 16 0 2 837 0618 2

4 684 1 8 1 1 638 0518 1

4 685 1 16 1 1 733 0618 1

4 686 2 8 1 2 655 0668 2

4 687 1 16 0 1 909 0468 1

4 688 1 8 0 1 687 0668 1

4 689 1 8 1 1 737 0468 1

4 690 2 8 1 2 749 0568 2

4 691 1 8 1 1 808 0518 2

4 692 1 32 1 1 599 0468 1

4 693 2 16 0 2 715 0618 2

4 694 2 16 1 2 905 0568 2

4 695 1 16 1 1 1055 0618 1

4 696 1 8 1 1 888 0568 2

4 697 1 8 1 1 859 0618 1

4 698 1 16 1 1 691 0668 1

4 699 1 8 1 1 589 0568 2

4 700 1 32 1 1 768 0668 1

4 701 2 8 1 2 724 0618 2

4 702 1 8 0 1 788 0468 1

4 703 1 32 1 1 2003 0518 1

4 704 1 8 1 1 717 0668 2

4 705 1 16 1 1 884 0518 1

4 706 1 32 1 1 561 0518 1

4 707 1 16 1 1 702 0668 1

4 708 2 32 0 2 641 0468 2

4 709 2 32 1 2 984 0468 2

4 710 1 32 1 1 856 0568 1

4 711 2 8 1 2 918 0668 2

4 712 1 32 1 1 792 0618 1

4 713 2 16 1 2 921 0668 2

4 714 1 16 1 1 1394 0618 1

4 715 2 8 1 2 1010 0568 2

4 716 2 32 1 2 814 0568 2

4 717 1 8 1 1 1012 0568 1

4 718 2 32 1 2 1204 0518 2

4 719 1 32 1 1 715 0468 1

4 720 2 32 1 2 1258 0468 2

4 721 2 8 1 2 1032 0568 2

4 722 1 32 1 1 766 0468 1

4 723 2 32 1 2 860 0618 2

4 724 1 8 1 1 781 0618 1

4 725 2 8 1 2 718 0568 2

4 726 2 8 1 2 1101 0518 1

4 727 2 8 1 2 698 0468 2

4 728 1 8 1 1 798 0518 1

4 729 2 8 1 2 939 0568 2

4 730 2 16 0 2 1135 0468 2

4 731 2 16 0 2 1694 0468 2

4 732 2 32 1 2 1429 0468 2

4 733 2 16 1 2 1794 0618 2

4 734 2 8 1 2 871 0568 2

4 735 2 8 1 2 727 0618 2

4 736 1 8 1 1 797 0568 1

4 737 1 32 1 1 673 0468 1

4 738 2 16 1 2 1026 0518 2

4 739 1 32 1 1 1141 0468 1

4 740 1 32 1 1 665 0518 1

4 741 2 32 1 2 836 0618 2

4 742 1 8 0 1 1003 0618 1

4 743 2 16 1 2 841 0668 2

4 744 1 32 1 1 685 0468 1

4 745 1 32 1 1 694 0618 1

4 746 2 32 1 2 851 0618 2

4 747 1 16 1 1 1033 0568 1

4 748 1 32 1 1 628 0518 1

4 749 1 8 1 1 810 0618 1

4 750 2 32 1 2 842 0668 2

4 751 1 32 1 1 905 0568 1

4 752 1 16 1 1 1309 0468 1

4 753 1 32 1 1 762 0518 1

4 754 1 16 0 1 834 0618 1

4 755 2 16 1 2 1337 0568 2

4 756 1 8 1 1 940 0468 1

4 757 1 16 1 1 971 0618 1

4 758 2 16 1 2 1861 0518 2

4 759 1 32 1 1 847 0618 1

4 760 2 16 1 2 1828 0468 2

4 761 2 32 1 2 1245 0568 2

4 762 1 16 1 1 1149 0468 1

4 763 1 8 1 1 877 0618 1

4 764 1 8 1 1 764 0518 1

4 765 1 32 1 1 698 0668 1

4 766 1 32 1 1 617 0618 1

4 767 1 8 1 1 753 0518 1

4 768 1 16 1 1 810 0668 1

4 769 2 8 1 2 828 0568 1

4 770 2 32 1 2 822 0568 2

4 771 1 16 1 1 982 0518 1

4 772 1 16 1 1 1231 0468 1

4 773 2 16 1 2 901 0518 2

4 774 2 32 1 2 805 0568 2

4 775 1 8 1 1 916 0568 2

4 776 2 32 1 2 1717 0518 2

4 777 1 16 1 1 1078 0618 1

4 778 1 16 1 1 871 0618 1

4 779 1 16 1 1 918 0468 1

4 780 2 16 1 2 823 0518 2

4 781 1 8 1 1 680 0668 2

4 782 2 8 1 2 797 0518 2

4 783 1 16 1 1 1316 0518 1

4 784 1 8 1 1 685 0618 1

4 785 1 16 1 1 625 0668 1

4 786 2 32 0 2 792 0668 2

4 787 1 16 1 1 1320 0568 1

4 788 2 32 1 2 815 0518 2

4 789 2 32 1 2 685 0618 2

4 790 2 32 1 2 1362 0518 2

4 791 2 8 1 2 833 0568 2

4 792 1 8 1 1 1071 0518 1

4 793 1 16 1 1 2765 0568 1

4 794 2 32 1 2 1006 0468 2

4 795 1 32 1 1 897 0668 1

4 796 2 16 1 2 1024 0468 2

4 797 2 8 1 2 726 0518 1

4 798 1 8 1 1 671 0568 2

4 799 1 8 1 1 882 0668 2

4 800 1 32 1 1 904 0618 1

4 801 1 8 1 1 745 0568 2

4 802 2 32 0 2 797 0518 2

4 803 1 8 1 1 959 0518 2

4 804 2 16 1 2 2564 0618 2

4 805 2 8 1 2 679 0468 2

4 806 2 8 1 2 635 0468 2

4 807 1 8 1 1 950 0468 1

4 808 2 8 1 2 760 0568 2

4 809 2 32 1 2 944 0568 2

4 810 1 16 1 1 982 0518 1

4 811 1 32 1 1 727 0618 1

4 812 1 8 1 1 1031 0668 1

4 813 2 8 1 2 706 0568 1

4 814 1 32 1 1 763 0668 1

4 815 1 8 1 1 1094 0618 1

4 816 2 8 1 2 1102 0468 2

4 817 1 16 1 1 950 0618 1

4 818 2 32 0 2 717 0468 2

4 819 2 32 0 2 929 0668 2

4 820 2 16 0 2 2108 0468 2

4 821 2 32 1 2 1312 0668 2

4 822 1 16 1 1 857 0668 1

4 823 1 8 1 1 1022 0668 2

4 824 1 8 1 1 940 0568 1

4 825 2 8 1 2 1132 0668 2

4 826 2 32 1 2 1232 0568 2

4 827 1 16 1 1 1608 0618 1

4 828 1 32 1 1 862 0568 1

4 829 2 16 1 2 1402 0568 2

4 830 2 32 1 2 1484 0468 2

4 831 1 16 1 1 1046 0618 1

4 832 1 8 1 1 727 0518 2

4 833 2 8 1 2 907 0668 2

4 834 1 16 1 1 946 0518 1

4 835 1 16 1 1 782 0668 1

4 836 2 8 1 2 744 0668 1

4 837 1 16 0 1 1081 0568 1

4 838 1 32 1 1 879 0668 1

4 839 1 16 1 1 884 0668 1

4 840 1 32 1 1 684 0668 1

4 841 1 32 1 1 768 0568 1

4 842 2 8 1 2 847 0618 2

4 843 2 16 1 2 1213 0568 2

4 844 1 32 1 1 1181 0468 1

4 845 2 16 1 2 948 0668 2

4 846 2 32 1 2 852 0518 2

4 847 2 16 1 2 784 0668 2

4 848 1 32 1 1 969 0618 1

4 849 1 32 0 1 537 0568 1

4 850 1 8 1 1 1167 0468 1

4 851 1 8 1 1 697 0468 1

4 852 2 16 1 2 814 0618 2

4 853 1 16 1 1 1324 0518 1

4 854 2 32 1 2 1016 0668 2

4 855 2 16 1 2 856 0618 2

4 856 1 32 1 1 709 0468 1

4 857 2 8 1 2 700 0668 2

4 858 1 8 1 1 886 0568 2

4 859 1 8 0 1 1409 0468 1

4 860 2 8 1 2 1142 0518 2

4 861 2 8 1 2 782 0618 2

4 862 1 32 1 1 735 0468 1

4 863 1 32 1 1 757 0668 1

4 864 2 16 0 2 1985 0518 2

4 865 2 16 1 2 1052 0668 2

4 866 2 8 1 2 815 0618 2

4 867 1 16 0 1 872 0468 1

4 868 1 16 1 1 1345 0518 1

4 869 2 32 1 2 1989 0518 2

4 870 2 8 1 2 859 0618 2

4 871 1 8 0 1 1664 0468 1

4 872 2 16 1 2 942 0618 2

4 873 2 32 1 2 903 0568 2

4 874 2 16 1 2 3645 0668 2

4 875 2 16 0 2 843 0568 2

4 876 1 32 1 1 769 0568 1

4 877 2 16 1 2 1188 0568 2

4 878 2 8 1 2 658 0618 2

4 879 2 8 1 2 735 0668 2

4 880 2 32 1 2 874 0618 2

4 881 1 32 1 1 852 0668 1

4 882 1 32 1 1 974 0518 1

4 883 2 8 1 2 976 0468 2

4 884 1 8 1 1 748 0668 1

4 885 2 32 1 2 1963 0468 2

4 886 1 8 1 1 1356 0668 1

4 887 2 16 0 2 1073 0568 2

4 888 1 16 1 1 2221 0468 1

4 889 2 16 0 2 1509 0468 2

4 890 1 16 1 1 1348 0618 1

4 891 1 16 1 1 1131 0518 1

4 892 1 32 1 1 936 0518 1

4 893 1 32 1 1 767 0568 1

4 894 2 8 1 2 818 0668 2

4 895 2 32 1 2 816 0468 2

4 896 2 16 0 2 858 0618 2

4 897 2 8 1 2 1315 0468 2

4 898 1 32 1 1 836 0618 1

4 899 2 16 0 2 2263 0468 2

4 900 1 8 1 1 1781 0618 1

4 901 2 8 1 2 1069 0618 2

4 902 1 32 1 1 819 0518 1

4 903 2 32 1 2 1042 0568 2

4 904 2 32 1 2 947 0668 2

4 905 1 16 1 1 1165 0568 1

4 906 2 8 1 2 2341 0668 1

4 907 2 16 1 2 1597 0568 2

4 908 1 8 1 1 805 0568 1

4 909 2 16 0 2 1036 0468 2

4 910 1 16 1 1 1011 0518 1

4 911 2 32 0 2 694 0518 2

4 912 2 8 1 2 813 0668 2

4 913 2 32 1 2 727 0618 2

4 914 1 8 1 1 828 0518 2

4 915 1 32 1 1 789 0568 1

4 916 1 32 1 1 776 0568 1

4 917 1 16 1 1 807 0668 1

4 918 1 8 1 1 659 0668 2

4 919 1 8 1 1 668 0668 1

4 920 1 32 1 1 857 0518 1

4 921 1 16 1 1 804 0568 1

4 922 2 16 1 2 969 0618 2

4 923 1 16 1 1 1707 0468 1

4 924 2 8 1 2 1031 0618 2

4 925 2 8 1 2 656 0468 2

4 926 2 16 1 2 699 0668 2

4 927 1 8 1 1 738 0518 2

4 928 1 8 1 1 844 0468 1

4 929 1 8 1 1 712 0618 1

4 930 1 32 0 1 1066 0668 1

4 931 2 32 1 2 1206 0468 2

4 932 2 32 1 2 1278 0518 2

4 933 1 32 1 1 940 0468 1

4 934 2 32 1 2 814 0618 2

4 935 1 16 1 1 954 0568 1

4 936 2 16 1 2 1154 0518 2

4 937 1 16 1 1 661 0668 1

4 938 2 16 0 2 630 0668 2

4 939 2 8 1 2 1286 0518 1

4 940 2 16 1 2 780 0518 2

4 941 1 16 1 1 834 0668 1

4 942 2 16 1 2 991 0568 2

4 943 2 32 1 2 1031 0568 2

4 944 2 16 1 2 719 0668 2

4 945 2 32 1 2 1073 0668 2

4 946 1 8 1 1 850 0568 1

4 947 2 32 1 2 920 0618 2

4 948 1 8 1 1 1227 0468 1

4 949 1 16 1 1 1299 0468 1

4 950 2 8 1 2 834 0518 1

4 951 1 32 1 1 2373 0518 1

4 952 2 8 1 2 1028 0518 2

4 953 2 8 1 2 1403 0468 2

4 954 2 16 1 2 931 0618 2

4 955 2 8 1 2 905 0518 2

4 956 2 32 1 2 873 0668 2

4 957 1 16 1 1 1068 0568 1

4 958 2 32 0 2 927 0468 2

4 959 2 16 1 2 1752 0518 2

4 960 1 32 1 1 1027 0618 1

4 961 1 8 1 1 1541 0568 2

4 962 2 8 1 2 784 0618 2

4 963 2 8 1 2 920 0468 2

4 964 1 8 1 1 670 0518 1

4 965 2 8 1 2 840 0618 2

4 966 2 8 1 2 795 0568 2

4 967 1 16 1 1 862 0668 1

4 968 1 32 1 1 1126 0468 1

4 969 1 16 1 1 748 0668 1

4 970 2 32 1 2 772 0618 2

4 971 1 8 1 1 847 0668 2

4 972 2 32 1 2 829 0668 2

4 973 2 16 1 2 722 0568 2

4 974 2 16 1 2 1030 0518 2

4 975 1 8 1 1 819 0518 2

4 976 1 16 0 1 879 0518 1

4 977 1 32 0 1 724 0618 1

4 978 1 16 1 1 1148 0568 1

4 979 1 8 1 1 716 0668 2

4 980 2 8 1 2 848 0468 2

4 981 1 16 1 1 2496 0668 1

4 982 1 32 1 1 714 0518 1

4 983 2 32 1 2 770 0568 2

4 984 2 8 1 2 730 0568 2

4 985 2 32 1 2 777 0618 2

4 986 2 32 1 2 1064 0568 2

4 987 1 16 0 1 1119 0468 1

4 988 2 16 1 2 1051 0568 2

4 989 1 8 1 1 1064 0568 1

4 990 1 32 1 1 813 0668 1

4 991 1 32 1 1 1002 0668 1

4 992 1 32 1 1 1912 0668 1

4 993 1 16 1 1 1823 0568 1

4 994 1 8 1 1 781 0618 1

4 995 2 16 0 2 745 0518 2

4 996 1 32 1 1 1016 0518 1

4 997 2 8 1 2 937 0468 2

4 998 2 8 1 2 753 0568 2

4 999 2 16 1 2 986 0518 2

4 1000 1 8 1 1 897 0468 1

4 1001 2 8 1 2 745 0668 1

4 1002 2 32 1 2 1084 0668 2

4 1003 1 8 1 1 709 0668 1

4 1004 2 8 1 2 934 0618 2

4 1005 1 32 1 1 793 0568 1

4 1006 2 32 1 2 917 0668 2

4 1007 1 8 1 1 917 0518 2

4 1008 1 8 1 1 838 0668 2

4 1009 2 16 1 2 728 0668 2

4 1010 2 32 1 2 959 0618 2

4 1011 2 32 1 2 878 0468 2

4 1012 1 16 1 1 1015 0568 1

4 1013 2 16 1 2 966 0568 2

4 1014 2 8 1 2 827 0518 2

4 1015 1 16 1 1 793 0468 1

4 1016 1 16 1 1 708 0668 1

4 1017 1 32 1 1 819 0668 1

4 1018 2 16 1 2 982 0568 2

4 1019 2 32 0 2 4252 0568 2

4 1020 1 16 1 1 1243 0468 1

4 1021 2 8 1 2 948 0468 2

4 1022 1 8 1 1 859 0568 1

4 1023 2 32 1 2 770 0468 2

4 1024 2 16 1 2 785 0568 2

4 1025 2 8 1 2 600 0668 2

4 1026 1 8 1 1 803 0568 2

4 1027 1 8 1 1 618 0668 1

4 1028 2 8 1 2 930 0518 2

4 1029 2 8 1 2 926 0518 1

4 1030 2 32 1 2 1520 0468 2

4 1031 1 32 1 1 890 0468 1

4 1032 1 8 1 1 816 0468 1

4 1033 1 16 0 1 761 0518 1

4 1034 1 16 1 1 991 0568 1

4 1035 1 8 1 1 811 0568 2

4 1036 1 16 1 1 677 0518 1

4 1037 1 16 1 1 759 0518 1

4 1038 2 32 0 2 748 0518 2

4 1039 1 16 1 1 1967 0518 1

4 1040 1 32 0 1 508 0468 1

4 1041 1 16 0 1 946 0568 1

4 1042 2 16 0 2 1399 0468 2

4 1043 2 8 1 2 968 0668 2

4 1044 2 16 1 2 1180 0568 2

4 1045 2 16 0 2 1088 0618 2

4 1046 2 16 1 2 825 0618 2

4 1047 1 16 1 1 794 0618 1

4 1048 1 32 1 1 791 0618 1

4 1049 1 8 0 1 866 0618 1

4 1050 2 8 1 2 987 0468 2

4 1051 2 8 1 2 1180 0468 2

4 1052 1 16 1 1 1081 0568 1

4 1053 2 8 0 2 1111 0668 1

4 1054 2 8 1 2 793 0468 2

4 1055 2 8 0 2 1301 0618 2

4 1056 1 32 1 1 853 0518 1

4 1057 2 16 1 2 1040 0618 2

4 1058 2 32 1 2 675 0518 2

4 1059 2 8 1 2 617 0618 2

4 1060 1 32 1 1 1205 0468 1

4 1061 2 8 1 2 752 0518 2

4 1062 1 32 1 1 650 0468 1

4 1063 1 32 1 1 648 0518 1

4 1064 2 8 1 2 1702 0518 1

4 1065 1 32 1 1 674 0568 1

4 1066 2 16 1 2 665 0668 2

4 1067 2 32 1 2 918 0518 2

4 1068 2 8 1 2 697 0568 2

4 1069 2 16 1 2 666 0668 2

4 1070 2 16 1 2 672 0518 2

4 1071 1 8 1 1 919 0518 2

4 1072 2 8 1 2 806 0668 2

4 1073 2 16 1 2 1413 0518 2

4 1074 2 32 1 2 1034 0668 2

4 1075 2 32 1 2 938 0668 2

4 1076 1 32 1 1 1201 0518 1

4 1077 1 8 1 1 1036 0468 1

4 1078 2 32 1 2 1076 0668 2

4 1079 2 8 1 2 1914 0618 2

4 1080 2 8 1 2 678 0518 1

4 1081 1 32 1 1 731 0568 1

4 1082 1 8 0 1 1268 0618 1

4 1083 1 8 1 1 1063 0518 1

4 1084 2 32 0 2 858 0468 2

4 1085 1 8 1 1 1003 0668 2

4 1086 1 8 1 1 761 0518 1

4 1087 2 8 1 2 751 0568 2

4 1088 1 16 1 1 1001 0618 1

4 1089 2 16 0 2 1673 0618 2

4 1090 1 16 1 1 936 0668 1

4 1091 2 8 1 2 861 0618 2

4 1092 1 8 1 1 867 0568 1

4 1093 2 32 1 2 1236 0668 2

4 1094 1 8 1 1 725 0518 1

4 1095 1 32 1 1 875 0618 1

4 1096 2 16 1 2 911 0668 2

4 1097 2 8 1 2 720 0668 2

4 1098 2 32 1 2 931 0568 2

4 1099 1 16 1 1 1055 0618 1

4 1100 2 8 1 2 697 0668 2

4 1101 1 16 1 1 1130 0518 1

4 1102 1 32 1 1 732 0618 1

4 1103 1 16 1 1 790 0668 1

4 1104 2 16 1 2 847 0618 2

4 1105 1 8 1 1 983 0468 1

4 1106 2 16 0 2 850 0618 2

4 1107 2 16 1 2 751 0668 2

4 1108 1 8 1 1 834 0468 1

4 1109 2 32 1 2 1047 0468 2

4 1110 2 32 1 2 1136 0568 2

4 1111 2 8 1 2 1098 0568 2

4 1112 1 16 1 1 916 0618 1

4 1113 1 16 0 1 850 0618 1

4 1114 1 32 1 1 1556 0468 1

4 1115 1 8 1 1 871 0468 1

4 1116 1 16 1 1 751 0468 1

4 1117 1 16 1 1 871 0518 1

4 1118 2 16 0 2 866 0618 2

4 1119 1 8 1 1 882 0618 1

4 1120 2 16 0 2 1042 0468 2

4 1121 1 32 1 1 1280 0518 1

4 1122 1 32 1 1 655 0518 1

4 1123 2 32 1 2 1251 0568 2

4 1124 2 8 1 2 943 0618 2

4 1125 1 32 1 1 766 0668 1

4 1126 1 32 1 1 926 0568 1

4 1127 2 32 1 2 845 0618 2

4 1128 1 16 1 1 1917 0618 1

4 1129 2 32 1 2 1041 0618 2

4 1130 2 16 0 2 2001 0468 2

4 1131 2 32 1 2 932 0468 2

4 1132 2 32 1 2 1441 0518 2

4 1133 2 32 1 2 1056 0468 2

4 1134 2 16 1 2 850 0568 2

4 1135 1 16 0 1 865 0468 1

4 1136 2 16 1 2 918 0668 2

4 1137 1 32 0 1 1163 0568 1

4 1138 2 16 1 2 1568 0618 2

4 1139 1 8 1 1 821 0618 1

4 1140 1 16 1 1 884 0668 1

4 1141 1 16 1 1 927 0618 1

4 1142 1 32 1 1 1802 0568 1

4 1143 2 16 1 2 1323 0518 2

4 1144 1 32 1 1 997 0618 1

4 1145 1 32 1 1 2758 0518 1

4 1146 1 16 1 1 909 0518 1

4 1147 2 16 0 2 1952 0468 2

4 1148 1 32 1 1 1240 0618 1

4 1149 1 16 1 1 1870 0668 1

4 1150 2 32 1 2 2422 0618 2

4 1151 2 16 1 2 1115 0668 2

4 1152 1 32 1 1 817 0618 1

4 1153 1 8 1 1 931 0518 2

4 1154 1 16 1 1 949 0618 1

4 1155 2 32 1 2 949 0518 2

4 1156 2 32 1 2 738 0468 2

4 1157 1 8 1 1 769 0618 1

4 1158 2 32 1 2 1226 0518 2

4 1159 2 32 1 2 858 0618 2

4 1160 2 16 1 2 950 0668 2

4 1161 1 8 1 1 818 0668 1

4 1162 1 16 0 1 817 0568 1

4 1163 2 16 0 2 1396 0468 2

4 1164 2 32 1 2 1118 0518 2

4 1165 2 16 1 2 5043 0468 2

4 1166 1 32 1 1 1035 0568 1

4 1167 1 16 0 1 2343 0468 1

4 1168 1 16 1 1 960 0568 1

4 1169 1 32 1 1 709 0668 1

4 1170 1 8 1 1 782 0468 1

4 1171 2 16 0 2 935 0518 2

4 1172 1 8 1 1 1019 0618 1

4 1173 2 16 0 2 2752 0568 2

4 1174 2 16 0 2 3449 0468 2

4 1175 2 32 1 2 1361 0518 2

4 1176 1 32 1 1 1098 0468 1

4 1177 1 32 1 1 903 0468 1

4 1178 2 32 1 2 1007 0568 2

4 1179 1 8 0 1 1129 0618 1

4 1180 1 8 1 1 1223 0468 1

4 1181 2 16 1 2 1157 0468 2

4 1182 2 32 1 2 847 0618 2

4 1183 1 16 1 1 904 0468 1

4 1184 1 16 1 1 2407 0468 1

4 1185 1 32 1 1 813 0668 1

4 1186 1 8 1 1 1461 0568 1

4 1187 2 8 0 2 3419 0518 1

4 1188 2 8 0 2 909 0568 1

4 1189 2 32 1 2 1078 0668 2

4 1190 2 8 1 2 1091 0468 2

4 1191 2 32 1 2 775 0568 2

4 1192 1 8 1 1 822 0568 2

4 1193 2 8 1 2 775 0568 1

4 1194 1 32 1 1 1146 0668 1

4 1195 1 32 1 1 701 0618 1

4 1196 2 16 1 2 995 0518 2

4 1197 1 32 1 1 754 0568 1

4 1198 2 8 1 2 680 0668 2

4 1199 1 8 1 1 769 0668 1

4 1200 2 8 1 2 1075 0518 2

5 1 2 8 1 2 735 0668 2

5 2 1 8 1 1 684 0668 1

5 3 2 32 1 2 1313 0518 2

5 4 2 32 0 2 914 0518 2

5 5 2 32 1 2 1652 0468 2

5 6 1 8 1 1 825 0668 2

5 7 2 16 0 2 2583 0518 2

5 8 1 8 0 1 1484 0468 1

5 9 1 32 1 1 1555 0468 1

5 10 1 32 1 1 940 0518 1

5 11 1 16 1 1 887 0668 1

5 12 1 8 1 1 661 0668 2

5 13 2 16 1 2 1491 0618 2

5 14 1 8 1 1 1477 0468 1

5 15 2 32 1 2 1169 0468 2

5 16 2 16 1 2 815 0668 2

5 17 1 8 1 1 889 0568 2

5 18 1 32 1 1 1052 0668 1

5 19 1 8 1 1 1950 0618 1

5 20 2 8 0 2 1083 0518 1

5 21 2 16 1 2 1301 0618 2

5 22 2 8 1 2 1129 0518 1

5 23 1 32 1 1 983 0618 1

5 24 2 8 1 2 984 0568 2

5 25 2 8 1 2 1080 0618 2

5 26 1 8 1 1 916 0618 1

5 27 2 32 1 2 1526 0468 2

5 28 1 32 1 1 1160 0618 1

5 29 1 8 1 1 956 0618 1

5 30 2 8 1 2 1313 0468 2

5 31 2 8 1 2 1343 0618 2

5 32 2 8 1 2 1033 0668 2

5 33 1 32 1 1 2300 0468 1

5 34 2 32 1 2 1214 0468 2

5 35 2 16 1 2 1418 0568 2

5 36 2 8 1 2 726 0568 1

5 37 1 32 1 1 1022 0618 1

5 38 2 8 1 2 763 0568 2

5 39 2 8 1 2 709 0568 2

5 40 2 16 0 2 963 0518 2

5 41 1 32 0 1 729 0618 1

5 42 2 16 0 2 1479 0468 2

5 43 2 32 1 2 1326 0568 2

5 44 2 16 1 2 1216 0668 2

5 45 1 32 1 1 819 0668 1

5 46 1 32 1 1 852 0618 1

5 47 2 16 1 2 1283 0568 2

5 48 1 8 1 1 789 0518 2

5 49 1 8 1 1 901 0468 1

5 50 1 16 1 1 1096 0518 1

5 51 2 32 1 2 1007 0668 2

5 52 1 16 1 1 1033 0468 1

5 53 2 32 1 2 824 0668 2

5 54 1 8 0 1 1379 0568 1

5 55 1 16 1 1 2015 0618 1

5 56 1 32 1 1 940 0568 1

5 57 1 8 0 1 1751 0468 1

5 58 2 32 1 2 858 0568 2

5 59 2 32 1 2 734 0618 2

5 60 1 8 1 1 991 0618 1

5 61 1 8 1 1 874 0568 2

5 62 1 32 1 1 1028 0568 1

5 63 2 8 1 2 869 0668 1

5 64 2 8 0 2 1355 0618 2

5 65 1 16 1 1 1208 0668 1

5 66 2 16 1 2 1172 0618 2

5 67 2 8 1 2 1224 0618 2

5 68 1 32 1 1 885 0668 1

5 69 2 16 1 2 914 0568 2

5 70 2 32 1 2 1024 0468 2

5 71 2 32 0 2 1025 0518 2

5 72 2 32 1 2 907 0568 2

5 73 2 32 1 2 1031 0518 2

5 74 1 8 1 1 917 0568 2

5 75 1 32 1 1 856 0518 1

5 76 1 8 1 1 836 0518 1

5 77 1 8 0 1 800 0468 1

5 78 1 32 1 1 948 0668 1

5 79 2 32 1 2 866 0568 2

5 80 2 8 1 2 917 0518 2

5 81 1 32 1 1 1733 0468 1

5 82 1 32 1 1 1155 0468 1

5 83 1 8 1 1 883 0668 1

5 84 1 16 1 1 1084 0618 1

5 85 2 32 1 2 888 0668 2

5 86 1 16 0 1 1102 0468 1

5 87 2 16 1 2 1381 0618 2

5 88 1 16 1 1 1156 0468 1

5 89 1 32 1 1 1104 0568 1

5 90 1 16 1 1 733 0668 1

5 91 2 32 1 2 738 0618 2

5 92 2 8 1 2 1305 0468 2

5 93 2 32 1 2 963 0468 2

5 94 1 8 1 1 720 0568 2

5 95 1 8 1 1 776 0518 1

5 96 1 16 1 1 1248 0468 1

5 97 2 8 1 2 738 0568 2

5 98 2 32 1 2 1096 0668 2

5 99 1 16 1 1 876 0518 1

5 100 1 8 1 1 684 0568 1

5 101 1 8 1 1 571 0668 1

5 102 1 16 0 1 1082 0568 1

5 103 2 16 1 2 1040 0568 2

5 104 1 32 1 1 1029 0518 1

5 105 1 8 0 1 1082 0518 1

5 106 1 8 1 1 1026 0468 1

5 107 2 32 1 2 869 0618 2

5 108 2 32 1 2 832 0668 2

5 109 2 8 1 2 874 0568 2

5 110 1 32 1 1 1135 0568 1

5 111 2 16 1 2 1321 0568 2

5 112 1 16 1 1 1639 0568 1

5 113 2 32 1 2 1258 0468 2

5 114 1 32 1 1 786 0618 1

5 115 1 32 0 1 1277 0568 1

5 116 2 16 1 2 1285 0468 2

5 117 1 16 1 1 2363 0518 1

5 118 1 16 1 1 952 0518 1

5 119 1 8 1 1 946 0518 2

5 120 1 32 1 1 1106 0618 1

5 121 1 32 0 1 1021 0518 1

5 122 1 8 1 1 970 0668 1

5 123 1 16 1 1 732 0668 1

5 124 2 16 1 2 809 0568 2

5 125 1 16 1 1 958 0468 1

5 126 1 32 1 1 1344 0468 1

5 127 2 8 1 2 960 0518 1

5 128 1 16 1 1 1226 0568 1

5 129 1 8 1 1 760 0468 1

5 130 2 32 1 2 949 0618 2

5 131 1 16 1 1 1606 0468 1

5 132 1 32 1 1 914 0668 1

5 133 1 8 1 1 919 0668 2

5 134 2 32 1 2 1124 0518 2

5 135 1 32 1 1 1178 0518 1

5 136 1 8 1 1 680 0668 2

5 137 2 16 1 2 1043 0568 2

5 138 1 32 1 1 768 0518 1

5 139 2 32 1 2 1195 0668 2

5 140 2 32 1 2 1003 0518 2

5 141 1 8 1 1 920 0618 1

5 142 1 16 1 1 1079 0568 1

5 143 1 32 1 1 865 0668 1

5 144 2 8 1 2 772 0668 2

5 145 2 8 1 2 664 0668 2

5 146 1 8 1 1 658 0568 1

5 147 2 16 1 2 886 0468 2

5 148 2 8 1 2 819 0668 2

5 149 2 16 1 2 1010 0468 2

5 150 1 32 1 1 1097 0468 1

5 151 2 16 1 2 1487 0468 2

5 152 2 16 1 2 920 0468 2

5 153 1 8 1 1 1174 0618 1

5 154 1 16 1 1 784 0668 1

5 155 2 16 1 2 1447 0668 2

5 156 2 8 1 2 825 0568 1

5 157 1 32 0 1 790 0518 1

5 158 2 16 1 2 1089 0668 2

5 159 2 8 1 2 1080 0468 2

5 160 1 32 1 1 870 0468 1

5 161 1 8 0 1 1842 0468 1

5 162 1 16 1 1 1318 0618 1

5 163 2 16 1 2 1118 0468 2

5 164 2 32 1 2 942 0618 2

5 165 2 16 0 2 1396 0518 2

5 166 2 16 1 2 1031 0518 2

5 167 2 16 1 2 994 0518 2

5 168 2 16 1 2 1385 0468 2

5 169 2 16 1 2 1156 0618 2

5 170 1 16 1 1 980 0668 1

5 171 2 16 1 2 1254 0518 2

5 172 2 8 1 2 768 0468 2

5 173 1 16 1 1 1183 0618 1

5 174 1 16 1 1 948 0518 1

5 175 2 16 1 2 748 0668 2

5 176 1 16 1 1 1424 0618 1

5 177 2 16 1 2 1106 0518 2

5 178 1 32 1 1 762 0568 1

5 179 1 32 1 1 744 0668 1

5 180 2 16 1 2 795 0668 2

5 181 1 16 1 1 909 0518 1

5 182 2 8 1 2 996 0618 2

5 183 1 32 1 1 720 0618 1

5 184 1 8 0 1 829 0618 1

5 185 2 32 1 2 939 0568 2

5 186 1 16 1 1 737 0568 1

5 187 1 16 1 1 1154 0568 1

5 188 2 16 1 2 1064 0618 2

5 189 1 16 0 1 1037 0568 1

5 190 2 8 1 2 1119 0468 2

5 191 1 16 1 1 800 0618 1

5 192 2 8 1 2 1003 0568 2

5 193 1 32 1 1 966 0568 1

5 194 1 16 1 1 1069 0518 1

5 195 1 32 1 1 759 0568 1

5 196 2 32 0 2 623 0568 2

5 197 1 16 1 1 945 0468 1

5 198 1 16 0 1 926 0568 1

5 199 1 16 1 1 1071 0668 1

5 200 2 32 1 2 897 0668 2

5 201 1 16 1 1 1175 0618 1

5 202 2 32 1 2 913 0468 2

5 203 2 8 1 2 841 0618 2

5 204 1 8 1 1 891 0518 1

5 205 2 16 1 2 787 0568 2

5 206 2 32 1 2 905 0568 2

5 207 1 8 1 1 755 0518 2

5 208 2 8 1 2 1005 0468 2

5 209 1 32 1 1 822 0468 1

5 210 1 16 1 1 944 0468 1

5 211 1 16 1 1 672 0668 1

5 212 2 32 1 2 838 0618 2

5 213 2 16 1 2 1431 0668 2

5 214 1 32 1 1 923 0668 1

5 215 1 16 1 1 1167 0618 1

5 216 2 32 1 2 1161 0518 2

5 217 2 32 1 2 1009 0668 2

5 218 2 32 1 2 687 0568 2

5 219 2 8 1 2 1240 0668 2

5 220 2 8 1 2 839 0518 2

5 221 2 16 1 2 2268 0618 2

5 222 2 8 1 2 767 0518 2

5 223 2 8 1 2 1212 0468 2

5 224 2 16 1 2 1173 0618 2

5 225 2 32 1 2 1294 0518 2

5 226 1 8 1 1 854 0618 1

5 227 1 16 1 1 826 0518 1

5 228 1 8 1 1 707 0568 1

5 229 2 8 1 2 1033 0618 2

5 230 2 8 1 2 763 0668 1

5 231 2 8 1 2 757 0618 2

5 232 1 8 1 1 810 0518 2

5 233 2 32 1 2 839 0618 2

5 234 2 16 1 2 787 0668 2

5 235 2 8 1 2 1312 0468 2

5 236 2 8 0 2 888 0518 2

5 237 2 32 1 2 864 0618 2

5 238 2 8 1 2 776 0518 1

5 239 1 32 1 1 738 0518 1

5 240 2 16 1 2 1078 0518 2

5 241 1 32 1 1 868 0668 1

5 242 1 32 1 1 1107 0468 1

5 243 2 32 1 2 1215 0518 2

5 244 2 32 1 2 802 0518 2

5 245 1 32 1 1 1093 0468 1

5 246 1 16 0 1 894 0518 1

5 247 1 16 1 1 1066 0518 1

5 248 1 32 1 1 707 0618 1

5 249 2 32 1 2 883 0518 2

5 250 2 32 1 2 903 0568 2

5 251 2 32 1 2 719 0518 2

5 252 1 8 1 1 1012 0468 1

5 253 2 32 1 2 911 0668 2

5 254 2 32 0 2 1004 0668 2

5 255 2 32 1 2 927 0668 2

5 256 2 16 1 2 979 0668 2

5 257 2 16 1 2 803 0568 2

5 258 2 16 1 2 831 0618 2

5 259 2 16 0 2 763 0568 2

5 260 2 16 1 2 1250 0468 2

5 261 2 8 1 2 741 0568 2

5 262 2 16 1 2 838 0568 2

5 263 2 16 0 2 944 0618 2

5 264 1 16 1 1 653 0568 1

5 265 2 16 1 2 1366 0568 2

5 266 1 16 1 1 907 0618 1

5 267 2 16 0 2 1579 0468 2

5 268 1 8 0 1 724 0568 2

5 269 2 32 1 2 1041 0468 2

5 270 1 32 1 1 772 0518 1

5 271 2 32 1 2 811 0568 2

5 272 1 32 1 1 929 0668 1

5 273 2 32 1 2 891 0568 2

5 274 1 8 1 1 655 0668 1

5 275 2 8 1 2 799 0568 2

5 276 1 8 1 1 963 0518 1

5 277 1 16 1 1 1158 0518 1

5 278 1 32 1 1 878 0568 1

5 279 2 8 1 2 1263 0518 2

5 280 2 8 1 2 806 0668 2

5 281 1 32 1 1 742 0568 1

5 282 2 8 1 2 828 0668 2

5 283 1 8 1 1 710 0468 1

5 284 1 32 1 1 674 0618 1

5 285 2 32 1 2 948 0668 2

5 286 1 8 1 1 797 0668 2

5 287 2 16 1 2 950 0518 2

5 288 2 8 1 2 695 0668 2

5 289 2 32 1 2 884 0618 2

5 290 2 8 1 2 539 0468 2

5 291 1 32 1 1 733 0518 1

5 292 1 8 1 1 796 0568 2

5 293 1 16 1 1 735 0668 1

5 294 1 8 1 1 851 0618 1

5 295 2 16 0 2 622 0518 2

5 296 1 32 1 1 704 0468 1

5 297 1 8 1 1 834 0668 1

5 298 2 32 1 2 852 0518 2

5 299 2 8 1 2 755 0468 2

5 300 1 16 1 1 829 0568 1

5 301 2 16 1 2 823 0568 2

5 302 2 8 1 2 687 0518 1

5 303 1 16 1 1 1360 0568 1

5 304 1 32 1 1 913 0668 1

5 305 1 16 1 1 900 0568 1

5 306 1 8 1 1 789 0518 2

5 307 2 32 0 2 1118 0618 2

5 308 2 16 1 2 922 0618 2

5 309 1 8 1 1 820 0568 1

5 310 1 16 1 1 756 0568 1

5 311 1 32 1 1 960 0518 1

5 312 1 8 1 1 985 0468 1

5 313 2 8 1 2 831 0668 2

5 314 1 16 1 1 1397 0618 1

5 315 2 16 1 2 1013 0618 2

5 316 1 32 1 1 714 0568 1

5 317 1 32 1 1 729 0518 1

5 318 2 8 1 2 844 0568 1

5 319 1 16 1 1 740 0668 1

5 320 2 16 1 2 1338 0468 2

5 321 1 8 1 1 537 0668 2

5 322 2 8 1 2 698 0518 2

5 323 1 16 1 1 995 0568 1

5 324 2 8 1 2 732 0618 2

5 325 1 8 1 1 832 0468 1

5 326 2 8 0 2 719 0468 2

5 327 2 16 1 2 1045 0468 2

5 328 2 32 0 2 1311 0468 2

5 329 1 16 1 1 1429 0468 1

5 330 2 8 1 2 748 0568 2

5 331 2 8 1 2 1537 0468 2

5 332 1 16 1 1 1081 0468 1

5 333 1 16 1 1 859 0618 1

5 334 1 32 1 1 778 0668 1

5 335 1 16 1 1 843 0618 1

5 336 2 32 1 2 767 0618 2

5 337 1 8 1 1 774 0468 1

5 338 2 16 1 2 1216 0518 2

5 339 1 16 1 1 756 0618 1

5 340 2 8 1 2 669 0618 2

5 341 1 16 1 1 721 0668 1

5 342 1 32 1 1 654 0568 1

5 343 1 8 1 1 691 0618 1

5 344 2 16 1 2 973 0518 2

5 345 1 32 1 1 979 0668 1

5 346 2 32 1 2 922 0518 2

5 347 1 8 1 1 619 0668 2

5 348 1 16 0 1 733 0618 1

5 349 2 16 1 2 1081 0518 2

5 350 1 8 1 1 731 0618 1

5 351 2 8 1 2 816 0518 2

5 352 1 32 1 1 752 0618 1

5 353 1 32 1 1 725 0618 1

5 354 1 16 0 1 953 0568 1

5 355 1 8 0 1 2428 0468 1

5 356 2 32 1 2 827 0618 2

5 357 2 8 1 2 1039 0518 1

5 358 1 32 1 1 1118 0468 1

5 359 1 8 1 1 728 0568 2

5 360 1 32 1 1 719 0618 1

5 361 2 32 1 2 829 0568 2

5 362 1 32 1 1 1108 0618 1

5 363 1 8 1 1 1338 0468 1

5 364 2 16 1 2 880 0568 2

5 365 2 16 1 2 2132 0468 2

5 366 2 16 1 2 835 0618 2

5 367 1 8 1 1 732 0668 2

5 368 1 32 0 1 745 0468 1

5 369 1 16 1 1 1024 0468 1

5 370 2 16 1 2 1059 0518 2

5 371 1 16 1 1 821 0618 1

5 372 2 16 1 2 826 0468 2

5 373 2 16 1 2 1352 0618 2

5 374 2 32 1 2 965 0468 2

5 375 2 8 1 2 768 0618 2

5 376 2 16 1 2 881 0668 2

5 377 1 32 1 1 772 0568 1

5 378 1 8 1 1 1003 0518 2

5 379 2 8 1 2 774 0568 1

5 380 2 32 1 2 1461 0468 2

5 381 1 8 1 1 726 0568 1

5 382 2 32 1 2 873 0668 2

5 383 1 32 1 1 741 0518 1

5 384 2 16 1 2 1305 0668 2

5 385 2 16 1 2 1028 0468 2

5 386 2 8 1 2 1513 0518 2

5 387 1 32 1 1 952 0568 1

5 388 1 16 1 1 890 0468 1

5 389 1 32 1 1 744 0668 1

5 390 1 16 1 1 734 0668 1

5 391 1 16 1 1 651 0668 1

5 392 2 32 1 2 1310 0618 2

5 393 2 8 1 2 906 0618 2

5 394 2 16 1 2 1448 0468 2

5 395 2 8 1 2 783 0618 2

5 396 2 32 0 2 1027 0518 2

5 397 2 8 1 2 867 0668 2

5 398 1 32 1 1 1399 0518 1

5 399 2 8 1 2 800 0468 2

5 400 1 8 1 1 834 0518 1

5 401 1 8 1 1 1103 0468 1

5 402 1 16 1 1 746 0668 1

5 403 1 8 1 1 840 0568 2

5 404 1 8 1 1 601 0668 1

5 405 2 32 1 2 2406 0468 2

5 406 1 8 1 1 810 0518 1

5 407 1 8 1 1 567 0668 1

5 408 1 8 1 1 1018 0618 1

5 409 1 32 1 1 810 0668 1

5 410 2 32 1 2 999 0568 2

5 411 2 16 1 2 772 0568 2

5 412 2 8 1 2 562 0568 2

5 413 1 16 0 1 1023 0468 1

5 414 2 8 1 2 675 0568 2

5 415 2 16 1 2 762 0518 2

5 416 2 32 1 2 801 0668 2

5 417 2 16 0 2 853 0568 2

5 418 1 16 1 1 980 0618 1

5 419 1 8 1 1 1054 0618 1

5 420 1 16 1 1 1035 0468 1

5 421 1 32 1 1 643 0468 1

5 422 2 8 1 2 838 0668 1

5 423 2 32 1 2 679 0468 2

5 424 2 32 1 2 653 0618 2

5 425 2 32 0 2 694 0568 2

5 426 1 16 1 1 732 0518 1

5 427 1 8 1 1 844 0518 2

5 428 2 32 1 2 739 0618 2

5 429 2 8 1 2 748 0568 2

5 430 2 8 1 2 590 0468 2

5 431 2 32 1 2 825 0568 2

5 432 1 8 1 1 815 0618 1

5 433 2 8 1 2 806 0668 1

5 434 2 8 1 2 645 0518 1

5 435 1 16 1 1 799 0518 1

5 436 1 16 1 1 644 0668 1

5 437 2 32 1 2 1142 0468 2

5 438 1 8 1 1 804 0568 1

5 439 2 16 1 2 930 0668 2

5 440 1 8 1 1 782 0618 1

5 441 1 8 1 1 756 0518 2

5 442 2 32 1 2 1044 0618 2

5 443 1 32 1 1 794 0668 1

5 444 1 8 1 1 1057 0618 1

5 445 1 32 1 1 698 0568 1

5 446 1 16 1 1 829 0568 1

5 447 2 32 1 2 851 0568 2

5 448 1 32 1 1 711 0468 1

5 449 2 16 1 2 1158 0518 2

5 450 1 16 1 1 807 0518 1

5 451 1 16 1 1 1027 0518 1

5 452 1 32 1 1 798 0518 1

5 453 2 16 1 2 644 0668 2

5 454 1 8 0 1 1104 0518 1

5 455 1 16 1 1 910 0518 1

5 456 2 8 1 2 766 0618 2

5 457 2 16 1 2 1281 0618 2

5 458 2 8 1 2 787 0518 1

5 459 1 8 1 1 690 0568 1

5 460 2 16 1 2 1345 0618 2

5 461 1 32 1 1 735 0618 1

5 462 2 32 1 2 1051 0668 2

5 463 2 16 1 2 1422 0668 2

5 464 1 32 1 1 1105 0568 1

5 465 1 16 1 1 1391 0468 1

5 466 2 8 1 2 720 0618 2

5 467 2 8 1 2 704 0618 2

5 468 2 32 1 2 737 0518 2

5 469 2 16 1 2 722 0668 2

5 470 2 8 1 2 1038 0468 2

5 471 2 8 1 2 767 0468 2

5 472 2 32 1 2 1576 0468 2

5 473 2 8 1 2 719 0668 2

5 474 1 16 0 1 1016 0468 1

5 475 1 32 1 1 868 0468 1

5 476 1 16 1 1 623 0668 1

5 477 2 32 1 2 944 0668 2

5 478 1 32 1 1 910 0518 1

5 479 1 32 1 1 811 0618 1

5 480 2 16 1 2 741 0668 2

5 481 1 8 0 1 683 0618 1

5 482 2 32 1 2 825 0468 2

5 483 2 32 1 2 1319 0468 2

5 484 1 32 1 1 613 0618 1

5 485 2 8 1 2 706 0668 2

5 486 2 16 1 2 1010 0568 2

5 487 1 8 1 1 685 0618 1

5 488 1 32 1 1 817 0468 1

5 489 2 16 1 2 627 0668 2

5 490 2 8 1 2 792 0668 2

5 491 1 16 1 1 876 0468 1

5 492 2 8 1 2 775 0468 2

5 493 1 32 1 1 648 0568 1

5 494 2 16 1 2 1123 0468 2

5 495 1 32 1 1 788 0668 1

5 496 1 32 1 1 707 0468 1

5 497 2 8 1 2 1139 0568 1

5 498 1 32 1 1 803 0518 1

5 499 1 8 0 1 743 0618 1

5 500 2 8 1 2 1016 0518 2

5 501 1 16 1 1 1160 0518 1

5 502 1 16 1 1 883 0618 1

5 503 2 16 1 2 760 0568 2

5 504 1 32 1 1 676 0568 1

5 505 1 8 1 1 775 0468 1

5 506 1 32 1 1 681 0518 1

5 507 2 16 1 2 706 0668 2

5 508 1 8 1 1 825 0468 1

5 509 2 32 1 2 778 0618 2

5 510 2 8 1 2 795 0518 2

5 511 1 32 1 1 1291 0668 1

5 512 2 8 1 2 902 0518 1

5 513 1 16 1 1 1196 0668 1

5 514 2 8 1 2 656 0518 1

5 515 1 16 1 1 746 0518 1

5 516 2 16 1 2 864 0468 2

5 517 2 8 1 2 733 0668 2

5 518 1 8 1 1 928 0668 1

5 519 1 8 0 1 746 0618 1

5 520 1 32 1 1 717 0618 1

5 521 2 32 1 2 865 0668 2

5 522 2 32 0 2 689 0518 2

5 523 1 8 1 1 881 0568 2

5 524 2 32 1 2 782 0518 2

5 525 2 32 1 2 734 0518 2

5 526 2 8 1 2 1183 0468 2

5 527 2 8 0 2 713 0468 2

5 528 2 8 1 2 749 0468 2

5 529 1 8 0 1 1366 0468 1

5 530 2 8 1 2 888 0618 2

5 531 2 32 1 2 938 0468 2

5 532 2 8 1 2 841 0518 2

5 533 1 8 1 1 642 0668 2

5 534 2 16 1 2 911 0668 2

5 535 1 8 1 1 1188 0568 2

5 536 2 32 1 2 912 0668 2

5 537 1 16 1 1 763 0668 1

5 538 1 32 1 1 1221 0518 1

5 539 2 32 1 2 782 0568 2

5 540 1 16 1 1 1083 0568 1

5 541 1 16 1 1 767 0618 1

5 542 2 8 1 2 1107 0568 2

5 543 2 16 1 2 791 0668 2

5 544 2 16 0 2 897 0618 2

5 545 1 32 1 1 776 0668 1

5 546 1 8 0 1 893 0468 1

5 547 1 8 1 1 771 0518 1

5 548 1 16 1 1 751 0518 1

5 549 2 16 1 2 1494 0468 2

5 550 2 8 1 2 759 0668 2

5 551 1 8 1 1 905 0568 1

5 552 1 16 1 1 2019 0468 1

5 553 2 16 0 2 1107 0518 2

5 554 1 32 1 1 899 0518 1

5 555 1 8 1 1 792 0518 2

5 556 1 32 1 1 883 0668 1

5 557 2 32 0 2 1262 0468 2

5 558 1 8 1 1 815 0568 1

5 559 2 32 1 2 772 0618 2

5 560 2 32 1 2 786 0568 2

5 561 2 8 1 2 639 0568 2

5 562 1 32 0 1 1191 0468 1

5 563 2 8 1 2 697 0568 2

5 564 2 16 1 2 802 0568 2

5 565 2 16 1 2 1067 0518 2

5 566 1 32 1 1 803 0618 1

5 567 1 8 1 1 879 0518 1

5 568 2 8 1 2 642 0618 2

5 569 2 32 1 2 820 0468 2

5 570 2 16 1 2 710 0568 2

5 571 2 8 1 2 754 0518 2

5 572 1 32 1 1 836 0568 1

5 573 1 32 1 1 729 0618 1

5 574 2 8 1 2 688 0568 1

5 575 1 8 1 1 672 0568 2

5 576 2 16 1 2 701 0618 2

5 577 2 8 1 2 764 0618 2

5 578 1 16 1 1 826 0568 1

5 579 2 16 1 2 947 0468 2

5 580 1 16 1 1 845 0618 1

5 581 2 8 1 2 859 0468 2

5 582 2 8 1 2 920 0668 1

5 583 2 32 1 2 2186 0468 2

5 584 2 16 1 2 1022 0568 2

5 585 1 16 1 1 646 0668 1

5 586 1 32 1 1 639 0618 1

5 587 1 32 1 1 663 0618 1

5 588 2 32 1 2 725 0618 2

5 589 1 16 0 1 1529 0468 1

5 590 1 32 1 1 830 0518 1

5 591 2 8 1 2 1095 0468 2

5 592 2 16 1 2 1805 0468 2

5 593 2 16 0 2 869 0668 2

5 594 1 8 1 1 943 0518 2

5 595 1 32 1 1 847 0668 1

5 596 1 16 1 1 655 0568 1

5 597 2 32 1 2 736 0568 2

5 598 1 16 1 1 823 0618 1

5 599 2 32 1 2 704 0618 2

5 600 1 32 1 1 1444 0468 1

5 601 2 16 0 2 832 0668 2

5 602 1 16 1 1 901 0618 1

5 603 1 8 1 1 1171 0468 1

5 604 1 16 1 1 784 0518 1

5 605 1 16 1 1 1005 0518 1

5 606 1 8 1 1 906 0518 2

5 607 1 16 1 1 309 0668 1

5 608 1 8 1 1 591 0668 1

5 609 1 8 1 1 491 0668 2

5 610 1 32 1 1 1276 0468 1

5 611 2 16 0 2 779 0518 2

5 612 1 16 1 1 933 0618 1

5 613 1 16 0 1 707 0618 1

5 614 1 8 1 1 746 0668 2

5 615 2 16 1 2 1127 0518 2

5 616 2 16 1 2 733 0618 2

5 617 1 16 1 1 723 0618 1

5 618 2 32 1 2 952 0668 2

5 619 1 8 1 1 827 0468 1

5 620 2 16 1 2 1479 0468 2

5 621 2 8 1 2 856 0468 2

5 622 1 32 1 1 792 0518 1

5 623 1 16 1 1 1218 0568 1

5 624 2 32 1 2 1342 0518 2

5 625 1 16 1 1 701 0668 1

5 626 1 32 1 1 695 0568 1

5 627 2 32 1 2 823 0568 2

5 628 1 8 0 1 881 0518 2

5 629 1 8 1 1 778 0668 2

5 630 2 16 1 2 807 0518 2

5 631 2 8 1 2 693 0618 2

5 632 2 32 1 2 777 0568 2

5 633 1 8 1 1 738 0468 1

5 634 1 8 1 1 827 0568 2

5 635 1 8 1 1 871 0618 1

5 636 2 16 1 2 698 0668 2

5 637 2 16 1 2 796 0618 2

5 638 2 16 1 2 774 0618 2

5 639 2 8 1 2 765 0468 2

5 640 2 32 1 2 1387 0468 2

5 641 2 16 1 2 859 0618 2

5 642 1 16 1 1 1000 0468 1

5 643 2 32 1 2 835 0618 2

5 644 2 16 1 2 1025 0518 2

5 645 2 8 1 2 647 0668 1

5 646 2 32 1 2 853 0668 2

5 647 1 32 1 1 802 0568 1

5 648 2 32 1 2 1334 0518 2

5 649 2 32 1 2 754 0518 2

5 650 2 16 0 2 945 0518 2

5 651 1 16 0 1 683 0468 1

5 652 2 8 0 2 1476 0618 2

5 653 1 32 1 1 958 0568 1

5 654 2 16 1 2 639 0668 2

5 655 2 32 1 2 801 0668 2

5 656 2 32 1 2 780 0568 2

5 657 2 8 1 2 708 0668 2

5 658 1 32 1 1 843 0518 1

5 659 2 32 1 2 890 0668 2

5 660 1 32 1 1 1165 0618 1

5 661 2 16 1 2 800 0468 2

5 662 2 32 1 2 795 0618 2

5 663 1 16 1 1 791 0568 1

5 664 2 16 1 2 994 0568 2

5 665 2 8 1 2 927 0518 1

5 666 1 32 1 1 664 0568 1

5 667 2 16 1 2 929 0618 2

5 668 1 16 1 1 608 0668 1

5 669 1 32 1 1 1387 0668 1

5 670 1 16 0 1 755 0568 1

5 671 1 32 1 1 911 0518 1

5 672 1 16 1 1 640 0668 1

5 673 1 32 1 1 1025 0468 1

5 674 2 16 0 2 799 0618 2

5 675 1 8 1 1 1075 0468 1

5 676 2 16 1 2 718 0468 2

5 677 2 8 0 2 768 0618 2

5 678 1 32 1 1 982 0668 1

5 679 1 32 1 1 626 0618 1

5 680 2 8 1 2 602 0568 2

5 681 1 32 1 1 1171 0468 1

5 682 1 16 1 1 913 0518 1

5 683 1 16 1 1 643 0568 1

5 684 1 8 1 1 605 0568 1

5 685 2 32 1 2 900 0518 2

5 686 2 8 1 2 772 0568 2

5 687 2 32 1 2 817 0618 2

5 688 2 8 1 2 733 0568 2

5 689 1 16 0 1 870 0518 1

5 690 1 16 1 1 935 0518 1

5 691 2 16 1 2 680 0568 2

5 692 1 16 1 1 883 0468 1

5 693 1 16 1 1 1102 0568 1

5 694 2 16 1 2 781 0568 2

5 695 1 8 0 1 826 0618 1

5 696 1 8 1 1 933 0568 1

5 697 2 32 1 2 1343 0568 2

5 698 2 32 1 2 901 0568 2

5 699 1 8 1 1 676 0618 1

5 700 2 32 1 2 952 0618 2

5 701 1 8 1 1 781 0668 1

5 702 1 8 1 1 996 0518 1

5 703 2 32 1 2 1226 0518 2

5 704 2 8 1 2 787 0618 2

5 705 1 32 1 1 1152 0468 1

5 706 2 8 1 2 654 0668 2

5 707 1 32 1 1 753 0568 1

5 708 2 8 1 2 702 0618 2

5 709 1 8 1 1 652 0668 1

5 710 1 16 1 1 740 0668 1

5 711 2 8 1 2 621 0518 1

5 712 2 32 1 2 877 0668 2

5 713 1 8 1 1 1110 0518 1

5 714 2 32 1 2 1267 0468 2

5 715 1 16 1 1 783 0468 1

5 716 1 16 1 1 722 0468 1

5 717 1 32 1 1 750 0668 1

5 718 2 16 0 2 785 0518 2

5 719 1 8 1 1 772 0618 1

5 720 2 32 1 2 880 0668 2

5 721 2 8 1 2 648 0568 2

5 722 2 16 1 2 927 0568 2

5 723 2 32 1 2 773 0518 2

5 724 1 32 1 1 746 0618 1

5 725 1 32 1 1 1174 0518 1

5 726 2 8 1 2 524 0518 1

5 727 1 32 1 1 985 0468 1

5 728 1 32 1 1 689 0618 1

5 729 2 8 1 2 685 0618 2

5 730 1 8 1 1 689 0618 1

5 731 2 16 1 2 788 0668 2

5 732 1 8 1 1 895 0668 1

5 733 1 16 1 1 556 0668 1

5 734 2 8 1 2 673 0518 2

5 735 1 8 0 1 609 0618 1

5 736 2 32 1 2 1430 0568 2

5 737 1 32 1 1 936 0468 1

5 738 1 8 1 1 1436 0568 1

5 739 1 16 1 1 890 0518 1

5 740 1 32 1 1 632 0618 1

5 741 1 16 1 1 883 0618 1

5 742 2 8 1 2 905 0468 2

5 743 1 8 1 1 673 0468 1

5 744 1 16 1 1 1183 0518 1

5 745 2 8 1 2 677 0668 2

5 746 2 8 0 2 973 0618 2

5 747 1 32 1 1 853 0668 1

5 748 2 32 1 2 894 0518 2

5 749 1 8 1 1 812 0468 1

5 750 1 8 1 1 793 0668 1

5 751 1 32 1 1 746 0668 1

5 752 1 8 1 1 554 0668 2

5 753 1 16 1 1 827 0468 1

5 754 2 8 1 2 749 0518 1

5 755 2 16 1 2 1117 0468 2

5 756 2 16 0 2 761 0468 2

5 757 1 16 0 1 758 0618 1

5 758 1 32 1 1 792 0468 1

5 759 1 16 1 1 819 0468 1

5 760 1 32 1 1 745 0668 1

5 761 1 8 0 1 871 0468 1

5 762 1 8 1 1 882 0518 2

5 763 1 32 1 1 906 0468 1

5 764 2 16 1 2 900 0668 2

5 765 1 32 1 1 733 0468 1

5 766 2 8 1 2 633 0668 2

5 767 1 32 1 1 829 0668 1

5 768 1 32 1 1 696 0668 1

5 769 2 16 1 2 753 0568 2

5 770 2 8 1 2 837 0468 2

5 771 2 8 1 2 655 0668 1

5 772 2 8 1 2 537 0568 2

5 773 2 16 1 2 1343 0618 2

5 774 2 32 1 2 932 0568 2

5 775 2 32 0 2 735 0468 2

5 776 1 32 1 1 708 0618 1

5 777 2 32 1 2 831 0668 2

5 778 2 8 1 2 745 0568 1

5 779 1 16 0 1 1151 0468 1

5 780 1 16 1 1 1010 0568 1

5 781 1 16 1 1 1460 0618 1

5 782 2 16 0 2 705 0518 2

5 783 1 8 1 1 621 0568 1

5 784 2 32 1 2 1411 0568 2

5 785 2 32 0 2 725 0468 2

5 786 2 32 1 2 789 0568 2

5 787 1 32 1 1 893 0568 1

5 788 2 16 1 2 1399 0568 2

5 789 2 32 1 2 1024 0668 2

5 790 2 32 1 2 766 0618 2

5 791 2 16 1 2 827 0618 2

5 792 1 16 1 1 691 0668 1

5 793 1 32 1 1 791 0468 1

5 794 2 16 1 2 794 0618 2

5 795 1 8 1 1 873 0668 2

5 796 2 32 1 2 828 0618 2

5 797 1 32 1 1 915 0668 1

5 798 2 8 1 2 709 0618 2

5 799 2 16 1 2 706 0568 2

5 800 2 16 1 2 652 0668 2

5 801 1 16 1 1 760 0568 1

5 802 2 32 1 2 749 0618 2

5 803 1 8 1 1 1015 0468 1

5 804 1 16 1 1 785 0618 1

5 805 1 32 1 1 680 0518 1

5 806 1 8 1 1 654 0568 1

5 807 2 16 1 2 970 0618 2

5 808 1 8 0 1 537 0568 2

5 809 1 16 0 1 614 0568 1

5 810 2 32 1 2 1007 0668 2

5 811 1 32 1 1 800 0518 1

5 812 1 32 1 1 723 0568 1

5 813 1 16 0 1 1111 0568 1

5 814 1 8 1 1 752 0518 2

5 815 2 32 1 2 884 0668 2

5 816 1 16 1 1 656 0668 1

5 817 1 16 1 1 828 0618 1

5 818 1 32 1 1 867 0468 1

5 819 1 32 1 1 1005 0518 1

5 820 2 8 1 2 722 0618 2

5 821 2 32 1 2 859 0518 2

5 822 1 16 1 1 849 0518 1

5 823 2 8 1 2 727 0518 2

5 824 1 8 1 1 1003 0468 1

5 825 1 16 1 1 718 0518 1

5 826 2 32 1 2 824 0618 2

5 827 2 8 1 2 691 0468 2

5 828 1 8 1 1 743 0568 1

5 829 2 32 1 2 791 0468 2

5 830 2 16 0 2 747 0618 2

5 831 1 32 1 1 735 0618 1

5 832 2 32 1 2 804 0518 2

5 833 2 16 1 2 785 0618 2

5 834 2 32 1 2 830 0468 2

5 835 2 8 1 2 1787 0618 2

5 836 2 32 1 2 837 0668 2

5 837 2 16 1 2 729 0668 2

5 838 2 8 1 2 678 0468 2

5 839 2 32 0 2 737 0668 2

5 840 2 8 1 2 773 0568 2

5 841 1 16 1 1 655 0668 1

5 842 2 32 1 2 864 0618 2

5 843 1 16 1 1 752 0518 1

5 844 2 16 0 2 1041 0518 2

5 845 2 8 0 2 661 0618 2

5 846 1 8 1 1 710 0668 2

5 847 2 8 1 2 753 0468 2

5 848 2 16 0 2 811 0468 2

5 849 2 32 1 2 844 0618 2

5 850 2 32 1 2 810 0618 2

5 851 1 16 1 1 763 0668 1

5 852 2 8 1 2 754 0568 1

5 853 2 16 1 2 749 0668 2

5 854 1 8 1 1 775 0518 1

5 855 2 16 1 2 1132 0518 2

5 856 2 16 1 2 689 0668 2

5 857 1 8 1 1 1211 0468 1

5 858 1 8 1 1 808 0468 1

5 859 1 32 1 1 789 0618 1

5 860 1 8 1 1 720 0518 2

5 861 1 16 1 1 618 0518 1

5 862 2 8 1 2 683 0518 1

5 863 1 8 1 1 586 0518 1

5 864 1 16 1 1 669 0568 1

5 865 2 16 1 2 1149 0468 2

5 866 1 16 1 1 694 0618 1

5 867 2 16 0 2 706 0518 2

5 868 2 16 0 2 861 0568 2

5 869 1 8 1 1 788 0618 1

5 870 1 8 0 1 765 0618 1

5 871 2 8 1 2 747 0518 1

5 872 2 8 1 2 659 0668 2

5 873 1 32 1 1 727 0568 1

5 874 2 8 1 2 623 0518 2

5 875 1 16 1 1 864 0568 1

5 876 2 8 1 2 705 0468 2

5 877 2 32 0 2 712 0468 2

5 878 2 16 1 2 1105 0518 2

5 879 2 8 1 2 633 0568 2

5 880 2 16 1 2 842 0668 2

5 881 1 32 1 1 650 0668 1

5 882 1 16 1 1 880 0618 1

5 883 2 32 1 2 1494 0518 2

5 884 1 16 1 1 682 0468 1

5 885 1 8 1 1 549 0568 2

5 886 1 16 1 1 604 0568 1

5 887 2 16 1 2 1208 0568 2

5 888 1 8 1 1 799 0618 1

5 889 1 8 1 1 856 0618 1

5 890 2 8 1 2 647 0668 1

5 891 1 32 1 1 795 0518 1

5 892 2 8 1 2 602 0668 2

5 893 1 16 1 1 875 0468 1

5 894 1 32 1 1 1146 0518 1

5 895 1 16 1 1 770 0568 1

5 896 1 8 1 1 686 0618 1

5 897 1 32 1 1 954 0618 1

5 898 1 32 1 1 781 0518 1

5 899 2 8 1 2 838 0468 2

5 900 1 32 1 1 879 0468 1

5 901 1 16 1 1 1351 0618 1

5 902 2 8 1 2 708 0668 2

5 903 2 32 1 2 1314 0618 2

5 904 1 16 1 1 1024 0518 1

5 905 2 16 1 2 1588 0468 2

5 906 2 16 1 2 1337 0468 2

5 907 2 16 0 2 1098 0468 2

5 908 2 8 1 2 977 0468 2

5 909 2 32 0 2 786 0568 2

5 910 1 8 1 1 866 0518 1

5 911 2 32 1 2 985 0668 2

5 912 1 32 1 1 705 0568 1

5 913 1 32 0 1 752 0518 1

5 914 1 16 1 1 787 0668 1

5 915 2 32 1 2 1229 0518 2

5 916 1 16 1 1 1738 0468 1

5 917 1 32 1 1 715 0618 1

5 918 2 16 1 2 630 0668 2

5 919 2 32 1 2 1198 0468 2

5 920 1 8 1 1 756 0668 1

5 921 2 8 1 2 607 0668 2

5 922 2 8 1 2 1083 0518 2

5 923 2 8 1 2 876 0618 2

5 924 1 16 1 1 838 0668 1

5 925 2 16 1 2 888 0518 2

5 926 2 16 1 2 1196 0618 2

5 927 1 32 1 1 708 0568 1

5 928 2 8 1 2 807 0618 2

5 929 2 32 0 2 873 0518 2

5 930 2 32 1 2 1057 0468 2

5 931 1 8 1 1 1110 0468 1

5 932 1 16 1 1 1206 0468 1

5 933 2 16 1 2 769 0518 2

5 934 1 8 0 1 1034 0618 1

5 935 1 16 1 1 717 0518 1

5 936 1 8 1 1 614 0668 2

5 937 2 32 1 2 1534 0468 2

5 938 1 8 1 1 680 0668 1

5 939 2 32 1 2 1013 0668 2

5 940 2 32 1 2 975 0568 2

5 941 2 8 1 2 1329 0568 2

5 942 2 32 1 2 854 0568 2

5 943 1 16 1 1 762 0668 1

5 944 2 32 1 2 895 0568 2

5 945 2 8 1 2 793 0568 2

5 946 1 32 1 1 764 0668 1

5 947 1 32 0 1 771 0568 1

5 948 1 8 1 1 747 0518 2

5 949 1 32 1 1 893 0568 1

5 950 2 16 0 2 1493 0468 2

5 951 2 16 1 2 865 0568 2

5 952 2 16 1 2 1438 0618 2

5 953 1 16 1 1 829 0468 1

5 954 2 32 1 2 791 0518 2

5 955 1 8 1 1 700 0568 2

5 956 1 32 1 1 695 0568 1

5 957 1 8 1 1 1016 0518 1

5 958 2 16 1 2 1567 0518 2

5 959 1 8 1 1 705 0568 2

5 960 2 16 1 2 1105 0568 2

5 961 1 8 1 1 595 0668 1

5 962 2 16 1 2 1076 0618 2

5 963 2 16 1 2 1618 0468 2

5 964 1 16 1 1 764 0568 1

5 965 1 32 1 1 771 0668 1

5 966 1 8 1 1 841 0568 2

5 967 2 16 0 2 1326 0468 2

5 968 2 8 1 2 731 0468 2

5 969 1 8 1 1 595 0568 2

5 970 2 16 1 2 1526 0618 2

5 971 1 16 1 1 784 0468 1

5 972 2 16 1 2 1030 0618 2

5 973 2 8 1 2 609 0518 2

5 974 1 32 1 1 798 0468 1

5 975 2 32 1 2 833 0568 2

5 976 1 16 1 1 820 0618 1

5 977 1 8 1 1 1172 0468 1

5 978 2 8 1 2 1284 0568 2

5 979 1 32 1 1 966 0468 1

5 980 1 16 1 1 1176 0518 1

5 981 1 16 1 1 637 0668 1

5 982 1 8 1 1 775 0618 1

5 983 2 8 0 2 669 0618 2

5 984 1 16 0 1 772 0618 1

5 985 1 16 1 1 843 0568 1

5 986 1 32 1 1 751 0468 1

5 987 2 32 1 2 844 0668 2

5 988 1 16 1 1 752 0668 1

5 989 1 16 1 1 585 0668 1

5 990 1 32 1 1 757 0518 1

5 991 2 32 1 2 728 0568 2

5 992 1 32 1 1 800 0668 1

5 993 2 16 1 2 984 0518 2

5 994 2 8 1 2 756 0618 2

5 995 2 8 1 2 622 0668 2

5 996 2 32 0 2 1243 0468 2

5 997 2 16 1 2 1006 0468 2

5 998 1 16 1 1 803 0568 1

5 999 2 32 1 2 841 0618 2

5 1000 1 32 1 1 703 0618 1

5 1001 1 16 1 1 938 0518 1

5 1002 1 8 1 1 777 0518 2

5 1003 2 32 1 2 954 0568 2

5 1004 1 8 1 1 651 0468 1

5 1005 1 8 1 1 516 0518 2

5 1006 2 8 1 2 760 0568 1

5 1007 2 8 1 2 738 0518 1

5 1008 1 32 1 1 827 0568 1

5 1009 1 16 0 1 700 0618 1

5 1010 1 8 1 1 732 0518 2

5 1011 1 16 1 1 744 0518 1

5 1012 1 32 1 1 1032 0618 1

5 1013 1 8 1 1 560 0668 1

5 1014 2 8 1 2 797 0518 1

5 1015 2 32 1 2 1000 0668 2

5 1016 1 32 1 1 675 0618 1

5 1017 2 8 1 2 632 0468 2

5 1018 1 32 1 1 740 0518 1

5 1019 2 16 0 2 946 0568 2

5 1020 1 8 1 1 628 0668 1

5 1021 1 32 1 1 681 0518 1

5 1022 2 16 1 2 1153 0468 2

5 1023 1 16 1 1 649 0518 1

5 1024 1 32 1 1 791 0668 1

5 1025 1 8 0 1 1017 0468 1

5 1026 1 16 1 1 1065 0618 1

5 1027 2 32 0 2 761 0518 2

5 1028 1 8 0 1 668 0618 1

5 1029 1 32 1 1 1119 0468 1

5 1030 1 16 0 1 762 0618 1

5 1031 1 16 1 1 670 0568 1

5 1032 2 8 1 2 792 0618 2

5 1033 2 16 1 2 923 0518 2

5 1034 2 8 1 2 821 0668 1

5 1035 2 8 1 2 742 0568 2

5 1036 1 16 1 1 685 0668 1

5 1037 2 8 1 2 867 0468 2

5 1038 1 16 1 1 804 0618 1

5 1039 2 8 1 2 635 0518 1

5 1040 1 32 1 1 710 0518 1

5 1041 1 32 1 1 697 0618 1

5 1042 1 32 1 1 739 0668 1

5 1043 2 32 0 2 568 0518 2

5 1044 1 32 1 1 667 0618 1

5 1045 1 32 1 1 597 0568 1

5 1046 1 32 1 1 538 0568 1

5 1047 2 8 1 2 737 0668 1

5 1048 2 32 1 2 842 0668 2

5 1049 2 32 1 2 865 0668 2

5 1050 2 8 1 2 650 0518 2

5 1051 2 32 1 2 806 0468 2

5 1052 2 32 1 2 922 0668 2

5 1053 2 16 1 2 780 0668 2

5 1054 1 16 1 1 1187 0468 1

5 1055 1 8 1 1 656 0668 1

5 1056 1 32 1 1 721 0568 1

5 1057 2 32 1 2 741 0568 2

5 1058 2 16 1 2 720 0668 2

5 1059 2 16 1 2 1040 0568 2

5 1060 1 16 1 1 773 0618 1

5 1061 1 8 1 1 640 0668 2

5 1062 2 32 1 2 798 0518 2

5 1063 2 32 1 2 999 0518 2

5 1064 2 16 1 2 1354 0468 2

5 1065 1 16 1 1 1180 0518 1

5 1066 2 8 1 2 610 0618 2

5 1067 1 8 1 1 708 0568 1

5 1068 1 8 1 1 519 0518 2

5 1069 2 16 1 2 676 0668 2

5 1070 1 32 1 1 805 0518 1

5 1071 2 16 0 2 1154 0618 2

5 1072 1 16 1 1 604 0668 1

5 1073 1 8 1 1 812 0568 1

5 1074 2 32 0 2 805 0468 2

5 1075 1 32 1 1 773 0518 1

5 1076 2 32 1 2 746 0568 2

5 1077 1 32 1 1 712 0468 1

5 1078 1 16 1 1 1114 0568 1

5 1079 2 32 1 2 733 0568 2

5 1080 2 16 1 2 864 0518 2

5 1081 2 32 1 2 736 0618 2

5 1082 2 32 1 2 770 0518 2

5 1083 2 16 1 2 894 0618 2

5 1084 1 16 1 1 891 0568 1

5 1085 1 8 1 1 563 0668 2

5 1086 2 8 1 2 642 0668 2

5 1087 2 16 0 2 769 0668 2

5 1088 2 8 0 2 678 0668 2

5 1089 2 32 1 2 1224 0518 2

5 1090 1 8 1 1 915 0618 1

5 1091 2 16 1 2 1108 0518 2

5 1092 1 16 1 1 819 0468 1

5 1093 2 16 1 2 674 0468 2

5 1094 1 32 0 1 1251 0668 1

5 1095 2 16 1 2 1000 0618 2

5 1096 1 8 1 1 850 0468 1

5 1097 1 16 1 1 702 0568 1

5 1098 2 16 0 2 674 0568 2

5 1099 1 32 1 1 705 0668 1

5 1100 2 8 1 2 872 0618 2

5 1101 2 16 1 2 666 0568 2

5 1102 1 32 1 1 604 0618 1

5 1103 1 16 1 1 588 0668 1

5 1104 2 8 1 2 809 0568 2

5 1105 1 8 1 1 695 0518 1

5 1106 2 8 1 2 701 0568 2

5 1107 2 16 1 2 967 0468 2

5 1108 1 16 1 1 613 0518 1

5 1109 1 32 1 1 577 0568 1

5 1110 1 8 1 1 729 0668 2

5 1111 1 8 1 1 624 0618 1

5 1112 2 8 1 2 663 0668 2

5 1113 2 8 1 2 770 0468 2

5 1114 1 32 1 1 838 0468 1

5 1115 1 8 1 1 730 0568 1

5 1116 2 32 0 2 1028 0468 2

5 1117 2 8 0 2 693 0668 2

5 1118 1 8 1 1 2116 0618 1

5 1119 2 32 1 2 835 0668 2

5 1120 1 16 1 1 981 0468 1

5 1121 2 32 1 2 689 0518 2

5 1122 1 8 1 1 1237 0468 1

5 1123 2 16 1 2 715 0668 2

5 1124 1 8 0 1 847 0468 1

5 1125 2 32 1 2 935 0618 2

5 1126 2 16 1 2 948 0568 2

5 1127 2 8 1 2 804 0518 2

5 1128 2 32 1 2 881 0618 2

5 1129 2 16 1 2 717 0568 2

5 1130 1 32 1 1 823 0668 1

5 1131 2 8 1 2 689 0568 1

5 1132 2 8 1 2 904 0468 2

5 1133 1 32 1 1 680 0618 1

5 1134 1 32 1 1 1112 0568 1

5 1135 1 8 1 1 588 0518 1

5 1136 1 16 1 1 840 0468 1

5 1137 2 16 0 2 761 0568 2

5 1138 2 8 1 2 1046 0468 2

5 1139 1 16 1 1 707 0618 1

5 1140 2 16 1 2 805 0668 2

5 1141 1 32 1 1 951 0468 1

5 1142 2 32 1 2 854 0468 2

5 1143 2 8 1 2 580 0568 2

5 1144 2 16 0 2 1043 0518 2

5 1145 1 16 1 1 741 0568 1

5 1146 1 16 1 1 826 0468 1

5 1147 1 8 1 1 706 0568 2

5 1148 1 32 1 1 717 0618 1

5 1149 2 32 1 2 1389 0468 2

5 1150 1 8 1 1 811 0468 1

5 1151 1 8 1 1 658 0618 1

5 1152 2 32 0 2 617 0518 2

5 1153 1 16 1 1 1451 0468 1

5 1154 2 32 1 2 805 0568 2

5 1155 2 8 1 2 935 0618 2

5 1156 1 32 1 1 760 0518 1

5 1157 2 8 1 2 978 0518 1

5 1158 2 32 1 2 811 0618 2

5 1159 1 32 1 1 1110 0468 1

5 1160 2 16 1 2 669 0668 2

5 1161 2 8 1 2 745 0618 2

5 1162 1 32 1 1 849 0568 1

5 1163 1 8 0 1 673 0618 1

5 1164 2 32 1 2 799 0618 2

5 1165 1 32 1 1 671 0518 1

5 1166 2 16 1 2 702 0518 2

5 1167 2 32 0 2 858 0568 2

5 1168 1 32 1 1 826 0568 1

5 1169 2 16 1 2 745 0618 2

5 1170 1 16 1 1 631 0668 1

5 1171 1 8 1 1 891 0518 1

5 1172 1 32 1 1 866 0668 1

5 1173 2 16 1 2 743 0518 2

5 1174 1 8 0 1 652 0468 1

5 1175 2 8 1 2 749 0468 2

5 1176 2 32 1 2 782 0618 2

5 1177 2 8 1 2 803 0618 2

5 1178 1 16 1 1 990 0518 1

5 1179 1 8 1 1 784 0568 2

5 1180 2 8 1 2 598 0668 2

5 1181 2 32 1 2 1300 0618 2

5 1182 2 8 1 2 954 0468 2

5 1183 2 32 1 2 833 0668 2

5 1184 2 16 1 2 924 0618 2

5 1185 2 8 1 2 851 0518 2

5 1186 2 32 0 2 1088 0468 2

5 1187 1 8 1 1 582 0668 2

5 1188 2 16 0 2 1201 0668 2

5 1189 2 32 1 2 1023 0468 2

5 1190 2 32 1 2 885 0668 2

5 1191 1 16 1 1 1090 0468 1

5 1192 1 8 0 1 741 0518 1

5 1193 1 8 1 1 801 0618 1

5 1194 2 8 1 2 709 0568 2

5 1195 1 16 1 1 1092 0518 1

5 1196 2 16 1 2 878 0568 2

5 1197 2 16 1 2 1125 0468 2

5 1198 2 16 1 2 1005 0518 2

5 1199 1 16 1 1 599 0668 1

5 1200 1 8 1 1 864 0568 1

6 1 2 16 1 2 3633 0568 2

6 2 1 16 1 1 2254 0568 1

6 3 2 8 1 2 1872 0468 2

6 4 2 8 1 2 1661 0618 2

6 5 1 8 1 1 1727 0518 2

6 6 1 16 1 1 2275 0618 1

6 7 1 16 1 1 3431 0518 1

6 8 1 16 0 1 3809 0468 1

6 9 2 32 1 2 2575 0568 2

6 10 2 16 1 2 3081 0618 2

6 11 2 32 1 2 1868 0618 2

6 12 2 8 1 2 2403 0568 2

6 13 2 32 1 2 2347 0468 2

6 14 1 8 1 1 1714 0518 2

6 15 1 32 1 1 1989 0518 1

6 16 2 8 1 2 2541 0468 2

6 17 1 16 1 1 3351 0468 1

6 18 1 16 1 1 4042 0568 1

6 19 1 32 1 1 3323 0518 1

6 20 1 8 1 1 2056 0518 2

6 21 1 16 1 1 1718 0618 1

6 22 2 16 1 2 1699 0668 2

6 23 1 8 1 1 1521 0668 1

6 24 1 32 1 1 2474 0468 1

6 25 2 16 1 2 3430 0468 2

6 26 2 16 1 2 2055 0468 2

6 27 1 16 1 1 1748 0618 1

6 28 2 32 1 2 1765 0518 2

6 29 1 8 1 1 1594 0618 1

6 30 2 16 1 2 2029 0618 2

6 31 1 32 1 1 2504 0468 1

6 32 2 32 1 2 1534 0668 2

6 33 2 32 1 2 2934 0668 2

6 34 2 16 1 2 2770 0568 2

6 35 1 32 1 1 1429 0668 1

6 36 2 8 1 2 5447 0468 2

6 37 2 16 1 2 3465 0668 2

6 38 1 32 1 1 3162 0518 1

6 39 2 8 1 2 1559 0568 2

6 40 2 16 1 2 2505 0618 2

6 41 1 8 1 1 1219 0568 1

6 42 2 16 1 2 3586 0568 2

6 43 1 32 1 1 1967 0468 1

6 44 1 32 1 1 1181 0568 1

6 45 2 8 1 2 1173 0568 1

6 46 2 16 1 2 8870 0468 2

6 47 2 8 1 2 3252 0468 2

6 48 1 8 1 1 1578 0468 1

6 49 2 32 1 2 2758 0518 2

6 50 1 8 1 1 1475 0568 1

6 51 1 16 1 1 2281 0518 1

6 52 2 16 1 2 3242 0668 2

6 53 2 16 1 2 3154 0518 2

6 54 1 8 1 1 1415 0518 1

6 55 1 8 1 1 1268 0668 1

6 56 2 16 1 2 4250 0468 2

6 57 1 16 1 1 2051 0568 1

6 58 2 16 1 2 3168 0518 2

6 59 2 16 1 2 1876 0668 2

6 60 1 16 1 1 1695 0618 1

6 61 1 8 1 1 1747 0668 2

6 62 2 32 1 2 1800 0568 2

6 63 2 16 1 2 2849 0468 2

6 64 2 16 1 2 1488 0668 2

6 65 1 8 1 1 1990 0618 1

6 66 1 16 1 1 2599 0518 1

6 67 2 8 1 2 1062 0668 2

6 68 2 32 1 2 1905 0568 2

6 69 1 32 1 1 1337 0668 1

6 70 2 32 1 2 2909 0468 2

6 71 2 16 1 2 3541 0518 2

6 72 1 16 1 1 1610 0668 1

6 73 1 16 1 1 2082 0468 1

6 74 1 32 1 1 2767 0468 1

6 75 2 32 1 2 1405 0468 2

6 76 1 32 1 1 1134 0618 1

6 77 1 8 1 1 2923 0468 1

6 78 2 16 1 2 2044 0668 2

6 79 1 8 1 1 1096 0568 2

6 80 1 8 1 1 1824 0518 1

6 81 1 16 1 1 1020 0668 1

6 82 2 16 1 2 2276 0618 2

6 83 1 16 1 1 1761 0518 1

6 84 1 32 1 1 1742 0668 1

6 85 2 16 1 2 2958 0668 2

6 86 1 8 1 1 4542 0668 1

6 87 2 8 1 2 1788 0668 2

6 88 1 32 1 1 1032 0618 1

6 89 2 32 1 2 2832 0518 2

6 90 1 16 1 1 1978 0618 1

6 91 2 8 1 2 1326 0518 1

6 92 2 32 1 2 1304 0568 2

6 93 2 8 1 2 1562 0568 2

6 94 2 32 1 2 1299 0518 2

6 95 2 32 1 2 1481 0568 2

6 96 2 32 1 2 1568 0618 2

6 97 1 32 1 1 1058 0618 1

6 98 2 16 1 2 3253 0568 2

6 99 2 32 1 2 4227 0468 2

6 100 1 32 1 1 1307 0468 1

6 101 1 8 1 1 1322 0518 1

6 102 1 16 0 1 2855 0468 1

6 103 1 32 1 1 3191 0468 1

6 104 2 16 1 2 3919 0468 2

6 105 2 32 1 2 1848 0568 2

6 106 1 16 1 1 1314 0668 1

6 107 1 32 1 1 1867 0468 1

6 108 2 8 1 2 2635 0618 2

6 109 1 16 1 1 2758 0518 1

6 110 1 16 1 1 1366 0568 1

6 111 2 32 1 2 1237 0618 2

6 112 2 32 1 2 910 0618 2

6 113 1 8 1 1 2053 0618 1

6 114 1 8 1 1 1211 0618 1

6 115 1 16 1 1 4031 0468 1

6 116 1 32 1 1 1291 0618 1

6 117 2 32 1 2 1370 0668 2

6 118 2 16 0 2 1918 0518 2

6 119 2 32 1 2 3634 0468 2

6 120 1 16 1 1 1761 0568 1

6 121 1 32 1 1 1463 0568 1

6 122 1 32 1 1 3137 0618 1

6 123 2 8 1 2 2521 0618 2

6 124 2 8 1 2 1087 0668 1

6 125 1 8 1 1 1714 0518 2

6 126 2 32 1 2 2765 0668 2

6 127 1 16 1 1 3560 0668 1

6 128 2 32 1 2 1132 0668 2

6 129 2 16 1 2 1906 0618 2

6 130 2 8 1 2 911 0468 2

6 131 1 8 1 1 1554 0468 1

6 132 2 8 1 2 1251 0518 2

6 133 2 8 1 2 1530 0518 2

6 134 2 32 1 2 930 0618 2

6 135 2 8 1 2 1193 0518 1

6 136 2 8 1 2 1606 0468 2

6 137 1 8 1 1 1721 0568 2

6 138 2 32 1 2 1144 0618 2

6 139 2 32 1 2 2199 0668 2

6 140 1 32 1 1 1347 0518 1

6 141 2 16 1 2 1006 0568 2

6 142 2 16 1 2 2122 0518 2

6 143 1 32 1 1 1070 0618 1

6 144 1 32 1 1 1630 0518 1

6 145 2 8 1 2 1279 0668 1

6 146 1 16 0 1 1738 0518 1

6 147 1 16 1 1 2334 0568 1

6 148 2 32 1 2 1729 0568 2

6 149 2 16 1 2 2359 0468 2

6 150 1 16 1 1 1469 0668 1

6 151 1 8 1 1 1047 0668 2

6 152 2 8 1 2 1377 0568 1

6 153 2 8 1 2 1437 0518 2

6 154 2 32 1 2 951 0668 2

6 155 1 16 0 1 1374 0518 1

6 156 2 16 1 2 2199 0618 2

6 157 2 16 1 2 1793 0568 2

6 158 2 32 1 2 1228 0668 2

6 159 2 8 1 2 1088 0518 1

6 160 1 16 1 1 2795 0668 1

6 161 1 8 1 1 1519 0568 1

6 162 2 16 1 2 1564 0518 2

6 163 2 8 1 2 1463 0618 2

6 164 1 8 1 1 2003 0618 1

6 165 2 32 1 2 3036 0518 2

6 166 2 32 1 2 2162 0468 2

6 167 1 16 1 1 2725 0668 1

6 168 1 32 1 1 1097 0618 1

6 169 1 8 1 1 1247 0568 1

6 170 1 16 1 1 2076 0568 1

6 171 2 8 1 2 905 0668 2

6 172 2 16 1 2 1709 0568 2

6 173 2 8 1 2 1120 0618 2

6 174 1 8 1 1 1234 0468 1

6 175 1 8 1 1 1845 0468 1

6 176 2 16 1 2 1568 0668 2

6 177 1 8 1 1 1815 0468 1

6 178 1 8 1 1 1509 0468 1

6 179 1 8 1 1 1033 0568 2

6 180 2 16 1 2 2594 0468 2

6 181 1 8 1 1 1377 0518 1

6 182 2 8 1 2 812 0618 2

6 183 1 32 1 1 1267 0568 1

6 184 1 32 1 1 1628 0518 1

6 185 1 32 1 1 1073 0668 1

6 186 1 16 1 1 1788 0618 1

6 187 1 8 1 1 1279 0468 1

6 188 1 32 1 1 1212 0468 1

6 189 2 8 1 2 704 0668 2

6 190 2 8 1 2 845 0618 2

6 191 1 8 1 1 910 0618 1

6 192 1 32 1 1 1159 0568 1

6 193 1 8 1 1 897 0668 2

6 194 2 16 1 2 1443 0518 2

6 195 2 16 1 2 1242 0618 2

6 196 2 8 0 2 1125 0568 2

6 197 1 8 1 1 1328 0668 1

6 198 2 32 1 2 1608 0518 2

6 199 2 8 1 2 1264 0468 2

6 200 2 8 1 2 919 0668 2

6 201 1 16 1 1 4051 0468 1

6 202 2 32 1 2 1112 0568 2

6 203 1 8 1 1 1207 0568 2

6 204 2 8 1 2 914 0568 2

6 205 2 16 0 2 1030 0568 2

6 206 1 32 1 1 1970 0518 1

6 207 1 32 1 1 3884 0518 1

6 208 1 8 1 1 4640 0618 1

6 209 1 32 1 1 831 0568 1

6 210 1 32 1 1 953 0668 1

6 211 1 16 1 1 2228 0668 1

6 212 2 32 1 2 1227 0518 2

6 213 2 8 1 2 1622 0468 2

6 214 2 16 1 2 1387 0618 2

6 215 1 8 1 1 1178 0668 2

6 216 2 32 1 2 2789 0518 2

6 217 2 32 1 2 2251 0468 2

6 218 1 32 1 1 1061 0668 1

6 219 2 32 1 2 1530 0618 2

6 220 1 16 1 1 1565 0568 1

6 221 1 32 1 1 1212 0618 1

6 222 1 16 1 1 1937 0468 1

6 223 1 32 1 1 1314 0668 1

6 224 1 32 1 1 1008 0568 1

6 225 1 16 1 1 2670 0468 1

6 226 2 32 1 2 2003 0618 2

6 227 2 8 1 2 1322 0518 2

6 228 1 16 1 1 1411 0618 1

6 229 2 32 1 2 997 0468 2

6 230 2 8 1 2 1041 0568 2

6 231 1 16 1 1 1277 0518 1

6 232 1 32 1 1 1227 0668 1

6 233 2 8 1 2 811 0668 2

6 234 2 8 1 2 914 0518 1

6 235 2 8 1 2 701 0618 2

6 236 1 32 1 1 889 0568 1

6 237 2 16 1 2 943 0518 2

6 238 1 8 1 1 1253 0618 1

6 239 1 16 1 1 1837 0618 1

6 240 1 32 1 1 979 0568 1

6 241 2 8 1 2 786 0618 2

6 242 2 16 1 2 1139 0618 2

6 243 2 32 1 2 1476 0468 2

6 244 2 16 1 2 1401 0618 2

6 245 2 32 1 2 1360 0618 2

6 246 2 32 1 2 1246 0518 2

6 247 1 8 1 1 1103 0618 1

6 248 2 32 1 2 1033 0618 2

6 249 2 32 1 2 1105 0568 2

6 250 2 8 1 2 2067 0518 1

6 251 1 8 1 1 1196 0568 2

6 252 1 32 1 1 927 0618 1

6 253 1 8 1 1 3208 0518 1

6 254 2 8 1 2 721 0668 2

6 255 2 8 1 2 1602 0468 2

6 256 1 16 1 1 3145 0518 1

6 257 2 32 1 2 1225 0518 2

6 258 1 32 1 1 872 0568 1

6 259 2 16 1 2 1520 0618 2

6 260 1 32 1 1 810 0668 1

6 261 2 32 1 2 1621 0518 2

6 262 2 8 1 2 2418 0468 2

6 263 1 8 1 1 1111 0518 1

6 264 1 8 1 1 1169 0568 1

6 265 2 32 1 2 2092 0468 2

6 266 2 16 1 2 2813 0568 2

6 267 1 16 1 1 1303 0618 1

6 268 1 16 1 1 1296 0518 1

6 269 2 8 1 2 986 0618 2

6 270 1 32 1 1 849 0618 1

6 271 2 16 1 2 2217 0518 2

6 272 2 16 0 2 2391 0668 2

6 273 2 32 1 2 1519 0468 2

6 274 1 32 1 1 2574 0468 1

6 275 2 16 1 2 3507 0468 2

6 276 2 32 1 2 1009 0568 2

6 277 1 32 1 1 1180 0518 1

6 278 1 8 1 1 1058 0618 1

6 279 1 32 1 1 1079 0568 1

6 280 2 16 1 2 1311 0668 2

6 281 1 32 1 1 1226 0568 1

6 282 1 32 1 1 1024 0668 1

6 283 2 16 1 2 1037 0518 2

6 284 1 32 1 1 785 0568 1

6 285 2 8 1 2 943 0618 2

6 286 1 16 1 1 1644 0468 1

6 287 2 16 1 2 1018 0518 2

6 288 2 16 0 2 2378 0468 2

6 289 2 16 1 2 1038 0568 2

6 290 2 8 1 2 966 0668 2

6 291 1 16 1 1 1791 0618 1

6 292 2 32 0 2 2026 0468 2

6 293 1 16 1 1 3091 0668 1

6 294 1 8 1 1 1115 0568 1

6 295 1 16 1 1 1038 0618 1

6 296 2 8 1 2 997 0618 2

6 297 2 16 1 2 1045 0568 2

6 298 1 8 1 1 1954 0468 1

6 299 1 32 1 1 835 0668 1

6 300 1 16 1 1 1195 0668 1

6 301 2 8 1 2 990 0518 2

6 302 1 16 1 1 1072 0468 1

6 303 2 8 1 2 818 0468 2

6 304 1 8 1 1 1390 0668 1

6 305 2 32 1 2 948 0518 2

6 306 2 8 0 2 828 0618 2

6 307 1 8 1 1 1777 0618 1

6 308 2 16 1 2 1160 0618 2

6 309 1 16 1 1 3661 0468 1

6 310 2 32 1 2 990 0568 2

6 311 1 32 1 1 2574 0468 1

6 312 1 8 1 1 1177 0618 1

6 313 1 8 0 1 825 0568 2

6 314 2 8 1 2 2036 0468 2

6 315 2 32 1 2 1456 0518 2

6 316 1 16 1 1 1395 0618 1

6 317 1 16 1 1 2162 0518 1

6 318 1 32 1 1 937 0568 1

6 319 1 8 1 1 1058 0618 1

6 320 2 8 1 2 763 0468 2

6 321 2 16 1 2 1471 0568 2

6 322 1 32 1 1 847 0618 1

6 323 2 8 1 2 983 0568 2

6 324 1 8 1 1 966 0468 1

6 325 1 16 1 1 1341 0468 1

6 326 1 8 1 1 917 0518 1

6 327 1 32 1 1 776 0618 1

6 328 1 8 1 1 1175 0618 1

6 329 1 8 1 1 1004 0568 1

6 330 1 16 1 1 2175 0618 1

6 331 2 32 1 2 1151 0668 2

6 332 1 32 1 1 1554 0468 1

6 333 2 32 1 2 1320 0468 2

6 334 2 8 1 2 1209 0518 2

6 335 2 16 1 2 1472 0618 2

6 336 2 16 1 2 1996 0568 2

6 337 2 16 1 2 1600 0568 2

6 338 1 32 1 1 972 0518 1

6 339 2 16 1 2 1042 0618 2

6 340 1 32 1 1 862 0568 1

6 341 2 16 1 2 1167 0618 2

6 342 1 8 1 1 1446 0468 1

6 343 2 32 1 2 899 0618 2

6 344 1 32 1 1 1311 0468 1

6 345 1 32 1 1 870 0668 1

6 346 2 8 1 2 895 0618 2

6 347 1 16 1 1 1914 0568 1

6 348 1 8 1 1 1058 0618 1

6 349 2 8 1 2 1115 0568 2

6 350 2 16 1 2 2246 0468 2

6 351 2 16 1 2 1535 0568 2

6 352 2 32 1 2 1054 0618 2

6 353 1 32 1 1 1026 0568 1

6 354 2 32 0 2 1341 0518 2

6 355 2 8 1 2 1204 0518 2

6 356 2 32 1 2 1168 0568 2

6 357 1 8 1 1 906 0568 1

6 358 1 8 1 1 1756 0618 1

6 359 2 8 1 2 789 0568 2

6 360 2 8 1 2 1151 0518 1

6 361 1 16 1 1 951 0668 1

6 362 1 16 1 1 1390 0518 1

6 363 1 8 1 1 1178 0668 1

6 364 1 32 1 1 840 0618 1

6 365 1 16 1 1 1414 0468 1

6 366 1 32 1 1 1058 0618 1

6 367 1 16 1 1 1016 0668 1

6 368 2 16 1 2 1064 0668 2

6 369 2 8 1 2 1753 0518 2

6 370 1 32 1 1 1362 0518 1

6 371 2 16 1 2 1060 0668 2

6 372 1 8 1 1 835 0518 2

6 373 1 16 1 1 912 0668 1

6 374 2 32 1 2 1434 0468 2

6 375 1 8 1 1 906 0668 1

6 376 1 8 1 1 1353 0468 1

6 377 2 8 1 2 1165 0618 2

6 378 2 32 1 2 2137 0468 2

6 379 2 16 1 2 1257 0568 2

6 380 1 16 1 1 2709 0468 1

6 381 2 32 1 2 1022 0618 2

6 382 1 8 1 1 739 0668 2

6 383 2 32 1 2 1424 0468 2

6 384 2 16 1 2 1365 0468 2

6 385 1 8 1 1 984 0518 2

6 386 1 32 1 1 1055 0668 1

6 387 1 8 1 1 1855 0468 1

6 388 2 32 1 2 795 0668 2

6 389 1 32 1 1 1496 0468 1

6 390 1 16 1 1 1003 0568 1

6 391 1 16 1 1 3491 0618 1

6 392 1 16 1 1 1242 0568 1

6 393 2 32 1 2 1211 0668 2

6 394 2 16 1 2 1418 0518 2

6 395 1 32 1 1 1118 0518 1

6 396 1 16 1 1 2232 0518 1

6 397 1 16 1 1 3593 0518 1

6 398 1 32 1 1 1898 0568 1

6 399 1 32 1 1 1393 0518 1

6 400 1 32 0 1 1366 0518 1

6 401 1 16 1 1 886 0668 1

6 402 2 32 1 2 1328 0568 2

6 403 1 16 0 1 2991 0468 1

6 404 1 8 1 1 3859 0468 1

6 405 2 16 1 2 824 0668 2

6 406 2 8 1 2 883 0668 1

6 407 1 8 1 1 5064 0568 2

6 408 2 16 1 2 2162 0468 2

6 409 1 32 1 1 1056 0668 1

6 410 2 16 1 2 1286 0618 2

6 411 2 8 1 2 1027 0468 2

6 412 1 16 1 1 2613 0618 1

6 413 2 32 1 2 1149 0568 2

6 414 2 16 1 2 1088 0518 2

6 415 2 32 1 2 888 0668 2

6 416 2 32 1 2 714 0618 2

6 417 1 16 1 1 1214 0568 1

6 418 2 16 1 2 1080 0518 2

6 419 2 8 1 2 988 0568 2

6 420 2 8 1 2 2731 0468 2

6 421 2 32 1 2 714 0668 2

6 422 2 32 1 2 858 0568 2

6 423 2 16 0 2 1940 0518 2

6 424 1 8 1 1 1716 0518 2

6 425 2 8 1 2 915 0668 1

6 426 2 32 1 2 884 0618 2

6 427 2 32 1 2 799 0668 2

6 428 2 32 1 2 942 0618 2

6 429 2 8 1 2 873 0668 2

6 430 1 8 1 1 1738 0468 1

6 431 1 16 1 1 1436 0668 1

6 432 1 16 1 1 1063 0568 1

6 433 1 32 1 1 1122 0518 1

6 434 2 8 1 2 1092 0568 1

6 435 1 8 1 1 1764 0668 1

6 436 1 32 1 1 1401 0468 1

6 437 2 8 1 2 1044 0568 2

6 438 2 16 0 2 2458 0468 2

6 439 2 8 1 2 1127 0518 1

6 440 2 8 1 2 1126 0568 2

6 441 1 8 1 1 758 0668 2

6 442 2 16 1 2 754 0668 2

6 443 1 16 1 1 1135 0618 1

6 444 2 8 1 2 983 0618 2

6 445 1 16 1 1 1104 0518 1

6 446 1 8 1 1 1226 0468 1

6 447 2 16 1 2 675 0668 2

6 448 1 32 1 1 982 0518 1

6 449 2 32 1 2 1759 0518 2

6 450 2 16 1 2 1278 0468 2

6 451 1 8 1 1 766 0668 2

6 452 1 32 1 1 1553 0468 1

6 453 2 32 1 2 707 0668 2

6 454 2 8 1 2 1085 0518 1

6 455 2 16 1 2 3083 0468 2

6 456 1 16 1 1 784 0668 1

6 457 1 32 1 1 1072 0668 1

6 458 2 16 1 2 883 0668 2

6 459 1 32 1 1 2051 0668 1

6 460 2 8 1 2 1273 0668 2

6 461 1 32 1 1 829 0618 1

6 462 1 16 1 1 1342 0518 1

6 463 1 16 1 1 965 0568 1

6 464 2 8 1 2 909 0568 1

6 465 2 8 1 2 698 0468 2

6 466 1 8 1 1 1065 0568 2

6 467 1 8 1 1 817 0668 2

6 468 2 32 1 2 1027 0568 2

6 469 2 32 1 2 1234 0518 2

6 470 1 32 1 1 924 0618 1

6 471 1 16 1 1 1829 0468 1

6 472 2 8 1 2 856 0668 2

6 473 2 8 1 2 945 0668 2

6 474 1 16 1 1 1193 0568 1

6 475 1 8 1 1 1361 0518 2

6 476 1 16 1 1 1265 0568 1

6 477 1 8 1 1 1197 0518 1

6 478 2 32 1 2 1184 0668 2

6 479 1 32 1 1 1432 0468 1

6 480 2 16 1 2 1311 0518 2

6 481 1 16 1 1 1223 0568 1

6 482 2 32 1 2 1455 0468 2

6 483 1 16 1 1 865 0668 1

6 484 1 32 1 1 964 0568 1

6 485 1 8 1 1 844 0568 2

6 486 2 8 1 2 719 0618 2

6 487 1 8 1 1 757 0568 1

6 488 1 8 1 1 1710 0568 2

6 489 2 8 1 2 758 0568 2

6 490 1 8 1 1 771 0518 2

6 491 2 8 1 2 723 0518 1

6 492 1 32 1 1 1757 0518 1

6 493 2 8 1 2 1363 0468 2

6 494 1 32 1 1 1299 0518 1

6 495 1 32 1 1 1050 0668 1

6 496 2 32 1 2 1011 0518 2

6 497 1 16 1 1 2123 0618 1

6 498 2 8 1 2 1909 0468 2

6 499 2 8 1 2 1392 0518 1

6 500 2 8 1 2 897 0568 2

6 501 1 8 1 1 890 0568 2

6 502 1 16 1 1 2064 0468 1

6 503 2 8 1 2 1000 0618 2

6 504 1 32 1 1 875 0618 1

6 505 2 16 1 2 1261 0618 2

6 506 2 16 0 2 1066 0618 2

6 507 2 32 1 2 1238 0568 2

6 508 2 32 1 2 1207 0668 2

6 509 1 16 1 1 1170 0568 1

6 510 2 32 1 2 1353 0468 2

6 511 1 8 1 1 1138 0618 1

6 512 2 8 1 2 1615 0468 2

6 513 1 32 1 1 793 0668 1

6 514 1 32 1 1 990 0568 1

6 515 1 32 1 1 1676 0568 1

6 516 1 16 1 1 1243 0568 1

6 517 1 8 1 1 1035 0518 1

6 518 2 32 1 2 1457 0468 2

6 519 2 32 1 2 1916 0468 2

6 520 1 16 1 1 1020 0568 1

6 521 1 8 1 1 983 0518 1

6 522 2 32 1 2 974 0568 2

6 523 2 16 1 2 1968 0468 2

6 524 2 16 1 2 2808 0468 2

6 525 1 16 1 1 2490 0468 1

6 526 1 8 1 1 856 0668 2

6 527 1 16 1 1 1586 0668 1

6 528 1 32 1 1 910 0618 1

6 529 2 16 1 2 2434 0468 2

6 530 1 8 1 1 803 0668 1

6 531 2 32 1 2 1604 0618 2

6 532 2 16 1 2 1936 0568 2

6 533 2 32 1 2 1240 0668 2

6 534 1 32 1 1 1357 0468 1

6 535 1 16 1 1 2025 0468 1

6 536 2 8 1 2 1848 0568 2

6 537 2 16 1 2 1178 0668 2

6 538 1 16 1 1 1726 0568 1

6 539 1 8 1 1 1218 0518 2

6 540 1 32 1 1 873 0568 1

6 541 2 16 1 2 988 0568 2

6 542 1 16 1 1 1507 0618 1

6 543 2 32 1 2 962 0568 2

6 544 1 8 1 1 1073 0618 1

6 545 1 8 1 1 1163 0468 1

6 546 1 32 1 1 1074 0468 1

6 547 2 16 1 2 1265 0518 2

6 548 2 8 1 2 1159 0468 2

6 549 2 16 1 2 707 0668 2

6 550 2 16 1 2 2697 0518 2

6 551 1 8 1 1 933 0618 1

6 552 2 32 1 2 1467 0518 2

6 553 1 8 1 1 896 0618 1

6 554 2 32 1 2 858 0668 2

6 555 2 32 1 2 1097 0468 2

6 556 2 8 1 2 925 0668 2

6 557 1 16 1 1 1265 0568 1

6 558 1 16 1 1 1564 0468 1

6 559 2 16 1 2 1593 0518 2

6 560 1 8 1 1 768 0668 1

6 561 2 8 1 2 800 0618 2

6 562 2 32 1 2 1329 0618 2

6 563 1 32 1 1 1387 0518 1

6 564 1 16 1 1 839 0668 1

6 565 2 32 1 2 1145 0518 2

6 566 1 32 1 1 1080 0668 1

6 567 2 8 1 2 1918 0518 2

6 568 1 32 1 1 1192 0468 1

6 569 2 32 1 2 1503 0618 2

6 570 1 8 1 1 1145 0618 1

6 571 2 16 1 2 1207 0568 2

6 572 2 8 1 2 987 0668 2

6 573 1 16 1 1 820 0668 1

6 574 2 32 1 2 1298 0518 2

6 575 2 8 1 2 967 0668 1

6 576 1 32 1 1 1280 0468 1

6 577 2 32 1 2 923 0668 2

6 578 1 16 1 1 1040 0618 1

6 579 1 32 1 1 1546 0518 1

6 580 2 32 1 2 813 0618 2

6 581 2 32 1 2 1161 0668 2

6 582 2 16 1 2 1195 0668 2

6 583 1 32 1 1 965 0618 1

6 584 2 8 1 2 2458 0468 2

6 585 1 16 1 1 1641 0468 1

6 586 1 8 1 1 1605 0468 1

6 587 2 16 1 2 861 0518 2

6 588 2 16 1 2 1062 0518 2

6 589 2 16 1 2 2728 0618 2

6 590 2 32 1 2 1948 0518 2

6 591 2 8 1 2 1353 0468 2

6 592 1 16 0 1 1749 0468 1

6 593 2 16 1 2 1062 0568 2

6 594 1 8 1 1 892 0468 1

6 595 1 32 1 1 976 0518 1

6 596 1 32 1 1 936 0568 1

6 597 1 16 1 1 1767 0518 1

6 598 1 8 1 1 933 0568 1

6 599 1 16 1 1 3323 0468 1

6 600 1 8 1 1 824 0668 1

6 601 2 32 1 2 1101 0618 2

6 602 2 8 1 2 859 0618 2

6 603 1 8 1 1 826 0668 2

6 604 2 16 1 2 2274 0618 2

6 605 1 8 1 1 701 0668 1

6 606 2 16 1 2 2132 0468 2

6 607 1 16 1 1 892 0668 1

6 608 1 32 1 1 777 0618 1

6 609 2 32 1 2 1226 0468 2

6 610 2 32 1 2 1048 0568 2

6 611 2 16 1 2 1079 0618 2

6 612 2 32 1 2 1237 0468 2

6 613 2 32 1 2 1113 0568 2

6 614 2 16 1 2 722 0668 2

6 615 2 8 1 2 805 0568 2

6 616 2 16 1 2 1119 0568 2

6 617 1 16 1 1 832 0518 1

6 618 2 32 1 2 1008 0568 2

6 619 1 32 1 1 839 0668 1

6 620 2 16 1 2 1048 0518 2

6 621 1 16 1 1 1029 0518 1

6 622 2 8 1 2 1434 0468 2

6 623 2 32 1 2 909 0468 2

6 624 1 32 1 1 733 0568 1

6 625 1 32 1 1 1253 0468 1

6 626 1 32 1 1 786 0668 1

6 627 1 16 1 1 1292 0618 1

6 628 1 8 1 1 918 0518 2

6 629 1 8 1 1 706 0668 2

6 630 2 16 1 2 943 0668 2

6 631 1 16 1 1 1017 0618 1

6 632 1 32 1 1 977 0618 1

6 633 1 8 1 1 1713 0468 1

6 634 2 32 1 2 1135 0568 2

6 635 2 32 1 2 1180 0668 2

6 636 1 8 1 1 950 0518 2

6 637 1 32 1 1 948 0468 1

6 638 2 16 1 2 1148 0618 2

6 639 2 8 1 2 679 0668 2

6 640 2 8 1 2 1044 0618 2

6 641 1 32 1 1 819 0518 1

6 642 1 8 1 1 1213 0618 1

6 643 1 32 1 1 952 0668 1

6 644 2 16 1 2 1456 0618 2

6 645 1 8 1 1 1279 0568 2

6 646 2 16 1 2 1665 0468 2

6 647 1 8 1 1 898 0618 1

6 648 1 16 1 1 1420 0668 1

6 649 1 8 1 1 959 0468 1

6 650 1 8 1 1 1174 0568 1

6 651 2 32 1 2 986 0668 2

6 652 1 16 1 1 1106 0518 1

6 653 2 16 1 2 807 0668 2

6 654 1 32 1 1 822 0618 1

6 655 2 16 1 2 741 0668 2

6 656 2 8 1 2 1455 0568 2

6 657 1 8 1 1 1366 0468 1

6 658 1 16 1 1 1182 0568 1

6 659 1 32 1 1 1567 0518 1

6 660 2 8 1 2 1453 0568 1

6 661 2 32 1 2 1038 0568 2

6 662 2 32 1 2 961 0518 2

6 663 1 16 1 1 1150 0518 1

6 664 1 8 1 1 922 0668 2

6 665 2 32 1 2 851 0618 2

6 666 2 16 1 2 1569 0518 2

6 667 1 16 1 1 1526 0518 1

6 668 1 16 1 1 1617 0468 1

6 669 2 16 1 2 1166 0468 2

6 670 2 8 1 2 744 0618 2

6 671 2 8 1 2 885 0568 2

6 672 2 8 1 2 604 0668 2

6 673 2 32 1 2 879 0618 2

6 674 2 16 1 2 1525 0468 2

6 675 1 32 1 1 1509 0468 1

6 676 2 32 1 2 998 0518 2

6 677 1 32 1 1 919 0518 1

6 678 1 32 1 1 883 0668 1

6 679 2 16 1 2 1196 0618 2

6 680 1 16 1 1 1163 0518 1

6 681 2 8 1 2 674 0518 2

6 682 2 8 1 2 1285 0468 2

6 683 2 8 1 2 815 0668 2

6 684 1 32 1 1 1085 0618 1

6 685 1 16 1 1 1342 0518 1

6 686 1 8 1 1 1501 0468 1

6 687 2 8 1 2 780 0568 1

6 688 1 32 1 1 765 0568 1

6 689 1 8 1 1 1109 0518 1

6 690 2 8 1 2 811 0618 2

6 691 2 16 1 2 1252 0568 2

6 692 2 8 1 2 757 0668 2

6 693 1 32 1 1 801 0618 1

6 694 1 32 1 1 2273 0468 1

6 695 1 16 1 1 1223 0618 1

6 696 2 16 1 2 1623 0468 2

6 697 2 8 1 2 960 0518 2

6 698 2 16 1 2 1019 0518 2

6 699 2 8 1 2 730 0668 1

6 700 2 32 1 2 1143 0668 2

6 701 1 16 1 1 2882 0618 1

6 702 1 8 1 1 1792 0468 1

6 703 2 8 1 2 1000 0518 1

6 704 1 16 1 1 1050 0568 1

6 705 1 8 1 1 908 0568 1

6 706 1 16 1 1 1049 0668 1

6 707 2 16 1 2 1423 0668 2

6 708 2 8 1 2 1503 0518 1

6 709 1 16 1 1 1171 0668 1

6 710 1 32 1 1 1032 0668 1

6 711 2 16 1 2 788 0568 2

6 712 1 32 1 1 829 0568 1

6 713 2 8 1 2 966 0518 2

6 714 2 8 1 2 1331 0618 2

6 715 2 32 1 2 1100 0518 2

6 716 2 32 1 2 1307 0618 2

6 717 1 8 1 1 1175 0518 1

6 718 1 16 1 1 1512 0618 1

6 719 2 16 1 2 1339 0568 2

6 720 1 8 1 1 852 0618 1

6 721 2 32 1 2 1219 0518 2

6 722 1 32 1 1 1197 0468 1

6 723 1 8 1 1 902 0568 1

6 724 1 16 1 1 1095 0468 1

6 725 1 32 1 1 1237 0468 1

6 726 2 8 1 2 1087 0468 2

6 727 1 16 1 1 1361 0468 1

6 728 2 32 1 2 963 0618 2

6 729 1 16 1 1 929 0668 1

6 730 2 16 1 2 818 0618 2

6 731 2 32 1 2 1080 0668 2

6 732 1 32 1 1 1375 0518 1

6 733 2 32 1 2 1138 0568 2

6 734 2 32 1 2 1148 0468 2

6 735 2 16 1 2 929 0568 2

6 736 1 32 1 1 822 0618 1

6 737 1 32 1 1 1075 0618 1

6 738 1 32 1 1 978 0518 1

6 739 1 32 1 1 1294 0468 1

6 740 2 8 1 2 930 0568 2

6 741 1 32 1 1 851 0618 1

6 742 1 16 1 1 1203 0518 1

6 743 2 32 1 2 1224 0568 2

6 744 1 32 1 1 972 0468 1

6 745 1 16 1 1 1483 0568 1

6 746 1 16 1 1 1361 0618 1

6 747 1 8 1 1 888 0668 1

6 748 1 8 1 1 731 0668 1

6 749 2 8 1 2 1082 0568 2

6 750 1 8 1 1 1941 0468 1

6 751 2 16 1 2 1301 0668 2

6 752 2 32 1 2 1261 0518 2

6 753 2 32 1 2 829 0668 2

6 754 1 16 1 1 998 0668 1

6 755 1 8 1 1 1225 0618 1

6 756 2 32 1 2 887 0618 2

6 757 2 8 1 2 830 0568 2

6 758 2 16 0 2 1756 0618 2

6 759 1 32 1 1 1495 0468 1

6 760 1 32 1 1 1042 0668 1

6 761 1 8 1 1 772 0668 2

6 762 2 8 1 2 674 0668 1

6 763 2 16 1 2 1069 0568 2

6 764 2 8 1 2 1247 0518 1

6 765 1 32 1 1 1306 0618 1

6 766 2 32 1 2 1558 0568 2

6 767 2 8 1 2 728 0668 2

6 768 2 32 1 2 1048 0618 2

6 769 1 32 1 1 1295 0468 1

6 770 2 16 1 2 1037 0668 2

6 771 1 32 1 1 1420 0518 1

6 772 1 32 1 1 1014 0468 1

6 773 2 8 1 2 731 0618 2

6 774 1 8 1 1 1310 0568 1

6 775 1 8 1 1 1869 0518 1

6 776 1 16 1 1 913 0668 1

6 777 2 8 1 2 700 0618 2

6 778 2 16 1 2 1034 0668 2

6 779 1 8 1 1 738 0668 2

6 780 1 16 1 1 2934 0468 1

6 781 2 32 1 2 1067 0618 2

6 782 2 32 1 2 2006 0468 2

6 783 2 32 1 2 1386 0668 2

6 784 1 32 1 1 999 0568 1

6 785 1 16 1 1 1015 0668 1

6 786 2 16 1 2 1393 0618 2

6 787 2 16 1 2 1811 0668 2

6 788 2 32 1 2 1001 0568 2

6 789 2 16 1 2 1449 0568 2

6 790 2 16 1 2 1155 0618 2

6 791 2 32 1 2 2653 0668 2

6 792 1 32 1 1 811 0618 1

6 793 2 32 1 2 1479 0518 2

6 794 1 8 1 1 1826 0468 1

6 795 2 16 1 2 4622 0468 2

6 796 2 8 1 2 2674 0468 2

6 797 2 32 1 2 1168 0468 2

6 798 1 16 0 1 1288 0518 1

6 799 1 16 1 1 1774 0618 1

6 800 2 8 1 2 3273 0618 2

6 801 1 32 1 1 887 0618 1

6 802 1 8 1 1 1441 0568 1

6 803 2 8 1 2 1228 0518 2

6 804 1 8 1 1 981 0518 1

6 805 1 16 1 1 2028 0568 1

6 806 2 8 1 2 1151 0468 2

6 807 1 32 1 1 1186 0668 1

6 808 1 16 1 1 1770 0568 1

6 809 1 16 1 1 1244 0568 1

6 810 1 8 1 1 1625 0618 1

6 811 2 16 1 2 1250 0468 2

6 812 2 8 1 2 873 0468 2

6 813 1 8 1 1 844 0618 1

6 814 1 32 1 1 1395 0618 1

6 815 1 16 1 1 1875 0518 1

6 816 1 16 1 1 1517 0518 1

6 817 1 8 1 1 880 0668 1

6 818 1 32 1 1 724 0568 1

6 819 1 16 1 1 2939 0468 1

6 820 1 8 1 1 941 0618 1

6 821 1 8 1 1 830 0468 1

6 822 2 16 1 2 1239 0468 2

6 823 2 16 1 2 859 0568 2

6 824 1 16 1 1 830 0668 1

6 825 2 8 1 2 1199 0518 1

6 826 1 32 1 1 810 0568 1

6 827 2 16 1 2 862 0518 2

6 828 1 8 1 1 1862 0618 1

6 829 2 8 1 2 808 0618 2

6 830 1 8 0 1 1181 0468 1

6 831 2 8 1 2 837 0618 2

6 832 2 16 1 2 777 0668 2

6 833 1 16 1 1 1153 0618 1

6 834 2 8 1 2 842 0668 2

6 835 1 8 1 1 3132 0468 1

6 836 1 8 1 1 1080 0518 2

6 837 2 32 1 2 967 0618 2

6 838 2 16 1 2 2650 0518 2

6 839 1 32 1 1 1541 0518 1

6 840 1 32 1 1 1085 0468 1

6 841 1 16 1 1 2171 0468 1

6 842 1 8 1 1 852 0668 1

6 843 2 32 1 2 977 0668 2

6 844 2 16 1 2 1217 0618 2

6 845 2 16 1 2 1393 0518 2

6 846 2 16 1 2 1525 0468 2

6 847 1 16 1 1 1429 0568 1

6 848 2 16 1 2 2704 0468 2

6 849 1 32 1 1 1221 0518 1

6 850 1 8 1 1 1052 0568 1

6 851 1 16 1 1 959 0618 1

6 852 2 8 1 2 772 0568 1

6 853 2 32 1 2 1280 0568 2

6 854 2 8 1 2 1117 0468 2

6 855 2 16 1 2 1198 0518 2

6 856 1 8 1 1 1117 0668 2

6 857 2 32 1 2 980 0468 2

6 858 2 8 1 2 690 0668 2

6 859 1 32 1 1 1143 0568 1

6 860 1 32 1 1 1196 0568 1

6 861 2 8 1 2 874 0668 2

6 862 2 16 1 2 2179 0518 2

6 863 1 8 1 1 2502 0468 1

6 864 1 8 1 1 925 0618 1

6 865 1 16 1 1 1672 0518 1

6 866 1 16 0 1 1484 0468 1

6 867 1 8 1 1 1371 0518 2

6 868 1 8 1 1 960 0468 1

6 869 2 8 1 2 653 0468 2

6 870 2 8 1 2 1066 0518 2

6 871 2 8 1 2 1494 0518 1

6 872 2 16 1 2 1779 0518 2

6 873 2 32 1 2 1349 0668 2

6 874 1 8 1 1 1538 0518 1

6 875 2 16 1 2 1411 0668 2

6 876 2 16 1 2 2235 0568 2

6 877 2 16 1 2 1138 0618 2

6 878 2 16 1 2 1246 0568 2

6 879 1 32 1 1 1007 0568 1

6 880 1 16 1 1 2433 0618 1

6 881 1 32 1 1 851 0618 1

6 882 1 32 1 1 2250 0518 1

6 883 2 8 1 2 862 0668 2

6 884 1 32 1 1 913 0668 1

6 885 2 8 1 2 1480 0468 2

6 886 2 32 1 2 1102 0518 2

6 887 2 32 1 2 1073 0618 2

6 888 2 16 1 2 1636 0518 2

6 889 2 8 1 2 1451 0518 2

6 890 2 8 1 2 717 0568 2

6 891 1 16 1 1 1074 0618 1

6 892 1 8 1 1 1202 0568 2

6 893 2 16 1 2 1891 0518 2

6 894 2 8 1 2 738 0568 1

6 895 2 16 1 2 1752 0468 2

6 896 1 32 1 1 833 0668 1

6 897 2 8 1 2 878 0518 2

6 898 2 32 1 2 1324 0518 2

6 899 2 32 1 2 1070 0518 2

6 900 2 16 1 2 1100 0568 2

6 901 1 32 1 1 1283 0518 1

6 902 1 8 1 1 1345 0468 1

6 903 2 32 1 2 973 0568 2

6 904 2 8 1 2 893 0568 2

6 905 2 32 1 2 1189 0618 2

6 906 1 32 1 1 1284 0668 1

6 907 2 8 1 2 975 0518 1

6 908 1 16 1 1 1093 0668 1

6 909 1 16 1 1 2079 0568 1

6 910 1 16 1 1 2139 0468 1

6 911 2 32 1 2 1260 0468 2

6 912 1 32 1 1 824 0568 1

6 913 1 8 1 1 842 0668 2

6 914 1 8 1 1 1043 0518 2

6 915 1 8 1 1 1095 0618 1

6 916 1 32 1 1 756 0668 1

6 917 1 32 1 1 2641 0518 1

6 918 2 32 1 2 1032 0568 2

6 919 1 32 1 1 859 0668 1

6 920 2 32 1 2 786 0518 2

6 921 1 8 1 1 753 0568 2

6 922 1 16 1 1 1110 0518 1

6 923 2 8 1 2 788 0618 2

6 924 1 32 1 1 756 0568 1

6 925 2 32 1 2 795 0468 2

6 926 1 16 1 1 900 0568 1

6 927 1 8 1 1 1037 0518 1

6 928 1 8 1 1 817 0568 2

6 929 2 32 1 2 867 0568 2

6 930 1 16 1 1 910 0518 1

6 931 2 8 1 2 653 0668 1

6 932 2 8 1 2 894 0618 2

6 933 1 16 1 1 2026 0618 1

6 934 1 16 1 1 1027 0668 1

6 935 2 32 1 2 821 0668 2

6 936 1 16 1 1 1002 0468 1

6 937 2 16 1 2 729 0668 2

6 938 2 16 0 2 4400 0468 2

6 939 2 32 1 2 1083 0618 2

6 940 1 16 1 1 1410 0568 1

6 941 2 32 1 2 837 0518 2

6 942 1 16 0 1 2085 0618 1

6 943 2 16 1 2 1727 0618 2

6 944 2 16 1 2 2184 0568 2

6 945 1 32 1 1 915 0668 1

6 946 2 16 1 2 1767 0468 2

6 947 2 32 1 2 1073 0668 2

6 948 2 8 1 2 790 0668 2

6 949 1 16 1 1 1681 0668 1

6 950 1 8 1 1 1926 0618 1

6 951 1 16 1 1 1596 0518 1

6 952 2 32 1 2 1809 0468 2

6 953 2 8 1 2 739 0568 2

6 954 1 8 1 1 918 0568 2

6 955 2 8 1 2 761 0618 2

6 956 2 8 1 2 2100 0468 2

6 957 1 8 1 1 1328 0518 2

6 958 2 32 1 2 1370 0468 2

6 959 2 16 1 2 1422 0618 2

6 960 2 16 1 2 707 0668 2

6 961 1 16 1 1 1169 0468 1

6 962 2 8 1 2 773 0668 1

6 963 2 16 1 2 2530 0568 2

6 964 1 8 1 1 885 0668 1

6 965 2 8 1 2 984 0518 1

6 966 2 32 1 2 1066 0518 2

6 967 2 8 1 2 831 0618 2

6 968 1 32 1 1 1239 0568 1

6 969 2 16 1 2 979 0618 2

6 970 1 32 1 1 1002 0568 1

6 971 1 32 1 1 992 0468 1

6 972 2 16 1 2 974 0518 2

6 973 1 16 1 1 2594 0618 1

6 974 1 16 1 1 1240 0568 1

6 975 2 16 1 2 4680 0468 2

6 976 1 16 1 1 1875 0518 1

6 977 1 16 1 1 1378 0618 1

6 978 1 32 1 1 858 0668 1

6 979 2 32 1 2 953 0618 2

6 980 2 32 1 2 867 0518 2

6 981 2 16 1 2 890 0668 2

6 982 1 16 1 1 1188 0568 1

6 983 1 16 1 1 1271 0468 1

6 984 2 32 1 2 817 0568 2

6 985 2 16 1 2 1106 0618 2

6 986 1 8 1 1 722 0518 2

6 987 1 32 1 1 1614 0518 1

6 988 1 16 1 1 1611 0518 1

6 989 1 8 1 1 1017 0518 2

6 990 1 8 1 1 823 0618 1

6 991 2 8 1 2 742 0618 2

6 992 1 32 1 1 906 0518 1

6 993 2 32 1 2 1238 0468 2

6 994 2 16 1 2 779 0668 2

6 995 2 16 0 2 1366 0518 2

6 996 2 8 1 2 1847 0518 1

6 997 1 32 1 1 846 0568 1

6 998 1 8 0 1 1811 0468 1

6 999 2 32 1 2 2156 0668 2

6 1000 2 32 1 2 997 0568 2

6 1001 1 32 1 1 782 0668 1

6 1002 2 8 1 2 933 0618 2

6 1003 2 16 1 2 1249 0568 2

6 1004 1 8 1 1 706 0668 2

6 1005 1 16 1 1 2077 0668 1

6 1006 1 16 1 1 1258 0568 1

6 1007 2 32 1 2 1283 0518 2

6 1008 2 8 1 2 903 0518 1

6 1009 2 32 1 2 1362 0468 2

6 1010 2 32 1 2 942 0518 2

6 1011 2 16 1 2 1504 0518 2

6 1012 1 32 1 1 1121 0618 1

6 1013 2 8 1 2 976 0568 2

6 1014 2 16 1 2 1951 0468 2

6 1015 2 32 1 2 1027 0668 2

6 1016 2 8 1 2 763 0668 2

6 1017 1 32 1 1 2260 0468 1

6 1018 2 8 1 2 1067 0468 2

6 1019 1 8 1 1 895 0668 1

6 1020 2 16 1 2 1682 0518 2

6 1021 1 8 1 1 814 0568 2

6 1022 2 8 1 2 955 0668 2

6 1023 1 32 1 1 970 0618 1

6 1024 2 32 1 2 915 0618 2

6 1025 2 8 1 2 808 0468 2

6 1026 1 8 1 1 1179 0518 1

6 1027 1 8 1 1 843 0668 1

6 1028 1 32 1 1 1067 0518 1

6 1029 1 32 1 1 717 0518 1

6 1030 2 32 1 2 839 0668 2

6 1031 1 8 1 1 804 0518 1

6 1032 1 32 1 1 774 0668 1

6 1033 1 16 1 1 789 0668 1

6 1034 1 32 1 1 1388 0468 1

6 1035 1 32 1 1 1058 0518 1

6 1036 2 8 1 2 917 0518 2

6 1037 2 16 1 2 1143 0568 2

6 1038 2 32 1 2 816 0568 2

6 1039 1 32 1 1 1048 0468 1

6 1040 2 16 1 2 922 0518 2

6 1041 1 8 1 1 913 0668 1

6 1042 2 16 1 2 1216 0518 2

6 1043 1 8 1 1 802 0668 2

6 1044 1 8 1 1 1413 0568 1

6 1045 2 32 1 2 1001 0668 2

6 1046 2 16 1 2 853 0568 2

6 1047 2 8 1 2 976 0468 2

6 1048 1 16 0 1 1074 0468 1

6 1049 1 16 1 1 2421 0618 1

6 1050 2 16 1 2 826 0668 2

6 1051 1 8 1 1 976 0618 1

6 1052 1 8 0 1 1332 0468 1

6 1053 1 32 1 1 1193 0668 1

6 1054 2 16 1 2 1044 0568 2

6 1055 2 16 1 2 1837 0468 2

6 1056 2 16 1 2 1124 0568 2

6 1057 2 32 1 2 1151 0518 2

6 1058 2 16 1 2 1090 0668 2

6 1059 1 16 1 1 2000 0468 1

6 1060 2 8 1 2 777 0568 2

6 1061 1 16 1 1 1022 0668 1

6 1062 2 8 1 2 1717 0618 2

6 1063 1 32 1 1 757 0568 1

6 1064 2 8 1 2 1250 0568 1

6 1065 2 8 1 2 868 0668 1

6 1066 1 16 1 1 985 0618 1

6 1067 2 32 1 2 984 0618 2

6 1068 1 32 1 1 788 0618 1

6 1069 2 32 1 2 1441 0468 2

6 1070 1 16 1 1 3600 0468 1

6 1071 1 16 1 1 1144 0568 1

6 1072 1 16 1 1 1549 0618 1

6 1073 2 8 1 2 922 0518 2

6 1074 2 32 1 2 1003 0518 2

6 1075 2 8 1 2 707 0568 2

6 1076 1 8 1 1 1606 0468 1

6 1077 2 32 1 2 1116 0468 2

6 1078 2 8 1 2 1847 0468 2

6 1079 1 16 1 1 1249 0518 1

6 1080 1 8 1 1 1296 0618 1

6 1081 1 8 1 1 859 0568 2

6 1082 1 16 1 1 963 0668 1

6 1083 2 8 1 2 919 0518 2

6 1084 1 16 1 1 2654 0518 1

6 1085 1 8 1 1 1038 0518 1

6 1086 2 8 1 2 730 0618 2

6 1087 2 8 1 2 705 0668 2

6 1088 2 16 0 2 1827 0518 2

6 1089 2 32 1 2 1163 0668 2

6 1090 1 16 1 1 991 0668 1

6 1091 2 16 1 2 1991 0668 2

6 1092 1 8 1 1 921 0568 1

6 1093 1 32 1 1 1142 0468 1

6 1094 1 32 1 1 972 0468 1

6 1095 1 32 1 1 1116 0518 1

6 1096 1 32 1 1 825 0568 1

6 1097 1 16 1 1 1389 0518 1

6 1098 1 8 1 1 977 0618 1

6 1099 2 16 0 2 1207 0618 2

6 1100 1 32 1 1 1282 0568 1

6 1101 2 16 1 2 1122 0618 2

6 1102 2 32 1 2 1378 0518 2

6 1103 2 16 1 2 871 0668 2

6 1104 2 32 1 2 1614 0618 2

6 1105 2 32 1 2 817 0568 2

6 1106 1 16 1 1 2185 0568 1

6 1107 1 8 1 1 932 0568 1

6 1108 1 16 1 1 2162 0668 1

6 1109 2 8 1 2 923 0568 2

6 1110 2 8 1 2 3411 0468 2

6 1111 1 8 1 1 950 0618 1

6 1112 1 16 1 1 1112 0668 1

6 1113 2 8 1 2 782 0468 2

6 1114 2 32 1 2 1150 0468 2

6 1115 1 32 1 1 782 0618 1

6 1116 1 8 1 1 1182 0518 1

6 1117 2 8 1 2 727 0668 2

6 1118 2 32 1 2 2048 0618 2

6 1119 2 32 1 2 1113 0568 2

6 1120 1 32 1 1 1267 0568 1

6 1121 2 16 1 2 1247 0568 2

6 1122 1 8 1 1 1440 0468 1

6 1123 2 16 1 2 744 0668 2

6 1124 2 8 1 2 713 0618 2

6 1125 1 32 1 1 1613 0518 1

6 1126 1 16 1 1 969 0568 1

6 1127 1 16 1 1 1309 0618 1

6 1128 1 32 1 1 846 0668 1

6 1129 2 16 1 2 2520 0468 2

6 1130 2 8 1 2 780 0668 2

6 1131 1 32 1 1 941 0618 1

6 1132 1 8 1 1 1178 0618 1

6 1133 1 32 1 1 849 0668 1

6 1134 1 8 1 1 1038 0568 1

6 1135 1 16 0 1 1500 0518 1

6 1136 2 8 1 2 805 0618 2

6 1137 2 16 1 2 919 0668 2

6 1138 2 32 1 2 879 0618 2

6 1139 2 32 1 2 1151 0668 2

6 1140 1 16 1 1 981 0518 1

6 1141 1 16 1 1 1325 0568 1

6 1142 1 8 1 1 1016 0568 2

6 1143 2 32 1 2 1187 0668 2

6 1144 2 16 1 2 1575 0568 2

6 1145 1 8 1 1 791 0618 1

6 1146 1 8 1 1 976 0618 1

6 1147 1 16 1 1 791 0668 1

6 1148 1 32 1 1 845 0618 1

6 1149 2 32 1 2 1097 0568 2

6 1150 2 16 1 2 1455 0618 2

6 1151 2 32 1 2 1306 0568 2

6 1152 2 16 1 2 1821 0468 2

6 1153 2 8 1 2 1356 0468 2

6 1154 1 16 0 1 1441 0468 1

6 1155 1 16 1 1 1002 0518 1

6 1156 2 8 1 2 749 0618 2

6 1157 1 16 1 1 1368 0618 1

6 1158 2 16 1 2 1098 0518 2

6 1159 2 32 1 2 999 0668 2

6 1160 2 32 1 2 867 0618 2

6 1161 2 16 1 2 1069 0618 2

6 1162 1 8 1 1 731 0668 2

6 1163 2 32 1 2 826 0618 2

6 1164 1 8 1 1 1045 0518 2

6 1165 2 8 1 2 856 0518 1

6 1166 2 32 1 2 1119 0468 2

6 1167 2 16 1 2 1586 0468 2

6 1168 1 32 1 1 1069 0468 1

6 1169 2 32 1 2 1160 0468 2

6 1170 2 8 1 2 875 0468 2

6 1171 1 32 1 1 929 0668 1

6 1172 2 16 1 2 1243 0618 2

6 1173 1 16 0 1 853 0618 1

6 1174 1 32 1 1 798 0568 1

6 1175 1 32 1 1 1599 0468 1

6 1176 1 32 1 1 749 0668 1

6 1177 2 16 1 2 1701 0618 2

6 1178 1 16 1 1 1570 0568 1

6 1179 2 8 1 2 756 0668 2

6 1180 1 16 1 1 1126 0468 1

6 1181 2 8 1 2 771 0568 2

6 1182 2 8 1 2 1215 0518 2

6 1183 1 8 1 1 827 0568 2

6 1184 2 16 1 2 1369 0468 2

6 1185 1 8 1 1 1067 0468 1

6 1186 1 8 1 1 984 0668 2

6 1187 1 32 1 1 1002 0518 1

6 1188 2 8 1 2 660 0568 1

6 1189 2 32 1 2 1281 0518 2

6 1190 1 8 1 1 964 0468 1

6 1191 1 8 1 1 1296 0468 1

6 1192 1 8 1 1 1352 0468 1

6 1193 2 32 1 2 1046 0468 2

6 1194 2 16 1 2 1322 0468 2

6 1195 2 8 1 2 1314 0568 2

6 1196 1 32 1 1 868 0618 1

6 1197 1 16 1 1 1132 0468 1

6 1198 1 8 1 1 871 0518 2

6 1199 2 32 1 2 1121 0568 2

6 1200 1 32 1 1 805 0618 1

7 1 1 8 1 1 943 0518 2

7 2 1 32 1 1 1823 0668 1

7 3 1 8 1 1 815 0668 1

7 4 2 16 1 2 1674 0668 2

7 5 2 8 1 2 901 0668 2

7 6 1 32 1 1 1821 0518 1

7 7 1 16 1 1 2324 0518 1

7 8 2 32 1 2 1883 0668 2

7 9 1 8 1 1 1822 0468 1

7 10 1 8 1 1 745 0568 1

7 11 2 16 1 2 1616 0518 2

7 12 1 32 1 1 1633 0668 1

7 13 1 16 1 1 1182 0668 1

7 14 1 8 1 1 757 0668 2

7 15 1 32 1 1 1478 0468 1

7 16 2 32 1 2 1102 0618 2

7 17 1 16 1 1 1468 0518 1

7 18 1 32 1 1 1477 0668 1

7 19 2 16 1 2 1548 0618 2

7 20 1 16 0 1 2391 0468 1

7 21 2 16 1 2 2145 0468 2

7 22 2 8 1 2 1751 0518 1

7 23 1 16 1 1 1789 0668 1

7 24 1 8 1 1 2445 0618 1

7 25 2 8 1 2 938 0668 2

7 26 2 16 1 2 2394 0668 2

7 27 1 16 1 1 1400 0468 1

7 28 2 8 1 2 1098 0568 2

7 29 2 16 1 2 1928 0568 2

7 30 1 8 1 1 1318 0518 1

7 31 1 32 1 1 2304 0468 1

7 32 1 32 1 1 1610 0568 1

7 33 2 32 1 2 2126 0518 2

7 34 1 8 1 1 1947 0468 1

7 35 1 8 1 1 867 0568 2

7 36 1 8 1 1 1227 0468 1

7 37 1 8 1 1 711 0468 1

7 38 2 8 1 2 1281 0468 2

7 39 2 32 1 2 2634 0518 2

7 40 2 8 1 2 1190 0518 1

7 41 1 16 1 1 2557 0618 1

7 42 1 8 1 1 770 0568 2

7 43 2 32 1 2 2343 0618 2

7 44 2 16 1 2 1364 0568 2

7 45 2 8 1 2 638 0668 1

7 46 2 16 1 2 1673 0668 2

7 47 2 8 1 2 787 0468 2

7 48 2 16 1 2 1325 0618 2

7 49 2 16 1 2 3048 0468 2

7 50 2 32 1 2 1332 0568 2

7 51 2 32 1 2 1896 0468 2

7 52 1 32 1 1 1445 0618 1

7 53 1 32 1 1 1234 0618 1

7 54 1 16 1 1 1138 0568 1

7 55 1 8 1 1 820 0668 2

7 56 2 32 1 2 1643 0468 2

7 57 1 16 1 1 1395 0618 1

7 58 1 32 1 1 1241 0618 1

7 59 2 16 1 2 1184 0668 2

7 60 2 32 1 2 1858 0518 2

7 61 1 16 1 1 936 0668 1

7 62 2 16 1 2 1071 0668 2

7 63 1 32 1 1 1092 0468 1

7 64 2 8 1 2 1402 0468 2

7 65 1 32 1 1 1464 0668 1

7 66 2 16 1 2 1011 0568 2

7 67 1 8 1 1 1135 0518 2

7 68 1 8 1 1 709 0468 1

7 69 1 16 1 1 959 0568 1

7 70 2 16 1 2 2228 0518 2

7 71 2 16 1 2 1529 0668 2

7 72 1 16 1 1 2004 0568 1

7 73 1 8 1 1 1830 0568 2

7 74 2 32 1 2 1622 0668 2

7 75 2 8 1 2 1365 0518 1

7 76 2 32 1 2 2430 0568 2

7 77 1 16 1 1 1063 0468 1

7 78 2 32 1 2 3208 0668 2

7 79 1 8 1 1 1192 0518 1

7 80 1 16 1 1 1633 0568 1

7 81 1 16 0 1 1481 0568 1

7 82 1 8 1 1 2130 0618 1

7 83 2 32 1 2 1113 0618 2

7 84 1 8 1 1 1775 0518 1

7 85 1 32 1 1 804 0568 1

7 86 1 16 1 1 1365 0618 1

7 87 2 32 1 2 1456 0518 2

7 88 2 16 1 2 2071 0468 2

7 89 2 8 1 2 824 0468 2

7 90 1 8 1 1 956 0568 1

7 91 2 16 1 2 1202 0568 2

7 92 2 16 1 2 1685 0618 2

7 93 1 16 1 1 1424 0668 1

7 94 1 16 1 1 2100 0518 1

7 95 1 8 1 1 758 0518 2

7 96 2 16 1 2 1322 0468 2

7 97 2 8 1 2 1251 0518 2

7 98 1 16 1 1 1357 0668 1

7 99 2 16 1 2 787 0668 2

7 100 2 16 1 2 1186 0568 2

7 101 2 32 1 2 1159 0668 2

7 102 2 32 1 2 1555 0618 2

7 103 1 32 1 1 859 0618 1

7 104 2 16 1 2 1128 0568 2

7 105 2 16 1 2 1646 0618 2

7 106 2 32 1 2 1413 0668 2

7 107 2 16 1 2 917 0668 2

7 108 2 16 1 2 945 0618 2

7 109 2 32 1 2 1121 0518 2

7 110 1 16 1 1 1723 0468 1

7 111 1 8 1 1 908 0668 1

7 112 2 32 1 2 1271 0568 2

7 113 2 32 1 2 1289 0568 2

7 114 2 16 1 2 999 0568 2

7 115 2 8 1 2 1783 0468 2

7 116 1 32 1 1 922 0568 1

7 117 2 32 1 2 1475 0468 2

7 118 2 32 1 2 1042 0568 2

7 119 1 8 1 1 2064 0468 1

7 120 2 16 1 2 1582 0618 2

7 121 2 8 0 2 1086 0568 2

7 122 2 8 1 2 1376 0568 1

7 123 2 16 1 2 1216 0518 2

7 124 1 32 1 1 1216 0518 1

7 125 2 32 1 2 1201 0618 2

7 126 1 16 1 1 2223 0518 1

7 127 1 16 1 1 876 0468 1

7 128 1 8 1 1 1126 0618 1

7 129 1 32 1 1 1097 0468 1

7 130 1 32 1 1 1037 0518 1

7 131 1 16 1 1 1011 0468 1

7 132 1 16 1 1 1189 0518 1

7 133 1 8 1 1 1180 0618 1

7 134 1 16 1 1 1248 0668 1

7 135 2 16 1 2 1969 0568 2

7 136 2 8 1 2 999 0618 2

7 137 2 8 1 2 903 0668 2

7 138 1 32 1 1 1232 0668 1

7 139 1 8 1 1 776 0568 1

7 140 1 16 1 1 800 0668 1

7 141 1 16 1 1 1100 0518 1

7 142 2 8 1 2 943 0568 2

7 143 2 32 0 2 952 0668 2

7 144 1 32 1 1 1223 0518 1

7 145 2 8 1 2 1256 0668 2

7 146 1 8 1 1 959 0568 2

7 147 1 8 1 1 885 0618 1

7 148 1 16 1 1 978 0568 1

7 149 2 32 1 2 1407 0468 2

7 150 2 8 1 2 1095 0518 2

7 151 1 32 1 1 964 0668 1

7 152 2 8 1 2 1290 0518 2

7 153 2 16 1 2 1041 0618 2

7 154 2 16 1 2 1101 0518 2

7 155 2 8 1 2 797 0518 1

7 156 2 32 1 2 912 0618 2

7 157 1 16 1 1 1265 0468 1

7 158 1 16 1 1 1081 0618 1

7 159 1 16 1 1 893 0568 1

7 160 1 32 1 1 752 0568 1

7 161 1 32 1 1 771 0668 1

7 162 2 8 1 2 963 0618 2

7 163 2 32 1 2 1016 0618 2

7 164 2 32 1 2 1309 0468 2

7 165 1 32 1 1 730 0568 1

7 166 2 32 1 2 1324 0518 2

7 167 2 16 1 2 1298 0468 2

7 168 2 32 1 2 951 0468 2

7 169 1 8 1 1 986 0618 1

7 170 2 16 1 2 1944 0468 2

7 171 2 8 1 2 1188 0568 2

7 172 1 8 1 1 764 0668 2

7 173 1 16 1 1 940 0468 1

7 174 2 8 1 2 1195 0468 2

7 175 2 8 1 2 850 0618 2

7 176 2 8 1 2 656 0618 2

7 177 1 16 1 1 1256 0618 1

7 178 2 8 1 2 781 0468 2

7 179 1 8 1 1 1295 0468 1

7 180 2 32 1 2 1082 0468 2

7 181 1 32 1 1 686 0568 1

7 182 2 32 1 2 892 0668 2

7 183 2 8 1 2 816 0568 1

7 184 2 8 1 2 694 0568 2

7 185 1 16 1 1 1296 0518 1

7 186 2 8 1 2 897 0518 2

7 187 1 32 1 1 855 0568 1

7 188 1 16 0 1 719 0518 1

7 189 2 32 1 2 1267 0518 2

7 190 1 32 1 1 888 0468 1

7 191 1 32 1 1 949 0618 1

7 192 2 16 1 2 1195 0518 2

7 193 1 16 1 1 844 0668 1

7 194 2 8 1 2 726 0618 2

7 195 2 32 1 2 822 0568 2

7 196 1 32 1 1 1120 0468 1

7 197 2 16 1 2 1488 0618 2

7 198 2 16 1 2 1280 0518 2

7 199 2 32 1 2 1001 0568 2

7 200 1 8 1 1 2490 0618 1

7 201 1 32 0 1 738 0518 1

7 202 2 32 1 2 1285 0568 2

7 203 2 8 1 2 1125 0668 1

7 204 1 16 1 1 1235 0618 1

7 205 1 32 1 1 701 0618 1

7 206 1 32 1 1 1116 0668 1

7 207 1 8 1 1 641 0668 1

7 208 1 8 1 1 928 0468 1

7 209 1 32 1 1 956 0468 1

7 210 2 16 1 2 1069 0468 2

7 211 1 8 1 1 1202 0518 2

7 212 2 32 1 2 1743 0618 2

7 213 1 32 1 1 912 0518 1

7 214 1 32 1 1 1173 0618 1

7 215 1 32 1 1 855 0518 1

7 216 2 8 1 2 857 0618 2

7 217 1 8 1 1 921 0518 1

7 218 1 8 1 1 595 0618 1

7 219 1 32 1 1 868 0518 1

7 220 1 8 1 1 644 0668 1

7 221 1 8 1 1 458 0668 2

7 222 2 8 1 2 836 0668 2

7 223 1 8 1 1 1052 0568 1

7 224 1 32 1 1 785 0618 1

7 225 2 16 1 2 1461 0468 2

7 226 2 32 0 2 1240 0668 2

7 227 2 8 1 2 910 0568 2

7 228 1 32 1 1 963 0468 1

7 229 2 8 1 2 783 0618 2

7 230 2 16 0 2 1161 0518 2

7 231 1 16 1 1 1463 0618 1

7 232 2 8 1 2 922 0668 2

7 233 2 8 1 2 1808 0468 2

7 234 2 32 1 2 1278 0468 2

7 235 1 16 1 1 1264 0568 1

7 236 1 32 1 1 1228 0568 1

7 237 2 16 1 2 1240 0518 2

7 238 2 32 1 2 1553 0518 2

7 239 2 8 1 2 906 0618 2

7 240 1 16 1 1 1105 0618 1

7 241 2 8 1 2 897 0568 2

7 242 2 8 1 2 688 0668 2

7 243 2 32 1 2 855 0618 2

7 244 2 32 1 2 1582 0468 2

7 245 1 16 1 1 954 0668 1

7 246 2 16 1 2 1570 0468 2

7 247 1 16 1 1 1491 0618 1

7 248 2 8 1 2 1914 0468 2

7 249 1 32 1 1 866 0618 1

7 250 1 8 1 1 928 0518 1

7 251 2 32 1 2 997 0568 2

7 252 2 32 1 2 1038 0568 2

7 253 2 8 1 2 1109 0518 2

7 254 2 16 1 2 1984 0468 2

7 255 2 8 1 2 738 0668 2

7 256 1 16 1 1 1954 0468 1

7 257 1 8 1 1 903 0618 1

7 258 2 16 1 2 2405 0518 2

7 259 1 32 1 1 1603 0468 1

7 260 2 16 1 2 1283 0568 2

7 261 1 8 1 1 1793 0468 1

7 262 1 8 1 1 697 0568 1

7 263 1 32 1 1 753 0468 1

7 264 2 8 1 2 794 0618 2

7 265 2 8 1 2 883 0518 1

7 266 1 16 0 1 698 0668 1

7 267 2 16 1 2 1029 0568 2

7 268 1 32 1 1 1330 0668 1

7 269 2 32 1 2 783 0618 2

7 270 2 16 1 2 1235 0518 2

7 271 1 16 1 1 1545 0518 1

7 272 1 32 1 1 1207 0618 1

7 273 1 32 1 1 975 0468 1

7 274 1 16 1 1 1728 0618 1

7 275 2 16 1 2 1214 0618 2

7 276 2 16 1 2 953 0668 2

7 277 2 16 1 2 823 0668 2

7 278 1 8 1 1 878 0568 2

7 279 1 8 0 1 914 0568 2

7 280 1 32 1 1 1036 0468 1

7 281 1 32 1 1 936 0618 1

7 282 1 8 1 1 1078 0468 1

7 283 1 32 1 1 617 0668 1

7 284 1 8 1 1 1019 0568 2

7 285 2 32 1 2 818 0668 2

7 286 1 8 1 1 762 0668 1

7 287 2 32 1 2 1160 0518 2

7 288 1 8 1 1 1001 0518 2

7 289 2 32 1 2 1107 0668 2

7 290 1 16 1 1 1391 0618 1

7 291 1 8 1 1 865 0468 1

7 292 1 32 1 1 602 0568 1

7 293 2 16 0 2 857 0618 2

7 294 2 32 0 2 923 0518 2

7 295 1 16 1 1 852 0568 1

7 296 2 16 1 2 738 0568 2

7 297 1 32 1 1 1600 0518 1

7 298 1 32 1 1 1064 0668 1

7 299 1 16 1 1 1493 0618 1

7 300 2 16 1 2 1030 0518 2

7 301 2 16 1 2 1215 0568 2

7 302 2 8 1 2 945 0518 2

7 303 1 16 1 1 1622 0468 1

7 304 2 8 1 2 1218 0618 2

7 305 2 8 1 2 705 0568 1

7 306 1 32 1 1 1103 0668 1

7 307 1 8 1 1 1155 0468 1

7 308 2 8 1 2 1079 0668 2

7 309 1 16 1 1 1535 0568 1

7 310 2 16 0 2 1531 0468 2

7 311 2 16 1 2 1709 0618 2

7 312 2 32 1 2 1503 0468 2

7 313 2 32 0 2 1297 0518 2

7 314 2 16 0 2 1237 0518 2

7 315 2 16 1 2 1030 0618 2

7 316 2 16 1 2 1091 0468 2

7 317 2 32 1 2 1309 0468 2

7 318 2 8 1 2 874 0618 2

7 319 2 16 1 2 2545 0468 2

7 320 2 16 1 2 667 0668 2

7 321 1 16 1 1 1531 0468 1

7 322 1 16 1 1 760 0518 1

7 323 1 8 1 1 581 0568 1

7 324 1 32 1 1 964 0518 1

7 325 1 8 1 1 604 0568 1

7 326 2 32 1 2 954 0618 2

7 327 1 16 1 1 1060 0618 1

7 328 1 16 0 1 804 0618 1

7 329 1 16 1 1 1060 0568 1

7 330 2 32 1 2 1405 0468 2

7 331 2 32 1 2 929 0618 2

7 332 2 8 1 2 1005 0618 2

7 333 2 16 1 2 994 0568 2

7 334 1 32 1 1 1721 0668 1

7 335 1 16 1 1 1610 0468 1

7 336 2 8 1 2 878 0468 2

7 337 1 32 1 1 1095 0568 1

7 338 2 32 1 2 1012 0618 2

7 339 2 32 1 2 1144 0468 2

7 340 2 16 1 2 1001 0518 2

7 341 2 32 1 2 639 0568 2

7 342 1 16 1 1 1328 0468 1

7 343 1 32 1 1 699 0618 1

7 344 1 32 1 1 848 0568 1

7 345 2 16 1 2 772 0668 2

7 346 2 16 1 2 861 0518 2

7 347 1 8 1 1 1102 0518 1

7 348 1 16 1 1 637 0668 1

7 349 1 32 1 1 701 0668 1

7 350 2 16 0 2 579 0668 2

7 351 1 8 1 1 1028 0618 1

7 352 2 32 1 2 984 0668 2

7 353 2 32 1 2 1200 0468 2

7 354 2 8 1 2 1312 0518 1

7 355 2 8 1 2 2235 0468 2

7 356 1 8 1 1 856 0618 1

7 357 2 8 1 2 948 0668 1

7 358 2 8 1 2 881 0468 2

7 359 1 8 1 1 931 0618 1

7 360 1 8 1 1 1002 0668 1

7 361 2 32 1 2 1148 0618 2

7 362 1 32 1 1 694 0618 1

7 363 2 8 1 2 755 0568 2

7 364 2 32 1 2 958 0668 2

7 365 2 8 1 2 831 0668 2

7 366 2 32 1 2 1396 0518 2

7 367 2 32 1 2 1129 0518 2

7 368 2 16 1 2 723 0668 2

7 369 1 32 1 1 933 0518 1

7 370 2 8 1 2 759 0568 2

7 371 2 8 1 2 768 0518 1

7 372 1 16 1 1 755 0568 1

7 373 2 32 1 2 942 0568 2

7 374 1 8 1 1 1161 0518 2

7 375 1 32 1 1 781 0568 1

7 376 1 32 1 1 786 0468 1

7 377 2 32 1 2 1127 0518 2

7 378 1 32 1 1 1251 0518 1

7 379 2 8 1 2 848 0568 2

7 380 1 16 1 1 1113 0668 1

7 381 1 32 1 1 630 0618 1

7 382 2 16 1 2 1258 0618 2

7 383 1 32 1 1 1008 0518 1

7 384 2 16 1 2 1286 0568 2

7 385 1 16 1 1 749 0668 1

7 386 2 32 1 2 820 0618 2

7 387 2 32 1 2 1036 0468 2

7 388 2 8 1 2 917 0518 2

7 389 1 8 1 1 857 0468 1

7 390 2 8 1 2 666 0468 2

7 391 1 32 1 1 1197 0568 1

7 392 1 8 0 1 678 0668 2

7 393 2 8 1 2 708 0568 1

7 394 2 32 1 2 1030 0668 2

7 395 1 32 1 1 813 0468 1

7 396 2 32 1 2 900 0568 2

7 397 1 8 1 1 771 0668 2

7 398 2 32 1 2 789 0518 2

7 399 1 32 1 1 848 0468 1

7 400 2 32 1 2 646 0568 2

7 401 2 16 1 2 1154 0468 2

7 402 1 32 1 1 605 0668 1

7 403 2 8 1 2 933 0568 2

7 404 2 16 1 2 1013 0618 2

7 405 2 32 1 2 944 0568 2

7 406 1 8 0 1 814 0618 1

7 407 1 32 1 1 802 0568 1

7 408 2 8 1 2 722 0618 2

7 409 2 16 1 2 912 0568 2

7 410 2 8 1 2 1322 0468 2

7 411 1 8 1 1 795 0668 2

7 412 2 32 1 2 960 0518 2

7 413 2 8 1 2 613 0618 2

7 414 1 8 1 1 774 0668 1

7 415 1 32 1 1 865 0568 1

7 416 2 32 1 2 1374 0668 2

7 417 2 8 1 2 705 0518 1

7 418 1 16 1 1 771 0568 1

7 419 2 8 1 2 790 0468 2

7 420 2 16 0 2 974 0518 2

7 421 2 16 1 2 930 0518 2

7 422 1 16 0 1 959 0468 1

7 423 1 16 1 1 1264 0568 1

7 424 1 8 1 1 852 0518 1

7 425 2 16 1 2 1586 0618 2

7 426 2 8 1 2 959 0568 2

7 427 2 32 1 2 1567 0668 2

7 428 1 8 1 1 880 0618 1

7 429 2 32 1 2 876 0668 2

7 430 1 8 0 1 557 0618 1

7 431 1 32 1 1 1057 0468 1

7 432 2 8 1 2 774 0618 2

7 433 2 16 0 2 1699 0468 2

7 434 2 16 1 2 1138 0618 2

7 435 1 16 0 1 1907 0668 1

7 436 2 16 1 2 1699 0568 2

7 437 1 16 1 1 819 0668 1

7 438 1 16 1 1 1033 0668 1

7 439 1 8 1 1 770 0668 1

7 440 2 32 1 2 1033 0468 2

7 441 2 8 1 2 762 0468 2

7 442 2 8 1 2 601 0668 2

7 443 1 16 1 1 2946 0618 1

7 444 2 8 1 2 653 0668 2

7 445 1 16 1 1 1160 0568 1

7 446 1 32 1 1 830 0518 1

7 447 2 32 0 2 808 0568 2

7 448 2 16 1 2 900 0668 2

7 449 1 32 1 1 989 0618 1

7 450 1 8 1 1 947 0668 2

7 451 1 16 1 1 1177 0568 1

7 452 1 16 1 1 930 0518 1

7 453 1 8 0 1 813 0468 1

7 454 1 32 1 1 663 0568 1

7 455 1 8 1 1 690 0468 1

7 456 2 8 1 2 757 0518 2

7 457 2 8 1 2 742 0618 2

7 458 1 8 1 1 773 0568 2

7 459 1 16 0 1 2099 0468 1

7 460 1 8 1 1 927 0568 1

7 461 2 16 1 2 897 0668 2

7 462 1 32 1 1 1358 0668 1

7 463 1 8 1 1 1675 0468 1

7 464 2 32 1 2 925 0618 2

7 465 1 32 1 1 785 0518 1

7 466 1 16 1 1 1283 0518 1

7 467 1 8 1 1 868 0618 1

7 468 2 8 1 2 713 0668 1

7 469 2 16 1 2 1846 0468 2

7 470 1 8 1 1 774 0518 1

7 471 1 16 1 1 2257 0468 1

7 472 1 8 1 1 783 0518 2

7 473 1 32 1 1 861 0618 1

7 474 1 16 1 1 1101 0618 1

7 475 1 16 1 1 946 0518 1

7 476 1 16 1 1 767 0518 1

7 477 1 16 1 1 1233 0518 1

7 478 1 32 1 1 650 0518 1

7 479 1 16 1 1 717 0518 1

7 480 1 8 1 1 1158 0518 2

7 481 1 16 0 1 1353 0468 1

7 482 1 8 1 1 881 0568 2

7 483 2 32 1 2 1171 0668 2

7 484 1 16 1 1 1102 0518 1

7 485 1 8 1 1 688 0518 1

7 486 1 8 1 1 773 0618 1

7 487 1 8 1 1 560 0668 1

7 488 1 8 1 1 1120 0618 1

7 489 2 16 0 2 978 0468 2

7 490 1 32 1 1 758 0568 1

7 491 2 16 1 2 1813 0468 2

7 492 2 16 1 2 752 0668 2

7 493 2 32 1 2 1042 0468 2

7 494 1 32 1 1 770 0568 1

7 495 1 8 1 1 913 0468 1

7 496 1 32 1 1 619 0618 1

7 497 1 32 1 1 591 0618 1

7 498 2 32 1 2 1289 0518 2

7 499 1 32 1 1 1180 0568 1

7 500 2 32 1 2 777 0618 2

7 501 1 8 1 1 898 0468 1

7 502 1 8 1 1 645 0518 2

7 503 1 8 1 1 722 0468 1

7 504 2 32 1 2 1330 0468 2

7 505 1 32 1 1 895 0668 1

7 506 1 16 1 1 1742 0518 1

7 507 2 16 1 2 921 0668 2

7 508 2 8 0 2 741 0468 2

7 509 2 8 1 2 991 0518 1

7 510 1 16 1 1 1047 0668 1

7 511 2 8 1 2 730 0668 1

7 512 1 16 1 1 913 0568 1

7 513 1 16 1 1 971 0618 1

7 514 2 8 1 2 917 0618 2

7 515 2 16 1 2 1006 0568 2

7 516 2 32 1 2 1035 0618 2

7 517 2 16 1 2 1023 0618 2

7 518 2 8 1 2 790 0618 2

7 519 2 32 1 2 911 0568 2

7 520 1 8 1 1 753 0618 1

7 521 1 8 0 1 672 0468 1

7 522 1 8 1 1 957 0568 1

7 523 2 32 1 2 1424 0668 2

7 524 1 16 1 1 981 0568 1

7 525 1 32 1 1 981 0668 1

7 526 1 32 1 1 688 0668 1

7 527 1 16 1 1 679 0668 1

7 528 1 8 1 1 953 0668 2

7 529 1 16 1 1 1253 0468 1

7 530 1 16 1 1 950 0618 1

7 531 1 8 1 1 823 0668 2

7 532 1 16 1 1 633 0668 1

7 533 1 8 1 1 636 0668 2

7 534 2 8 1 2 1698 0518 2

7 535 2 32 1 2 4442 0668 2

7 536 2 8 1 2 644 0568 2

7 537 2 8 1 2 727 0618 2

7 538 2 32 1 2 872 0568 2

7 539 1 8 1 1 1078 0468 1

7 540 2 32 1 2 1047 0568 2

7 541 1 16 1 1 766 0568 1

7 542 1 32 1 1 852 0668 1

7 543 1 32 1 1 707 0618 1

7 544 2 32 1 2 871 0468 2

7 545 2 16 1 2 2059 0618 2

7 546 2 8 1 2 1054 0468 2

7 547 1 8 1 1 831 0518 2

7 548 2 16 0 2 953 0518 2

7 549 1 32 1 1 897 0518 1

7 550 2 8 1 2 723 0568 1

7 551 1 32 1 1 1379 0468 1

7 552 2 8 0 2 554 0518 1

7 553 2 32 1 2 1072 0518 2

7 554 1 32 1 1 738 0618 1

7 555 2 16 1 2 932 0468 2

7 556 1 8 1 1 723 0568 1

7 557 1 32 1 1 945 0668 1

7 558 1 8 1 1 678 0568 1

7 559 2 16 1 2 983 0518 2

7 560 2 32 0 2 881 0518 2

7 561 2 32 1 2 1113 0668 2

7 562 1 16 1 1 1456 0518 1

7 563 1 16 1 1 1169 0618 1

7 564 2 8 1 2 933 0518 1

7 565 2 32 1 2 843 0568 2

7 566 1 8 1 1 867 0568 2

7 567 1 32 0 1 677 0568 1

7 568 2 16 0 2 1033 0568 2

7 569 1 32 1 1 1247 0468 1

7 570 1 8 1 1 729 0668 1

7 571 2 8 1 2 943 0468 2

7 572 2 8 1 2 840 0568 1

7 573 2 32 1 2 1044 0668 2

7 574 1 32 1 1 775 0618 1

7 575 1 16 1 1 1169 0568 1

7 576 2 16 1 2 1879 0618 2

7 577 1 8 1 1 1315 0468 1

7 578 2 8 1 2 1171 0468 2

7 579 1 16 1 1 921 0668 1

7 580 1 16 1 1 676 0668 1

7 581 1 16 1 1 1278 0618 1

7 582 2 8 1 2 766 0668 2

7 583 1 8 0 1 810 0518 2

7 584 2 32 1 2 1095 0518 2

7 585 2 16 1 2 913 0568 2

7 586 2 8 1 2 758 0568 2

7 587 2 8 1 2 622 0668 2

7 588 2 32 1 2 1285 0668 2

7 589 1 16 1 1 1088 0568 1

7 590 2 32 1 2 1208 0468 2

7 591 2 32 1 2 957 0668 2

7 592 1 32 1 1 973 0468 1

7 593 1 32 1 1 902 0468 1

7 594 2 8 1 2 824 0518 2

7 595 1 16 1 1 1142 0518 1

7 596 1 8 1 1 770 0668 2

7 597 2 16 0 2 1009 0568 2

7 598 1 8 0 1 949 0518 1

7 599 2 32 1 2 1168 0468 2

7 600 1 32 1 1 794 0468 1

7 601 2 16 1 2 1111 0618 2

7 602 2 16 1 2 1354 0618 2

7 603 1 8 1 1 1114 0618 1

7 604 1 8 0 1 861 0618 1

7 605 2 32 1 2 1262 0518 2

7 606 2 8 1 2 626 0618 2

7 607 2 8 0 2 1331 0468 2

7 608 2 16 1 2 935 0568 2

7 609 2 32 1 2 980 0568 2

7 610 2 32 1 2 893 0568 2

7 611 2 16 1 2 1042 0468 2

7 612 2 32 1 2 897 0518 2

7 613 2 16 1 2 1010 0618 2

7 614 2 32 1 2 1036 0568 2

7 615 2 32 1 2 1338 0468 2

7 616 2 16 1 2 1230 0518 2

7 617 1 32 1 1 811 0518 1

7 618 2 8 1 2 1040 0568 2

7 619 2 16 1 2 1526 0518 2

7 620 2 16 1 2 767 0518 2

7 621 2 16 1 2 757 0668 2

7 622 2 16 1 2 886 0618 2

7 623 2 8 1 2 859 0668 2

7 624 2 16 1 2 968 0468 2

7 625 2 16 0 2 1604 0518 2

7 626 1 8 1 1 1989 0468 1

7 627 1 32 1 1 784 0568 1

7 628 1 32 1 1 660 0668 1

7 629 1 32 1 1 1028 0568 1

7 630 1 32 1 1 587 0518 1

7 631 1 32 1 1 783 0518 1

7 632 1 32 1 1 812 0518 1

7 633 2 16 1 2 1208 0668 2

7 634 1 32 1 1 1463 0518 1

7 635 1 8 1 1 1499 0468 1

7 636 2 16 1 2 904 0618 2

7 637 1 16 0 1 966 0468 1

7 638 1 16 1 1 1163 0468 1

7 639 2 32 1 2 1837 0468 2

7 640 1 8 1 1 2037 0618 1

7 641 2 16 1 2 716 0668 2

7 642 1 16 1 1 1442 0468 1

7 643 2 16 0 2 947 0568 2

7 644 1 16 1 1 2038 0468 1

7 645 1 32 1 1 1227 0468 1

7 646 2 8 1 2 745 0468 2

7 647 1 32 1 1 851 0568 1

7 648 1 16 1 1 958 0518 1

7 649 2 16 1 2 767 0668 2

7 650 2 8 1 2 918 0568 2

7 651 1 16 0 1 625 0518 1

7 652 1 32 1 1 753 0518 1

7 653 1 16 1 1 1306 0518 1

7 654 2 8 1 2 740 0668 1

7 655 2 32 1 2 904 0618 2

7 656 2 8 1 2 898 0518 2

7 657 1 8 1 1 960 0618 1

7 658 1 32 1 1 914 0618 1

7 659 2 8 1 2 993 0668 2

7 660 2 16 1 2 883 0518 2

7 661 1 16 1 1 801 0668 1

7 662 1 16 0 1 1415 0518 1

7 663 2 32 1 2 1130 0468 2

7 664 1 8 0 1 670 0568 2

7 665 2 16 1 2 944 0668 2

7 666 2 8 1 2 661 0668 2

7 667 1 32 0 1 893 0618 1

7 668 2 16 1 2 1110 0468 2

7 669 1 32 1 1 1044 0668 1

7 670 1 8 1 1 849 0518 1

7 671 2 32 1 2 1584 0518 2

7 672 2 16 1 2 973 0518 2

7 673 1 32 1 1 905 0568 1

7 674 1 16 1 1 1585 0468 1

7 675 2 8 1 2 696 0568 2

7 676 1 16 1 1 799 0568 1

7 677 2 8 1 2 1346 0568 2

7 678 2 32 1 2 846 0618 2

7 679 1 8 1 1 1121 0518 1

7 680 1 16 0 1 1102 0618 1

7 681 2 8 1 2 739 0618 2

7 682 1 8 1 1 740 0568 1

7 683 2 8 1 2 1109 0518 2

7 684 2 8 0 2 1105 0618 2

7 685 2 8 1 2 737 0668 2

7 686 1 16 1 1 1702 0468 1

7 687 2 32 1 2 883 0618 2

7 688 2 16 1 2 1127 0468 2

7 689 1 32 1 1 1597 0468 1

7 690 2 8 1 2 806 0518 1

7 691 2 8 1 2 663 0468 2

7 692 2 32 1 2 1358 0618 2

7 693 1 32 1 1 932 0468 1

7 694 1 16 1 1 2163 0618 1

7 695 2 8 1 2 945 0468 2

7 696 1 8 1 1 961 0568 2

7 697 1 8 1 1 1414 0518 2

7 698 2 32 1 2 1027 0568 2

7 699 1 16 1 1 853 0568 1

7 700 2 16 1 2 961 0668 2

7 701 1 8 1 1 713 0668 1

7 702 2 32 1 2 631 0618 2

7 703 1 32 1 1 705 0518 1

7 704 2 32 1 2 930 0618 2

7 705 2 16 1 2 1398 0568 2

7 706 1 16 1 1 685 0668 1

7 707 1 16 1 1 1598 0618 1

7 708 1 16 1 1 880 0618 1

7 709 1 16 1 1 772 0568 1

7 710 2 8 1 2 950 0618 2

7 711 1 8 1 1 871 0618 1

7 712 1 32 1 1 793 0618 1

7 713 1 8 1 1 629 0668 1

7 714 2 8 1 2 863 0618 2

7 715 2 32 1 2 863 0518 2

7 716 2 32 1 2 919 0668 2

7 717 2 16 1 2 857 0468 2

7 718 1 16 1 1 914 0668 1

7 719 1 32 1 1 916 0668 1

7 720 2 16 1 2 1214 0568 2

7 721 1 8 1 1 1571 0468 1

7 722 1 16 1 1 760 0668 1

7 723 1 16 1 1 979 0468 1

7 724 2 16 1 2 976 0468 2

7 725 2 8 1 2 836 0568 1

7 726 2 8 1 2 1310 0468 2

7 727 2 32 1 2 694 0618 2

7 728 1 16 1 1 853 0668 1

7 729 2 8 1 2 826 0618 2

7 730 1 16 1 1 1241 0568 1

7 731 2 8 1 2 904 0668 1

7 732 1 32 0 1 782 0518 1

7 733 2 32 1 2 1125 0468 2

7 734 2 8 1 2 792 0618 2

7 735 1 16 1 1 1760 0568 1

7 736 2 32 1 2 958 0518 2

7 737 1 8 1 1 1532 0618 1

7 738 1 32 1 1 775 0518 1

7 739 1 32 1 1 693 0668 1

7 740 1 32 1 1 746 0468 1

7 741 1 32 1 1 780 0618 1

7 742 1 32 1 1 631 0618 1

7 743 2 16 1 2 1174 0518 2

7 744 2 16 1 2 650 0668 2

7 745 1 16 0 1 842 0568 1

7 746 1 8 1 1 1130 0468 1

7 747 1 16 1 1 1259 0618 1

7 748 2 16 1 2 1397 0668 2

7 749 2 16 1 2 1285 0618 2

7 750 2 16 1 2 834 0568 2

7 751 2 32 1 2 1233 0518 2

7 752 2 8 1 2 739 0568 1

7 753 1 16 1 1 928 0518 1

7 754 2 16 1 2 1141 0568 2

7 755 2 8 1 2 823 0568 2

7 756 1 8 1 1 937 0468 1

7 757 1 32 1 1 847 0468 1

7 758 2 16 1 2 1245 0618 2

7 759 1 8 1 1 797 0618 1

7 760 2 32 1 2 928 0568 2

7 761 2 16 1 2 807 0668 2

7 762 2 16 1 2 1256 0518 2

7 763 2 8 0 2 1385 0468 2

7 764 1 8 0 1 802 0468 1

7 765 1 8 1 1 905 0568 2

7 766 1 32 1 1 986 0568 1

7 767 1 16 1 1 634 0668 1

7 768 1 16 1 1 1493 0468 1

7 769 1 16 1 1 846 0518 1

7 770 2 16 1 2 1073 0468 2

7 771 1 16 1 1 1367 0518 1

7 772 1 32 1 1 895 0518 1

7 773 1 8 1 1 575 0568 1

7 774 1 16 1 1 1302 0468 1

7 775 2 32 0 2 591 0568 2

7 776 2 32 1 2 1379 0518 2

7 777 2 32 1 2 1669 0668 2

7 778 2 8 1 2 810 0468 2

7 779 1 8 1 1 1072 0568 2

7 780 2 8 1 2 953 0568 2

7 781 2 8 1 2 908 0468 2

7 782 2 32 1 2 878 0618 2

7 783 2 32 1 2 674 0618 2

7 784 2 16 1 2 905 0618 2

7 785 1 32 1 1 1154 0468 1

7 786 1 8 1 1 1113 0668 1

7 787 2 32 1 2 994 0568 2

7 788 2 16 0 2 705 0468 2

7 789 2 32 1 2 1287 0468 2

7 790 1 32 1 1 803 0468 1

7 791 2 8 1 2 719 0618 2

7 792 1 16 1 1 1074 0618 1

7 793 2 8 1 2 732 0568 2

7 794 1 16 1 1 1257 0618 1

7 795 2 32 1 2 1176 0668 2

7 796 2 32 1 2 621 0518 2

7 797 1 32 1 1 815 0568 1

7 798 1 16 1 1 1004 0568 1

7 799 2 16 1 2 944 0468 2

7 800 1 16 1 1 735 0668 1

7 801 1 32 1 1 914 0668 1

7 802 2 8 1 2 778 0668 2

7 803 1 16 1 1 1251 0618 1

7 804 1 8 1 1 751 0518 1

7 805 1 16 1 1 597 0668 1

7 806 1 8 1 1 838 0618 1

7 807 2 16 1 2 1167 0618 2

7 808 2 8 1 2 914 0668 2

7 809 1 32 1 1 1164 0568 1

7 810 1 8 1 1 687 0668 2

7 811 2 32 0 2 631 0568 2

7 812 2 16 1 2 1331 0568 2

7 813 2 8 1 2 924 0518 1

7 814 2 16 1 2 1360 0518 2

7 815 2 16 1 2 871 0468 2

7 816 2 16 1 2 1024 0618 2

7 817 2 32 1 2 777 0618 2

7 818 1 8 1 1 1049 0668 2

7 819 2 32 1 2 1003 0468 2

7 820 2 16 1 2 997 0518 2

7 821 2 16 1 2 647 0668 2

7 822 2 8 1 2 862 0468 2

7 823 2 8 1 2 590 0618 2

7 824 2 32 1 2 1010 0468 2

7 825 2 8 1 2 552 0668 1

7 826 1 32 1 1 892 0568 1

7 827 1 8 1 1 692 0568 2

7 828 2 32 0 2 767 0468 2

7 829 1 32 1 1 944 0668 1

7 830 2 16 1 2 1544 0668 2

7 831 2 16 1 2 711 0668 2

7 832 1 8 1 1 1290 0518 2

7 833 1 8 1 1 828 0618 1

7 834 2 8 1 2 850 0618 2

7 835 1 32 1 1 1124 0468 1

7 836 1 32 1 1 729 0518 1

7 837 1 32 1 1 543 0618 1

7 838 1 16 1 1 1093 0518 1

7 839 2 8 0 2 615 0518 2

7 840 2 32 1 2 1263 0518 2

7 841 2 8 1 2 792 0668 2

7 842 2 32 1 2 859 0568 2

7 843 2 32 1 2 995 0668 2

7 844 1 32 1 1 734 0668 1

7 845 1 8 1 1 770 0518 2

7 846 2 8 1 2 967 0518 2

7 847 1 32 1 1 1200 0468 1

7 848 1 8 1 1 568 0668 1

7 849 2 32 1 2 856 0618 2

7 850 1 32 1 1 1470 0618 1

7 851 1 16 1 1 1212 0618 1

7 852 1 8 1 1 633 0518 1

7 853 1 16 1 1 686 0568 1

7 854 1 8 1 1 1235 0468 1

7 855 2 16 1 2 805 0668 2

7 856 2 32 0 2 859 0618 2

7 857 2 16 1 2 843 0518 2

7 858 2 16 1 2 986 0618 2

7 859 1 16 0 1 804 0468 1

7 860 2 16 1 2 1143 0518 2

7 861 2 8 1 2 686 0668 2

7 862 2 16 1 2 572 0668 2

7 863 1 8 1 1 1360 0468 1

7 864 1 16 0 1 1232 0618 1

7 865 1 8 1 1 803 0668 2

7 866 2 32 1 2 1232 0518 2

7 867 2 8 1 2 1001 0518 2

7 868 1 16 1 1 1068 0518 1

7 869 2 8 1 2 1154 0518 1

7 870 1 16 1 1 925 0568 1

7 871 1 8 0 1 895 0668 1

7 872 1 32 1 1 922 0668 1

7 873 2 16 1 2 1305 0618 2

7 874 1 16 1 1 1735 0518 1

7 875 2 8 1 2 807 0518 2

7 876 1 16 1 1 908 0518 1

7 877 1 16 1 1 1025 0468 1

7 878 1 16 1 1 770 0468 1

7 879 1 32 1 1 632 0568 1

7 880 1 32 1 1 618 0468 1

7 881 2 16 1 2 1098 0568 2

7 882 1 8 1 1 994 0568 1

7 883 1 8 0 1 1034 0518 1

7 884 2 16 1 2 1727 0468 2

7 885 1 16 1 1 840 0668 1

7 886 1 32 1 1 617 0568 1

7 887 1 16 1 1 883 0568 1

7 888 1 8 1 1 578 0568 1

7 889 1 32 1 1 691 0518 1

7 890 2 16 0 2 952 0468 2

7 891 1 32 1 1 655 0618 1

7 892 2 8 1 2 926 0518 1

7 893 1 32 1 1 702 0668 1

7 894 2 32 0 2 800 0668 2

7 895 2 32 0 2 1480 0468 2

7 896 2 32 1 2 1852 0468 2

7 897 2 16 1 2 1115 0518 2

7 898 2 8 1 2 753 0568 2

7 899 1 32 1 1 662 0618 1

7 900 2 8 1 2 957 0618 2

7 901 1 8 0 1 712 0468 1

7 902 2 32 1 2 1094 0668 2

7 903 2 16 1 2 676 0568 2

7 904 2 32 1 2 905 0618 2

7 905 2 32 1 2 701 0568 2

7 906 1 16 1 1 749 0668 1

7 907 1 8 1 1 984 0568 2

7 908 1 16 1 1 759 0668 1

7 909 2 8 1 2 1304 0468 2

7 910 2 8 1 2 642 0518 1

7 911 1 16 1 1 906 0468 1

7 912 1 32 1 1 744 0518 1

7 913 1 32 1 1 992 0518 1

7 914 2 32 1 2 928 0668 2

7 915 2 16 1 2 819 0518 2

7 916 1 32 1 1 806 0668 1

7 917 1 16 1 1 1416 0518 1

7 918 2 32 1 2 816 0668 2

7 919 2 8 1 2 1001 0568 2

7 920 2 8 1 2 596 0618 2

7 921 1 32 1 1 1139 0668 1

7 922 1 32 1 1 755 0468 1

7 923 2 8 1 2 621 0668 2

7 924 2 8 1 2 744 0668 2

7 925 2 32 1 2 780 0668 2

7 926 2 32 0 2 764 0518 2

7 927 1 8 1 1 1164 0668 1

7 928 1 8 1 1 1296 0468 1

7 929 2 8 1 2 977 0468 2

7 930 1 16 0 1 920 0468 1

7 931 1 32 1 1 1313 0618 1

7 932 2 16 0 2 1818 0468 2

7 933 1 16 1 1 1226 0618 1

7 934 1 16 1 1 1706 0618 1

7 935 2 16 1 2 1898 0568 2

7 936 2 8 1 2 857 0618 2

7 937 2 32 1 2 1357 0518 2

7 938 1 32 1 1 1743 0568 1

7 939 1 8 1 1 777 0618 1

7 940 1 8 1 1 1037 0568 1

7 941 2 16 1 2 1630 0618 2

7 942 2 16 0 2 2231 0568 2

7 943 2 32 1 2 2391 0618 2

7 944 1 8 1 1 1465 0668 2

7 945 2 8 1 2 1003 0568 2

7 946 1 32 1 1 853 0518 1

7 947 2 32 1 2 915 0568 2

7 948 1 32 1 1 750 0568 1

7 949 1 16 1 1 1015 0568 1

7 950 1 8 1 1 989 0618 1

7 951 2 8 1 2 956 0468 2

7 952 1 8 1 1 824 0618 1

7 953 2 32 0 2 766 0568 2

7 954 2 32 1 2 1359 0468 2

7 955 2 16 1 2 788 0568 2

7 956 1 32 1 1 890 0618 1

7 957 1 8 1 1 799 0618 1

7 958 1 8 1 1 1013 0518 2

7 959 1 8 1 1 849 0518 1

7 960 1 8 1 1 668 0518 2

7 961 2 8 1 2 922 0518 2

7 962 1 16 1 1 1660 0618 1

7 963 2 8 1 2 775 0668 1

7 964 1 16 1 1 1491 0618 1

7 965 2 16 1 2 1418 0668 2

7 966 1 32 1 1 1025 0668 1

7 967 2 16 1 2 716 0668 2

7 968 2 8 1 2 911 0518 1

7 969 2 16 1 2 1306 0618 2

7 970 2 32 1 2 1262 0568 2

7 971 1 32 1 1 1680 0518 1

7 972 2 8 1 2 1143 0468 2

7 973 1 8 1 1 805 0568 1

7 974 1 16 1 1 1054 0568 1

7 975 1 8 1 1 1471 0518 2

7 976 2 16 1 2 1214 0518 2

7 977 1 16 1 1 859 0568 1

7 978 1 16 1 1 1606 0668 1

7 979 1 8 1 1 1299 0668 1

7 980 2 8 1 2 860 0618 2

7 981 1 8 1 1 1093 0568 1

7 982 1 8 1 1 798 0668 2

7 983 2 16 1 2 821 0568 2

7 984 1 8 1 1 1168 0668 2

7 985 2 8 1 2 940 0568 2

7 986 1 8 1 1 2063 0618 1

7 987 2 16 1 2 1511 0518 2

7 988 2 8 1 2 637 0568 2

7 989 1 32 1 1 916 0518 1

7 990 2 32 1 2 1183 0468 2

7 991 2 32 1 2 1004 0568 2

7 992 2 8 1 2 666 0618 2

7 993 1 8 1 1 847 0668 2

7 994 1 16 1 1 1127 0518 1

7 995 1 32 1 1 718 0568 1

7 996 2 8 1 2 879 0468 2

7 997 2 8 1 2 865 0568 2

7 998 1 8 0 1 608 0668 2

7 999 1 8 1 1 1119 0618 1

7 1000 2 8 1 2 1141 0618 2

7 1001 2 16 1 2 802 0668 2

7 1002 1 32 1 1 985 0468 1

7 1003 1 16 1 1 1109 0618 1

7 1004 1 16 1 1 934 0618 1

7 1005 1 32 1 1 831 0618 1

7 1006 1 32 1 1 736 0518 1

7 1007 2 16 1 2 839 0668 2

7 1008 2 32 1 2 1008 0518 2

7 1009 1 16 1 1 1129 0568 1

7 1010 1 32 1 1 782 0568 1

7 1011 1 32 1 1 1024 0468 1

7 1012 1 16 1 1 636 0568 1

7 1013 2 32 1 2 906 0668 2

7 1014 1 32 1 1 930 0668 1

7 1015 2 32 1 2 978 0518 2

7 1016 2 32 1 2 711 0618 2

7 1017 1 16 1 1 1025 0618 1

7 1018 2 8 1 2 733 0618 2

7 1019 1 32 1 1 589 0618 1

7 1020 2 16 1 2 965 0468 2

7 1021 2 16 1 2 882 0468 2

7 1022 1 8 1 1 698 0668 1

7 1023 2 32 1 2 899 0618 2

7 1024 2 32 1 2 773 0668 2

7 1025 2 16 1 2 673 0668 2

7 1026 2 8 1 2 489 0668 2

7 1027 1 32 1 1 846 0568 1

7 1028 1 16 0 1 902 0468 1

7 1029 2 16 1 2 1126 0468 2

7 1030 1 32 1 1 959 0468 1

7 1031 2 32 1 2 1403 0468 2

7 1032 2 32 1 2 829 0668 2

7 1033 1 16 1 1 799 0668 1

7 1034 1 8 1 1 959 0668 1

7 1035 1 8 1 1 926 0618 1

7 1036 2 16 1 2 983 0468 2

7 1037 1 16 1 1 898 0518 1

7 1038 1 32 1 1 1009 0618 1

7 1039 2 32 0 2 806 0668 2

7 1040 1 32 1 1 887 0618 1

7 1041 1 8 1 1 819 0468 1

7 1042 2 32 1 2 943 0518 2

7 1043 2 32 1 2 964 0468 2

7 1044 1 32 1 1 1052 0468 1

7 1045 2 32 1 2 1061 0468 2

7 1046 1 8 1 1 1271 0518 1

7 1047 2 16 1 2 1003 0468 2

7 1048 1 16 1 1 1279 0668 1

7 1049 1 16 0 1 1055 0518 1

7 1050 2 16 1 2 895 0618 2

7 1051 1 8 1 1 1082 0668 1

7 1052 2 16 1 2 992 0518 2

7 1053 1 32 1 1 787 0518 1

7 1054 2 32 1 2 1219 0468 2

7 1055 1 16 1 1 1457 0568 1

7 1056 2 16 1 2 1752 0568 2

7 1057 1 16 1 1 898 0618 1

7 1058 1 8 1 1 935 0568 2

7 1059 2 8 0 2 611 0668 2

7 1060 1 8 1 1 1623 0468 1

7 1061 1 16 1 1 1798 0518 1

7 1062 1 16 1 1 949 0568 1

7 1063 1 32 1 1 834 0518 1

7 1064 1 8 1 1 733 0518 1

7 1065 1 32 1 1 636 0568 1

7 1066 2 8 1 2 1230 0468 2

7 1067 2 16 1 2 1224 0468 2

7 1068 1 16 0 1 1528 0518 1

7 1069 1 8 1 1 1062 0518 1

7 1070 2 8 1 2 757 0468 2

7 1071 1 32 0 1 918 0468 1

7 1072 2 8 1 2 707 0518 2

7 1073 1 8 1 1 1459 0468 1

7 1074 2 16 1 2 1561 0518 2

7 1075 2 32 1 2 1183 0568 2

7 1076 2 16 1 2 904 0618 2

7 1077 2 8 1 2 623 0668 2

7 1078 2 8 1 2 761 0518 1

7 1079 1 8 1 1 1288 0468 1

7 1080 1 16 0 1 748 0568 1

7 1081 1 8 1 1 867 0568 2

7 1082 1 32 1 1 947 0518 1

7 1083 2 8 1 2 1034 0568 2

7 1084 1 16 1 1 1328 0518 1

7 1085 1 16 1 1 707 0668 1

7 1086 2 8 1 2 805 0468 2

7 1087 1 8 1 1 754 0468 1

7 1088 2 8 1 2 759 0568 2

7 1089 1 16 1 1 3254 0468 1

7 1090 2 8 1 2 1267 0468 2

7 1091 2 16 1 2 1259 0568 2

7 1092 2 32 1 2 1060 0518 2

7 1093 1 8 1 1 739 0568 1

7 1094 2 32 1 2 1276 0668 2

7 1095 1 16 0 1 901 0568 1

7 1096 1 16 1 1 1279 0618 1

7 1097 2 8 1 2 765 0668 2

7 1098 2 16 1 2 779 0668 2

7 1099 2 16 1 2 697 0518 2

7 1100 1 32 1 1 860 0668 1

7 1101 1 16 0 1 4366 0468 1

7 1102 1 16 1 1 1990 0468 1

7 1103 1 8 1 1 778 0568 2

7 1104 1 8 1 1 1064 0618 1

7 1105 2 16 1 2 1027 0518 2

7 1106 2 8 1 2 651 0618 2

7 1107 2 32 1 2 1460 0568 2

7 1108 2 16 1 2 1078 0618 2

7 1109 2 32 1 2 1271 0468 2

7 1110 1 32 1 1 789 0568 1

7 1111 2 32 1 2 995 0568 2

7 1112 1 8 0 1 637 0518 2

7 1113 2 32 1 2 1009 0618 2

7 1114 1 8 1 1 1159 0568 2

7 1115 1 16 0 1 1137 0468 1

7 1116 2 8 1 2 1203 0518 1

7 1117 2 32 1 2 1512 0518 2

7 1118 2 32 1 2 963 0568 2

7 1119 1 32 1 1 926 0518 1

7 1120 2 32 1 2 1122 0618 2

7 1121 2 8 1 2 612 0618 2

7 1122 1 8 1 1 1153 0468 1

7 1123 2 32 1 2 871 0618 2

7 1124 2 32 1 2 788 0668 2

7 1125 2 8 1 2 663 0468 2

7 1126 2 8 1 2 599 0668 2

7 1127 1 16 1 1 1498 0468 1

7 1128 1 16 1 1 619 0668 1

7 1129 2 32 1 2 927 0568 2

7 1130 2 16 1 2 1508 0468 2

7 1131 1 16 1 1 1877 0518 1

7 1132 1 32 1 1 822 0668 1

7 1133 1 32 1 1 646 0568 1

7 1134 2 16 1 2 1448 0568 2

7 1135 2 32 1 2 884 0668 2

7 1136 2 32 1 2 984 0518 2

7 1137 1 8 1 1 831 0618 1

7 1138 1 8 1 1 681 0618 1

7 1139 2 16 1 2 1465 0618 2

7 1140 1 8 1 1 1349 0468 1

7 1141 2 16 0 2 1095 0568 2

7 1142 1 16 1 1 791 0668 1

7 1143 1 32 1 1 931 0618 1

7 1144 1 32 1 1 565 0618 1

7 1145 2 8 1 2 689 0618 2

7 1146 2 16 1 2 925 0668 2

7 1147 1 32 1 1 716 0668 1

7 1148 2 16 1 2 1019 0618 2

7 1149 1 32 1 1 650 0568 1

7 1150 2 16 1 2 1303 0568 2

7 1151 2 8 1 2 657 0468 2

7 1152 1 32 1 1 835 0618 1

7 1153 1 16 1 1 1427 0468 1

7 1154 1 16 1 1 798 0468 1

7 1155 1 8 1 1 900 0568 1

7 1156 2 32 1 2 1366 0468 2

7 1157 2 8 1 2 641 0568 1

7 1158 1 32 1 1 924 0468 1

7 1159 2 8 1 2 744 0618 2

7 1160 2 16 1 2 1037 0518 2

7 1161 2 32 1 2 1020 0618 2

7 1162 1 8 1 1 766 0518 2

7 1163 1 32 1 1 858 0468 1

7 1164 2 32 1 2 850 0618 2

7 1165 2 8 1 2 704 0668 1

7 1166 2 16 1 2 1550 0618 2

7 1167 1 8 1 1 1231 0618 1

7 1168 2 16 1 2 1438 0568 2

7 1169 2 16 1 2 1276 0518 2

7 1170 1 32 1 1 924 0568 1

7 1171 2 8 1 2 715 0568 2

7 1172 2 32 1 2 1314 0468 2

7 1173 2 16 1 2 898 0468 2

7 1174 2 32 1 2 1129 0668 2

7 1175 2 8 1 2 871 0518 2

7 1176 1 8 1 1 831 0618 1

7 1177 2 8 0 2 916 0518 2

7 1178 2 16 1 2 725 0668 2

7 1179 1 16 1 1 1372 0618 1

7 1180 1 16 1 1 1133 0518 1

7 1181 1 16 1 1 640 0668 1

7 1182 2 8 1 2 764 0668 2

7 1183 1 8 1 1 933 0518 1

7 1184 1 8 1 1 1282 0468 1

7 1185 1 16 1 1 666 0668 1

7 1186 1 32 1 1 851 0468 1

7 1187 1 32 1 1 1006 0668 1

7 1188 2 32 0 2 731 0568 2

7 1189 2 16 1 2 1019 0568 2

7 1190 1 32 1 1 2007 0618 1

7 1191 2 32 1 2 1342 0518 2

7 1192 1 32 1 1 1143 0518 1

7 1193 2 32 1 2 982 0618 2

7 1194 1 32 1 1 1101 0668 1

7 1195 2 32 1 2 1300 0518 2

7 1196 2 8 1 2 821 0568 1

7 1197 1 32 1 1 1012 0668 1

7 1198 2 16 1 2 2408 0618 2

7 1199 2 8 1 2 615 0518 1

7 1200 1 8 1 1 929 0518 2

8 1 1 8 1 1 3106 0618 1

8 2 2 8 1 2 819 0468 2

8 3 2 16 1 2 1989 0468 2

8 4 1 32 1 1 1174 0468 1

8 5 1 16 1 1 1929 0468 1

8 6 2 16 1 2 947 0468 2

8 7 1 8 1 1 726 0468 1

8 8 2 32 1 2 750 0468 2

8 9 1 16 1 1 1129 0468 1

8 10 2 8 1 2 846 0518 2

8 11 2 32 1 2 956 0518 2

8 12 1 8 1 1 744 0618 1

8 13 1 16 1 1 1070 0468 1

8 14 1 8 1 1 832 0518 2

8 15 2 32 1 2 826 0568 2

8 16 1 32 1 1 1026 0618 1

8 17 1 8 1 1 967 0618 1

8 18 1 8 1 1 772 0518 1

8 19 2 32 1 2 751 0518 2

8 20 1 32 1 1 1102 0568 1

8 21 1 8 1 1 2467 0468 1

8 22 1 8 1 1 727 0568 2

8 23 1 32 1 1 837 0618 1

8 24 1 16 1 1 743 0668 1

8 25 2 32 1 2 857 0568 2

8 26 2 32 1 2 1223 0568 2

8 27 1 32 1 1 1110 0668 1

8 28 1 8 1 1 986 0618 1

8 29 2 16 1 2 983 0668 2

8 30 1 32 1 1 1403 0518 1

8 31 1 8 1 1 681 0568 2

8 32 2 16 1 2 1491 0518 2

8 33 2 16 1 2 999 0568 2

8 34 2 16 1 2 728 0668 2

8 35 2 16 1 2 1123 0518 2

8 36 2 32 1 2 1219 0568 2

8 37 1 16 1 1 1303 0518 1

8 38 1 32 1 1 1276 0518 1

8 39 1 16 1 1 1068 0668 1

8 40 2 16 1 2 865 0668 2

8 41 1 8 1 1 950 0518 1

8 42 2 16 1 2 2144 0468 2

8 43 1 32 1 1 1201 0668 1

8 44 2 8 1 2 864 0668 2

8 45 1 16 1 1 1079 0568 1

8 46 1 16 1 1 1461 0568 1

8 47 1 16 1 1 987 0468 1

8 48 2 32 1 2 1119 0468 2

8 49 1 32 1 1 981 0518 1

8 50 2 16 1 2 1705 0618 2

8 51 2 8 1 2 815 0568 2

8 52 2 32 1 2 1044 0568 2

8 53 2 32 1 2 1351 0668 2

8 54 2 32 1 2 837 0668 2

8 55 1 32 1 1 1398 0568 1

8 56 2 16 1 2 1245 0618 2

8 57 2 16 1 2 1399 0568 2

8 58 2 16 1 2 992 0468 2

8 59 1 16 0 1 1894 0618 1

8 60 1 16 1 1 2353 0668 1

8 61 2 32 0 2 952 0468 2

8 62 2 8 1 2 1208 0518 2

8 63 2 16 1 2 2279 0618 2

8 64 1 16 1 1 3765 0618 1

8 65 2 32 1 2 1140 0568 2

8 66 2 16 1 2 1185 0568 2

8 67 1 32 1 1 1278 0668 1

8 68 1 8 1 1 1171 0618 1

8 69 2 32 1 2 1179 0468 2

8 70 1 32 1 1 915 0568 1

8 71 1 32 1 1 2398 0618 1

8 72 1 8 1 1 829 0518 2

8 73 2 16 1 2 1327 0618 2

8 74 2 32 1 2 874 0618 2

8 75 2 32 1 2 886 0668 2

8 76 2 32 1 2 733 0618 2

8 77 2 8 1 2 1458 0468 2

8 78 2 8 0 2 1074 0468 2

8 79 2 32 1 2 904 0568 2

8 80 2 8 1 2 736 0668 2

8 81 1 16 1 1 940 0518 1

8 82 1 16 1 1 1440 0618 1

8 83 2 8 1 2 623 0668 1

8 84 2 16 0 2 5724 0468 2

8 85 1 8 1 1 1098 0668 1

8 86 1 8 1 1 782 0668 2

8 87 2 32 1 2 805 0668 2

8 88 1 8 1 1 750 0568 2

8 89 2 8 1 2 755 0568 2

8 90 2 16 1 2 675 0668 2

8 91 1 16 1 1 1308 0468 1

8 92 1 16 1 1 737 0668 1

8 93 1 16 1 1 969 0618 1

8 94 1 32 1 1 1483 0668 1

8 95 1 32 1 1 1868 0668 1

8 96 2 32 1 2 847 0468 2

8 97 1 32 1 1 972 0568 1

8 98 2 8 1 2 1559 0568 2

8 99 1 32 1 1 849 0568 1

8 100 2 32 0 2 911 0518 2

8 101 2 32 1 2 1083 0668 2

8 102 2 8 0 2 973 0518 1

8 103 2 8 1 2 1314 0568 1

8 104 2 8 1 2 642 0668 2

8 105 1 16 1 1 859 0668 1

8 106 1 16 1 1 938 0518 1

8 107 1 32 1 1 1422 0468 1

8 108 2 8 1 2 690 0618 2

8 109 2 8 1 2 840 0468 2

8 110 2 8 1 2 1128 0618 2

8 111 2 32 1 2 1030 0618 2

8 112 1 32 1 1 1403 0518 1

8 113 2 16 1 2 1363 0568 2

8 114 1 32 1 1 1160 0518 1

8 115 1 32 1 1 1330 0618 1

8 116 2 16 1 2 1273 0518 2

8 117 1 32 1 1 1560 0568 1

8 118 1 16 1 1 788 0668 1

8 119 2 8 1 2 1110 0518 1

8 120 2 8 1 2 653 0668 2

8 121 2 32 1 2 1001 0618 2

8 122 1 32 1 1 956 0568 1

8 123 1 8 1 1 1382 0518 1

8 124 1 8 1 1 1053 0518 2

8 125 1 16 0 1 873 0568 1

8 126 2 8 1 2 927 0468 2

8 127 1 32 0 1 1308 0568 1

8 128 1 8 1 1 876 0518 2

8 129 2 8 1 2 919 0468 2

8 130 1 16 1 1 2801 0618 1

8 131 1 16 1 1 1742 0518 1

8 132 2 16 1 2 1137 0568 2

8 133 2 8 1 2 10951 0568 1

8 134 2 32 1 2 1111 0618 2

8 135 1 16 1 1 834 0668 1

8 136 2 16 1 2 1608 0518 2

8 137 2 8 1 2 905 0468 2

8 138 1 8 1 1 875 0568 1

8 139 1 16 1 1 2872 0518 1

8 140 1 8 1 1 878 0518 1

8 141 2 8 1 2 921 0518 2

8 142 2 16 1 2 708 0568 2

8 143 2 32 1 2 721 0518 2

8 144 2 32 1 2 855 0468 2

8 145 2 32 1 2 909 0518 2

8 146 1 16 1 1 1107 0568 1

8 147 1 32 1 1 1533 0518 1

8 148 2 8 1 2 847 0668 2

8 149 2 8 1 2 1013 0568 2

8 150 1 32 1 1 1089 0668 1

8 151 2 16 1 2 1068 0568 2

8 152 2 8 1 2 821 0518 1

8 153 1 16 1 1 3131 0618 1

8 154 1 8 1 1 1750 0468 1

8 155 2 16 1 2 1418 0518 2

8 156 1 32 1 1 1280 0518 1

8 157 2 32 1 2 1456 0518 2

8 158 1 16 1 1 1273 0468 1

8 159 2 8 1 2 913 0568 2

8 160 2 16 1 2 824 0668 2

8 161 1 16 1 1 1011 0568 1

8 162 1 32 1 1 1144 0618 1

8 163 1 16 1 1 1023 0568 1

8 164 1 8 1 1 895 0568 1

8 165 1 8 1 1 992 0468 1

8 166 2 8 1 2 783 0618 2

8 167 1 32 1 1 1014 0668 1

8 168 2 16 1 2 1350 0518 2

8 169 2 16 1 2 918 0568 2

8 170 2 32 1 2 1795 0518 2

8 171 2 16 1 2 1104 0618 2

8 172 2 32 1 2 852 0618 2

8 173 1 32 1 1 899 0618 1

8 174 2 16 1 2 1705 0618 2

8 175 2 16 1 2 3316 0468 2

8 176 1 8 1 1 785 0568 1

8 177 1 16 1 1 849 0518 1

8 178 1 8 1 1 622 0668 2

8 179 2 8 1 2 1571 0618 2

8 180 1 8 1 1 1847 0668 2

8 181 1 16 1 1 2643 0618 1

8 182 1 32 1 1 1055 0668 1

8 183 1 16 1 1 1173 0468 1

8 184 2 8 1 2 801 0568 2

8 185 1 32 1 1 1245 0468 1

8 186 1 32 1 1 2246 0518 1

8 187 2 8 1 2 748 0668 2

8 188 2 32 1 2 836 0668 2

8 189 1 8 1 1 725 0668 1

8 190 1 8 1 1 1224 0468 1

8 191 2 16 1 2 1404 0518 2

8 192 1 8 1 1 951 0668 2

8 193 2 8 1 2 1127 0618 2

8 194 2 16 0 2 941 0468 2

8 195 2 32 1 2 803 0518 2

8 196 1 16 1 1 780 0668 1

8 197 1 8 1 1 843 0668 1

8 198 1 8 1 1 938 0618 1

8 199 2 8 1 2 798 0618 2

8 200 2 32 1 2 891 0618 2

8 201 2 32 1 2 1106 0618 2

8 202 2 8 1 2 785 0618 2

8 203 1 8 1 1 918 0618 1

8 204 2 32 1 2 1084 0568 2

8 205 2 16 0 2 956 0468 2

8 206 1 8 1 1 849 0568 1

8 207 1 16 1 1 834 0518 1

8 208 2 32 1 2 1153 0468 2

8 209 1 32 1 1 934 0468 1

8 210 2 16 1 2 1095 0618 2

8 211 2 16 1 2 739 0668 2

8 212 1 8 1 1 877 0668 1

8 213 2 8 1 2 1518 0518 2

8 214 2 8 1 2 846 0668 1

8 215 2 16 1 2 789 0668 2

8 216 1 16 1 1 1201 0618 1

8 217 1 8 1 1 1001 0568 2

8 218 2 32 1 2 1034 0668 2

8 219 1 32 1 1 3840 0468 1

8 220 1 16 1 1 993 0518 1

8 221 1 8 1 1 816 0468 1

8 222 1 8 1 1 797 0468 1

8 223 2 16 1 2 994 0518 2

8 224 2 32 1 2 938 0468 2

8 225 1 16 1 1 2979 0468 1

8 226 1 8 1 1 4781 0468 1

8 227 2 8 0 2 1073 0518 1

8 228 1 32 1 1 5886 0468 1

8 229 2 16 1 2 1153 0668 2

8 230 1 32 1 1 884 0468 1

8 231 1 32 1 1 881 0468 1

8 232 2 32 1 2 816 0668 2

8 233 2 8 1 2 985 0618 2

8 234 1 16 1 1 921 0568 1

8 235 2 16 1 2 1547 0618 2

8 236 1 8 1 1 1066 0618 1

8 237 2 8 1 2 927 0468 2

8 238 1 16 1 1 1097 0568 1

8 239 1 32 1 1 1252 0618 1

8 240 1 32 1 1 2735 0618 1

8 241 1 8 1 1 812 0618 1

8 242 1 16 0 1 1088 0468 1

8 243 1 8 1 1 777 0568 2

8 244 1 8 1 1 762 0668 1

8 245 2 32 1 2 852 0618 2

8 246 1 32 1 1 880 0568 1

8 247 1 32 1 1 701 0618 1

8 248 1 16 1 1 795 0518 1

8 249 1 32 1 1 828 0668 1

8 250 1 16 1 1 3697 0518 1

8 251 2 8 0 2 744 0468 2

8 252 1 8 1 1 778 0518 2

8 253 1 16 1 1 833 0618 1

8 254 2 8 1 2 1248 0618 2

8 255 1 8 1 1 670 0618 1

8 256 2 16 1 2 1308 0568 2

8 257 1 16 1 1 736 0518 1

8 258 1 8 1 1 750 0518 2

8 259 2 32 1 2 851 0568 2

8 260 1 8 1 1 688 0468 1

8 261 2 32 1 2 886 0668 2

8 262 2 16 0 2 1037 0468 2

8 263 1 8 1 1 2546 0468 1

8 264 1 32 1 1 898 0568 1

8 265 2 32 1 2 734 0568 2

8 266 1 32 1 1 875 0618 1

8 267 1 8 1 1 855 0668 2

8 268 1 16 1 1 3508 0518 1

8 269 1 32 1 1 813 0618 1

8 270 1 8 1 1 702 0668 1

8 271 2 16 1 2 860 0668 2

8 272 2 16 1 2 1869 0518 2

8 273 2 32 1 2 1218 0618 2

8 274 2 8 1 2 1002 0518 1

8 275 1 32 1 1 1038 0668 1

8 276 1 8 1 1 4517 0468 1

8 277 2 32 1 2 1161 0518 2

8 278 1 32 1 1 823 0568 1

8 279 1 8 1 1 1285 0618 1

8 280 2 16 0 2 1011 0468 2

8 281 1 32 1 1 1028 0518 1

8 282 2 16 0 2 968 0518 2

8 283 2 32 1 2 1138 0568 2

8 284 1 16 1 1 858 0468 1

8 285 2 8 1 2 800 0568 1

8 286 1 16 1 1 1104 0618 1

8 287 2 8 1 2 834 0668 2

8 288 2 16 1 2 1631 0618 2

8 289 1 32 1 1 7138 0568 1

8 290 1 32 1 1 828 0618 1

8 291 1 8 1 1 858 0568 1

8 292 2 8 1 2 793 0618 2

8 293 1 16 1 1 786 0518 1

8 294 1 32 1 1 674 0568 1

8 295 2 32 0 2 925 0668 2

8 296 1 16 1 1 827 0518 1

8 297 2 16 1 2 716 0668 2

8 298 1 8 1 1 547 0518 1

8 299 2 32 1 2 967 0518 2

8 300 1 16 1 1 595 0518 1

8 301 1 32 1 1 961 0468 1

8 302 1 32 1 1 781 0668 1

8 303 2 16 1 2 2510 0568 2

8 304 1 32 0 1 1138 0518 1

8 305 1 8 1 1 1305 0668 2

8 306 2 32 1 2 731 0518 2

8 307 2 32 1 2 1298 0468 2

8 308 1 8 1 1 715 0568 2

8 309 2 32 1 2 994 0468 2

8 310 2 16 0 2 784 0518 2

8 311 1 16 1 1 6165 0618 1

8 312 2 32 1 2 1094 0568 2

8 313 2 32 1 2 1398 0468 2

8 314 2 16 1 2 1800 0668 2

8 315 2 16 1 2 1910 0618 2

8 316 1 8 1 1 961 0618 1

8 317 1 32 1 1 6393 0518 1

8 318 1 8 1 1 764 0568 1

8 319 1 8 0 1 814 0518 1

8 320 2 8 1 2 791 0568 2

8 321 1 32 1 1 7281 0518 1

8 322 1 8 1 1 726 0568 1

8 323 2 8 1 2 946 0518 1

8 324 1 32 1 1 801 0618 1

8 325 2 32 1 2 1259 0668 2

8 326 2 8 1 2 913 0668 2

8 327 2 32 1 2 1116 0668 2

8 328 2 32 1 2 923 0618 2

8 329 1 16 1 1 2227 0468 1

8 330 2 16 1 2 825 0668 2

8 331 1 16 1 1 1235 0618 1

8 332 2 16 1 2 1083 0618 2

8 333 2 32 1 2 912 0568 2

8 334 1 32 1 1 963 0618 1

8 335 2 16 1 2 763 0618 2

8 336 1 16 1 1 4867 0468 1

8 337 2 8 1 2 921 0568 2

8 338 2 8 1 2 889 0568 2

8 339 1 32 1 1 1422 0668 1

8 340 2 8 1 2 770 0668 2

8 341 1 16 1 1 1150 0618 1

8 342 2 8 1 2 864 0618 2

8 343 1 8 1 1 819 0618 1

8 344 2 16 1 2 1019 0518 2

8 345 2 8 1 2 631 0668 1

8 346 1 32 1 1 1526 0568 1

8 347 1 32 1 1 873 0568 1

8 348 2 8 1 2 642 0668 1

8 349 1 16 1 1 1079 0568 1

8 350 1 16 1 1 1563 0568 1

8 351 1 16 1 1 808 0668 1

8 352 1 8 1 1 1465 0618 1

8 353 2 8 1 2 1345 0518 2

8 354 2 8 1 2 1135 0468 2

8 355 1 8 1 1 864 0518 1

8 356 1 8 1 1 1070 0568 2

8 357 1 8 1 1 1400 0468 1

8 358 1 32 1 1 6854 0668 1

8 359 2 32 1 2 1315 0618 2

8 360 1 16 1 1 1106 0468 1

8 361 2 8 1 2 812 0618 2

8 362 2 8 1 2 1278 0468 2

8 363 1 8 1 1 1355 0468 1

8 364 2 16 1 2 1030 0468 2

8 365 2 32 1 2 882 0568 2

8 366 1 32 1 1 1351 0518 1

8 367 2 16 1 2 806 0568 2

8 368 1 16 1 1 1241 0568 1

8 369 2 8 1 2 841 0468 2

8 370 2 8 1 2 816 0518 2

8 371 2 8 1 2 746 0468 2

8 372 2 16 1 2 1236 0618 2

8 373 1 8 1 1 5493 0468 1

8 374 2 16 1 2 745 0568 2

8 375 2 16 1 2 1576 0468 2

8 376 1 32 1 1 985 0618 1

8 377 2 16 1 2 1522 0668 2

8 378 1 8 1 1 4812 0568 1

8 379 2 8 1 2 651 0518 2

8 380 1 16 1 1 966 0668 1

8 381 1 32 1 1 1067 0518 1

8 382 1 32 1 1 855 0468 1

8 383 2 16 1 2 1193 0468 2

8 384 1 32 1 1 870 0468 1

8 385 2 16 1 2 1003 0618 2

8 386 1 16 1 1 703 0568 1

8 387 1 16 1 1 725 0668 1

8 388 2 16 1 2 596 0668 2

8 389 1 8 1 1 800 0668 1

8 390 2 32 1 2 1011 0468 2

8 391 2 32 1 2 1332 0618 2

8 392 1 16 1 1 1770 0618 1

8 393 1 16 1 1 5336 0568 1

8 394 1 16 1 1 868 0468 1

8 395 1 8 1 1 1024 0468 1

8 396 2 8 1 2 807 0618 2

8 397 2 32 1 2 1016 0618 2

8 398 2 32 1 2 714 0568 2

8 399 1 8 1 1 812 0668 2

8 400 2 16 1 2 1132 0518 2

8 401 2 8 1 2 725 0568 2

8 402 2 32 1 2 624 0618 2

8 403 2 32 1 2 751 0518 2

8 404 2 32 1 2 742 0468 2

8 405 1 8 1 1 1118 0468 1

8 406 2 16 1 2 763 0568 2

8 407 2 32 1 2 767 0468 2

8 408 1 8 1 1 731 0668 1

8 409 2 32 1 2 867 0518 2

8 410 2 8 1 2 1016 0468 2

8 411 1 32 1 1 1163 0568 1

8 412 2 16 1 2 814 0568 2

8 413 2 16 1 2 825 0468 2

8 414 1 16 1 1 1303 0618 1

8 415 1 8 1 1 1137 0618 1

8 416 1 16 1 1 4119 0618 1

8 417 2 32 0 2 1039 0518 2

8 418 1 32 1 1 781 0668 1

8 419 2 16 0 2 716 0668 2

8 420 2 8 1 2 896 0568 1

8 421 2 8 1 2 825 0568 2

8 422 1 8 1 1 854 0518 1

8 423 2 8 1 2 623 0468 2

8 424 1 32 0 1 826 0518 1

8 425 2 16 1 2 1535 0468 2

8 426 2 8 1 2 739 0568 2

8 427 1 32 1 1 1031 0468 1

8 428 1 16 1 1 1098 0568 1

8 429 2 32 1 2 1314 0468 2

8 430 2 8 1 2 651 0618 2

8 431 2 16 1 2 1103 0568 2

8 432 1 8 1 1 1178 0618 1

8 433 1 16 1 1 1508 0468 1

8 434 2 32 1 2 6451 0518 2

8 435 2 32 1 2 1050 0668 2

8 436 1 16 1 1 770 0668 1

8 437 2 8 1 2 819 0618 2

8 438 1 32 1 1 942 0618 1

8 439 1 8 1 1 805 0668 2

8 440 1 32 1 1 1059 0668 1

8 441 2 8 1 2 1209 0518 2

8 442 1 32 1 1 907 0468 1

8 443 2 16 1 2 808 0668 2

8 444 2 8 1 2 883 0518 1

8 445 1 16 1 1 723 0668 1

8 446 2 32 1 2 1036 0568 2

8 447 1 32 1 1 1615 0468 1

8 448 1 16 1 1 1184 0468 1

8 449 1 16 1 1 915 0668 1

8 450 2 16 1 2 940 0518 2

8 451 2 16 1 2 1001 0468 2

8 452 1 16 1 1 1143 0568 1

8 453 1 16 1 1 762 0668 1

8 454 1 32 1 1 1367 0468 1

8 455 2 16 1 2 863 0518 2

8 456 2 8 1 2 781 0618 2

8 457 2 8 1 2 728 0468 2

8 458 2 8 1 2 710 0668 2

8 459 1 8 1 1 914 0568 2

8 460 2 32 1 2 861 0468 2

8 461 2 16 1 2 1393 0618 2

8 462 1 16 1 1 1105 0668 1

8 463 1 16 0 1 1104 0568 1

8 464 2 8 1 2 744 0518 1

8 465 2 32 1 2 715 0618 2

8 466 1 32 1 1 871 0518 1

8 467 2 32 1 2 872 0668 2

8 468 1 8 1 1 4521 0518 2

8 469 1 16 1 1 1277 0518 1

8 470 2 16 1 2 755 0518 2

8 471 2 8 1 2 722 0668 2

8 472 2 32 1 2 1476 0518 2

8 473 2 16 1 2 997 0568 2

8 474 2 8 1 2 586 0668 2

8 475 1 32 1 1 4469 0468 1

8 476 2 32 1 2 1335 0668 2

8 477 1 8 1 1 860 0518 2

8 478 2 32 1 2 807 0668 2

8 479 1 32 1 1 798 0668 1

8 480 2 16 1 2 1479 0618 2

8 481 2 32 1 2 755 0568 2

8 482 2 16 1 2 673 0668 2

8 483 1 8 1 1 734 0568 2

8 484 1 16 1 1 2206 0468 1

8 485 2 16 1 2 1600 0468 2

8 486 2 32 1 2 1117 0518 2

8 487 1 32 1 1 869 0468 1

8 488 1 8 1 1 1023 0618 1

8 489 1 16 1 1 1011 0518 1

8 490 2 32 0 2 964 0518 2

8 491 1 16 1 1 756 0568 1

8 492 2 32 1 2 794 0668 2

8 493 1 16 1 1 1237 0468 1

8 494 2 16 1 2 1313 0618 2

8 495 2 8 1 2 1606 0468 2

8 496 1 32 1 1 977 0568 1

8 497 1 32 1 1 729 0518 1

8 498 1 32 1 1 1020 0518 1

8 499 1 16 1 1 952 0468 1

8 500 2 32 1 2 1132 0468 2

8 501 2 8 1 2 1195 0518 2

8 502 1 32 1 1 1221 0518 1

8 503 2 8 1 2 1358 0518 2

8 504 1 32 1 1 4525 0668 1

8 505 2 8 1 2 760 0668 2

8 506 2 16 1 2 955 0468 2

8 507 2 8 0 2 764 0618 2

8 508 2 8 1 2 570 0618 2

8 509 2 32 1 2 738 0668 2

8 510 2 8 1 2 741 0468 2

8 511 1 32 1 1 1993 0668 1

8 512 1 16 1 1 5342 0568 1

8 513 2 16 1 2 843 0568 2

8 514 2 8 1 2 831 0618 2

8 515 2 32 1 2 992 0468 2

8 516 1 32 1 1 907 0668 1

8 517 1 16 1 1 889 0668 1

8 518 2 16 1 2 886 0618 2

8 519 2 16 1 2 1107 0518 2

8 520 1 16 1 1 1544 0468 1

8 521 2 8 1 2 754 0568 1

8 522 1 8 1 1 896 0568 2

8 523 1 16 1 1 1279 0518 1

8 524 1 8 0 1 639 0618 1

8 525 1 32 1 1 923 0468 1

8 526 2 8 1 2 721 0668 1

8 527 1 8 1 1 779 0668 1

8 528 2 8 1 2 650 0668 2

8 529 2 16 1 2 1127 0518 2

8 530 1 8 1 1 813 0518 1

8 531 1 32 1 1 1152 0668 1

8 532 2 32 1 2 1152 0668 2

8 533 1 8 1 1 10440 0518 1

8 534 1 8 1 1 856 0568 2

8 535 2 32 1 2 770 0618 2

8 536 2 16 1 2 904 0668 2

8 537 1 16 1 1 815 0568 1

8 538 1 8 1 1 862 0668 2

8 539 1 32 1 1 961 0568 1

8 540 2 16 1 2 1593 0618 2

8 541 1 8 1 1 726 0668 1

8 542 1 32 1 1 1018 0518 1

8 543 1 32 1 1 899 0618 1

8 544 2 8 1 2 986 0618 2

8 545 1 32 1 1 621 0618 1

8 546 1 32 1 1 705 0518 1

8 547 1 32 1 1 889 0668 1

8 548 2 32 1 2 793 0618 2

8 549 2 32 1 2 1123 0618 2

8 550 2 32 1 2 1285 0468 2

8 551 1 16 1 1 1159 0568 1

8 552 1 32 1 1 1042 0668 1

8 553 1 16 0 1 1464 0618 1

8 554 1 8 0 1 776 0618 1

8 555 2 16 0 2 992 0518 2

8 556 2 16 1 2 2816 0518 2

8 557 1 16 1 1 1174 0568 1

8 558 2 8 1 2 1287 0518 1

8 559 1 32 1 1 994 0668 1

8 560 1 32 1 1 715 0568 1

8 561 2 16 0 2 1303 0518 2

8 562 2 16 1 2 1438 0618 2

8 563 1 8 1 1 756 0668 2

8 564 2 16 1 2 745 0668 2

8 565 2 32 1 2 912 0618 2

8 566 1 16 0 1 1459 0518 1

8 567 1 16 1 1 2825 0468 1

8 568 2 16 1 2 1443 0468 2

8 569 2 16 1 2 938 0468 2

8 570 2 32 1 2 977 0568 2

8 571 2 16 1 2 1615 0568 2

8 572 2 8 1 2 837 0518 1

8 573 2 16 1 2 797 0668 2

8 574 2 16 1 2 705 0568 2

8 575 2 32 1 2 842 0668 2

8 576 1 16 1 1 2840 0618 1

8 577 2 16 1 2 907 0568 2

8 578 2 16 1 2 638 0668 2

8 579 1 16 0 1 865 0518 1

8 580 2 8 1 2 653 0668 2

8 581 2 8 1 2 703 0668 2

8 582 2 32 1 2 989 0468 2

8 583 1 16 1 1 3391 0618 1

8 584 2 8 1 2 1857 0568 2

8 585 1 16 1 1 1034 0468 1

8 586 1 8 1 1 3558 0518 2

8 587 1 16 1 1 1552 0618 1

8 588 1 16 1 1 1359 0618 1

8 589 2 16 1 2 889 0568 2

8 590 1 8 1 1 822 0668 2

8 591 1 8 1 1 641 0518 2

8 592 1 32 1 1 1504 0468 1

8 593 2 32 1 2 707 0568 2

8 594 1 32 1 1 913 0468 1

8 595 2 32 1 2 941 0568 2

8 596 1 32 1 1 741 0618 1

8 597 2 32 1 2 917 0468 2

8 598 2 8 1 2 1007 0568 2

8 599 2 32 1 2 797 0568 2

8 600 2 8 1 2 1070 0468 2

8 601 1 32 1 1 1116 0468 1

8 602 1 8 1 1 820 0518 2

8 603 2 32 1 2 1148 0618 2

8 604 1 8 1 1 804 0618 1

8 605 2 8 1 2 1158 0568 2

8 606 2 32 1 2 738 0668 2

8 607 1 32 1 1 864 0568 1

8 608 1 16 1 1 925 0518 1

8 609 1 8 1 1 738 0468 1

8 610 1 8 1 1 1127 0618 1

8 611 1 8 1 1 706 0518 1

8 612 1 16 1 1 647 0518 1

8 613 2 32 1 2 757 0518 2

8 614 1 32 1 1 962 0668 1

8 615 1 8 1 1 653 0468 1

8 616 1 8 1 1 713 0668 2

8 617 2 8 1 2 842 0618 2

8 618 1 8 1 1 729 0468 1

8 619 1 8 1 1 895 0468 1

8 620 2 8 1 2 851 0618 2

8 621 2 32 1 2 737 0668 2

8 622 2 16 1 2 1340 0468 2

8 623 2 8 1 2 780 0568 2

8 624 1 16 1 1 1608 0668 1

8 625 1 8 1 1 938 0568 1

8 626 1 8 1 1 4027 0618 1

8 627 2 16 1 2 1155 0618 2

8 628 1 16 1 1 752 0618 1

8 629 1 8 0 1 635 0618 1

8 630 1 32 1 1 895 0518 1

8 631 2 16 1 2 1100 0468 2

8 632 1 16 1 1 684 0668 1

8 633 1 16 1 1 785 0568 1

8 634 1 32 1 1 3253 0518 1

8 635 1 8 1 1 755 0468 1

8 636 2 8 1 2 777 0468 2

8 637 2 8 1 2 819 0468 2

8 638 2 32 1 2 1065 0468 2

8 639 2 16 1 2 857 0618 2

8 640 1 8 1 1 1010 0568 2

8 641 1 16 1 1 697 0668 1

8 642 1 8 1 1 708 0568 1

8 643 2 8 1 2 2105 0568 1

8 644 1 16 1 1 758 0568 1

8 645 2 32 1 2 835 0518 2

8 646 2 32 1 2 582 0568 2

8 647 2 8 1 2 760 0518 1

8 648 1 16 1 1 892 0668 1

8 649 1 32 1 1 1532 0468 1

8 650 1 32 1 1 1643 0618 1

8 651 1 16 1 1 643 0668 1

8 652 1 32 1 1 675 0618 1

8 653 1 16 1 1 687 0568 1

8 654 1 16 1 1 797 0518 1

8 655 1 16 1 1 1110 0468 1

8 656 1 8 1 1 846 0568 1

8 657 1 8 1 1 901 0468 1

8 658 2 8 1 2 756 0518 2

8 659 2 16 1 2 1080 0568 2

8 660 1 8 1 1 843 0568 1

8 661 2 32 1 2 1459 0518 2

8 662 2 16 1 2 1300 0468 2

8 663 1 8 1 1 747 0468 1

8 664 2 8 1 2 1025 0668 1

8 665 2 16 1 2 1056 0468 2

8 666 2 8 1 2 835 0618 2

8 667 1 32 1 1 1241 0568 1

8 668 1 32 1 1 1285 0518 1

8 669 1 32 1 1 1007 0568 1

8 670 1 8 1 1 826 0518 2

8 671 1 16 1 1 673 0668 1

8 672 1 32 1 1 771 0468 1

8 673 2 8 1 2 767 0518 2

8 674 2 32 1 2 1123 0518 2

8 675 1 8 1 1 3940 0668 1

8 676 2 32 1 2 928 0618 2

8 677 2 16 1 2 2076 0518 2

8 678 1 16 1 1 1245 0618 1

8 679 2 8 1 2 795 0518 1

8 680 1 8 1 1 706 0668 1

8 681 1 32 1 1 890 0568 1

8 682 2 8 1 2 840 0568 2

8 683 2 32 1 2 887 0468 2

8 684 2 32 1 2 714 0618 2

8 685 2 16 1 2 952 0618 2

8 686 1 8 1 1 967 0518 1

8 687 2 8 1 2 927 0468 2

8 688 2 8 1 2 1151 0468 2

8 689 2 8 1 2 652 0668 2

8 690 2 16 1 2 1103 0618 2

8 691 2 32 1 2 732 0568 2

8 692 1 32 1 1 951 0618 1

8 693 2 8 1 2 840 0568 2

8 694 2 32 1 2 812 0518 2

8 695 2 8 1 2 712 0668 2

8 696 2 32 1 2 731 0668 2

8 697 2 32 1 2 1112 0518 2

8 698 2 16 1 2 711 0518 2

8 699 2 16 0 2 953 0568 2

8 700 2 16 1 2 589 0668 2

8 701 1 32 1 1 1073 0618 1

8 702 2 16 1 2 1023 0568 2

8 703 2 32 1 2 1028 0568 2

8 704 2 8 1 2 878 0618 2

8 705 2 8 1 2 709 0468 2

8 706 1 16 1 1 1155 0618 1

8 707 1 32 1 1 3302 0568 1

8 708 1 16 1 1 781 0518 1

8 709 2 16 1 2 592 0668 2

8 710 1 16 1 1 910 0668 1

8 711 2 32 1 2 738 0668 2

8 712 2 32 1 2 1101 0468 2

8 713 1 16 1 1 742 0468 1

8 714 1 8 0 1 937 0468 1

8 715 1 32 1 1 846 0468 1

8 716 2 32 1 2 841 0618 2

8 717 2 16 1 2 835 0518 2

8 718 2 16 1 2 675 0668 2

8 719 1 8 1 1 889 0618 1

8 720 1 32 1 1 880 0618 1

8 721 2 16 1 2 911 0468 2

8 722 1 16 1 1 1523 0618 1

8 723 2 32 1 2 977 0468 2

8 724 1 16 0 1 1236 0468 1

8 725 1 8 1 1 829 0468 1

8 726 2 16 1 2 1363 0468 2

8 727 2 8 1 2 691 0518 2

8 728 1 16 0 1 645 0518 1

8 729 2 8 1 2 951 0518 1

8 730 2 8 1 2 663 0618 2

8 731 1 16 1 1 1852 0618 1

8 732 2 8 1 2 1007 0518 1

8 733 2 8 1 2 907 0468 2

8 734 1 16 0 1 1888 0468 1

8 735 2 32 1 2 872 0468 2

8 736 1 8 1 1 745 0668 2

8 737 1 16 1 1 1421 0568 1

8 738 1 8 1 1 799 0518 1

8 739 1 16 1 1 1753 0518 1

8 740 2 8 1 2 1075 0618 2

8 741 2 16 1 2 886 0668 2

8 742 2 16 1 2 647 0668 2

8 743 2 32 1 2 794 0668 2

8 744 2 8 1 2 748 0468 2

8 745 2 16 1 2 789 0568 2

8 746 1 32 1 1 5908 0618 1

8 747 1 16 0 1 1317 0518 1

8 748 1 16 1 1 825 0518 1

8 749 2 8 1 2 1343 0518 1

8 750 1 8 1 1 1195 0468 1

8 751 2 32 1 2 1090 0568 2

8 752 1 8 1 1 1038 0568 2

8 753 2 16 1 2 1359 0618 2

8 754 2 32 1 2 900 0518 2

8 755 2 32 1 2 817 0568 2

8 756 2 8 1 2 787 0468 2

8 757 2 32 1 2 918 0668 2

8 758 2 8 1 2 686 0668 1

8 759 2 16 1 2 690 0618 2

8 760 2 32 1 2 850 0468 2

8 761 1 32 0 1 810 0468 1

8 762 2 16 1 2 786 0568 2

8 763 2 32 1 2 768 0518 2

8 764 2 32 1 2 630 0618 2

8 765 1 16 1 1 1419 0568 1

8 766 2 32 1 2 2759 0618 2

8 767 1 32 1 1 811 0568 1

8 768 2 16 1 2 840 0568 2

8 769 2 16 1 2 1323 0568 2

8 770 2 32 1 2 914 0518 2

8 771 2 8 1 2 704 0668 2

8 772 1 16 1 1 872 0668 1

8 773 2 16 1 2 1402 0618 2

8 774 2 16 1 2 1293 0518 2

8 775 2 8 1 2 713 0468 2

8 776 1 32 1 1 1202 0668 1

8 777 1 32 1 1 855 0518 1

8 778 1 32 1 1 702 0668 1

8 779 2 16 1 2 984 0568 2

8 780 2 32 1 2 979 0568 2

8 781 2 32 1 2 1074 0668 2

8 782 1 16 1 1 3561 0668 1

8 783 2 16 1 2 762 0668 2

8 784 2 16 1 2 1257 0518 2

8 785 1 32 1 1 865 0518 1

8 786 2 16 1 2 971 0518 2

8 787 2 16 1 2 1832 0618 2

8 788 1 32 1 1 935 0518 1

8 789 1 32 1 1 1417 0568 1

8 790 1 32 1 1 1932 0568 1

8 791 1 32 1 1 4600 0618 1

8 792 2 32 1 2 816 0568 2

8 793 1 8 1 1 807 0518 2

8 794 2 32 1 2 884 0518 2

8 795 1 16 1 1 951 0518 1

8 796 2 32 1 2 761 0668 2

8 797 1 16 1 1 945 0518 1

8 798 1 32 1 1 864 0618 1

8 799 1 8 1 1 766 0568 1

8 800 1 16 1 1 1175 0618 1

8 801 2 8 1 2 737 0568 1

8 802 2 32 1 2 1199 0618 2

8 803 1 8 1 1 798 0618 1

8 804 1 8 1 1 759 0618 1

8 805 1 8 1 1 637 0668 2

8 806 1 16 1 1 736 0468 1

8 807 1 8 1 1 901 0568 1

8 808 2 32 1 2 929 0468 2

8 809 1 32 1 1 893 0518 1

8 810 1 32 1 1 714 0468 1

8 811 2 8 1 2 700 0668 1

8 812 2 32 1 2 810 0668 2

8 813 1 32 1 1 923 0568 1

8 814 1 32 1 1 784 0468 1

8 815 1 8 1 1 1342 0468 1

8 816 2 8 1 2 1011 0568 2

8 817 1 8 1 1 1036 0518 2

8 818 2 32 1 2 1109 0518 2

8 819 1 8 1 1 1119 0568 2

8 820 2 32 1 2 862 0468 2

8 821 2 8 1 2 966 0468 2

8 822 1 32 0 1 1307 0668 1

8 823 1 32 1 1 4115 0618 1

8 824 2 8 1 2 841 0568 1

8 825 1 32 1 1 746 0568 1

8 826 2 16 1 2 1104 0468 2

8 827 1 32 1 1 657 0618 1

8 828 1 8 1 1 1005 0618 1

8 829 2 32 1 2 1018 0618 2

8 830 2 8 1 2 1053 0518 2

8 831 1 16 1 1 1003 0618 1

8 832 1 8 1 1 842 0668 1

8 833 1 8 1 1 669 0468 1

8 834 1 8 1 1 4354 0468 1

8 835 1 16 1 1 745 0518 1

8 836 1 8 1 1 785 0518 1

8 837 1 16 1 1 792 0568 1

8 838 1 8 1 1 4381 0518 1

8 839 2 32 1 2 1067 0468 2

8 840 2 16 1 2 835 0468 2

8 841 1 16 1 1 1149 0568 1

8 842 1 16 1 1 769 0618 1

8 843 1 8 1 1 695 0568 1

8 844 2 32 1 2 821 0518 2

8 845 1 16 1 1 685 0668 1

8 846 2 16 1 2 695 0668 2

8 847 1 16 1 1 1378 0468 1

8 848 2 8 1 2 633 0618 2

8 849 2 8 0 2 716 0568 2

8 850 2 32 1 2 866 0618 2

8 851 1 32 1 1 1932 0668 1

8 852 2 32 1 2 845 0668 2

8 853 1 8 1 1 996 0468 1

8 854 2 8 1 2 787 0668 2

8 855 1 16 1 1 1096 0668 1

8 856 2 16 1 2 894 0568 2

8 857 1 8 1 1 923 0618 1

8 858 1 32 1 1 982 0468 1

8 859 2 8 1 2 741 0568 2

8 860 2 16 1 2 712 0668 2

8 861 1 8 1 1 1048 0618 1

8 862 2 32 1 2 2419 0468 2

8 863 2 16 1 2 1386 0618 2

8 864 2 32 1 2 1047 0668 2

8 865 2 16 1 2 1215 0468 2

8 866 1 32 1 1 858 0618 1

8 867 2 8 1 2 879 0518 2

8 868 2 16 1 2 780 0518 2

8 869 2 32 1 2 710 0668 2

8 870 2 8 1 2 647 0668 2

8 871 1 32 1 1 923 0568 1

8 872 2 8 1 2 774 0618 2

8 873 1 16 1 1 1721 0568 1

8 874 1 32 1 1 863 0618 1

8 875 1 32 1 1 1222 0568 1

8 876 1 16 0 1 1058 0468 1

8 877 2 16 1 2 1119 0468 2

8 878 2 16 1 2 1157 0518 2

8 879 1 8 1 1 3045 0468 1

8 880 1 16 1 1 911 0618 1

8 881 2 8 1 2 1033 0518 2

8 882 2 16 1 2 718 0668 2

8 883 1 8 1 1 1064 0618 1

8 884 2 32 1 2 802 0568 2

8 885 1 32 1 1 848 0668 1

8 886 1 16 0 1 1154 0468 1

8 887 2 8 1 2 773 0618 2

8 888 1 16 1 1 884 0618 1

8 889 2 32 1 2 722 0568 2

8 890 1 16 0 1 1374 0468 1

8 891 1 8 1 1 806 0568 1

8 892 2 16 0 2 900 0518 2

8 893 2 8 1 2 783 0568 2

8 894 1 16 1 1 1017 0568 1

8 895 1 16 1 1 909 0668 1

8 896 1 8 1 1 698 0668 2

8 897 1 8 1 1 1241 0518 2

8 898 1 32 1 1 778 0618 1

8 899 1 8 1 1 2924 0668 2

8 900 2 16 1 2 854 0668 2

8 901 1 8 1 1 654 0568 2

8 902 2 8 1 2 669 0668 2

8 903 2 16 0 2 802 0618 2

8 904 1 16 1 1 1049 0518 1

8 905 2 16 1 2 862 0618 2

8 906 1 8 1 1 948 0618 1

8 907 2 16 1 2 1124 0468 2

8 908 2 16 1 2 1837 0468 2

8 909 1 8 1 1 716 0668 1

8 910 1 8 1 1 928 0668 1

8 911 1 32 0 1 938 0468 1

8 912 1 32 1 1 1007 0468 1

8 913 1 16 1 1 724 0668 1

8 914 2 32 1 2 1083 0618 2

8 915 2 32 1 2 823 0518 2

8 916 1 16 1 1 707 0468 1

8 917 1 32 1 1 1798 0668 1

8 918 2 16 1 2 831 0668 2

8 919 2 8 1 2 1203 0518 1

8 920 2 8 1 2 1016 0668 2

8 921 1 8 1 1 878 0618 1

8 922 2 8 1 2 787 0568 2

8 923 2 8 1 2 812 0468 2

8 924 1 32 1 1 771 0668 1

8 925 2 32 1 2 708 0618 2

8 926 2 16 1 2 895 0618 2

8 927 1 8 1 1 1658 0518 2

8 928 1 32 1 1 2013 0518 1

8 929 2 8 1 2 781 0618 2

8 930 1 8 1 1 2526 0568 2

8 931 1 32 1 1 1868 0518 1

8 932 1 8 1 1 1044 0518 1

8 933 2 16 1 2 4405 0518 2

8 934 2 8 1 2 1044 0468 2

8 935 2 32 1 2 1524 0518 2

8 936 1 32 1 1 971 0468 1

8 937 1 16 1 1 1046 0568 1

8 938 2 32 1 2 1326 0468 2

8 939 2 32 1 2 875 0568 2

8 940 1 8 1 1 1166 0468 1

8 941 1 32 1 1 1015 0518 1

8 942 1 16 1 1 1028 0618 1

8 943 2 8 1 2 956 0468 2

8 944 2 8 1 2 1592 0618 2

8 945 2 8 1 2 2039 0618 2

8 946 2 8 1 2 927 0568 2

8 947 2 32 1 2 907 0618 2

8 948 2 32 1 2 1711 0568 2

8 949 2 16 1 2 860 0568 2

8 950 1 16 1 1 1017 0668 1

8 951 1 32 1 1 910 0568 1

8 952 1 8 1 1 894 0668 1

8 953 1 16 1 1 685 0668 1

8 954 1 32 1 1 984 0518 1

8 955 2 16 1 2 976 0568 2

8 956 1 16 1 1 859 0568 1

8 957 2 8 1 2 634 0668 2

8 958 2 16 1 2 813 0518 2

8 959 1 32 1 1 1004 0668 1

8 960 1 32 1 1 963 0468 1

8 961 1 32 1 1 713 0568 1

8 962 2 32 0 2 809 0668 2

8 963 2 16 1 2 1108 0468 2

8 964 2 32 1 2 1489 0518 2

8 965 1 32 1 1 907 0668 1

8 966 1 16 1 1 849 0518 1

8 967 1 8 1 1 1333 0468 1

8 968 1 16 1 1 902 0518 1

8 969 2 8 1 2 1361 0518 1

8 970 1 32 1 1 880 0468 1

8 971 1 16 1 1 931 0668 1

8 972 1 8 1 1 806 0618 1

8 973 1 32 1 1 1302 0468 1

8 974 2 8 1 2 1065 0618 2

8 975 1 32 1 1 821 0518 1

8 976 1 16 1 1 806 0618 1

8 977 2 8 1 2 986 0668 2

8 978 2 8 1 2 1161 0568 1

8 979 2 32 1 2 3899 0668 2

8 980 2 8 1 2 870 0568 1

8 981 1 16 1 1 865 0668 1

8 982 2 16 1 2 2273 0618 2

8 983 2 16 1 2 701 0568 2

8 984 2 16 1 2 1273 0518 2

8 985 2 16 1 2 708 0468 2

8 986 2 32 1 2 1060 0568 2

8 987 2 32 1 2 870 0618 2

8 988 1 32 1 1 770 0568 1

8 989 2 32 1 2 754 0618 2

8 990 2 8 1 2 694 0568 2

8 991 1 16 0 1 839 0568 1

8 992 2 8 1 2 1289 0468 2

8 993 1 32 1 1 821 0618 1

8 994 2 16 1 2 680 0668 2

8 995 1 8 1 1 710 0568 1

8 996 1 32 1 1 763 0618 1

8 997 1 32 1 1 898 0668 1

8 998 2 16 1 2 811 0568 2

8 999 1 8 1 1 3354 0618 1

8 1000 1 8 0 1 719 0668 2

8 1001 1 8 1 1 879 0618 1

8 1002 1 8 1 1 796 0568 2

8 1003 1 32 1 1 848 0568 1

8 1004 1 32 1 1 1062 0668 1

8 1005 1 8 1 1 739 0668 1

8 1006 1 16 1 1 1585 0568 1

8 1007 1 32 1 1 727 0618 1

8 1008 1 16 1 1 647 0668 1

8 1009 2 32 1 2 2142 0668 2

8 1010 1 8 1 1 1006 0518 2

8 1011 1 8 1 1 1088 0618 1

8 1012 2 32 1 2 820 0668 2

8 1013 1 16 1 1 860 0518 1

8 1014 1 8 1 1 881 0518 1

8 1015 1 16 0 1 1169 0518 1

8 1016 2 16 1 2 1133 0468 2

8 1017 2 8 1 2 949 0468 2

8 1018 2 16 1 2 1194 0518 2

8 1019 1 16 1 1 781 0568 1

8 1020 1 32 1 1 1211 0518 1

8 1021 1 32 1 1 805 0568 1

8 1022 1 8 1 1 860 0518 1

8 1023 1 8 1 1 1401 0468 1

8 1024 2 32 1 2 866 0618 2

8 1025 1 16 0 1 1257 0468 1

8 1026 2 32 1 2 853 0568 2

8 1027 2 8 1 2 819 0468 2

8 1028 1 32 1 1 1004 0668 1

8 1029 2 8 1 2 743 0618 2

8 1030 1 8 1 1 936 0568 1

8 1031 1 16 1 1 1195 0468 1

8 1032 1 8 1 1 834 0618 1

8 1033 2 16 0 2 882 0468 2

8 1034 2 16 1 2 1121 0618 2

8 1035 1 32 1 1 1323 0568 1

8 1036 1 32 1 1 748 0468 1

8 1037 1 8 1 1 772 0568 2

8 1038 2 8 1 2 1036 0468 2

8 1039 2 8 1 2 694 0668 1

8 1040 2 8 1 2 692 0618 2

8 1041 1 16 1 1 822 0568 1

8 1042 2 32 1 2 958 0518 2

8 1043 1 32 1 1 1053 0518 1

8 1044 1 8 1 1 1957 0468 1

8 1045 2 16 1 2 1186 0618 2

8 1046 1 8 0 1 954 0468 1

8 1047 1 8 0 1 873 0468 1

8 1048 1 16 1 1 1388 0568 1

8 1049 2 32 1 2 915 0518 2

8 1050 1 32 1 1 1007 0618 1

8 1051 2 8 1 2 793 0618 2

8 1052 2 8 1 2 958 0568 2

8 1053 1 16 1 1 942 0468 1

8 1054 1 8 1 1 800 0568 1

8 1055 2 16 1 2 1009 0518 2

8 1056 1 32 1 1 804 0468 1

8 1057 1 16 1 1 1119 0468 1

8 1058 2 8 1 2 810 0468 2

8 1059 1 8 1 1 1008 0518 2

8 1060 2 16 1 2 926 0618 2

8 1061 2 32 0 2 1058 0468 2

8 1062 2 16 1 2 722 0668 2

8 1063 2 8 1 2 1171 0618 2

8 1064 2 8 1 2 817 0518 1

8 1065 2 32 1 2 921 0668 2

8 1066 2 8 1 2 1051 0468 2

8 1067 1 8 1 1 984 0468 1

8 1068 2 8 1 2 904 0568 2

8 1069 2 32 1 2 707 0568 2

8 1070 1 16 1 1 1169 0518 1

8 1071 2 16 1 2 693 0668 2

8 1072 2 8 1 2 677 0568 2

8 1073 1 16 1 1 667 0668 1

8 1074 1 8 1 1 913 0668 1

8 1075 1 32 1 1 989 0668 1

8 1076 2 32 1 2 1053 0468 2

8 1077 2 32 1 2 714 0568 2

8 1078 1 32 1 1 880 0618 1

8 1079 2 8 1 2 801 0518 1

8 1080 1 8 1 1 1162 0618 1

8 1081 2 8 1 2 614 0668 2

8 1082 1 32 1 1 910 0518 1

8 1083 1 8 1 1 747 0568 2

8 1084 1 16 1 1 714 0668 1

8 1085 1 8 1 1 614 0668 2

8 1086 2 8 0 2 946 0618 2

8 1087 1 16 1 1 668 0668 1

8 1088 1 8 1 1 996 0618 1

8 1089 2 16 0 2 930 0518 2

8 1090 2 16 1 2 708 0668 2

8 1091 2 8 1 2 549 0668 2

8 1092 2 16 1 2 570 0668 2

8 1093 1 32 1 1 1349 0468 1

8 1094 2 32 1 2 881 0468 2

8 1095 2 32 1 2 702 0618 2

8 1096 1 16 1 1 1032 0568 1

8 1097 2 8 1 2 733 0518 2

8 1098 1 16 1 1 1360 0618 1

8 1099 1 8 1 1 1459 0618 1

8 1100 2 8 1 2 732 0518 1

8 1101 1 32 1 1 722 0568 1

8 1102 1 8 1 1 758 0568 2

8 1103 1 8 1 1 793 0468 1

8 1104 2 8 1 2 747 0668 2

8 1105 1 32 1 1 4591 0618 1

8 1106 2 32 1 2 986 0518 2

8 1107 1 32 1 1 715 0518 1

8 1108 2 16 0 2 944 0518 2

8 1109 2 8 1 2 687 0468 2

8 1110 1 32 1 1 833 0668 1

8 1111 2 16 1 2 1465 0468 2

8 1112 2 8 1 2 788 0618 2

8 1113 2 32 1 2 841 0618 2

8 1114 1 32 1 1 736 0518 1

8 1115 2 32 1 2 780 0518 2

8 1116 2 32 1 2 1011 0468 2

8 1117 1 32 1 1 3801 0668 1

8 1118 2 16 1 2 1284 0518 2

8 1119 2 8 1 2 877 0518 2

8 1120 2 32 1 2 977 0568 2

8 1121 2 32 1 2 1678 0568 2

8 1122 2 16 1 2 1029 0468 2

8 1123 2 16 1 2 753 0668 2

8 1124 1 16 1 1 756 0518 1

8 1125 1 16 1 1 1281 0618 1

8 1126 2 8 1 2 609 0518 2

8 1127 2 32 1 2 921 0668 2

8 1128 1 8 1 1 733 0518 1

8 1129 2 16 1 2 693 0618 2

8 1130 1 8 1 1 633 0518 2

8 1131 1 16 1 1 1733 0618 1

8 1132 1 8 0 1 720 0518 2

8 1133 2 16 1 2 638 0518 2

8 1134 2 16 1 2 1018 0618 2

8 1135 2 8 1 2 687 0568 2

8 1136 2 8 1 2 627 0668 2

8 1137 2 32 1 2 710 0518 2

8 1138 2 8 1 2 2998 0518 2

8 1139 2 32 1 2 603 0618 2

8 1140 1 16 1 1 1145 0518 1

8 1141 1 16 1 1 802 0468 1

8 1142 1 32 1 1 949 0618 1

8 1143 2 32 1 2 942 0468 2

8 1144 1 8 1 1 680 0668 2

8 1145 1 32 1 1 942 0618 1

8 1146 2 8 1 2 1052 0668 2

8 1147 2 32 1 2 1112 0668 2

8 1148 1 16 1 1 963 0518 1

8 1149 2 16 1 2 682 0668 2

8 1150 2 16 0 2 848 0618 2

8 1151 2 16 1 2 857 0468 2

8 1152 2 32 1 2 1184 0468 2

8 1153 2 8 1 2 1339 0468 2

8 1154 1 8 1 1 1271 0518 1

8 1155 1 32 1 1 918 0468 1

8 1156 1 16 0 1 1535 0468 1

8 1157 2 8 1 2 648 0568 2

8 1158 1 32 1 1 780 0668 1

8 1159 2 32 1 2 801 0568 2

8 1160 1 16 1 1 1322 0618 1

8 1161 1 16 1 1 776 0668 1

8 1162 1 8 1 1 953 0468 1

8 1163 2 16 1 2 1521 0568 2

8 1164 2 16 1 2 761 0568 2

8 1165 1 32 1 1 736 0468 1

8 1166 2 16 1 2 807 0468 2

8 1167 2 32 1 2 965 0468 2

8 1168 2 32 1 2 788 0568 2

8 1169 1 32 1 1 707 0568 1

8 1170 1 16 1 1 1425 0618 1

8 1171 1 8 1 1 742 0568 1

8 1172 1 16 1 1 1836 0468 1

8 1173 2 32 1 2 688 0518 2

8 1174 1 16 1 1 879 0568 1

8 1175 1 16 0 1 1038 0468 1

8 1176 1 32 1 1 799 0518 1

8 1177 1 16 1 1 1483 0618 1

8 1178 1 32 1 1 612 0568 1

8 1179 2 8 1 2 701 0668 1

8 1180 2 32 1 2 1962 0518 2

8 1181 1 8 1 1 956 0668 2

8 1182 2 16 1 2 1030 0568 2

8 1183 1 32 1 1 1385 0468 1

8 1184 2 8 1 2 742 0618 2

8 1185 2 16 0 2 733 0618 2

8 1186 1 16 1 1 877 0668 1

8 1187 1 16 1 1 800 0618 1

8 1188 1 32 1 1 865 0518 1

8 1189 2 16 1 2 952 0568 2

8 1190 1 8 1 1 651 0668 1

8 1191 2 16 1 2 708 0568 2

8 1192 2 32 1 2 873 0618 2

8 1193 2 16 1 2 756 0518 2

8 1194 2 16 0 2 843 0568 2

8 1195 1 8 1 1 1165 0668 1

8 1196 1 16 0 1 645 0568 1

8 1197 2 32 1 2 1123 0468 2

8 1198 2 16 1 2 700 0668 2

8 1199 2 32 1 2 812 0618 2

8 1200 2 32 1 2 1058 0668 2

Key (relevant columns):

Column 1: Participant number

Column 2: Trial number

Column 4: Pattern size (number of sections)

Column 5: Error (0) or correct response (1)

Column 7: Reaction time (milliseconds)

Column 8: Ratio (1) 1:1.468 (2) 1:1.518 (3) 1:1.568 (4) 1:1.618 (5) 1:1.668
