# Supplementary material for: The Golden Section as Optical Limitation
Source: PLoS One. 2015 Jul 8;10(7):e0131045. doi: 10.1371/journal.pone.0131045 (PMC4495923; doi:10.1371/journal.pone.0131045)
Supplement: S3 Dataset — (DOCX) [file pone.0131045.s003.docx]

**Dataset S3. Mean Reaction Times Experiment 3**

| **C1** | **C2** | **C3** | **C4** | **C5** | **S** |
| --- | --- | --- | --- | --- | --- |
| 1220.74 | 1030.19 | 911.05 | 746.22 | 780.18 | 1 |
| 1564.27 | 1220.35 | 1130.92 | 894.91 | 923.79 | 2 |
| 2304.16 | 1715.57 | 1280.25 | 992.27 | 1057.13 | 3 |
| 1535.64 | 1374.51 | 1201.72 | 960.66 | 1017.51 | 4 |
| 1531.85 | 1333.30 | 1054.96 | 886.61 | 949.06 | 5 |
| 1368.46 | 1185.33 | 899.16 | 704.17 | 746.38 | 6 |
| 1660.58 | 1458.17 | 1351.71 | 1186.04 | 1237.57 | 7 |
| 1093.44 | 1031.67 | 998.08 | 765.99 | 789.83 | 8 |
| 1308.42 | 1155.26 | 1069.33 | 957.47 | 949.81 | 9 |
| 1481.96 | 1203.19 | 1084.57 | 854.83 | 939.17 | 10 |

Key Row 1:

C1 8-paired sections 1:1.468 ratio

C2 8-paired sections 1:1.518 ratio

C3 8 paired sections 1:1.568 ratio

C4 8-paired sections 1:1.618 ratio

C5 8-paired sections 1:1.668 ratio

S = Participant number

12 1104 1 2

12 1184 1 3

12 1829 1 2

12 1723 1 1

12 929 1 1

12 722 1 4

12 996 1 3

12 837 1 2

12 744 1 5

12 908 1 5

12 826 1 5

12 891 1 5

12 2230 1 1

12 1193 1 3

12 1350 1 1

12 649 1 4

12 1266 1 1

12 809 1 3

12 569 1 4

12 751 1 4

12 1015 0 2

12 1154 1 2

12 831 1 1

12 643 1 4

12 904 1 5

12 2222 1 5

12 1510 0 3

12 856 1 4

12 872 0 1

12 795 1 3

12 680 0 2

12 981 1 3

12 922 0 2

12 827 1 4

12 747 1 5

12 867 0 1

12 865 1 3

12 1302 1 5

12 678 1 2

12 910 1 4

12 866 1 1

12 749 1 2

12 659 1 4

12 658 1 4

12 772 1 4

12 991 1 4

12 769 1 3

12 745 1 5

12 565 1 5

12 780 1 4

12 1108 1 1

12 831 1 5

12 793 1 3

12 963 1 2

12 705 0 1

12 571 0 4

12 881 1 1

12 691 1 3

12 700 1 5

12 1323 1 4

12 831 1 3

12 647 1 5

12 883 1 3

12 604 1 2

12 831 1 3

12 529 1 2

12 573 1 2

12 521 0 2

12 1369 1 1

12 745 0 2

12 691 1 5

12 966 1 1

12 613 1 5

12 839 1 1

12 1139 0 2

12 561 0 4

12 1477 0 3

12 930 1 3

12 1277 1 1

12 691 1 5

12 1762 0 1

12 528 1 5

12 718 1 4

12 1137 1 2

12 774 1 5

12 628 0 3

12 1548 1 1

12 634 1 4

12 1011 1 2

12 1324 1 1

12 656 1 2

12 663 0 5

12 727 1 4

12 747 1 3

12 663 1 2

12 663 1 5

12 928 0 4

12 1052 1 5

12 1358 1 1

12 611 1 4

12 782 1 3

12 1312 1 2

12 704 1 3

12 1001 1 3

12 960 1 3

12 683 1 4

12 781 1 5

12 1147 1 2

12 1370 1 1

12 1935 1 1

12 878 1 3

12 1013 1 1

12 1047 1 3

12 795 1 5

12 525 1 4

12 834 1 5

12 1186 0 1

12 1209 1 2

12 1195 1 2

12 737 1 4

12 568 1 5

12 609 1 4

12 657 1 3

12 571 1 5

12 1010 1 2

12 788 1 4

12 831 0 3

12 1062 1 4

12 703 1 4

12 1095 0 1

12 919 0 1

12 742 1 1

12 911 1 2

12 1545 1 3

12 1372 1 2

12 738 1 5

12 1275 1 1

12 612 0 2

12 574 1 3

12 832 1 2

12 655 0 2

12 709 0 1

12 1184 1 2

12 803 1 5

12 1147 1 1

12 1069 1 1

12 920 1 5

12 828 1 5

12 1103 1 3

12 699 1 4

12 598 1 5

12 740 1 3

12 874 0 2

12 1160 0 1

12 664 0 4

12 894 1 3

12 794 1 4

12 969 0 4

12 1247 1 3

12 782 1 5

12 838 1 4

12 650 1 4

12 617 1 5

12 779 1 4

12 615 1 3

12 839 1 3

12 2027 1 1

12 1004 1 2

12 1294 1 1

12 1102 1 3

12 1047 1 2

12 703 1 5

12 1059 1 2

12 1012 0 1

12 1008 1 1

12 879 1 2

12 703 1 4

12 843 1 5

12 872 1 2

12 569 1 5

12 828 0 2

12 802 1 4

12 937 1 3

12 1095 1 2

12 880 1 5

12 603 1 3

12 957 1 1

12 699 1 3

12 1133 1 3

12 1011 1 4

12 836 1 5

12 879 1 4

12 1103 1 1

12 607 1 4

12 1408 1 2

12 1144 1 1

12 1149 1 3

12 956 1 5

12 1274 1 5

12 1142 1 1

12 1400 1 2

12 931 1 2

12 902 1 5

12 1006 1 2

12 1146 1 3

12 795 1 3

12 2073 1 1

12 786 1 4

12 1245 1 3

12 809 1 5

12 923 1 1

12 919 1 3

12 960 1 5

12 569 1 5

12 739 1 3

12 610 1 4

12 1133 1 2

12 1810 1 1

12 683 1 4

12 839 1 3

12 1537 1 2

12 607 1 4

12 702 1 2

12 558 1 4

12 479 1 5

12 1191 1 2

12 1494 1 2

12 964 1 3

12 840 1 4

12 628 1 4

12 961 1 1

12 1142 1 1

12 1144 1 1

12 969 1 5

12 728 1 4

12 1588 1 1

12 697 1 5

12 573 1 5

12 610 1 3

12 1104 1 1

12 922 1 3

12 920 1 1

12 873 1 1

12 1052 1 3

12 735 1 5

12 606 1 5

12 750 1 4

12 1401 1 1

12 914 1 5

12 572 1 4

12 743 1 5

12 612 1 5

12 602 1 4

12 782 1 4

12 605 1 5

12 1804 1 2

12 1537 1 2

12 1136 1 2

12 815 1 3

12 1535 1 1

12 1155 1 5

12 1794 1 2

12 615 1 4

12 1133 1 2

12 1180 1 3

12 1148 1 1

12 615 1 5

12 883 1 3

12 929 1 1

12 1047 1 1

12 1714 1 3

12 656 1 2

12 875 1 3

12 974 1 4

12 1110 1 3

12 1050 1 2

12 603 0 2

12 603 1 4

12 607 1 4

12 1142 1 1

12 875 1 3

12 959 1 3

12 702 1 5

12 957 1 5

12 831 1 5

12 529 1 4

12 870 0 2

12 1501 1 1

12 1579 1 1

12 743 1 4

12 743 1 5

12 879 1 4

12 918 0 1

12 760 1 4

12 1188 1 3

12 1183 1 2

12 914 0 2

12 827 1 4

12 958 1 5

12 921 1 2

12 1412 1 1

12 1931 1 1

12 801 1 3

12 733 1 4

12 912 1 3

12 792 1 4

12 1144 1 1

12 1278 1 3

12 1375 1 3

12 792 1 2

12 1012 1 2

12 1304 1 1

12 702 0 3

12 744 1 5

12 792 1 2

12 834 0 5

12 1060 1 1

12 725 1 5

12 1319 1 2

12 739 1 4

12 953 1 3

12 814 1 3

12 749 1 4

12 656 1 4

12 787 1 1

12 607 1 4

12 699 1 5

12 913 1 5

12 1058 0 2

12 1811 1 1

12 1012 1 2

12 794 1 3

12 1103 1 4

12 828 1 5

12 659 1 4

12 922 1 1

12 657 1 5

12 916 1 2

12 693 1 4

12 2075 0 1

12 915 1 5

12 693 1 4

12 750 1 5

12 823 1 3

12 743 1 3

12 1321 1 2

12 1184 1 1

12 561 1 2

12 1145 1 5

12 1080 1 3

12 1182 1 2

12 1573 1 2

12 820 1 3

12 704 1 4

12 2024 1 1

12 612 1 2

12 873 1 5

12 793 1 3

12 1048 1 1

12 779 0 1

12 995 1 2

12 1147 1 2

12 2362 1 1

12 1059 1 5

12 834 1 1

12 1360 1 1

12 880 1 1

12 521 1 5

12 1016 1 3

12 913 1 4

12 1056 1 3

12 3089 1 1

12 787 1 4

12 920 1 4

12 1386 1 2

12 697 1 5

12 926 1 4

12 2384 1 4

12 964 1 1

12 871 1 3

12 920 1 5

12 1012 1 1

12 836 1 3

12 961 1 3

12 824 1 1

12 872 1 3

12 1012 1 2

12 740 1 5

12 1185 1 2

12 879 1 5

12 881 1 4

12 1089 1 2

12 742 1 5

12 929 1 3

12 705 1 4

12 1500 1 2

12 825 1 2

12 897 1 5

12 642 1 4

12 834 1 3

13 2380 1 2

13 2914 1 1

13 3556 1 2

13 1918 1 3

13 1756 1 3

13 1206 1 5

13 3311 1 1

13 1006 1 4

13 1054 1 4

13 2936 1 1

13 1262 1 5

13 1973 1 2

13 1567 1 3

13 2728 1 4

13 1335 1 5

13 1984 1 3

13 1526 1 2

13 1389 1 4

13 1717 1 2

13 1500 1 1

13 1397 0 2

13 1036 1 4

13 1696 1 1

13 4180 1 3

13 3321 1 4

13 1368 1 3

13 1671 1 3

13 1286 1 3

13 1126 1 5

13 1261 1 5

13 1648 1 1

13 1235 1 5

13 1446 1 5

13 1098 1 4

13 972 1 4

13 1432 1 5

13 2473 1 1

13 2026 1 1

13 1581 1 2

13 1402 1 2

13 2104 1 3

13 1232 1 2

13 1716 1 2

13 1591 1 2

13 1458 1 2

13 966 1 5

13 785 1 4

13 1897 1 1

13 794 1 5

13 1764 1 4

13 2079 1 5

13 964 1 3

13 1016 1 4

13 1621 1 3

13 1094 1 3

13 1137 1 1

13 1187 1 1

13 829 1 4

13 966 1 4

13 1097 1 2

13 1457 1 1

13 1050 1 5

13 782 1 3

13 1408 1 1

13 832 1 4

13 1727 1 1

13 1938 1 1

13 840 1 5

13 875 1 3

13 1393 1 2

13 881 1 5

13 1017 1 5

13 704 1 5

13 1377 1 3

13 1838 1 2

13 991 1 4

13 1109 1 3

13 1187 1 2

13 1278 1 1

13 2160 1 4

13 1847 1 1

13 747 1 5

13 971 1 5

13 1367 1 2

13 1589 1 1

13 1450 1 1

13 2285 1 3

13 1141 1 4

13 1806 1 5

13 1933 1 1

13 1090 1 4

13 1141 1 3

13 1497 1 2

13 1325 1 3

13 1056 1 5

13 824 1 5

13 1724 1 3

13 1230 1 3

13 1077 1 4

13 1587 1 3

13 961 1 2

13 880 1 4

13 750 1 4

13 823 1 5

13 1172 1 2

13 875 1 5

13 4189 1 1

13 875 1 4

13 882 1 3

13 690 1 5

13 787 1 4

13 1942 1 1

13 923 1 2

13 1054 1 2

13 825 0 1

13 1026 0 3

13 902 1 4

13 1146 1 2

13 1235 1 1

13 1430 1 2

13 1217 1 3

13 1090 1 1

13 1274 1 5

13 961 1 4

13 1096 1 3

13 1225 1 3

13 1533 1 1

13 2328 1 1

13 2386 1 1

13 960 1 5

13 1365 1 2

13 1675 1 1

13 913 1 4

13 1367 1 2

13 1633 1 5

13 2514 1 2

13 1231 1 2

13 1138 1 4

13 874 1 5

13 836 1 4

13 788 1 4

13 1179 1 1

13 745 1 5

13 1713 1 2

13 976 1 3

13 1178 1 5

13 2107 1 1

13 1313 1 3

13 4077 1 1

13 787 1 4

13 751 1 4

13 1080 1 3

13 2254 1 2

13 1311 1 2

13 902 1 5

13 1136 1 2

13 1402 1 3

13 871 1 5

13 878 1 3

13 734 1 4

13 1409 1 1

13 789 1 4

13 1049 1 2

13 1799 1 3

13 1452 1 1

13 746 1 4

13 1058 1 2

13 700 1 4

13 1223 1 1

13 747 1 5

13 1402 1 2

13 839 1 3

13 1046 1 1

13 793 1 5

13 827 1 3

13 614 1 4

13 825 0 3

13 1938 1 1

13 662 1 5

13 1234 1 5

13 959 1 3

13 1543 1 2

13 923 1 5

13 1184 1 2

13 956 1 3

13 1276 1 1

13 790 1 2

13 1008 1 4

13 1886 1 1

13 924 1 5

13 705 1 5

13 918 1 3

13 693 1 4

13 824 1 4

13 965 1 3

13 690 1 5

13 1361 1 1

13 1235 1 2

13 734 1 4

13 1135 1 2

13 1309 1 2

13 1190 1 1

13 838 1 2

13 917 0 1

13 748 1 3

13 911 1 3

13 1148 1 2

13 794 1 4

13 880 1 3

13 1316 1 3

13 780 1 4

13 1280 1 1

13 911 1 2

13 836 1 5

13 702 1 4

13 1306 1 1

13 827 1 3

13 1079 1 3

13 1008 1 3

13 781 1 4

13 1224 1 1

13 1005 1 2

13 745 1 5

13 1101 1 1

13 934 1 5

13 780 1 5

13 1236 1 4

13 828 1 5

13 880 1 5

13 744 1 5

13 1181 1 2

13 867 1 4

13 1223 1 2

13 698 1 5

13 2195 1 1

13 822 1 2

13 687 1 4

13 1138 1 3

13 660 1 4

13 1852 1 1

13 669 1 4

13 1124 1 1

13 788 1 5

13 1007 1 5

13 875 1 2

13 657 1 4

13 1057 1 3

13 698 1 5

13 690 1 4

13 654 1 5

13 1187 1 1

13 1171 1 2

13 780 1 2

13 1103 1 1

13 1046 1 3

13 702 1 4

13 1093 1 2

13 1143 1 2

13 1269 1 1

13 1491 1 4

13 1280 1 1

13 836 1 4

13 1227 1 3

13 790 1 5

13 1046 1 2

13 1148 1 3

13 706 1 5

13 831 1 3

13 1148 1 1

13 831 1 5

13 1104 1 2

13 1041 1 3

13 1412 1 1

13 1405 1 1

13 923 1 3

13 976 1 4

13 904 1 3

13 916 1 4

13 739 1 5

13 1893 1 2

13 832 1 3

13 822 1 4

13 961 1 1

13 836 1 3

13 738 1 4

13 741 1 5

13 920 1 2

13 961 1 3

13 791 1 5

13 920 1 2

13 1979 1 1

13 1235 1 3

13 1371 1 3

13 1315 1 1

13 871 1 5

13 873 1 4

13 1154 1 5

13 1015 1 4

13 873 1 4

13 873 1 5

13 1273 1 2

13 916 1 2

13 1097 1 2

13 966 1 3

13 781 1 5

13 918 1 2

13 879 1 3

13 1276 1 2

13 878 1 4

13 1006 1 5

13 1360 1 1

13 846 1 4

13 861 1 5

13 614 1 4

13 883 1 2

13 1090 1 1

13 1184 1 1

13 1621 1 1

13 1227 1 3

13 1584 1 1

13 828 1 5

13 748 1 5

13 911 1 4

13 1055 1 3

13 1126 1 2

13 962 1 2

13 837 1 2

13 1463 1 1

13 1190 1 1

13 911 1 3

13 826 1 3

13 703 1 4

13 1045 1 3

13 881 1 5

13 1547 1 3

13 837 1 5

13 3095 1 1

13 795 1 4

13 1182 1 4

13 1099 1 3

13 1890 1 5

13 1449 1 3

13 1511 1 2

13 1708 1 1

13 921 1 5

13 1094 1 3

13 2085 1 1

13 683 1 2

13 1001 1 1

13 904 1 4

13 1050 1 1

13 694 1 4

13 873 1 4

13 698 1 4

13 881 1 5

13 4097 1 1

13 2024 1 2

13 1011 1 2

13 1320 1 2

13 1005 1 5

13 875 1 4

13 1676 1 1

13 834 1 1

13 1400 1 1

13 781 1 3

13 1537 1 1

13 1015 1 2

13 742 1 3

13 916 1 3

13 789 1 4

13 889 1 2

13 735 0 4

13 891 1 3

13 826 1 5

13 619 1 4

13 1726 1 1

13 1040 1 2

13 1179 1 2

13 798 1 5

13 962 1 5

13 1085 1 2

13 966 1 2

13 1045 1 1

13 1192 1 2

13 833 1 5

13 912 1 3

13 1058 1 1

13 1177 0 1

13 856 1 4

13 651 1 4

13 955 1 2

13 831 1 3

13 823 1 4

13 971 1 5

13 1224 1 3

13 1018 1 5

13 1001 1 3

13 828 1 4

13 832 1 5

13 868 1 5

14 6719 1 1

14 1590 1 5

14 1943 1 2

14 4102 0 1

14 1132 1 4

14 3346 0 1

14 1833 1 3

14 1267 1 4

14 1250 1 4

14 1223 1 3

14 1318 1 5

14 1755 1 2

14 1450 1 3

14 1577 1 3

14 1362 1 2

14 1147 1 5

14 6280 1 1

14 1622 1 4

14 3715 1 1

14 2024 1 2

14 2700 1 1

14 1135 1 5

14 1800 1 2

14 2640 1 1

14 1987 1 5

14 1273 1 5

14 962 1 4

14 1061 1 5

14 1496 1 2

14 1044 1 4

14 1275 1 3

14 1098 1 4

14 1281 1 3

14 2645 0 1

14 2469 1 3

14 2421 1 2

14 1400 1 2

14 1094 1 4

14 1050 1 3

14 914 1 5

14 2252 1 5

14 2731 1 2

14 1547 1 4

14 1414 1 4

14 3314 1 2

14 4319 1 1

14 1226 1 2

14 1586 1 3

14 1226 1 3

14 4375 0 1

14 1005 1 5

14 2688 0 1

14 1090 1 4

14 2820 1 1

14 2800 1 2

14 1139 1 5

14 1326 1 5

14 1048 1 4

14 1370 1 4

14 1314 1 5

14 1804 1 3

14 1227 1 2

14 2120 1 3

14 3793 0 1

14 1360 1 3

14 1129 1 3

14 1058 1 5

14 4902 1 1

14 2952 1 2

14 956 1 5

14 2158 1 1

14 1143 1 5

14 881 1 4

14 662 1 4

14 1452 1 2

14 3394 1 3

14 959 1 4

14 1002 1 3

14 3257 1 1

14 2205 1 2

14 2201 1 1

14 1180 1 3

14 3093 1 1

14 970 1 5

14 1118 1 3

14 1273 1 2

14 831 1 5

14 1901 1 3

14 1626 0 2

14 985 1 4

14 1424 1 5

14 3568 1 2

14 1490 1 2

14 1009 1 4

14 956 1 4

14 3574 1 1

14 3267 0 1

14 1223 1 4

14 2830 1 2

14 1104 1 5

14 1059 1 5

14 1895 1 3

14 1191 1 3

14 1009 1 3

14 1276 1 3

14 1187 1 1

14 873 1 4

14 2601 1 2

14 2118 0 1

14 1291 1 5

14 2071 1 1

14 1887 1 2

14 970 0 4

14 2115 1 2

14 2773 1 4

14 1228 1 5

14 8403 0 1

14 1267 1 3

14 824 1 4

14 1117 1 5

14 1061 1 3

14 958 1 5

14 3488 1 2

14 834 1 4

14 1006 1 3

14 875 1 4

14 1585 1 2

14 1578 1 3

14 3801 1 1

14 6800 1 1

14 1193 1 3

14 824 1 4

14 1277 1 3

14 1501 1 5

14 1056 1 4

14 2732 0 1

14 1930 1 1

14 1502 1 5

14 915 1 5

14 823 1 5

14 6008 0 1

14 728 1 4

14 1190 1 5

14 1762 1 3

14 3489 0 1

14 869 1 5

14 1181 1 3

14 2064 1 1

14 3191 1 2

14 956 1 4

14 1406 0 2

14 1294 1 4

14 1458 1 2

14 890 1 2

14 1937 1 3

14 1415 1 2

14 3484 0 1

14 894 1 4

14 833 1 5

14 3130 1 2

14 1177 1 5

14 617 1 5

14 2421 1 1

14 832 1 4

14 1353 1 3

14 1177 1 5

14 876 1 5

14 4022 1 3

14 1671 1 5

14 1184 1 4

14 5471 1 1

14 1362 1 2

14 1362 1 3

14 1368 1 1

14 6545 0 1

14 932 1 4

14 2681 1 2

14 2387 1 2

14 922 1 3

14 1180 1 4

14 1326 1 4

14 964 1 4

14 1177 1 3

14 4728 1 1

14 1587 1 2

14 1759 1 2

14 3576 0 1

14 1106 1 5

14 1140 1 5

14 1318 1 3

14 1091 1 2

14 1266 1 3

14 1664 1 2

14 2081 1 1

14 911 1 4

14 1316 1 4

14 3132 0 1

14 1508 1 3

14 794 1 5

14 1357 1 2

14 1183 1 4

14 3124 1 2

14 1187 1 2

14 3217 0 1

14 793 1 4

14 1444 1 2

14 1134 1 1

14 1584 0 1

14 923 1 5

14 1136 1 3

14 1050 1 5

14 1696 1 2

14 884 1 3

14 955 1 5

14 1591 1 2

14 790 1 5

14 882 1 4

14 1004 1 3

14 562 1 5

14 1147 1 1

14 1014 1 5

14 926 1 5

14 1191 1 3

14 1497 1 1

14 702 1 4

14 1092 1 4

14 877 1 4

14 1852 0 1

14 1104 1 4

14 4680 0 1

14 1001 1 2

14 912 1 3

14 1500 1 3

14 1046 1 2

14 1368 1 1

14 1626 1 3

14 971 1 3

14 1343 1 5

14 1544 1 2

14 919 1 4

14 1134 1 1

14 649 1 5

14 3507 0 1

14 854 1 4

14 954 1 2

14 4773 0 1

14 1358 1 1

14 1102 1 5

14 1181 1 3

14 1193 1 2

14 844 1 4

14 2948 1 3

14 999 1 3

14 824 1 5

14 655 1 4

14 1448 1 4

14 1717 1 2

14 2697 0 1

14 735 1 4

14 1135 1 2

14 3526 1 1

14 2647 1 2

14 791 1 4

14 2645 1 2

14 748 1 4

14 912 1 5

14 794 1 5

14 2418 1 5

14 827 1 5

14 1102 1 3

14 1115 1 3

14 1680 1 3

14 1670 1 4

14 1182 1 5

14 1585 1 2

14 3613 0 1

14 3591 0 1

14 1234 1 3

14 4454 1 2

14 1095 1 3

14 847 1 5

14 1626 1 2

14 1454 1 1

14 1542 0 2

14 1323 1 5

14 1048 1 4

14 2903 1 3

14 1669 1 3

14 1183 1 2

14 1102 1 2

14 1225 1 2

14 965 1 3

14 867 1 4

14 1002 1 4

14 1224 1 2

14 3708 1 2

14 2353 0 1

14 3303 1 1

14 3483 1 1

14 1002 1 5

14 959 1 4

14 922 1 5

14 3080 1 2

14 969 1 5

14 1406 1 3

14 1191 1 3

14 743 1 5

14 923 1 3

14 1229 1 1

14 947 1 5

14 994 1 5

14 1425 1 1

14 2507 0 1

14 765 1 4

14 760 1 4

14 1361 1 3

14 961 1 3

14 791 1 4

14 788 1 4

14 1845 1 1

14 749 1 4

14 1039 1 5

14 969 1 3

14 871 1 4

14 768 1 5

14 1356 1 2

14 784 1 5

14 3259 0 1

14 795 1 3

14 1316 1 2

14 3126 1 5

14 1018 1 5

14 1092 1 2

14 1369 1 2

14 694 1 4

14 1321 1 2

14 793 1 3

14 693 1 4

14 1763 1 1

14 782 1 4

14 1575 1 1

14 1184 1 3

14 2778 0 1

14 693 1 3

14 1059 1 3

14 3348 1 1

14 865 1 5

14 762 1 5

14 1362 1 1

14 1226 1 2

14 1685 1 4

14 5154 1 1

14 766 1 4

14 1094 1 5

14 1134 0 1

14 1471 1 3

14 1011 1 3

14 825 1 4

14 1272 1 2

14 1047 1 2

14 1088 1 2

14 1094 1 5

14 1721 0 1

14 3492 0 1

14 1361 1 4

14 973 1 3

14 959 1 2

14 1630 1 1

14 966 1 2

14 1107 1 2

14 1611 1 4

14 924 1 3

14 931 1 5

14 692 1 4

14 3621 1 2

14 1413 1 2

14 1236 1 5

14 1069 1 3

14 1145 1 5

14 1146 1 3

14 2097 0 1

14 916 1 4

14 1274 1 1

14 1757 1 1

14 1052 1 3

14 923 1 5

14 964 1 3

14 1237 1 1

14 1625 1 4

14 873 1 5

14 787 1 4

14 1229 1 3

14 8935 1 2

14 873 1 5

14 1101 1 5

14 913 1 3

14 1147 1 4

14 1100 1 2

14 921 1 1

14 868 1 4

15 1393 1 5

15 1811 1 3

15 3183 1 1

15 1190 1 4

15 1190 1 5

15 1237 1 5

15 2421 1 1

15 1462 1 4

15 2742 1 2

15 2607 1 3

15 1834 1 4

15 1945 1 5

15 1857 1 3

15 1038 1 3

15 2514 0 1

15 2097 1 2

15 2116 1 4

15 1976 1 5

15 1522 1 5

15 2828 1 1

15 2548 1 2

15 1474 1 5

15 3184 1 4

15 2534 1 1

15 2038 1 1

15 1083 1 2

15 1366 1 3

15 873 1 4

15 1493 1 2

15 1417 1 4

15 1038 1 2

15 2453 1 1

15 2748 1 2

15 879 1 4

15 1545 1 3

15 1397 1 5

15 1369 1 3

15 2520 1 2

15 5499 1 3

15 3029 0 1

15 3143 1 5

15 1948 1 2

15 2871 0 1

15 2464 1 4

15 2286 1 3

15 2295 1 5

15 1359 1 2

15 1283 1 3

15 914 1 5

15 751 1 4

15 917 1 5

15 1579 1 2

15 1150 1 1

15 1318 1 1

15 910 1 5

15 1047 1 3

15 922 1 5

15 1279 1 2

15 1400 1 3

15 1046 1 2

15 1032 1 1

15 1672 1 1

15 1003 1 4

15 918 1 5

15 1872 1 2

15 1185 1 4

15 1142 1 3

15 876 1 3

15 1012 1 4

15 794 1 3

15 769 1 4

15 477 1 4

15 2110 0 1

15 1716 1 1

15 2785 1 2

15 880 1 5

15 2743 1 1

15 1811 1 3

15 1147 1 4

15 2301 1 2

15 3878 1 2

15 985 1 1

15 1094 1 2

15 1234 1 1

15 1669 1 2

15 1187 1 5

15 1404 1 2

15 2114 1 3

15 2922 1 1

15 1724 1 4

15 1409 1 3

15 880 1 5

15 1055 1 5

15 1494 1 5

15 1280 1 4

15 2079 1 3

15 1184 1 4

15 1065 1 4

15 1118 1 5

15 1985 1 1

15 1048 1 2

15 2420 1 2

15 2109 0 3

15 3840 1 2

15 1054 1 5

15 1144 1 4

15 2430 1 1

15 2213 1 1

15 927 1 4

15 1766 1 1

15 982 1 3

15 978 1 3

15 964 1 3

15 1060 1 4

15 790 1 5

15 1047 1 5

15 1500 1 3

15 1226 1 1

15 739 1 4

15 902 1 2

15 1100 1 1

15 963 1 3

15 1499 1 1

15 796 1 2

15 706 1 5

15 1673 1 3

15 1029 1 2

15 863 1 5

15 2112 1 5

15 968 1 4

15 1535 1 2

15 1401 1 3

15 1226 0 2

15 1023 1 5

15 965 1 4

15 603 1 4

15 1091 1 3

15 1675 1 2

15 1188 1 4

15 1150 1 3

15 1060 1 4

15 2157 1 1

15 1054 1 4

15 1137 1 2

15 4691 1 2

15 1011 1 1

15 782 1 5

15 2159 1 1

15 701 1 5

15 1013 1 1

15 927 1 5

15 696 1 4

15 2079 1 1

15 1059 1 3

15 829 1 3

15 1147 1 2

15 1142 1 5

15 1365 1 1

15 831 1 4

15 1597 1 3

15 1278 1 5

15 1544 1 1

15 928 1 4

15 610 1 5

15 1014 1 3

15 744 1 5

15 1365 1 1

15 970 1 3

15 962 1 5

15 869 1 2

15 1049 1 2

15 1371 1 2

15 790 1 3

15 786 1 4

15 1269 1 1

15 916 1 2

15 1712 1 4

15 837 0 1

15 1184 1 2

15 1185 1 1

15 747 1 5

15 1194 1 5

15 2063 1 3

15 1633 1 1

15 1491 1 1

15 1183 1 3

15 1456 1 2

15 867 1 4

15 845 1 4

15 749 1 3

15 839 1 1

15 1230 1 3

15 1008 1 5

15 612 1 4

15 969 1 3

15 1134 1 2

15 937 1 2

15 766 1 4

15 1945 1 5

15 793 1 4

15 1984 1 1

15 744 1 2

15 926 1 5

15 3795 0 1

15 1038 1 1

15 1141 1 3

15 1281 1 2

15 1130 1 4

15 1503 1 4

15 2256 1 2

15 2169 1 3

15 916 1 3

15 914 1 5

15 1415 1 3

15 1887 1 3

15 1008 1 5

15 1588 1 2

15 1140 1 4

15 1896 0 1

15 888 1 3

15 699 1 5

15 869 1 4

15 827 1 4

15 792 1 4

15 918 0 2

15 848 1 5

15 1013 1 2

15 1936 1 1

15 827 1 5

15 1254 0 1

15 1383 0 2

15 2508 1 1

15 1232 1 4

15 1052 1 5

15 780 1 5

15 1282 1 1

15 825 1 2

15 1322 1 4

15 969 1 3

15 1194 1 3

15 1207 1 1

15 2205 1 5

15 1496 1 3

15 1358 1 2

15 1368 1 3

15 916 1 5

15 1946 1 1

15 1525 1 4

15 1311 1 1

15 1405 1 1

15 1406 1 3

15 2338 1 1

15 923 1 4

15 815 1 3

15 749 1 4

15 1228 1 1

15 2165 1 5

15 1137 1 2

15 1477 1 4

15 1321 1 2

15 874 1 4

15 1631 1 1

15 796 1 5

15 705 1 4

15 2071 1 5

15 653 1 3

15 1006 1 4

15 699 1 2

15 1311 1 3

15 1280 1 2

15 796 1 3

15 1413 1 2

15 706 1 5

15 746 1 4

15 928 1 1

15 2202 1 2

15 1359 1 3

15 751 1 5

15 1322 1 2

15 920 1 5

15 920 1 1

15 788 1 5

15 830 1 3

15 661 1 4

15 746 1 5

15 872 1 2

15 1051 1 2

15 914 1 4

15 2123 1 1

15 878 1 2

15 969 1 5

15 651 1 5

15 1271 1 3

15 1017 1 3

15 1800 1 2

15 1224 1 3

15 922 1 5

15 1274 1 2

15 1054 1 3

15 649 1 4

15 602 1 5

15 2072 0 1

15 1425 1 3

15 1985 1 1

15 2068 0 1

15 1469 1 2

15 837 1 1

15 740 1 4

15 1179 1 1

15 971 1 4

15 874 1 1

15 655 1 4

15 879 1 5

15 970 1 4

15 829 1 3

15 1944 1 2

15 872 1 4

15 880 1 2

15 661 1 5

15 1014 1 3

15 1935 1 2

15 1766 0 1

15 592 1 4

15 792 1 4

15 612 1 4

15 794 1 2

15 562 1 5

15 1229 1 5

15 2423 1 5

15 875 1 3

15 1281 1 1

15 903 1 2

15 1006 1 5

15 1319 1 1

15 1182 1 5

15 1001 1 2

15 1150 1 1

15 869 1 5

15 1093 1 4

15 663 1 4

15 838 1 3

15 1011 1 3

15 837 0 2

15 1282 1 2

15 1179 1 3

15 1186 1 1

15 1714 0 1

15 624 1 4

15 1013 1 3

15 610 1 4

15 658 1 5

15 1189 1 2

15 1406 1 3

15 563 1 4

15 1723 0 1

15 987 1 3

15 1312 1 2

15 1413 1 3

15 697 1 5

15 2832 1 1

15 829 1 3

15 1281 1 1

15 1058 1 2

15 1498 1 1

15 1365 1 1

15 744 1 2

15 615 1 4

15 663 1 5

15 1394 1 2

15 1015 1 2

15 1323 1 1

15 709 1 5

15 913 1 4

15 839 1 4

15 747 1 5

15 616 1 5

15 2111 0 1

15 975 1 2

15 1239 1 2

15 873 1 3

15 3577 1 2

15 527 0 5

15 704 1 3

15 882 1 2

15 884 1 1

15 833 1 5

15 649 1 4

15 1158 1 5

15 1363 1 5

15 1365 1 3

15 880 1 4

15 1434 1 4

15 657 1 4

15 968 1 3

15 1978 1 1

15 1447 1 3

15 1063 1 3

15 2378 1 4

15 2962 0 1

15 969 1 3

16 1812 0 1

16 1040 1 2

16 1725 1 1

16 952 1 4

16 2070 1 2

16 989 1 5

16 1189 1 5

16 1809 1 1

16 1100 1 2

16 1253 1 2

16 921 1 5

16 958 1 3

16 783 1 4

16 948 1 5

16 871 1 4

16 785 1 3

16 1609 1 2

16 1779 1 1

16 1789 1 1

16 1367 1 2

16 1410 1 2

16 1017 1 3

16 1842 1 2

16 1039 1 5

16 1047 1 4

16 2344 1 1

16 1013 1 3

16 1034 1 1

16 923 1 3

16 788 1 5

16 1231 1 3

16 1218 1 5

16 738 1 4

16 1148 1 3

16 1050 1 4

16 1135 1 5

16 4158 1 1

16 1004 1 4

16 1104 1 3

16 910 1 4

16 1357 1 4

16 1142 1 5

16 1984 1 1

16 1411 1 2

16 2465 1 2

16 1147 1 4

16 563 1 3

16 788 1 5

16 724 1 4

16 832 1 5

16 1974 1 2

16 701 1 4

16 781 1 4

16 1547 1 3

16 1459 0 3

16 2616 1 2

16 1089 1 2

16 2162 1 1

16 960 1 1

16 782 1 3

16 1003 1 4

16 898 1 4

16 1177 1 5

16 1282 1 2

16 1400 1 2

16 743 0 1

16 1106 1 5

16 1448 1 5

16 654 1 5

16 1844 1 1

16 1274 1 5

16 1628 1 1

16 879 1 3

16 1236 1 2

16 1145 1 3

16 1639 1 1

16 972 1 4

16 1096 1 3

16 1634 1 1

16 1711 1 3

16 1667 1 1

16 2066 1 1

16 1798 1 2

16 962 1 3

16 1221 1 5

16 839 1 4

16 2291 1 1

16 1763 1 2

16 1369 1 5

16 1403 1 2

16 1518 1 1

16 3437 1 4

16 1404 1 5

16 1532 1 5

16 1134 1 3

16 825 1 3

16 792 1 5

16 570 1 4

16 4146 0 1

16 1410 1 2

16 1717 1 3

16 737 1 5

16 828 1 1

16 1717 1 2

16 962 1 2

16 793 1 4

16 1030 1 1

16 794 1 5

16 1148 1 4

16 1398 1 4

16 1060 1 5

16 1322 1 4

16 2070 1 2

16 1803 1 2

16 1054 0 3

16 1621 1 1

16 1005 1 4

16 1494 1 3

16 1361 1 3

16 1205 1 3

16 832 1 5

16 1177 1 2

16 1809 1 2

16 1100 1 5

16 872 1 4

16 1189 1 2

16 1579 1 2

16 1147 1 1

16 1127 1 3

16 1060 1 2

16 1179 1 4

16 875 1 1

16 698 1 5

16 884 1 5

16 1414 1 4

16 1406 1 1

16 1669 1 1

16 1297 1 3

16 1946 1 5

16 1493 1 2

16 956 1 4

16 1451 1 3

16 835 1 3

16 865 1 4

16 1133 1 5

16 1003 1 1

16 1048 1 2

16 1133 1 3

16 1028 1 3

16 821 1 5

16 871 1 4

16 2201 1 1

16 1092 1 1

16 1498 1 3

16 2514 1 1

16 968 1 5

16 1402 1 4

16 1047 1 3

16 1272 1 2

16 962 1 4

16 1000 1 4

16 1976 1 1

16 1321 1 1

16 878 1 4

16 1005 1 1

16 786 1 5

16 703 1 5

16 747 1 5

16 749 1 3

16 514 1 5

16 1491 1 1

16 745 1 4

16 1099 1 2

16 1310 1 2

16 1323 1 1

16 868 1 5

16 1269 1 3

16 1181 1 1

16 1139 1 5

16 809 1 3

16 811 1 4

16 1629 1 2

16 882 1 5

16 1100 1 3

16 1135 1 4

16 1236 1 2

16 1443 1 2

16 879 1 4

16 1236 1 2

16 1146 1 2

16 927 1 3

16 1044 1 2

16 1982 1 1

16 910 1 5

16 1487 1 1

16 1018 1 4

16 1233 1 3

16 1326 1 3

16 1321 1 3

16 653 1 4

16 889 1 5

16 773 1 4

16 1229 1 2

16 851 1 3

16 1235 1 2

16 1181 0 1

16 1375 1 1

16 1091 1 5

16 1410 1 3

16 878 1 5

16 966 1 2

16 1012 1 3

16 786 1 4

16 1052 1 3

16 1001 1 3

16 1010 1 5

16 1018 1 5

16 2427 1 1

16 968 1 2

16 605 1 3

16 836 1 5

16 1782 1 1

16 1407 1 1

16 1103 1 2

16 740 1 4

16 1849 1 1

16 1355 0 4

16 649 1 5

16 1099 1 2

16 780 1 4

16 882 1 4

16 959 1 2

16 1496 1 1

16 962 1 3

16 690 1 4

16 567 1 4

16 1189 1 2

16 876 1 5

16 1144 1 1

16 690 1 3

16 694 1 5

16 1057 1 3

16 1274 1 1

16 1045 1 2

16 1015 1 4

16 1629 1 5

16 838 1 5

16 3973 1 1

16 1540 1 1

16 1630 1 1

16 1148 1 2

16 1101 0 3

16 1466 1 1

16 1501 1 3

16 959 1 3

16 1056 1 5

16 1230 1 2

16 735 1 3

16 916 1 3

16 514 1 4

16 641 1 4

16 659 1 4

16 834 0 1

16 957 1 5

16 965 1 4

16 963 1 5

16 1222 1 1

16 832 1 3

16 823 1 5

16 967 1 2

16 926 1 2

16 1096 1 3

16 3103 1 2

16 1127 1 2

16 1410 1 2

16 1059 1 4

16 1006 1 4

16 1454 1 1

16 778 1 5

16 659 1 4

16 1357 1 2

16 699 1 4

16 957 1 5

16 1230 1 2

16 1281 0 2

16 1094 1 3

16 1365 1 5

16 2598 1 1

16 1443 1 1

16 923 1 1

16 743 1 4

16 1675 1 2

16 833 1 4

16 791 1 3

16 967 1 4

16 957 1 4

16 1231 1 2

16 1237 1 3

16 3007 1 1

16 751 1 5

16 692 1 4

16 1371 0 1

16 1677 1 1

16 967 1 5

16 1666 1 2

16 1089 1 2

16 964 1 5

16 809 1 3

16 2072 1 1

16 1274 1 5

16 1319 1 3

16 1384 1 3

16 979 1 4

16 1144 1 4

16 1231 0 5

16 3279 1 2

16 838 1 3

16 2378 1 3

16 1023 1 5

16 1224 0 1

16 1401 1 2

16 693 1 4

16 832 1 5

16 1588 1 1

16 785 1 4

16 1180 1 4

16 1140 1 1

16 1363 1 2

16 660 1 5

16 1401 1 2

16 1933 0 1

16 891 1 4

16 1790 1 1

16 989 1 3

16 1365 1 2

16 735 1 4

16 1605 1 1

16 836 1 5

16 772 1 2

16 920 1 3

16 1357 1 1

16 2078 1 2

16 2562 1 5

16 1236 1 3

16 1146 1 2

16 959 1 3

16 1586 1 4

16 1010 1 3

16 652 1 3

16 789 1 5

16 1052 1 3

16 1446 1 3

16 1368 1 2

16 1409 1 1

16 650 1 5

16 617 1 4

16 1356 1 1

16 654 1 5

16 912 1 4

16 1053 1 5

16 1096 1 5

16 748 1 2

16 1044 1 1

16 1187 1 5

16 1226 1 2

16 1053 1 1

16 944 1 5

16 837 1 5

16 1307 1 3

16 648 1 5

16 1044 1 3

16 1146 1 2

16 662 1 4

16 623 1 3

16 999 1 4

16 647 1 5

16 1591 1 1

16 1008 1 1

16 1230 1 2

16 1497 1 3

16 1002 1 3

16 727 1 5

16 879 1 4

16 1147 1 2

16 1237 1 2

16 828 1 2

16 1367 1 2

16 646 1 4

16 732 1 3

16 690 1 1

16 1146 1 1

16 1683 1 1

16 1049 1 5

16 600 1 4

16 1136 1 3

16 783 1 4

16 1195 1 3

16 918 0 1

16 672 1 4

16 830 1 4

17 3183 1 5

17 1610 1 2

17 1278 1 4

17 828 1 5

17 2164 0 1

17 804 1 5

17 2272 1 1

17 1095 1 4

17 1324 1 2

17 925 1 5

17 1718 1 2

17 3353 1 2

17 1805 1 1

17 2285 1 2

17 1370 1 3

17 1492 1 5

17 958 1 3

17 656 1 4

17 1672 1 4

17 1184 1 3

17 1887 1 1

17 790 1 4

17 788 1 4

17 693 1 4

17 701 1 5

17 1181 1 3

17 1135 1 5

17 1269 1 2

17 1890 1 2

17 870 1 3

17 872 1 4

17 1228 1 2

17 1722 0 1

17 2030 1 1

17 1048 1 3

17 1234 0 1

17 2329 1 1

17 1225 1 3

17 2116 1 3

17 828 1 5

17 1852 1 3

17 1182 0 2

17 1348 1 1

17 1445 1 2

17 875 1 5

17 568 1 5

17 485 1 4

17 773 1 4

17 1894 1 1

17 969 1 4

17 603 1 5

17 1537 1 1

17 1234 1 2

17 739 1 5

17 1141 1 2

17 1718 1 3

17 1135 1 1

17 1049 1 2

17 1052 1 5

17 1315 1 1

17 780 1 3

17 1854 1 1

17 1181 1 2

17 1343 1 5

17 1125 1 2

17 694 1 4

17 925 1 3

17 872 1 5

17 2512 1 1

17 1091 1 3

17 1142 1 3

17 882 1 4

17 966 1 3

17 691 1 4

17 1098 1 2

17 973 1 3

17 654 1 4

17 691 1 5

17 1452 1 1

17 963 1 4

17 1055 1 2

17 1810 1 1

17 941 1 5

17 1434 1 1

17 782 1 3

17 694 1 4

17 1056 1 3

17 872 1 3

17 1892 1 1

17 654 0 4

17 1558 0 2

17 926 1 5

17 883 1 4

17 1311 1 2

17 1494 1 1

17 795 1 5

17 924 1 3

17 1045 1 2

17 2020 1 1

17 1094 1 2

17 1101 1 5

17 654 1 5

17 739 1 4

17 746 1 5

17 830 1 4

17 783 1 3

17 862 1 3

17 1279 1 1

17 1323 1 2

17 1274 1 1

17 784 1 3

17 780 1 3

17 1900 0 1

17 1011 1 2

17 747 1 5

17 678 1 4

17 1094 1 2

17 729 1 5

17 571 1 4

17 852 1 4

17 1226 1 1

17 659 1 4

17 991 1 4

17 743 1 4

17 835 1 5

17 1351 1 2

17 703 1 5

17 1009 1 5

17 607 1 5

17 743 1 3

17 1145 1 1

17 790 1 5

17 2041 1 2

17 1541 1 2

17 1011 1 3

17 744 1 5

17 1201 1 1

17 1502 1 1

17 786 1 4

17 827 1 5

17 1012 1 3

17 615 1 4

17 1538 1 1

17 718 1 3

17 1490 1 1

17 1044 1 1

17 1534 1 2

17 1089 1 2

17 1096 1 3

17 1501 0 1

17 1274 1 2

17 1363 1 2

17 775 1 5

17 1054 1 3

17 829 1 3

17 1402 1 4

17 916 1 2

17 693 1 4

17 744 1 3

17 816 1 4

17 568 1 5

17 559 1 5

17 1055 1 2

17 1979 0 1

17 640 1 4

17 1050 1 2

17 1178 1 3

17 1142 0 1

17 694 1 4

17 613 1 5

17 1454 1 2

17 1312 1 2

17 924 1 1

17 1090 1 1

17 964 1 2

17 876 1 3

17 1620 1 1

17 1227 1 1

17 835 1 4

17 1269 1 2

17 740 1 4

17 1578 1 2

17 837 1 3

17 1315 1 3

17 744 1 5

17 834 1 3

17 649 1 5

17 646 1 4

17 607 1 4

17 1052 1 2

17 861 1 3

17 972 1 1

17 786 1 3

17 701 1 4

17 876 1 5

17 568 1 4

17 606 1 5

17 689 1 5

17 1090 1 3

17 1583 1 1

17 562 1 4

17 1155 1 3

17 1042 1 1

17 871 1 1

17 922 1 3

17 734 1 5

17 646 1 5

17 881 1 2

17 689 1 4

17 1501 1 1

17 693 1 4

17 939 1 5

17 713 1 3

17 1359 1 2

17 618 1 3

17 693 1 4

17 617 1 5

17 1113 1 1

17 1723 0 1

17 1541 1 2

17 1000 1 3

17 688 1 4

17 697 1 5

17 922 1 2

17 695 1 3

17 1141 1 2

17 915 1 3

17 602 1 4

17 1358 1 2

17 744 1 5

17 1128 1 1

17 1368 0 1

17 582 1 4

17 1100 1 2

17 855 1 3

17 921 0 1

17 763 1 5

17 1396 0 2

17 658 1 5

17 434 1 4

17 649 1 5

17 469 1 5

17 608 1 4

17 608 1 4

17 1099 1 2

17 1588 1 1

17 958 1 3

17 882 1 1

17 774 1 3

17 654 1 5

17 1404 1 1

17 1537 1 1

17 696 1 5

17 1221 1 3

17 696 1 5

17 704 1 4

17 1095 1 3

17 1323 1 1

17 635 1 4

17 959 1 2

17 1177 1 1

17 617 1 4

17 825 1 2

17 1208 1 2

17 591 0 4

17 661 1 4

17 1405 1 1

17 1140 1 1

17 1144 1 2

17 747 1 2

17 617 1 5

17 780 1 3

17 610 1 5

17 958 1 3

17 720 1 5

17 695 1 4

17 615 1 3

17 1004 1 3

17 1032 1 2

17 1443 0 2

17 611 1 3

17 782 1 2

17 603 1 3

17 1267 1 2

17 1799 1 1

17 913 1 4

17 574 1 3

17 563 1 4

17 1237 1 2

17 661 1 4

17 1146 1 2

17 749 1 5

17 1134 1 1

17 1347 1 1

17 972 1 1

17 740 1 4

17 1057 1 1

17 1284 0 1

17 1360 1 1

17 735 1 3

17 668 1 4

17 827 1 1

17 1081 1 2

17 741 1 4

17 612 1 4

17 1221 1 2

17 696 1 5

17 838 1 3

17 787 1 5

17 572 1 3

17 744 1 3

17 702 1 3

17 789 1 5

17 564 1 5

17 690 1 5

17 566 1 4

17 956 1 2

17 740 1 5

17 698 1 5

17 1268 1 2

17 768 1 5

17 1136 1 2

17 1406 1 1

17 696 1 4

17 1407 1 1

17 567 1 5

17 1267 0 1

17 1202 1 1

17 871 1 3

17 603 1 4

17 961 0 3

17 593 1 4

17 650 1 5

17 787 1 3

17 690 1 3

17 780 1 5

17 1463 1 3

17 1492 1 2

17 609 1 5

17 615 1 5

17 1118 1 1

17 827 1 2

17 745 1 4

17 526 1 4

17 1765 1 1

17 735 1 5

17 826 1 2

17 1102 1 2

17 807 1 3

17 1452 1 2

17 651 1 3

17 648 1 5

17 741 1 4

17 1318 1 1

17 790 1 4

17 751 1 3

17 1268 1 1

17 921 1 2

17 828 1 2

17 792 1 4

17 882 1 2

17 1025 1 2

17 1838 0 1

17 763 1 4

17 913 1 2

17 660 1 3

17 528 1 4

17 612 1 4

17 655 1 4

17 653 1 5

17 1361 1 2

17 1329 1 1

17 740 0 3

17 653 1 5

17 1600 1 1

17 716 1 5

17 824 1 3

17 808 1 3

17 1137 1 1

17 873 1 2

17 1318 1 2

17 915 1 2

17 1008 1 3

17 605 1 5

17 1058 1 5

17 1143 0 1

17 525 1 4

17 697 1 5

17 534 1 4

17 658 1 3

17 507 1 5

17 1229 1 1

17 829 1 3

17 1058 1 1

17 565 1 4

17 1487 1 1

17 524 1 4

17 962 1 3

17 606 1 5

17 1406 1 2

18 3737 1 2

18 3144 1 3

18 2194 1 5

18 3134 1 1

18 2300 1 5

18 2232 1 4

18 2065 1 3

18 1486 1 4

18 2368 1 4

18 2456 1 5

18 2120 1 4

18 1232 1 4

18 1829 1 2

18 1572 1 2

18 1913 1 2

18 2522 1 4

18 2074 1 3

18 1348 1 2

18 1461 1 2

18 4770 1 1

18 2823 1 4

18 2393 1 3

18 1687 1 3

18 8307 0 1

18 1824 1 3

18 2937 1 5

18 1725 1 3

18 2650 1 5

18 2834 1 1

18 1723 1 1

18 3162 1 1

18 1371 1 4

18 1319 1 1

18 4028 1 3

18 1940 1 5

18 2689 1 5

18 1367 1 5

18 3335 1 2

18 2020 1 1

18 1320 1 2

18 3214 1 2

18 1668 1 5

18 3705 0 1

18 6493 1 1

18 2298 1 1

18 2640 1 2

18 1586 1 3

18 1001 1 4

18 1105 1 5

18 2299 1 2

18 1634 1 3

18 1406 1 2

18 1149 1 3

18 1445 1 4

18 1058 1 5

18 1193 1 2

18 1314 1 3

18 5220 1 4

18 1679 1 4

18 1192 1 5

18 1136 1 1

18 1139 1 4

18 1363 1 2

18 1234 1 3

18 1121 1 5

18 3358 0 1

18 1548 1 3

18 1623 1 5

18 2161 1 5

18 1989 1 3

18 1544 1 4

18 1898 1 2

18 1547 1 1

18 1992 1 4

18 1543 1 1

18 2322 1 4

18 1636 1 2

18 1228 1 3

18 1360 1 5

18 4691 0 1

18 1765 1 2

18 1179 1 5

18 1635 1 2

18 1479 1 5

18 1377 1 1

18 1405 1 4

18 1762 0 3

18 1502 1 3

18 2255 1 2

18 1485 1 4

18 1267 1 3

18 959 1 4

18 1586 1 3

18 1315 1 4

18 1772 1 1

18 2158 1 1

18 1229 1 3

18 876 1 5

18 1100 1 5

18 1047 1 4

18 3447 1 4

18 1677 1 2

18 1449 1 2

18 914 1 5

18 738 1 4

18 967 1 5

18 1268 1 2

18 1492 1 1

18 1017 1 1

18 1099 1 2

18 1268 1 5

18 1049 1 5

18 869 1 3

18 1638 0 1

18 1593 1 2

18 1577 1 3

18 1457 1 1

18 1010 1 1

18 790 1 3

18 966 1 4

18 1010 1 3

18 1013 1 4

18 955 1 4

18 1450 1 2

18 1893 0 1

18 1107 1 3

18 1098 1 5

18 1976 1 3

18 1274 1 4

18 2074 1 2

18 1320 1 2

18 1228 1 1

18 1324 1 3

18 2153 1 1

18 1140 1 5

18 1395 1 1

18 1232 1 3

18 1091 1 1

18 1642 1 2

18 874 1 5

18 842 1 4

18 1168 1 1

18 1322 1 2

18 1273 1 4

18 1054 1 5

18 1893 1 4

18 1276 1 3

18 1544 1 1

18 1061 1 5

18 1057 1 3

18 1807 1 1

18 1188 1 5

18 1145 1 4

18 1269 1 2

18 1091 1 5

18 1003 1 2

18 917 1 3

18 1637 1 4

18 1361 1 5

18 1236 1 2

18 1220 1 1

18 870 1 5

18 1719 1 1

18 873 1 3

18 1009 1 5

18 885 1 4

18 1383 1 5

18 1666 0 1

18 1024 1 3

18 878 1 5

18 1626 1 4

18 958 1 4

18 1671 1 3

18 1143 1 5

18 778 1 3

18 1235 1 3

18 1077 1 2

18 872 1 2

18 1324 1 4

18 1095 1 2

18 788 1 4

18 1232 1 2

18 1845 1 5

18 1015 1 5

18 1327 1 3

18 923 1 5

18 876 1 4

18 2477 0 1

18 927 1 1

18 1402 1 2

18 2514 0 1

18 1905 1 2

18 1419 1 1

18 1052 1 2

18 1490 1 2

18 964 1 3

18 1062 1 4

18 1133 1 4

18 1271 1 1

18 1095 1 3

18 962 1 3

18 1058 1 4

18 967 1 2

18 1031 1 5

18 1535 1 1

18 928 1 4

18 1401 1 1

18 1354 1 3

18 1189 1 3

18 1142 1 2

18 2289 1 1

18 965 1 2

18 785 1 5

18 961 1 5

18 867 1 4

18 2639 1 2

18 1386 1 1

18 1520 1 4

18 836 1 5

18 1497 1 2

18 1372 1 2

18 1106 1 5

18 970 1 4

18 1496 1 5

18 2071 0 1

18 1296 1 1

18 2166 1 4

18 1189 1 1

18 1053 1 4

18 1139 1 3

18 1497 1 3

18 1852 1 2

18 1361 1 5

18 1235 1 3

18 1138 1 3

18 1060 1 3

18 2566 1 4

18 1359 1 2

18 2739 1 1

18 967 1 5

18 1412 1 3

18 1977 1 1

18 1146 1 1

18 1015 1 1

18 1452 1 4

18 1005 1 5

18 837 1 5

18 1050 1 5

18 1892 1 3

18 1277 1 2

18 1189 1 4

18 1629 0 1

18 878 1 4

18 1458 1 3

18 1141 1 3

18 1624 1 2

18 1143 1 4

18 967 1 3

18 1143 1 2

18 1324 1 5

18 1319 1 2

18 2422 1 5

18 1358 1 3

18 1147 1 4

18 1209 1 2

18 746 1 4

18 750 1 5

18 927 1 4

18 1186 1 2

18 1316 1 3

18 1537 1 5

18 1115 1 2

18 1907 1 3

18 2167 1 1

18 1757 1 1

18 869 1 5

18 1405 1 1

18 1052 1 2

18 1225 1 1

18 968 1 4

18 834 1 4

18 914 1 4

18 767 1 4

18 826 1 4

18 835 1 4

18 783 1 5

18 918 1 4

18 1664 1 5

18 1150 1 3

18 1849 1 2

18 1003 1 2

18 1635 1 1

18 1935 1 3

18 961 1 1

18 1636 1 2

18 1669 1 3

18 695 1 5

18 1059 1 5

18 1138 1 2

18 1228 1 1

18 1222 1 2

18 1980 1 3

18 883 1 5

18 1628 1 2

18 1500 1 2

18 1623 1 3

18 1504 1 3

18 1184 1 5

18 1055 1 3

18 1269 1 2

18 873 1 4

18 1852 0 1

18 1402 1 1

18 1979 1 1

18 2212 1 1

18 1178 1 5

18 1192 1 4

18 1055 1 1

18 1147 0 5

18 2450 1 3

18 1094 1 2

18 751 1 4

18 1801 1 2

18 4768 0 3

18 884 1 4

18 6316 1 1

18 785 1 5

18 1846 1 5

18 1405 1 5

18 2154 1 2

18 2065 1 5

18 868 1 5

18 2333 1 1

18 1047 1 3

18 1548 1 2

18 1283 1 2

18 803 1 4

18 1130 1 3

18 1238 1 3

18 964 1 3

18 1188 1 1

18 1225 1 3

18 835 1 4

18 739 1 4

18 1238 1 5

18 653 1 4

18 916 1 2

18 1187 1 1

18 1371 1 1

18 960 1 5

18 2696 1 1

18 1049 1 3

18 2872 1 1

18 910 1 5

18 1013 1 4

18 4066 1 1

18 4898 1 2

18 1449 1 3

18 1091 1 4

18 1449 1 2

18 711 1 4

18 2744 1 5

18 1937 1 1

18 1671 1 3

18 1672 1 1

18 1005 1 4

18 964 1 5

18 1150 1 1

18 958 1 4

18 1010 1 4

18 1375 1 3

18 1179 1 3

18 926 1 2

18 1767 1 1

18 2288 1 4

18 919 1 3

18 1671 1 5

18 1184 1 5

18 1444 1 2

18 1315 1 5

18 1311 1 1

18 1368 1 5

18 1104 1 3

18 918 1 1

18 2067 1 5

18 782 1 4

18 824 1 2

18 925 1 4

18 1453 1 1

18 1272 1 2

18 834 1 4

18 735 1 5

18 1262 1 1

18 1193 1 2

18 1233 1 2

18 1587 1 3

18 1095 0 2

18 1241 1 2

18 737 1 3

18 1582 1 3

19 696 1 5

19 833 1 3

19 1375 1 3

19 1080 1 2

19 2405 1 5

19 1937 1 2

19 919 1 4

19 1770 1 3

19 691 1 4

19 695 1 4

19 1403 1 1

19 711 1 5

19 1305 0 1

19 1086 1 1

19 680 1 4

19 616 1 5

19 1011 1 5

19 1051 1 4

19 722 1 5

19 676 1 4

19 1154 1 3

19 768 1 5

19 1476 1 1

19 1233 0 1

19 1069 1 2

19 672 1 4

19 1086 0 2

19 923 1 2

19 1231 1 1

19 1809 1 1

19 1285 1 3

19 871 1 4

19 948 1 1

19 844 1 3

19 1013 1 2

19 1270 1 2

19 1265 1 2

19 765 1 5

19 1207 1 3

19 1037 1 3

19 931 1 4

19 735 1 4

19 829 1 1

19 661 1 5

19 1110 1 3

19 1094 1 3

19 1092 1 1

19 759 1 3

19 784 1 2

19 826 1 5

19 1190 0 1

19 722 1 5

19 1444 1 3

19 839 1 4

19 789 1 5

19 912 1 3

19 1180 0 1

19 759 0 2

19 1441 1 3

19 701 1 4

19 1401 1 3

19 886 1 4

19 996 1 2

19 861 1 2

19 704 1 4

19 695 1 5

19 1059 1 2

19 1108 0 3

19 1163 1 1

19 834 1 4

19 1005 1 5

19 1409 1 1

19 736 1 4

19 697 1 5

19 928 1 2

19 1058 1 1

19 749 1 5

19 931 1 2

19 1147 1 2

19 1063 1 1

19 706 1 4

19 1191 1 3

19 1591 1 1

19 746 1 4

19 1149 0 3

19 700 1 4

19 648 1 3

19 876 1 5

19 925 1 4

19 1498 1 2

19 869 1 4

19 1600 1 1

19 986 1 5

19 829 1 5

19 883 1 5

19 914 1 1

19 663 1 4

19 1055 1 2

19 1541 1 1

19 743 1 5

19 1156 0 2

19 1119 1 2

19 878 1 4

19 789 1 5

19 916 0 1

19 1155 1 1

19 1363 1 2

19 1016 1 5

19 1011 1 1

19 793 1 3

19 880 1 2

19 1282 1 2

19 870 1 3

19 820 1 3

19 1142 0 1

19 1474 1 2

19 1626 1 3

19 746 1 4

19 1728 1 3

19 740 1 5

19 917 1 1

19 1222 1 1

19 791 1 5

19 1139 1 1

19 654 1 5

19 875 1 5

19 744 1 4

19 1015 1 2

19 1021 1 1

19 1042 1 4

19 1410 0 1

19 1756 0 2

19 1721 1 1

19 1893 1 5

19 748 1 4

19 702 1 5

19 2378 1 4

19 1010 0 2

19 710 1 3

19 826 1 1

19 1155 1 2

19 1226 1 2

19 862 1 3

19 741 1 4

19 1093 1 3

19 702 1 2

19 783 1 5

19 1101 1 4

19 1379 1 3

19 748 1 4

19 653 1 5

19 1138 1 5

19 1100 1 3

19 1274 0 2

19 739 1 2

19 2144 1 3

19 1458 1 3

19 746 1 4

19 772 1 3

19 1075 0 1

19 829 1 4

19 883 0 1

19 732 0 3

19 880 1 1

19 884 1 1

19 1093 1 3

19 969 1 5

19 1228 1 2

19 743 1 5

19 791 1 5

19 612 1 4

19 1186 1 3

19 744 1 4

19 830 1 3

19 1017 1 1

19 810 1 4

19 831 0 3

19 1160 1 1

19 708 1 2

19 701 1 5

19 922 1 5

19 921 1 1

19 1098 1 3

19 661 0 2

19 1016 1 1

19 915 1 2

19 1052 1 3

19 609 1 5

19 699 1 4

19 784 1 4

19 692 1 4

19 835 0 2

19 764 1 2

19 1015 1 2

19 1054 1 2

19 875 1 3

19 1233 0 1

19 627 1 4

19 526 1 5

19 656 1 5

19 740 0 1

19 589 1 5

19 969 1 2

19 1139 1 5

19 1094 1 3

19 661 1 4

19 880 1 3

19 1074 1 2

19 997 1 2

19 790 1 2

19 921 0 3

19 686 1 5

19 830 1 5

19 918 1 1

19 1322 0 1

19 694 1 2

19 882 1 4

19 656 1 4

19 789 1 4

19 794 1 4

19 694 1 3

19 1104 1 3

19 663 1 4

19 876 1 5

19 703 1 5

19 768 1 4

19 780 1 3

19 700 1 4

19 1278 1 1

19 954 1 3

19 971 1 5

19 1004 1 1

19 1008 1 1

19 1365 1 2

19 1116 1 2

19 757 1 1

19 791 1 5

19 1230 0 3

19 1252 1 2

19 1271 0 1

19 885 1 2

19 827 0 2

19 1218 1 2

19 832 1 2

19 974 1 3

19 1365 1 1

19 746 1 5

19 584 1 4

19 915 1 4

19 1445 0 1

19 919 1 2

19 768 1 4

19 785 1 5

19 915 1 4

19 964 1 4

19 1273 1 1

19 922 1 4

19 1021 1 5

19 1274 1 2

19 1099 0 1

19 1291 1 2

19 1097 1 3

19 662 1 5

19 705 1 5

19 1230 0 1

19 979 0 3

19 977 1 3

19 1102 0 1

19 808 1 5

19 611 1 5

19 527 1 4

19 825 1 3

19 913 1 3

19 794 1 1

19 1548 1 1

19 706 1 4

19 923 1 3

19 656 1 3

19 805 1 2

19 957 1 5

19 881 1 3

19 1004 1 3

19 877 1 1

19 744 1 5

19 1103 0 1

19 811 1 1

19 1188 1 2

19 974 1 4

19 658 1 4

19 524 1 5

19 854 1 4

19 562 1 4

19 617 1 5

19 1052 1 2

19 827 1 2

19 837 0 1

19 1236 1 1

19 967 1 5

19 959 1 3

19 907 1 1

19 745 1 4

19 926 1 2

19 1057 1 3

19 1283 0 2

19 2399 1 2

19 604 0 3

19 1272 1 2

19 879 1 1

19 971 1 5

19 903 1 5

19 1272 1 3

19 920 1 1

19 889 1 5

19 749 1 5

19 1090 1 3

19 914 0 2

19 1011 0 4

19 737 1 4

19 742 1 4

19 591 1 3

19 1142 1 2

19 834 1 5

19 1181 1 1

19 1239 1 1

19 788 1 3

19 853 1 4

19 921 1 2

19 1143 1 3

19 1014 1 3

19 1459 1 2

19 1017 1 3

19 965 0 2

19 848 1 4

19 1283 1 2

19 1252 1 1

19 696 1 4

19 705 1 5

19 1225 1 1

19 782 1 3

19 966 1 2

19 693 1 5

19 615 1 4

19 827 1 3

19 1102 1 1

19 873 1 3

19 789 1 5

19 655 1 5

19 742 1 4

19 752 1 2

19 601 1 5

19 650 1 4

19 1315 0 1

19 856 1 4

19 1058 1 5

19 836 1 3

19 608 1 4

19 839 1 2

19 1048 0 1

19 833 1 1

19 786 0 5

19 660 1 5

19 746 1 2

19 782 1 3

19 841 1 3

19 965 1 1

19 613 1 4

19 530 1 5

19 717 1 5

19 1098 1 1

19 1094 1 1

19 1013 1 1

19 884 1 2

19 657 1 4

19 1015 1 2

19 699 1 4

19 691 1 3

19 926 1 2

19 1187 1 2

19 697 1 5

19 1067 1 5

19 777 1 2

19 1362 1 3

19 829 1 1

19 795 1 4

19 1149 1 1

19 914 1 3

19 618 1 4

19 1138 1 1

19 645 1 3

19 573 0 5

19 704 1 5

19 974 1 2

19 1136 1 1

19 601 1 4

19 881 1 4

19 665 1 5

19 1358 1 3

19 783 1 2

19 821 1 3

19 836 1 4

20 3073 1 2

20 2670 1 3

20 2733 1 2

20 2509 1 3

20 1599 1 3

20 1230 1 4

20 2767 1 1

20 1641 1 3

20 1348 1 5

20 1646 1 3

20 2105 1 5

20 861 1 4

20 1593 1 2

20 2383 1 1

20 926 1 5

20 1067 1 3

20 2111 0 5

20 3499 1 4

20 1788 1 1

20 2563 1 2

20 1054 1 5

20 1187 1 5

20 2247 1 1

20 1269 1 4

20 1765 1 1

20 1083 1 2

20 1195 1 4

20 1237 1 4

20 740 1 5

20 2429 1 1

20 1141 1 4

20 1630 1 3

20 1000 1 2

20 1058 1 4

20 1230 1 5

20 1697 1 3

20 1576 1 1

20 1837 1 2

20 1389 1 1

20 1577 1 2

20 927 1 5

20 1278 1 2

20 1856 0 1

20 1058 1 1

20 1537 1 1

20 1326 1 5

20 782 1 5

20 1368 1 1

20 1579 1 2

20 1106 1 4

20 793 1 4

20 1103 1 5

20 1269 1 2

20 1143 1 4

20 1519 1 3

20 1756 1 5

20 968 1 3

20 1233 1 2

20 1843 1 1

20 971 1 3

20 1147 1 5

20 1503 1 3

20 1192 1 1

20 1039 1 4

20 1345 1 2

20 1402 1 2

20 1228 1 4

20 1599 1 3

20 1005 1 3

20 1077 1 2

20 1277 1 1

20 1323 1 2

20 1319 0 3

20 1736 1 5

20 1403 1 3

20 1679 1 1

20 1101 1 4

20 1268 1 5

20 1274 1 4

20 781 1 4

20 1190 1 1

20 829 1 3

20 1280 1 1

20 1017 1 3

20 1224 1 1

20 1358 1 4

20 1719 1 1

20 1449 1 3

20 1399 1 1

20 1135 1 3

20 927 1 3

20 914 1 2

20 1000 1 2

20 962 1 5

20 1010 1 5

20 832 1 5

20 1179 1 3

20 1015 1 3

20 868 1 2

20 968 1 4

20 694 1 5

20 2033 1 1

20 914 1 4

20 1016 1 4

20 1366 1 2

20 783 1 5

20 1444 1 2

20 972 1 5

20 1385 1 4

20 1270 1 2

20 1580 1 2

20 1149 1 3

20 961 1 4

20 1187 1 5

20 962 1 5

20 1101 1 4

20 1519 1 1

20 981 1 2

20 1140 1 1

20 921 1 4

20 1541 1 5

20 960 1 3

20 966 1 5

20 718 1 3

20 1140 0 2

20 1164 1 3

20 1409 1 2

20 1056 1 1

20 965 1 5

20 1482 1 3

20 1268 1 1

20 1344 1 4

20 1458 1 4

20 1453 1 2

20 1053 1 3

20 1142 1 5

20 957 1 5

20 1330 1 3

20 975 1 2

20 1715 0 1

20 889 1 1

20 832 1 2

20 1015 1 4

20 603 1 5

20 1139 1 1

20 1149 1 5

20 2020 1 1

20 1047 1 5

20 972 1 4

20 1416 1 2

20 1586 1 3

20 749 1 4

20 1054 1 2

20 830 1 3

20 794 1 4

20 703 1 4

20 1785 1 1

20 1135 1 2

20 1005 1 1

20 1017 1 4

20 785 1 5

20 781 1 5

20 1015 1 2

20 1001 1 1

20 1282 1 1

20 1720 1 1

20 1582 1 1

20 780 1 4

20 968 1 3

20 915 1 4

20 958 1 5

20 1193 1 5

20 1499 1 3

20 1311 1 1

20 866 1 4

20 1113 1 3

20 1262 1 3

20 1016 1 5

20 1148 1 1

20 1223 1 3

20 835 1 4

20 1092 1 5

20 1764 1 4

20 1282 1 1

20 1090 1 2

20 1504 1 2

20 1131 1 4

20 1049 1 2

20 1405 1 4

20 1366 0 1

20 784 1 2

20 862 1 3

20 923 1 5

20 920 1 3

20 1095 1 2

20 925 1 2

20 1011 1 3

20 704 1 4

20 741 1 5

20 1059 1 2

20 961 1 4

20 793 1 4

20 1178 1 3

20 1233 1 1

20 837 1 3

20 1056 1 4

20 1230 1 2

20 1370 1 4

20 810 1 5

20 1121 1 1

20 963 1 5

20 927 1 4

20 1056 1 2

20 703 1 3

20 838 1 1

20 609 1 4

20 1190 1 1

20 1006 1 5

20 788 1 5

20 1155 1 3

20 1533 1 2

20 1047 1 2

20 874 1 4

20 773 1 5

20 1104 1 5

20 1317 1 2

20 751 1 3

20 1225 1 2

20 924 1 1

20 965 1 3

20 957 1 2

20 921 1 4

20 924 1 3

20 1143 1 5

20 1144 1 1

20 1107 1 2

20 1317 1 1

20 1092 1 1

20 924 1 3

20 1496 1 5

20 1226 1 1

20 838 1 3

20 1185 1 1

20 868 1 4

20 794 1 5

20 561 1 4

20 572 1 4

20 610 1 5

20 1067 1 1

20 1233 1 2

20 649 1 4

20 956 1 3

20 980 1 2

20 1237 0 2

20 1161 0 2

20 884 0 5

20 784 1 5

20 826 0 3

20 953 1 3

20 783 1 5

20 1047 1 1

20 1134 1 2

20 1098 1 1

20 823 1 4

20 1230 1 1

20 928 1 5

20 796 1 1

20 824 1 5

20 618 1 3

20 1095 1 3

20 1063 1 2

20 923 1 2

20 1015 1 3

20 735 1 5

20 835 1 4

20 1004 1 4

20 870 1 1

20 1050 1 2

20 1181 1 3

20 910 1 4

20 1267 1 2

20 826 1 3

20 702 1 3

20 1093 1 4

20 1109 1 1

20 783 1 5

20 1135 1 4

20 961 1 2

20 1404 1 2

20 838 1 3

20 793 1 5

20 824 1 5

20 1003 1 5

20 1139 1 5

20 1452 1 1

20 835 1 4

20 1455 0 1

20 828 1 4

20 870 1 4

20 1011 1 4

20 973 1 2

20 1673 1 1

20 1056 1 2

20 1145 1 2

20 1012 1 4

20 704 1 5

20 874 1 3

20 1022 1 2

20 1350 1 1

20 1316 1 3

20 745 1 3

20 925 1 1

20 703 1 2

20 855 1 4

20 918 1 5

20 1990 1 1

20 833 1 5

20 1135 1 3

20 914 1 3

20 1008 1 1

20 837 1 3

20 783 1 3

20 835 1 5

20 1005 1 5

20 1008 1 3

20 703 1 4

20 652 1 4

20 912 1 4

20 1012 1 2

20 1135 1 2

20 1314 1 1

20 840 1 1

20 974 1 2

20 1173 1 1

20 965 1 4

20 796 1 3

20 660 1 2

20 845 1 5

20 1191 1 2

20 1543 1 1

20 790 1 4

20 1360 1 1

20 1060 1 3

20 968 1 1

20 781 1 4

20 933 1 5

20 749 1 2

20 740 1 5

20 1002 1 1

20 871 1 5

20 947 1 2

20 1459 1 3

20 968 1 1

20 1048 0 2

20 1244 1 5

20 838 1 4

20 786 1 4

20 1359 1 3

20 945 1 3

20 726 1 5

20 1500 1 1

20 1091 1 2

20 700 1 4

20 1225 1 1

20 726 1 5

20 1766 1 4

20 781 1 5

20 914 1 3

20 805 1 3

20 698 1 5

20 928 1 3

20 1589 0 5

20 650 1 4

20 1143 1 1

20 1415 1 1

20 1047 1 2

20 945 1 3

20 750 1 2

20 832 1 5

20 1223 1 2

20 610 1 4

20 792 1 5

20 914 1 4

20 824 1 4

20 1585 1 1

20 917 1 1

20 777 1 2

20 1462 1 1

20 1047 1 2

20 1237 1 2

20 916 1 4

20 1046 1 2

20 926 1 4

20 1058 1 5

20 744 1 3

20 968 1 3

20 1090 1 3

20 832 1 3

20 906 1 5

20 1585 1 1

21 1128 1 5

21 1235 1 4

21 884 1 4

21 1093 1 4

21 1234 1 2

21 916 1 4

21 1580 1 3

21 1140 1 2

21 1047 1 5

21 1533 1 1

21 781 1 4

21 1046 1 3

21 1670 1 5

21 1094 1 2

21 1135 1 2

21 1181 1 3

21 1809 1 1

21 1542 1 2

21 967 0 3

21 1317 1 2

21 1322 1 3

21 1104 1 5

21 2336 1 1

21 960 1 4

21 1315 1 2

21 1181 1 5

21 1192 1 1

21 1322 1 3

21 1008 1 5

21 1144 1 3

21 1013 1 3

21 2610 0 1

21 1099 1 4

21 1618 1 1

21 965 1 5

21 837 1 4

21 2033 1 1

21 1090 1 2

21 1409 1 5

21 1366 1 1

21 739 1 3

21 918 1 4

21 1136 0 3

21 1780 1 1

21 1193 1 1

21 924 1 5

21 2205 1 2

21 1829 1 2

21 1326 1 2

21 1145 1 5

21 1542 1 1

21 1120 1 2

21 2035 1 1

21 917 1 5

21 1275 1 4

21 1189 1 1

21 1192 1 3

21 1049 1 4

21 1459 1 3

21 1366 1 2

21 1136 1 1

21 960 1 5

21 1009 1 4

21 1188 1 3

21 1189 1 4

21 1011 1 4

21 880 1 5

21 1104 1 2

21 871 1 3

21 1184 1 2

21 1015 1 5

21 2198 1 1

21 824 1 5

21 1320 1 2

21 1938 1 1

21 1671 1 4

21 919 1 5

21 1226 1 3

21 1233 1 3

21 1135 1 4

21 1011 1 3

21 919 1 3

21 1090 1 1

21 1276 1 3

21 2959 1 1

21 1193 1 2

21 790 1 4

21 1011 1 5

21 1712 1 1

21 1015 1 5

21 1018 0 5

21 877 1 5

21 1103 1 2

21 1227 1 4

21 1323 1 3

21 1454 1 1

21 3530 1 1

21 2241 1 2

21 1233 1 1

21 651 1 4

21 735 1 4

21 993 1 3

21 1124 1 5

21 1851 1 1

21 916 1 4

21 788 1 5

21 1051 1 5

21 1058 1 4

21 1099 1 4

21 1540 1 3

21 1319 1 2

21 944 1 3

21 1013 1 3

21 925 1 4

21 1409 1 2

21 1497 1 2

21 1046 1 2

21 1103 1 2

21 979 1 5

21 1355 1 1

21 1228 1 2

21 784 1 5

21 914 1 2

21 928 1 2

21 925 1 5

21 1535 1 1

21 696 1 5

21 923 1 3

21 1764 1 2

21 1044 1 3

21 4988 1 1

21 922 1 2

21 1856 1 1

21 1319 1 1

21 827 1 3

21 925 1 4

21 789 1 5

21 830 1 4

21 1205 1 2

21 837 1 3

21 1661 1 1

21 748 1 5

21 1745 1 1

21 1223 1 2

21 1189 1 1

21 1362 1 3

21 1280 1 1

21 840 1 4

21 1134 1 4

21 737 1 5

21 833 1 5

21 1761 1 2

21 877 1 3

21 880 1 3

21 876 1 4

21 791 1 5

21 656 1 4

21 745 1 4

21 655 1 4

21 1402 1 3

21 1102 1 3

21 915 1 2

21 1015 1 3

21 1216 1 2

21 781 1 4

21 926 1 5

21 1278 1 1

21 2249 1 1

21 1805 1 1

21 827 1 4

21 949 1 5

21 880 1 1

21 750 0 4

21 1134 1 2

21 1137 1 2

21 2432 1 1

21 1403 1 2

21 886 1 4

21 1491 1 5

21 1496 1 2

21 921 1 5

21 1591 1 3

21 871 1 4

21 1051 1 3

21 962 1 1

21 995 1 3

21 1009 1 5

21 1136 1 3

21 1259 1 1

21 968 1 2

21 1358 1 3

21 1039 1 2

21 839 1 4

21 1363 1 5

21 825 1 4

21 966 1 5

21 825 1 3

21 745 1 4

21 1481 0 1

21 1067 1 5

21 872 1 2

21 837 1 4

21 700 1 4

21 830 1 5

21 1281 1 1

21 793 1 5

21 1447 1 3

21 1314 1 2

21 921 1 4

21 923 1 3

21 1186 1 2

21 873 1 3

21 1496 1 1

21 882 1 5

21 1045 1 3

21 1066 1 3

21 741 1 4

21 1847 1 1

21 1000 1 5

21 1003 1 4

21 1011 0 2

21 1081 1 1

21 662 1 4

21 1325 1 2

21 1542 1 3

21 927 1 5

21 1495 0 1

21 881 1 5

21 1710 1 2

21 1502 1 3

21 1138 1 1

21 832 1 5

21 1136 1 5

21 1226 1 2

21 749 1 4

21 1488 1 1

21 1011 1 3

21 1045 1 1

21 882 1 4

21 940 1 2

21 1137 1 2

21 1103 1 3

21 877 1 1

21 791 1 4

21 827 1 5

21 747 1 4

21 839 1 4

21 1185 1 2

21 915 1 4

21 985 1 4

21 1045 1 2

21 1147 1 2

21 695 1 3

21 1360 1 3

21 1109 0 2

21 1106 0 1

21 1367 0 1

21 926 1 4

21 1102 1 2

21 1983 1 1

21 1187 1 2

21 793 1 5

21 790 1 4

21 1102 1 3

21 1580 1 1

21 743 1 4

21 831 1 5

21 1105 1 2

21 1008 1 5

21 1011 1 5

21 1094 1 3

21 1716 0 1

21 1147 1 3

21 2252 1 1

21 785 1 5

21 3056 1 1

21 1317 1 3

21 693 1 5

21 702 1 3

21 1055 1 5

21 912 1 3

21 824 1 4

21 1401 1 1

21 1137 1 1

21 1007 1 2

21 786 1 5

21 1006 1 2

21 688 1 4

21 834 1 4

21 879 1 3

21 1404 1 1

21 920 1 3

21 1138 1 1

21 1497 1 1

21 751 0 5

21 1053 1 3

21 1008 1 5

21 875 1 2

21 780 1 5

21 1237 1 3

21 1416 1 1

21 1413 1 2

21 1015 1 2

21 701 1 5

21 959 1 3

21 879 1 4

21 632 1 4

21 1370 1 1

21 1050 1 2

21 701 1 4

21 1344 1 2

21 1071 1 1

21 785 1 3

21 1113 1 5

21 747 1 5

21 1012 1 3

21 649 1 4

21 1311 1 2

21 1014 1 5

21 819 1 4

21 1186 1 1

21 1045 1 1

21 787 1 5

21 830 1 5

21 1710 1 1

21 1418 1 1

21 741 1 4

21 1009 1 3

21 748 1 4

21 655 1 4

21 698 1 4

21 658 0 4

21 1665 1 1

21 871 1 5

21 791 1 5

21 1138 1 2

21 1268 1 2

21 1093 1 2

21 823 1 4

21 1097 1 2

21 972 1 3

21 838 1 5

21 1237 1 2

21 822 1 1

21 786 1 4

21 828 1 5

21 793 1 4

21 1100 1 5

21 877 1 3

21 2023 1 3

21 1009 1 1

21 1146 1 2

21 943 1 3

21 1011 1 3

21 1234 1 2

21 1675 1 3

21 966 1 3

21 913 1 5

21 1379 1 1

21 1270 1 2

21 1109 1 5

21 1100 1 1

21 883 1 4

21 1051 1 2

21 1810 1 1

21 747 1 4

21 1104 1 2

21 918 1 3

21 1281 1 2

21 1237 1 2

21 1070 1 2

21 987 1 5

21 961 1 3

21 1452 1 3

21 1093 1 1

21 1010 1 3

21 791 1 4

21 921 1 5

21 1269 1 3

21 1403 1 1

21 1095 1 1

21 970 1 4

21 697 1 3

21 703 1 4

21 1361 1 1

21 960 1 2

21 1057 1 5

21 743 1 4

21 835 1 5

21 1045 1 1

21 1280 1 3

21 795 1 4

21 1266 1 5

21 792 1 5

21 1100 1 2

21 832 1 4

21 1136 1 3

21 1138 1 5

21 1226 1 1

21 1311 1 2

Key:

Column 1: Participant number

Column 2: Reaction time (milliseconds)

Column 3: Error (0) or correct response (1)

Column 4: Ratio (1) 1:1.468 (2) 1:1.518 (3) 1:1.568 (4) 1:1.618 (5) 1:1.668
