# Supplementary material for: The Golden Section as Optical Limitation
Source: PLoS One. 2015 Jul 8;10(7):e0131045. doi: 10.1371/journal.pone.0131045 (PMC4495923; doi:10.1371/journal.pone.0131045)
Supplement: S1 Table — (DOCX) [file pone.0131045.s004.docx]

**Table S1. Mean scale values for each ratio derived by the law of comparative judgment**

| **c1** | **c2** | **c3** | **c4** | **c5** | **c6** | **c7** | **c8** | **c9** | **c10** | **c11** | **c12** | **c13** | **c14** | **c15** | **S** |
| --- | --- | --- | --- | --- | --- | --- | --- | --- | --- | --- | --- | --- | --- | --- | --- |
| 0 | 0.03 | 0.18 | 0.18 | 0.13 | 0 | 0.02 | 0 | 0.13 | 0.1 | 0 | 0.02 | 0.13 | 0.46 | 0.28 | 2 |
| 0.07 | 0.15 | 0.05 | 0 | 1.12 | 0 | 0.04 | 0.5 | 0.63 | 1.6 | 0 | 0.32 | 1 | 1.24 | 1.92 | 3 |
| 0 | 0.31 | 0.44 | 0.49 | 0.31 | 0 | 0.21 | 0.18 | 0.33 | 0.05 | 0.08 | 0.24 | 0.24 | 0 | 0.24 | 4 |
| 0.94 | 0.43 | 0.37 | 1.34 | 0 | 0.87 | 0.76 | 0.33 | 0.26 | 0 | 0.2 | 0.08 | 0.15 | 0 | 0.08 | 5 |
| 0 | 0.36 | 0.49 | 0 | 0.67 | 0.57 | 0.22 | 0.4 | 0.04 | 0 | 0.78 | 0.84 | 0.65 | 0.46 | 0 | 6 |
| 0.1 | 0.09 | 0.02 | 0.29 | 0 | 0.13 | 0.21 | 0 | 0 | 0.05 | 0 | 0.18 | 0.23 | 0.1 | 0.25 | 7 |
| 0.32 | 0 | 0.16 | 0.14 | 0.19 | 0.07 | 0 | 0.12 | 0.28 | 0.25 | 0.52 | 0.33 | 0.35 | 0 | 0.29 | 8 |
| 0 | 0.16 | 0.03 | 0.34 | 0 | 0 | 0.34 | 0.24 | 0.41 | 0.31 | 0.1 | 0.14 | 0.29 | 0.66 | 0 | 9 |
| 0.69 | 0.9 | 0.66 | 0.67 | 0 | 1.16 | 1.06 | 0.81 | 0.34 | 0 | 1.27 | 0.97 | 0.89 | 0.62 | 0 | 10 |
| 0.05 | 0 | 0 | 0.08 | 0.13 | 0.36 | 0.32 | 0.47 | 0.42 | 0 | 0.08 | 0.05 | 0.13 | 0.39 | 0 | 11 |
| 0.28 | 0 | 0.23 | 0.33 | 0.18 | 0 | 0.13 | 0.13 | 0.07 | 0.05 | 0.23 | 0.15 | 0 | 0.23 | 0.28 | 12 |
| 0.35 | 0 | 0.29 | 0.17 | 0 | 0 | 0.29 | 0.29 | 0.12 | 0.4 | 0.26 | 0.13 | 0 | 0.08 | 0.05 | 13 |

Note scale values were calculated and returned as output by the experimental program.

Key Row 1:

C1 4-paired sections 1:1.468 ratio

C2 4-paired sections 1:1.518 ratio

C3 4 paired sections 1:1.568 ratio

C4 4-paired sections 1:1.618 ratio

C5 4-paired sections 1:1.668 ratio

C6 8-paired sections 1:1.468 ratio

C7 8-paired sections 1:1.518 ratio

C8 8 paired sections 1:1.568 ratio

C9 8-paired sections 1:1.618 ratio

C10 8-paired sections 1:1.668 ratio

C11 16-paired sections 1:1.468 ratio

C12 16-paired sections 1:1.518 ratio

C13 16 paired sections 1:1.568 ratio

C14 16-paired sections 1:1.618 ratio

C15 16-paired sections 1:1.668 ratio

S = Participant number
