# Supplementary material for: The Golden Section as Optical Limitation
Source: PLoS One. 2015 Jul 8;10(7):e0131045. doi: 10.1371/journal.pone.0131045 (PMC4495923; doi:10.1371/journal.pone.0131045)
Supplement: S2 Table — (DOCX) [file pone.0131045.s005.docx]

**Table S2. Arcsin (square-root) transformed error proportions in Experiment 1**

| **c1** | **c2** | **c3** | **c4** | **c5** | **c6** | **c7** | **c8** | **c9** | **c10** | **c11** | **c12** | **c13** | **c14** | **c15** | **S** |
| --- | --- | --- | --- | --- | --- | --- | --- | --- | --- | --- | --- | --- | --- | --- | --- |
| .51 | .58 | .36 | .46 | .36 | .57 | .84 | .49 | .70 | .43 | .49 | .40 | .43 | .43 | .52 | 2 |
| .16 | .25 | .11 | .19 | .11 | .40 | .62 | .25 | .28 | .11 | .16 | .03 | .03 | .11 | .11 | 3 |
| .45 | .30 | .11 | .03 | .16 | .36 | .57 | .23 | .32 | .23 | .11 | .11 | .28 | .32 | .11 | 4 |
| .43 | .25 | .23 | .30 | .32 | .63 | .63 | .30 | .34 | .16 | .23 | .25 | .23 | .16 | .19 | 5 |
| .28 | .25 | .16 | .28 | .19 | .32 | .71 | .34 | .55 | .11 | .11 | .28 | .23 | .19 | .11 | 6 |
| .51 | .46 | .49 | .58 | .52 | .57 | .75 | .40 | .70 | .25 | .48 | .45 | .36 | .34 | .48 | 7 |
| .58 | .41 | .38 | .45 | .38 | .68 | .85 | .54 | .57 | .48 | .41 | .45 | .38 | .41 | .48 | 8 |
| .52 | .16 | .19 | .16 | .11 | .61 | .77 | .11 | .28 | .11 | .16 | .16 | .03 | .16 | .28 | 9 |
| .25 | .28 | .19 | .23 | .19 | .59 | .74 | .19 | .46 | .16 | .28 | .19 | .19 | .16 | .23 | 10 |
| .51 | .23 | .36 | .34 | .25 | .70 | .84 | .45 | .41 | .30 | .40 | .30 | .45 | .30 | .34 | 11 |
| .38 | .25 | .25 | .28 | .28 | .59 | .82 | .40 | .49 | .19 | .41 | .38 | .30 | .28 | .28 | 12 |
| .68 | .49 | .48 | .41 | .36 | .76 | .86 | .49 | .68 | .43 | .52 | .43 | .49 | .32 | .38 | 13 |

Key Row 1:

C1 4-paired sections 1:1.468 ratio

C2 4-paired sections 1:1.518 ratio

C3 4 paired sections 1:1.568 ratio

C4 4-paired sections 1:1.618 ratio

C5 4-paired sections 1:1.668 ratio

C6 8-paired sections 1:1.468 ratio

C7 8-paired sections 1:1.518 ratio

C8 8 paired sections 1:1.568 ratio

C9 8-paired sections 1:1.618 ratio

C10 8-paired sections 1:1.668 ratio

C11 16-paired sections 1:1.468 ratio

C12 16-paired sections 1:1.518 ratio

C13 16 paired sections 1:1.568 ratio

C14 16-paired sections 1:1.618 ratio

C15 16-paired sections 1:1.668 ratio

S = Participant number
