# Supplementary material for: The Golden Section as Optical Limitation
Source: PLoS One. 2015 Jul 8;10(7):e0131045. doi: 10.1371/journal.pone.0131045 (PMC4495923; doi:10.1371/journal.pone.0131045)
Supplement: S3 Table — (DOCX) [file pone.0131045.s006.docx]

**Table S3. Arcsin (square-root) transformed error proportions in Experiment 2**

| **c1** | **c2** | **c3** | **c4** | **c5** | **c6** | **c7** | **c8** | **c9** | **c10** | **c11** | **c12** | **c13** | **c14** | **c15** | **S** |
| --- | --- | --- | --- | --- | --- | --- | --- | --- | --- | --- | --- | --- | --- | --- | --- |
| .40 | .25 | .23 | .19 | .25 | .58 | .40 | .40 | .48 | .23 | .43 | .49 | .28 | .23 | .30 | 1 |
| .46 | .25 | .11 | .19 | .16 | .63 | .54 | .45 | .36 | .23 | .28 | .36 | .23 | .16 | .23 | 2 |
| .25 | .19 | .23 | .11 | .16 | .41 | .28 | .25 | .23 | .03 | .23 | .16 | .16 | .11 | .16 | 3 |
| .25 | .11 | .19 | .25 | .23 | .70 | .38 | .36 | .38 | .16 | .34 | .25 | .23 | .11 | .23 | 4 |
| .41 | .28 | .19 | .46 | .16 | .41 | .43 | .41 | .38 | .23 | .38 | .38 | .28 | .16 | .19 | 5 |
| .19 | .03 | .16 | .11 | .03 | .36 | .32 | .11 | .25 | .11 | .11 | .16 | .03 | .03 | .03 | 6 |
| .30 | .30 | .19 | .23 | .23 | .46 | .36 | .34 | .23 | .19 | .19 | .28 | .28 | .16 | .23 | 7 |

Key Row 1:

C1 4-paired sections 1:1.468 ratio

C2 4-paired sections 1:1.518 ratio

C3 4 paired sections 1:1.568 ratio

C4 4-paired sections 1:1.618 ratio

C5 4-paired sections 1:1.668 ratio

C6 8-paired sections 1:1.468 ratio

C7 8-paired sections 1:1.518 ratio

C8 8 paired sections 1:1.568 ratio

C9 8-paired sections 1:1.618 ratio

C10 8-paired sections 1:1.668 ratio

C11 16-paired sections 1:1.468 ratio

C12 16-paired sections 1:1.518 ratio

C13 16 paired sections 1:1.568 ratio

C14 16-paired sections 1:1.618 ratio

C15 16-paired sections 1:1.668 ratio

S = Participant number
