# Supplementary material for: The Golden Section as Optical Limitation
Source: PLoS One. 2015 Jul 8;10(7):e0131045. doi: 10.1371/journal.pone.0131045 (PMC4495923; doi:10.1371/journal.pone.0131045)
Supplement: S4 Table — (DOCX) [file pone.0131045.s007.docx]

**Table S4. Arcsin (square-root) transformed error proportions in Experiment 3**

| **C1** | **C2** | **C3** | **C4** | **C5** | **S** |
| --- | --- | --- | --- | --- | --- |
| .41 | .43 | .25 | .25 | .16 | 1 |
| .19 | .11 | .16 | .11 | .03 | 2 |
| .70 | .19 | .03 | .11 | .03 | 3 |
| .45 | .23 | .11 | .03 | .11 | 4 |
| .34 | .11 | .19 | .11 | .11 | 5 |
| .43 | .23 | .16 | .16 | .03 | 6 |
| .40 | .11 | .16 | .03 | .11 | 7 |
| .54 | .40 | .32 | .11 | .16 | 8 |
| .23 | .23 | .16 | .03 | .19 | 9 |
| .28 | .16 | .16 | .16 | .16 | 10 |

Key Row 1:

C1 8-paired sections 1:1.468 ratio

C2 8-paired sections 1:1.518 ratio

C3 8 paired sections 1:1.568 ratio

C4 8-paired sections 1:1.618 ratio

C5 8-paired sections 1:1.668 ratio

S = Participant number
